# Mitochondrial calcium uniporter-mediated mitochondrial dynamics imbalance contributes to contrast medium-induced renal tubular cell injury

**DOI:** 10.3389/fmolb.2026.1848361

**Published:** 2026-06-29

**Authors:** Yangyang Chen, Manyu Zhang, Sha Chen, Meiling Lv, Shuo Huang, Dingwei Yang

**Affiliations:** 1 School of Integrative Medicine, Tianjin University of Traditional Chinese Medicine, Tianjin, China; 2 Department of Nephrology, Tianjin Hospital, Tianjin University, Tianjin, China; 3 Clinical College of Orthopedics, Tianjin Medical University, Tianjin, China

**Keywords:** apoptosis, contrast medium, MCU, mitochondrial dynamics, mitochondrial dysfunction

## Abstract

**Background:**

Cellular and intramitochondrial calcium (Ca^2+^) overload, along with mitochondrial dysfunction, play a critical role in contrast-induced renal tubular epithelial cell injury. This study aims to clarify the role and mechanism of the Mitochondrial Calcium Uniporter (MCU) and mitochondrial dynamics in this process.

**Methods:**

Part 1: An *in vitro* CI-AKI model was established using human renal proximal tubular epithelial (HK-2) cells. The experimental design comprised a normal control group and iohexol-treated groups (100 mg I/mL) incubated for 4, 8, and 12 h, respectively. Part 2: To investigate the role of MCU, HK-2 cells were assigned to four conditions: normal control, iohexol alone, MCU inhibitor + iohexol group, and MCU agonist + iohexol group. We evaluated tubular cell injury and mitochondrial impairment, focusing on MCU expression, mitochondrial dynamics and Ca^2+^ loading, to clarify the molecular mechanisms.

**Results:**

Iohexol induced time-dependent cellular injury and apoptosis, accompanied by MCU upregulation, elevated intramitochondrial Ca^2+^ and mitochondrial dynamic imbalance. It also triggered mitochondrial membrane potential (ΔΨm) loss and mitochondrial reactive oxygen species (mtROS) accumulation. MCU inhibition with Ru360 enhanced cell viability, reduced apoptosis, improved mitochondrial function, suppressed Dynamin-related protein 1 (DRP1), reduced mitochondrial Ca^2+^, preserved ΔΨm and decreased mtROS. Conversely, MCU activation with spermine exacerbated the injury.

**Conclusion:**

Contrast-induced upregulation of MCU exacerbates intramitochondrial Ca^2+^ overload, preferentially promotes mitochondrial fission, resulting in the dissipation of ΔΨm and aggravated oxidative stress, which ultimately leads to cellular injury and apoptosis. Critically, inhibition of MCU conferred a protective effect against contrast-induced injury.

## 1 Introduction

Contrast-induced acute kidney injury (CI-AKI) is a frequent cause of hospital-acquired acute kidney injury. With the growing use of contrast-enhanced computed tomography and coronary angiography, the incidence of CI-AKI has been rising steadily (Guler et al., 2025; Deng et al., 2025). About 10%–12% of patients accepting Contrast Medium (CM) would suffer CI-AKI, causing serious complications including renal failure and even mortality (Mohammed et al., 2013; Strauß et al., 2025). Current epidemiological data further indicate that the incidence of CI-AKI among cardiac patients undergoing Percutaneous Coronary Intervention (PCI) ranges from approximately 2%–25%. In high-risk patients, such as those with chronic kidney disease or diabetes, the incidence can be even higher, potentially exceeding 50% (Campion et al., 2024; Watabe et al., 2014; Zhang L. et al., 2025; Li and Wang, 2024). Given its high incidence, elucidating the underlying pathogenesis is of critical importance.

Intracellular Ca^2+^ overload leading to mitochondrial injury represents a core mechanism in the pathophysiology of various diseases. Current research demonstrates its significant role across diverse fields, including cardiovascular diseases, neurodegenerative disorders, metabolic diseases, and cancer, and it has emerged as a potential therapeutic target (Bao et al., 2025; Yang X. et al., 2025; Li and Liu, 2024; Shi et al., 2025; Rozich et al., 2025). For instance, during cardiac ischemia-reperfusion, Ca^2+^ overload generates high levels of oxidative stress and causes a loss of mitochondrial integrity, ultimately leading to myocardial apoptosis and necrosis (Rozich et al., 2025; Liu L. et al., 2025). Our previous studies have confirmed that CM can induce intracellular Ca^2+^ overload and trigger apoptosis (Yang et al., 2013). Furthermore, mitochondrial dysfunction and morphological alterations are prevalent in CI-AKI, which further leads to the release of cytochrome c (Cyt c) and activation of the caspase family (Huang et al., 2020). Therefore, We propose that there is a Ca^2+^-dependent mechanism by which CM-induced disruption of mitochondrial homeostasis in renal tubular epithelial cells (RTECs).

Mitochondrial calcium uniporter (MCU), localized in the inner mitochondrial membrane (IMM), is the most important unidirectional channel responsible for Ca^2+^ influx into mitochondria (Guan et al., 2019). The function of MCU was studied more thoroughly in cardiac disease and neurological complications, namely, the regulation of mitochondrial Ca^2+^ homeostasis that is essential to ATP production and metabolism (Kwong et al., 2015; Williams et al., 2015). MCU is responsible for mitochondrial Ca^2+^ overload, opening of the mitochondrial permeability transition pores and cell death (Oropeza-Almazán et al., 2017). Downregulation of MCU by siRNA plays a protective role in cardiac ischemia/reperfusion (I/R) injury, whereas the upregulation of MCU aggravates cardiac lesion and neuropathy (Hamilton et al., 2021; George et al., 2022). However, the underlying mechanism that whether the function of MCU is involved in the CI-AKI remains unknown.

There is evidence from animal studies that other acute kidney injuries (AKI) induced by multiple pathogenic factors such as Ischemia Reperfusion Injury (IRI), cisplatin and sepsis are associated with mitochondrial dynamics, including fission and fusion (Tang et al., 2021; Xie et al., 2023; Zhang et al., 2024). Mitochondrial dynamics, an essential component of mitochondrial quality control, is responsible for maintaining the relative stability of mitochondrial quantity, morphology and function, that is, a state known as mitochondrial homeostasis. Recently, the Liu et al. demonstrated that Mdivi-1, a specific DRP1 inhibitor, mitigates the risk of acute kidney injury and preserves renal function by suppressing excessive mitochondrial fission (Liu J. et al., 2025). To the best of our knowledge, the underlying mechanisms of mitochondrial quality control in CI-AKI remain poorly understood. We hypothesize that CM can interact with and activate the MCU on the mitochondrial membrane. The MCU mediates Ca^2+^ influx into mitochondria, which in turn induces mitochondrial fission, suppresses mitochondrial fusion, and disrupts mitochondrial dynamics, leading to mitochondrial dysfunction and ultimately triggering apoptosis. Thus, this study aims to elucidate the link between the MCU, mitochondrial dynamics, and mitochondrial homeostasis *in vitro* model of CI-AKI. HK-2 cells and iohexol were used to simulate contrast-induced renal tubular epithelial cells (RTECs) injury.

## 2 Materials and methods

### 2.1 Reagents and antibodies

Human renal proximal tubular epithelial (HK-2) cells (GDC0152) were purchased from the China center for type culture collection (Beijing, China). DMEM/F12 medium (PM150312) was obtained from Procell (Wuhan, China). Fetal bovine serum (FBS; 10437028) and penicillin-streptomycin antibiotics (15140122) were obtained from Gibco (NY, United States). Iohexol (300 mg iodine/mL) was purchased from Beilu Pharmaceutical Co. (Beijing, China). We obtained Cell Counting Kit 8 (WST-8/CCK8) (ab228554), anti-OPA1 (ab157457), anti-DRP1 (ab184248) antibodies and fluorescent Ca^2+^ indicator (Rhod-2 AM) (ab142780) from Abcam (Cambridge, United Kingdom). Antibodies against MCU (26312-1-AP) were obtained from Proteintech (Chicago, United States). Bata Tubulin (YM3431) were obtained from Immunoway (Texas, United States). Mitotracker red (M9940) was obtained from Solarbio Life sciences (Beijing, China). Ru360 (an inhibitor of MCU, 557,440-500UG) and Spermine (an agonist of MCU, S3256-5G) were purchased from Sigma-Aldrich (St. Louis, United States). Annexin V-FITC apoptosis staining kit (C1062M) and JC-1 mitochondrial membrane potential assay kit (C2005) were obtained from Beyotime (Shanghai, China). MitoSOX Red (HY-D1055) was obtained from MedChemExpress (Monmouth Junction, NJ, United States).

### 2.2 Cell culture and treatment

HK-2 cells were cultured in DMEM/F12 medium containing 10% FBS and 1% penicillin-streptomycin antibiotics at 37 °C with 5% CO_2_. Cells were used after at least five passages. To eliminate batch effects and variations in culture duration, cells were seeded using a reverse time-point plating method, ensuring that all cells were simultaneously seeded, harvested, stained, and analyzed. Each experimental group was tested with three biological replicates. **Part 1:** To develop a CI-AKI model *in vitro*, HK-2 cells were treated with iohexol at a concentration of 100 mg I/mL. The experimental design comprised a normal control group and iohexol-treated groups, incubated for 4, 8, and 12 h, respectively. Subsequently, cell viability, apoptosis, mitochondrial morphology and function, as well as expression levels of the MCU and mitochondrial dynamics-related proteins were assessed. These analyses aimed to identify an optimal treatment duration for establishing the model group in subsequent experiments investigating the role of MCU. **Part 2:** To examine the effect of MCU intervention on CM-induced injury, HK-2 cells were divided into four groups: Normal control group, Iohexol-treated group (exposed to iohexol for 8 h),MCU inhibitor + iohexol group (pretreated with 10 μM Ru360 1 h before iohexol exposure), MCU agonist + iohexol group (pretreated with 10 μM spermine 1 h before iohexol exposure). This experimental setup allowed evaluation of how MCU inhibition or activation influences CM-induced HK-2 cell injury, mitochondrial abnormalities, and dynamics.

### 2.3 Cell viability and cell apoptosis

The cell viability assay was measured by using Cell Counting Kit-8 (CCK-8) kit according to the manufacturer’s instructions and the OD values of 450 nm were read by the multifunctional microplate spectrophotometer Thermo 3001 (Thermo Fisher Scientific, United States). The values of cell viability were calculated as a percentage of control. Cells were seeded in 96-well plates at a density of 2 × 10^3^ cells per well in 100 μL of culture medium. After cell attachment, cells were treated according to the experimental groups, with three replicate wells per group. Following a 24 h incubation, 10 μL of CCK-8 solution was added to each well, and the plates were incubated for an additional 4 h at 37 °C with 5% CO_2_. The absorbance (optical density, OD) at 450 nm was measured using a microplate reader. Cell viability was then calculated using the following formula: Cell viability (%) = [(As–Ab)/(Ac–Ab)] × 100%, where As represents the absorbance of experimental wells, Ac represents the absorbance of control wells, and Ab represents the absorbance of blank wells. Apoptosis of HK-2 cells was detected through flow cytometry (CyFlow®Cube 6, Sysmex, Japan) using the Annexin V-FITC/PI double detection kit (Beyotime, Shanghai, China). Briefly, HK-2 cells were washed with cold PBS, centrifuged, and resuspended in 400 µL binding buffer including 5 µL annexin V-FITC and 10 µL PI, and then they were prepared to be analyzed with flow cytometry. Mostly Annexin V+/PI- cells and Annexin V+/PI + cells, which indicate early and late apoptotic cells, were both considered apoptosis, while Annexin V-/PI + cells were considered necrosis. Data was analyzed using FlowJo Software (Tree Star Inc., Ashland, OR).

### 2.4 Detection of mitochondrial membrane potential (MMP) by JC-1

MMP was detected by staining the cells with JC-1 fluorescence dye, which was added to each sample with a final concentration of 300 nM and incubated for 20 min at 37 °C in a culture incubator. Then the supernatants were discarded, and the cells were resuspended by JC-1 staining buffer and culture medium. Fluorescence intensities were recorded by Olympus inverted fluorescence microscope (CKX31-A12PHP, Japan) and data was analyzed through ImageJ. The level of MMP was reflected as the ratio of red/green fluorescence areas.

### 2.5 Fluorescence quantitative detection of the Ca^2+^ concentration in mitochondria

Mitochondrial Ca^2+^ specific fluorescence was detected by the mitochondrial Ca^2+^ fluorescent probe (Rhod-2 AM), which can combine with Ca^2+^ and cause a remarkable increase in fluorescence intensity. Briefly, cells were washed twice with plain DMEM/F12 and then incubated with Rhod-2 AM in medium for 15 min at 37 °C in incubator under the manufacturer’s instructions. Then cells were washed three times with Hanks’ balanced salt solution (HBSS). Cells were then immediately subjected to Carl Zeiss LSM710 confocal laser-scanning microscope (Jena, Germany).

### 2.6 Mitotracker for the morphology of mitochondria by using the confocal microscopy

HK-2 cells cultured on sterile round coverslips (18 mm diameter) were stained with 150 nM MitoTracker Red CMXRos (M9940, Beyotime) at 37 °C for 30 min in the dark. Followed three washes with pre-warmed culture medium, cells were fixed with 4% paraformaldehyde for 15 min and stained with 10 μg/mL DAPI for 5–10 min. The coverslips were then mounted using an antifade mounting medium. Fluorescence images were acquired using a confocal laser scanning microscope with 405 nm (DAPI) and 561 nm (MitoTracker) lasers and a 60× oil-immersion objective. Mitochondrial morphology was analyzed using the Mitochondria Analyzer plugin. The main parameters for the 2D Threshold were set as follows: block size, 1.35 µm; C-value, 5. Subsequent analyses in 2D Analyses were conducted using the default settings. Perimeter was used to evaluate mitochondrial length, while Form Factor (FF) and Aspect Ratio (AR) were used to evaluate mitochondrial roundness. Form Factor close to 1 indicates a circular shape, whereas a higher Aspect Ratio indicates a more elongated morphology.

### 2.7 Western blot of the relevant protein

HK-2 cells were lysed with cell lysis buffer (RIPA: PMSF = 100: 1, The final concentration of PMSF was 1 mM. Beyotime Biotechnology, China) on ice for 30 min and then centrifuged at 13,200 rpm for 15 min at 4 °C. The protein concentration was determined by BCA protein assay reagent kit (Beyotime Biotechnology, China). Then 30 μg of protein per sample was separated on 12% sodium dodecyl sulfate (SDS)-polyacrylamide gel electrophoresis (PAGE) and transferred to polyvinylidene difluoride (PVDF) membrane. Membranes were blocked on a shaker at room temperature (RT) with 5% non-fat milk for 2 h, followed by incubation with primary antibodies anti-MCU, anti-OPA1, and anti-DRP1 overnight at 4 °C. The blot membrane was rinsed with TBST 3 times for 5 min on the shaker and subsequently incubated with goat anti-rabbit HRP-conjugated second antibodies for 1 h at RT. Chemiluminescence was imaged in a Molecular Imager® Chemi DocTM XRS + with Image LabTM software (Bio-Rad, CA, United States).

### 2.8 Measurement of mitochondrial superoxide with MitoSOX red

Cells were plated on coverslips in 24-well plates at an appropriate density and cultured overnight to allow adhesion. After treatment according to experimental groups, cells were incubated with 5 μM MitoSOX Red working solution (prepared by diluting the stock solution in PBS) at 37 °C for 10 min in the dark to label mitochondrial superoxide. Following incubation, the cells were gently washed three times with pre-warmed PBS. Nuclei were then counterstained with DAPI, followed by an additional three PBS washes. Finally, coverslips were mounted using an antifade mounting medium, and images were acquired using a fluorescence microscope.

### 2.9 Transmission electron microscopy (TEM)

Cultured HK-2 cells were rinsed with PBS and gently scraped off. The cells were collected in a 1.5 mL centrifuge tube and centrifuged at 1,000–1,500 rpm for 5 min. The supernatant was removed without disturbing the cell pellet. The pellet was fixed in pre-cooled 3% glutaraldehyde at 4 °C for 2 h. After removing the fixative, the pellet was post-fixed with 1% osmium tetroxide at 4 °C for 1 h. The pellet was then dehydrated through a graded acetone series, followed by infiltration and embedding in epoxy resin. After polymerization, ultrathin sections (60–90 nm) were cut using an ultramicrotome and sequentially stained with uranyl acetate and lead citrate. Finally, the sections were examined with a Hitachi H-7650 transmission electron microscope (Tokyo, Japan).

### 2.10 Statistical analysis

We used GraphPad Prism 10.4.1 to analyze statistical data that were expressed as means ± SD. Statistical comparisons were determined using one-way analysis of variance (ANOVA) when comparing three or more groups, followed by Tukey’s multiple comparisons test for *post hoc* pairwise comparisons to control the familywise error rate All statistical results were derived from at least three independent biological replicates, denoted as n. P < 0.05 was regarded as statistically significant.

## 3 Results

### 3.1 Iohexol treatment led to decreased cell viability and increased apoptosis in HK-2 cells

**FIGURE 1:**
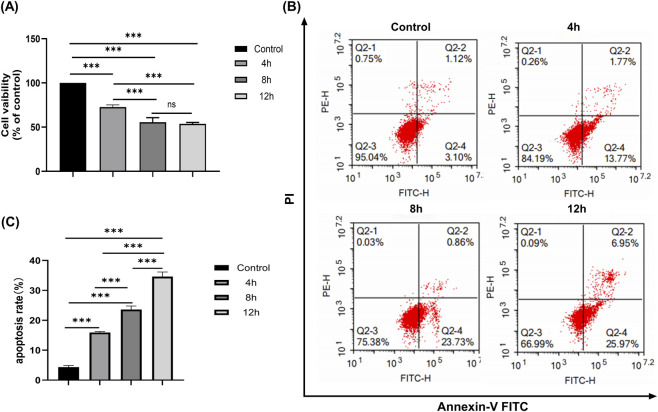
**(A)** Cell viability was determined by CCK-8 assay, which was shown as the percentage of controls (n = 3). **(B,C)** The iohexol-induced apoptosis was determined by flow cytometry and quantitation analysis (n = 3). Data were representative of at least three independent biological replicates (n = 3) and expressed as the means ± SD. Statistical analysis was performed using one-way ANOVA followed by Tukey’s multiple comparisons test. *P < 0.05; **P < 0.01; ***P < 0.001.

To simulate CM-induced kidney injury and investigate the underlying mechanism of CM-induced RTEC injury, HK-2 cells were incubated with a proper concentration (100 mg I/mL) of iohexol for varying durations (0, 4, 8, and 12 h). In Figure 1A, the CCK-8 assay showed that iohexol induced a time-dependent reduction in cell viability of HK-2 cells (Control vs. 4 h, 100% ± 0% vs. 72.63% ± 2.51%; Control vs. 8 h, 100% ± 0% vs. 55.61% ± 5.07%; Control vs. 12 h, 100% ± 0% vs. 53.73% ± 1.58%; P < 0.0001). According to Figures 1B,C, the average apoptosis rate of HK-2 cells increased significantly following iohexol treatment (Control vs. 4 h, 4.35 %± 0.54% vs. 15.92% ± 0.34%, P < 0.0001; Control vs. 8 h, 4.35 %± 0.54% vs. 23.62% ± 1.15%, P < 0.0001; Control vs. 12 h, 4.35 %± 0.54% vs. 34.63% ± 1.53%, P < 0.0001), indicating that iohexol induces apoptosis in HK-2 cells in a time-dependent manner within a certain range.

### 3.2 Iohexol induced mitochondrial injury, high MCU expression and disrupted mitochondrial dynamics homeostasis

#### 3.2.1 Iohexol-induced mitochondrial injury

**FIGURE 2:**
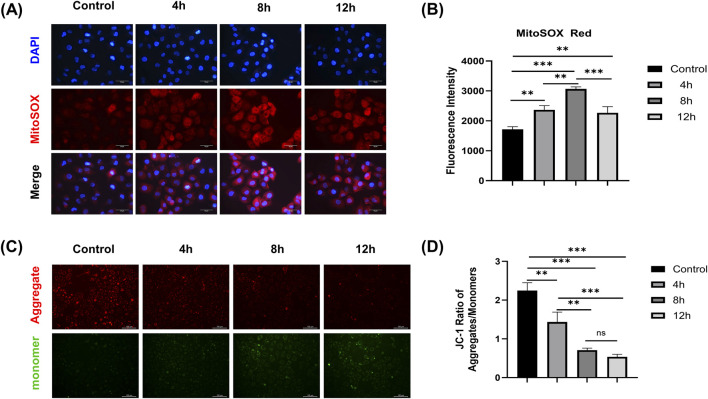
**(A,B)** ROS generation was visualized by MitoSOX Red fluorescent staining (n = 3). Excitation (Ex)/Emission (Em): 396/610 nm. Magnification: ×400, Scale bar: 50 µm. **(C,D)** MMP was detected by JC-1, and the fluorescence of JC-1 was recorded on the fluorescence microscope (n = 3). The changes of MMP were reflected by the aggregates (red fluorescence)/monomers (green fluorescence) ratio. Magnification: ×200, Scale bar: 100 µm. Data were analyzed using one-way ANOVA and followed by Tukey’s multiple comparisons test. Data were representative of at least three independent biological replicates (n = 3) and expressed as the means ± SD. *P < 0.05; **P < 0.01; ***P < 0.001.

To evaluate the impact of the CM on mitochondria, MitoSOX Red fluorescent staining was used to assess mitochondrial superoxide levels. As shown in Figures 2A,B, HK-2 cells treated with iohexol exhibited a time-dependent increase in red fluorescence intensity, which peaked at 8 h. These findings indicate elevated mitochondrial superoxide production and increased mitochondrial reactive oxygen species (ROS), reaching a maximum at the 8-h time point (Control vs. 8 h, 1716% ± 89.17% vs. 3068% ± 66.86%, P < 0.0001). Mitochondrial membrane potential (ΔΨm) was assessed using the JC-1 fluorescent probe. A shift in JC-1 fluorescence from red to green serves as an indicator of early apoptosis, typically reflecting a loss of membrane potential and mitochondrial impairment. Our data in Figures 2C,D, the red-to-green fluorescence ratio was significantly reduced in iohexol-treated cells compared with the control group (Control vs. 4 h, 2.25% ± 0.20% vs.1.44% ± 0.25%, P = 0.0015; Control vs. 8 h, Control vs. 12 h, 2.25% ± 0.20% vs. 0.71% ± 0.05%, vs. 0.54% ± 0.07%, P < 0.0001).

#### 3.2.2 Iohexol-induced high MCU expression and disruption of mitochondrial dynamics homeostasis

**FIGURE 3:**
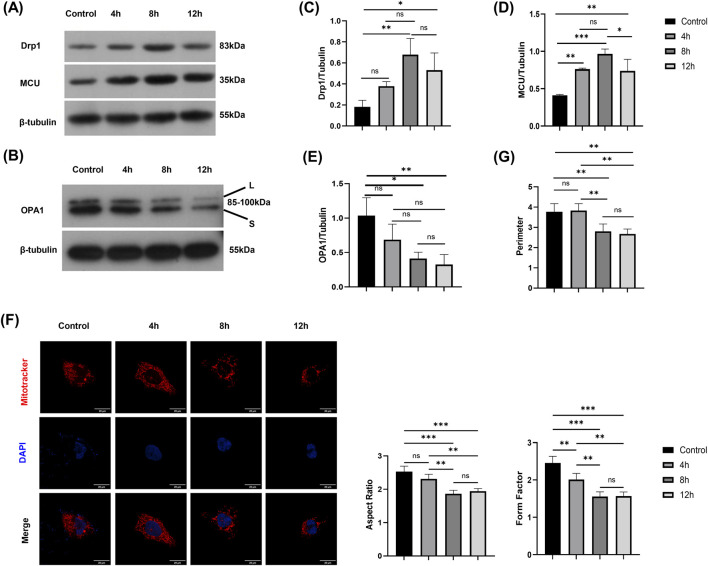
**(A,C,D)** Western blot analysis and quantification of the expression of MCU and DRP1 (n = 3). **(B,E)** Western blot analysis and quantification of the expression of OPA1 (n = 3). **(F,G)** Mitochondrial morphology was observed using Mitotracker red staining, and the fragmentation of mitochondria was analyzed using ImageJ, including the Perimeter, Form Factor (FF) and Aspect Ratio (AR). (n = 3). Scale bar: 20 µm. Data were analyzed using one-way ANOVA and followed by Tukey’s multiple comparisons test. Data were representative of at least three independent biological replicates (n = 3) and expressed as the means ± SD. *P < 0.05; **P < 0.01; ***P < 0.001.

Then, we examined the expression of MCU, DRP1 and OPA1 by Western blot. Our results in Figures 3A,D, prolonged exposure of HK-2 cells to iohexol resulted in a time-dependent increase in the expression of the MCU, which peaked at 8 h with statistical significance (Control vs. 4 h, 0.41% ± 0.01% vs. 0.76% ± 0.01%, P = 0.0041; Control vs. 8 h, 0.41% ± 0.01% vs. 0.97% ± 0.07%, P = 0.0002; Control vs. 12 h, 0.41% ± 0.01% vs. 0.74% ± 0.15%, P = 0.0062). Concurrently, the expression of DRP1 was significantly elevated after 8 and 12 h of treatment (Figures 3A–C: Control vs. 8 h, 0.18% ± 0.06% vs. 0.68% ± 0.15%, P = 0.0040; Control vs. 12 h, 0.18% ± 0.06% vs. 0.53% ± 0.16%, P = 0.0287). In contrast, the fusion-related protein OPA1 was markedly downregulated at 4, 8, and 12 h (Figures 3B–E; Control vs. 8h, 1.04% ± 0.26% vs. 0.41% ± 0.09%, P = 0.0175; Control vs. 12 h, 1.04% ± 0.26% vs. 0.33% ± 0.15%, P = 0.0085). We simultaneously used Mitotracker Red to trace mitochondria and further analyzed changes in mitochondrial morphology. Mitotracker Red fluorescent staining (Figures 3F,G) showed that the mitochondrial morphology changed from an elongated network into small spheres or short rods after iohexol treatment and significantly shortened the mean mitochondrial perimeter (Control vs. 4 h, 3.76% ± 0.40% vs. 3.83% ± 0.35%, P > 0.99; Control vs. 8 h, 3.76% ± 0.40% vs. 2.81% ± 0.36%, P = 0.007; Control vs. 12 h, 3.76% ± 0.40% vs. 2.67% ± 0.24%, P = 0.002). The values of both form factors and aspect ratio were reduced, with the most significant reduction observed at 8 h (aspect ratio: Control vs. 8 h, 2.53% ± 0.16% vs. 1.87% ± 0.11%, P < 0.001; form factors: Control vs. 8 h, 2.46% ± 0.18% vs. 1.56% ± 0.12%, P < 0.001), indicating progressive mitochondrial morphological shortening or mitochondrial network fragmentation following iohexol exposure, with the most severe fragmentation occurring at 8 h. These findings suggest that iohexol disrupts the balance between fission and fusion by promoting fission and inhibiting fusion, thereby impairing normal mitochondrial morphology and leading to excessive mitochondrial fragmentation.

### 3.3 Upregulation of MCU by iohexol induces mitochondrial Ca^2+^ overload

**FIGURE 4:**
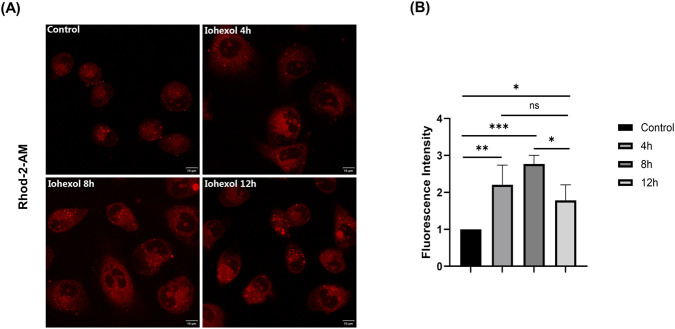
**(A,B)** HK-2 cells were labeled with Rhod-2 as described in the materials and methods part to express the content of mitochondrial Ca^2+^ and then subjected to the lapse confocal. Representative images were shown. The fluorescence intensities were quantified and normalized (n = 3). Magnification: ×1000, Scale bar: 10 µm. Data were representative of at least three independent biological replicates (n = 3) and expressed as the means ± SD. Statistical analysis was performed using one-way ANOVA and followed by Tukey’s multiple comparisons test. *P < 0.05; **P < 0.01; ***P < 0.001.

Mitochondrial calcium levels were assessed using the Rhod-2 AM fluorescent probe, where an increase in red fluorescence intensity indicates a rise in calcium concentration, serving as another indicator of mitochondrial dysfunction. As indicated in Figures 4A,B, the red fluorescence intensity of Ca^2+^ in RTEC strengthened with prolonged iohexol exposure, peaking most significantly at 8 h (Control vs. 8 h, 1.00% ± 0.0% vs. 2.77% ± 0.24%, P < 0.0001). Thus, iohexol treatment leads to increased MCU expression and subsequent mitochondrial calcium overload. In summary, it can be inferred that MCU upregulation is an upstream event in mitochondrial damage and cellular apoptosis. Furthermore, since several experiments indicated that mitochondrial injury and dynamics alterations were most pronounced at the 8-h time point, this duration was selected as the optimal stimulation time for iohexol in subsequent experiments.

### 3.4 MCU was involved in iohexol-induced cell damage and cell apoptosis

**FIGURE 5:**
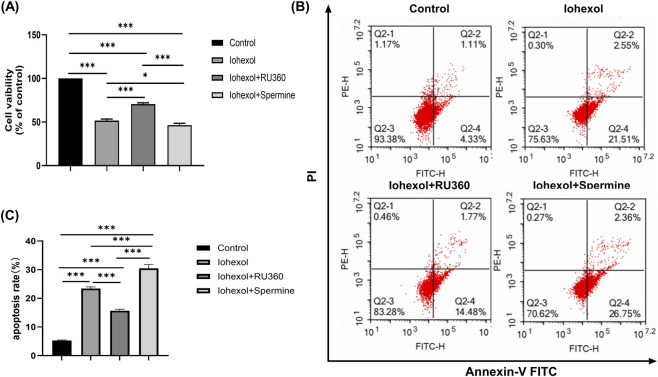
**(A)** Cell viability was determined by CCK-8 assay (n = 3). **(B,C)** The apoptosis distribution was determined by flow cytometry and the analysis of quantitation (n = 3). Data were representative of at least three independent biological replicates (n = 3) and expressed as the means ± SD. Statistical analysis was performed using one-way ANOVA and followed by Tukey’s multiple comparisons test. *P < 0.05; **P < 0.01; ***P < 0.001.

Based on the collective experimental evidence, we propose that increased MCU expression and the resultant mitochondrial Ca^2+^ overload represent an upstream event in mitochondrial damage and cellular apoptosis. To investigate whether MCU participates in iohexol-induced RTEC injury, this study employed Ru360 (10 µM) to inhibit and spermine (10 µM) to activate MCU function in HK-2 cells. Both agents were administered 1 hour prior to iohexol exposure. CCK-8 assay results (Figure 5A) revealed a significant decrease in cell viability in the iohexol group, which was markedly rescued by Ru360 pretreatment (Iohexol vs. Iohexol + Ru360, 51.65% ± 1.82% vs. 70.60% ± 1.47%, P < 0.0001). Conversely, spermine pretreatment further reduced cell viability (Iohexol vs. Iohexol + Spermine, 51.65% ± 1.82% vs. 46.46% ± 2.15%, P = 0.0167). Consistent with this, flow cytometry analysis (Figures 5B,C) indicated that iohexol induced significant apoptosis, which was attenuated by Ru360 pretreatment (Iohexol vs. Iohexol + Ru360, 23.42% ± 0.56% vs. 15.68% ± 0.53%, P < 0.0001). In contrast, the MCU agonist spermine further increased the apoptotic rate (Iohexol vs. Iohexol + Spermine, 23.42% ± 0.56% vs. 30.47% ± 1.29%, P < 0.0001). These results demonstrate that MCU contributes to iohexol-induced HK-2 cell injury and apoptosis, and that inhibiting MCU function effectively mitigates the detrimental effects of iohexol.

### 3.5 MCU mediates iohexol-induced disruption of mitochondrial dynamics and Ca^2+^ uptake in HK-2 cells

**FIGURE 6:**
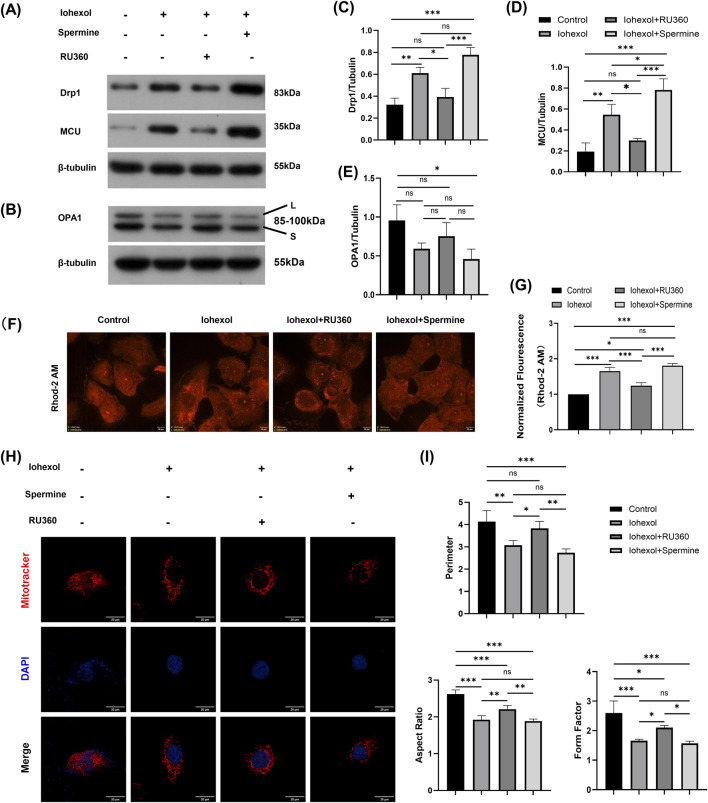
**(A,C,D)** Western blot analysis and quantification of the expression of MCU and DRP1 (n = 3). **(B,E)** Western blot analysis and quantification of the expression of OPA1(n = 3). **(F,G)** HK-2 cells were labeled with Rhod-2 as described in the materials and methods part to express the content of mitochondrial Ca^2+^ and then subjected to the lapse confocal. Representative images were shown. The fluorescence intensities were quantified and normalized (n = 3). Magnification: ×1000, Scale bar: 10 µm. **(H,I)** Mitochondrial morphology was observed using Mitotracker red staining, and the fragmentation of mitochondria was analyzed using ImageJ, including the Perimeter, Form Factor (FF) and Aspect Ratio (AR). (n = 3). Scale bar: 20 µm. Data were representative of at least three independent biological replicates (n = 3) and expressed as the means ± SD. Statistical analysis was performed using one-way ANOVA and followed by Tukey’s multiple comparisons test. *P < 0.05; **P < 0.01; ***P < 0.001. Note: No statistically significant differences were observed between the Iohexol group and the Iohexol + Spermine group for the expression of DRP1 and OPA1, mitochondrial Ca^2+^ uptake, and Mitotracker Red staining (Perimeter, Form Factor and Aspect Ratio). Additionally, OPA1 expression did not differ significantly between the Iohexol group and the Iohexol + RU360 group. These non-significant comparisons are denoted as “ns” in the figures.

We verified the protein expression of MCU by Western blot and drew the conclusion that the MCU can indeed be upregulated by spermine and downregulated by Ru360 in iohexol-treated cells. (Figures 6A,D: Iohexol vs. Iohexol + Spermine, 0.55% ± 0.10% vs. 0.78% ± 0.11%, P = 0.0370; Iohexol vs. Iohexol + Ru360, 0.55% ± 0.10% vs. 0.30% ± 0.02%, P = 0.0287). As depicted in Figures 6F,G, through Rhod-2 staining, we found that the Iohexol + Ru360 group exhibited a lower level of mitochondrial calcium uptake compared to the iohexol group (1.65% ± 0.11% vs. 1.24% ± 0.09%, P = 0.0008). Conversely, although an increasing trend was observed in the Iohexol + Spermine group, it did not reach statistical significance.

To investigate the role of MCU in iohexol-induced mitochondrial dynamic disorder in HK-2 cells, we evaluated the expression levels of mitochondrial dynamic-associated proteins, namely, DRP1 and OPA1, under different levels of MCU expression. As shown in Figures 6A,C, compared with the iohexol-alone group, co-treatment with Ru360 downregulated DRP1 expression, whereas co-treatment with iohexol and spermine only showed an increasing trend but the difference was not statistically significant (Iohexol vs. Iohexol + Spermine, 0.61% ± 0.05% vs. 0.78% ± 0.07%, P = 0.0550; Iohexol vs. Iohexol + Ru360, 0.61% ± 0.05% vs. 0.39% ± 0.08%, P = 0.0147), suggesting that inhibited MCU activity reduces mitochondrial fission. However, further activation of MCU by spermine did not observably exacerbate contrast agent-induced mitochondrial fission. Concurrently, the iohexol-induced downregulation of OPA1 expression was further decreased by spermine treatment and partially rescued by RU360 treatment; however, none of these changes reached statistical significance (Figures 6B,E: Control vs. Iohexol, 0.96% ± 0.20% vs. 0.59% ± 0.07%, P = 0.0753; Iohexol vs. Iohexol + Ru360, 0.59% ± 0.07% vs. 0.75% ± 0.18%, P = 0.2955; Iohexol vs. Iohexol + Spermine, 0.59% ± 0.07% vs. 0.46% ± 0.13%, P = 0.7197), suggesting that both inhibition and activation of MCU exert minimal impact on OPA1 expression. MitoTracker Red staining (Figures 6H,I) revealed that HK-2 cells co-treated with spermine and iohexol reduced the mean mitochondrial perimeter, form factor, and aspect ratio compared to the iohexol-only group, but it did not reach statistical significance (Iohexol vs. Iohexol + Spermine, perimeter: 3.07% ± 0.21% vs. 2.73% ± 0.17%, P = 0.47; aspect ratio: 1.92% ± 0.11% vs. 1.89% ± 0.06%, P = 0.95; form factor: 1.66% ± 0.05% vs. 1.57% ± 0.07%, P = 0.93). In contrast, co-treatment with Ru360 significantly ameliorated iohexol-induced mitochondrial fragmentation (Iohexol vs. Iohexol + Ru360, perimeter: 3.07% ± 0.21% vs. 3.91% ± 0.34%, P = 0.02; aspect ratio: 1.92% ± 0.11% vs. 2.21% ± 0.10%, P = 0.007; form factor: 1.66% ± 0.05% vs. 2.13% ± 0.10%, P = 0.04). In summary, these data preliminarily indicate that MCU contributes to iohexol-induced imbalance in mitochondrial dynamics and increased mitochondrial fragmentation by increasing the fission-related protein under the conditions of this study.

### 3.6 The impact of MCU on iohexol-induced mitochondrial injury in HK-2 cells

**FIGURE 7:**
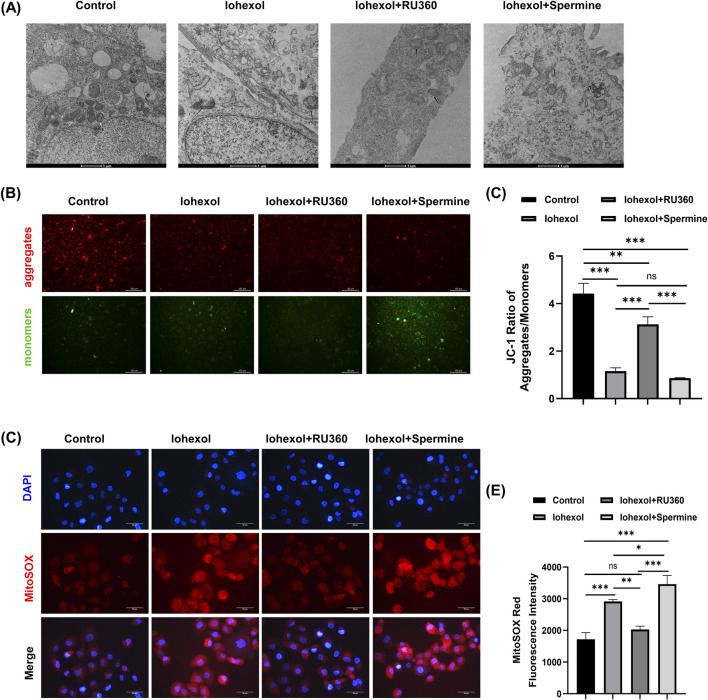
**(A)** Representative images of transmission electron microscope micrographs of mitochondria in HK-2 cells (n = 3). Scale bar: 1 µm. White arrows: normal mitochondria, black arrows: damaged mitochondria. **(B,C)** MMP was detected by JC-1, and the fluorescence of JC-1 was recorded on the fluorescence microscope (n = 3). The changes of MMP were reflected by the aggregates (red fluorescence)/monomers (green fluorescence) ratio. Magnification: ×200. Scale bar: 100 µm. **(D,E)** ROS generation was visualized by MitoSOX Red fluorescent staining (n = 3). Ex/Em: 396/610 nm. Magnification: ×400, Scale bar: 50 µm. Data were representative of at least three independent biological replicates (n = 3) and expressed as the means ± SD. Statistical analysis was performed using one-way ANOVA and followed by Tukey’s multiple comparisons test. *P < 0.05; **P < 0.01; ***P < 0.001. Note: Variation in mitochondrial profile size within the image is largely due to random sectioning. The area of an individual profile does not directly represent mitochondrial volume.

Alterations in mitochondrial morphology and a decrease in the red-to-green fluorescence ratio of JC-1 staining are important marks of mitochondrial damage. TEM (Figure 7A) revealed that, in the Iohexol + spermine group at high magnification, mitochondria exhibited marked matrix dissolution, disruption and loss of cristae, as well as swelling and even rupture. In contrast, the Iohexol + Ru360 group showed attenuated structural damage compared to the iohexol-alone group, although mitochondrial morphology did not fully revert to the normal state observed in controls. Assessment of mitochondrial function using the JC-1 probe (Figures 7B,C) demonstrated a slightly lower red-to-green fluorescence ratio in the Iohexol + Spermine group compared to the iohexol group (1.16% ± 0.14% vs. 0.86% ± 0.02%, P = 0.5842), but it did not reach statistical significance. Conversely, the Iohexol + Ru360 group exhibited a markedly higher ratio (1.16% ± 0.14% vs. 3.13% ± 0.32%, P = 0.0001). Furthermore, MitoSOX Red fluorescent staining (Figures 7D,E) showed that the Iohexol + Spermine group displayed increased red fluorescence intensity compared to the iohexol group (2914% ± 59.31% vs. 3462% ± 273.2%, P = 0.0271), suggesting elevated mitochondrial superoxide production and an increase in mtROS. The Iohexol + Ru360 group, however, showed a significant reduction in fluorescence intensity (2914% ± 59.31% vs. 2027% ± 107.9%, P = 0.0016). These results collectively demonstrate that MCU mediates iohexol-induced mitochondrial damage. The above studies preliminarily revealed that MCU inhibition appears to protect mitochondria from contrast media-induced damage through the attenuation of mitochondrial calcium overload and dynamics disruption.

## 4 Discussion

CI-AKI has become the third most common reason for hospital-acquired AKI (Fähling et al., 2017). Our team has confirmed the involvement of elevated intracellular Ca^2+^ concentration (Yang et al., 2013) and mitochondrial dysfunction in the pathogenesis of CI-AKI in rats, accompanied by observations of disrupted mitochondrial morphology and substantial release of reactive oxygen species (ROS) (Huang et al., 2020). This study further elucidated one of the mechanisms of CI-AKI from the perspective of mitochondria. MCU was upregulated in CM-induced RTEC injury, which increased Ca^2+^ intake of mitochondria. Mitochondrial Ca^2+^ overload promoted mitochondrial fission, leading to mitochondrial dysfunction and inducing apoptosis.

Intracellular Ca^2+^ overload has been identified to be one of the primary factors responsible for contrast-induced proximal tubular epithelial cell toxicity (Yang et al., 2013; Yamashita et al., 2001). Our group’s prior findings further support that upregulated expression of the Na+/Ca2+ exchanger (NCX) in RTECs following contrast exposure leads to intracellular Ca^2+^ overload, which subsequently induces mitochondrial injury and apoptosis (Yang et al., 2013). However, the relationship between aberrantly elevated intracellular Ca^2+^ and mitochondrial damage, along with its underlying mechanisms, remains incompletely defined. Therefore, this study specifically focuses on the role of MCU. MCU serves as the primary channel for unidirectional Ca^2+^ transfer from the cytosol into the mitochondrial matrix. Dysregulation of MCU function, leading to mitochondrial Ca^2+^ overload, is a critical pathological mechanism in the development and progression of various diseases, involving the regulation of cellular energy metabolism, ROS generation, and programmed cell death (Amjid et al., 2024; Cui et al., 2019; Weiser et al., 2023; D'Angelo and Rizzuto, 2023). Recent research has identified Ca^2+^ overload in renal tubular cells of the aging kidney, concomitant with a significant upregulation of MCU expression. Agonism of MCU significantly increases mitochondrial Ca^2+^ accumulation, induces elevated ROS production, and promotes renal tubular cell senescence and renal fibrosis. Conversely, suppressing MCU reduces ROS generation, restores mitochondrial homeostasis, delays cellular senescence, and confers protection against the detrimental effects of kidney aging (Xiong et al., 2024). Yuan et al. demonstrated that in palmitic acid-induced podocyte apoptosis, MCU expression was elevated, concomitant with increased mitochondrial Ca^2+^ concentration and cytochrome c (Cyt c) release. Exogenous MCU antagonism suppressed mitochondrial Ca^2+^ overload and ameliorated the podocyte apoptosis induced by palmitic acid (Yuan et al., 2017). Additionally, MCU expression is significantly upregulated in patients with heart failure, driving mitochondrial Ca^2+^ overload that contributes to energetic dysfunction and pathological cardiac remodeling (Liu et al., 2020). The Liu team found that silencing MCU enhanced cell viability and protected against impaired Ca^2+^ handling capacity. Conversely, MCU overexpression enhanced mitochondrial Ca^2+^ uptake to promote the growth and proliferation of colorectal cancer (CRC) cells (Yu et al., 2025). Consistent with previous findings, our current study observed that following contrast agent stimulation, there was not only significant HK-2 cell injury and apoptosis but also a concomitant upregulation of MCU expression and mitochondrial Ca^2+^ overload. Rhod-2 AM is a mitochondrion-targeted fluorescent probe for calcium ions. It is initially non-fluorescent upon entry into cells but is subsequently hydrolyzed by esterases to yield Rhod-2. Due to its positive charge, the probe predominantly accumulates within mitochondria. Its fluorescence intensity is directly proportional to the mitochondrial calcium concentration and is currently regarded as one of the gold standards for directly measuring mitochondrial calcium levels (Shen et al., 2025; Fu et al., 2026; Chen et al., 2023). These findings suggest that MCU likely serves as the pivotal mediator of CM-induced mitochondrial Ca^2+^ overload and is a primary initiating event for the subsequent disruption of mitochondrial dynamics and functional impairment, thereby contributing to the pathogenesis of CI-AKI.

MCU is responsible for regulating Ca^2+^ influx into the mitochondrial matrix. Its aberrant activation can induce mitochondrial Ca^2+^ overload, which in turn triggers a series of mitochondrial injury responses, including increased ROS production, loss of mitochondrial membrane potential and disruptions in mitochondrial dynamics (Guan et al., 2019; Yu et al., 2025; Mannella et al., 2025; Gray et al., 2025; Boyman et al., 2020; Qin et al., 2023). As previously mentioned, we observed that in iohexol-treated RTECs, upregulation of MCU expression was accompanied by notable epithelial damage and apoptosis. Concurrently, the study also noted an imbalance in mitochondrial dynamics characterized by excessive fission and functional impairment. To further investigate the functional role of MCU in CI-AKI, we employed Ru360 and spermine to inhibit and activate MCU, respectively. Ru360 and spermine are currently recognized as the most used exogenous inhibitor and endogenous activator of the MCU, respectively. Ru360 directly blocks the MCU pore, thereby abolishing Ca^2+^ influx, whereas spermine enhances MCU’s affinity for Ca^2+^, promoting Ca^2+^ uptake (Nathan et al., 2017; Tu et al., 2025). To further substantiate our findings, we also assessed MCU expression levels and intracellular Ca^2+^ concentrations in our experiments, confirming the targeted modulation of MCU and intracellular calcium. Therefore, the use of these two pharmacological agents ensures sufficient causal and logical grounding in our experimental design. Our study suggested that Ru360 reduced the transfer of cytosolic Ca^2+^ into the mitochondria, and that suppressing excessive mitochondrial fission protected mitochondrial morphology, preserved normal mitochondrial function, and attenuated apoptosis in HK-2 cells under the present study conditions. Briefly, MCU inhibition shows consistent protective effects, whereas MCU activation exhibited a tendency to exacerbate mitochondrial fragmentation and cellular damage, but this did not consistently reach statistical significance. We referenced the mitochondrial morphology study design as described by the teams of Xiao and Wang. (Wang et al., 2020; Xiao et al., 2022). Mitochondrial length was defined and assessed by calculating the perimeter, while mitochondrial roundness was analyzed using the form factor and the aspect ratio, allowing for an estimation of the extent of mitochondrial fragmentation. Collectively, these results support the conclusion that MCU-mediated mitochondrial Ca^2+^ overload may represent an upstream event in mitochondrial injury and dysfunction. However, unlike the specific inhibitory effect of Ru360, spermine did not produce the anticipated activating effect on MCU. Compared to the iohexol-only group, the Iohexol + Spermine group exhibited an increase in mitochondrial Ca^2+^ levels, but the difference was not statistically significant. Furthermore, the degree of mitochondrial membrane potential loss and the expression of DRP1 did not show a further increase compared to the iohexol group. We speculate that it may be because MCU-mediated Ca^2+^ uptake in HK-2 cells was already significantly elevated by iohexol treatment, approaching a state of saturation. Consequently, further activation of MCU with spermine could not elicit a meaningful additional increase in the intramitochondrial Ca^2+^ concentration. The impact of this MCU-mediated Ca^2+^ overload on mitochondrial dynamics appears to preferentially drive mitochondrial fission, leading to increased fragmentation, while exerting a relatively minor effect on fusion. This observed limited influence on fusion may also be linked to the over inhibition of mitochondrial fusion processes by iohexol itself. Therefore, Accordingly, neither inhibitory nor activatory interventions targeting MCU successfully restored the mitochondrial fusion process. Based on these findings, we hypothesize that rectifying the imbalance in mitochondrial dynamics may effectively ameliorate mitochondrial dysfunction and mitigate RTECs injury and apoptosis, potentially representing a novel therapeutic target for CI-AKI.

This study demonstrates the mechanism underlying MCU-mediated mitochondrial injury. Substantial evidence from recent years indicates that mitochondrial quality control plays a critical role in the pathogenesis, progression, and repair phases of acute kidney injury (Li et al., 2020). As a crucial component of mitochondrial quality control, mitochondrial dynamics may be functionally linked to MCU. Mitochondria are highly dynamic organelles that maintain a homeostatic balance through continuous fission and fusion, which is called mitochondrial dynamics (Zhan et al., 2013). Fission is a multi-step process centrally mediated by DRP1, which is itself regulated by post-translational modifications such as phosphorylation and SUMOylation (Labbé et al., 2014; Romanello and Sandri, 2015). The mitochondrial fusion process is precisely regulated by the outer membrane mitofusins (MFN-1, MFN-2) and the inner membrane protein OPA1 (Wang et al., 2023). Accumulating evidence indicates that mutations in fission/fusion proteins can trigger extensive apoptosis and autophagy, and disruption of the balance between fusion and fission alters mitochondrial morphology and impairs mitochondrial function and cell viability (Slupe et al., 2013; Huang et al., 2025). Thereby, mitochondrial fission and fusion are now recognized as cornerstones of cell survival, exerting a profound influence on both health and disease states (Marcuzzo et al., 2025; Yako et al., 2021; Rahmani et al., 2023).

Imbalanced mitochondrial dynamics has been established as a contributor to the pathophysiology of diseases such as hypertensive heart disease (HHD), diabetes, inflammatory bowel disease (IBD), and cancer. Excessive activation or upregulation of DRP1 promotes mitochondrial fragmentation and cellular injury. This fragmentation impairs mitochondrial function by reducing ATP production, increasing ROS generation, and facilitating the release of pro-apoptotic factors (Mweene et al., 2025; Kumar et al., 2022; Kowluru and Kumar, 2025; Chen et al., 2024; Guo et al., 2025). Our findings provide further evidence implicating disrupted mitochondrial dynamics in CM-induced RTEC injury. MCU upregulation exacerbates mitochondrial fission and fragmentation, ultimately leading to increased apoptosis in HK-2 cells. Furthermore, impaired mitochondrial fusion serves as another critical factor leading to mitochondrial fragmentation. Specific deletion of MFN-2 in proximal tubular epithelial cells results in excessive mitochondrial fragmentation and increased release of apoptotic factors (Li et al., 2025; Gall et al., 2015; Dang et al., 2022). These findings are consistent with our conclusion that the extent of excessive mitochondrial fission is positively correlated with the degree of mitochondrial damage and cellular apoptosis. Our study further confirms that MCU upregulation exacerbates mitochondrial fission, fragmentation, and disruption of cristae structure, ultimately leading to increased apoptosis in HK-2 cells. Furthermore, we observed a cytoprotective effect resulting from the pharmacological inhibition of MCU by Ru360, which acts through the suppression of excessive mitochondrial fission. Current clinical evidence indicates that pharmacological inhibitors of DRP1, such as Mdivi-1, reduce mitochondrial fission, ameliorate oxidative stress and inflammatory responses, and thereby promote mitochondrial biogenesis. Knockdown or targeted inhibition of DRP1 prevents pathological mitochondrial fission at its source (Zhang et al., 2023; Ye et al., 2024; Yang Y. et al., 2025; Ma et al., 2025; Zhang C. et al., 2025; Mishra et al., 2025). Collectively, these findings demonstrate that aberrant mitochondrial fission is critically implicated in a spectrum of pathologies. Strategies aimed at inhibiting excessive mitochondrial fission show significant therapeutic potential, particularly in metabolic, cardiovascular, and neurodegenerative diseases. Our study is the first to identify a mechanism that MCU inhibition confers protection against CM-induced RTEC injury by attenuating excessive mitochondrial fission, presenting a potential novel strategy for the prevention and treatment of CI-AKI.

However, this study also existed limitations: First, the investigation was confined to *in vitro* cell models; it lacked *in vivo* validation in animal models or did not involve further verification in primary mouse RTECs. Our research is focused on mechanistic exploration using the HK-2 cell line, which offers high purity and excellent reproducibility. Second, we did not experimentally confirm whether MCU contributes to mitochondrial injury specifically by disrupting mitochondrial dynamics, nor did we perform interventional studies targeting mitochondrial fission. Then, the use of three biological replicates each group, while meeting the minimum acceptable sample size in the field, may compromise statistical power, particularly given the multiple comparisons and the substantial variability inherent in mitochondrial morphology and fluorescence intensity measurements. Thus, these findings and the oversaturation hypothesis require further validation in larger cohorts. Finally, the precise mechanisms underlying the increased MCU expression remain elusive and warrant further investigation.

## 5 Conclusion

The present study revealed a novel path mechanism of CI-AKI: In the *in vitro* model of CI-AKI, the upregulation of MCU disturbed mitochondrial dynamics homeostasis (excessive mitochondrial fission and increased mitochondrial fragmentation) via accelerating Ca^2+^ influx into mitochondria, thereby causing mitochondrial dysfunction and inducing cell apoptosis. Briefly, these results provide initial evidence that MCU-mediated imbalance of mitochondrial dynamics could play a critical role in CI-AKI. Thus, inhibiting MCU may serve as a potential therapeutic strategy against CI-AKI.

## Data Availability

The raw data supporting the conclusions of this article will be made available by the authors, without undue reservation.
