# Supplementary material for: Mitochondrial calcium uniporter-mediated mitochondrial dynamics imbalance contributes to contrast medium-induced renal tubular cell injury
Source: Front Mol Biosci. 2026 Jun 29;13:1848361. doi: 10.3389/fmolb.2026.1848361 (PMC13357276; doi:10.3389/fmolb.2026.1848361)
Supplement: Supplementary file 1 [file DataSheet3.zip › Flow Cytometry Assay(1,2)/Flow Cytometry Assay-1/╧╕░√╡≥═÷-1/╡≥═÷ 1/▒¿╕μ - ╡≥═÷ 1.pdf]

凋亡 1 报告

标本名：凋亡 1　　　　检验时间：2024/7/4 11:04

仪器：NovoCyte 451160320945　　　　软件：NovoExpress 1.2.4

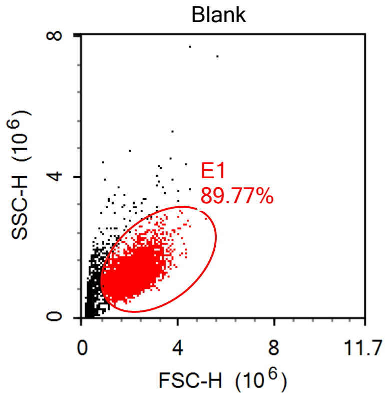

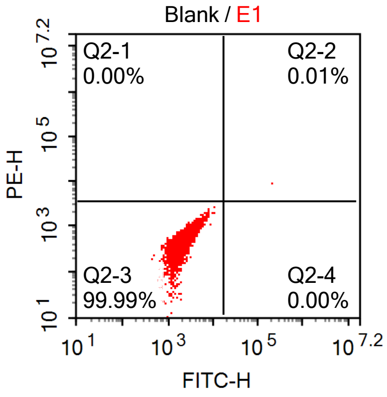

Gate	Count	% All	Mean X	M[illegible]
All	10,000	100.00%	1,899,064	1,[illegible]
E1	8,977	89.77%	2,058,449	1,[illegible]

Gate	Count	% E1	% All	Mean X	Mean Y	Median X	Median Y
E1	8,977	100.00%	89.77%	2,208	519	2,059	488
Q2-1	0	0.00%	0.00%	0	0	0	0
Q2-2	1	0.01%	0.01%	188,598	8,970	188,598	8,970
Q2-3	8,976	99.99%	89.76%	2,187	518	2,058	488
Q2-4	0	0.00%	0.00%	0	0	0	0

样本统计表格 - Blank

Gate	Count	% Parent	% All	X	Y	Mean X	Mean Y	Median X	Median Y
All	10,000								
E1	8,977	89.77%	89.77%	FSC-H	SSC-H	2,058,449	1,136,148	2,022,254	1,085,849
Q2-1	0	0.00%	0.00%	FITC-H	PE-H	0	0	0	0
Q2-2	1	0.01%	0.01%	FITC-H	PE-H	188,598	8,970	188,598	8,970
Q2-3	8,976	99.99%	89.76%	FITC-H	PE-H	2,187	518	2,058	488
Q2-4	0	0.00%	0.00%	FITC-H	PE-H	0	0	0	0

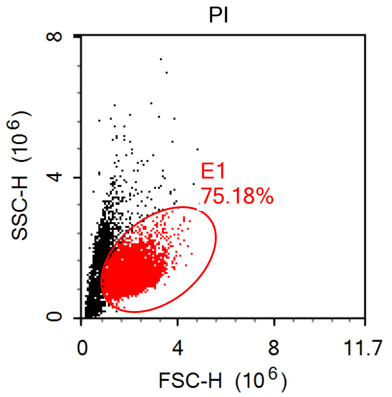

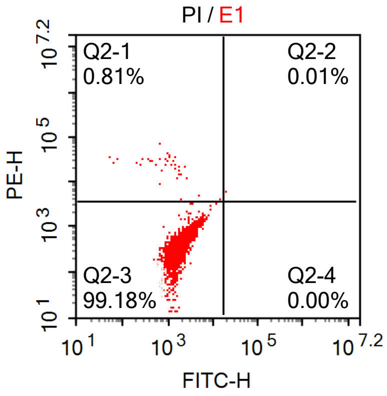

Gate	Count	% All	Mean X	M[illegible]
All	10,000	100.00%	1,622,617	1,[illegible]
E1	7,518	75.18%	1,962,360	1,[illegible]

Gate	Count	% E1	% All	Mean X	Mean Y	Median X	Median Y
E1	7,518	100.00%	75.18%	1,966	715	1,851	453
Q2-1	61	0.81%	0.61%	1,137	29,152	619	29,690
Q2-2	1	0.01%	0.01%	19,207	6,172	19,207	6,172
Q2-3	7,456	99.18%	74.56%	1,971	482	1,854	452
Q2-4	0	0.00%	0.00%	0	0	0	0

样本统计表格 - PI

Gate	Count	% Parent	% All	X	Y	Mean X	Mean Y	Median X	Median Y
All	10,000								
E1	7,518	75.18%	75.18%	FSC-H	SSC-H	1,962,360	1,205,916	1,924,004	1,155,358
Q2-1	61	0.81%	0.61%	FITC-H	PE-H	1,137	29,152	619	29,690
Q2-2	1	0.01%	0.01%	FITC-H	PE-H	19,207	6,172	19,207	6,172
Q2-3	7,456	99.18%	74.56%	FITC-H	PE-H	1,971	482	1,854	452
Q2-4	0	0.00%	0.00%	FITC-H	PE-H	0	0	0	0

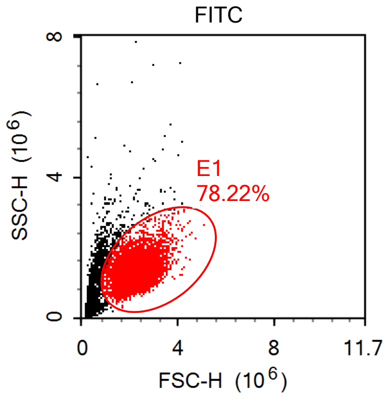

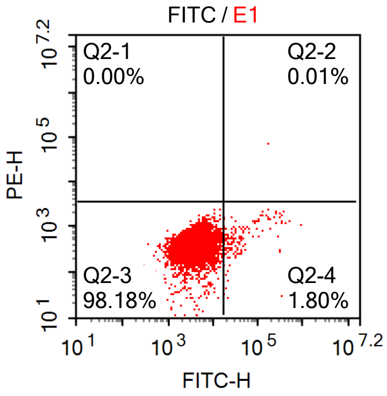

Gate	Count	% All	Mean X	M[illegible]
All	10,000	100.00%	1,844,370	1,[illegible]
E1	7,822	78.22%	2,218,423	1,[illegible]

Gate	Count	% E1	% All	Mean X	Mean Y	Median X	Median Y
E1	7,822	100.00%	78.22%	5,573	486	3,428	442
Q2-1	0	0.00%	0.00%	0	0	0	0
Q2-2	1	0.01%	0.01%	158,762	75,317	158,762	75,317
Q2-3	7,680	98.18%	76.80%	4,121	470	3,388	440
Q2-4	141	1.80%	1.41%	83,575	791	33,419	603

样本统计表格 - FITC

Gate	Count	% Parent	% All	X	Y	Mean X	Mean Y	Median X	Median Y
All	10,000								
E1	7,822	78.22%	78.22%	FSC-H	SSC-H	2,218,423	1,249,316	2,197,299	1,193,096
Q2-1	0	0.00%	0.00%	FITC-H	PE-H	0	0	0	0
Q2-2	1	0.01%	0.01%	FITC-H	PE-H	158,762	75,317	158,762	75,317
Q2-3	7,680	98.18%	76.80%	FITC-H	PE-H	4,121	470	3,388	440
Q2-4	141	1.80%	1.41%	FITC-H	PE-H	83,575	791	33,419	603

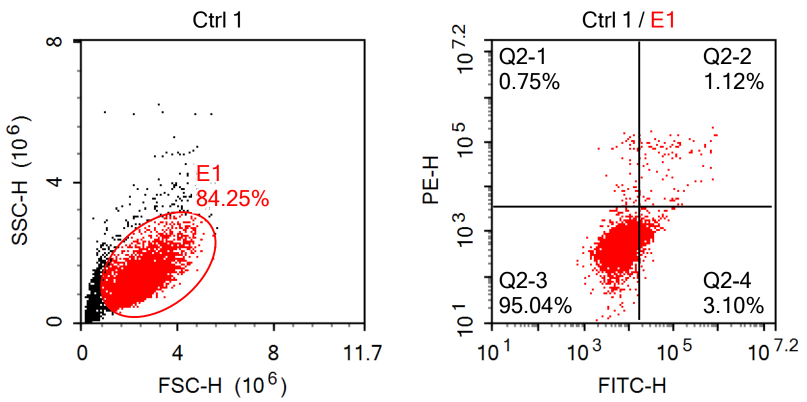

Gate	Count	% All	Mean X	M[illegible]
All	10,000	100.00%	2,014,427	1,[illegible]
E1	8,425	84.25%	2,266,676	1,[illegible]

Gate	Count	% E1	% All	Mean X	Mean Y	Median X	Median Y
E1	8,425	100.00%	84.25%	9,335	1,576	6,530	547
Q2-1	63	0.75%	0.63%	10,695	47,238	10,180	20,943
Q2-2	94	1.12%	0.94%	160,685	56,217	90,579	55,023
Q2-3	8,007	95.04%	80.07%	6,989	590	6,361	535
Q2-4	261	3.10%	2.61%	26,450	1,111	22,541	988

样本统计表格 - Ctrl 1

Gate	Count	% Parent	% All	X	Y	Mean X	Mean Y	Median X	Median Y
All	10,000								
E1	8,425	84.25%	84.25%	FSC-H	SSC-H	2,266,676	1,117,872	2,199,039	1,016,148
Q2-1	63	0.75%	0.63%	FITC-H	PE-H	10,695	47,238	10,180	20,943
Q2-2	94	1.12%	0.94%	FITC-H	PE-H	160,685	56,217	90,579	55,023
Q2-3	8,007	95.04%	80.07%	FITC-H	PE-H	6,989	590	6,361	535
Q2-4	261	3.10%	2.61%	FITC-H	PE-H	26,450	1,111	22,541	988

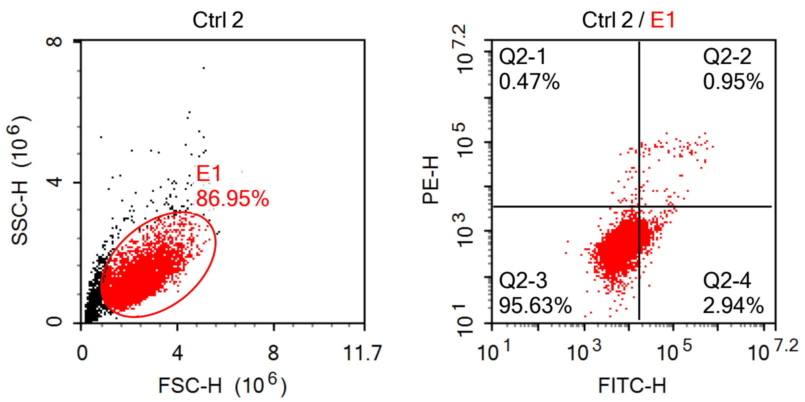

Gate	Count	% All	Mean X	M(	Gate	Count	% E1	% All	Mean X	Mean Y	Median X	Median Y
All	10,000	100.00%	2,028,216	1,(	E1	8,695	100.00%	86.95%	8,926	1,226	6,483	539
E1	8,695	86.95%	2,230,791	1,(	Q2-1	41	0.47%	0.41%	10,705	21,923	11,412	9,133
					Q2-2	83	0.95%	0.83%	150,051	56,144	96,376	61,459
					Q2-3	8,315	95.63%	83.15%	6,924	580	6,313	527
					Q2-4	256	2.94%	2.56%	27,924	1,086	22,513	916

样本统计表格 - Ctrl 2

Gate	Count	% Parent	% All	X	Y	Mean X	Mean Y	Median X	Median Y
All	10,000								
E1	8,695	86.95%	86.95%	FSC-H	SSC-H	2,230,791	1,078,756	2,173,001	986,162
Q2-1	41	0.47%	0.41%	FITC-H	PE-H	10,705	21,923	11,412	9,133
Q2-2	83	0.95%	0.83%	FITC-H	PE-H	150,051	56,144	96,376	61,459
Q2-3	8,315	95.63%	83.15%	FITC-H	PE-H	6,924	580	6,313	527
Q2-4	256	2.94%	2.56%	FITC-H	PE-H	27,924	1,086	22,513	916

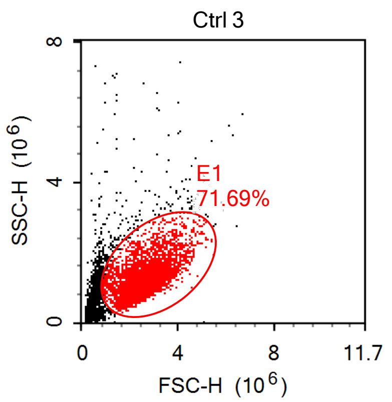

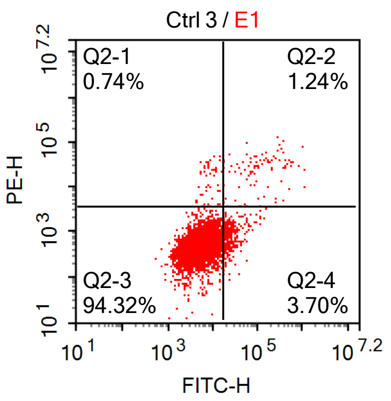

Gate	Count	% All	Mean X	M
All	10,000	100.00%	1,866,350	1,(
E1	7,169	71.69%	2,397,830	1,'

Gate	Count	% E1	% All	Mean X	Mean Y	Median X	Median Y
E1	7,169	100.00%	71.69%	8,892	1,126	4,926	542
Q2-1	53	0.74%	0.53%	9,076	19,792	7,898	19,334
Q2-2	89	1.24%	0.89%	196,178	31,512	105,437	28,804
Q2-3	6,762	94.32%	67.62%	5,625	586	4,730	528
Q2-4	265	3.70%	2.65%	29,302	988	22,974	873

样本统计表格 - Ctrl 3

Gate	Count	% Parent	% All	X	Y	Mean X	Mean Y	Median X	Median Y
All	10,000								
└ E1	7,169	71.69%	71.69%	FSC-H	SSC-H	2,397,830	1,102,823	2,323,327	999,389
├ Q2-1	53	0.74%	0.53%	FITC-H	PE-H	9,076	19,792	7,898	19,334
├ Q2-2	89	1.24%	0.89%	FITC-H	PE-H	196,178	31,512	105,437	28,804
├ Q2-3	6,762	94.32%	67.62%	FITC-H	PE-H	5,625	586	4,730	528
└ Q2-4	265	3.70%	2.65%	FITC-H	PE-H	29,302	988	22,974	873

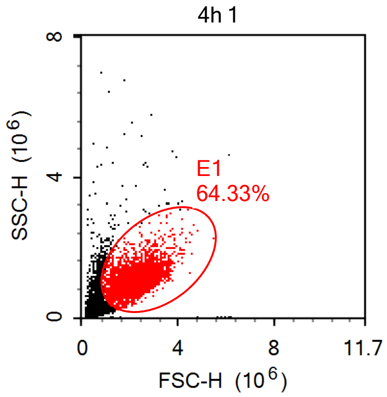

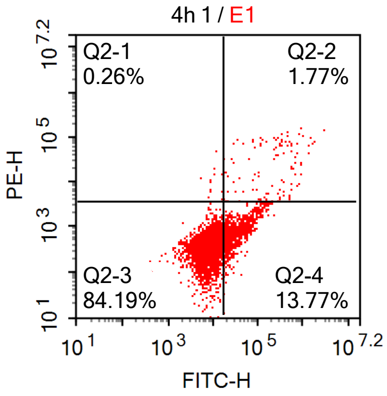

Gate	Count	% All	Mean X	M(	Gate	Count	% E1	% All	Mean X	Mean Y	Median X	Median Y
All	10,000	100.00%	1,557,672	81	E1	6,433	100.00%	64.33%	18,569	1,192	8,531	409
E1	6,433	64.33%	2,195,061	1,(	Q2-1	17	0.26%	0.17%	11,524	13,263	10,163	10,413
					Q2-2	114	1.77%	1.14%	360,160	38,855	241,561	18,340
					Q2-3	5,416	84.19%	54.16%	8,358	405	7,717	377
					Q2-4	886	13.77%	8.86%	37,167	923	27,339	777

样本统计表格 - 4h 1

Gate	Count	% Parent	% All	X	Y	Mean X	Mean Y	Median X	Median Y
All	10,000								
E1	6,433	64.33%	64.33%	FSC-H	SSC-H	2,195,061	1,031,441	2,170,522	994,141
Q2-1	17	0.26%	0.17%	FITC-H	PE-H	11,524	13,263	10,163	10,413
Q2-2	114	1.77%	1.14%	FITC-H	PE-H	360,160	38,855	241,561	18,340
Q2-3	5,416	84.19%	54.16%	FITC-H	PE-H	8,358	405	7,717	377
Q2-4	886	13.77%	8.86%	FITC-H	PE-H	37,167	923	27,339	777

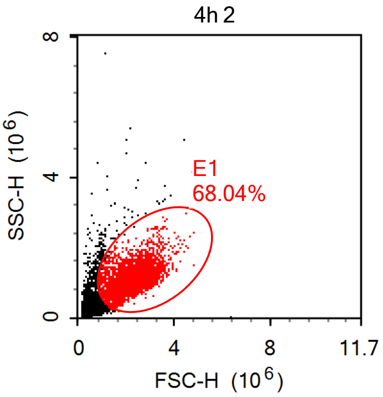

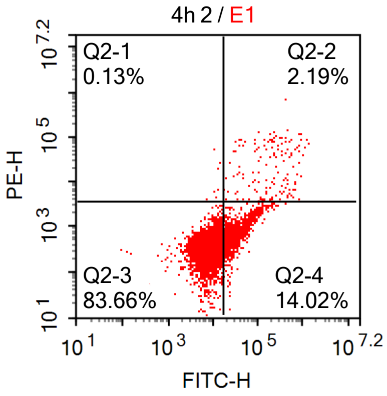

Gate	Count	% All	Mean X	M(
All	10,000	100.00%	1,610,469	82
E1	6,804	68.04%	2,194,650	1,(

Gate	Count	% E1	% All	Mean X	Mean Y	Median X	Median Y
E1	6,804	100.00%	68.04%	18,715	1,422	8,639	410
Q2-1	9	0.13%	0.09%	11,422	15,260	11,458	6,523
Q2-2	149	2.19%	1.49%	289,191	42,285	202,277	22,180
Q2-3	5,692	83.66%	56.92%	8,349	409	7,747	376
Q2-4	954	14.02%	9.54%	38,394	951	27,563	777

样本统计表格 - 4h 2

Gate	Count	% Parent	% All	X	Y	Mean X	Mean Y	Median X	Median Y
All	10,000								
E1	6,804	68.04%	68.04%	FSC-H	SSC-H	2,194,650	1,041,152	2,177,377	1,005,437
Q2-1	9	0.13%	0.09%	FITC-H	PE-H	11,422	15,260	11,458	6,523
Q2-2	149	2.19%	1.49%	FITC-H	PE-H	289,191	42,285	202,277	22,180
Q2-3	5,692	83.66%	56.92%	FITC-H	PE-H	8,349	409	7,747	376
Q2-4	954	14.02%	9.54%	FITC-H	PE-H	38,394	951	27,563	777

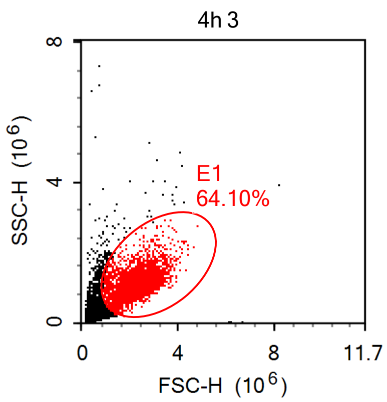

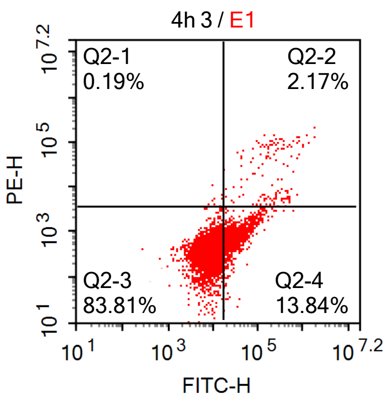

Gate	Count	% All	Mean X	M(
All	10,000	100.00%	1,533,101	78
E1	6,410	64.10%	2,180,423	1,(

Gate	Count	% E1	% All	Mean X	Mean Y	Median X	Median Y
E1	6,410	100.00%	64.10%	18,938	1,398	8,774	413
Q2-1	12	0.19%	0.12%	11,600	6,894	12,018	4,576
Q2-2	139	2.17%	1.39%	291,974	41,957	203,086	18,772
Q2-3	5,372	83.81%	53.72%	8,491	410	7,884	378
Q2-4	887	13.84%	8.87%	39,523	953	28,186	792

样本统计表格 - 4h 3

Gate	Count	% Parent	% All	X	Y	Mean X	Mean Y	Median X	Median Y
All	10,000								
E1	6,410	64.10%	64.10%	FSC-H	SSC-H	2,180,423	1,026,844	2,154,077	984,024
Q2-1	12	0.19%	0.12%	FITC-H	PE-H	11,600	6,894	12,018	4,576
Q2-2	139	2.17%	1.39%	FITC-H	PE-H	291,974	41,957	203,086	18,772
Q2-3	5,372	83.81%	53.72%	FITC-H	PE-H	8,491	410	7,884	378
Q2-4	887	13.84%	8.87%	FITC-H	PE-H	39,523	953	28,186	792

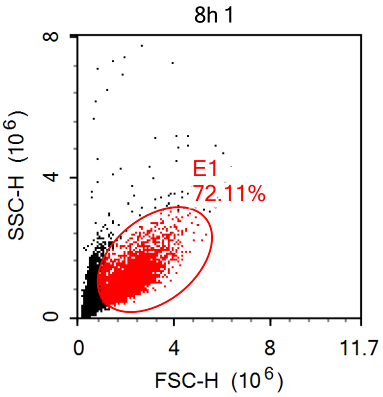

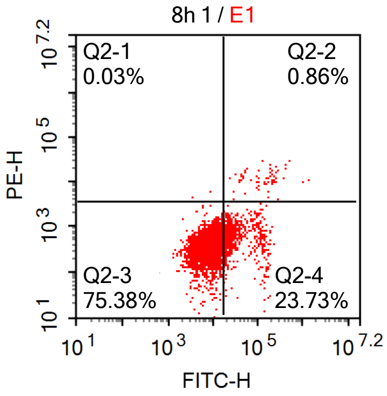

Gate	Count	% All	Mean X	M
All	10,000	100.00%	1,507,721	86
E1	7,211	72.11%	1,911,262	97

Gate	Count	% E1	% All	Mean X	Mean Y	Median X	Median Y
E1	7,211	100.00%	72.11%	42,266	252	9,885	414
Q2-1	2	0.03%	0.02%	6,012	5,496	6,012	5,496
Q2-2	62	0.86%	0.62%	176,900	13,198	140,057	12,203
Q2-3	5,436	75.38%	54.36%	8,678	462	8,184	435
Q2-4	1,711	23.73%	17.11%	144,141	-891	132,118	-334

样本统计表格 - 8h 1

Gate	Count	% Parent	% All	X	Y	Mean X	Mean Y	Median X	Median Y
All	10,000								
E1	7,211	72.11%	72.11%	FSC-H	SSC-H	1,911,262	975,023	1,829,278	893,744
Q2-1	2	0.03%	0.02%	FITC-H	PE-H	6,012	5,496	6,012	5,496
Q2-2	62	0.86%	0.62%	FITC-H	PE-H	176,900	13,198	140,057	12,203
Q2-3	5,436	75.38%	54.36%	FITC-H	PE-H	8,678	462	8,184	435
Q2-4	1,711	23.73%	17.11%	FITC-H	PE-H	144,141	-891	132,118	-334

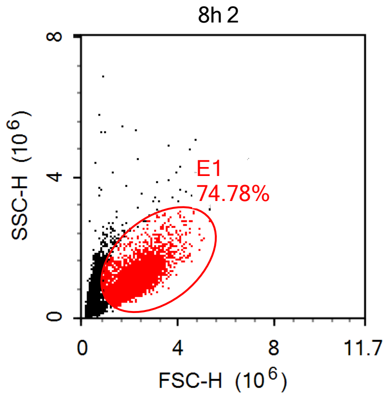

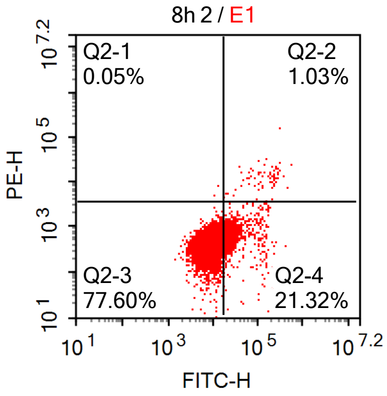

Gate	Count	% All	Mean X	M(
All	10,000	100.00%	1,618,126	88
E1	7,478	74.78%	2,022,122	99

Gate	Count	% E1	% All	Mean X	Mean Y	Median X	Median Y
E1	7,478	100.00%	74.78%	33,478	426	10,533	444
Q2-1	4	0.05%	0.04%	14,077	6,005	14,190	5,319
Q2-2	77	1.03%	0.77%	167,103	15,253	177,149	12,947
Q2-3	5,803	77.60%	58.03%	9,347	478	8,920	446
Q2-4	1,594	21.32%	15.94%	114,918	-494	58,569	361

样本统计表格 - 8h 2

Gate	Count	% Parent	% All	X	Y	Mean X	Mean Y	Median X	Median Y
All	10,000								
E1	7,478	74.78%	74.78%	FSC-H	SSC-H	2,022,122	992,111	1,941,984	911,820
Q2-1	4	0.05%	0.04%	FITC-H	PE-H	14,077	6,005	14,190	5,319
Q2-2	77	1.03%	0.77%	FITC-H	PE-H	167,103	15,253	177,149	12,947
Q2-3	5,803	77.60%	58.03%	FITC-H	PE-H	9,347	478	8,920	446
Q2-4	1,594	21.32%	15.94%	FITC-H	PE-H	114,918	-494	58,569	361

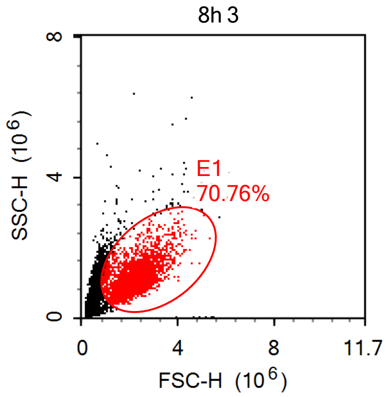

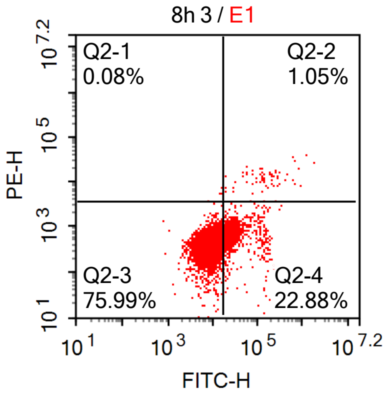

Gate	Count	% All	Mean X	M	Gate	Count	% E1	% All	Mean X	Mean Y	Median X	Median Y
All	10,000	100.00%	1,562,039	88	E1	7,076	100.00%	70.76%	37,089	370	10,484	434
E1	7,076	70.76%	2,012,054	97	Q2-1	6	0.08%	0.06%	11,642	5,004	11,995	4,779
					Q2-2	74	1.05%	0.74%	223,899	13,232	130,948	12,990
					Q2-3	5,377	75.99%	53.77%	9,234	473	8,739	442
					Q2-4	1,619	22.88%	16.19%	121,159	-577	97,881	224

样本统计表格 - 8h 3

Gate	Count	% Parent	% All	X	Y	Mean X	Mean Y	Median X	Median Y
All	10,000								
E1	7,076	70.76%	70.76%	FSC-H	SSC-H	2,012,054	979,664	1,949,530	909,373
Q2-1	6	0.08%	0.06%	FITC-H	PE-H	11,642	5,004	11,995	4,779
Q2-2	74	1.05%	0.74%	FITC-H	PE-H	223,899	13,232	130,948	12,990
Q2-3	5,377	75.99%	53.77%	FITC-H	PE-H	9,234	473	8,739	442
Q2-4	1,619	22.88%	16.19%	FITC-H	PE-H	121,159	-577	97,881	224

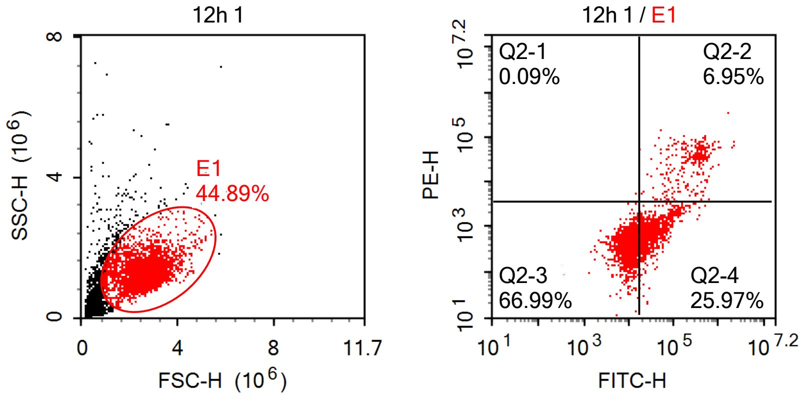

Gate	Count	% All	Mean X	M
All	10,000	100.00%	1,350,614	72
E1	4,489	44.89%	2,613,480	1,:

Gate	Count	% E1	% All	Mean X	Mean Y	Median X	Median Y
E1	4,489	100.00%	44.89%	38,824	3,294	13,433	551
Q2-1	4	0.09%	0.04%	13,300	16,359	13,431	4,433
Q2-2	312	6.95%	3.12%	312,567	38,919	299,651	36,357
Q2-3	3,007	66.99%	30.07%	10,944	499	10,777	471
Q2-4	1,166	25.97%	11.66%	37,564	924	25,033	781

样本统计表格 - 12h 1

Gate	Count	% Parent	% All	X	Y	Mean X	Mean Y	Median X	Median Y
All	10,000								
E1	4,489	44.89%	44.89%	FSC-H	SSC-H	2,613,480	1,241,023	2,644,095	1,177,818
Q2-1	4	0.09%	0.04%	FITC-H	PE-H	13,300	16,359	13,431	4,433
Q2-2	312	6.95%	3.12%	FITC-H	PE-H	312,567	38,919	299,651	36,357
Q2-3	3,007	66.99%	30.07%	FITC-H	PE-H	10,944	499	10,777	471
Q2-4	1,166	25.97%	11.66%	FITC-H	PE-H	37,564	924	25,033	781

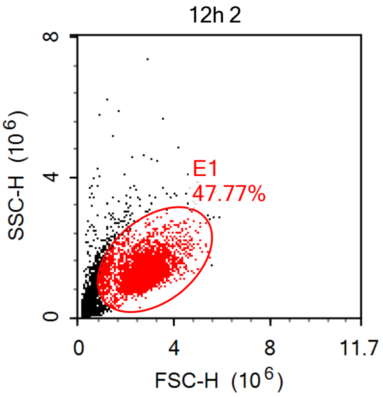

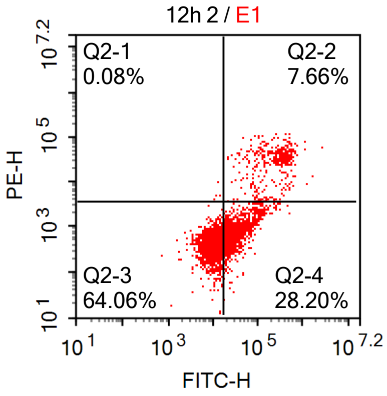

Gate	Count	% All	Mean X	M
All	10,000	100.00%	1,443,112	76
E1	4,777	47.77%	2,662,036	1,:

Gate	Count	% E1	% All	Mean X	Mean Y	Median X	Median Y
E1	4,777	100.00%	47.77%	38,810	3,236	14,002	571
Q2-1	4	0.08%	0.04%	11,549	6,103	11,780	5,137
Q2-2	366	7.66%	3.66%	276,675	34,481	251,289	33,564
Q2-3	3,060	64.06%	30.60%	11,039	504	10,946	484
Q2-4	1,347	28.20%	13.47%	37,348	942	24,817	788

样本统计表格 - 12h 2

Gate	Count	% Parent	% All	X	Y	Mean X	Mean Y	Median X	Median Y
All	10,000								
E1	4,777	47.77%	47.77%	FSC-H	SSC-H	2,662,036	1,262,321	2,695,323	1,197,803
Q2-1	4	0.08%	0.04%	FITC-H	PE-H	11,549	6,103	11,780	5,137
Q2-2	366	7.66%	3.66%	FITC-H	PE-H	276,675	34,481	251,289	33,564
Q2-3	3,060	64.06%	30.60%	FITC-H	PE-H	11,039	504	10,946	484
Q2-4	1,347	28.20%	13.47%	FITC-H	PE-H	37,348	942	24,817	788

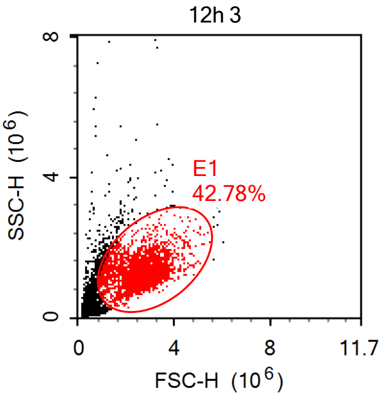

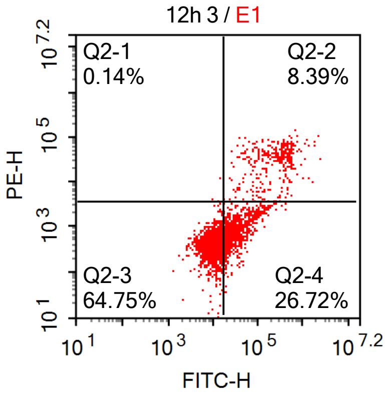

Gate	Count	% All	Mean X	M
All	10,000	100.00%	1,303,543	70
E1	4,278	42.78%	2,617,946	1,:

Gate	Count	% E1	% All	Mean X	Mean Y	Median X	Median Y
E1	4,278	100.00%	42.78%	42,996	3,723	14,044	566
Q2-1	6	0.14%	0.06%	13,465	5,201	13,134	3,960
Q2-2	359	8.39%	3.59%	310,444	37,396	287,126	35,932
Q2-3	2,770	64.75%	27.70%	11,211	505	11,287	477
Q2-4	1,143	26.72%	11.43%	36,177	937	24,402	765

样本统计表格 - 12h 3

Gate	Count	% Parent	% All	X	Y	Mean X	Mean Y	Median X	Median Y
All	10,000								
E1	4,278	42.78%	42.78%	FSC-H	SSC-H	2,617,946	1,244,918	2,680,125	1,180,688
Q2-1	6	0.14%	0.06%	FITC-H	PE-H	13,465	5,201	13,134	3,960
Q2-2	359	8.39%	3.59%	FITC-H	PE-H	310,444	37,396	287,126	35,932
Q2-3	2,770	64.75%	27.70%	FITC-H	PE-H	11,211	505	11,287	477
Q2-4	1,143	26.72%	11.43%	FITC-H	PE-H	36,177	937	24,402	765
