# Supplementary material for: Mitochondrial calcium uniporter-mediated mitochondrial dynamics imbalance contributes to contrast medium-induced renal tubular cell injury
Source: Front Mol Biosci. 2026 Jun 29;13:1848361. doi: 10.3389/fmolb.2026.1848361 (PMC13357276; doi:10.3389/fmolb.2026.1848361)
Supplement: Supplementary file 1 [file DataSheet3.zip › Flow Cytometry Assay(1,2)/Flow Cytometry Assay-1/╧╕░√╡≥═÷-1/╡≥═÷ 2/▒¿╕μ - ╡≥═÷ 2.pdf]

凋亡 2 报告

标本名：凋亡 2　　　　　　　　　　　　　　检验时间：2024/7/4 11:11

仪器：NovoCyte 451160320945　　　　　　　软件：NovoExpress 1.2.4

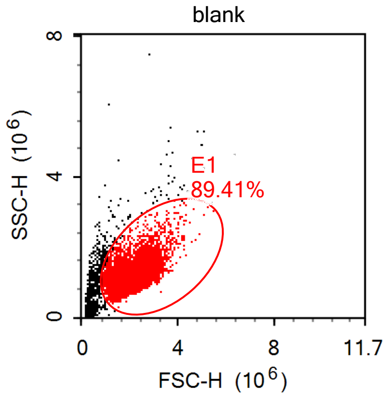

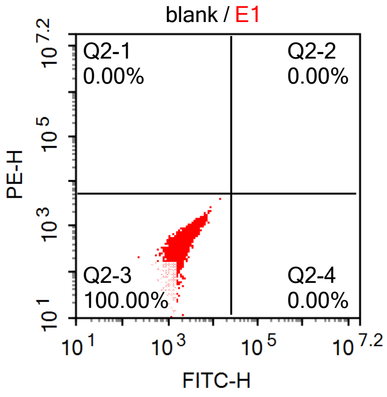

Gate	Count	% All	Mean X	M
All	10,000	100.00%	1,951,842	1,
E1	8,941	89.41%	2,119,785	1,

Gate	Count	% E1	% All	Mean X	Mean Y	Median X	Median Y
E1	8,941	100.00%	89.41%	2,165	502	2,022	470
Q2-1	0	0.00%	0.00%	0	0	0	0
Q2-2	0	0.00%	0.00%	0	0	0	0
Q2-3	8,941	100.00%	89.41%	2,165	502	2,022	470
Q2-4	0	0.00%	0.00%	0	0	0	0

样本统计表格 - blank

Gate	Count	% Parent	% All	X	Y	Mean X	Mean Y	Median X	Median Y
All	10,000								
E1	8,941	89.41%	89.41%	FSC-H	SSC-H	2,119,785	1,203,887	2,084,747	1,151,915
Q2-1	0	0.00%	0.00%	FITC-H	PE-H	0	0	0	0
Q2-2	0	0.00%	0.00%	FITC-H	PE-H	0	0	0	0
Q2-3	8,941	100.00%	89.41%	FITC-H	PE-H	2,165	502	2,022	470
Q2-4	0	0.00%	0.00%	FITC-H	PE-H	0	0	0	0

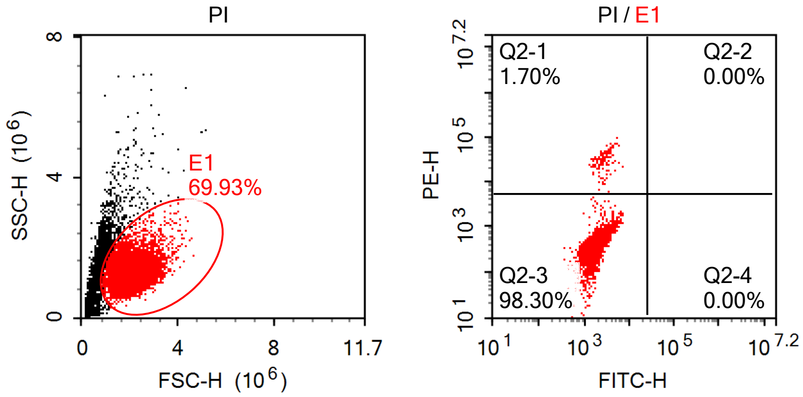

Gate	Count	% All	Mean X	M[illegible]
All	10,000	100.00%	1,652,113	1,[illegible]
E1	6,993	69.93%	2,110,110	1,[illegible]

Gate	Count	% E1	% All	Mean X	Mean Y	Median X	Median Y
E1	6,993	100.00%	69.93%	1,974	1,017	1,859	440
Q2-1	119	1.70%	1.19%	2,773	32,501	2,583	31,056
Q2-2	0	0.00%	0.00%	0	0	0	0
Q2-3	6,874	98.30%	68.74%	1,961	472	1,851	436
Q2-4	0	0.00%	0.00%	0	0	0	0

样本统计表格 - PI

Gate	Count	% Parent	% All	X	Y	Mean X	Mean Y	Median X	Median Y
All	10,000								
E1	6,993	69.93%	69.93%	FSC-H	SSC-H	2,110,110	1,259,372	2,097,687	1,199,750
Q2-1	119	1.70%	1.19%	FITC-H	PE-H	2,773	32,501	2,583	31,056
Q2-2	0	0.00%	0.00%	FITC-H	PE-H	0	0	0	0
Q2-3	6,874	98.30%	68.74%	FITC-H	PE-H	1,961	472	1,851	436
Q2-4	0	0.00%	0.00%	FITC-H	PE-H	0	0	0	0

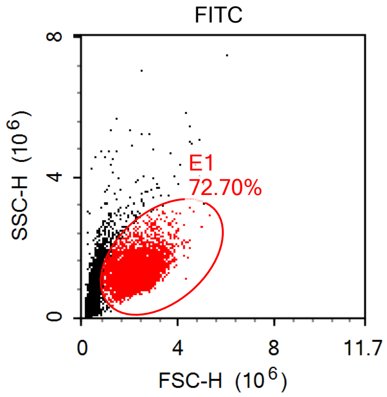

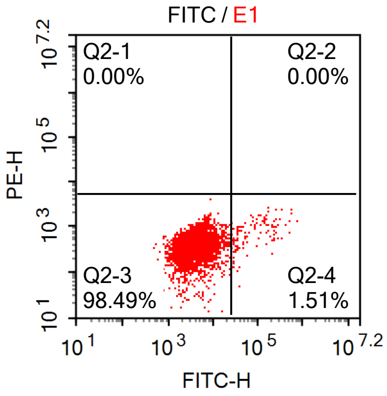

Gate	Count	% All	Mean X	M[illegible]
All	10,000	100.00%	1,723,438	1,[illegible]
E1	7,270	72.70%	2,206,422	1,[illegible]

Gate	Count	% E1	% All	Mean X	Mean Y	Median X	Median Y
E1	7,270	100.00%	72.70%	6,038	471	3,429	441
Q2-1	0	0.00%	0.00%	0	0	0	0
Q2-2	0	0.00%	0.00%	0	0	0	0
Q2-3	7,160	98.49%	71.60%	4,122	464	3,384	439
Q2-4	110	1.51%	1.10%	130,704	949	89,617	859

样本统计表格 - FITC

Gate	Count	% Parent	% All	X	Y	Mean X	Mean Y	Median X	Median Y
All	10,000								
E1	7,270	72.70%	72.70%	FSC-H	SSC-H	2,206,422	1,233,904	2,192,501	1,171,797
Q2-1	0	0.00%	0.00%	FITC-H	PE-H	0	0	0	0
Q2-2	0	0.00%	0.00%	FITC-H	PE-H	0	0	0	0
Q2-3	7,160	98.49%	71.60%	FITC-H	PE-H	4,122	464	3,384	439
Q2-4	110	1.51%	1.10%	FITC-H	PE-H	130,704	949	89,617	859

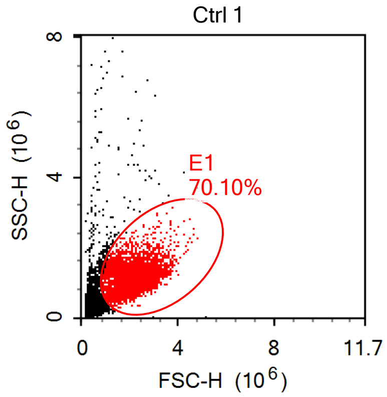

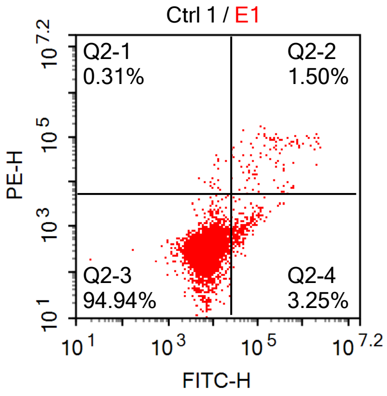

Gate	Count	% All	Mean X	M
All	10,000	100.00%	1,638,265	91
E1	7,010	70.10%	2,185,091	1,(

Gate	Count	% E1	% All	Mean X	Mean Y	Median X	Median Y
E1	7,010	100.00%	70.10%	15,269	1,299	7,122	364
Q2-1	22	0.31%	0.22%	13,114	18,261	11,828	11,920
Q2-2	105	1.50%	1.05%	409,050	55,835	221,889	54,741
Q2-3	6,655	94.94%	66.55%	7,802	387	6,919	352
Q2-4	228	3.25%	2.28%	52,078	1,151	40,399	938

样本统计表格 - Ctrl 1

Gate	Count	% Parent	% All	X	Y	Mean X	Mean Y	Median X	Median Y
All	10,000								
E1	7,010	70.10%	70.10%	FSC-H	SSC-H	2,185,091	1,080,641	2,180,647	1,033,955
Q2-1	22	0.31%	0.22%	FITC-H	PE-H	13,114	18,261	11,828	11,920
Q2-2	105	1.50%	1.05%	FITC-H	PE-H	409,050	55,835	221,889	54,741
Q2-3	6,655	94.94%	66.55%	FITC-H	PE-H	7,802	387	6,919	352
Q2-4	228	3.25%	2.28%	FITC-H	PE-H	52,078	1,151	40,399	938

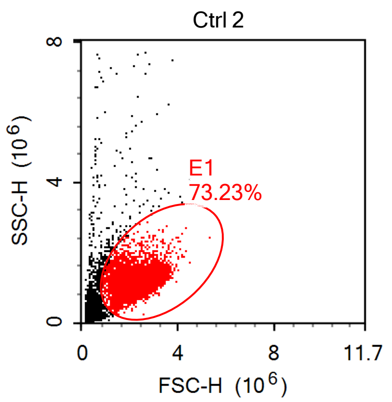

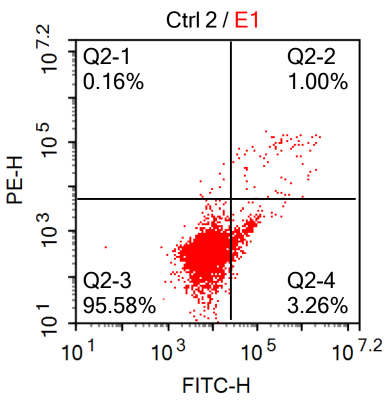

Gate	Count	% All	Mean X	M
All	10,000	100.00%	1,721,512	99
E1	7,323	73.23%	2,202,526	1,(

Gate	Count	% E1	% All	Mean X	Mean Y	Median X	Median Y
E1	7,323	100.00%	73.23%	14,331	1,077	6,865	367
Q2-1	12	0.16%	0.12%	10,446	11,646	10,235	8,563
Q2-2	73	1.00%	0.73%	516,812	64,839	313,548	64,148
Q2-3	6,999	95.58%	69.99%	7,666	392	6,709	357
Q2-4	239	3.26%	2.39%	56,241	1,149	43,756	927

样本统计表格 - Ctrl 2

Gate	Count	% Parent	% All	X	Y	Mean X	Mean Y	Median X	Median Y
All	10,000								
E1	7,323	73.23%	73.23%	FSC-H	SSC-H	2,202,526	1,096,847	2,204,781	1,056,830
Q2-1	12	0.16%	0.12%	FITC-H	PE-H	10,446	11,646	10,235	8,563
Q2-2	73	1.00%	0.73%	FITC-H	PE-H	516,812	64,839	313,548	64,148
Q2-3	6,999	95.58%	69.99%	FITC-H	PE-H	7,666	392	6,709	357
Q2-4	239	3.26%	2.39%	FITC-H	PE-H	56,241	1,149	43,756	927

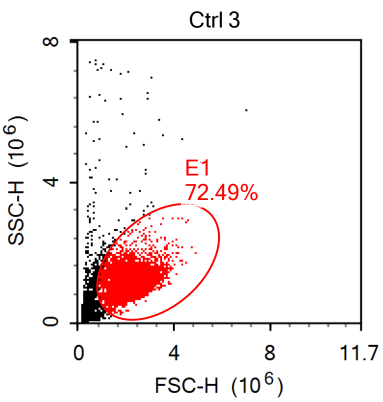

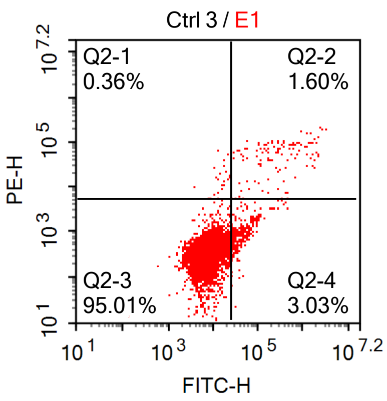

Gate	Count	% All	Mean X	M(
All	10,000	100.00%	1,700,529	95
E1	7,249	72.49%	2,202,993	1,(

Gate	Count	% E1	% All	Mean X	Mean Y	Median X	Median Y
E1	7,249	100.00%	72.49%	18,213	1,484	7,165	365
Q2-1	26	0.36%	0.26%	15,418	17,717	15,355	14,694
Q2-2	116	1.60%	1.16%	558,443	63,256	275,182	60,004
Q2-3	6,887	95.01%	68.87%	7,961	389	6,979	356
Q2-4	220	3.03%	2.20%	54,599	1,258	39,417	977

样本统计表格 - Ctrl 3

Gate	Count	% Parent	% All	X	Y	Mean X	Mean Y	Median X	Median Y
All	10,000								
E1	7,249	72.49%	72.49%	FSC-H	SSC-H	2,202,993	1,096,903	2,201,722	1,051,110
Q2-1	26	0.36%	0.26%	FITC-H	PE-H	15,418	17,717	15,355	14,694
Q2-2	116	1.60%	1.16%	FITC-H	PE-H	558,443	63,256	275,182	60,004
Q2-3	6,887	95.01%	68.87%	FITC-H	PE-H	7,961	389	6,979	356
Q2-4	220	3.03%	2.20%	FITC-H	PE-H	54,599	1,258	39,417	977

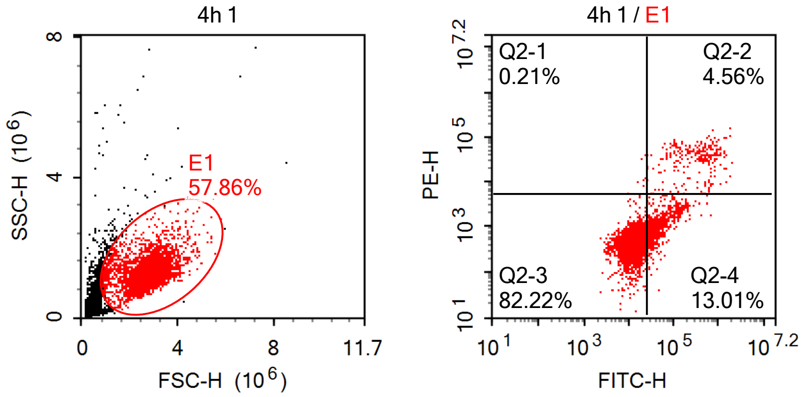

Gate	Count	% All	Mean X	M
All	10,000	100.00%	1,678,395	85
E1	5,786	57.86%	2,664,801	1,

Gate	Count	% E1	% All	Mean X	Mean Y	Median X	Median Y
E1	5,786	100.00%	57.86%	38,240	2,452	13,996	530
Q2-1	12	0.21%	0.12%	20,071	18,297	21,229	12,113
Q2-2	264	4.56%	2.64%	438,345	40,145	376,510	38,827
Q2-3	4,757	82.22%	47.57%	13,186	507	12,484	478
Q2-4	753	13.01%	7.53%	56,525	1,270	37,703	952

样本统计表格 - 4h 1

Gate	Count	% Parent	% All	X	Y	Mean X	Mean Y	Median X	Median Y
All	10,000								
E1	5,786	57.86%	57.86%	FSC-H	SSC-H	2,664,801	1,196,822	2,684,838	1,131,582
Q2-1	12	0.21%	0.12%	FITC-H	PE-H	20,071	18,297	21,229	12,113
Q2-2	264	4.56%	2.64%	FITC-H	PE-H	438,345	40,145	376,510	38,827
Q2-3	4,757	82.22%	47.57%	FITC-H	PE-H	13,186	507	12,484	478
Q2-4	753	13.01%	7.53%	FITC-H	PE-H	56,525	1,270	37,703	952

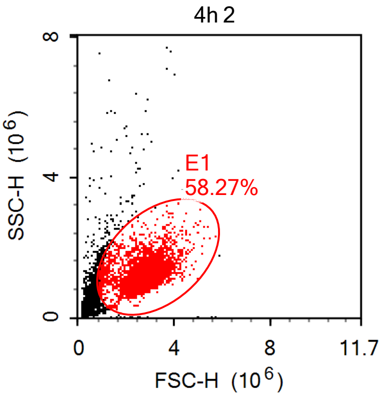

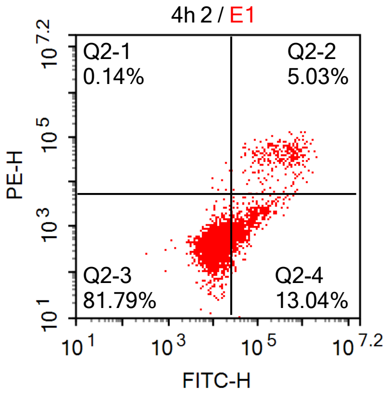

Gate	Count	% All	Mean X	M
All	10,000	100.00%	1,687,611	86
E1	5,827	58.27%	2,653,719	1,'

Gate	Count	% E1	% All	Mean X	Mean Y	Median X	Median Y
E1	5,827	100.00%	58.27%	40,149	2,718	14,081	526
Q2-1	8	0.14%	0.08%	16,232	13,490	16,055	11,819
Q2-2	293	5.03%	2.93%	435,341	42,226	374,313	39,256
Q2-3	4,766	81.79%	47.66%	13,132	507	12,423	474
Q2-4	760	13.04%	7.60%	57,466	1,241	37,602	955

样本统计表格 - 4h 2

Gate	Count	% Parent	% All	X	Y	Mean X	Mean Y	Median X	Median Y
All	10,000								
E1	5,827	58.27%	58.27%	FSC-H	SSC-H	2,653,719	1,188,012	2,675,382	1,118,071
Q2-1	8	0.14%	0.08%	FITC-H	PE-H	16,232	13,490	16,055	11,819
Q2-2	293	5.03%	2.93%	FITC-H	PE-H	435,341	42,226	374,313	39,256
Q2-3	4,766	81.79%	47.66%	FITC-H	PE-H	13,132	507	12,423	474
Q2-4	760	13.04%	7.60%	FITC-H	PE-H	57,466	1,241	37,602	955

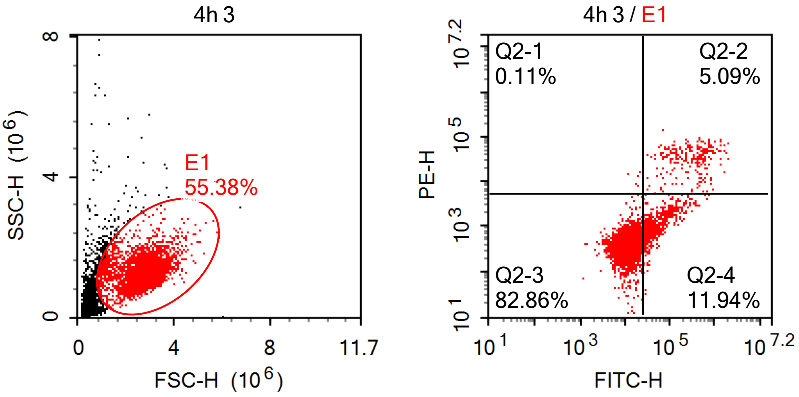

Gate	Count	% All	Mean X	M
All	10,000	100.00%	1,598,030	81
E1	5,538	55.38%	2,627,088	1,'

Gate	Count	% E1	% All	Mean X	Mean Y	Median X	Median Y
E1	5,538	100.00%	55.38%	39,107	2,614	13,573	518
Q2-1	6	0.11%	0.06%	17,124	10,983	18,270	10,900
Q2-2	282	5.09%	2.82%	414,298	39,875	380,256	38,104
Q2-3	4,589	82.86%	45.89%	13,014	506	12,239	470
Q2-4	661	11.94%	6.61%	60,387	1,279	37,719	967

样本统计表格 - 4h 3

Gate	Count	% Parent	% All	X	Y	Mean X	Mean Y	Median X	Median Y
All	10,000								
E1	5,538	55.38%	55.38%	FSC-H	SSC-H	2,627,088	1,176,689	2,656,419	1,116,975
Q2-1	6	0.11%	0.06%	FITC-H	PE-H	17,124	10,983	18,270	10,900
Q2-2	282	5.09%	2.82%	FITC-H	PE-H	414,298	39,875	380,256	38,104
Q2-3	4,589	82.86%	45.89%	FITC-H	PE-H	13,014	506	12,239	470
Q2-4	661	11.94%	6.61%	FITC-H	PE-H	60,387	1,279	37,719	967

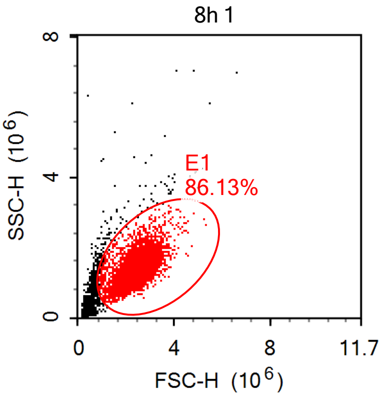

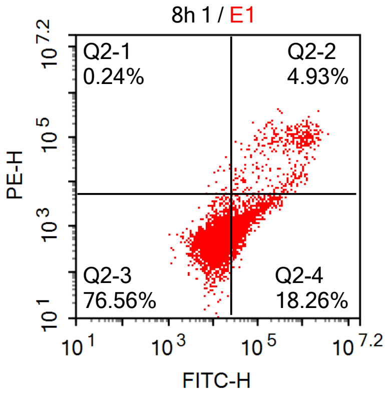

Gate	Count	% All	Mean X	M[illegible]
All	10,000	100.00%	1,983,479	1,[illegible]
E1	8,613	86.13%	2,228,853	1,[illegible]

Gate	Count	% E1	% All	Mean X	Mean Y	Median X	Median Y
E1	8,613	100.00%	86.13%	52,540	4,424	15,388	614
Q2-1	21	0.24%	0.21%	16,889	11,772	16,488	7,005
Q2-2	425	4.93%	4.25%	666,950	74,909	546,498	71,631
Q2-3	6,594	76.56%	65.94%	13,575	597	12,898	535
Q2-4	1,573	18.26%	15.73%	50,353	1,329	36,731	1,062

样本统计表格 - 8h 1

Gate	Count	% Parent	% All	X	Y	Mean X	Mean Y	Median X	Median Y
All	10,000								
E1	8,613	86.13%	86.13%	FSC-H	SSC-H	2,228,853	1,254,267	2,193,890	1,193,485
Q2-1	21	0.24%	0.21%	FITC-H	PE-H	16,889	11,772	16,488	7,005
Q2-2	425	4.93%	4.25%	FITC-H	PE-H	666,950	74,909	546,498	71,631
Q2-3	6,594	76.56%	65.94%	FITC-H	PE-H	13,575	597	12,898	535
Q2-4	1,573	18.26%	15.73%	FITC-H	PE-H	50,353	1,329	36,731	1,062

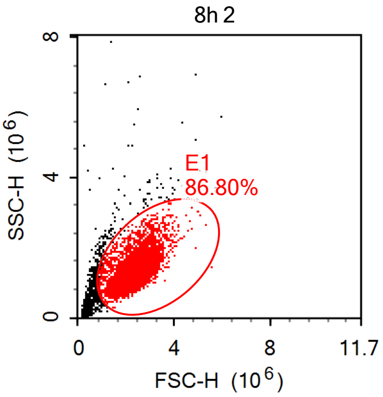

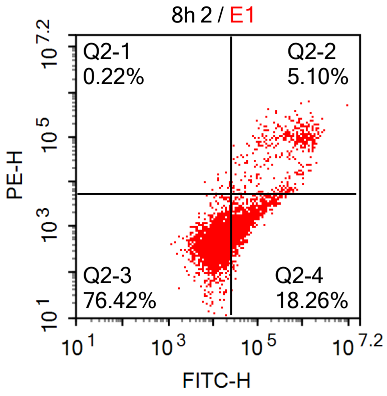

Gate	Count	% All	Mean X	M
All	10,000	100.00%	1,990,100	1,
E1	8,680	86.80%	2,215,576	1,

Gate	Count	% E1	% All	Mean X	Mean Y	Median X	Median Y
E1	8,680	100.00%	86.80%	54,292	5,202	15,484	619
Q2-1	19	0.22%	0.19%	16,060	8,915	14,523	8,036
Q2-2	443	5.10%	4.43%	684,544	87,997	497,615	78,114
Q2-3	6,633	76.42%	66.33%	13,700	593	13,041	536
Q2-4	1,585	18.26%	15.85%	48,469	1,305	36,014	1,023

样本统计表格 - 8h 2

Gate	Count	% Parent	% All	X	Y	Mean X	Mean Y	Median X	Median Y
All	10,000								
E1	8,680	86.80%	86.80%	FSC-H	SSC-H	2,215,576	1,271,216	2,187,612	1,202,924
Q2-1	19	0.22%	0.19%	FITC-H	PE-H	16,060	8,915	14,523	8,036
Q2-2	443	5.10%	4.43%	FITC-H	PE-H	684,544	87,997	497,615	78,114
Q2-3	6,633	76.42%	66.33%	FITC-H	PE-H	13,700	593	13,041	536
Q2-4	1,585	18.26%	15.85%	FITC-H	PE-H	48,469	1,305	36,014	1,023

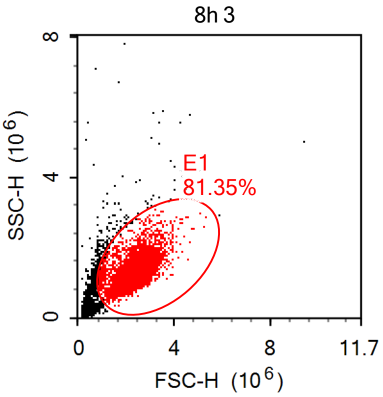

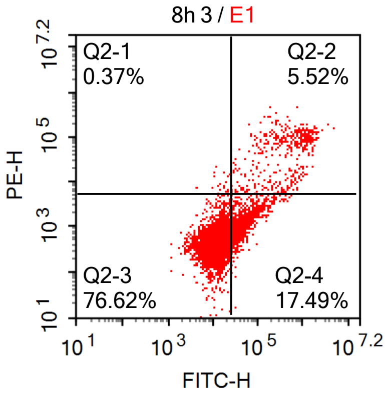

Gate	Count	% All	Mean X	M
All	10,000	100.00%	1,875,692	1,
E1	8,135	81.35%	2,207,899	1,

Gate	Count	% E1	% All	Mean X	Mean Y	Median X	Median Y
E1	8,135	100.00%	81.35%	51,899	4,798	15,131	604
Q2-1	30	0.37%	0.30%	17,059	9,125	17,430	7,442
Q2-2	449	5.52%	4.49%	596,681	74,012	440,138	70,413
Q2-3	6,233	76.62%	62.33%	13,412	582	12,717	526
Q2-4	1,423	17.49%	14.23%	49,320	1,334	36,612	1,069

样本统计表格 - 8h 3

Gate	Count	% Parent	% All	X	Y	Mean X	Mean Y	Median X	Median Y
All	10,000								
E1	8,135	81.35%	81.35%	FSC-H	SSC-H	2,207,899	1,228,915	2,180,975	1,169,297
Q2-1	30	0.37%	0.30%	FITC-H	PE-H	17,059	9,125	17,430	7,442
Q2-2	449	5.52%	4.49%	FITC-H	PE-H	596,681	74,012	440,138	70,413
Q2-3	6,233	76.62%	62.33%	FITC-H	PE-H	13,412	582	12,717	526
Q2-4	1,423	17.49%	14.23%	FITC-H	PE-H	49,320	1,334	36,612	1,069

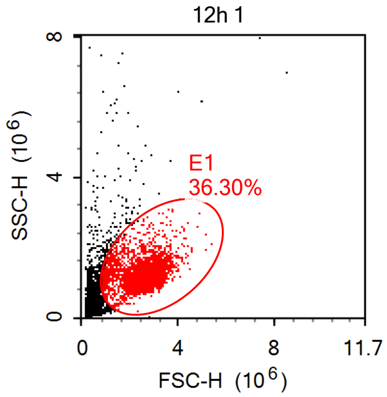

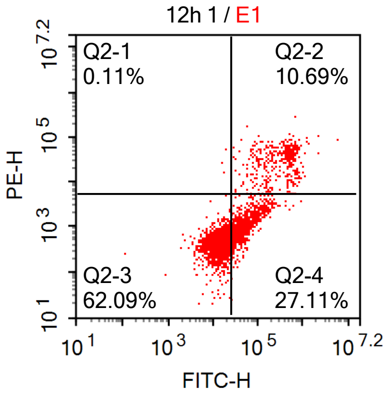

Gate	Count	% All	Mean X	M
All	10,000	100.00%	1,088,887	65
E1	3,630	36.30%	2,481,664	1,:

Gate	Count	% E1	% All	Mean X	Mean Y	Median X	Median Y
E1	3,630	100.00%	36.30%	67,388	4,805	20,335	640
Q2-1	4	0.11%	0.04%	17,298	25,424	17,564	21,301
Q2-2	388	10.69%	3.88%	389,817	38,268	389,693	35,715
Q2-3	2,254	62.09%	22.54%	15,286	536	14,987	509
Q2-4	984	27.11%	9.84%	59,802	1,303	41,871	1,025

样本统计表格 - 12h 1

Gate	Count	% Parent	% All	X	Y	Mean X	Mean Y	Median X	Median Y
All	10,000								
E1	3,630	36.30%	36.30%	FSC-H	SSC-H	2,481,664	1,207,287	2,565,684	1,146,905
Q2-1	4	0.11%	0.04%	FITC-H	PE-H	17,298	25,424	17,564	21,301
Q2-2	388	10.69%	3.88%	FITC-H	PE-H	389,817	38,268	389,693	35,715
Q2-3	2,254	62.09%	22.54%	FITC-H	PE-H	15,286	536	14,987	509
Q2-4	984	27.11%	9.84%	FITC-H	PE-H	59,802	1,303	41,871	1,025

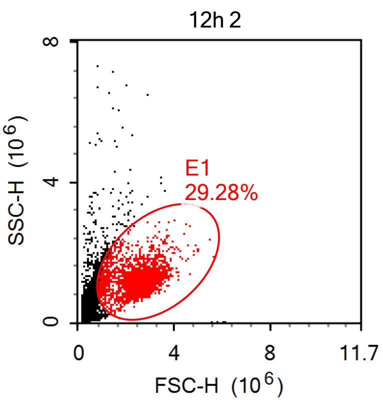

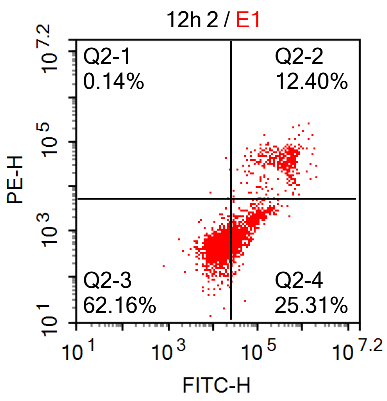

Gate	Count	% All	Mean X	M
All	10,000	100.00%	921,116	57
E1	2,928	29.28%	2,441,710	1,

Gate	Count	% E1	% All	Mean X	Mean Y	Median X	Median Y
E1	2,928	100.00%	29.28%	74,059	6,014	20,427	652
Q2-1	4	0.14%	0.04%	17,149	20,676	19,335	15,763
Q2-2	363	12.40%	3.63%	401,309	42,849	392,304	38,010
Q2-3	1,820	62.16%	18.20%	15,270	549	14,919	526
Q2-4	741	25.31%	7.41%	58,449	1,313	39,463	983

样本统计表格 - 12h 2

Gate	Count	% Parent	% All	X	Y	Mean X	Mean Y	Median X	Median Y
All	10,000								
E1	2,928	29.28%	29.28%	FSC-H	SSC-H	2,441,710	1,185,823	2,529,884	1,125,808
Q2-1	4	0.14%	0.04%	FITC-H	PE-H	17,149	20,676	19,335	15,763
Q2-2	363	12.40%	3.63%	FITC-H	PE-H	401,309	42,849	392,304	38,010
Q2-3	1,820	62.16%	18.20%	FITC-H	PE-H	15,270	549	14,919	526
Q2-4	741	25.31%	7.41%	FITC-H	PE-H	58,449	1,313	39,463	983

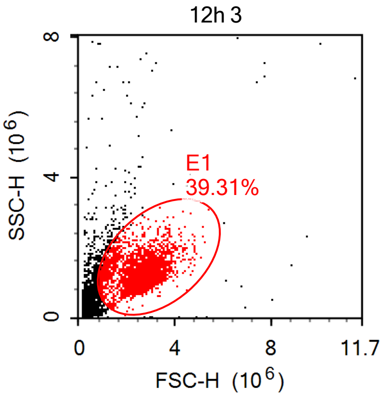

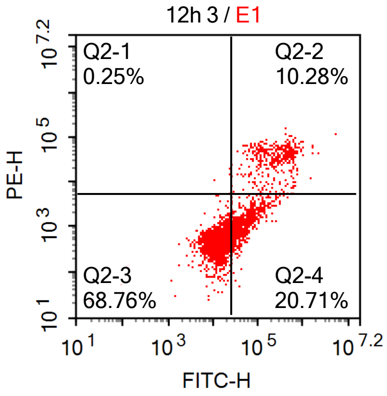

Gate	Count	% All	Mean X	M	Gate	Count	% E1	% All	Mean X	Mean Y	Median X	Median Y
All	10,000	100.00%	1,202,422	70	E1	3,931	100.00%	39.31%	56,580	4,815	17,778	639
E1	3,931	39.31%	2,554,061	1,:	Q2-1	10	0.25%	0.10%	20,101	11,499	22,847	9,644
					Q2-2	404	10.28%	4.04%	341,952	39,975	285,558	38,677
					Q2-3	2,703	68.76%	27.03%	14,474	573	14,011	533
					Q2-4	814	20.71%	8.14%	55,211	1,369	39,330	1,073

样本统计表格 - 12h 3

Gate	Count	% Parent	% All	X	Y	Mean X	Mean Y	Median X	Median Y
All	10,000								
E1	3,931	39.31%	39.31%	FSC-H	SSC-H	2,554,061	1,229,057	2,628,739	1,164,392
Q2-1	10	0.25%	0.10%	FITC-H	PE-H	20,101	11,499	22,847	9,644
Q2-2	404	10.28%	4.04%	FITC-H	PE-H	341,952	39,975	285,558	38,677
Q2-3	2,703	68.76%	27.03%	FITC-H	PE-H	14,474	573	14,011	533
Q2-4	814	20.71%	8.14%	FITC-H	PE-H	55,211	1,369	39,330	1,073
