# Supplementary material for: Mitochondrial calcium uniporter-mediated mitochondrial dynamics imbalance contributes to contrast medium-induced renal tubular cell injury
Source: Front Mol Biosci. 2026 Jun 29;13:1848361. doi: 10.3389/fmolb.2026.1848361 (PMC13357276; doi:10.3389/fmolb.2026.1848361)
Supplement: Supplementary file 1 [file DataSheet3.zip › Flow Cytometry Assay(1,2)/Flow Cytometry Assay-1/╧╕░√╡≥═÷-1/╡≥═÷ 3/▒¿╕μ - ╡≥═÷ 3.pdf]

凋亡 3 报告

标本名：凋亡 3　　　　检验时间：2024/9/14 18:15

仪器：NovoCyte 451160320945　　　　软件：NovoExpress 1.2.4

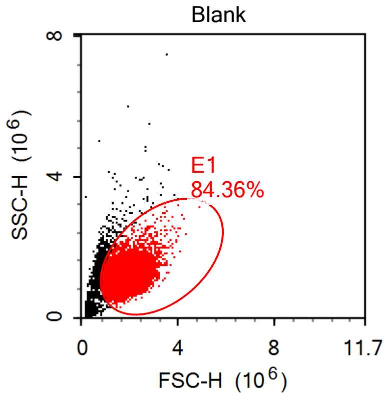

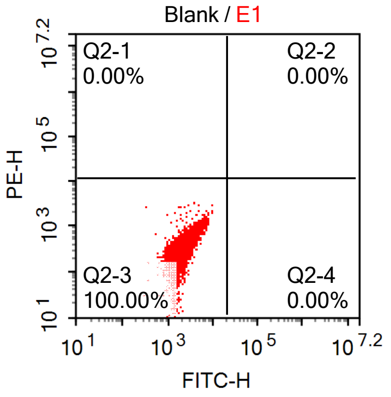

Gate	Count	% All	Mean X	M
All	10,000	100.00%	1,642,036	1,(
E1	8,436	84.36%	1,845,869	1,(

Gate	Count	% E1	% All	Mean X	Mean Y	Median X	Median Y
E1	8,436	100.00%	84.36%	2,263	410	2,126	380
Q2-1	0	0.00%	0.00%	0	0	0	0
Q2-2	0	0.00%	0.00%	0	0	0	0
Q2-3	8,436	100.00%	84.36%	2,263	410	2,126	380
Q2-4	0	0.00%	0.00%	0	0	0	0

样本统计表格 - Blank

Gate	Count	% Parent	% All	X	Y	Mean X	Mean Y	Median X	Median Y
All	10,000								
E1	8,436	84.36%	84.36%	FSC-H	SSC-H	1,845,869	1,089,576	1,812,281	1,020,501
Q2-1	0	0.00%	0.00%	FITC-H	PE-H	0	0	0	0
Q2-2	0	0.00%	0.00%	FITC-H	PE-H	0	0	0	0
Q2-3	8,436	100.00%	84.36%	FITC-H	PE-H	2,263	410	2,126	380
Q2-4	0	0.00%	0.00%	FITC-H	PE-H	0	0	0	0

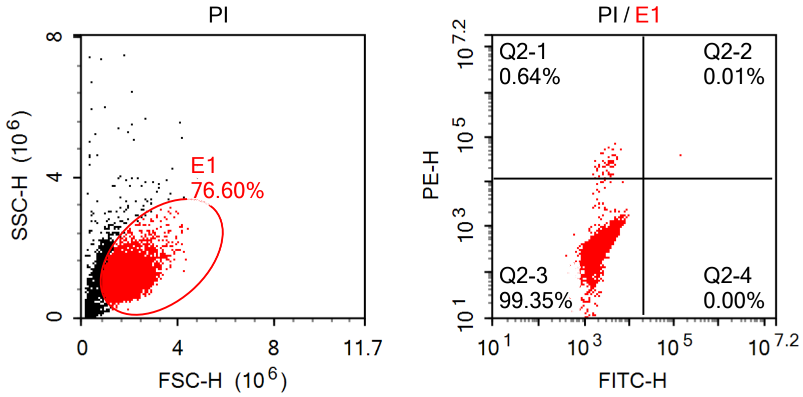

Gate	Count	% All	Mean X	M(
All	10,000	100.00%	1,536,161	1,(
E1	7,660	76.60%	1,863,115	1,(

Gate	Count	% E1	% All	Mean X	Mean Y	Median X	Median Y
E1	7,660	100.00%	76.60%	2,211	622	2,042	390
Q2-1	49	0.64%	0.49%	3,320	28,761	3,405	25,369
Q2-2	1	0.01%	0.01%	134,278	38,742	134,278	38,742
Q2-3	7,610	99.35%	76.10%	2,186	435	2,037	389
Q2-4	0	0.00%	0.00%	0	0	0	0

样本统计表格 - PI

Gate	Count	% Parent	% All	X	Y	Mean X	Mean Y	Median X	Median Y
All	10,000								
E1	7,660	76.60%	76.60%	FSC-H	SSC-H	1,863,115	1,086,936	1,829,792	1,018,317
Q2-1	49	0.64%	0.49%	FITC-H	PE-H	3,320	28,761	3,405	25,369
Q2-2	1	0.01%	0.01%	FITC-H	PE-H	134,278	38,742	134,278	38,742
Q2-3	7,610	99.35%	76.10%	FITC-H	PE-H	2,186	435	2,037	389
Q2-4	0	0.00%	0.00%	FITC-H	PE-H	0	0	0	0

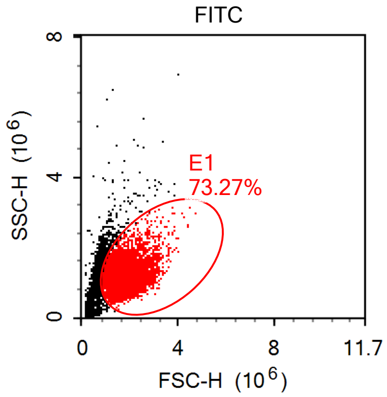

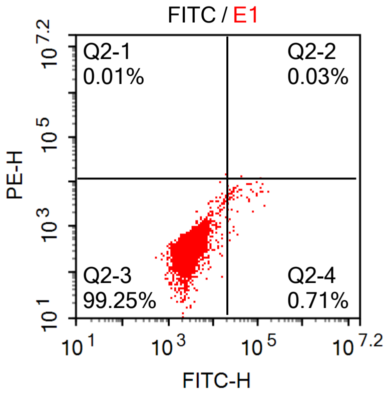

Gate	Count	% All	Mean X	M(
All	10,000	100.00%	1,473,025	99
E1	7,327	73.27%	1,847,022	1,(

Gate	Count	% E1	% All	Mean X	Mean Y	Median X	Median Y
E1	7,327	100.00%	73.27%	3,554	454	2,908	367
Q2-1	1	0.01%	0.01%	18,243	15,261	18,243	15,261
Q2-2	2	0.03%	0.02%	75,263	12,400	75,263	12,400
Q2-3	7,272	99.25%	72.72%	3,199	413	2,897	365
Q2-4	52	0.71%	0.52%	50,245	5,454	32,734	5,841

样本统计表格 - FITC

Gate	Count	% Parent	% All	X	Y	Mean X	Mean Y	Median X	Median Y
All	10,000								
E1	7,327	73.27%	73.27%	FSC-H	SSC-H	1,847,022	1,088,738	1,816,412	1,016,956
Q2-1	1	0.01%	0.01%	FITC-H	PE-H	18,243	15,261	18,243	15,261
Q2-2	2	0.03%	0.02%	FITC-H	PE-H	75,263	12,400	75,263	12,400
Q2-3	7,272	99.25%	72.72%	FITC-H	PE-H	3,199	413	2,897	365
Q2-4	52	0.71%	0.52%	FITC-H	PE-H	50,245	5,454	32,734	5,841

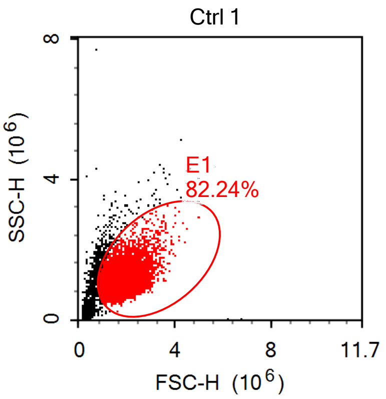

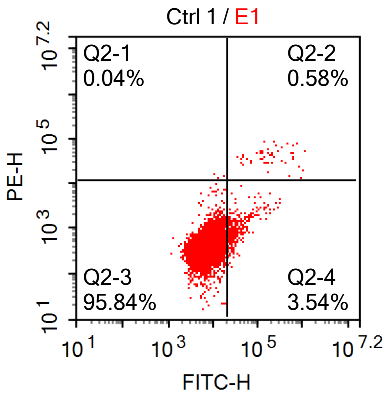

Gate	Count	% All	Mean X	M
All	10,000	100.00%	1,621,077	1,
E1	8,224	82.24%	1,858,183	1,

Gate	Count	% E1	% All	Mean X	Mean Y	Median X	Median Y
E1	8,224	100.00%	82.24%	10,867	807	7,392	473
Q2-1	3	0.04%	0.03%	13,291	15,159	12,747	14,630
Q2-2	48	0.58%	0.48%	278,368	44,277	165,220	41,326
Q2-3	7,882	95.84%	78.82%	8,028	518	7,225	463
Q2-4	291	3.54%	2.91%	43,630	1,340	29,906	948

样本统计表格 - Ctrl 1

Gate	Count	% Parent	% All	X	Y	Mean X	Mean Y	Median X	Median Y
All	10,000								
E1	8,224	82.24%	82.24%	FSC-H	SSC-H	1,858,183	1,126,125	1,819,040	1,055,517
Q2-1	3	0.04%	0.03%	FITC-H	PE-H	13,291	15,159	12,747	14,630
Q2-2	48	0.58%	0.48%	FITC-H	PE-H	278,368	44,277	165,220	41,326
Q2-3	7,882	95.84%	78.82%	FITC-H	PE-H	8,028	518	7,225	463
Q2-4	291	3.54%	2.91%	FITC-H	PE-H	43,630	1,340	29,906	948

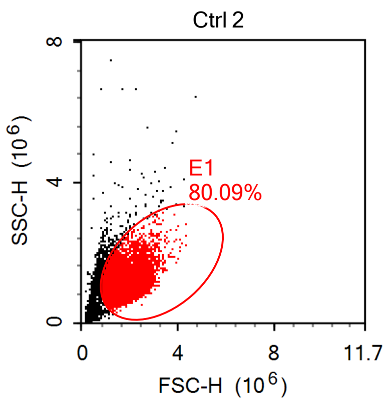

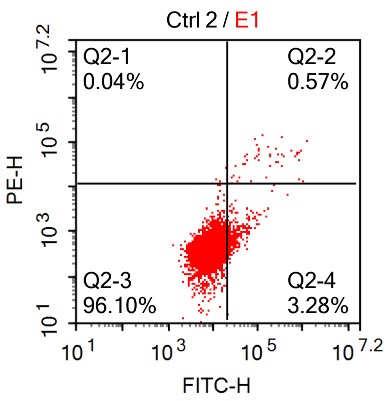

Gate	Count	% All	Mean X	M[illegible]
All	10,000	100.00%	1,548,137	1,[illegible]
E1	8,009	80.09%	1,808,746	1,[illegible]

Gate	Count	% E1	% All	Mean X	Mean Y	Median X	Median Y
E1	8,009	100.00%	80.09%	10,933	823	7,780	469
Q2-1	3	0.04%	0.03%	14,376	18,388	13,678	14,713
Q2-2	46	0.57%	0.46%	275,056	50,451	147,639	48,497
Q2-3	7,697	96.10%	76.97%	8,409	509	7,618	461
Q2-4	263	3.28%	2.63%	38,568	1,145	27,396	809

样本统计表格 - Ctrl 2

Gate	Count	% Parent	% All	X	Y	Mean X	Mean Y	Median X	Median Y
All	10,000								
E1	8,009	80.09%	80.09%	FSC-H	SSC-H	1,808,746	1,153,060	1,773,341	1,092,981
Q2-1	3	0.04%	0.03%	FITC-H	PE-H	14,376	18,388	13,678	14,713
Q2-2	46	0.57%	0.46%	FITC-H	PE-H	275,056	50,451	147,639	48,497
Q2-3	7,697	96.10%	76.97%	FITC-H	PE-H	8,409	509	7,618	461
Q2-4	263	3.28%	2.63%	FITC-H	PE-H	38,568	1,145	27,396	809

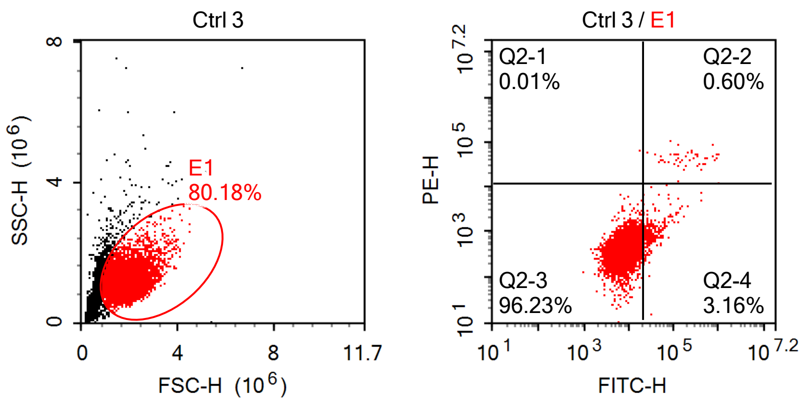

Gate	Count	% All	Mean X	M	Gate	Count	% E1	% All	Mean X	Mean Y	Median X	Median Y
All	10,000	100.00%	1,595,100	1,	E1	8,018	100.00%	80.18%	10,772	822	7,464	475
E1	8,018	80.18%	1,865,040	1,	Q2-1	1	0.01%	0.01%	16,223	64,319	16,223	64,319
					Q2-2	48	0.60%	0.48%	292,210	46,162	179,472	44,362
					Q2-3	7,716	96.23%	77.16%	8,059	515	7,300	466
					Q2-4	253	3.16%	2.53%	40,119	1,312	28,072	932

样本统计表格 - Ctrl 3

Gate	Count	% Parent	% All	X	Y	Mean X	Mean Y	Median X	Median Y
All	10,000								
E1	8,018	80.18%	80.18%	FSC-H	SSC-H	1,865,040	1,124,273	1,833,671	1,058,075
Q2-1	1	0.01%	0.01%	FITC-H	PE-H	16,223	64,319	16,223	64,319
Q2-2	48	0.60%	0.48%	FITC-H	PE-H	292,210	46,162	179,472	44,362
Q2-3	7,716	96.23%	77.16%	FITC-H	PE-H	8,059	515	7,300	466
Q2-4	253	3.16%	2.53%	FITC-H	PE-H	40,119	1,312	28,072	932

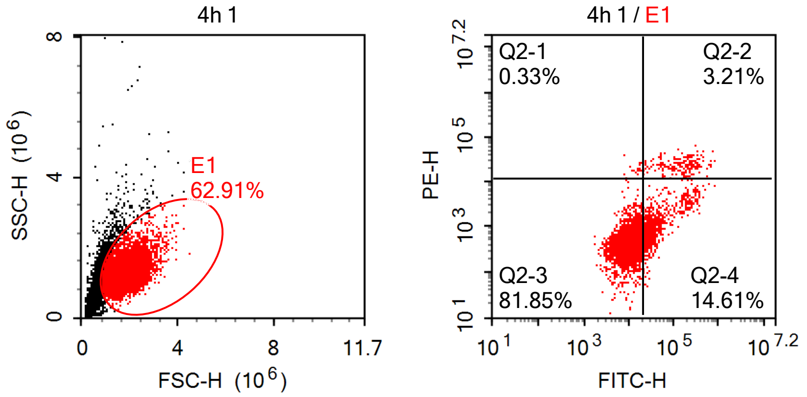

Gate	Count	% All	Mean X	M(
All	10,000	100.00%	1,276,710	1,(
E1	6,291	62.91%	1,756,134	1,:

Gate	Count	% E1	% All	Mean X	Mean Y	Median X	Median Y
E1	6,291	100.00%	62.91%	23,353	1,526	11,612	508
Q2-1	21	0.33%	0.21%	14,099	20,184	15,272	18,658
Q2-2	202	3.21%	2.02%	192,006	25,166	174,481	23,206
Q2-3	5,149	81.85%	51.49%	10,848	532	10,191	461
Q2-4	919	14.61%	9.19%	56,556	1,469	29,748	909

样本统计表格 - 4h 1

Gate	Count	% Parent	% All	X	Y	Mean X	Mean Y	Median X	Median Y
All	10,000								
E1	6,291	62.91%	62.91%	FSC-H	SSC-H	1,756,134	1,290,165	1,718,028	1,243,036
Q2-1	21	0.33%	0.21%	FITC-H	PE-H	14,099	20,184	15,272	18,658
Q2-2	202	3.21%	2.02%	FITC-H	PE-H	192,006	25,166	174,481	23,206
Q2-3	5,149	81.85%	51.49%	FITC-H	PE-H	10,848	532	10,191	461
Q2-4	919	14.61%	9.19%	FITC-H	PE-H	56,556	1,469	29,748	909

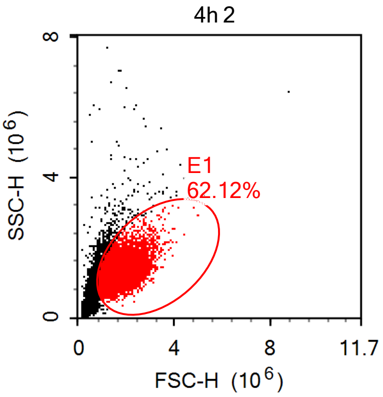

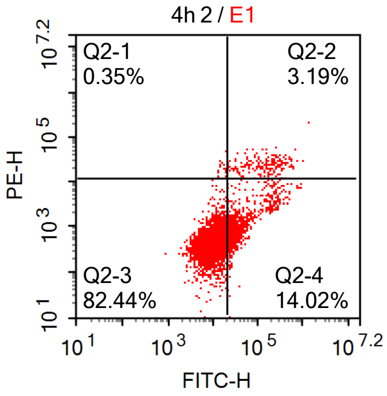

Gate	Count	% All	Mean X	M[illegible]
All	10,000	100.00%	1,280,384	1,[illegible]
E1	6,212	62.12%	1,773,209	1,[illegible]

Gate	Count	% E1	% All	Mean X	Mean Y	Median X	Median Y
E1	6,212	100.00%	62.12%	23,911	1,606	11,523	516
Q2-1	22	0.35%	0.22%	16,351	22,358	16,757	20,960
Q2-2	198	3.19%	1.98%	190,224	26,542	154,300	23,955
Q2-3	5,121	82.44%	51.21%	10,736	550	10,175	473
Q2-4	871	14.02%	8.71%	63,755	1,626	29,609	905

样本统计表格 - 4h 2

Gate	Count	% Parent	% All	X	Y	Mean X	Mean Y	Median X	Median Y
All	10,000								
E1	6,212	62.12%	62.12%	FSC-H	SSC-H	1,773,209	1,298,830	1,730,518	1,250,490
Q2-1	22	0.35%	0.22%	FITC-H	PE-H	16,351	22,358	16,757	20,960
Q2-2	198	3.19%	1.98%	FITC-H	PE-H	190,224	26,542	154,300	23,955
Q2-3	5,121	82.44%	51.21%	FITC-H	PE-H	10,736	550	10,175	473
Q2-4	871	14.02%	8.71%	FITC-H	PE-H	63,755	1,626	29,609	905

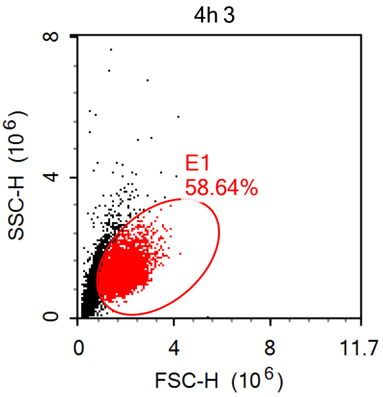

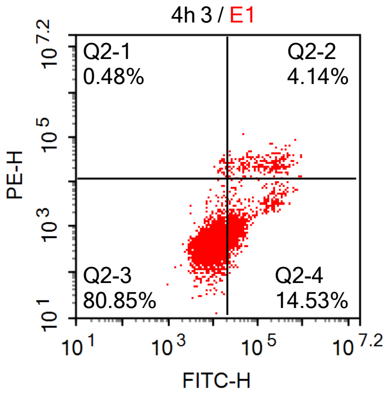

Gate	Count	% All	Mean X	M
All	10,000	100.00%	1,190,088	1,(
E1	5,864	58.64%	1,718,602	1,:

Gate	Count	% E1	% All	Mean X	Mean Y	Median X	Median Y
E1	5,864	100.00%	58.64%	25,620	1,917	11,359	513
Q2-1	28	0.48%	0.28%	15,904	23,630	16,250	21,622
Q2-2	243	4.14%	2.43%	194,285	27,576	155,719	24,224
Q2-3	4,741	80.85%	47.41%	10,577	528	9,850	463
Q2-4	852	14.53%	8.52%	61,540	1,617	29,164	944

样本统计表格 - 4h 3

Gate	Count	% Parent	% All	X	Y	Mean X	Mean Y	Median X	Median Y
All	10,000								
E1	5,864	58.64%	58.64%	FSC-H	SSC-H	1,718,602	1,271,605	1,683,391	1,222,245
Q2-1	28	0.48%	0.28%	FITC-H	PE-H	15,904	23,630	16,250	21,622
Q2-2	243	4.14%	2.43%	FITC-H	PE-H	194,285	27,576	155,719	24,224
Q2-3	4,741	80.85%	47.41%	FITC-H	PE-H	10,577	528	9,850	463
Q2-4	852	14.53%	8.52%	FITC-H	PE-H	61,540	1,617	29,164	944

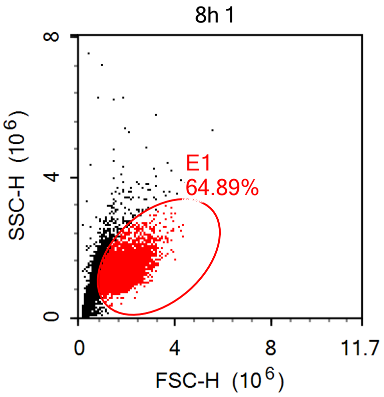

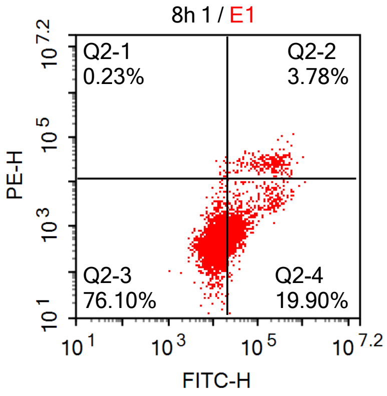

Gate	Count	% All	Mean X	M
All	10,000	100.00%	1,301,059	1,
E1	6,489	64.89%	1,746,990	1,:

Gate	Count	% E1	% All	Mean X	Mean Y	Median X	Median Y
E1	6,489	100.00%	64.89%	28,225	1,839	13,749	531
Q2-1	15	0.23%	0.15%	16,650	20,847	16,701	17,922
Q2-2	245	3.78%	2.45%	215,044	28,403	199,702	26,248
Q2-3	4,938	76.10%	49.38%	12,225	562	11,812	467
Q2-4	1,291	19.90%	12.91%	54,105	1,465	29,220	874

样本统计表格 - 8h 1

Gate	Count	% Parent	% All	X	Y	Mean X	Mean Y	Median X	Median Y
All	10,000								
E1	6,489	64.89%	64.89%	FSC-H	SSC-H	1,746,990	1,321,919	1,694,417	1,267,373
Q2-1	15	0.23%	0.15%	FITC-H	PE-H	16,650	20,847	16,701	17,922
Q2-2	245	3.78%	2.45%	FITC-H	PE-H	215,044	28,403	199,702	26,248
Q2-3	4,938	76.10%	49.38%	FITC-H	PE-H	12,225	562	11,812	467
Q2-4	1,291	19.90%	12.91%	FITC-H	PE-H	54,105	1,465	29,220	874

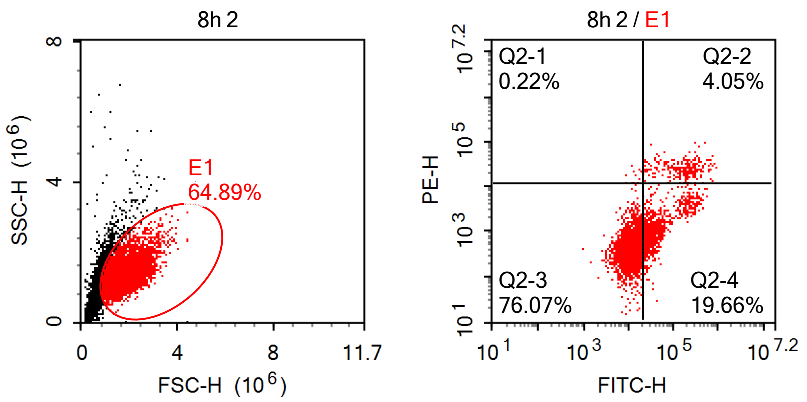

Gate	Count	% All	Mean X	M
All	10,000	100.00%	1,307,954	1,
E1	6,489	64.89%	1,760,060	1,

Gate	Count	% E1	% All	Mean X	Mean Y	Median X	Median Y
E1	6,489	100.00%	64.89%	28,389	1,861	13,598	536
Q2-1	14	0.22%	0.14%	14,828	18,916	14,597	18,084
Q2-2	263	4.05%	2.63%	210,217	27,607	188,585	25,297
Q2-3	4,936	76.07%	49.36%	12,129	545	11,638	468
Q2-4	1,276	19.66%	12.76%	53,963	1,454	28,756	898

样本统计表格 - 8h 2

Gate	Count	% Parent	% All	X	Y	Mean X	Mean Y	Median X	Median Y
All	10,000								
E1	6,489	64.89%	64.89%	FSC-H	SSC-H	1,760,060	1,336,557	1,711,552	1,280,425
Q2-1	14	0.22%	0.14%	FITC-H	PE-H	14,828	18,916	14,597	18,084
Q2-2	263	4.05%	2.63%	FITC-H	PE-H	210,217	27,607	188,585	25,297
Q2-3	4,936	76.07%	49.36%	FITC-H	PE-H	12,129	545	11,638	468
Q2-4	1,276	19.66%	12.76%	FITC-H	PE-H	53,963	1,454	28,756	898

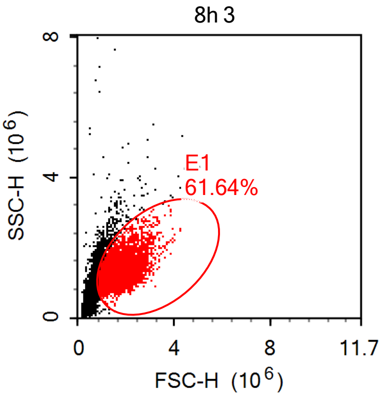

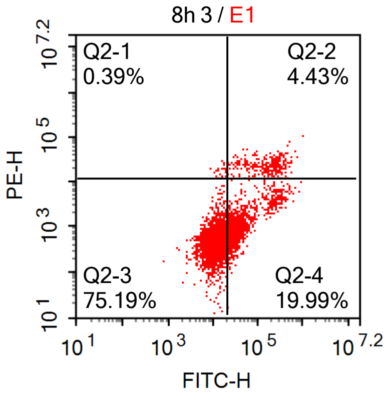

Gate	Count	% All	Mean X	[illegible]
All	10,000	100.00%	1,243,088	[illegible]
E1	6,164	61.64%	1,732,500	[illegible]

Gate	Count	% E1	% All	Mean X	Mean Y	Median X	Median Y
E1	6,164	100.00%	61.64%	29,608	1,955	13,733	541
Q2-1	24	0.39%	0.24%	15,479	19,248	15,584	18,908
Q2-2	273	4.43%	2.73%	195,307	26,226	189,495	24,316
Q2-3	4,635	75.19%	46.35%	12,088	554	11,632	474
Q2-4	1,232	19.99%	12.32%	59,079	1,509	29,147	905

样本统计表格 - 8h 3

Gate	Count	% Parent	% All	X	Y	Mean X	Mean Y	Median X	Median Y
All	10,000								
E1	6,164	61.64%	61.64%	FSC-H	SSC-H	1,732,500	1,324,679	1,687,527	1,270,185
Q2-1	24	0.39%	0.24%	FITC-H	PE-H	15,479	19,248	15,584	18,908
Q2-2	273	4.43%	2.73%	FITC-H	PE-H	195,307	26,226	189,495	24,316
Q2-3	4,635	75.19%	46.35%	FITC-H	PE-H	12,088	554	11,632	474
Q2-4	1,232	19.99%	12.32%	FITC-H	PE-H	59,079	1,509	29,147	905

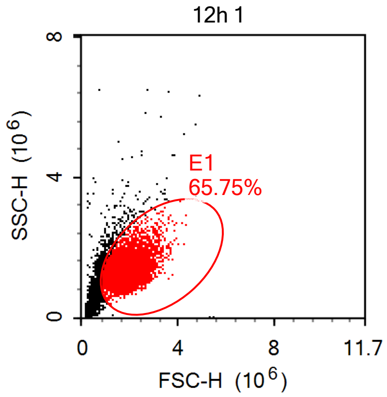

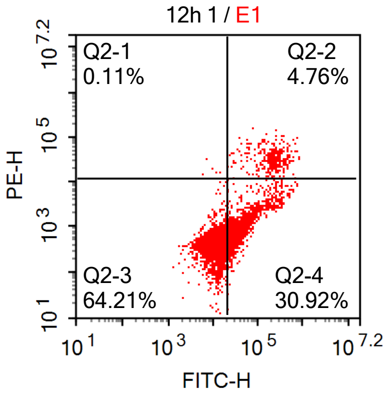

Gate	Count	% All	Mean X	M
All	10,000	100.00%	1,360,984	1,
E1	6,575	65.75%	1,836,188	1,

Gate	Count	% E1	% All	Mean X	Mean Y	Median X	Median Y
E1	6,575	100.00%	65.75%	34,918	2,621	16,495	544
Q2-1	7	0.11%	0.07%	12,525	19,279	11,076	16,875
Q2-2	313	4.76%	3.13%	226,667	40,048	209,005	33,752
Q2-3	4,222	64.21%	42.22%	12,817	491	12,639	448
Q2-4	2,033	30.92%	20.33%	51,373	1,225	31,806	815

样本统计表格 - 12h 1

Gate	Count	% Parent	% All	X	Y	Mean X	Mean Y	Median X	Median Y
All	10,000								
E1	6,575	65.75%	65.75%	FSC-H	SSC-H	1,836,188	1,332,387	1,797,764	1,274,307
Q2-1	7	0.11%	0.07%	FITC-H	PE-H	12,525	19,279	11,076	16,875
Q2-2	313	4.76%	3.13%	FITC-H	PE-H	226,667	40,048	209,005	33,752
Q2-3	4,222	64.21%	42.22%	FITC-H	PE-H	12,817	491	12,639	448
Q2-4	2,033	30.92%	20.33%	FITC-H	PE-H	51,373	1,225	31,806	815

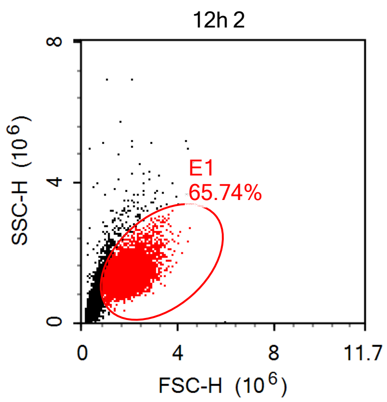

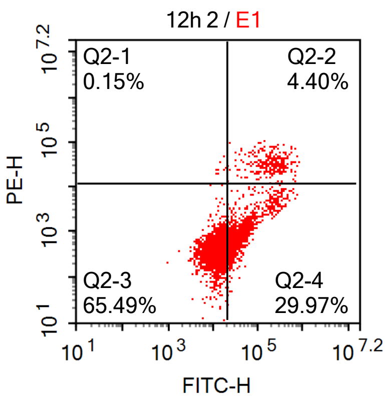

Gate	Count	% All	Mean X	M
All	10,000	100.00%	1,360,628	1,(
E1	6,574	65.74%	1,842,917	1,:

Gate	Count	% E1	% All	Mean X	Mean Y	Median X	Median Y
E1	6,574	100.00%	65.74%	33,420	2,354	16,494	544
Q2-1	10	0.15%	0.10%	15,163	27,256	15,040	25,134
Q2-2	289	4.40%	2.89%	223,881	36,945	201,370	32,603
Q2-3	4,305	65.49%	43.05%	13,002	490	12,770	452
Q2-4	1,970	29.97%	19.70%	50,192	1,226	30,872	801

样本统计表格 - 12h 2

Gate	Count	% Parent	% All	X	Y	Mean X	Mean Y	Median X	Median Y
All	10,000								
E1	6,574	65.74%	65.74%	FSC-H	SSC-H	1,842,917	1,336,683	1,800,774	1,281,729
Q2-1	10	0.15%	0.10%	FITC-H	PE-H	15,163	27,256	15,040	25,134
Q2-2	289	4.40%	2.89%	FITC-H	PE-H	223,881	36,945	201,370	32,603
Q2-3	4,305	65.49%	43.05%	FITC-H	PE-H	13,002	490	12,770	452
Q2-4	1,970	29.97%	19.70%	FITC-H	PE-H	50,192	1,226	30,872	801

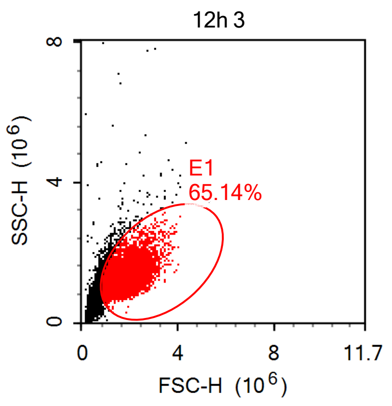

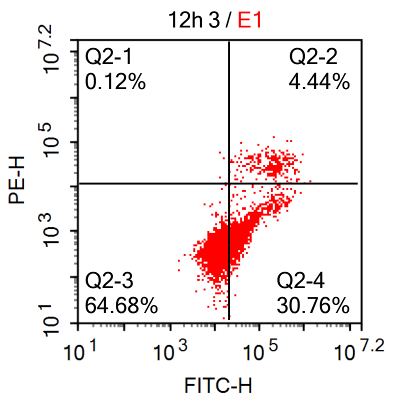

Gate	Count	% All	Mean X	M(
All	10,000	100.00%	1,351,660	1,(
E1	6,514	65.14%	1,848,857	1,:

Gate	Count	% E1	% All	Mean X	Mean Y	Median X	Median Y
E1	6,514	100.00%	65.14%	34,362	2,304	16,411	553
Q2-1	8	0.12%	0.08%	17,431	31,369	18,152	29,697
Q2-2	289	4.44%	2.89%	233,324	35,070	205,713	31,454
Q2-3	4,213	64.68%	42.13%	12,985	491	12,775	452
Q2-4	2,004	30.76%	20.04%	50,678	1,274	31,639	841

样本统计表格 - 12h 3

Gate	Count	% Parent	% All	X	Y	Mean X	Mean Y	Median X	Median Y
All	10,000								
E1	6,514	65.14%	65.14%	FSC-H	SSC-H	1,848,857	1,336,885	1,814,723	1,279,399
Q2-1	8	0.12%	0.08%	FITC-H	PE-H	17,431	31,369	18,152	29,697
Q2-2	289	4.44%	2.89%	FITC-H	PE-H	233,324	35,070	205,713	31,454
Q2-3	4,213	64.68%	42.13%	FITC-H	PE-H	12,985	491	12,775	452
Q2-4	2,004	30.76%	20.04%	FITC-H	PE-H	50,678	1,274	31,639	841
