# Supplementary material for: Mitochondrial calcium uniporter-mediated mitochondrial dynamics imbalance contributes to contrast medium-induced renal tubular cell injury
Source: Front Mol Biosci. 2026 Jun 29;13:1848361. doi: 10.3389/fmolb.2026.1848361 (PMC13357276; doi:10.3389/fmolb.2026.1848361)
Supplement: Supplementary file 1 [file DataSheet3.zip › Flow Cytometry Assay(1,2)/Flow Cytometry Assay-2/╧╕░√╡≥═÷-2/HK-2 ╡≥═÷ 1/▒¿╕μ - HK-2 ╡≥═÷ 1.pdf]

HK-2 凋亡 1 报告

标本名：HK-2 凋亡 1　　检验时间：2024/9/20 11:36

仪器：NovoCyte 451160320945　　软件：NovoExpress 1.2.4

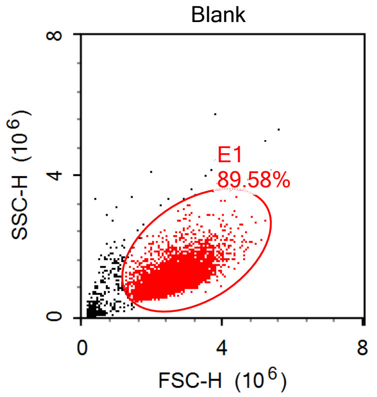

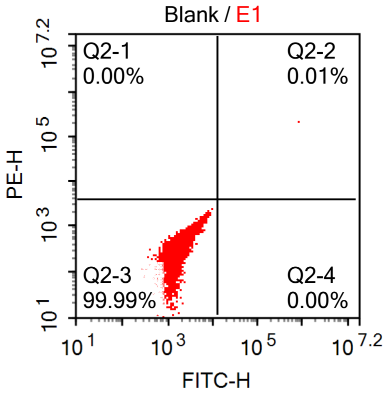

Gate	Count	% All	Mean X	M(
All	10,000	100.00%	2,309,043	1,(
E1	8,958	89.58%	2,535,845	1,(

Gate	Count	% E1	% All	Mean X	Mean Y	Median X	Median Y
E1	8,958	100.00%	89.58%	1,988	434	1,755	384
Q2-1	0	0.00%	0.00%	0	0	0	0
Q2-2	1	0.01%	0.01%	819,107	213,150	819,107	213,150
Q2-3	8,957	99.99%	89.57%	1,897	410	1,755	384
Q2-4	0	0.00%	0.00%	0	0	0	0

样本统计表格 - Blank

Gate	Count	% Parent	% All	X	Y	Mean X	Mean Y	Median X	Median Y
All	10,000								
E1	8,958	89.58%	89.58%	FSC-H	SSC-H	2,535,845	1,075,291	2,517,288	1,009,103
Q2-1	0	0.00%	0.00%	FITC-H	PE-H	0	0	0	0
Q2-2	1	0.01%	0.01%	FITC-H	PE-H	819,107	213,150	819,107	213,150
Q2-3	8,957	99.99%	89.57%	FITC-H	PE-H	1,897	410	1,755	384
Q2-4	0	0.00%	0.00%	FITC-H	PE-H	0	0	0	0

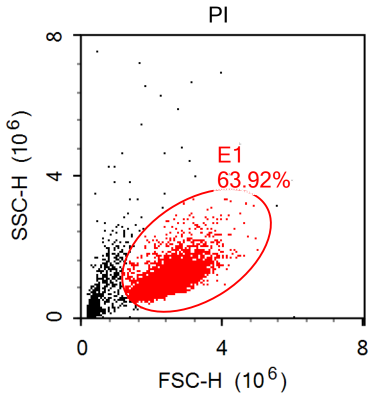

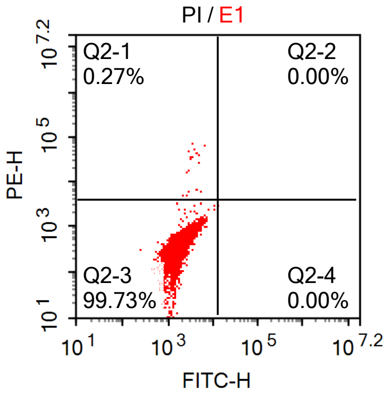

Gate	Count	% All	Mean X	M
All	10,000	100.00%	1,645,796	77
E1	6,392	63.92%	2,433,192	1,(

Gate	Count	% E1	% All	Mean X	Mean Y	Median X	Median Y
E1	6,392	100.00%	63.92%	1,848	493	1,713	381
Q2-1	17	0.27%	0.17%	3,570	30,951	3,325	33,638
Q2-2	0	0.00%	0.00%	0	0	0	0
Q2-3	6,375	99.73%	63.75%	1,843	411	1,711	381
Q2-4	0	0.00%	0.00%	0	0	0	0

样本统计表格 - PI

Gate	Count	% Parent	% All	X	Y	Mean X	Mean Y	Median X	Median Y
All	10,000								
E1	6,392	63.92%	63.92%	FSC-H	SSC-H	2,433,192	1,061,296	2,405,962	984,899
Q2-1	17	0.27%	0.17%	FITC-H	PE-H	3,570	30,951	3,325	33,638
Q2-2	0	0.00%	0.00%	FITC-H	PE-H	0	0	0	0
Q2-3	6,375	99.73%	63.75%	FITC-H	PE-H	1,843	411	1,711	381
Q2-4	0	0.00%	0.00%	FITC-H	PE-H	0	0	0	0

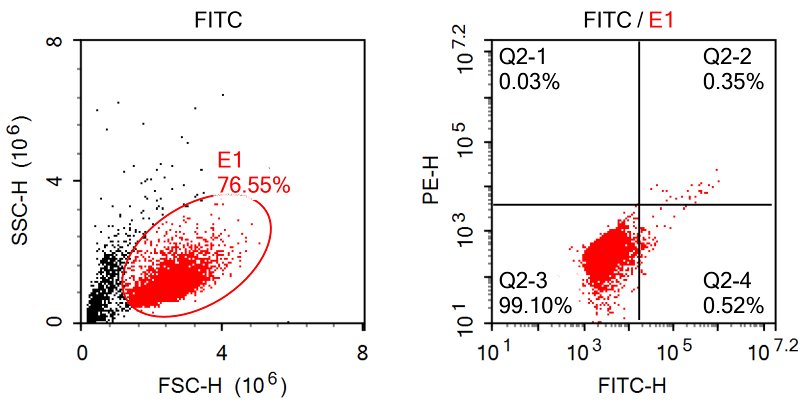

Gate	Count	% All	Mean X	M(
All	10,000	100.00%	1,958,590	93
E1	7,655	76.55%	2,439,243	1,(

Gate	Count	% E1	% All	Mean X	Mean Y	Median X	Median Y
E1	7,655	100.00%	76.55%	4,760	431	3,015	372
Q2-1	2	0.03%	0.02%	14,308	3,956	14,308	3,956
Q2-2	27	0.35%	0.27%	326,857	8,900	282,808	8,317
Q2-3	7,586	99.10%	75.86%	3,458	398	2,999	371
Q2-4	40	0.52%	0.40%	33,779	875	24,636	591

样本统计表格 - FITC

Gate	Count	% Parent	% All	X	Y	Mean X	Mean Y	Median X	Median Y
All	10,000								
E1	7,655	76.55%	76.55%	FSC-H	SSC-H	2,439,243	1,052,263	2,421,853	976,858
Q2-1	2	0.03%	0.02%	FITC-H	PE-H	14,308	3,956	14,308	3,956
Q2-2	27	0.35%	0.27%	FITC-H	PE-H	326,857	8,900	282,808	8,317
Q2-3	7,586	99.10%	75.86%	FITC-H	PE-H	3,458	398	2,999	371
Q2-4	40	0.52%	0.40%	FITC-H	PE-H	33,779	875	24,636	591

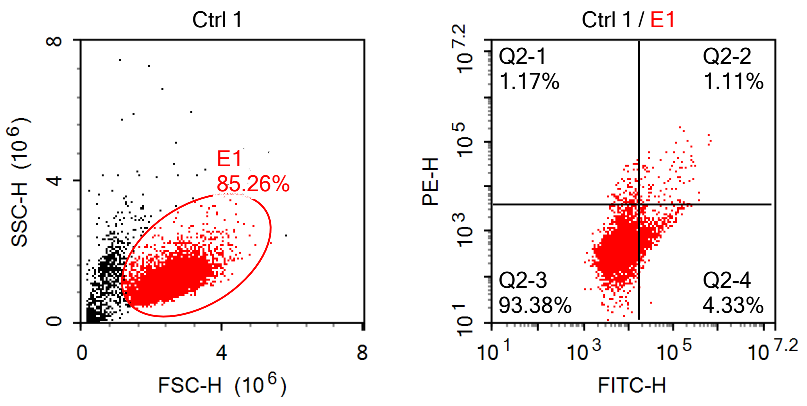

Gate	Count	% All	Mean X	M
All	10,000	100.00%	2,176,174	1,
E1	8,526	85.26%	2,466,760	1,

Gate	Count	% E1	% All	Mean X	Mean Y	Median X	Median Y
E1	8,526	100.00%	85.26%	9,275	952	6,297	465
Q2-1	100	1.17%	1.00%	10,177	9,410	9,783	6,246
Q2-2	95	1.11%	0.95%	114,655	26,980	83,664	13,277
Q2-3	7,962	93.38%	79.62%	6,744	524	6,102	450
Q2-4	369	4.33%	3.69%	36,504	1,193	26,814	960

样本统计表格 - Ctrl 1

Gate	Count	% Parent	% All	X	Y	Mean X	Mean Y	Median X	Median Y
All	10,000								
E1	8,526	85.26%	85.26%	FSC-H	SSC-H	2,466,760	1,104,656	2,458,179	1,040,152
Q2-1	100	1.17%	1.00%	FITC-H	PE-H	10,177	9,410	9,783	6,246
Q2-2	95	1.11%	0.95%	FITC-H	PE-H	114,655	26,980	83,664	13,277
Q2-3	7,962	93.38%	79.62%	FITC-H	PE-H	6,744	524	6,102	450
Q2-4	369	4.33%	3.69%	FITC-H	PE-H	36,504	1,193	26,814	960

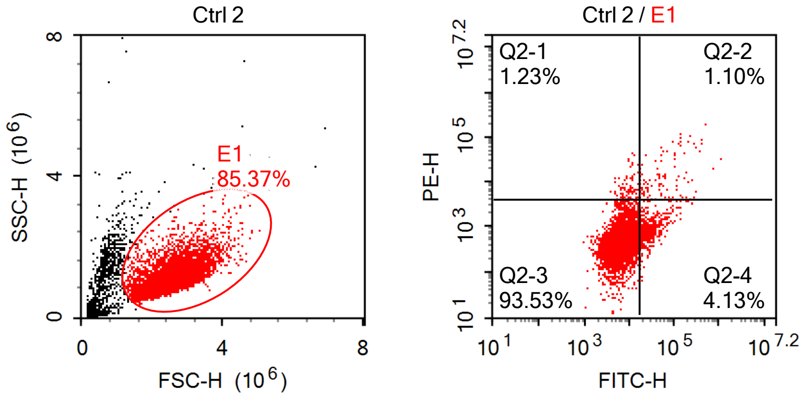

Gate	Count	% All	Mean X	M[illegible]
All	10,000	100.00%	2,221,515	1,[illegible]
E1	8,537	85.37%	2,518,453	1,[illegible]

Gate	Count	% E1	% All	Mean X	Mean Y	Median X	Median Y
E1	8,537	100.00%	85.37%	9,233	958	6,429	482
Q2-1	105	1.23%	1.05%	10,168	9,399	9,598	6,299
Q2-2	94	1.10%	0.94%	117,042	26,320	63,770	10,946
Q2-3	7,985	93.53%	79.85%	6,880	542	6,186	465
Q2-4	353	4.13%	3.53%	33,475	1,106	24,990	899

样本统计表格 - Ctrl 2

Gate	Count	% Parent	% All	X	Y	Mean X	Mean Y	Median X	Median Y
All	10,000								
E1	8,537	85.37%	85.37%	FSC-H	SSC-H	2,518,453	1,124,035	2,494,891	1,057,648
Q2-1	105	1.23%	1.05%	FITC-H	PE-H	10,168	9,399	9,598	6,299
Q2-2	94	1.10%	0.94%	FITC-H	PE-H	117,042	26,320	63,770	10,946
Q2-3	7,985	93.53%	79.85%	FITC-H	PE-H	6,880	542	6,186	465
Q2-4	353	4.13%	3.53%	FITC-H	PE-H	33,475	1,106	24,990	899

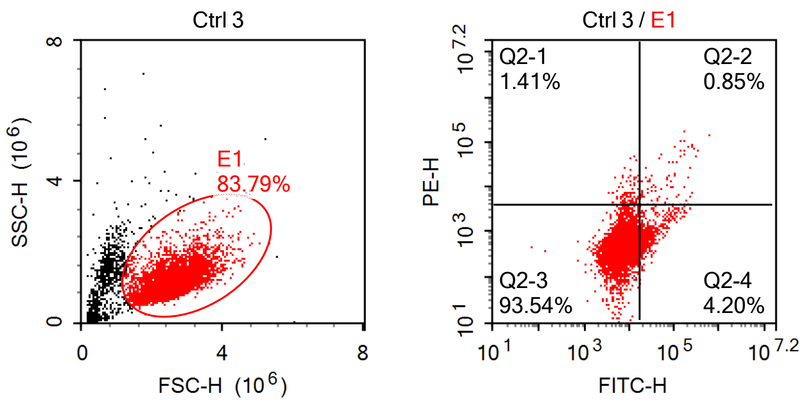

Gate	Count	% All	Mean X	M[illegible]
All	10,000	100.00%	2,172,767	1,[illegible]
E1	8,379	83.79%	2,496,931	1,[illegible]

Gate	Count	% E1	% All	Mean X	Mean Y	Median X	Median Y
E1	8,379	100.00%	83.79%	9,096	919	6,312	474
Q2-1	118	1.41%	1.18%	10,482	8,547	9,862	5,871
Q2-2	71	0.85%	0.71%	106,947	28,466	74,777	13,072
Q2-3	7,838	93.54%	78.38%	6,784	540	6,102	459
Q2-4	352	4.20%	3.52%	40,377	1,245	27,920	963

样本统计表格 - Ctrl 3

Gate	Count	% Parent	% All	X	Y	Mean X	Mean Y	Median X	Median Y
All	10,000								
E1	8,379	83.79%	83.79%	FSC-H	SSC-H	2,496,931	1,103,966	2,480,458	1,033,305
Q2-1	118	1.41%	1.18%	FITC-H	PE-H	10,482	8,547	9,862	5,871
Q2-2	71	0.85%	0.71%	FITC-H	PE-H	106,947	28,466	74,777	13,072
Q2-3	7,838	93.54%	78.38%	FITC-H	PE-H	6,784	540	6,102	459
Q2-4	352	4.20%	3.52%	FITC-H	PE-H	40,377	1,245	27,920	963

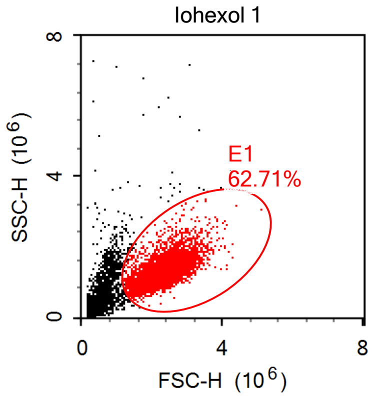

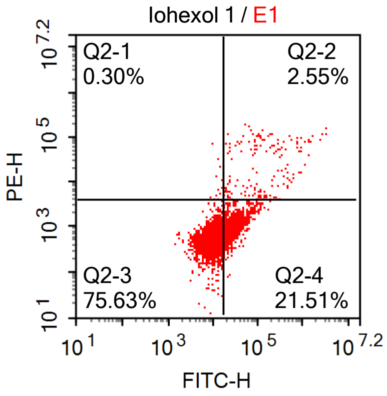

Gate	Count	% All	Mean X	M(
All	10,000	100.00%	1,555,524	97
E1	6,271	62.71%	2,266,840	1,:

Gate	Count	% E1	% All	Mean X	Mean Y	Median X	Median Y
E1	6,271	100.00%	62.71%	22,490	1,920	12,769	690
Q2-1	19	0.30%	0.19%	13,938	12,386	13,625	6,784
Q2-2	160	2.55%	1.60%	301,726	44,574	111,300	22,363
Q2-3	4,743	75.63%	47.43%	11,117	654	10,906	613
Q2-4	1,349	21.51%	13.49%	29,480	1,163	24,592	1,022

样本统计表格 - Iohexol 1

Gate	Count	% Parent	% All	X	Y	Mean X	Mean Y	Median X	Median Y
All	10,000								
E1	6,271	62.71%	62.71%	FSC-H	SSC-H	2,266,840	1,271,500	2,243,236	1,211,011
Q2-1	19	0.30%	0.19%	FITC-H	PE-H	13,938	12,386	13,625	6,784
Q2-2	160	2.55%	1.60%	FITC-H	PE-H	301,726	44,574	111,300	22,363
Q2-3	4,743	75.63%	47.43%	FITC-H	PE-H	11,117	654	10,906	613
Q2-4	1,349	21.51%	13.49%	FITC-H	PE-H	29,480	1,163	24,592	1,022

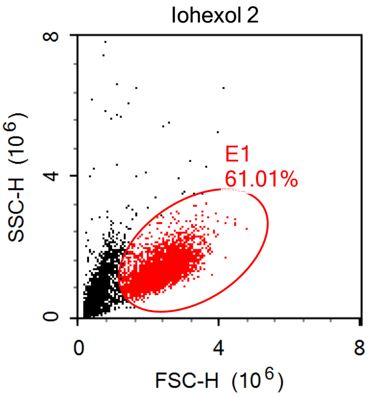

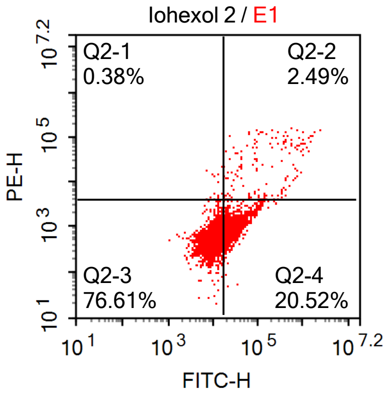

Gate	Count	% All	Mean X	M
All	10,000	100.00%	1,514,444	96
E1	6,101	61.01%	2,252,488	1,:

Gate	Count	% E1	% All	Mean X	Mean Y	Median X	Median Y
E1	6,101	100.00%	61.01%	23,247	1,896	12,627	683
Q2-1	23	0.38%	0.23%	12,349	8,660	12,082	4,900
Q2-2	152	2.49%	1.52%	335,374	45,021	149,759	25,582
Q2-3	4,674	76.61%	46.74%	11,199	658	10,974	614
Q2-4	1,252	20.52%	12.52%	30,531	1,160	24,297	1,011

样本统计表格 - Iohexol 2

Gate	Count	% Parent	% All	X	Y	Mean X	Mean Y	Median X	Median Y
All	10,000								
E1	6,101	61.01%	61.01%	FSC-H	SSC-H	2,252,488	1,267,159	2,228,460	1,205,529
Q2-1	23	0.38%	0.23%	FITC-H	PE-H	12,349	8,660	12,082	4,900
Q2-2	152	2.49%	1.52%	FITC-H	PE-H	335,374	45,021	149,759	25,582
Q2-3	4,674	76.61%	46.74%	FITC-H	PE-H	11,199	658	10,974	614
Q2-4	1,252	20.52%	12.52%	FITC-H	PE-H	30,531	1,160	24,297	1,011

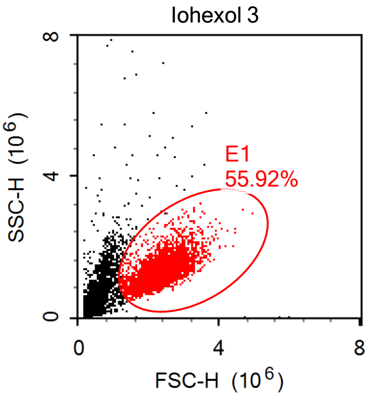

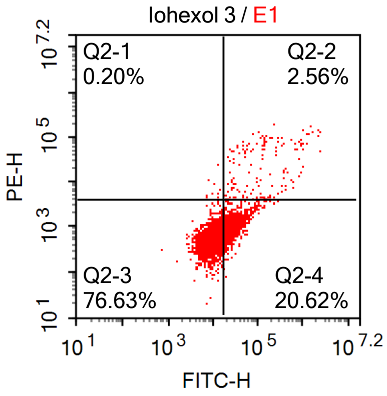

Gate	Count	% All	Mean X	M(
All	10,000	100.00%	1,425,396	92
E1	5,592	55.92%	2,266,774	1,:

Gate	Count	% E1	% All	Mean X	Mean Y	Median X	Median Y
E1	5,592	100.00%	55.92%	22,269	1,839	12,386	684
Q2-1	11	0.20%	0.11%	11,466	8,071	12,011	5,610
Q2-2	143	2.56%	1.43%	303,578	42,350	116,369	25,793
Q2-3	4,285	76.63%	42.85%	10,978	653	10,751	611
Q2-4	1,153	20.62%	11.53%	29,446	1,160	23,995	1,029

样本统计表格 - Iohexol 3

Gate	Count	% Parent	% All	X	Y	Mean X	Mean Y	Median X	Median Y
All	10,000								
E1	5,592	55.92%	55.92%	FSC-H	SSC-H	2,266,774	1,263,420	2,239,310	1,199,456
Q2-1	11	0.20%	0.11%	FITC-H	PE-H	11,466	8,071	12,011	5,610
Q2-2	143	2.56%	1.43%	FITC-H	PE-H	303,578	42,350	116,369	25,793
Q2-3	4,285	76.63%	42.85%	FITC-H	PE-H	10,978	653	10,751	611
Q2-4	1,153	20.62%	11.53%	FITC-H	PE-H	29,446	1,160	23,995	1,029

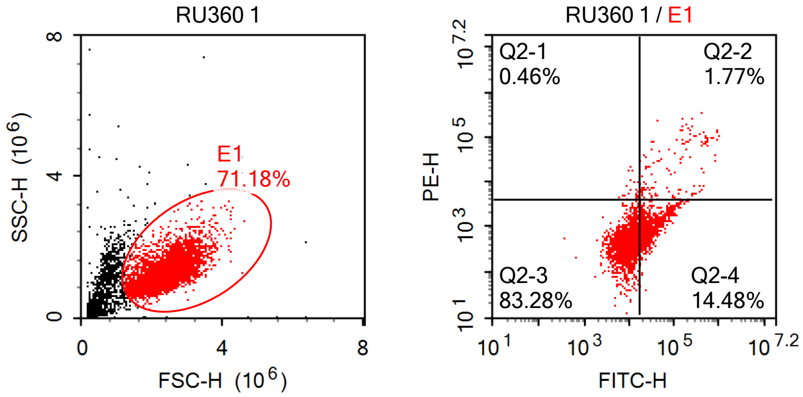

Gate	Count	% All	Mean X	M(
All	10,000	100.00%	1,748,664	1,(
E1	7,118	71.18%	2,298,330	1,:

Gate	Count	% E1	% All	Mean X	Mean Y	Median X	Median Y
E1	7,118	100.00%	71.18%	17,301	1,634	11,125	558
Q2-1	33	0.46%	0.33%	13,037	8,449	13,911	5,976
Q2-2	126	1.77%	1.26%	216,066	54,614	137,039	23,088
Q2-3	5,928	83.28%	59.28%	10,510	563	10,183	512
Q2-4	1,031	14.48%	10.31%	32,188	1,100	24,464	918

样本统计表格 - RU360 1

Gate	Count	% Parent	% All	X	Y	Mean X	Mean Y	Median X	Median Y
All	10,000								
E1	7,118	71.18%	71.18%	FSC-H	SSC-H	2,298,330	1,222,698	2,272,575	1,161,894
Q2-1	33	0.46%	0.33%	FITC-H	PE-H	13,037	8,449	13,911	5,976
Q2-2	126	1.77%	1.26%	FITC-H	PE-H	216,066	54,614	137,039	23,088
Q2-3	5,928	83.28%	59.28%	FITC-H	PE-H	10,510	563	10,183	512
Q2-4	1,031	14.48%	10.31%	FITC-H	PE-H	32,188	1,100	24,464	918

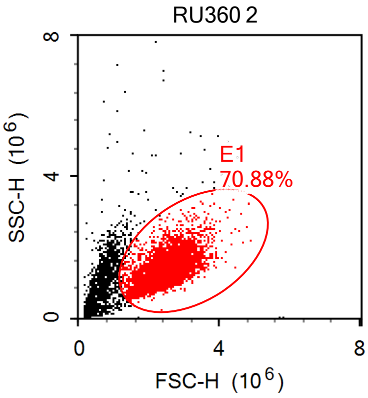

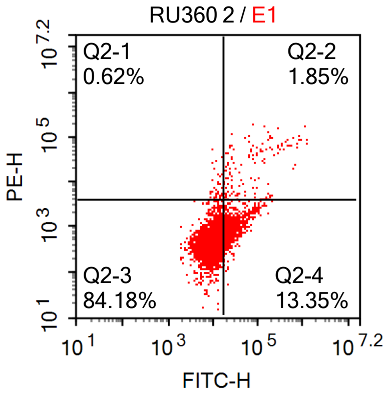

Gate	Count	% All	Mean X	M[illegible]
All	10,000	100.00%	1,815,145	1,[illegible]
E1	7,088	70.88%	2,374,622	1,[illegible]

Gate	Count	% E1	% All	Mean X	Mean Y	Median X	Median Y
E1	7,088	100.00%	70.88%	16,012	1,511	10,865	593
Q2-1	44	0.62%	0.44%	13,198	11,830	13,010	6,512
Q2-2	131	1.85%	1.31%	175,611	42,289	86,335	33,645
Q2-3	5,967	84.18%	59.67%	10,333	603	9,975	548
Q2-4	946	13.35%	9.46%	29,866	1,108	23,537	968

样本统计表格 - RU360 2

Gate	Count	% Parent	% All	X	Y	Mean X	Mean Y	Median X	Median Y
All	10,000								
E1	7,088	70.88%	70.88%	FSC-H	SSC-H	2,374,622	1,290,934	2,354,564	1,219,348
Q2-1	44	0.62%	0.44%	FITC-H	PE-H	13,198	11,830	13,010	6,512
Q2-2	131	1.85%	1.31%	FITC-H	PE-H	175,611	42,289	86,335	33,645
Q2-3	5,967	84.18%	59.67%	FITC-H	PE-H	10,333	603	9,975	548
Q2-4	946	13.35%	9.46%	FITC-H	PE-H	29,866	1,108	23,537	968

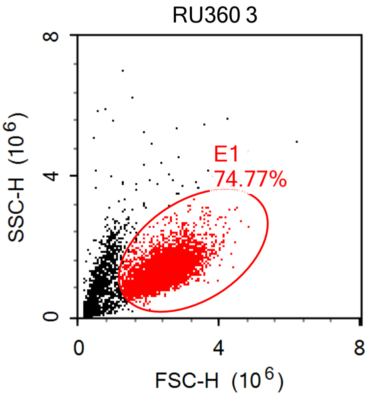

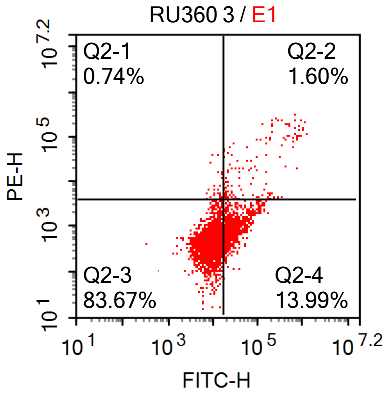

Gate	Count	% All	Mean X	M
All	10,000	100.00%	1,827,145	1,(
E1	7,477	74.77%	2,294,160	1,:

Gate	Count	% E1	% All	Mean X	Mean Y	Median X	Median Y
E1	7,477	100.00%	74.77%	17,044	1,671	11,106	543
Q2-1	55	0.74%	0.55%	14,039	10,274	14,899	5,682
Q2-2	120	1.60%	1.20%	243,265	61,589	141,520	20,941
Q2-3	6,256	83.67%	62.56%	10,485	551	10,175	503
Q2-4	1,046	13.99%	10.46%	30,480	1,046	24,228	888

样本统计表格 - RU360 3

Gate	Count	% Parent	% All	X	Y	Mean X	Mean Y	Median X	Median Y
All	10,000								
E1	7,477	74.77%	74.77%	FSC-H	SSC-H	2,294,160	1,234,980	2,272,393	1,168,588
Q2-1	55	0.74%	0.55%	FITC-H	PE-H	14,039	10,274	14,899	5,682
Q2-2	120	1.60%	1.20%	FITC-H	PE-H	243,265	61,589	141,520	20,941
Q2-3	6,256	83.67%	62.56%	FITC-H	PE-H	10,485	551	10,175	503
Q2-4	1,046	13.99%	10.46%	FITC-H	PE-H	30,480	1,046	24,228	888

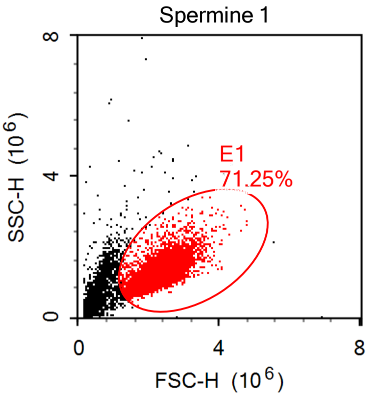

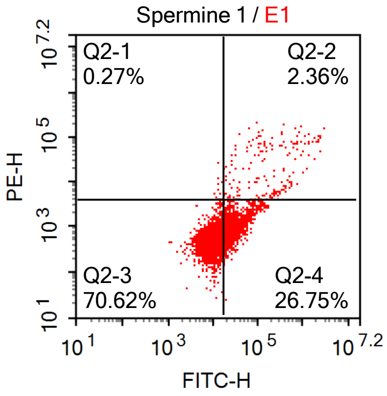

Gate	Count	% All	Mean X	M
All	10,000	100.00%	1,741,334	1,(
E1	7,125	71.25%	2,265,858	1,:

Gate	Count	% E1	% All	Mean X	Mean Y	Median X	Median Y
E1	7,125	100.00%	71.25%	26,170	1,806	13,723	684
Q2-1	19	0.27%	0.19%	14,061	8,609	13,752	5,449
Q2-2	168	2.36%	1.68%	413,050	43,687	186,657	20,830
Q2-3	5,032	70.62%	50.32%	11,549	639	11,470	594
Q2-4	1,906	26.75%	19.06%	30,791	1,128	25,024	976

样本统计表格 - Spermine 1

Gate	Count	% Parent	% All	X	Y	Mean X	Mean Y	Median X	Median Y
All	10,000								
E1	7,125	71.25%	71.25%	FSC-H	SSC-H	2,265,858	1,263,571	2,227,263	1,199,567
Q2-1	19	0.27%	0.19%	FITC-H	PE-H	14,061	8,609	13,752	5,449
Q2-2	168	2.36%	1.68%	FITC-H	PE-H	413,050	43,687	186,657	20,830
Q2-3	5,032	70.62%	50.32%	FITC-H	PE-H	11,549	639	11,470	594
Q2-4	1,906	26.75%	19.06%	FITC-H	PE-H	30,791	1,128	25,024	976

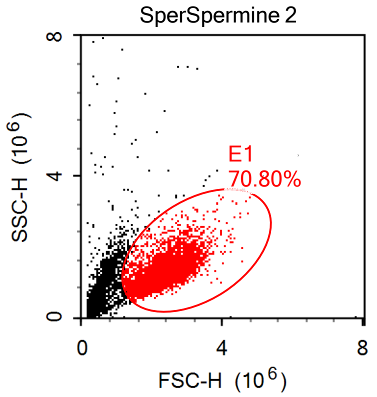

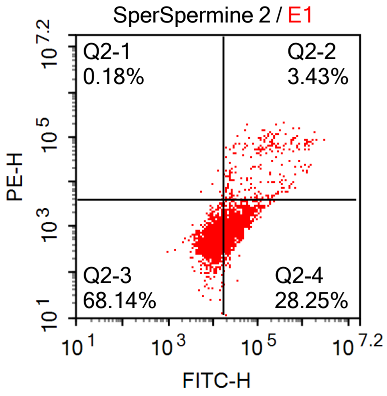

Gate	Count	% All	Mean X	M[illegible]
All	10,000	100.00%	1,740,568	1,[illegible]
E1	7,080	70.80%	2,273,707	1,[illegible]

Gate	Count	% E1	% All	Mean X	Mean Y	Median X	Median Y
E1	7,080	100.00%	70.80%	26,669	2,269	14,443	706
Q2-1	13	0.18%	0.13%	14,948	9,279	16,229	6,977
Q2-2	243	3.43%	2.43%	296,163	43,425	146,067	24,613
Q2-3	4,824	68.14%	48.24%	11,879	658	11,883	611
Q2-4	2,000	28.25%	20.00%	29,675	1,107	24,679	979

样本统计表格 - SperSpermine 2

Gate	Count	% Parent	% All	X	Y	Mean X	Mean Y	Median X	Median Y
All	10,000								
E1	7,080	70.80%	70.80%	FSC-H	SSC-H	2,273,707	1,258,327	2,244,829	1,191,363
Q2-1	13	0.18%	0.13%	FITC-H	PE-H	14,948	9,279	16,229	6,977
Q2-2	243	3.43%	2.43%	FITC-H	PE-H	296,163	43,425	146,067	24,613
Q2-3	4,824	68.14%	48.24%	FITC-H	PE-H	11,879	658	11,883	611
Q2-4	2,000	28.25%	20.00%	FITC-H	PE-H	29,675	1,107	24,679	979

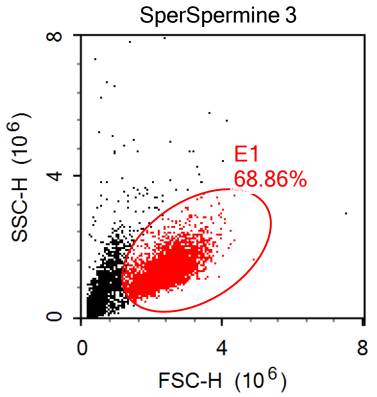

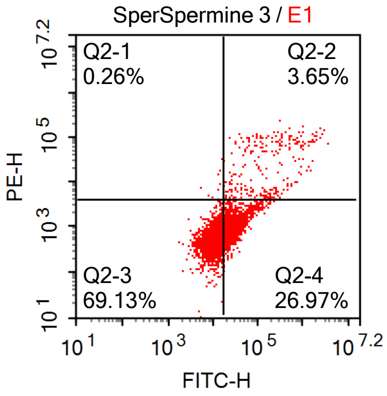

Gate	Count	% All	Mean X	M
All	10,000	100.00%	1,702,078	1,(
E1	6,886	68.86%	2,272,645	1,:

Gate	Count	% E1	% All	Mean X	Mean Y	Median X	Median Y
E1	6,886	100.00%	68.86%	29,758	2,451	14,200	712
Q2-1	18	0.26%	0.18%	14,261	10,003	15,860	6,365
Q2-2	251	3.65%	2.51%	368,332	45,323	154,308	24,723
Q2-3	4,760	69.13%	47.60%	11,830	662	11,723	617
Q2-4	1,857	26.97%	18.57%	30,100	1,170	24,687	1,014

样本统计表格 - SperSpermine 3

Gate	Count	% Parent	% All	X	Y	Mean X	Mean Y	Median X	Median Y
All	10,000								
E1	6,886	68.86%	68.86%	FSC-H	SSC-H	2,272,645	1,259,465	2,241,999	1,200,577
Q2-1	18	0.26%	0.18%	FITC-H	PE-H	14,261	10,003	15,860	6,365
Q2-2	251	3.65%	2.51%	FITC-H	PE-H	368,332	45,323	154,308	24,723
Q2-3	4,760	69.13%	47.60%	FITC-H	PE-H	11,830	662	11,723	617
Q2-4	1,857	26.97%	18.57%	FITC-H	PE-H	30,100	1,170	24,687	1,014
