# Supplementary figures and images for: Mitochondrial calcium uniporter-mediated mitochondrial dynamics imbalance contributes to contrast medium-induced renal tubular cell injury (part 2 of 4)
Source: Front Mol Biosci. 2026 Jun 29;13:1848361. doi: 10.3389/fmolb.2026.1848361 (PMC13357276; doi:10.3389/fmolb.2026.1848361)

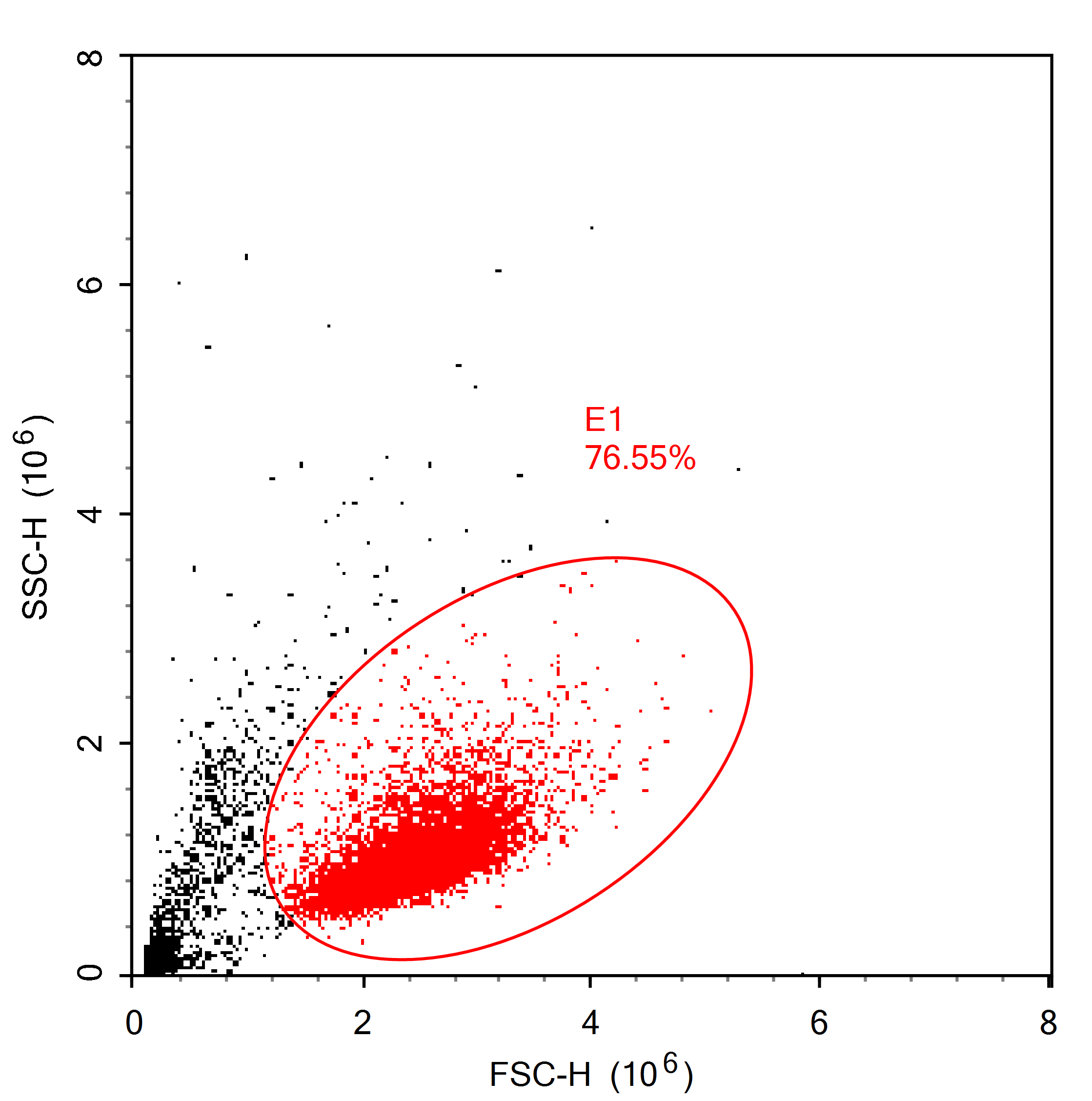

Supplement: Supplementary file 1 [file DataSheet3.zip › Flow Cytometry Assay(1,2)/Flow Cytometry Assay-2/╧╕░√╡≥═÷-2/HK-2 ╡≥═÷ 1/═╝╞1⁄4/FITC/═╝1.tiff]

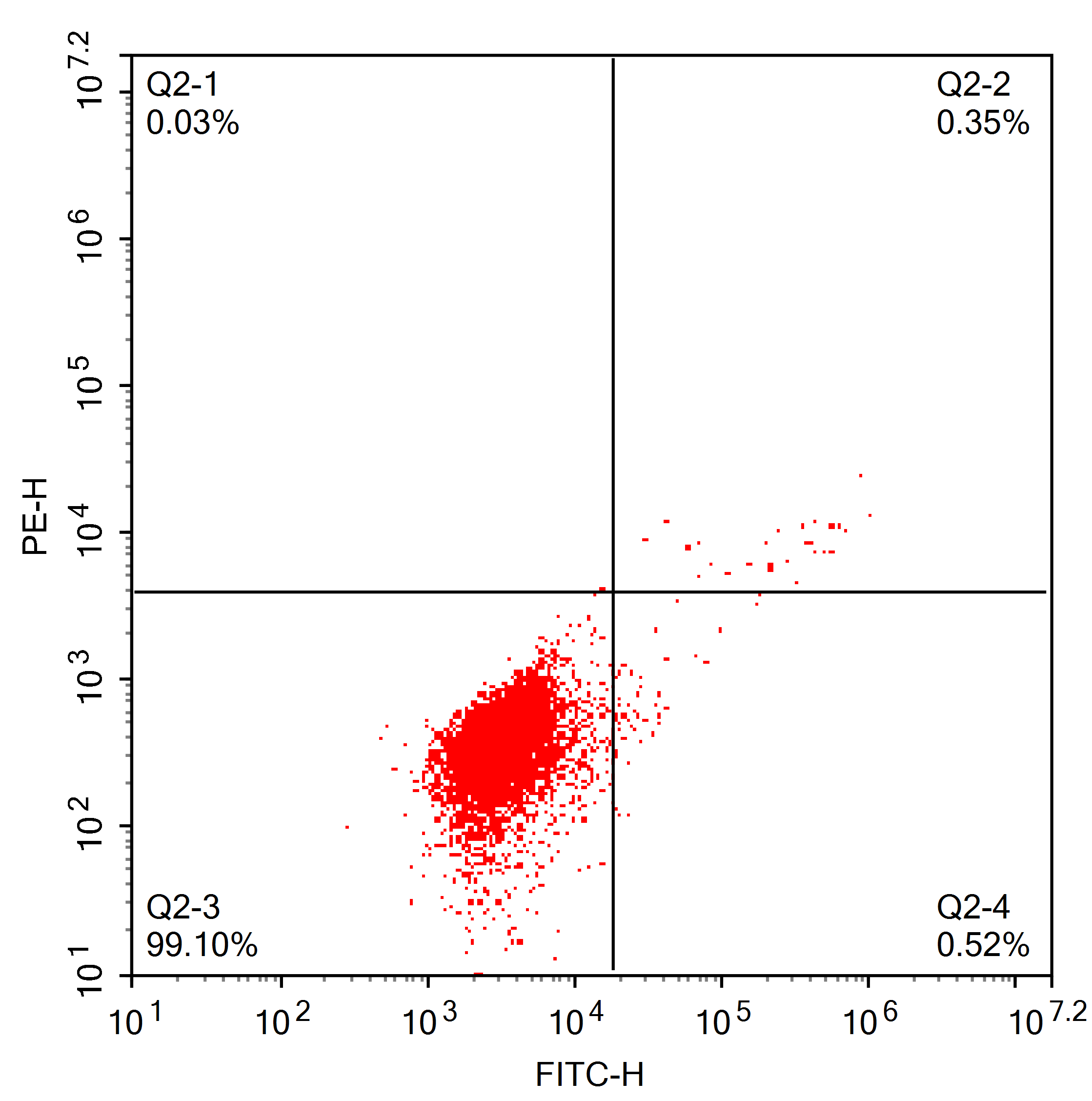

Supplement: Supplementary file 1 [file DataSheet3.zip › Flow Cytometry Assay(1,2)/Flow Cytometry Assay-2/╧╕░√╡≥═÷-2/HK-2 ╡≥═÷ 1/═╝╞1⁄4/FITC/═╝2.tiff]

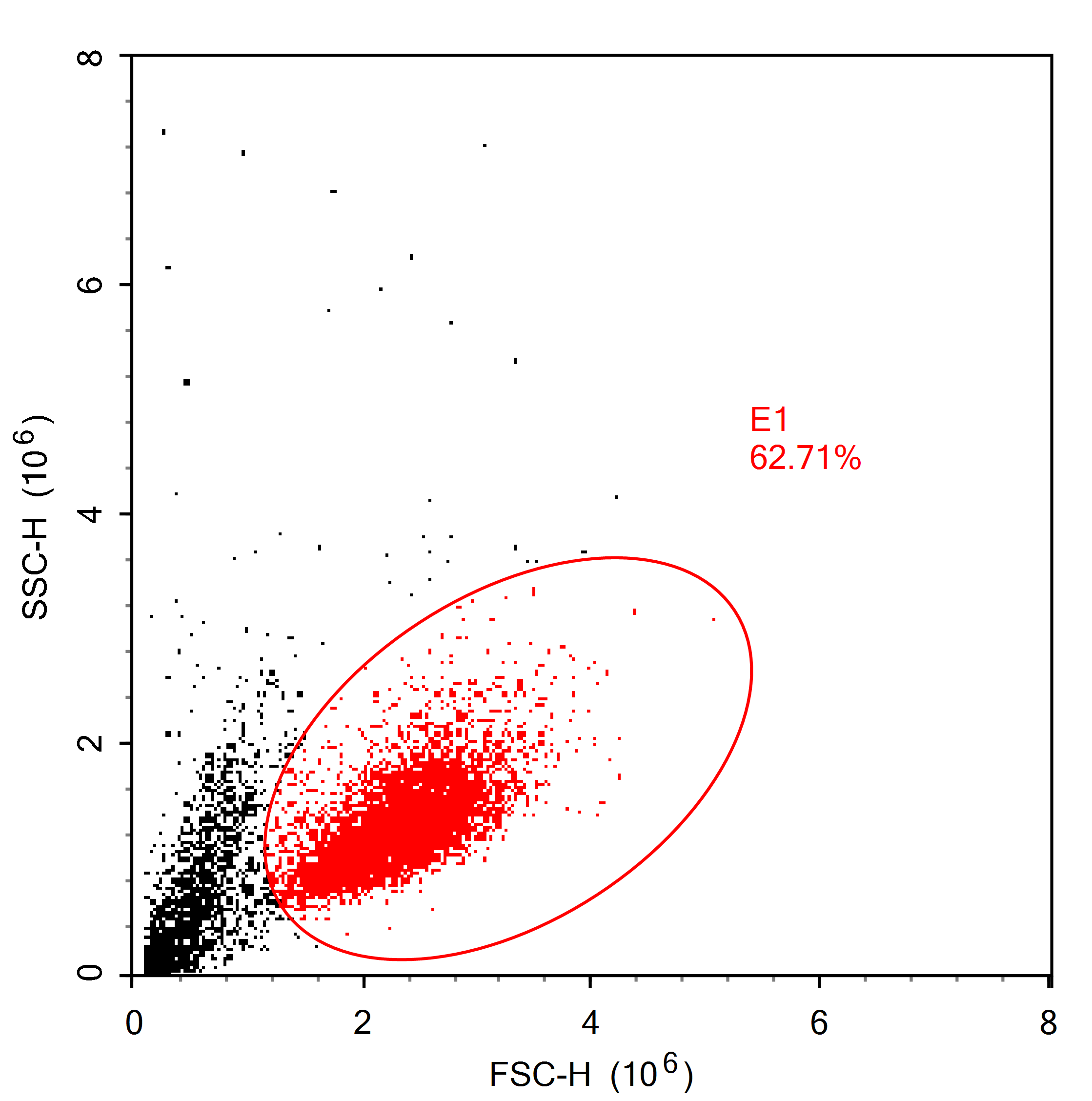

Supplement: Supplementary file 1 [file DataSheet3.zip › Flow Cytometry Assay(1,2)/Flow Cytometry Assay-2/╧╕░√╡≥═÷-2/HK-2 ╡≥═÷ 1/═╝╞1⁄4/Iohexol 1/═╝1.tiff]

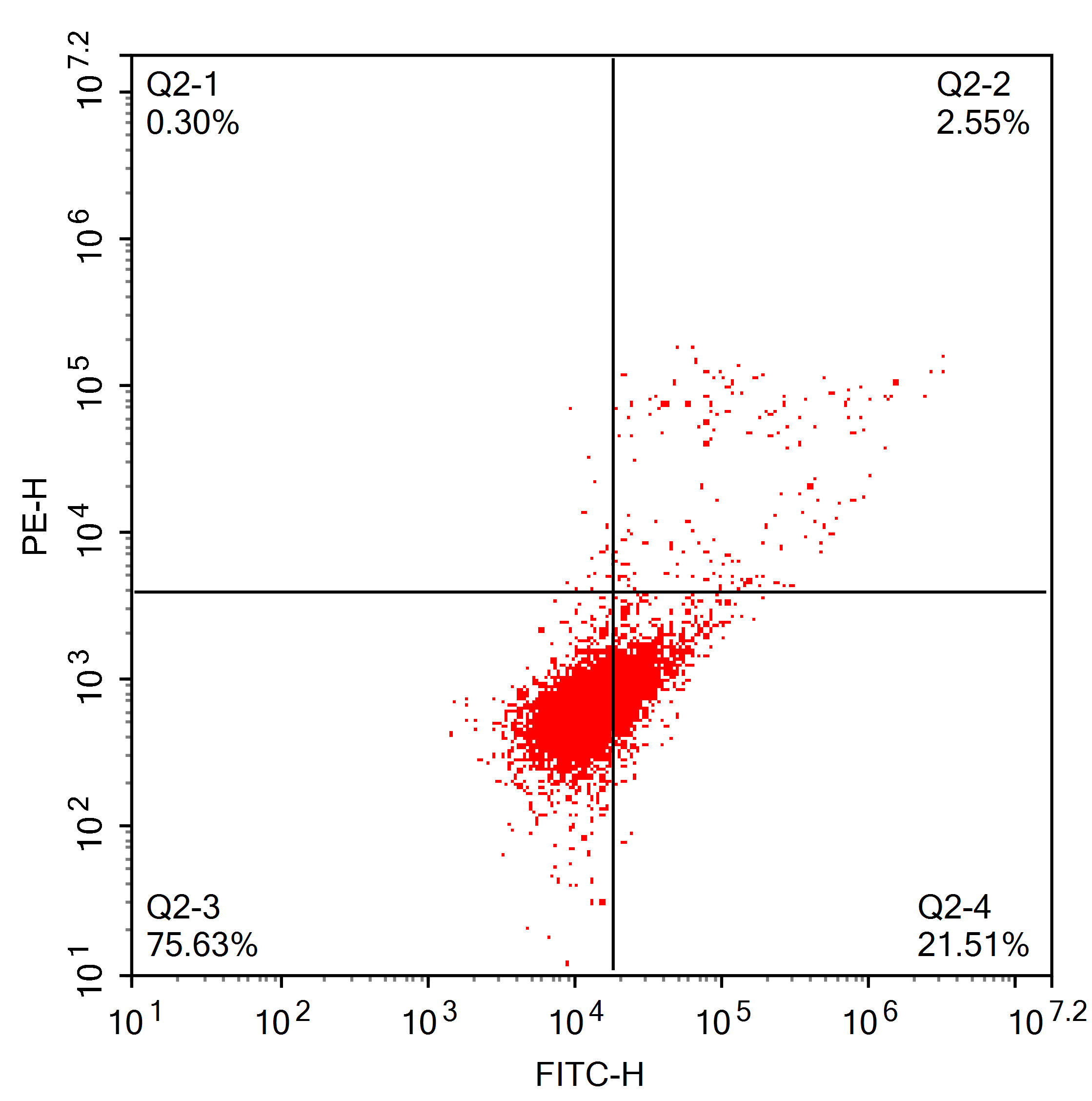

Supplement: Supplementary file 1 [file DataSheet3.zip › Flow Cytometry Assay(1,2)/Flow Cytometry Assay-2/╧╕░√╡≥═÷-2/HK-2 ╡≥═÷ 1/═╝╞1⁄4/Iohexol 1/═╝2.tiff]

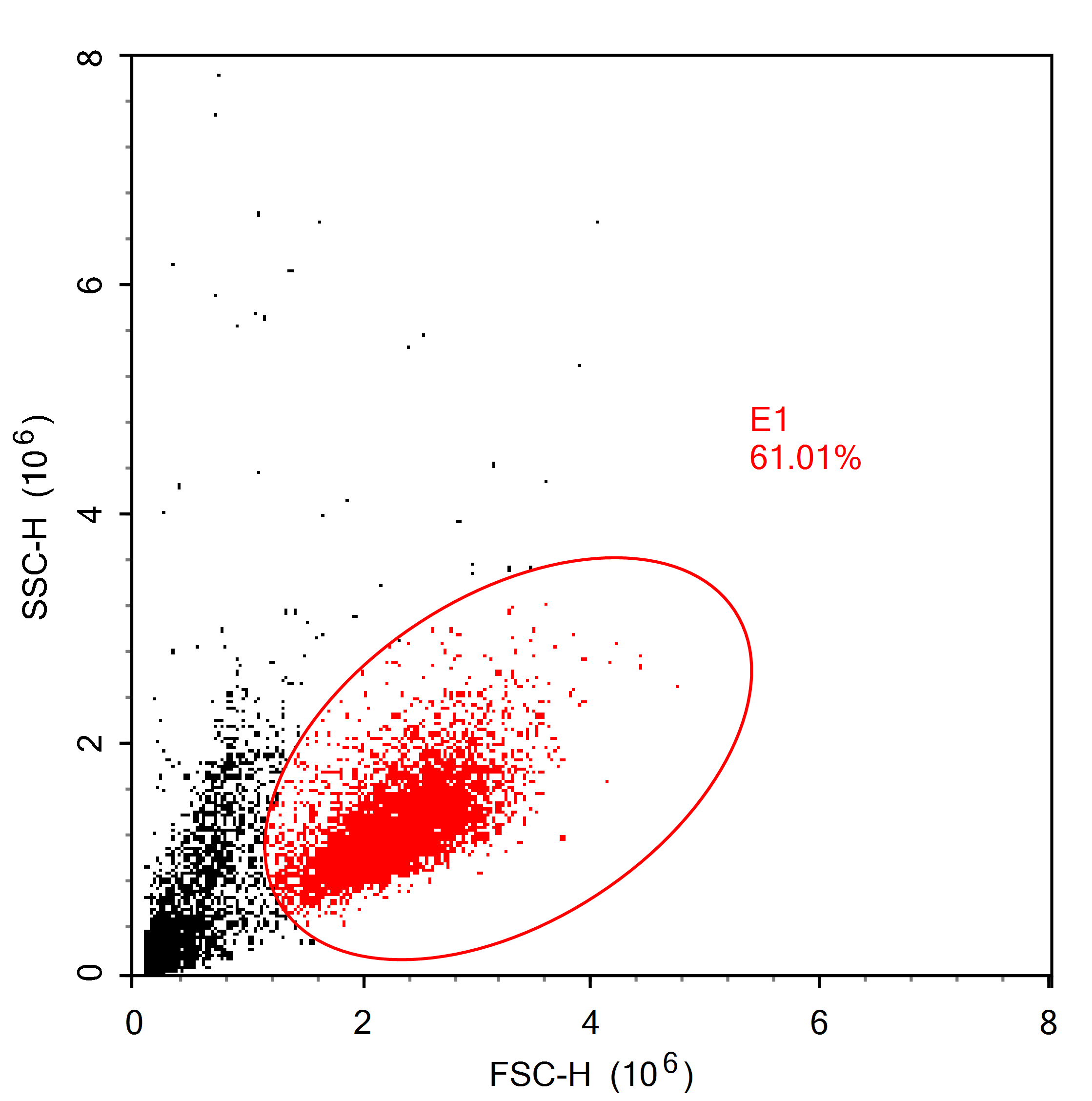

Supplement: Supplementary file 1 [file DataSheet3.zip › Flow Cytometry Assay(1,2)/Flow Cytometry Assay-2/╧╕░√╡≥═÷-2/HK-2 ╡≥═÷ 1/═╝╞1⁄4/Iohexol 2/═╝1.tiff]

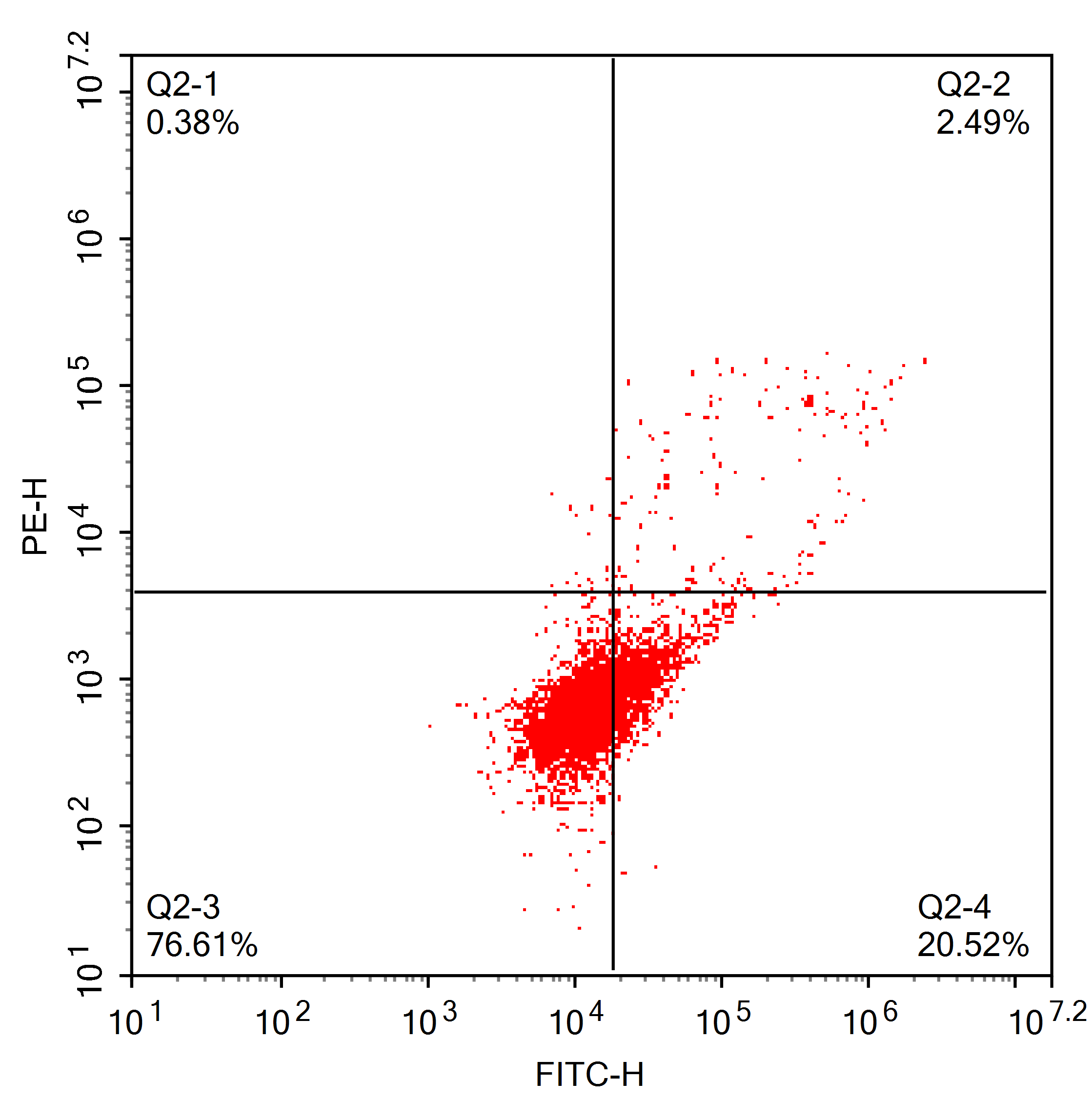

Supplement: Supplementary file 1 [file DataSheet3.zip › Flow Cytometry Assay(1,2)/Flow Cytometry Assay-2/╧╕░√╡≥═÷-2/HK-2 ╡≥═÷ 1/═╝╞1⁄4/Iohexol 2/═╝2.tiff]

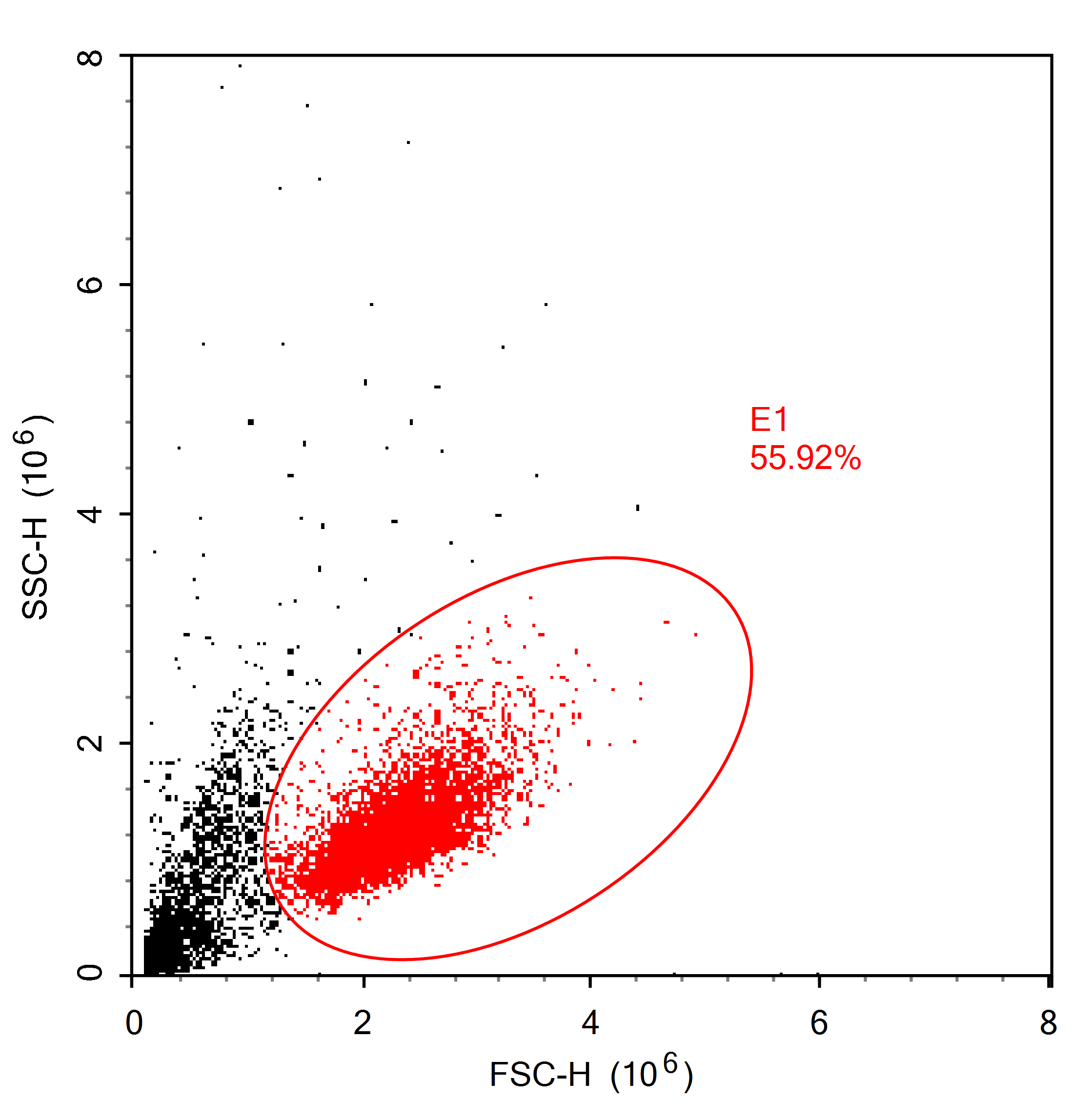

Supplement: Supplementary file 1 [file DataSheet3.zip › Flow Cytometry Assay(1,2)/Flow Cytometry Assay-2/╧╕░√╡≥═÷-2/HK-2 ╡≥═÷ 1/═╝╞1⁄4/Iohexol 3/═╝1.tiff]

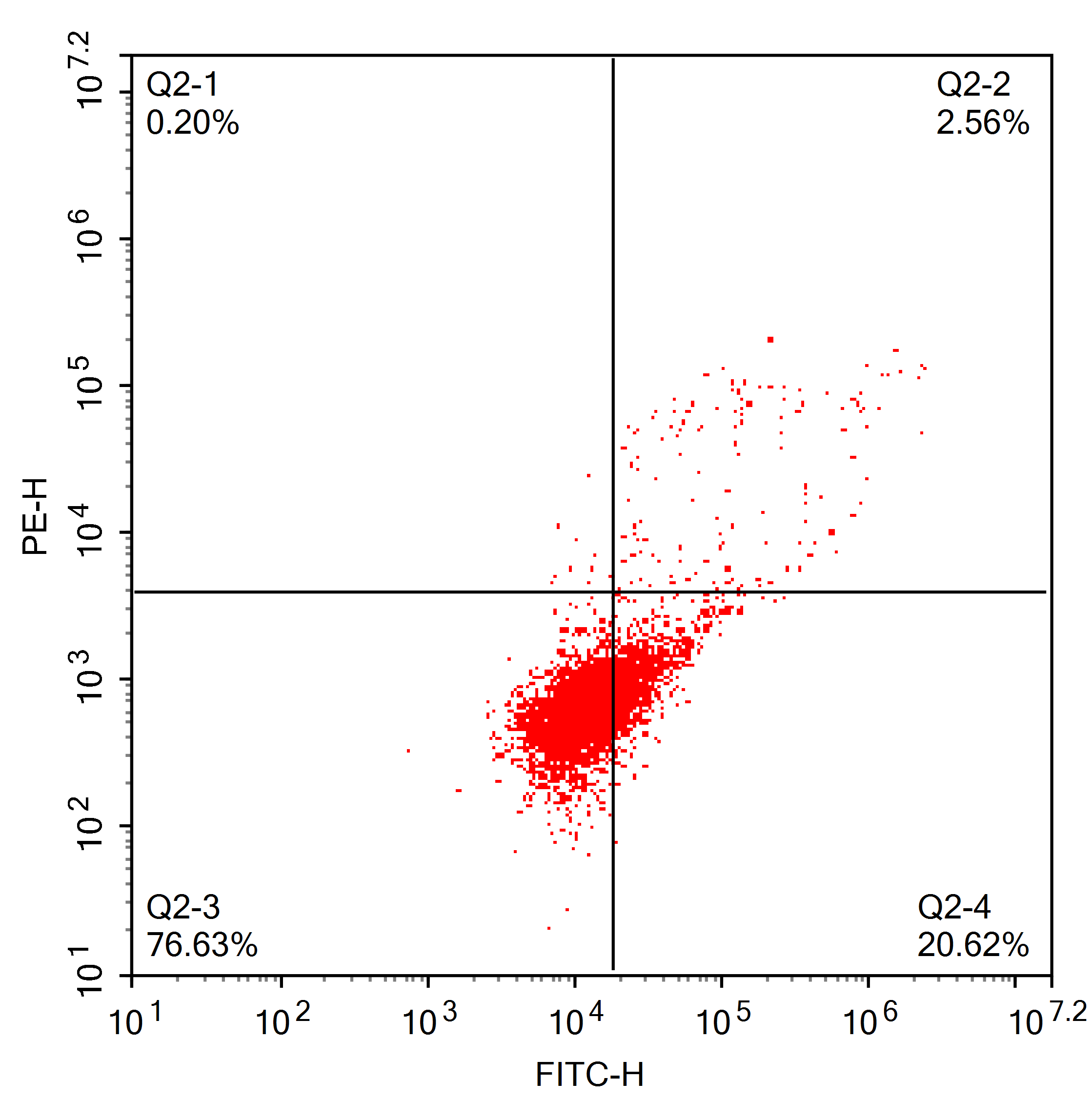

Supplement: Supplementary file 1 [file DataSheet3.zip › Flow Cytometry Assay(1,2)/Flow Cytometry Assay-2/╧╕░√╡≥═÷-2/HK-2 ╡≥═÷ 1/═╝╞1⁄4/Iohexol 3/═╝2.tiff]

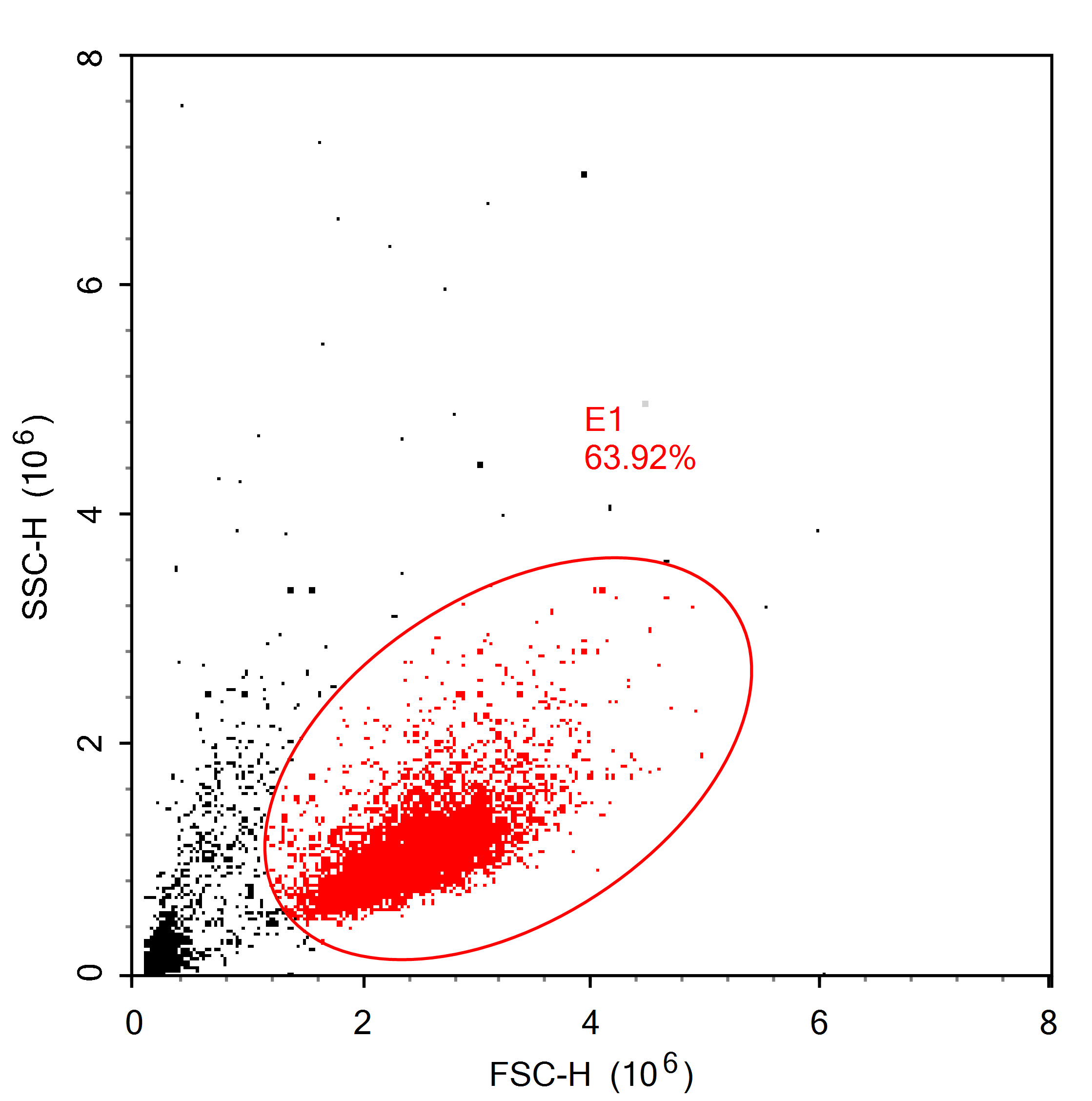

Supplement: Supplementary file 1 [file DataSheet3.zip › Flow Cytometry Assay(1,2)/Flow Cytometry Assay-2/╧╕░√╡≥═÷-2/HK-2 ╡≥═÷ 1/═╝╞1⁄4/PI/═╝1.tiff]

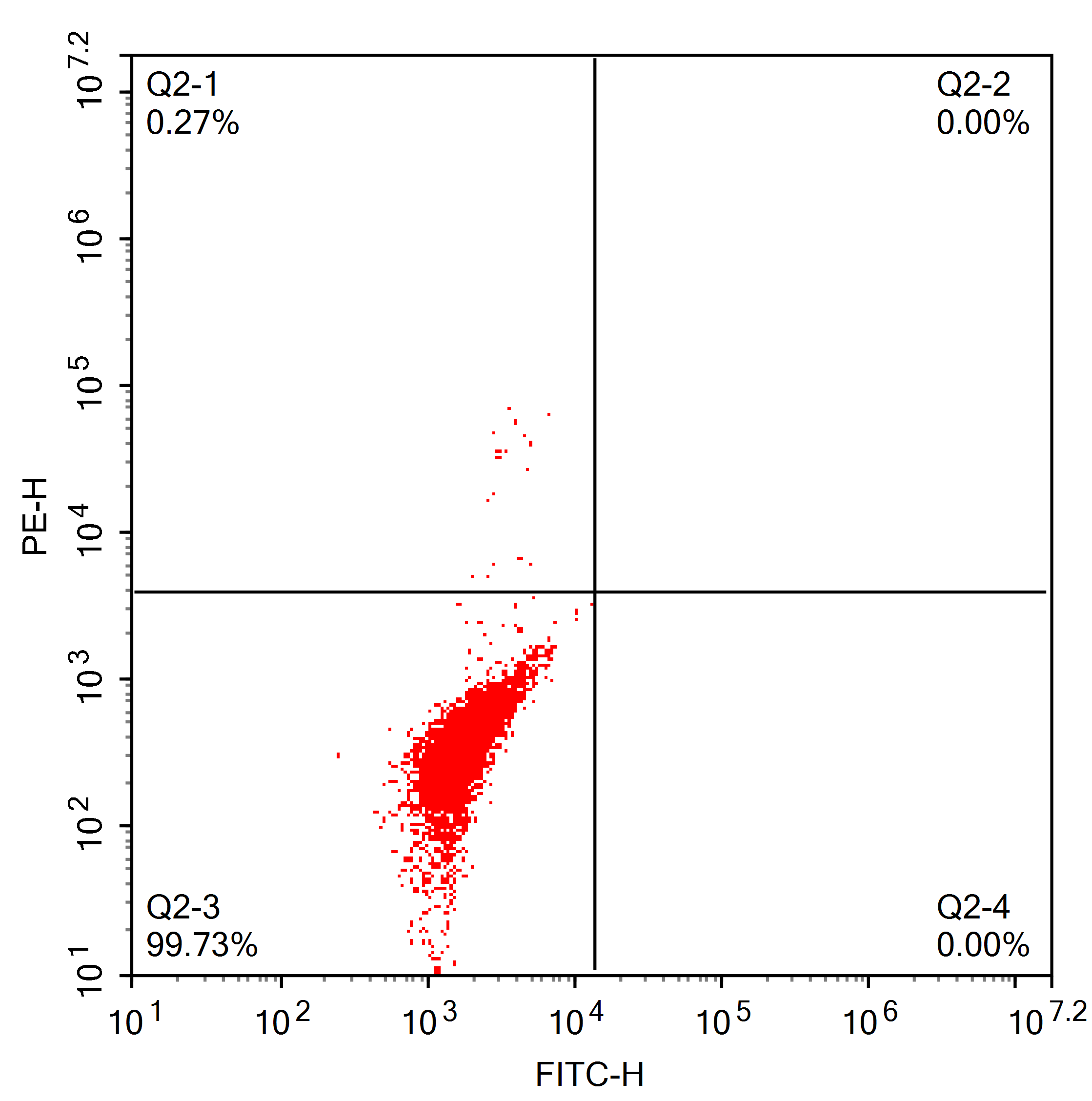

Supplement: Supplementary file 1 [file DataSheet3.zip › Flow Cytometry Assay(1,2)/Flow Cytometry Assay-2/╧╕░√╡≥═÷-2/HK-2 ╡≥═÷ 1/═╝╞1⁄4/PI/═╝2.tiff]

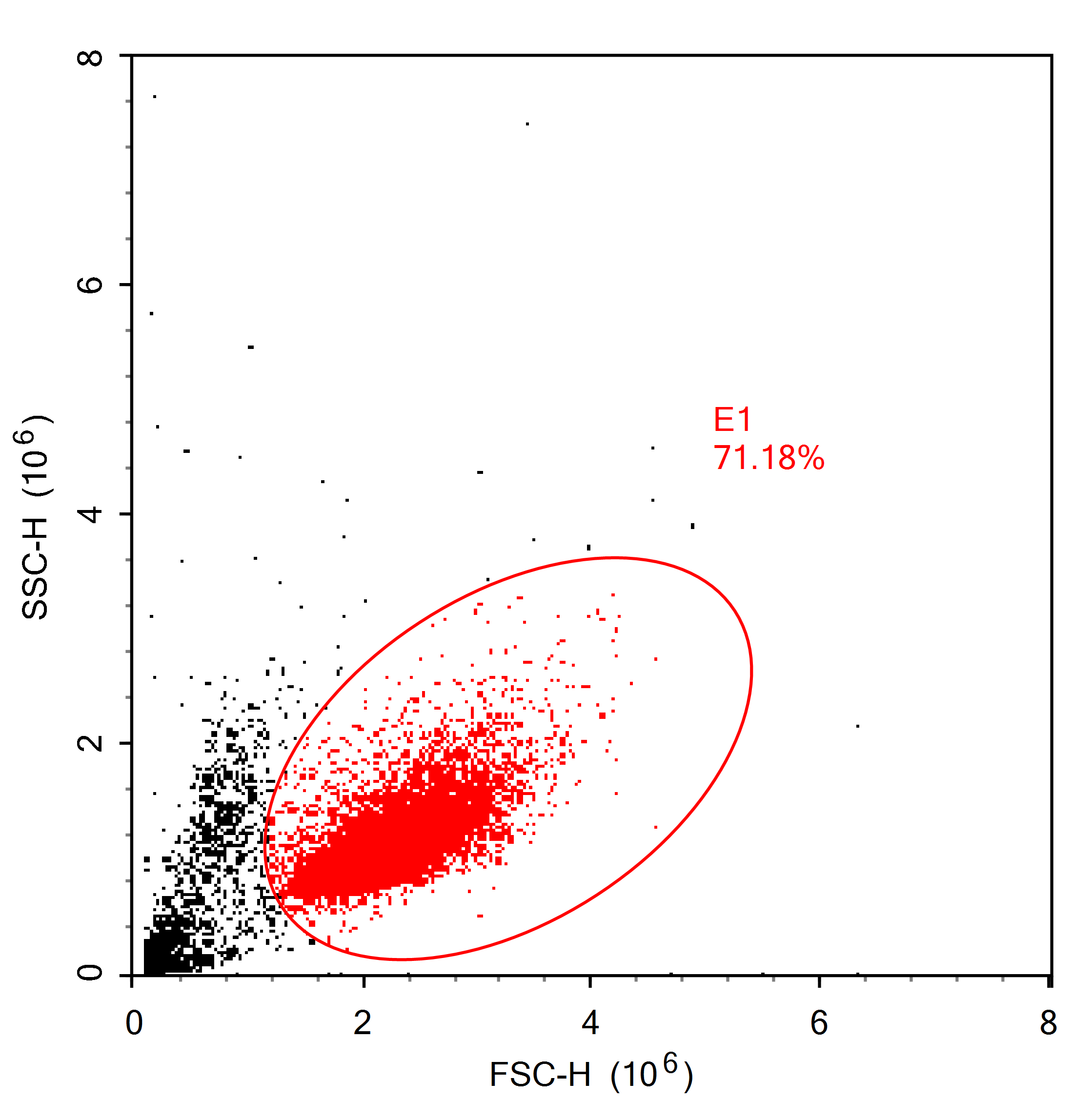

Supplement: Supplementary file 1 [file DataSheet3.zip › Flow Cytometry Assay(1,2)/Flow Cytometry Assay-2/╧╕░√╡≥═÷-2/HK-2 ╡≥═÷ 1/═╝╞1⁄4/RU360 1/═╝1.tiff]

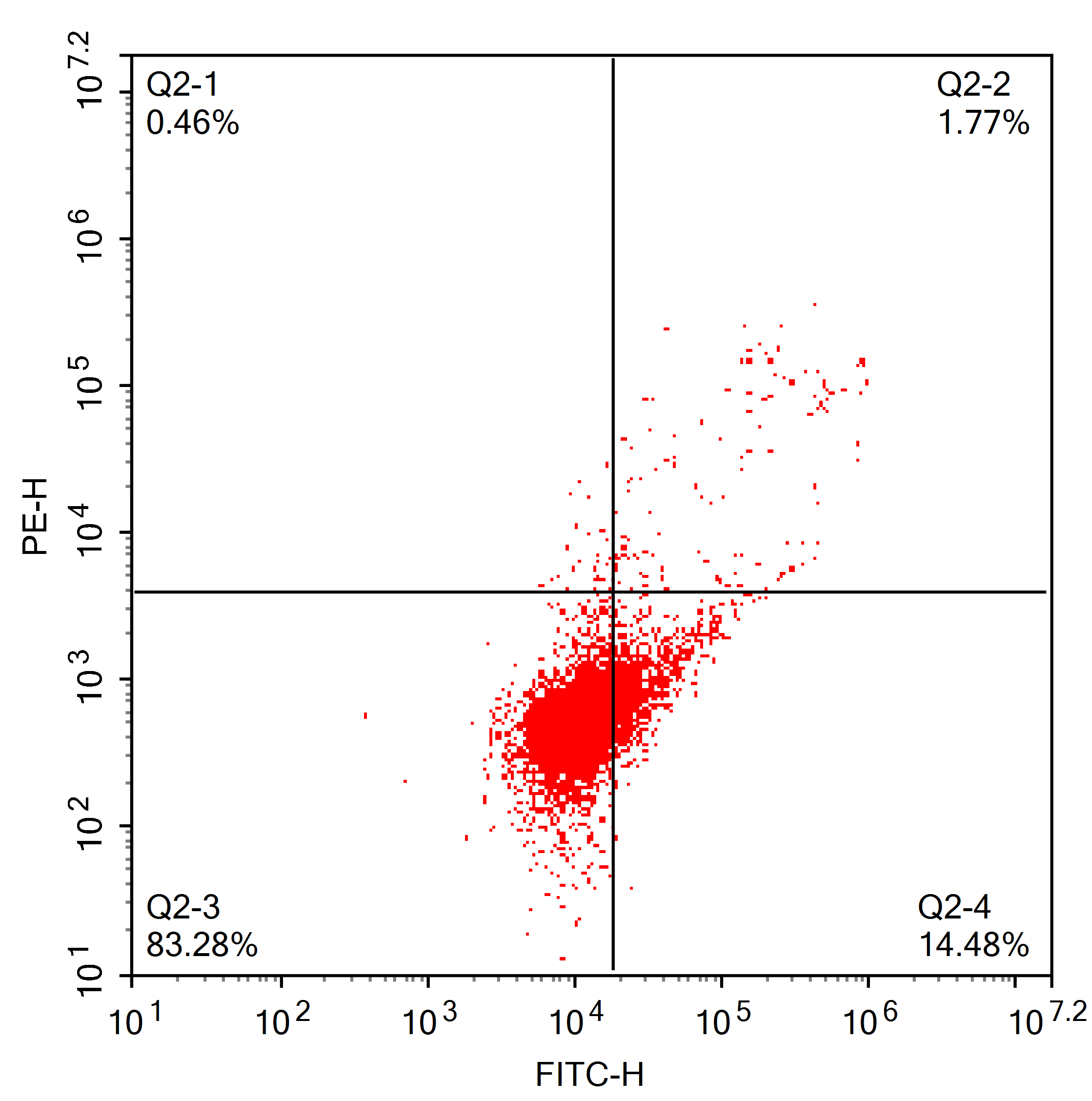

Supplement: Supplementary file 1 [file DataSheet3.zip › Flow Cytometry Assay(1,2)/Flow Cytometry Assay-2/╧╕░√╡≥═÷-2/HK-2 ╡≥═÷ 1/═╝╞1⁄4/RU360 1/═╝2.tiff]

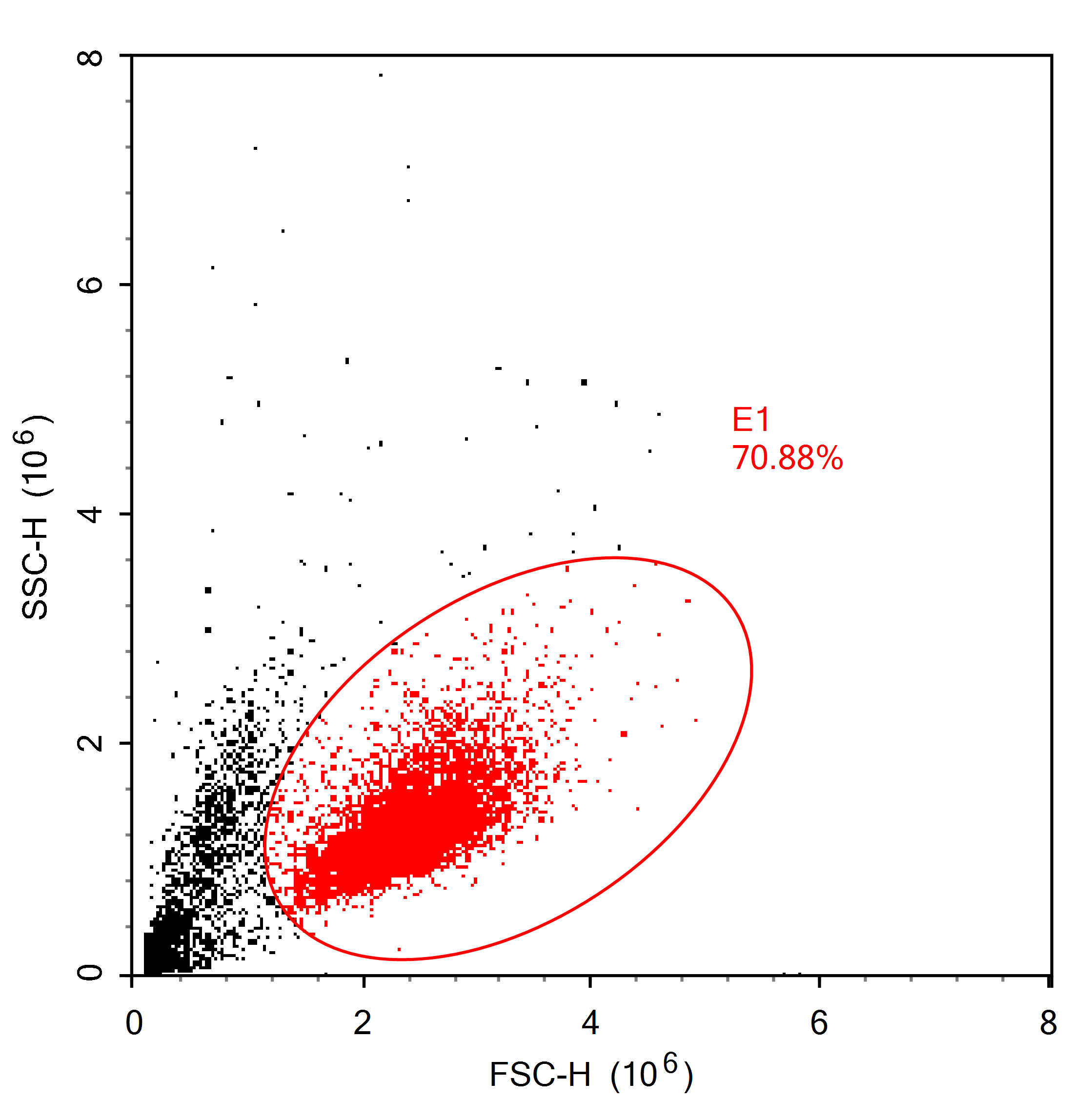

Supplement: Supplementary file 1 [file DataSheet3.zip › Flow Cytometry Assay(1,2)/Flow Cytometry Assay-2/╧╕░√╡≥═÷-2/HK-2 ╡≥═÷ 1/═╝╞1⁄4/RU360 2/═╝1.tiff]

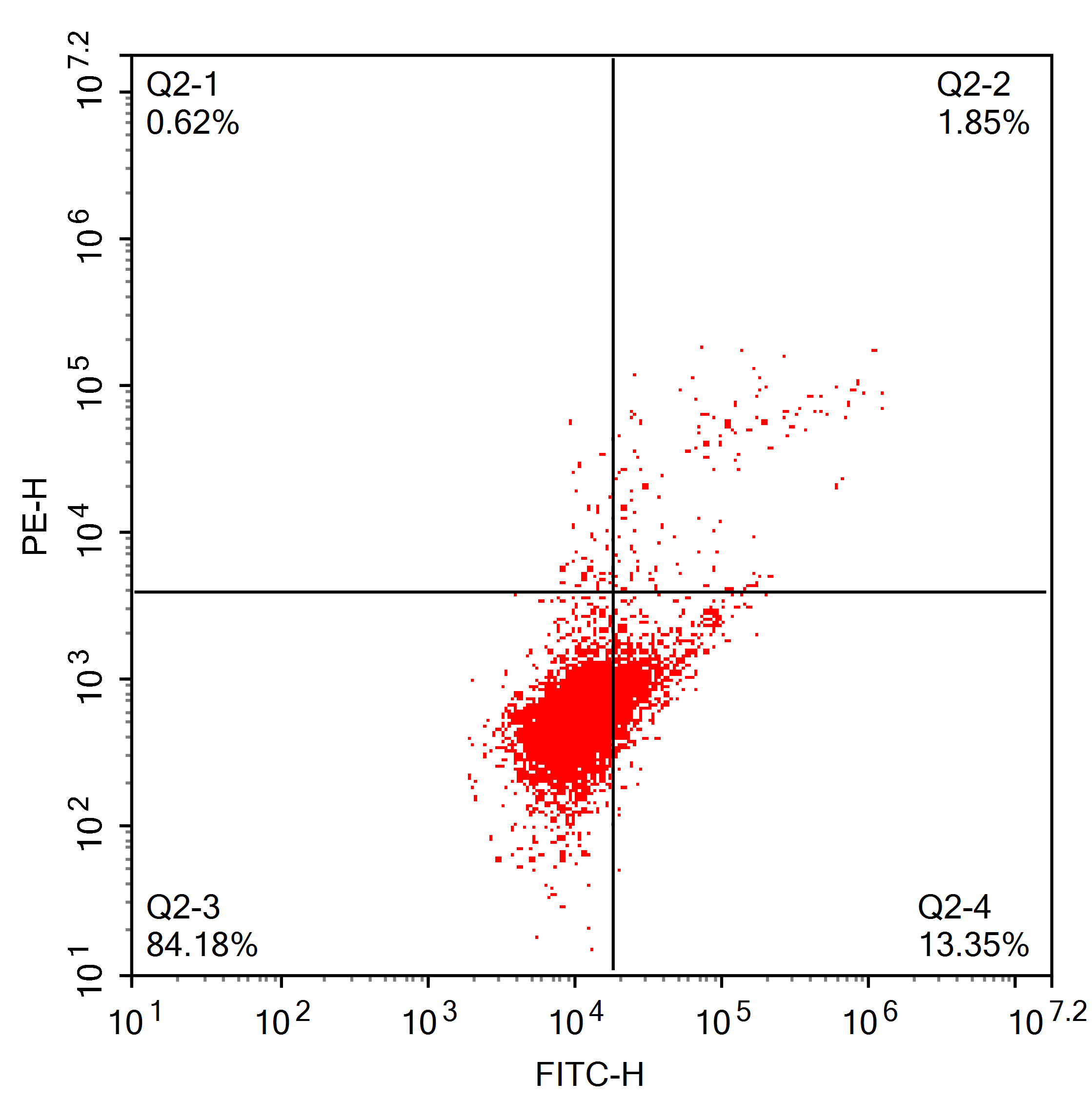

Supplement: Supplementary file 1 [file DataSheet3.zip › Flow Cytometry Assay(1,2)/Flow Cytometry Assay-2/╧╕░√╡≥═÷-2/HK-2 ╡≥═÷ 1/═╝╞1⁄4/RU360 2/═╝2.tiff]

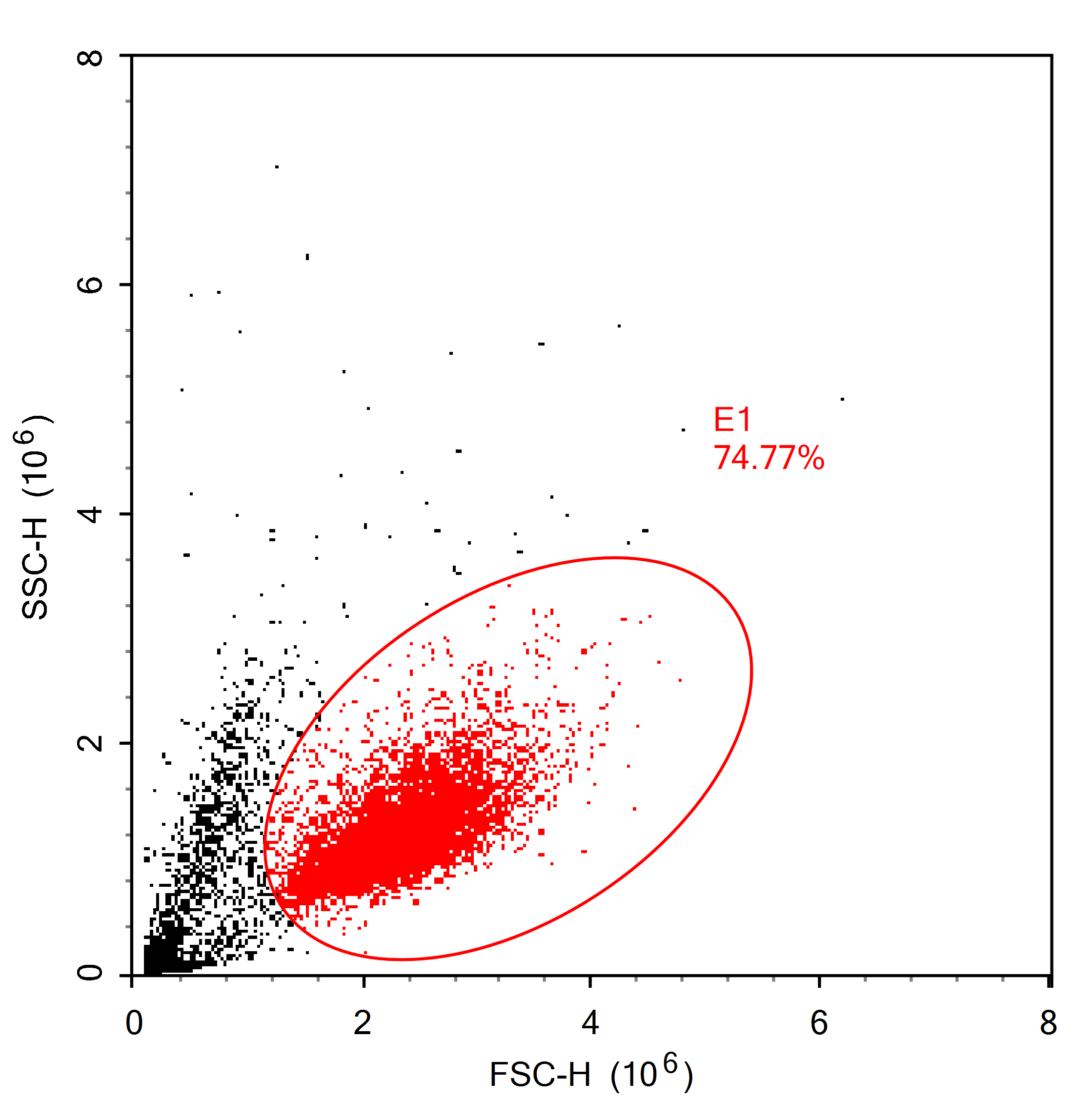

Supplement: Supplementary file 1 [file DataSheet3.zip › Flow Cytometry Assay(1,2)/Flow Cytometry Assay-2/╧╕░√╡≥═÷-2/HK-2 ╡≥═÷ 1/═╝╞1⁄4/RU360 3/═╝1.tiff]

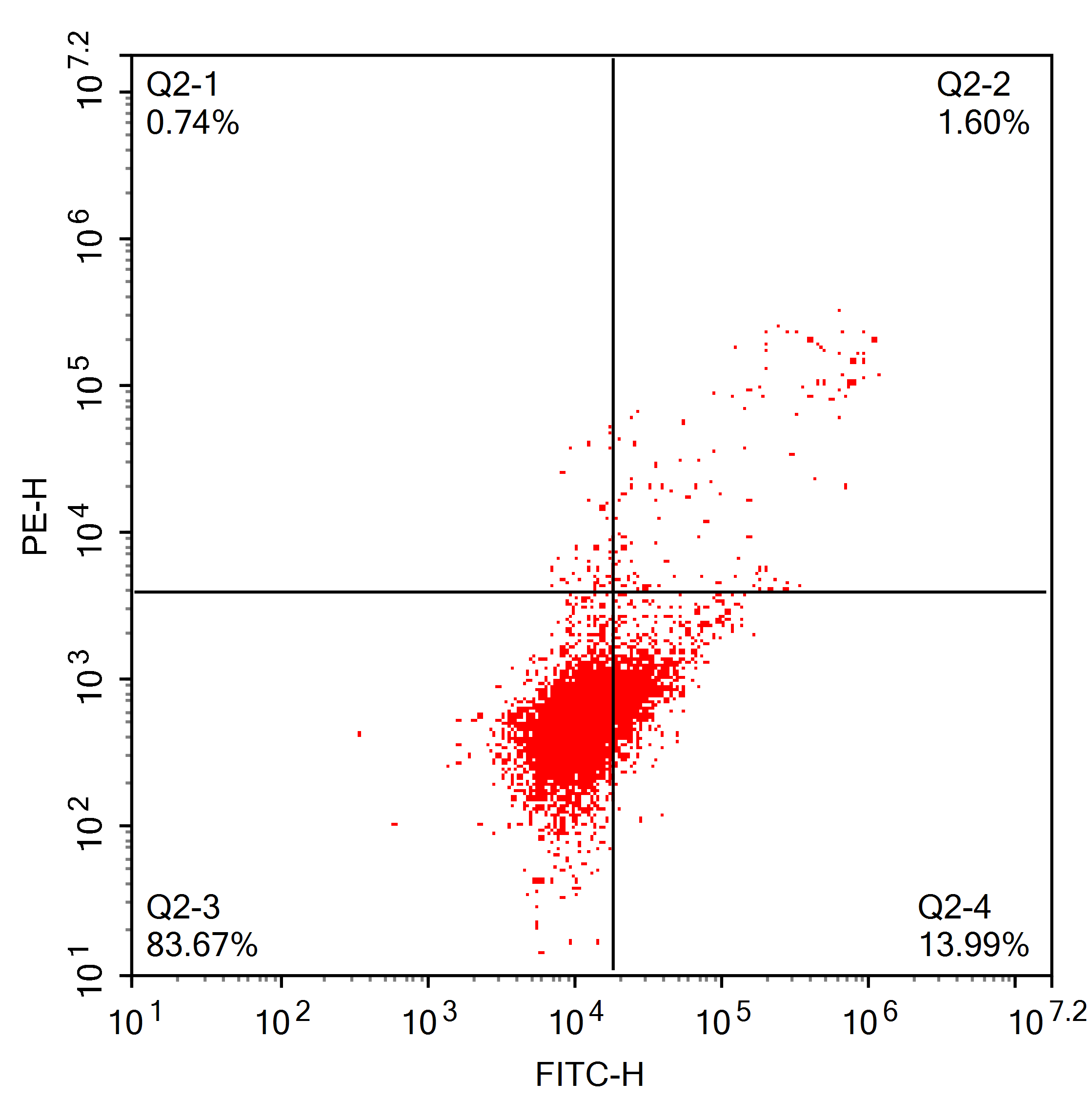

Supplement: Supplementary file 1 [file DataSheet3.zip › Flow Cytometry Assay(1,2)/Flow Cytometry Assay-2/╧╕░√╡≥═÷-2/HK-2 ╡≥═÷ 1/═╝╞1⁄4/RU360 3/═╝2.tiff]

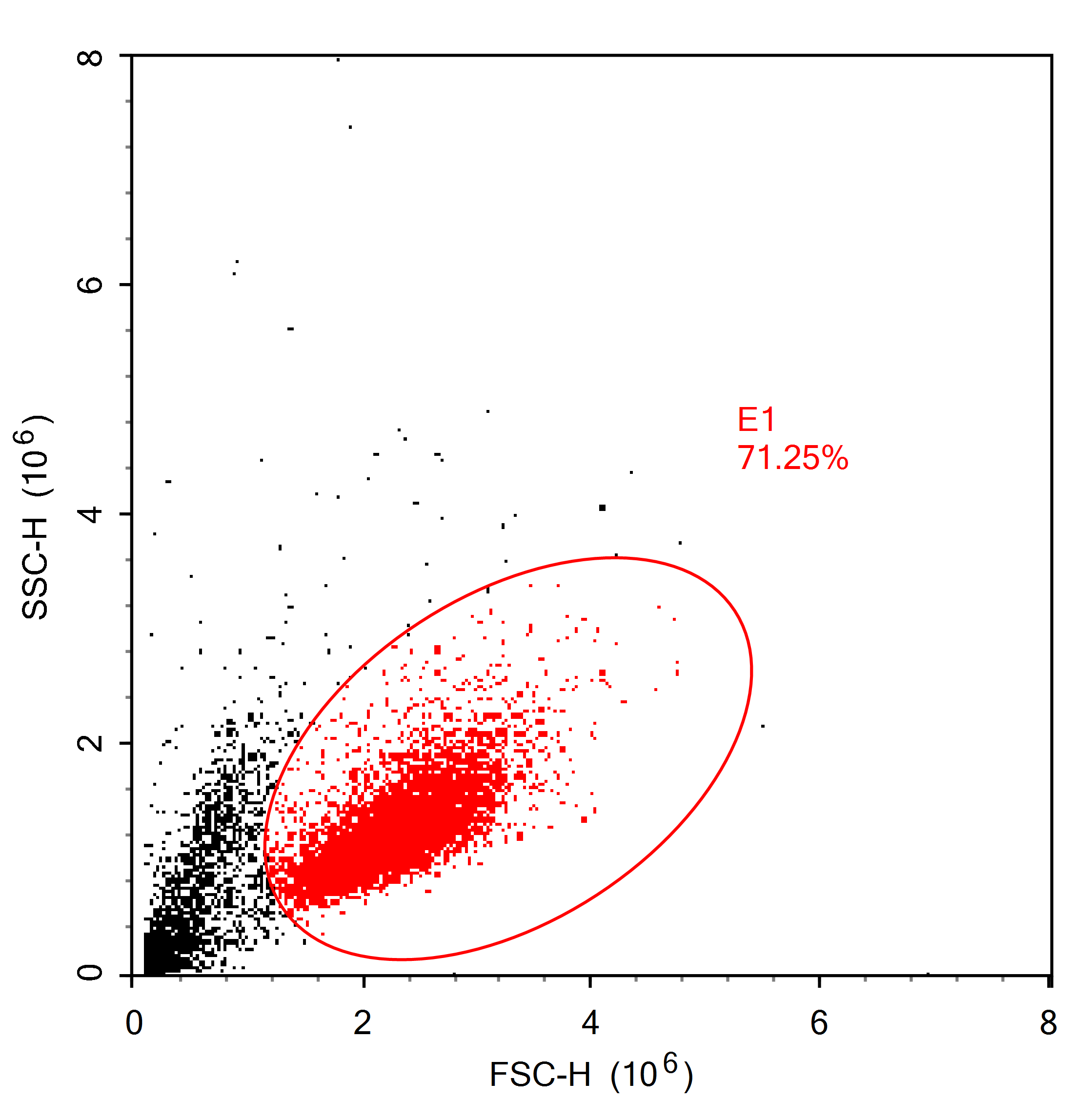

Supplement: Supplementary file 1 [file DataSheet3.zip › Flow Cytometry Assay(1,2)/Flow Cytometry Assay-2/╧╕░√╡≥═÷-2/HK-2 ╡≥═÷ 1/═╝╞1⁄4/Spermine 1/═╝1.tiff]

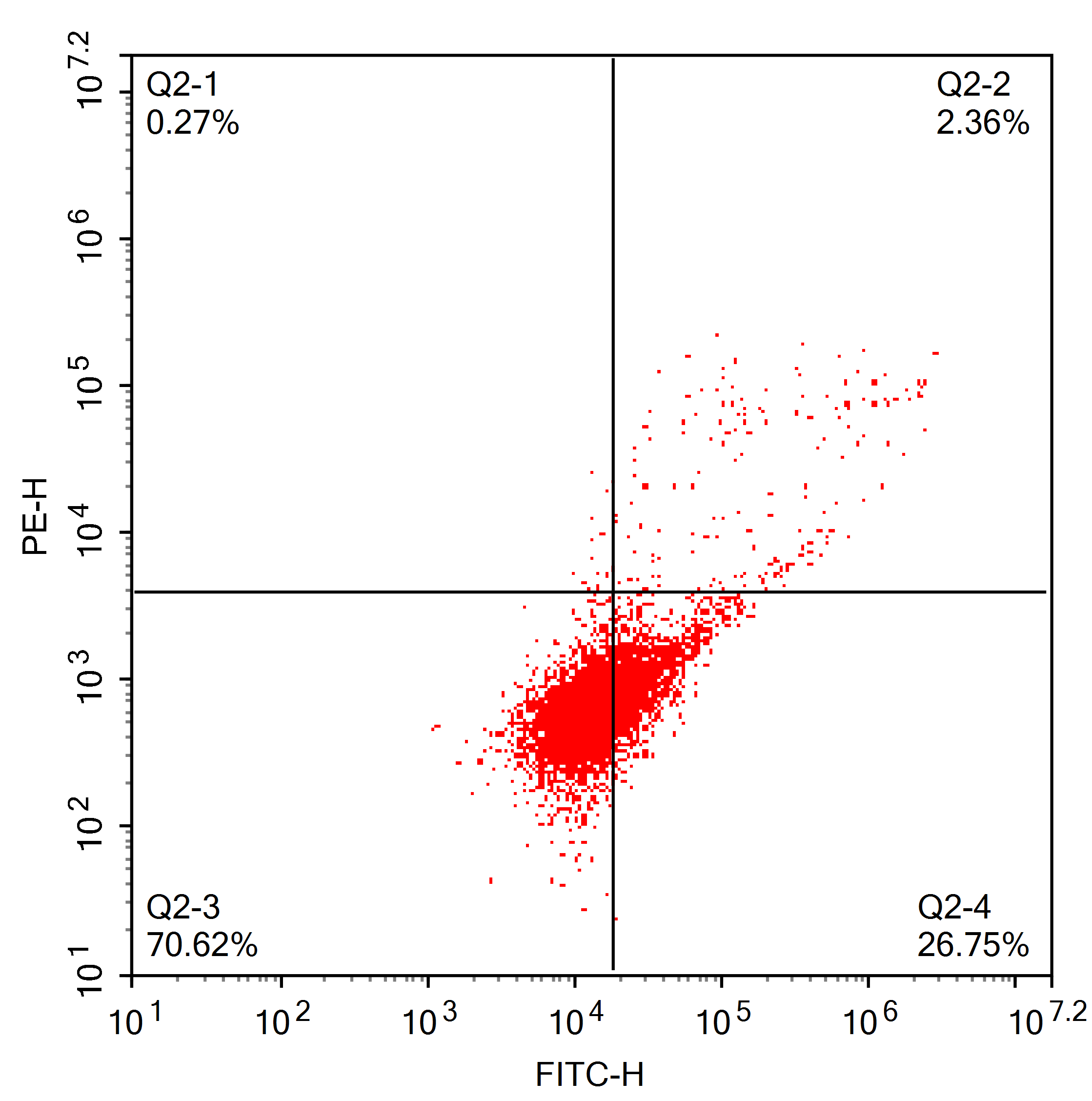

Supplement: Supplementary file 1 [file DataSheet3.zip › Flow Cytometry Assay(1,2)/Flow Cytometry Assay-2/╧╕░√╡≥═÷-2/HK-2 ╡≥═÷ 1/═╝╞1⁄4/Spermine 1/═╝2.tiff]

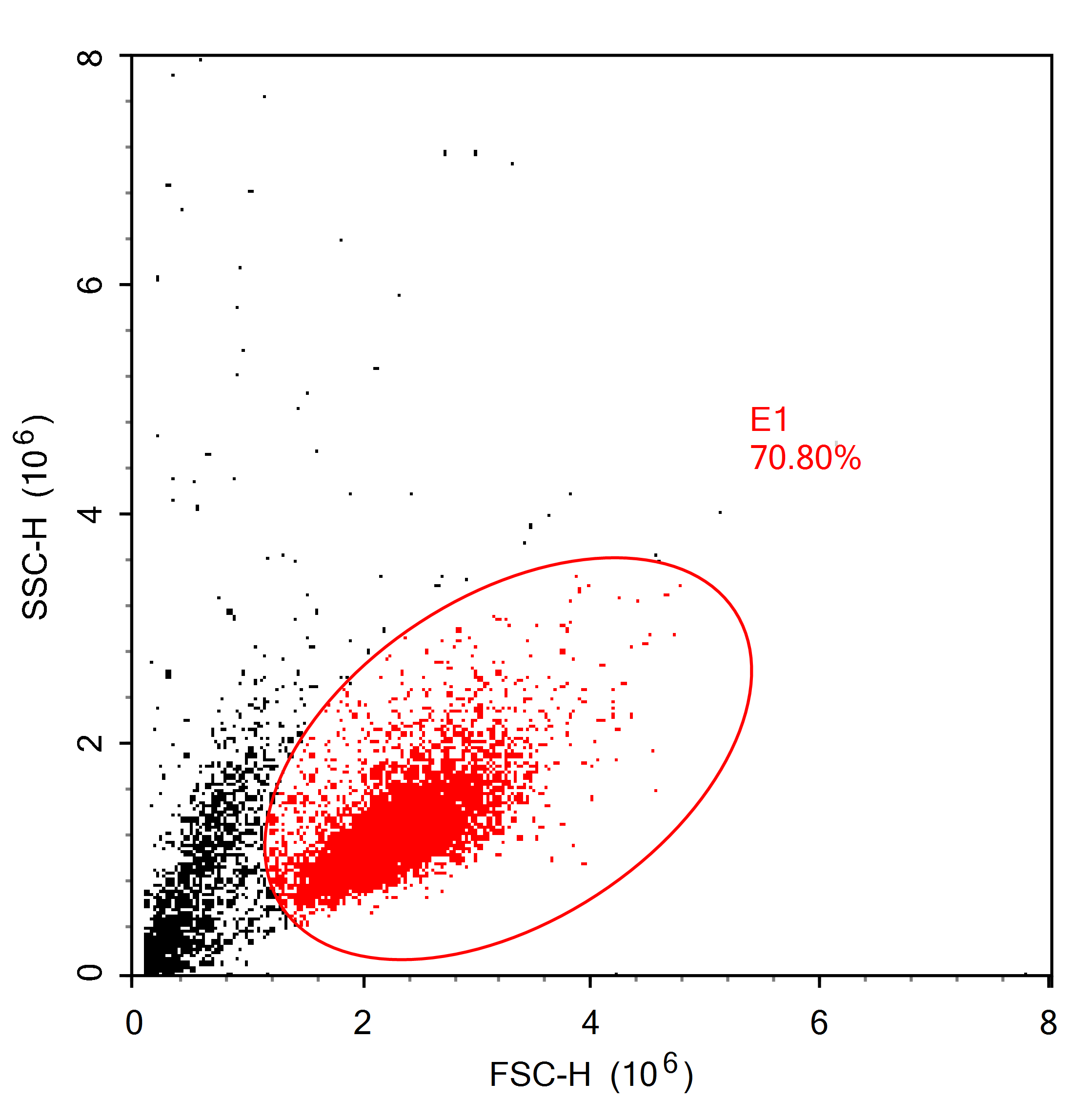

Supplement: Supplementary file 1 [file DataSheet3.zip › Flow Cytometry Assay(1,2)/Flow Cytometry Assay-2/╧╕░√╡≥═÷-2/HK-2 ╡≥═÷ 1/═╝╞1⁄4/SperSpermine 2/═╝1.tiff]

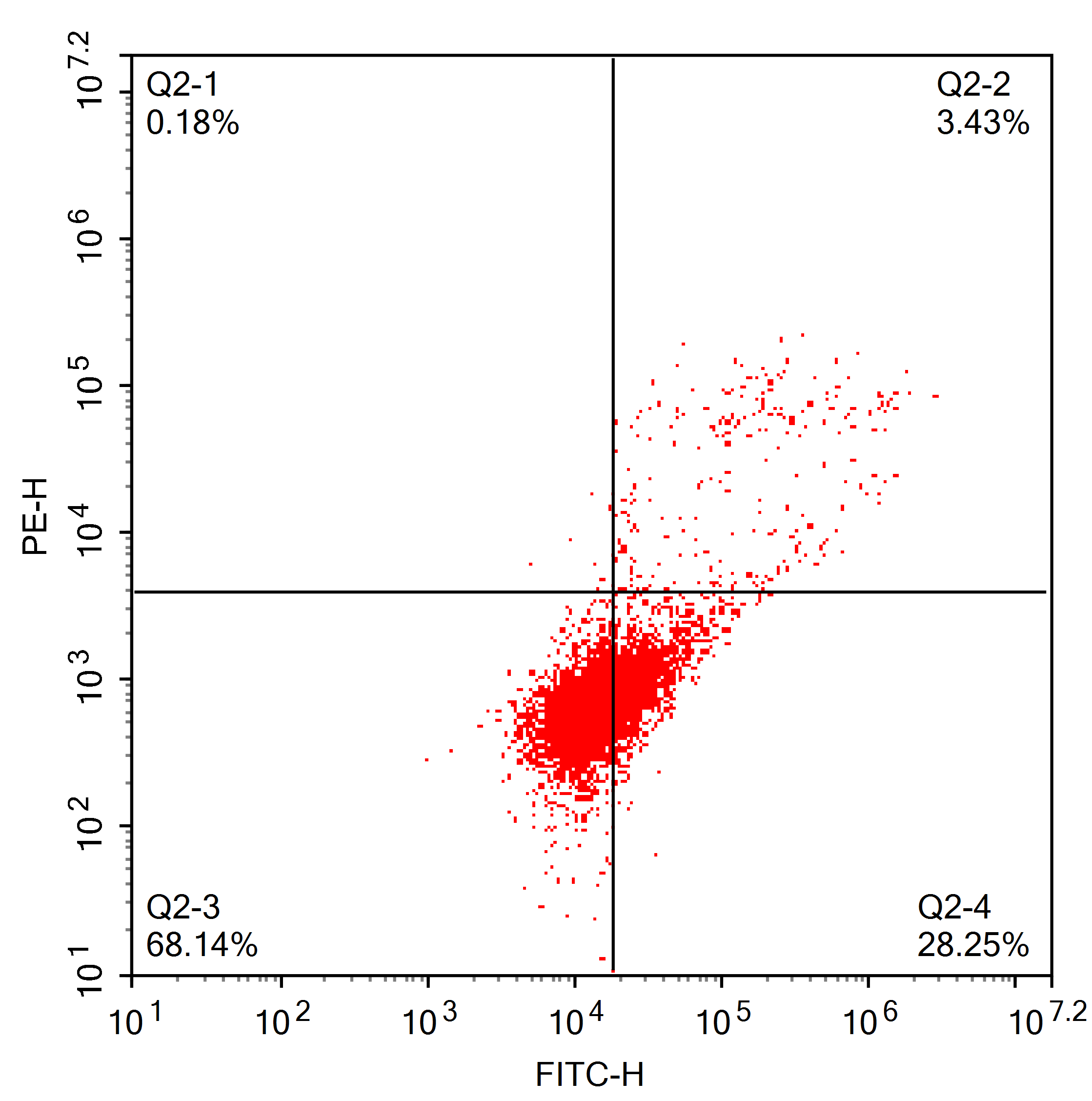

Supplement: Supplementary file 1 [file DataSheet3.zip › Flow Cytometry Assay(1,2)/Flow Cytometry Assay-2/╧╕░√╡≥═÷-2/HK-2 ╡≥═÷ 1/═╝╞1⁄4/SperSpermine 2/═╝2.tiff]

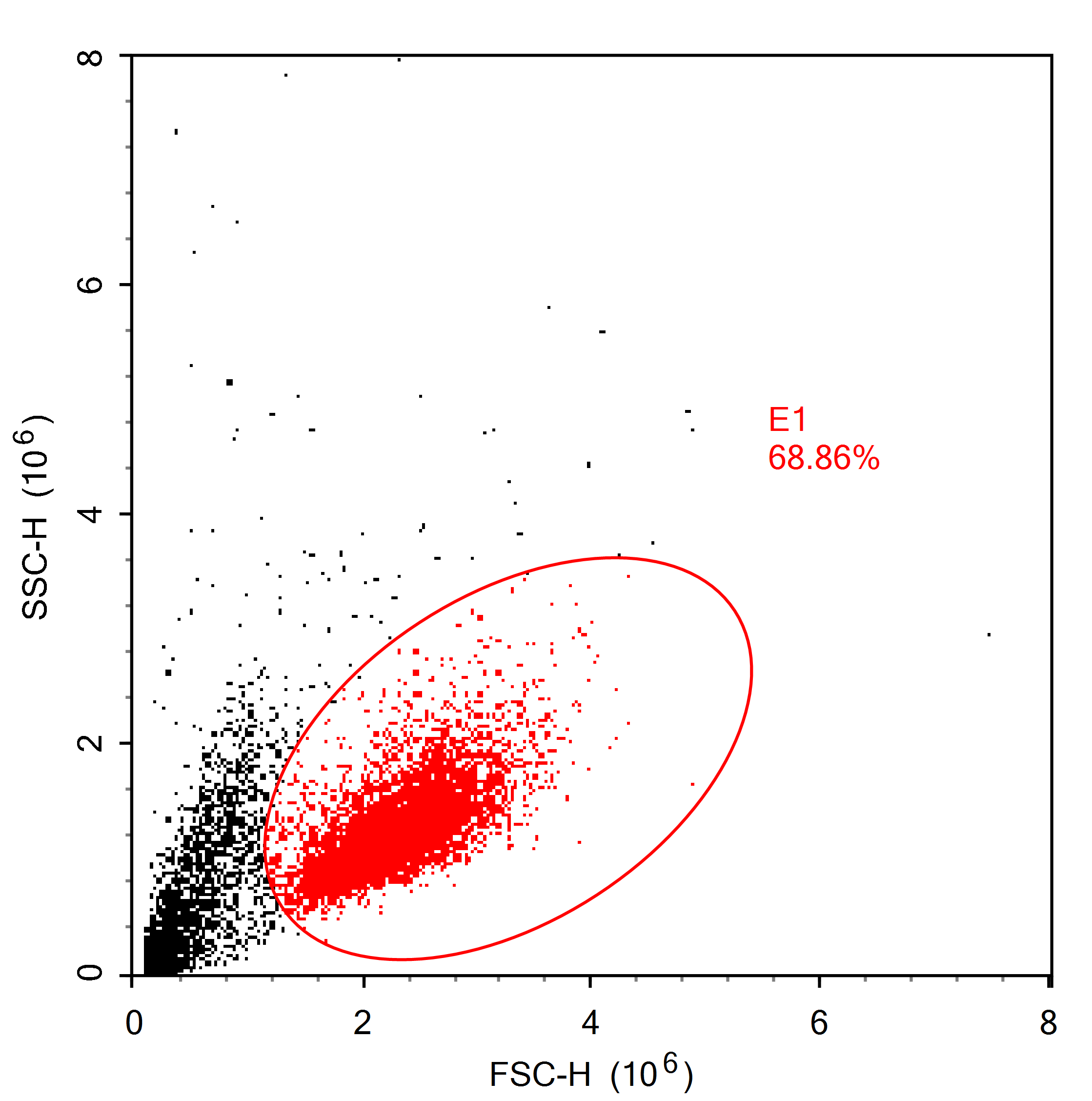

Supplement: Supplementary file 1 [file DataSheet3.zip › Flow Cytometry Assay(1,2)/Flow Cytometry Assay-2/╧╕░√╡≥═÷-2/HK-2 ╡≥═÷ 1/═╝╞1⁄4/SperSpermine 3/═╝1.tiff]

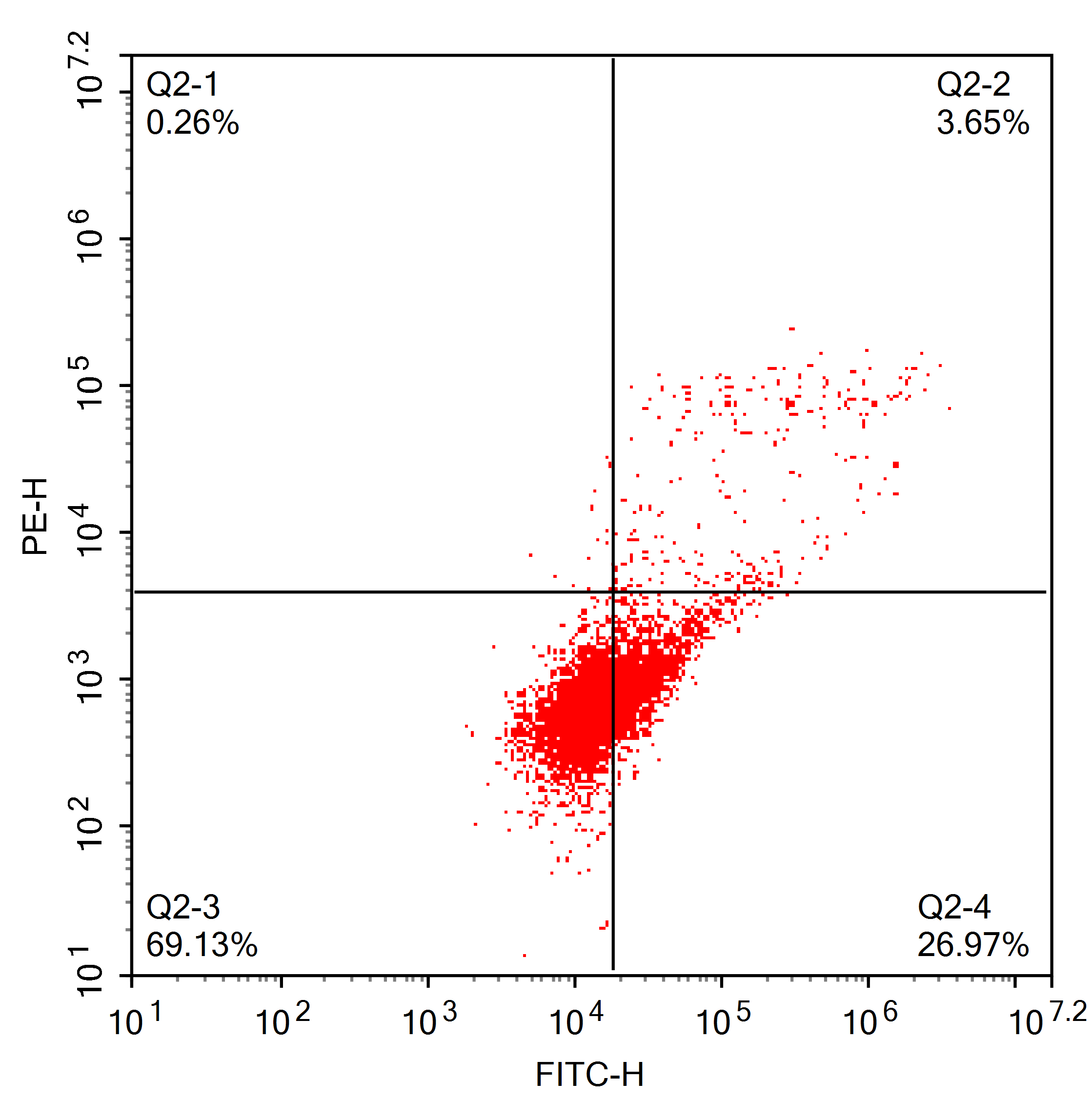

Supplement: Supplementary file 1 [file DataSheet3.zip › Flow Cytometry Assay(1,2)/Flow Cytometry Assay-2/╧╕░√╡≥═÷-2/HK-2 ╡≥═÷ 1/═╝╞1⁄4/SperSpermine 3/═╝2.tiff]

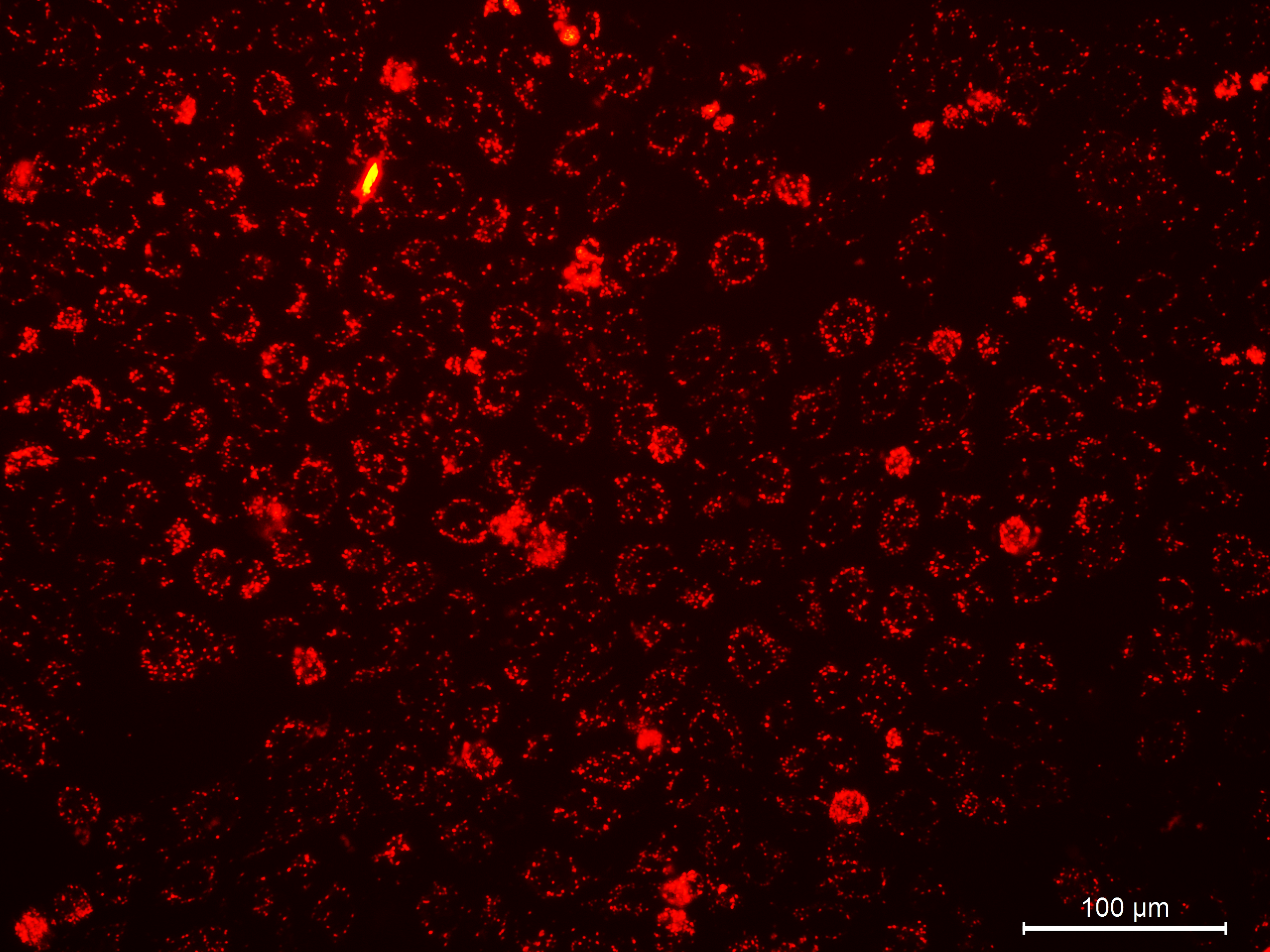

Supplement: Supplementary file 2 [file DataSheet8.zip › JC-1╢¿┴┐2/JC-1-2═╝╞1⁄4/Control 1.tif]

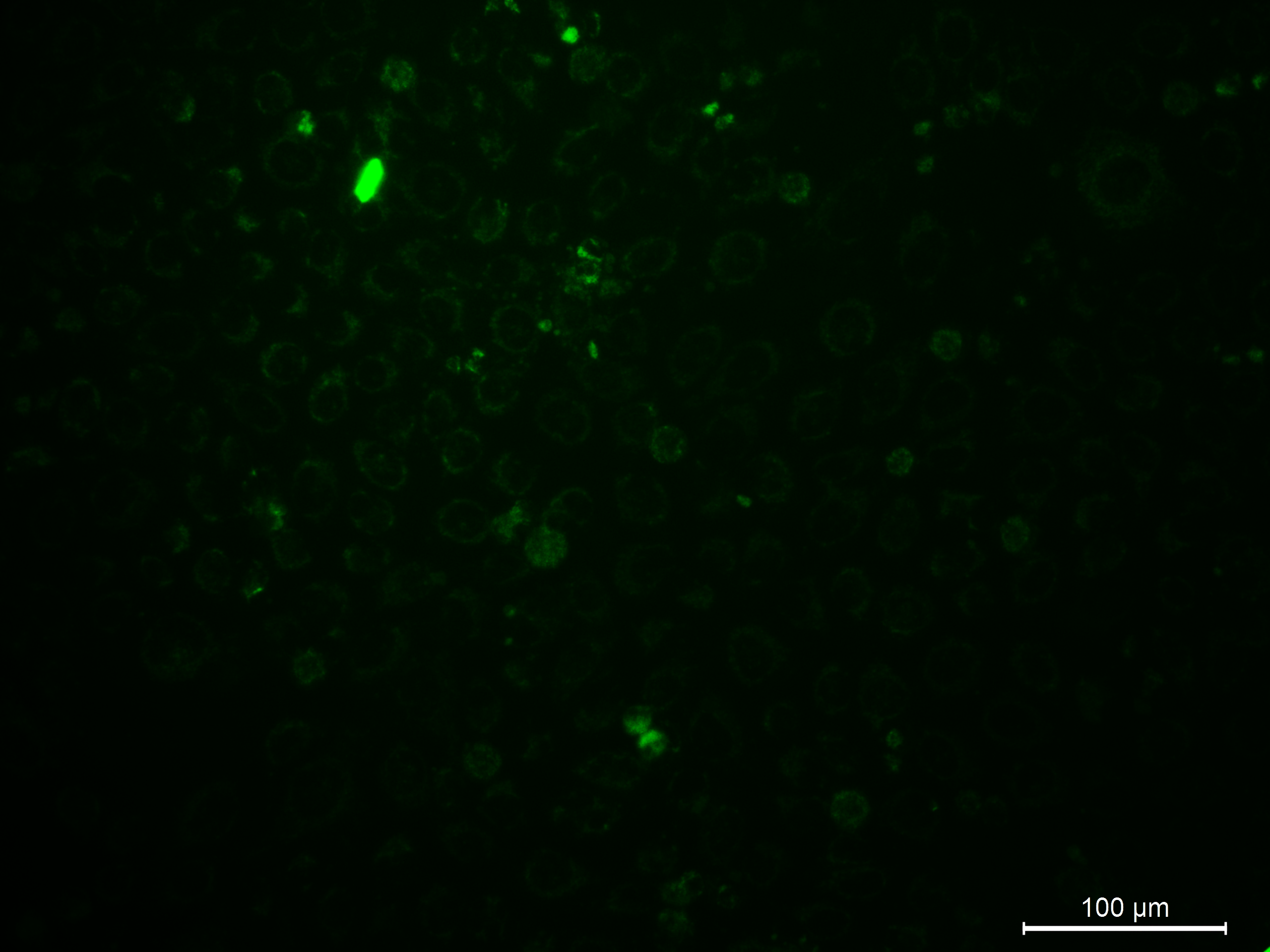

Supplement: Supplementary file 2 [file DataSheet8.zip › JC-1╢¿┴┐2/JC-1-2═╝╞1⁄4/Control 1-1.tif]

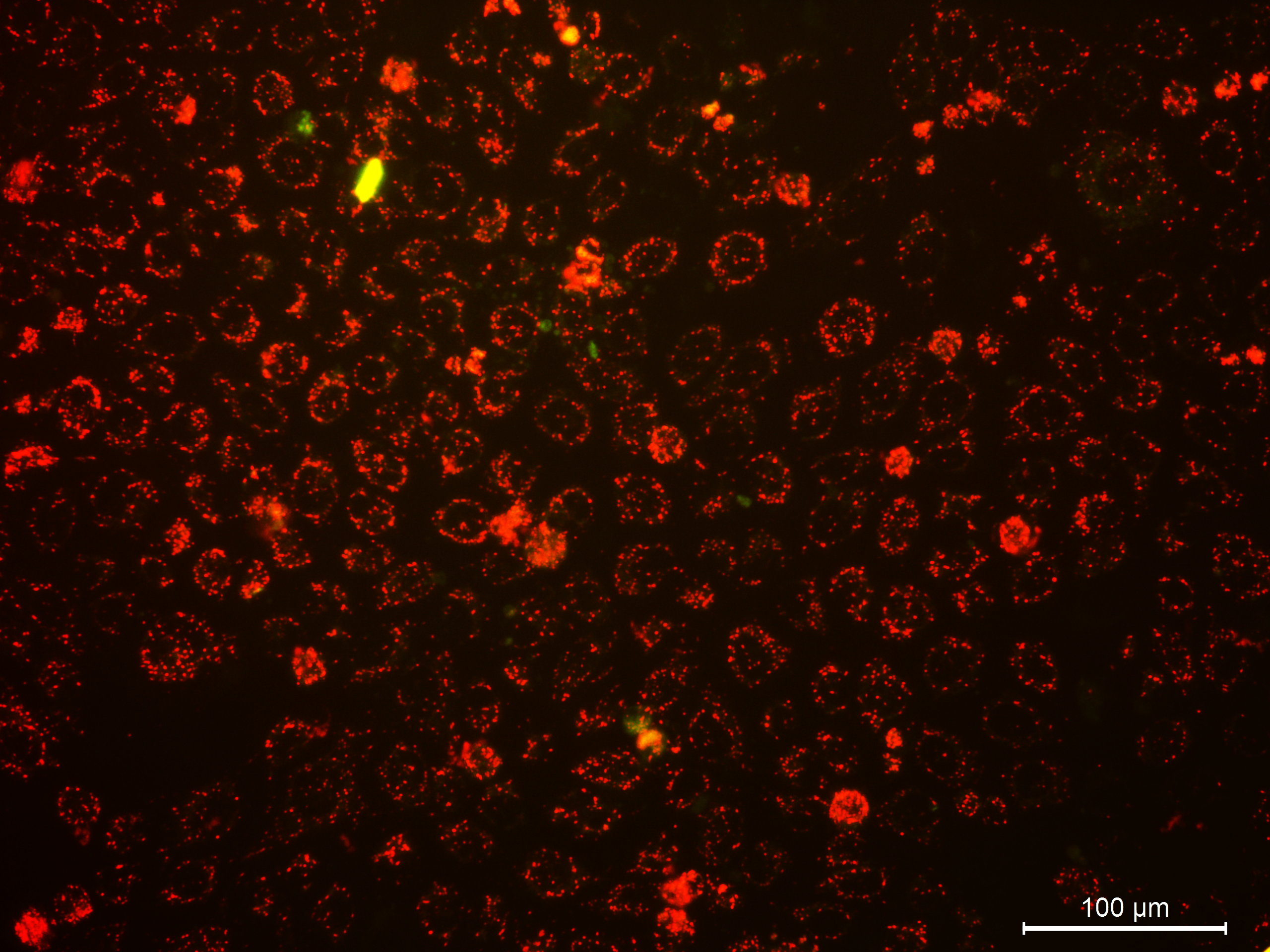

Supplement: Supplementary file 2 [file DataSheet8.zip › JC-1╢¿┴┐2/JC-1-2═╝╞1⁄4/Control 1║╧.tif]

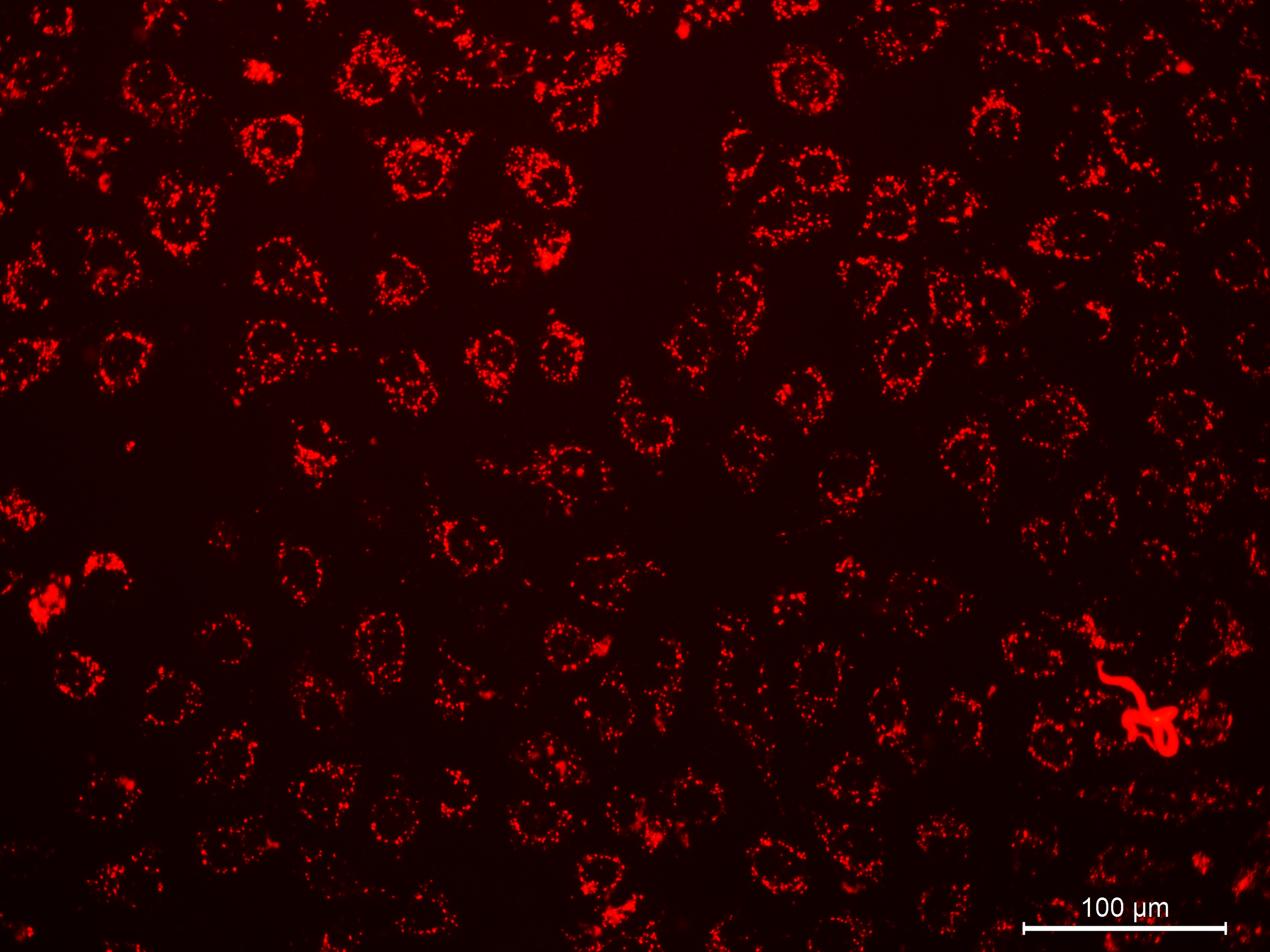

Supplement: Supplementary file 2 [file DataSheet8.zip › JC-1╢¿┴┐2/JC-1-2═╝╞1⁄4/Control 2.tif]

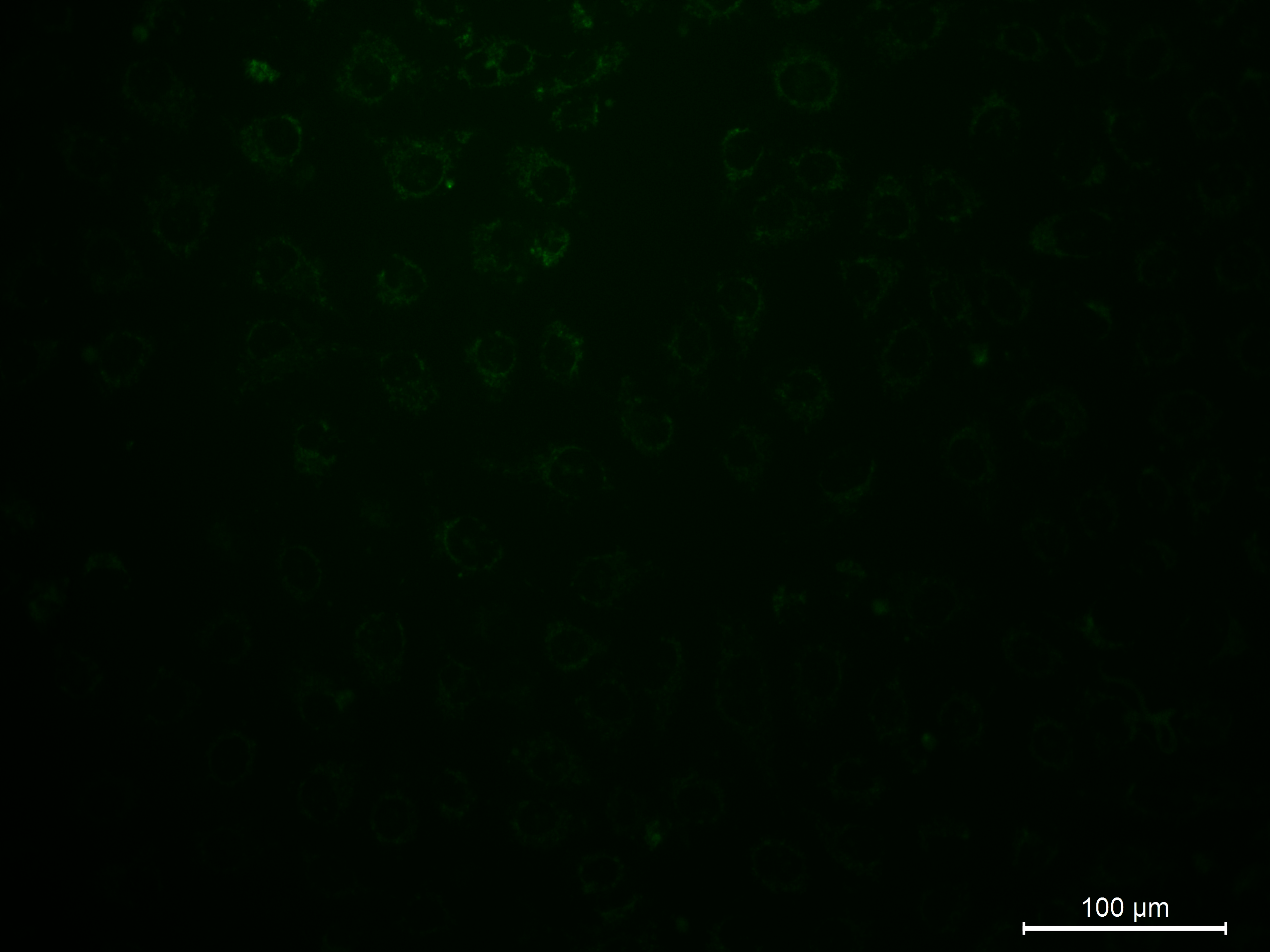

Supplement: Supplementary file 2 [file DataSheet8.zip › JC-1╢¿┴┐2/JC-1-2═╝╞1⁄4/Control 2-1.tif]

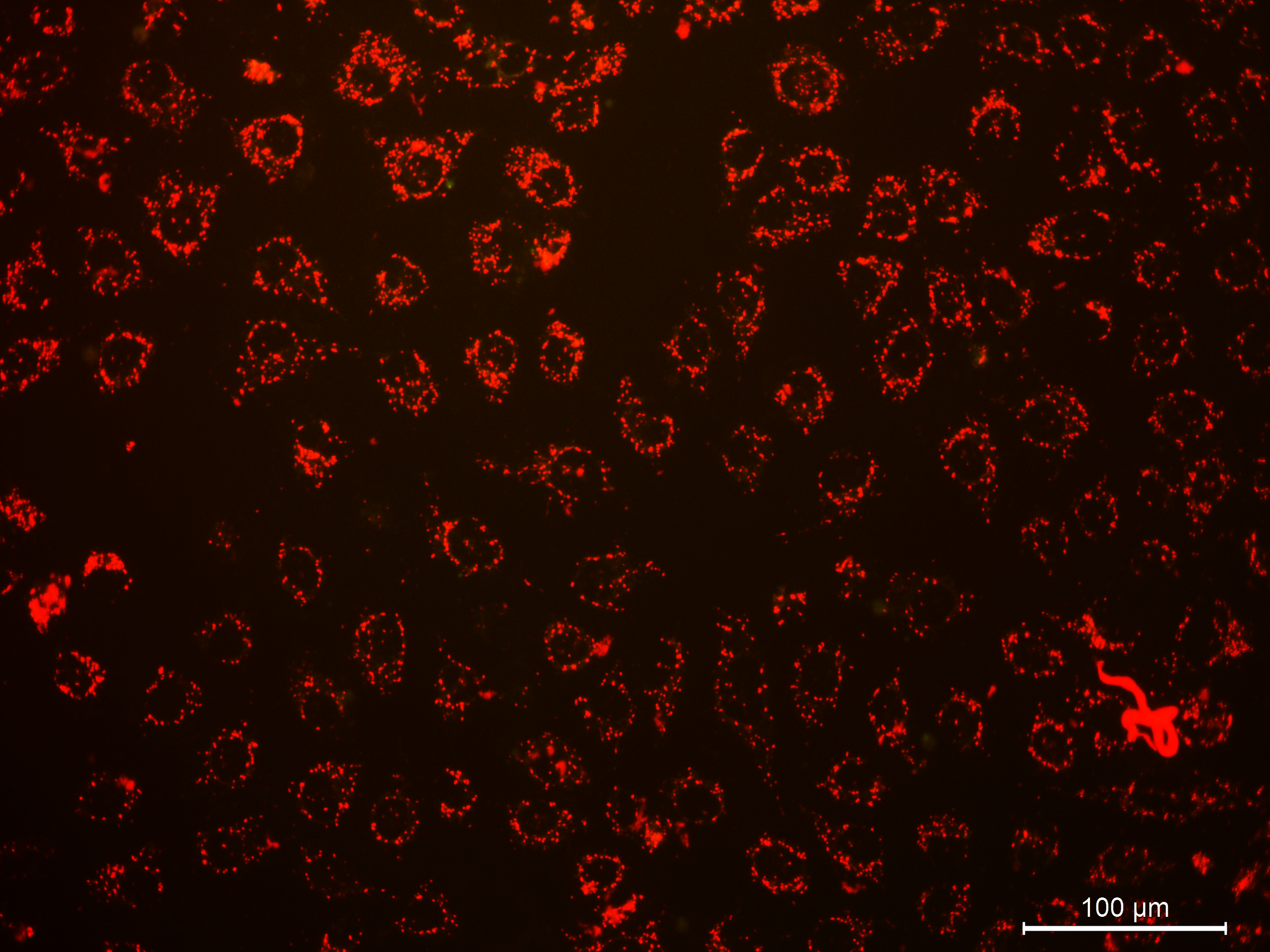

Supplement: Supplementary file 2 [file DataSheet8.zip › JC-1╢¿┴┐2/JC-1-2═╝╞1⁄4/Control 2║╧.tif]

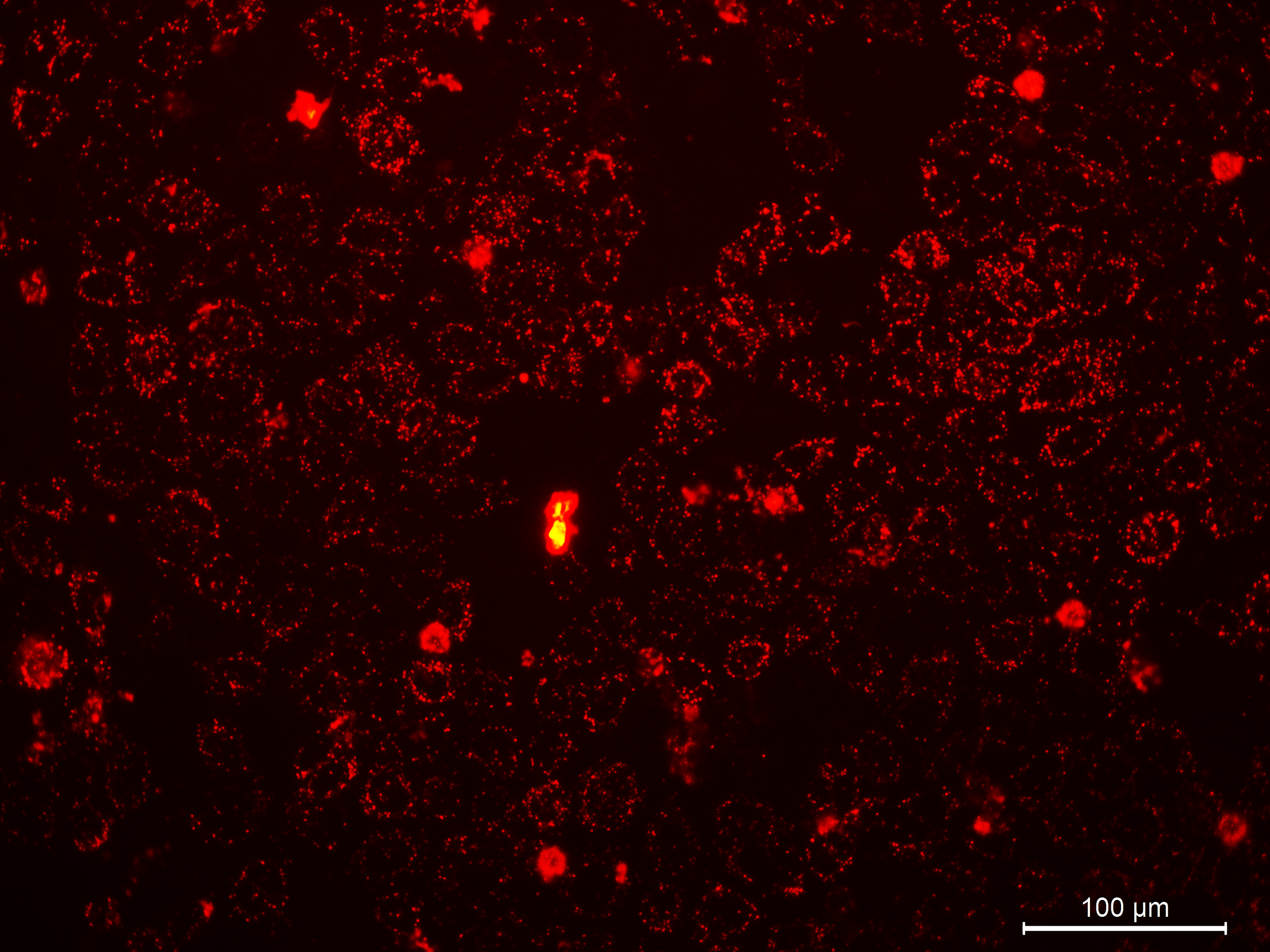

Supplement: Supplementary file 2 [file DataSheet8.zip › JC-1╢¿┴┐2/JC-1-2═╝╞1⁄4/Control 3.tif]

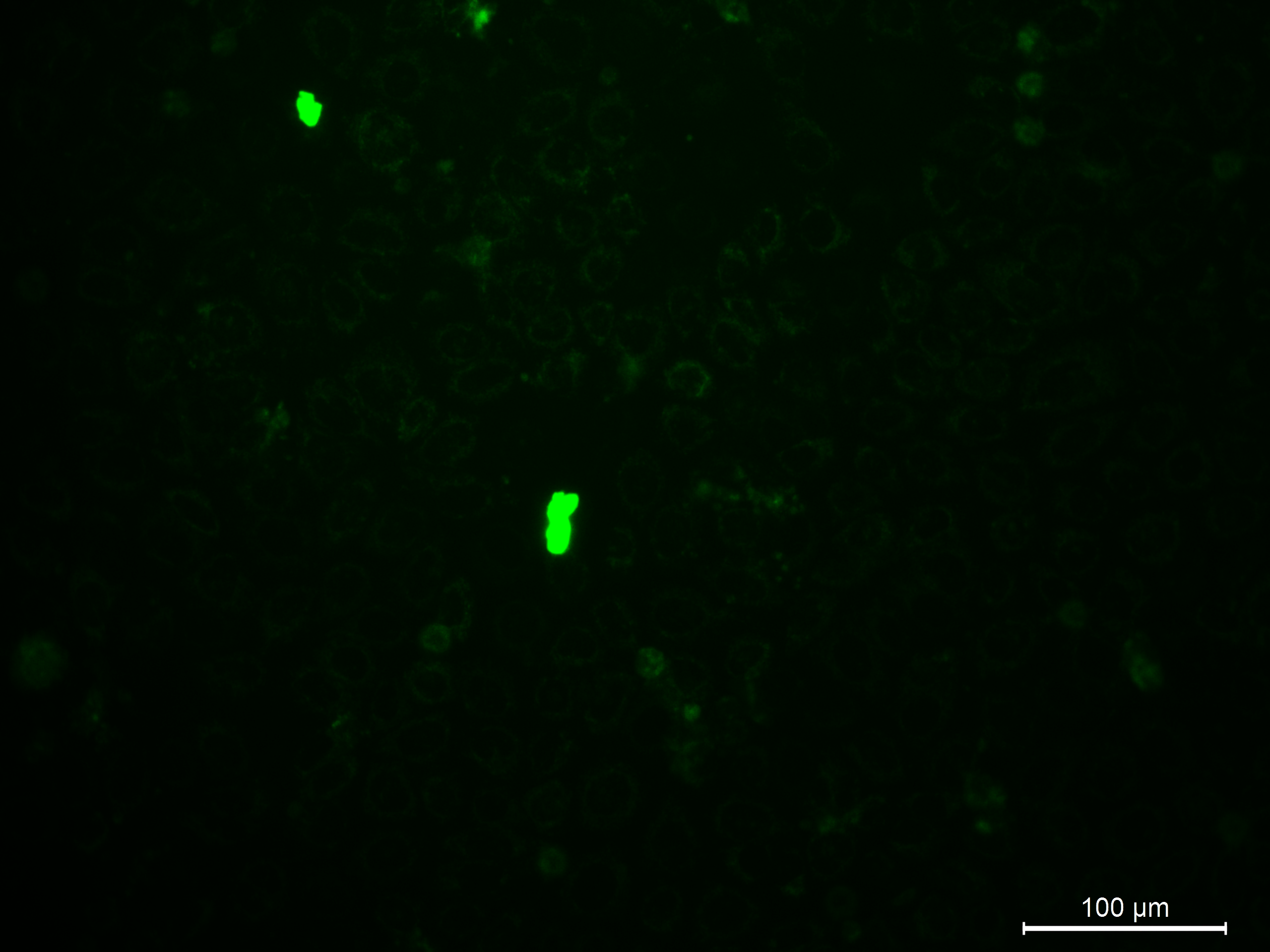

Supplement: Supplementary file 2 [file DataSheet8.zip › JC-1╢¿┴┐2/JC-1-2═╝╞1⁄4/Control 3-1.tif]

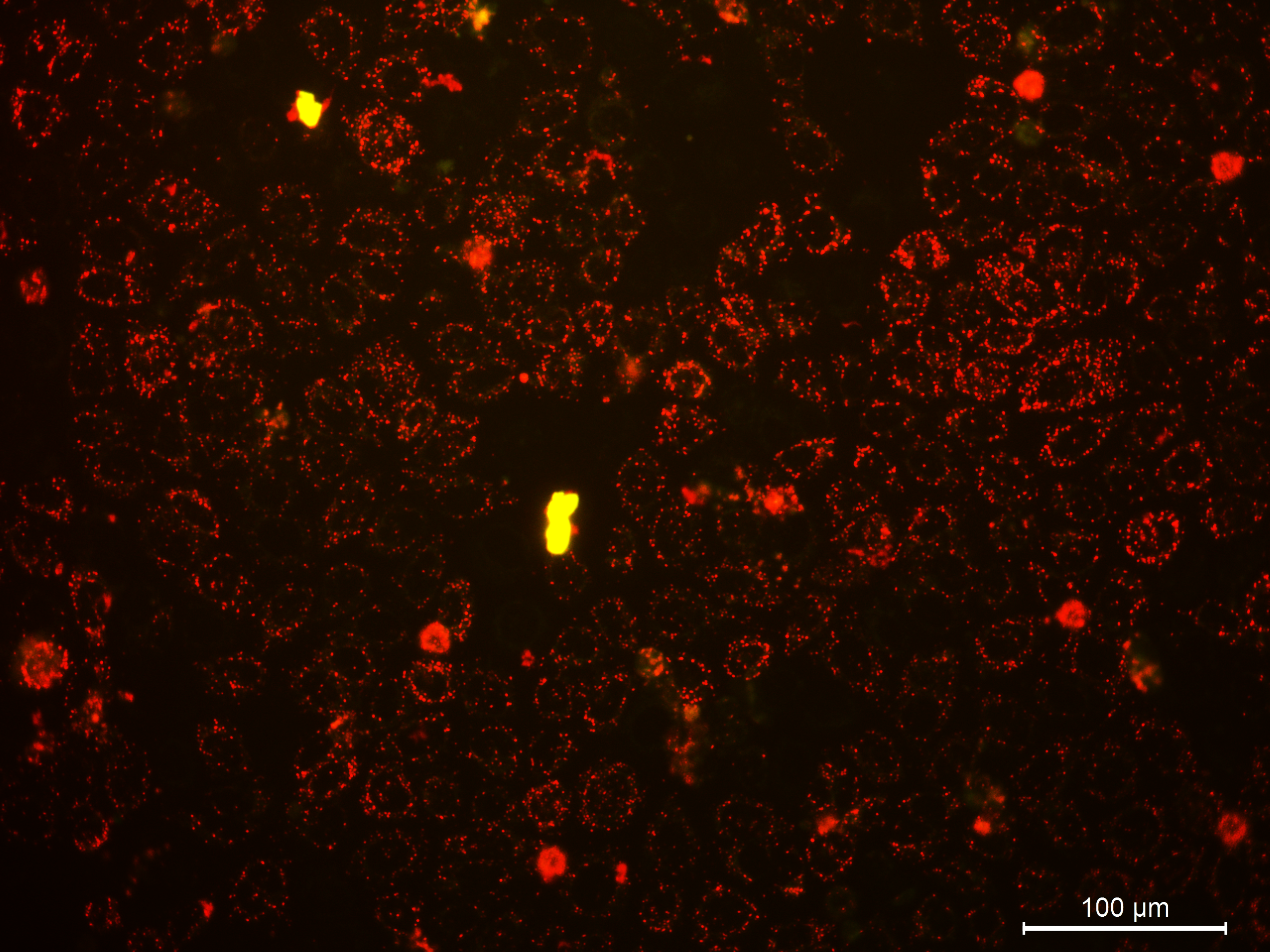

Supplement: Supplementary file 2 [file DataSheet8.zip › JC-1╢¿┴┐2/JC-1-2═╝╞1⁄4/Control 3║╧.tif]

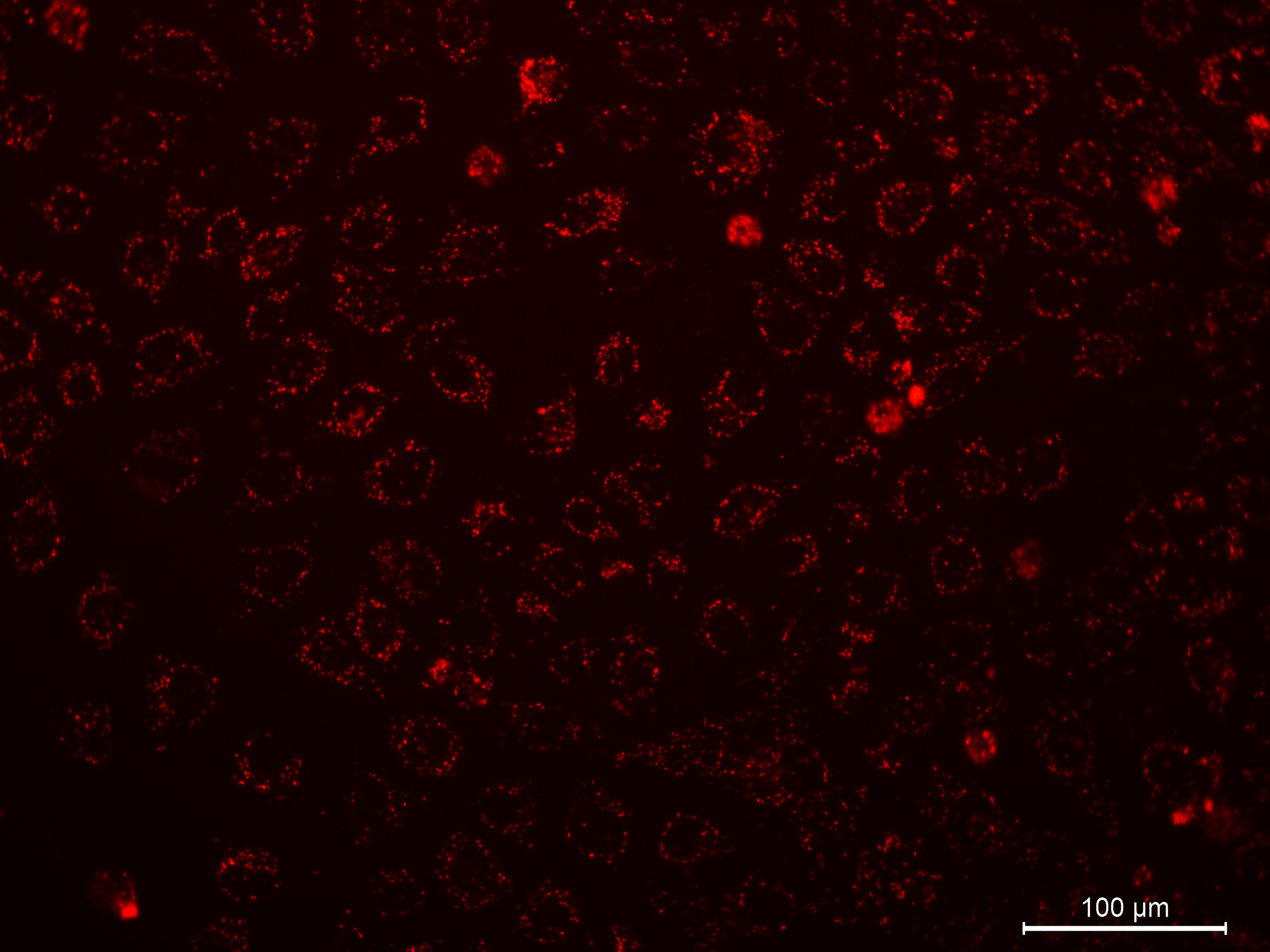

Supplement: Supplementary file 2 [file DataSheet8.zip › JC-1╢¿┴┐2/JC-1-2═╝╞1⁄4/Iohexol 1.tif]

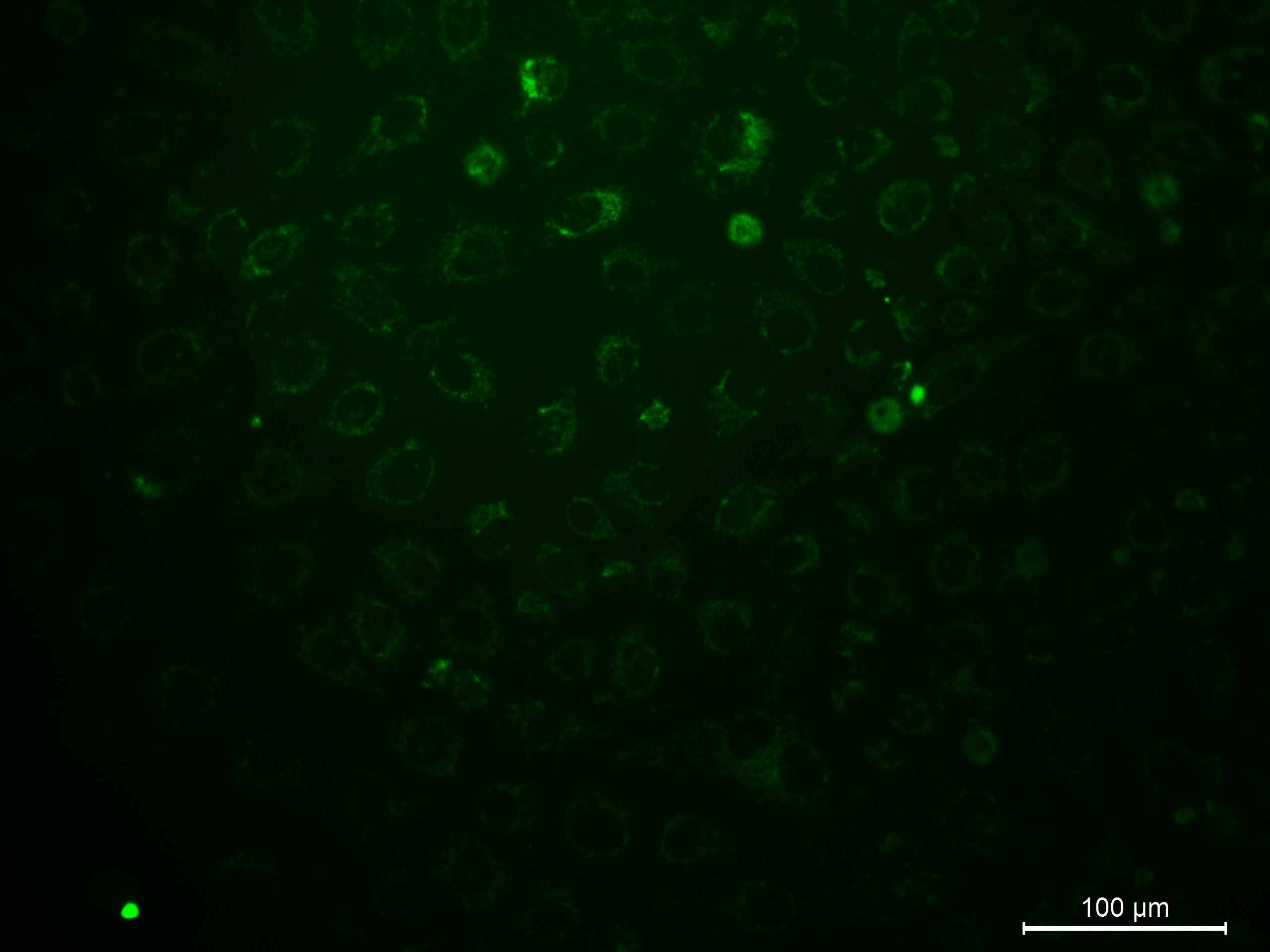

Supplement: Supplementary file 2 [file DataSheet8.zip › JC-1╢¿┴┐2/JC-1-2═╝╞1⁄4/Iohexol 1-1.tif]

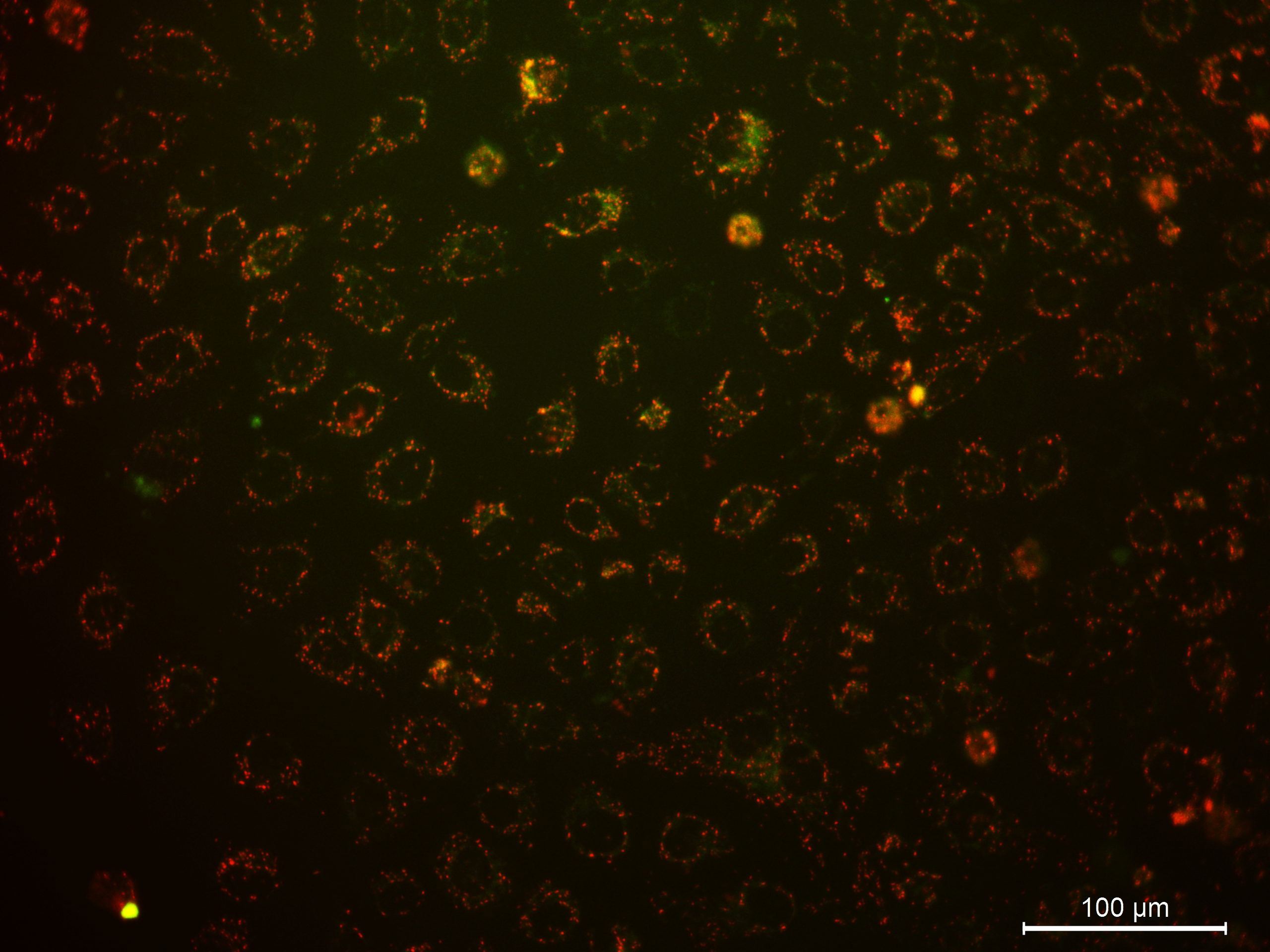

Supplement: Supplementary file 2 [file DataSheet8.zip › JC-1╢¿┴┐2/JC-1-2═╝╞1⁄4/Iohexol 1║╧.tif]

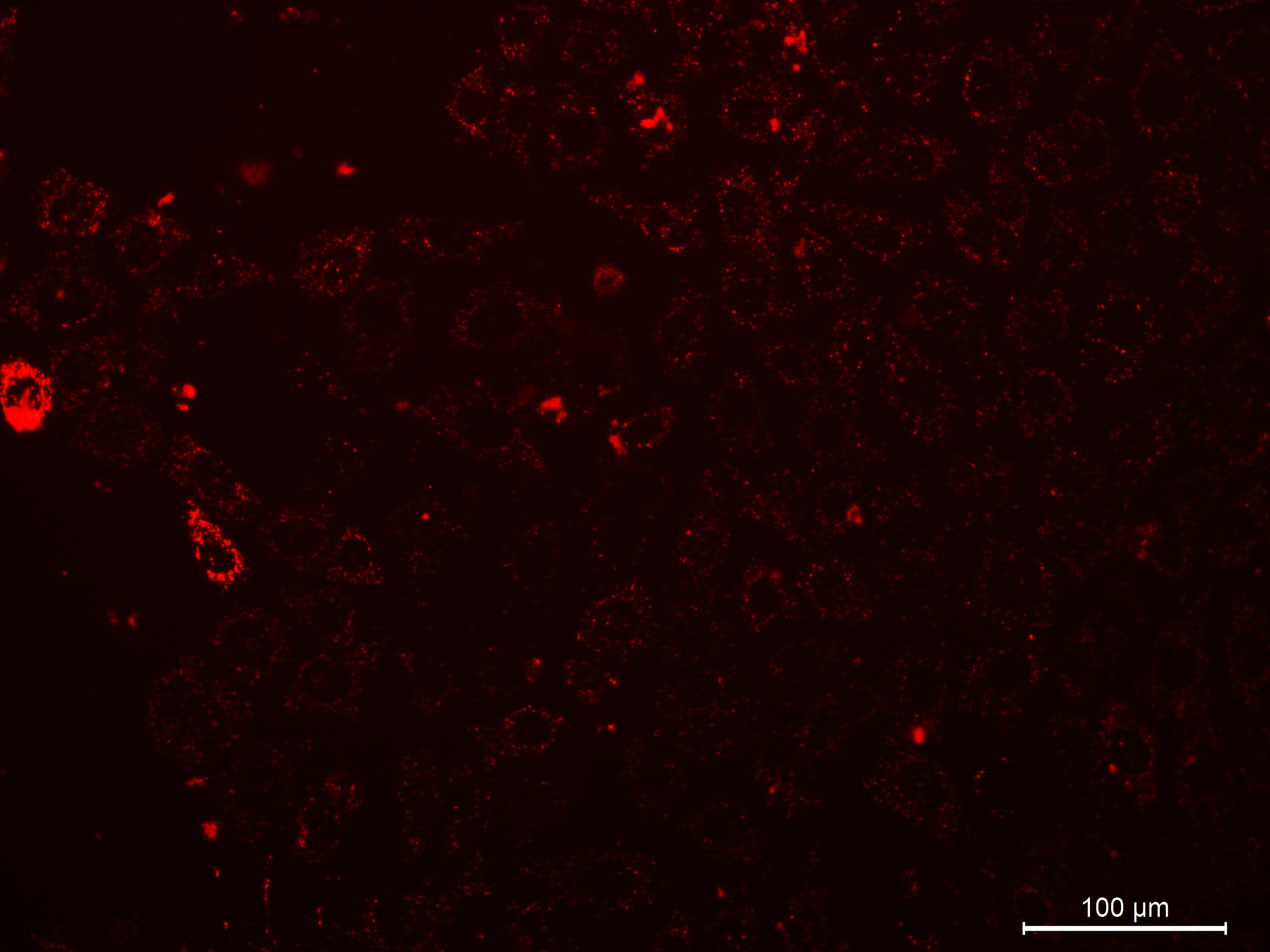

Supplement: Supplementary file 2 [file DataSheet8.zip › JC-1╢¿┴┐2/JC-1-2═╝╞1⁄4/Iohexol 2.tif]

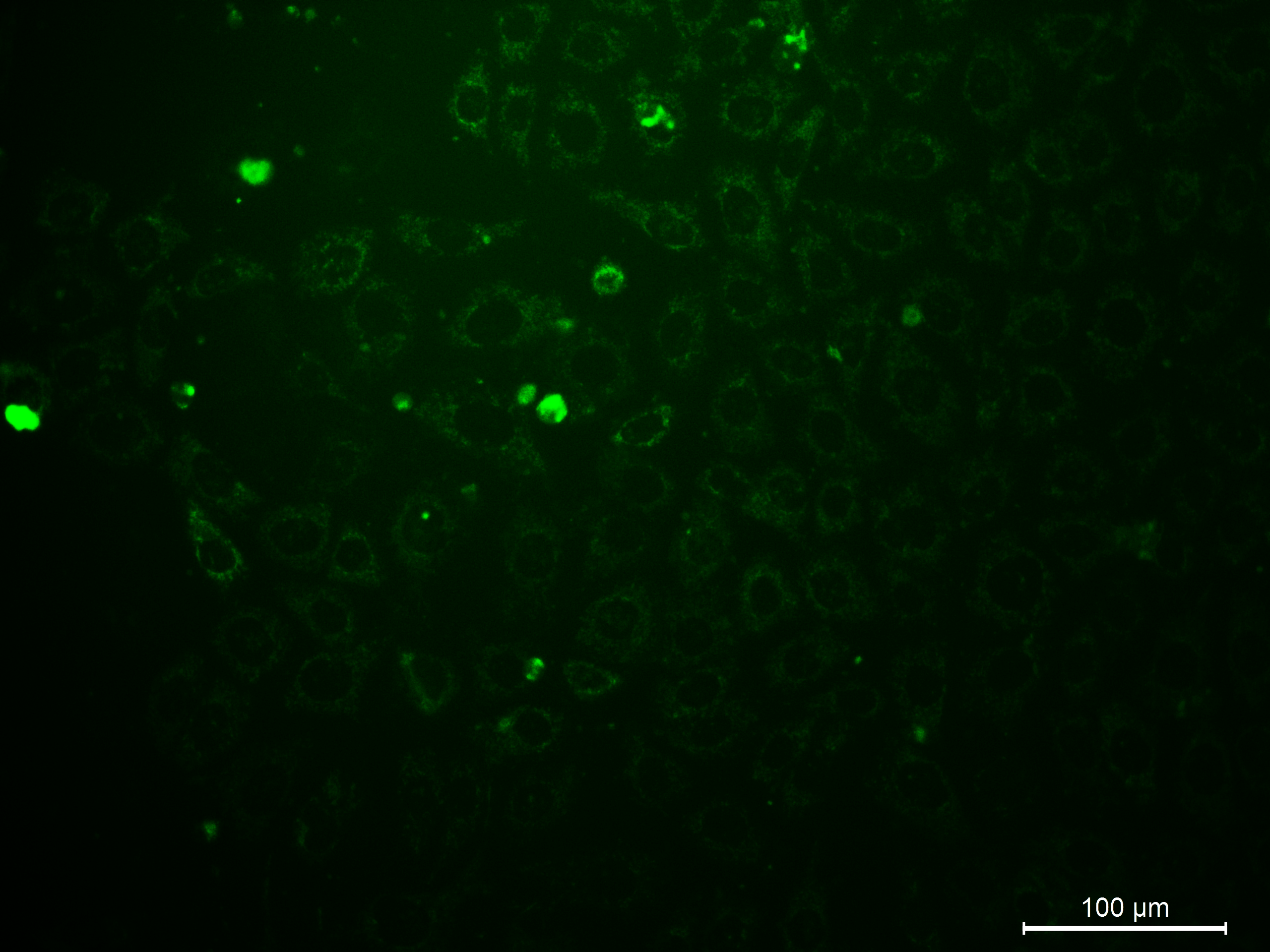

Supplement: Supplementary file 2 [file DataSheet8.zip › JC-1╢¿┴┐2/JC-1-2═╝╞1⁄4/Iohexol 2-1.tif]

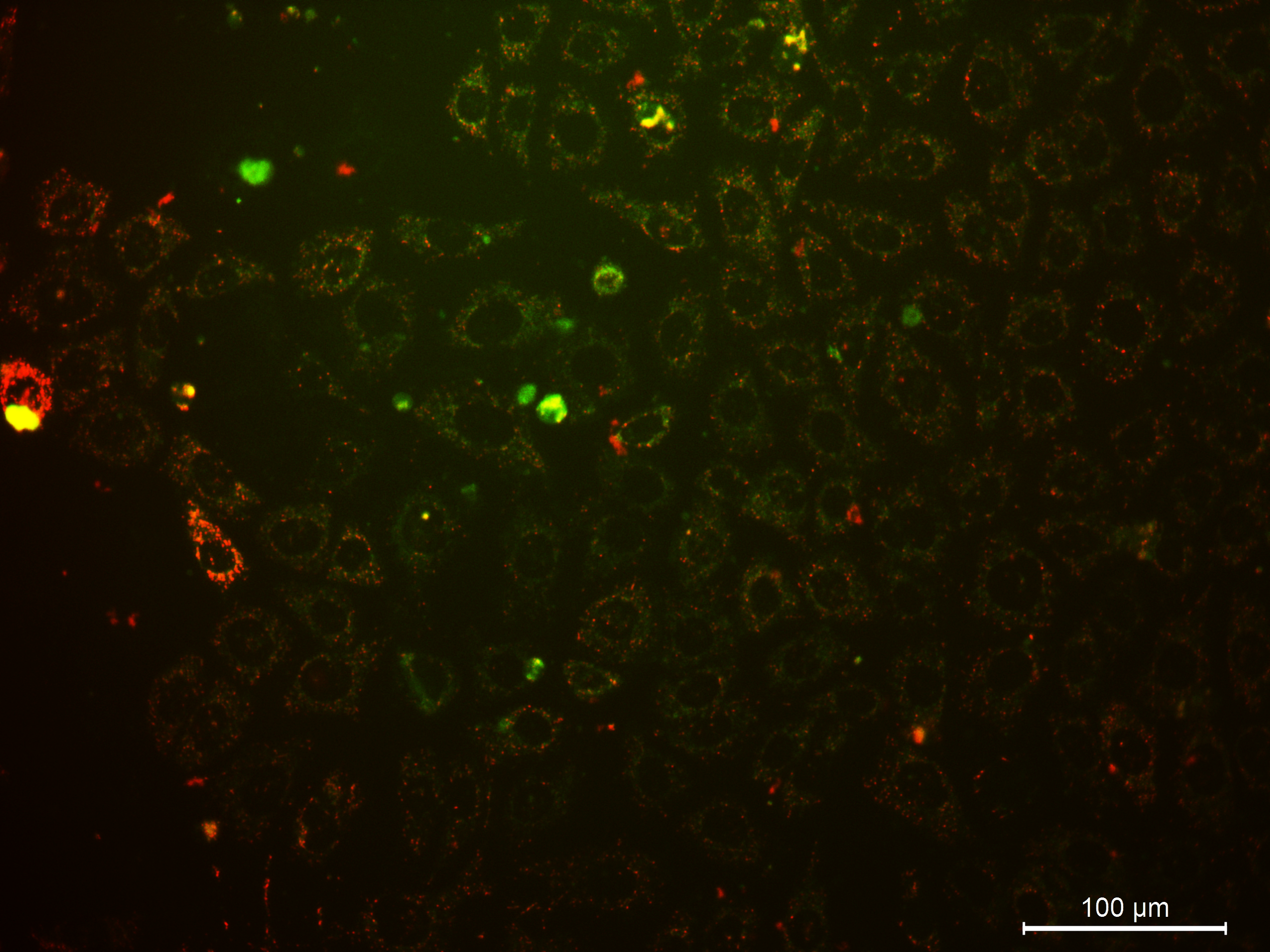

Supplement: Supplementary file 2 [file DataSheet8.zip › JC-1╢¿┴┐2/JC-1-2═╝╞1⁄4/Iohexol 2║╧.tif]

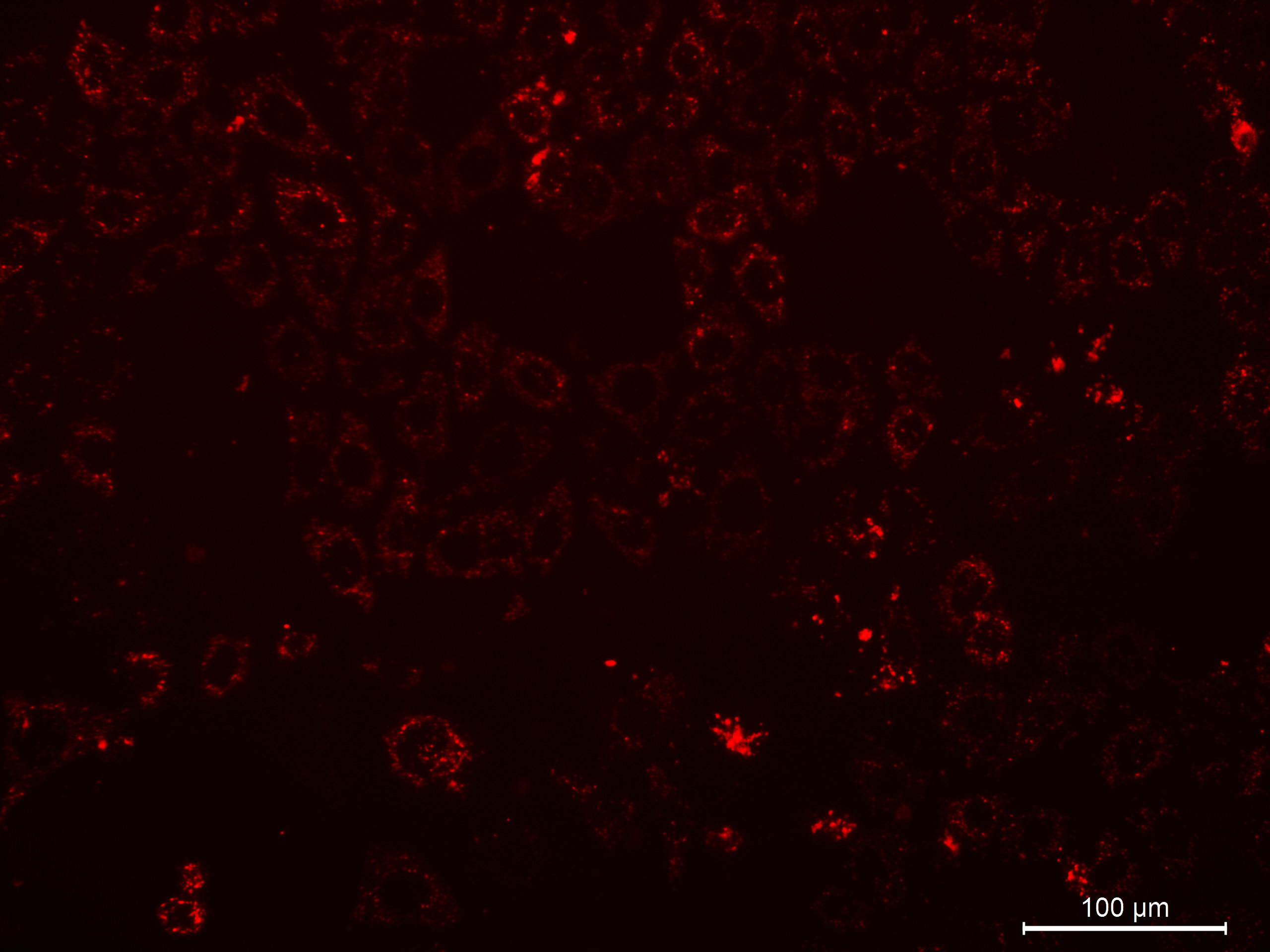

Supplement: Supplementary file 2 [file DataSheet8.zip › JC-1╢¿┴┐2/JC-1-2═╝╞1⁄4/Iohexol 3.tif]

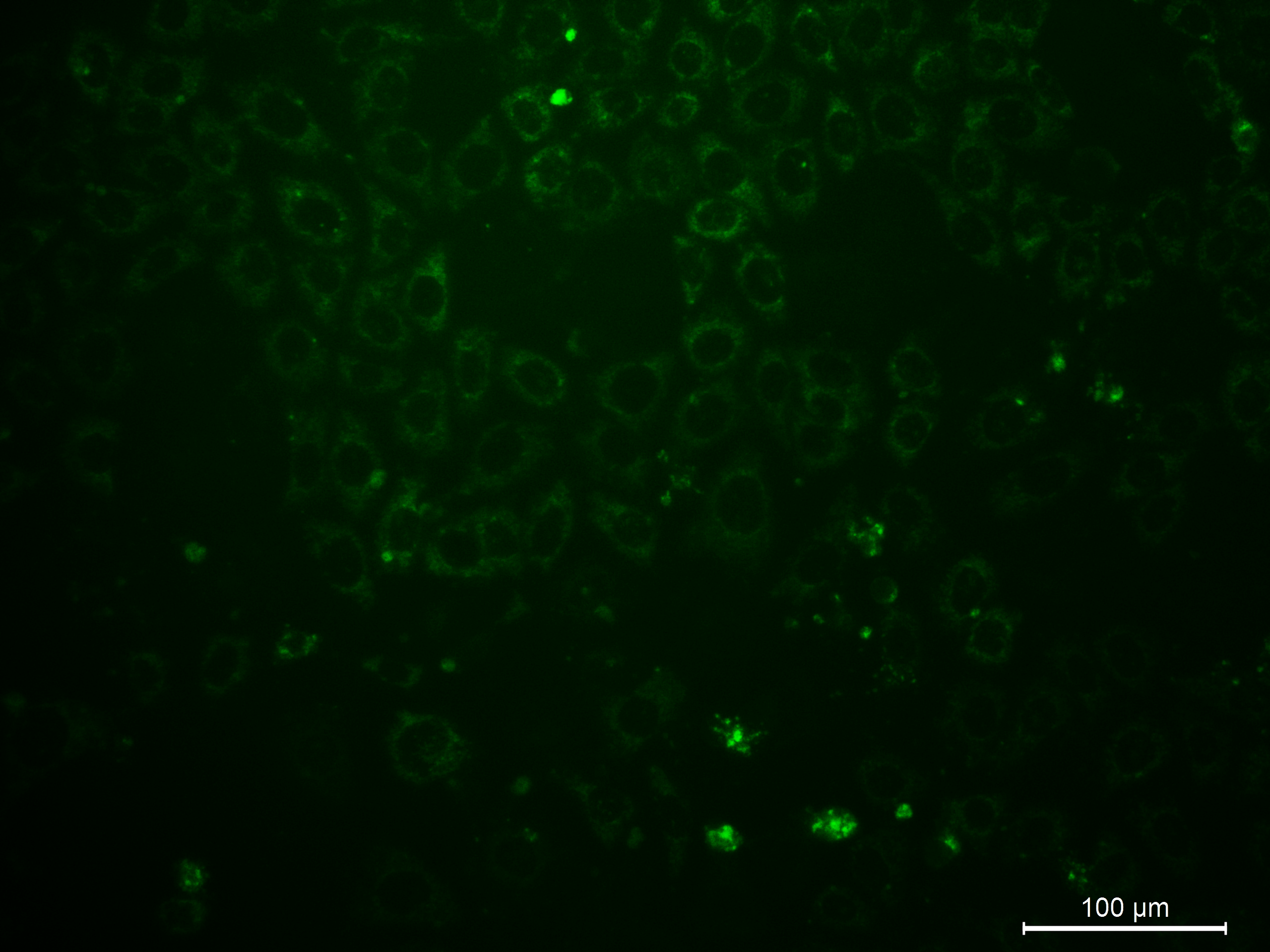

Supplement: Supplementary file 2 [file DataSheet8.zip › JC-1╢¿┴┐2/JC-1-2═╝╞1⁄4/Iohexol 3-1.tif]

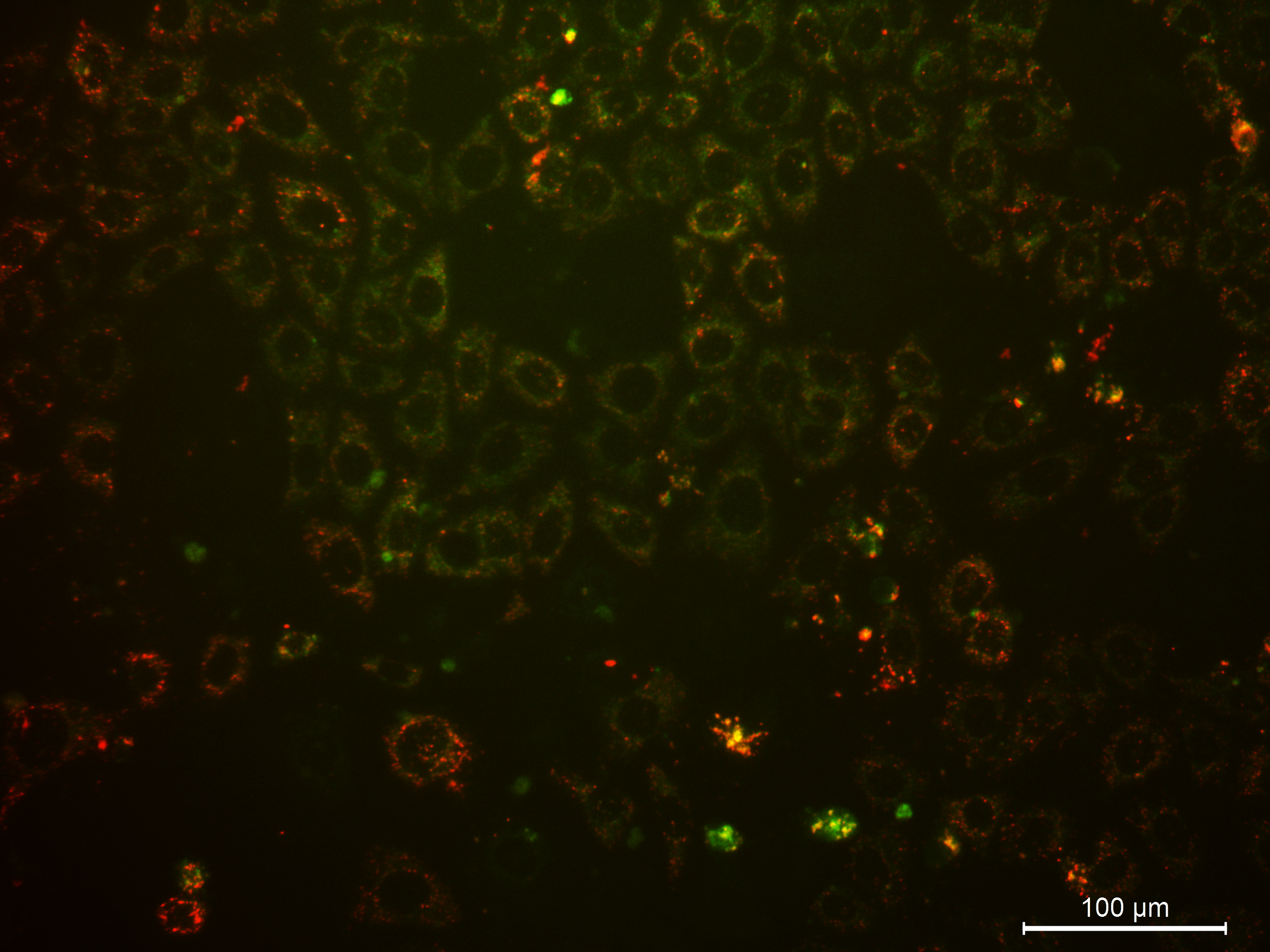

Supplement: Supplementary file 2 [file DataSheet8.zip › JC-1╢¿┴┐2/JC-1-2═╝╞1⁄4/Iohexol 3║╧.tif]

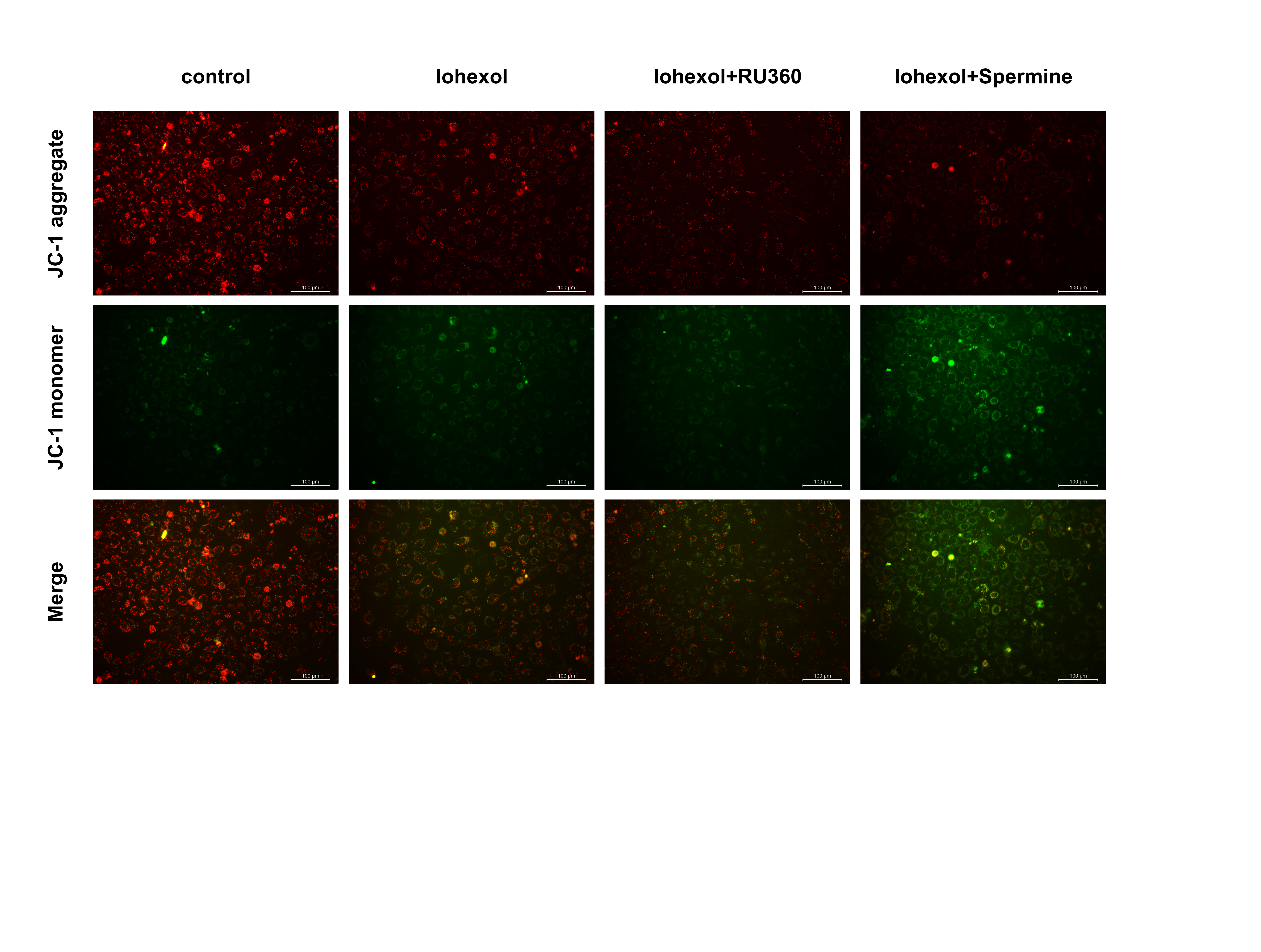

Supplement: Supplementary file 2 [file DataSheet8.zip › JC-1╢¿┴┐2/JC-1-2═╝╞1⁄4/JC-1-2║╧.tif]

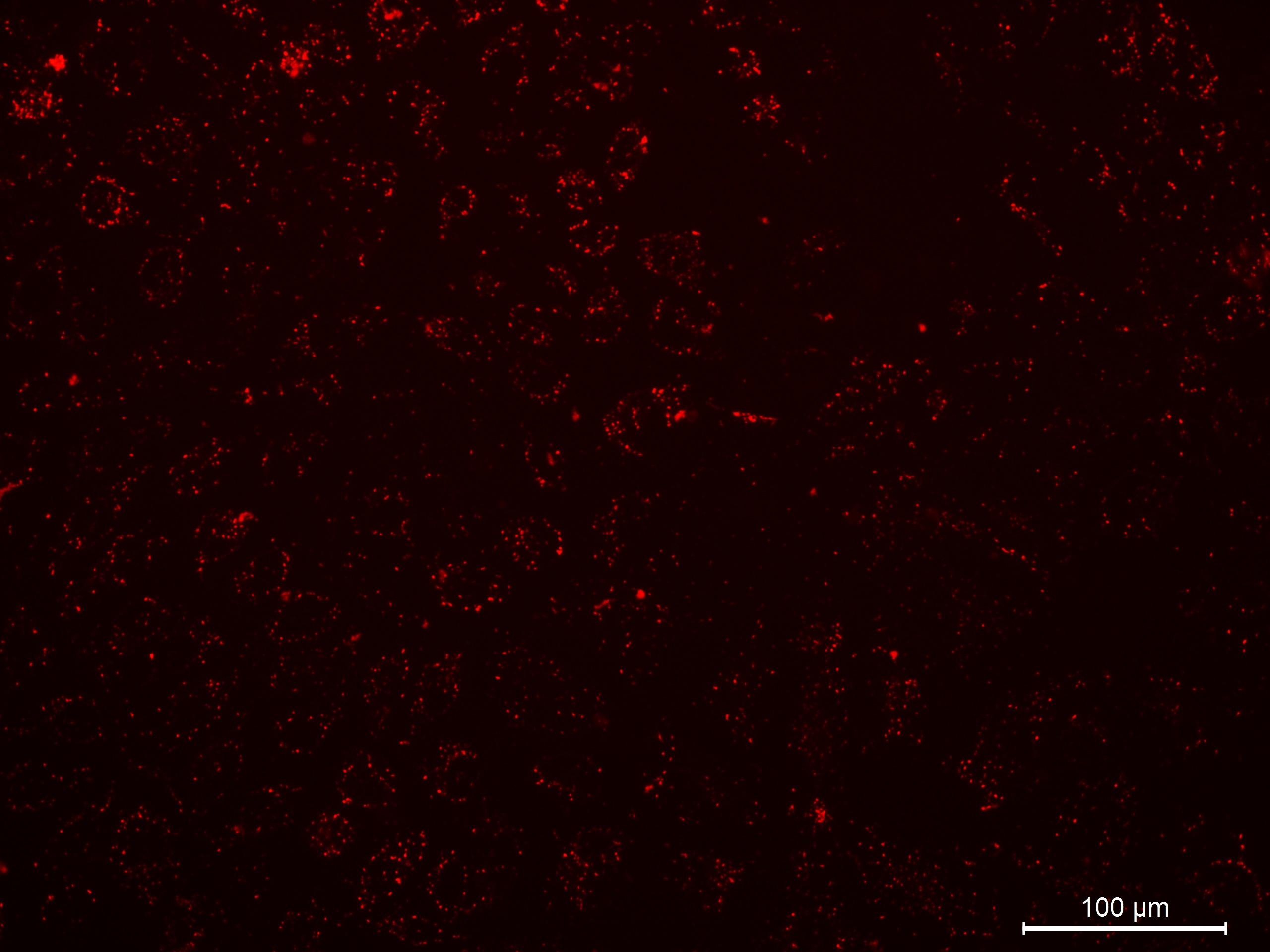

Supplement: Supplementary file 2 [file DataSheet8.zip › JC-1╢¿┴┐2/JC-1-2═╝╞1⁄4/RU360+Iohexol 1.tif]

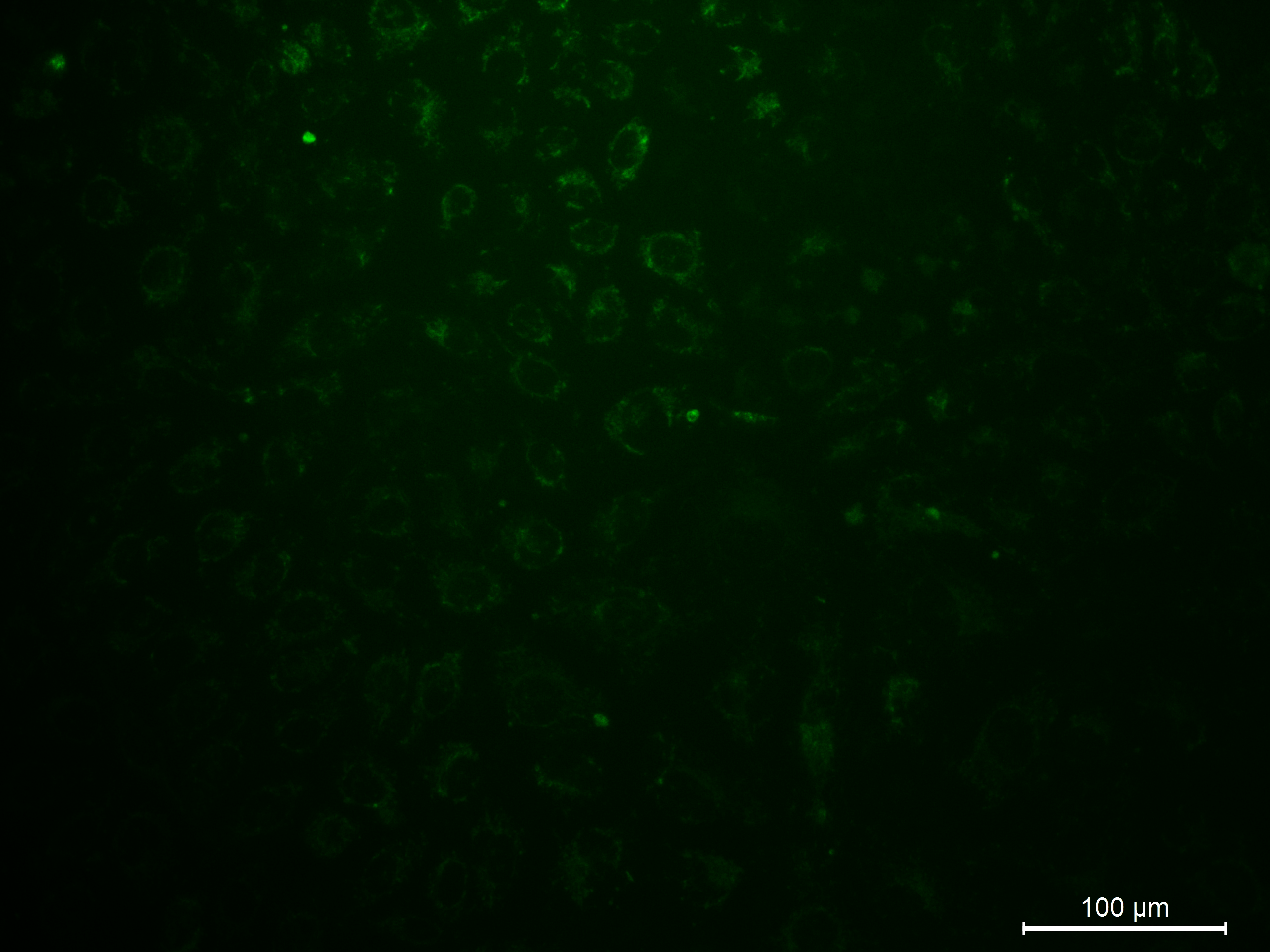

Supplement: Supplementary file 2 [file DataSheet8.zip › JC-1╢¿┴┐2/JC-1-2═╝╞1⁄4/RU360+Iohexol 1-1.tif]

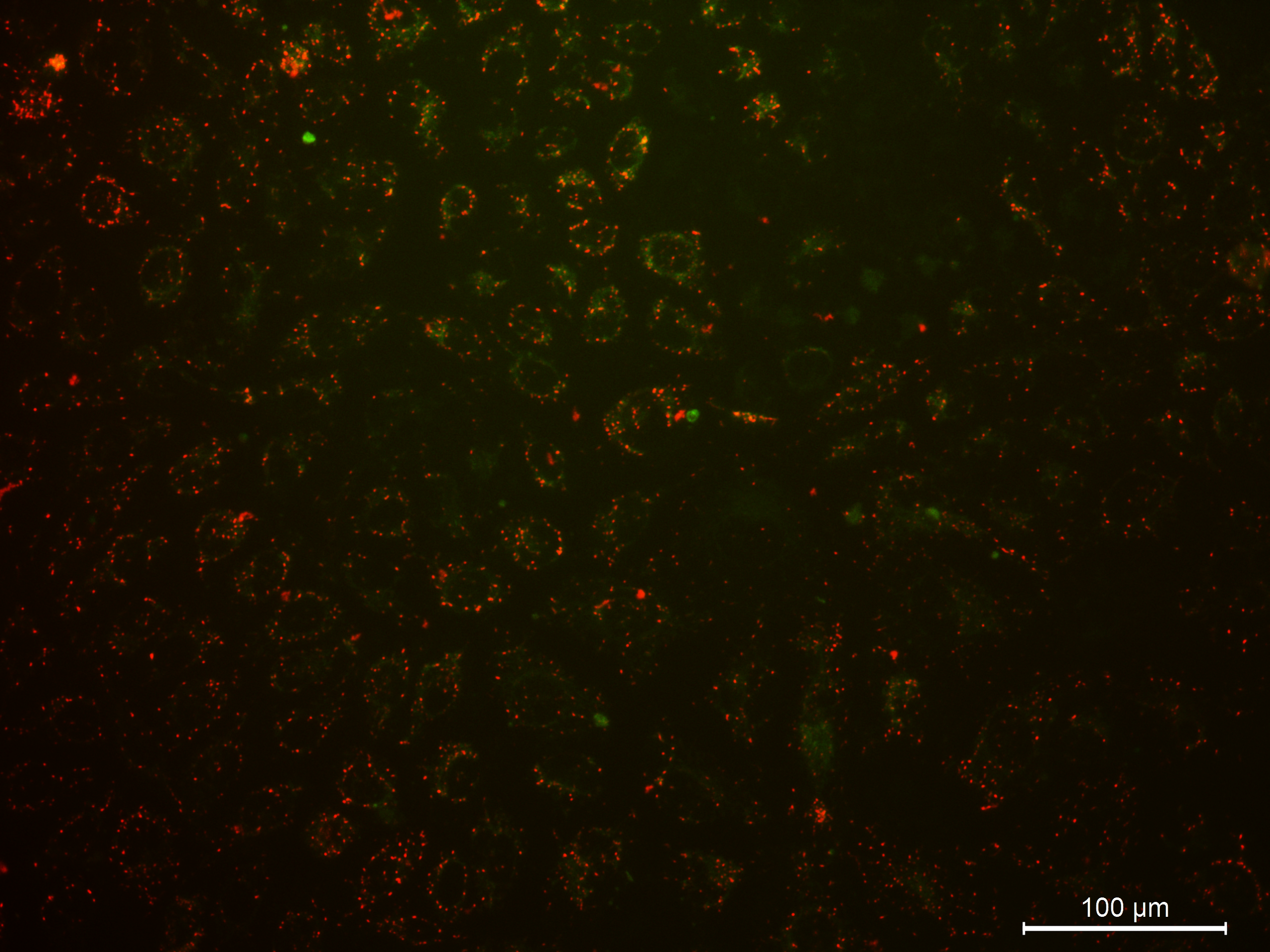

Supplement: Supplementary file 2 [file DataSheet8.zip › JC-1╢¿┴┐2/JC-1-2═╝╞1⁄4/RU360+Iohexol 1║╧.tif]

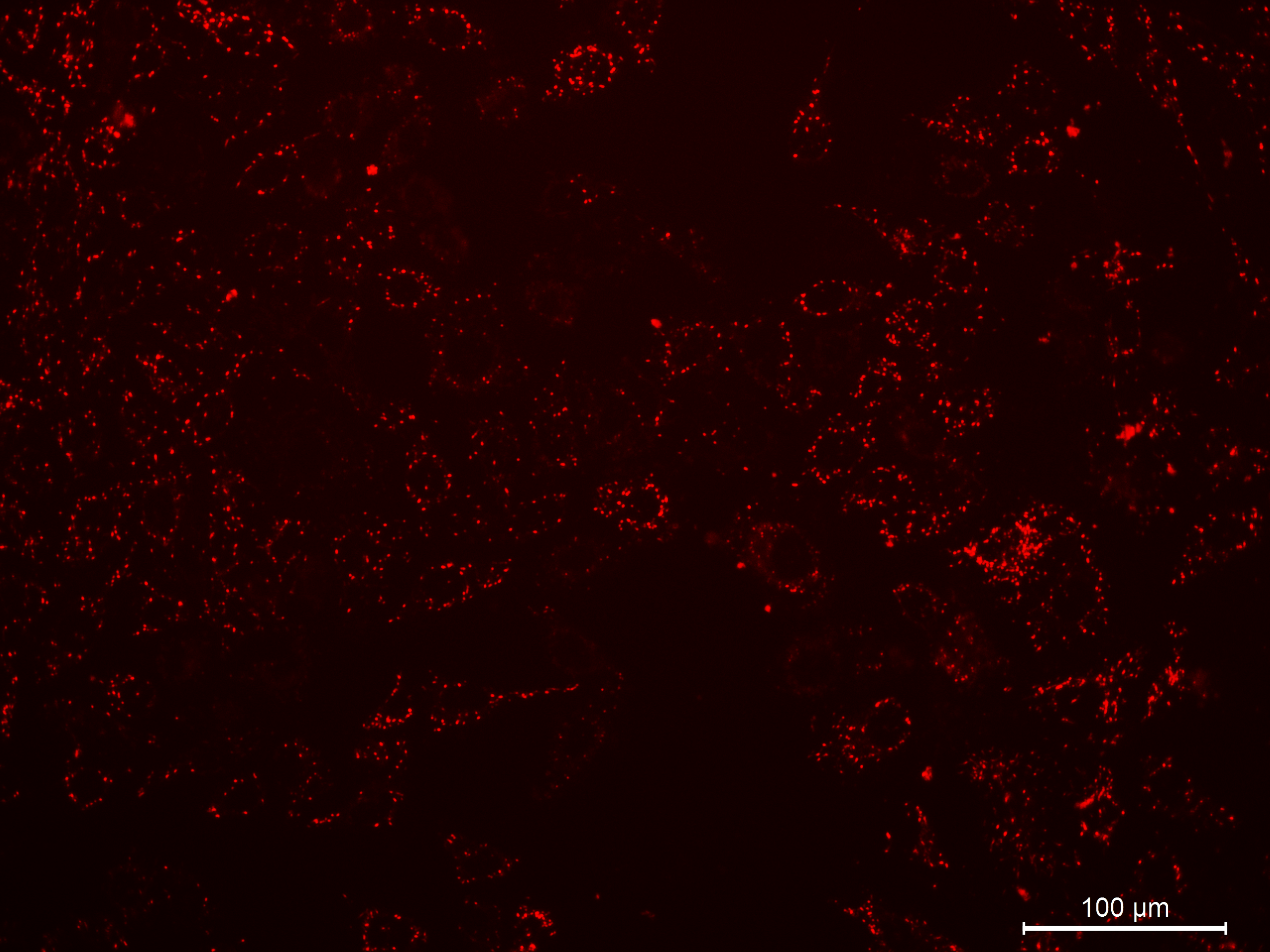

Supplement: Supplementary file 2 [file DataSheet8.zip › JC-1╢¿┴┐2/JC-1-2═╝╞1⁄4/RU360+Iohexol 2.tif]

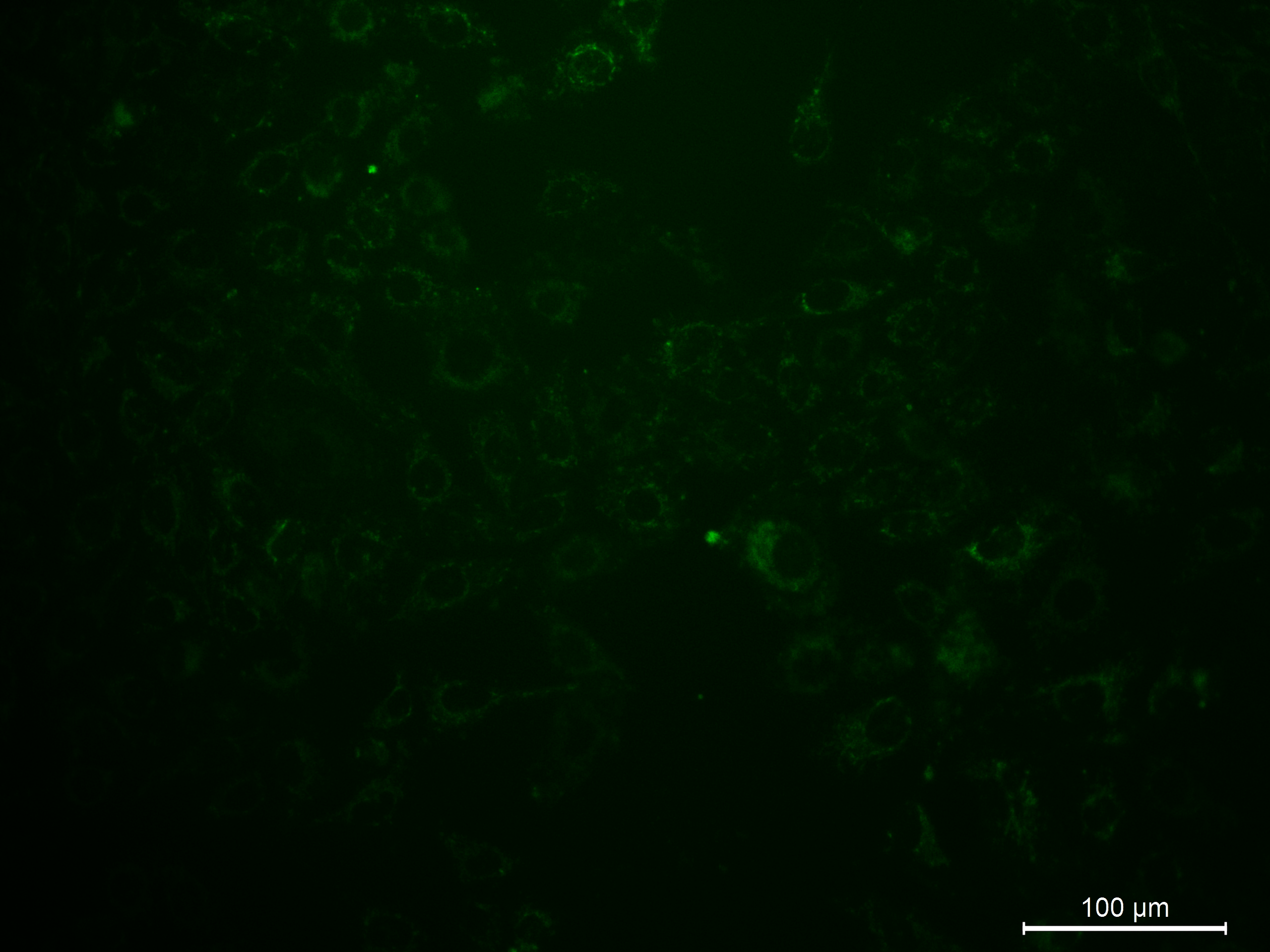

Supplement: Supplementary file 2 [file DataSheet8.zip › JC-1╢¿┴┐2/JC-1-2═╝╞1⁄4/RU360+Iohexol 2-1.tif]

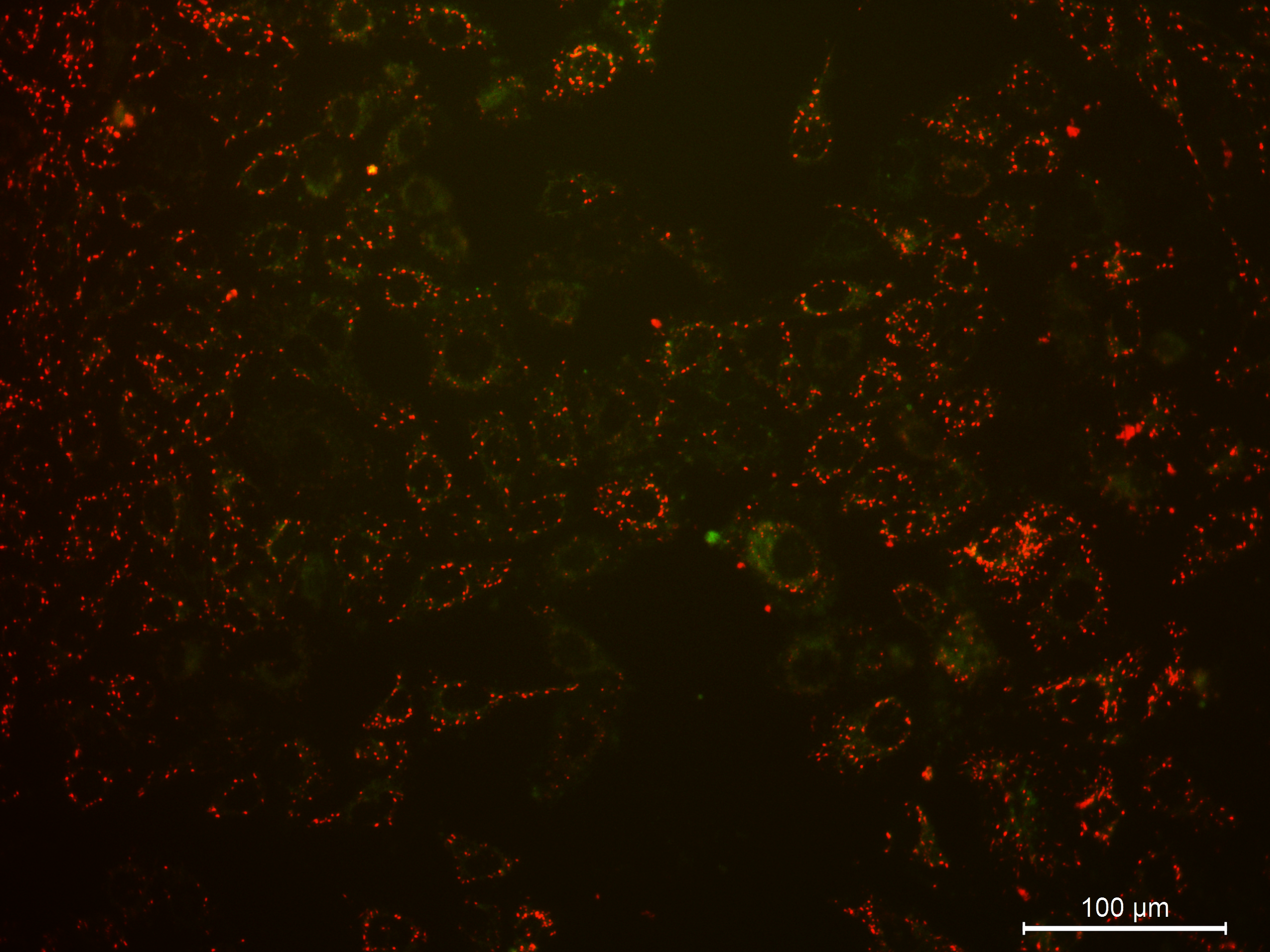

Supplement: Supplementary file 2 [file DataSheet8.zip › JC-1╢¿┴┐2/JC-1-2═╝╞1⁄4/RU360+Iohexol 2║╧.tif]

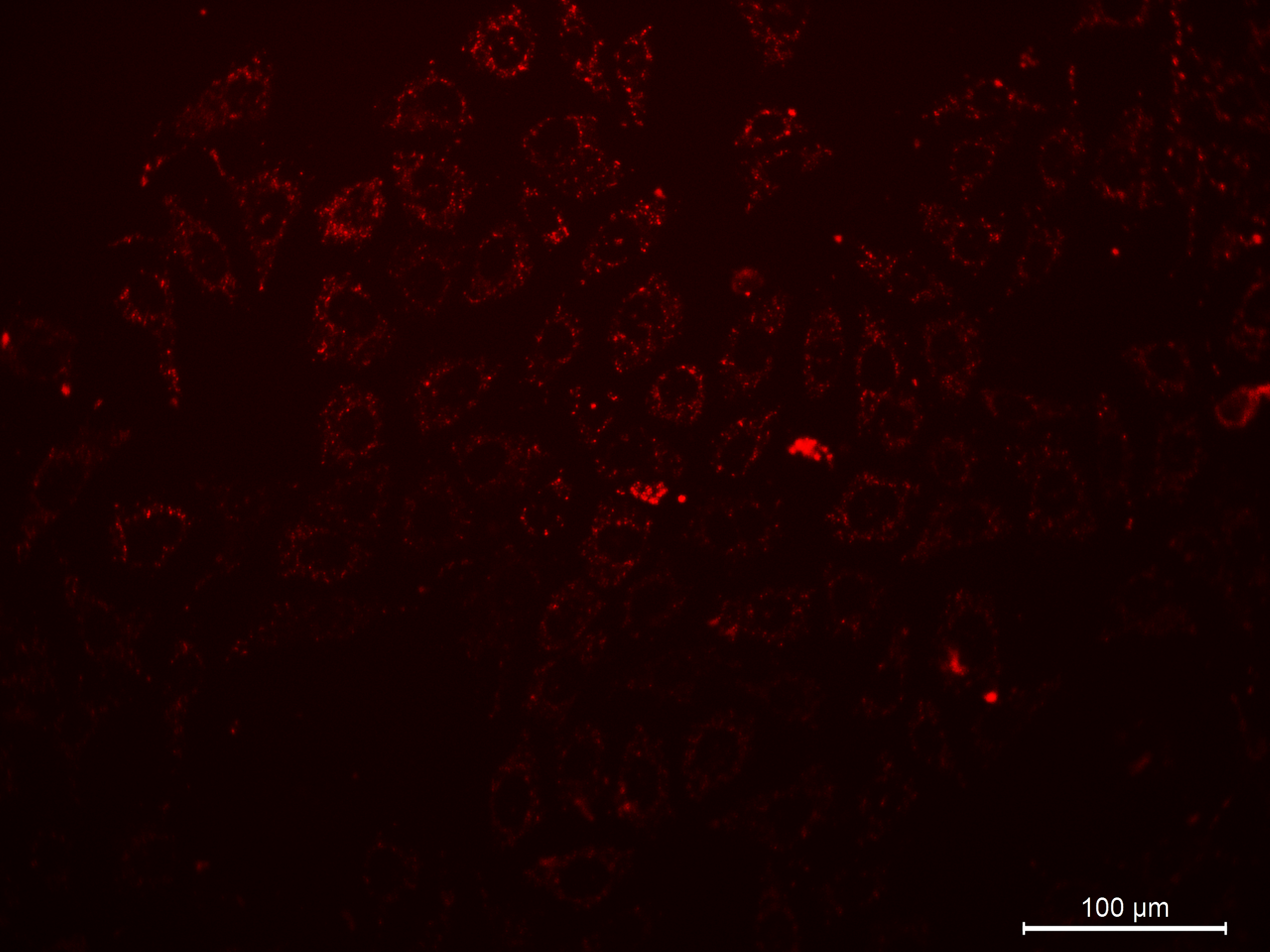

Supplement: Supplementary file 2 [file DataSheet8.zip › JC-1╢¿┴┐2/JC-1-2═╝╞1⁄4/RU360+Iohexol 3.tif]

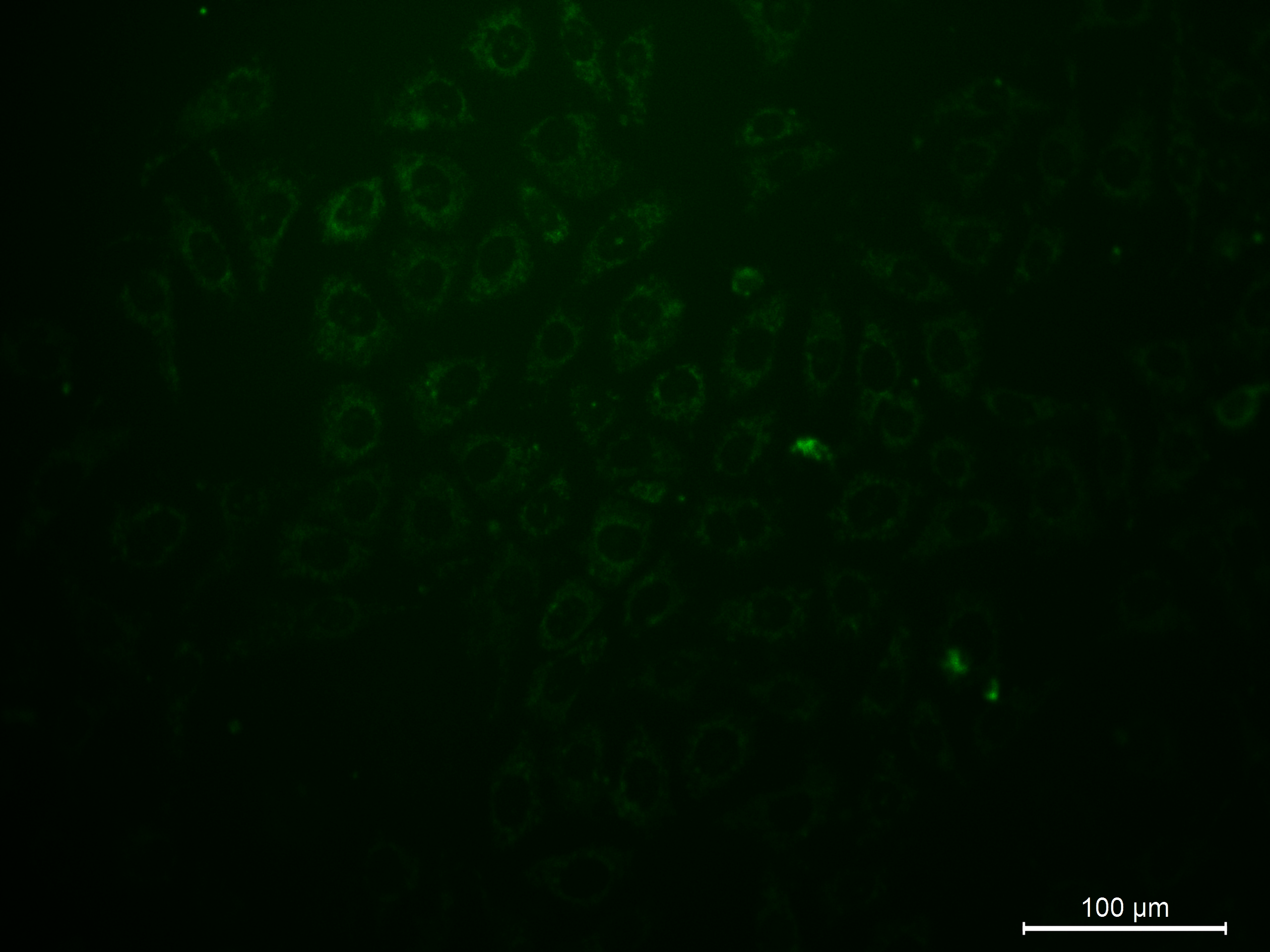

Supplement: Supplementary file 2 [file DataSheet8.zip › JC-1╢¿┴┐2/JC-1-2═╝╞1⁄4/RU360+Iohexol 3-1.tif]

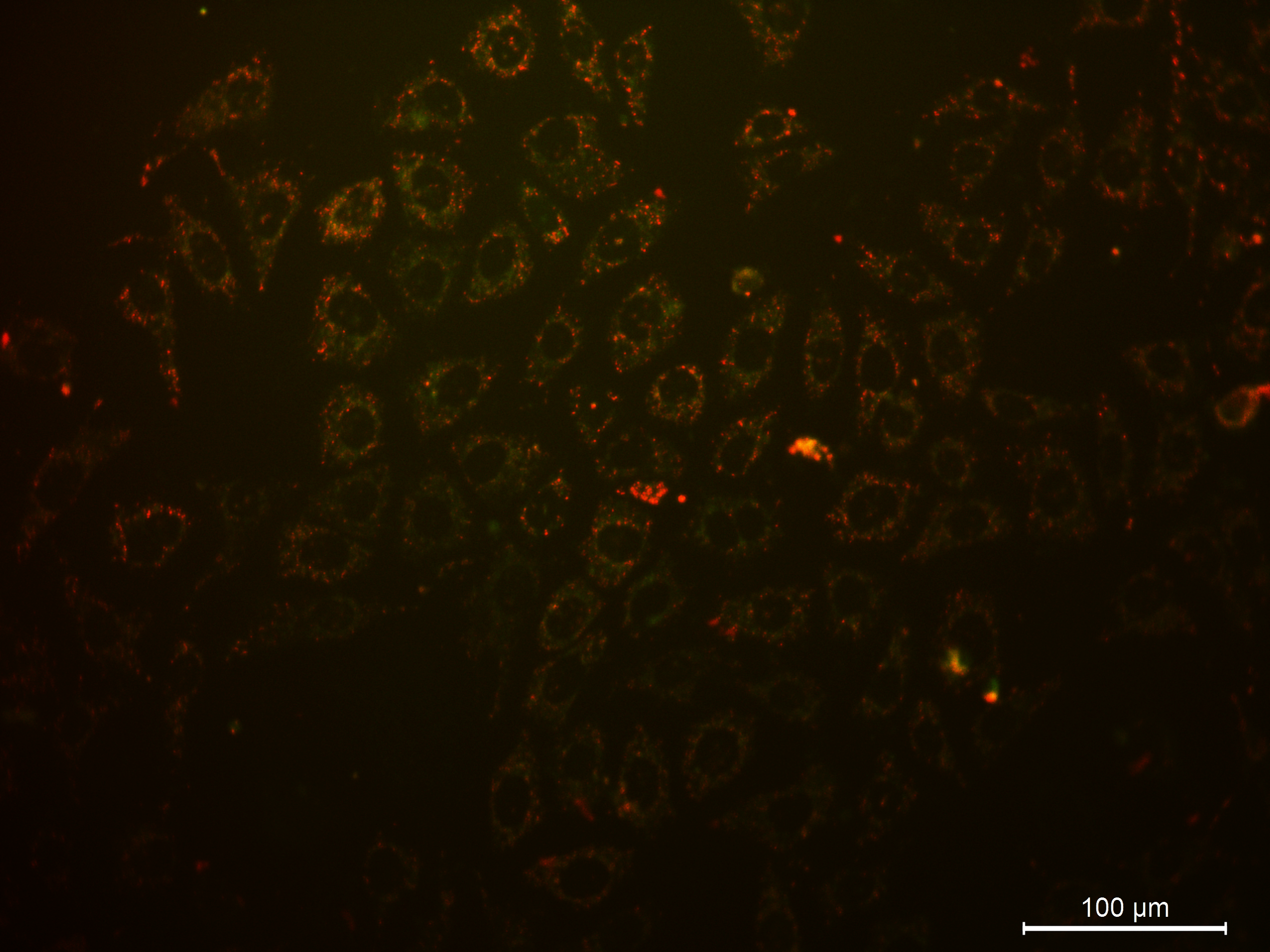

Supplement: Supplementary file 2 [file DataSheet8.zip › JC-1╢¿┴┐2/JC-1-2═╝╞1⁄4/RU360+Iohexol 3║╧.tif]

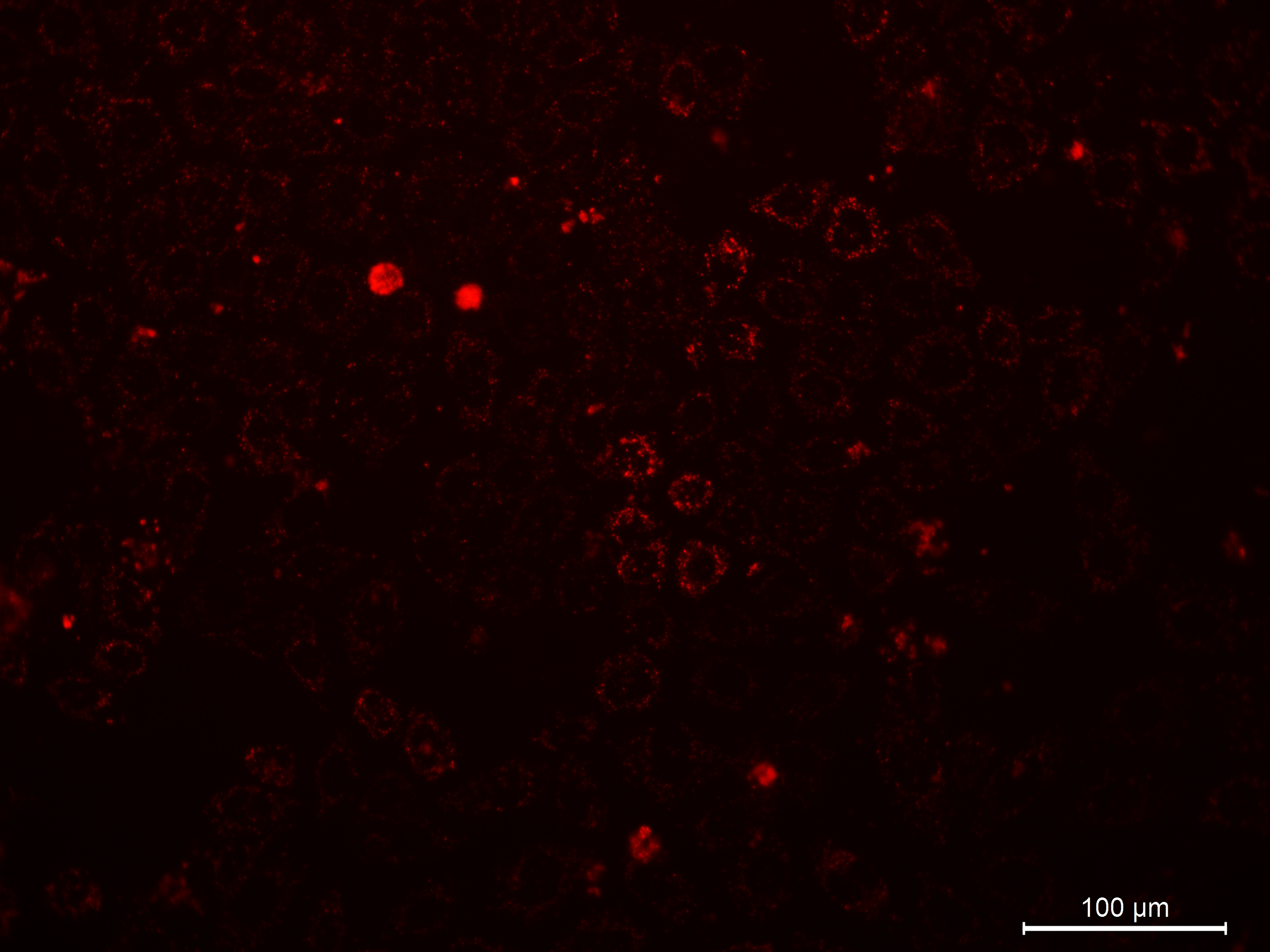

Supplement: Supplementary file 2 [file DataSheet8.zip › JC-1╢¿┴┐2/JC-1-2═╝╞1⁄4/Spermine+Iohexol 1.tif]

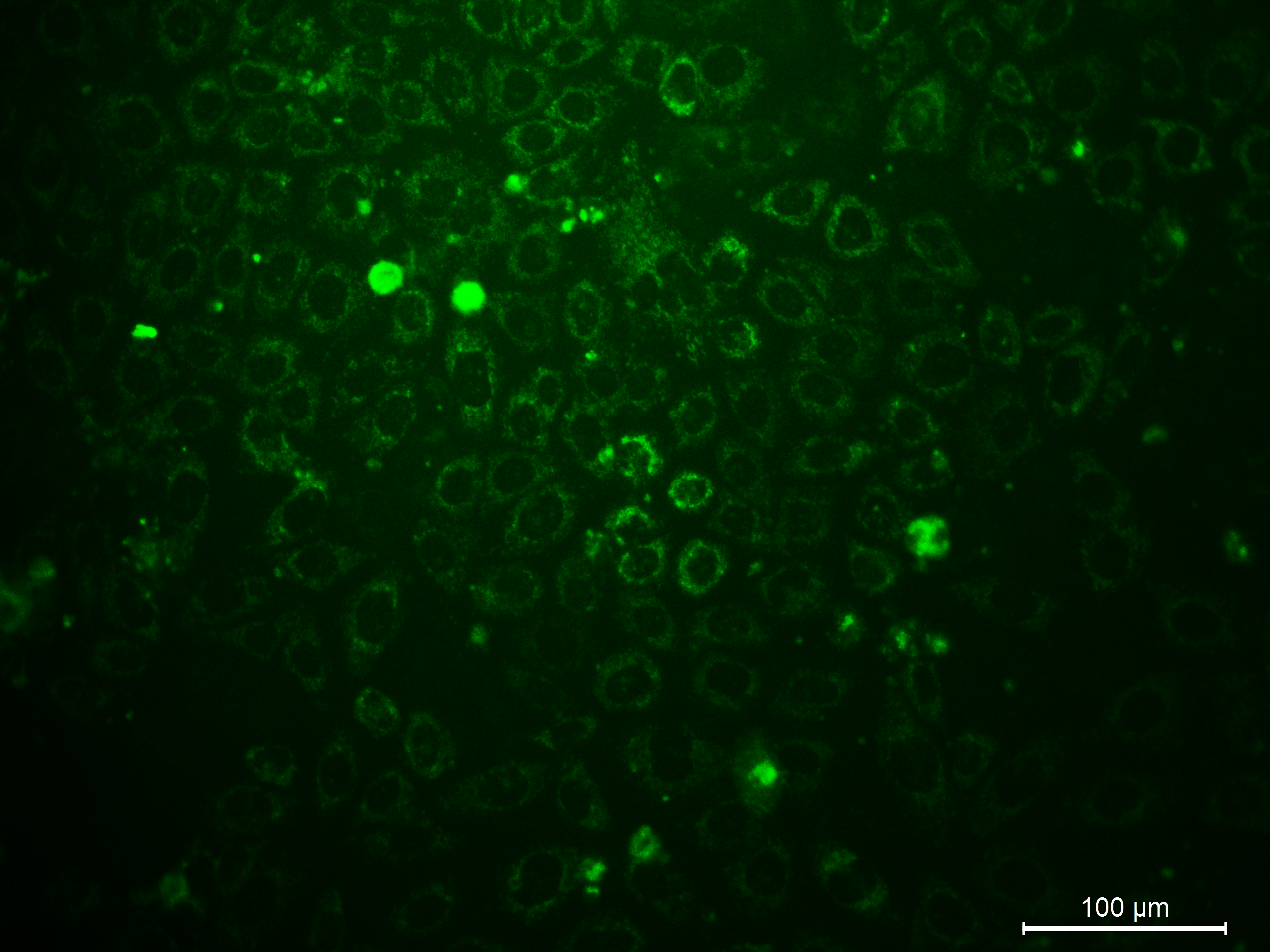

Supplement: Supplementary file 2 [file DataSheet8.zip › JC-1╢¿┴┐2/JC-1-2═╝╞1⁄4/Spermine+Iohexol 1-1.tif]

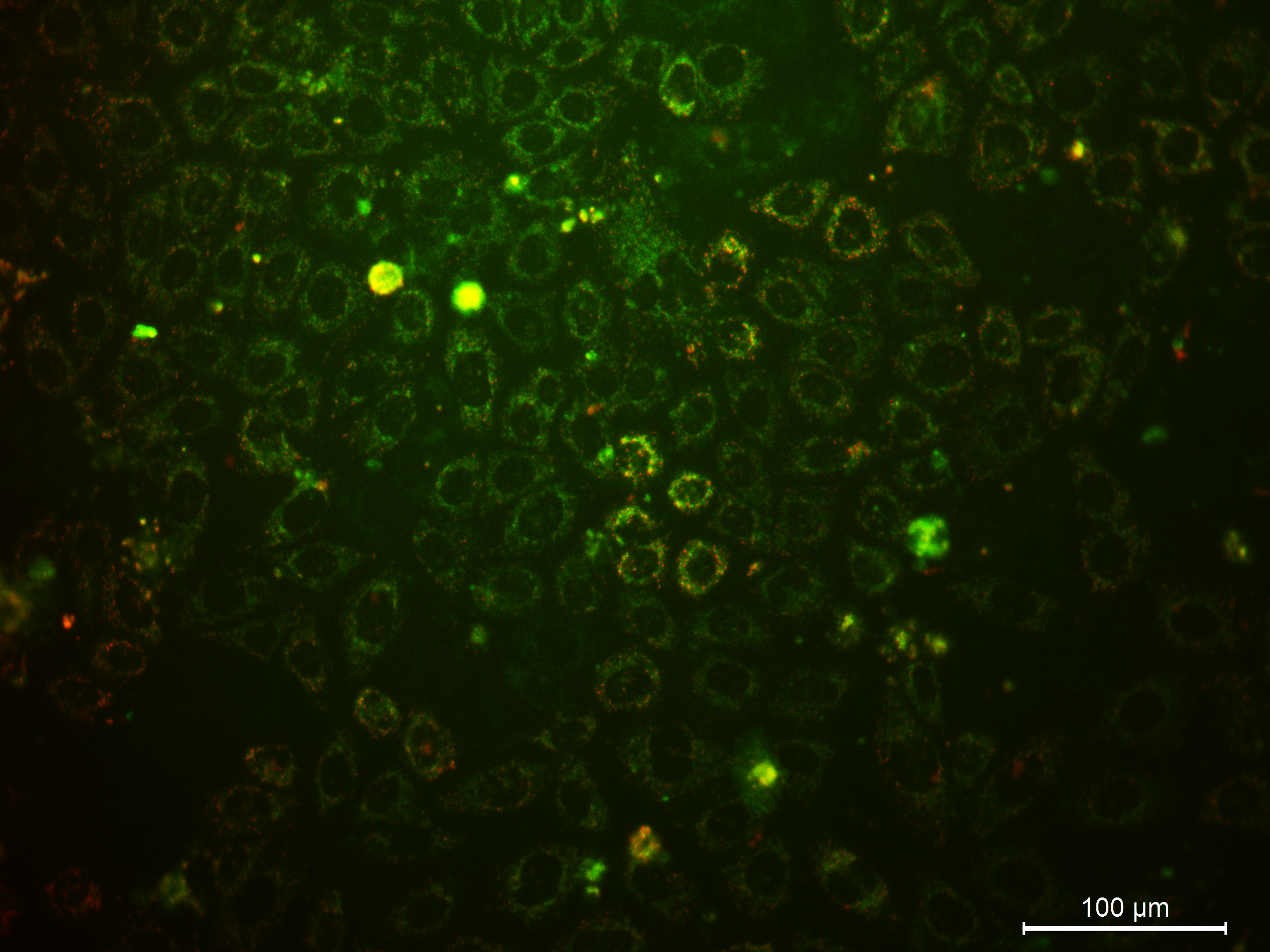

Supplement: Supplementary file 2 [file DataSheet8.zip › JC-1╢¿┴┐2/JC-1-2═╝╞1⁄4/Spermine+Iohexol 1║╧.tif]

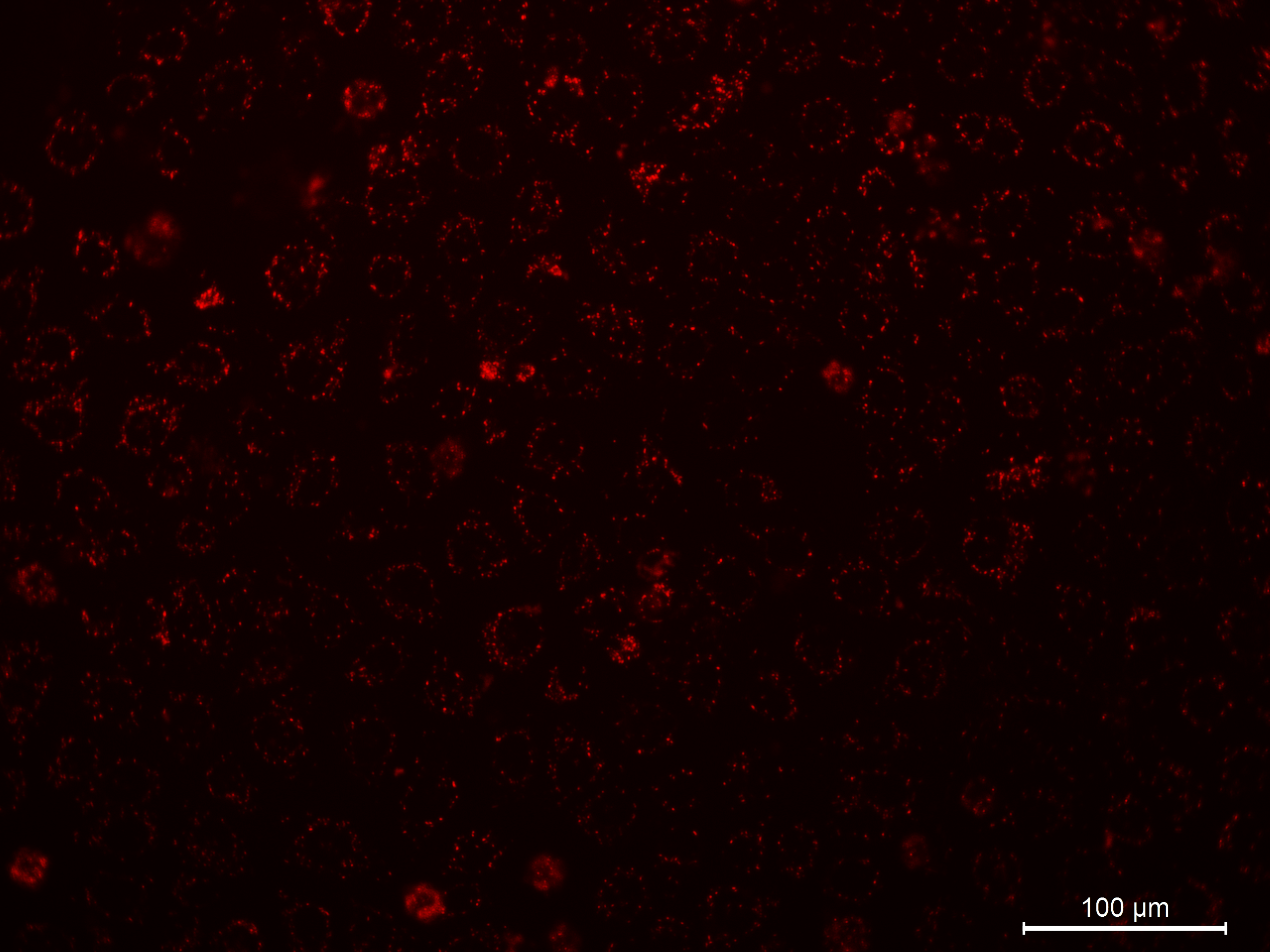

Supplement: Supplementary file 2 [file DataSheet8.zip › JC-1╢¿┴┐2/JC-1-2═╝╞1⁄4/Spermine+Iohexol 2.tif]

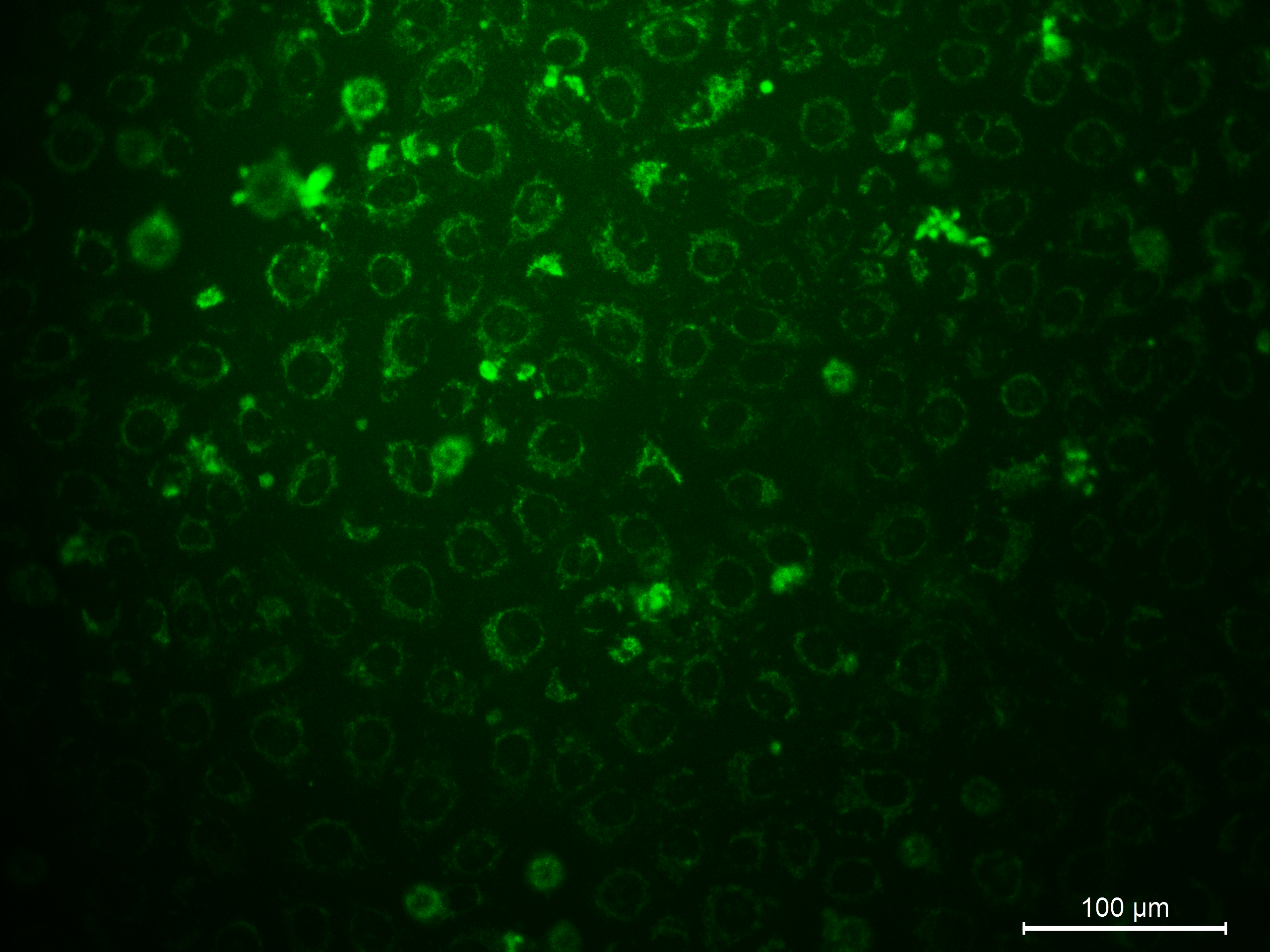

Supplement: Supplementary file 2 [file DataSheet8.zip › JC-1╢¿┴┐2/JC-1-2═╝╞1⁄4/Spermine+Iohexol 2-1.tif]

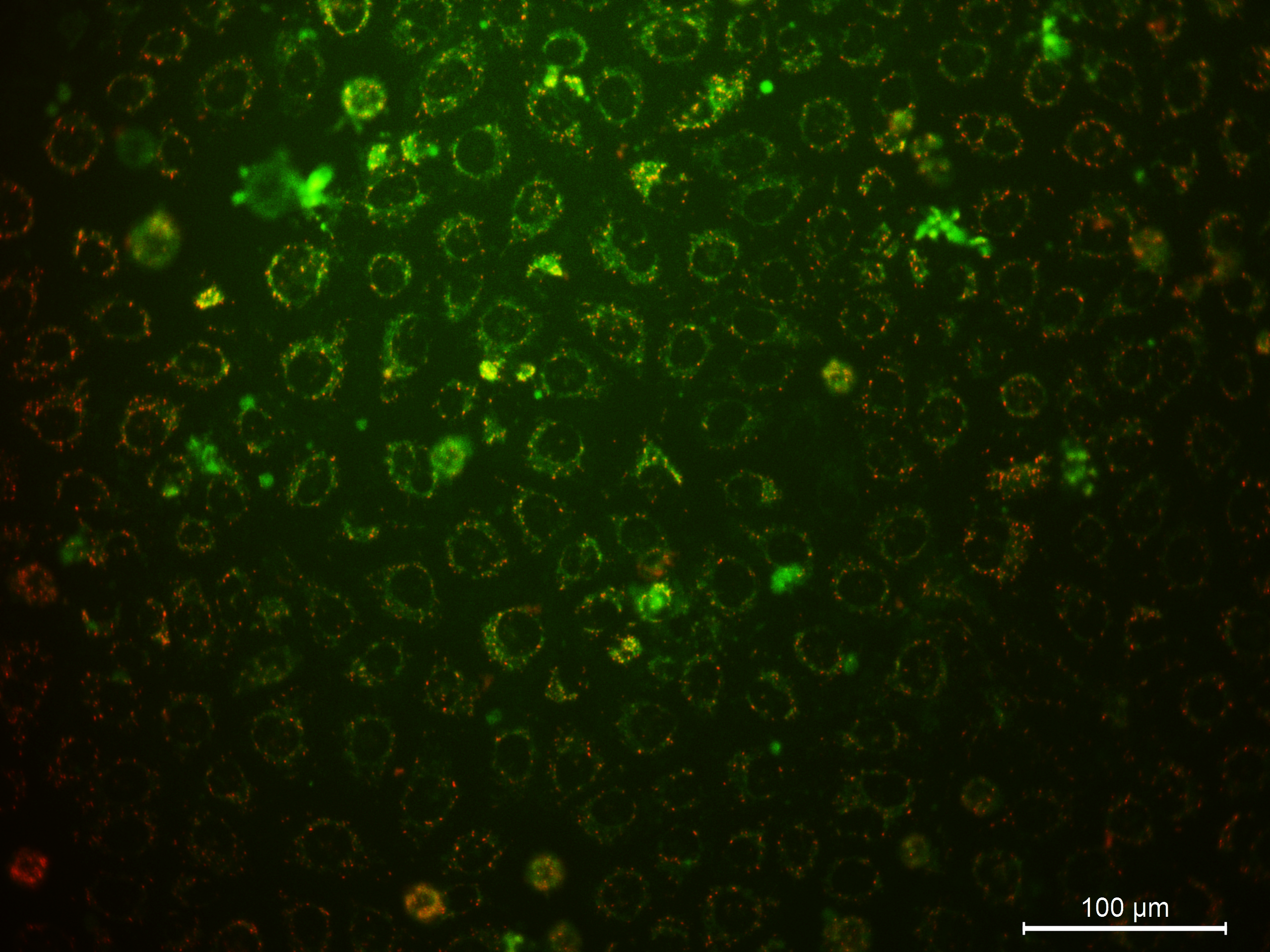

Supplement: Supplementary file 2 [file DataSheet8.zip › JC-1╢¿┴┐2/JC-1-2═╝╞1⁄4/Spermine+Iohexol 2║╧.tif]

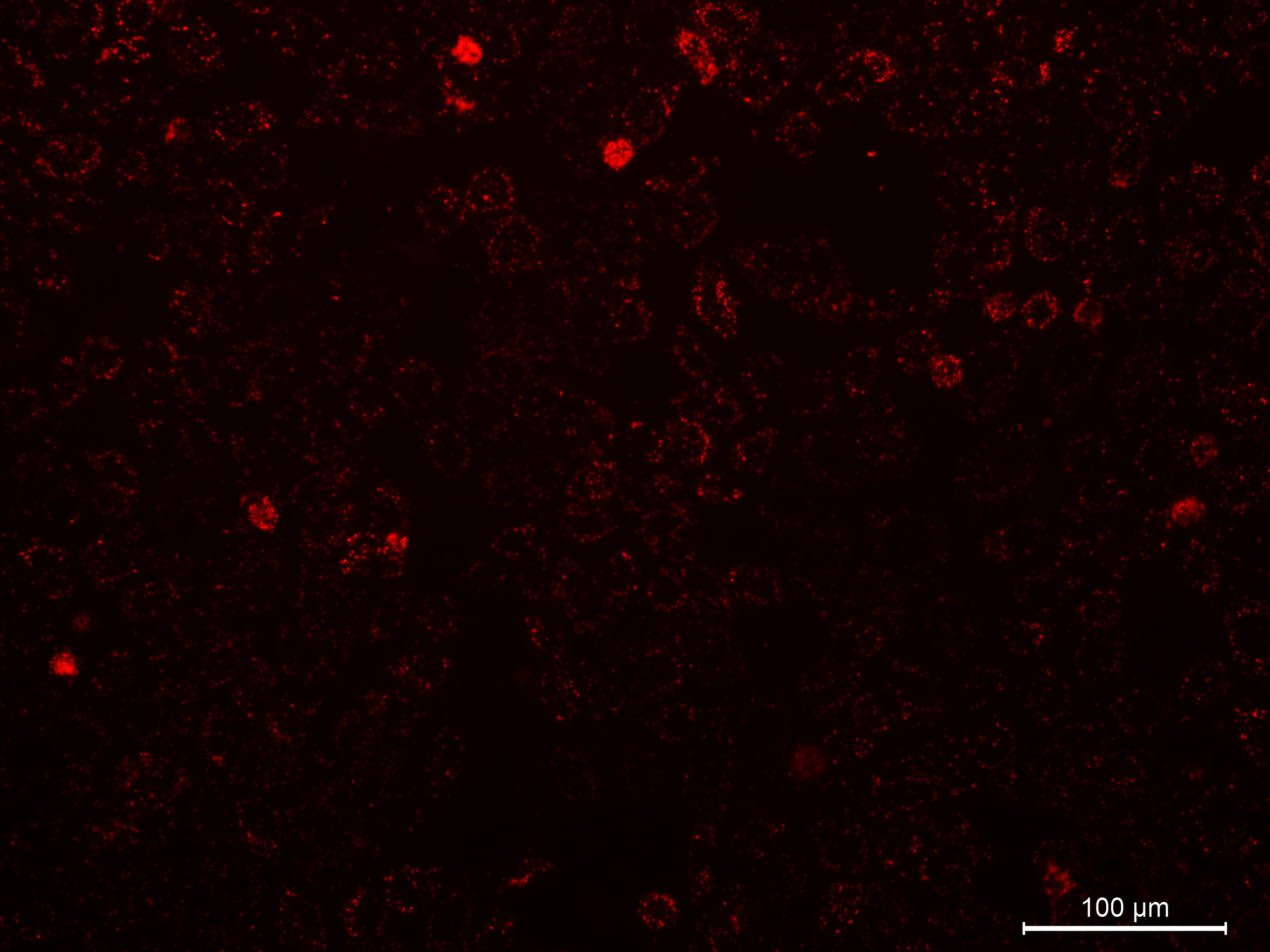

Supplement: Supplementary file 2 [file DataSheet8.zip › JC-1╢¿┴┐2/JC-1-2═╝╞1⁄4/Spermine+Iohexol 3.tif]

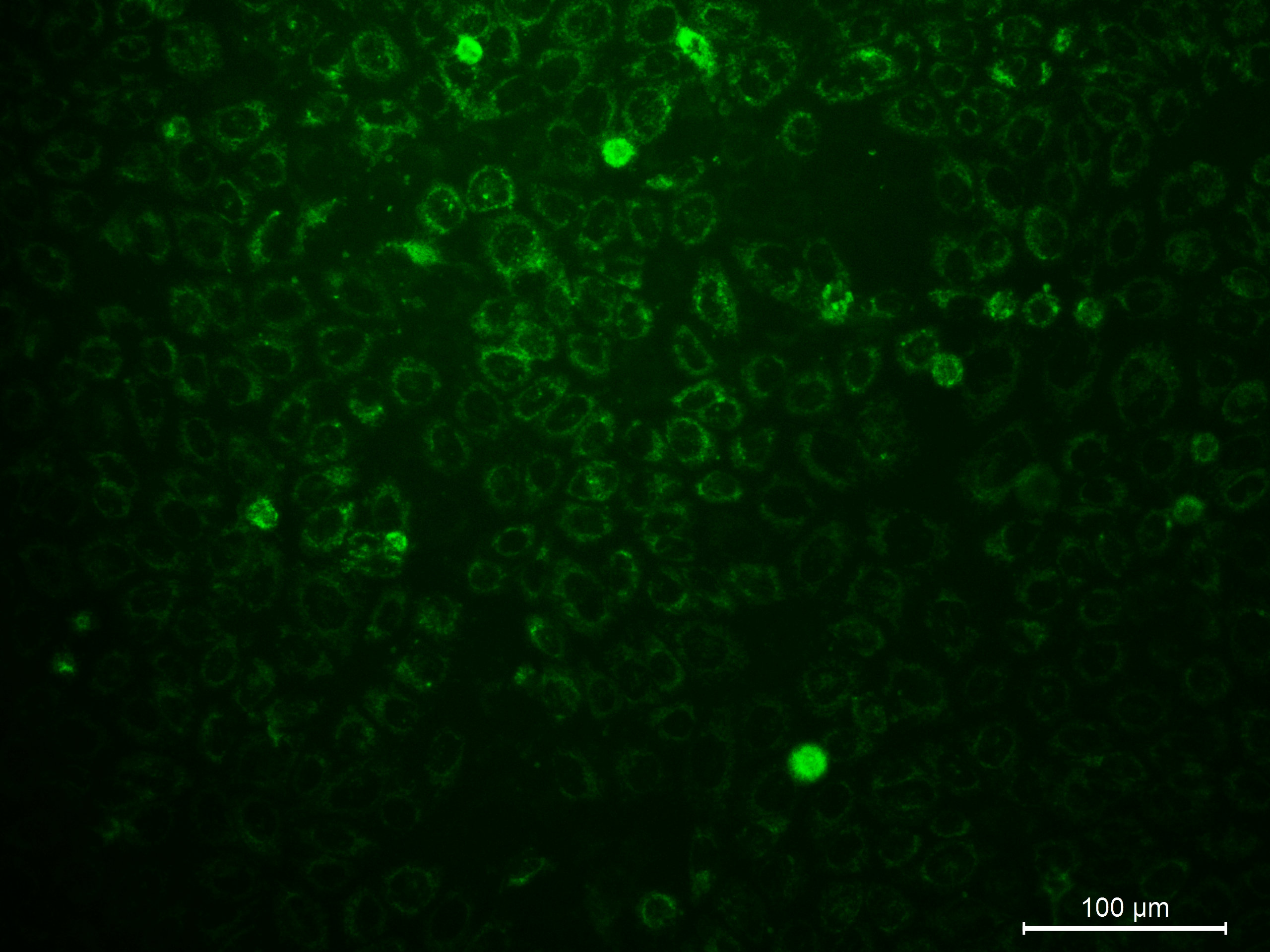

Supplement: Supplementary file 2 [file DataSheet8.zip › JC-1╢¿┴┐2/JC-1-2═╝╞1⁄4/Spermine+Iohexol 3-1.tif]

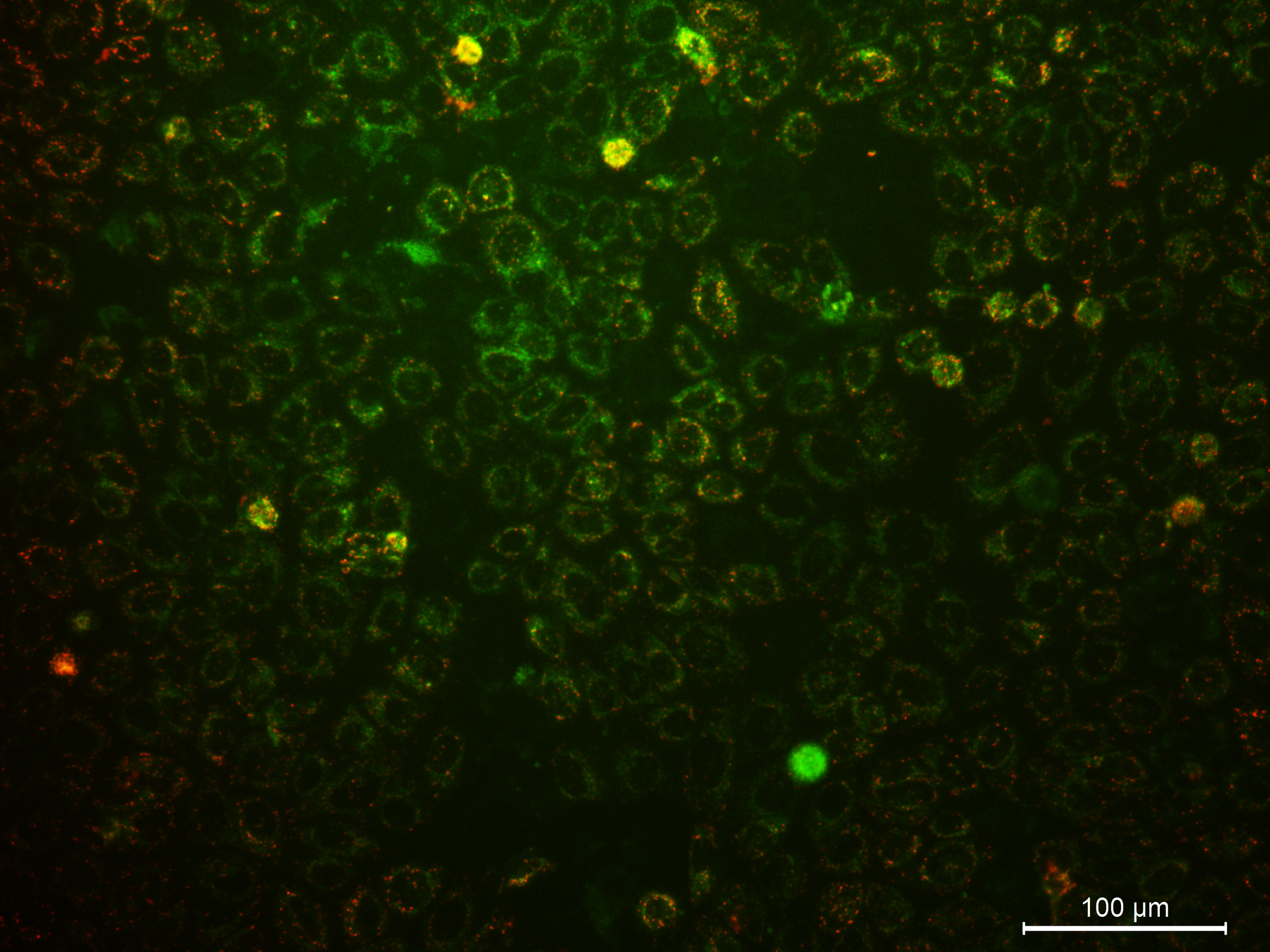

Supplement: Supplementary file 2 [file DataSheet8.zip › JC-1╢¿┴┐2/JC-1-2═╝╞1⁄4/Spermine+Iohexol 3║╧.tif]

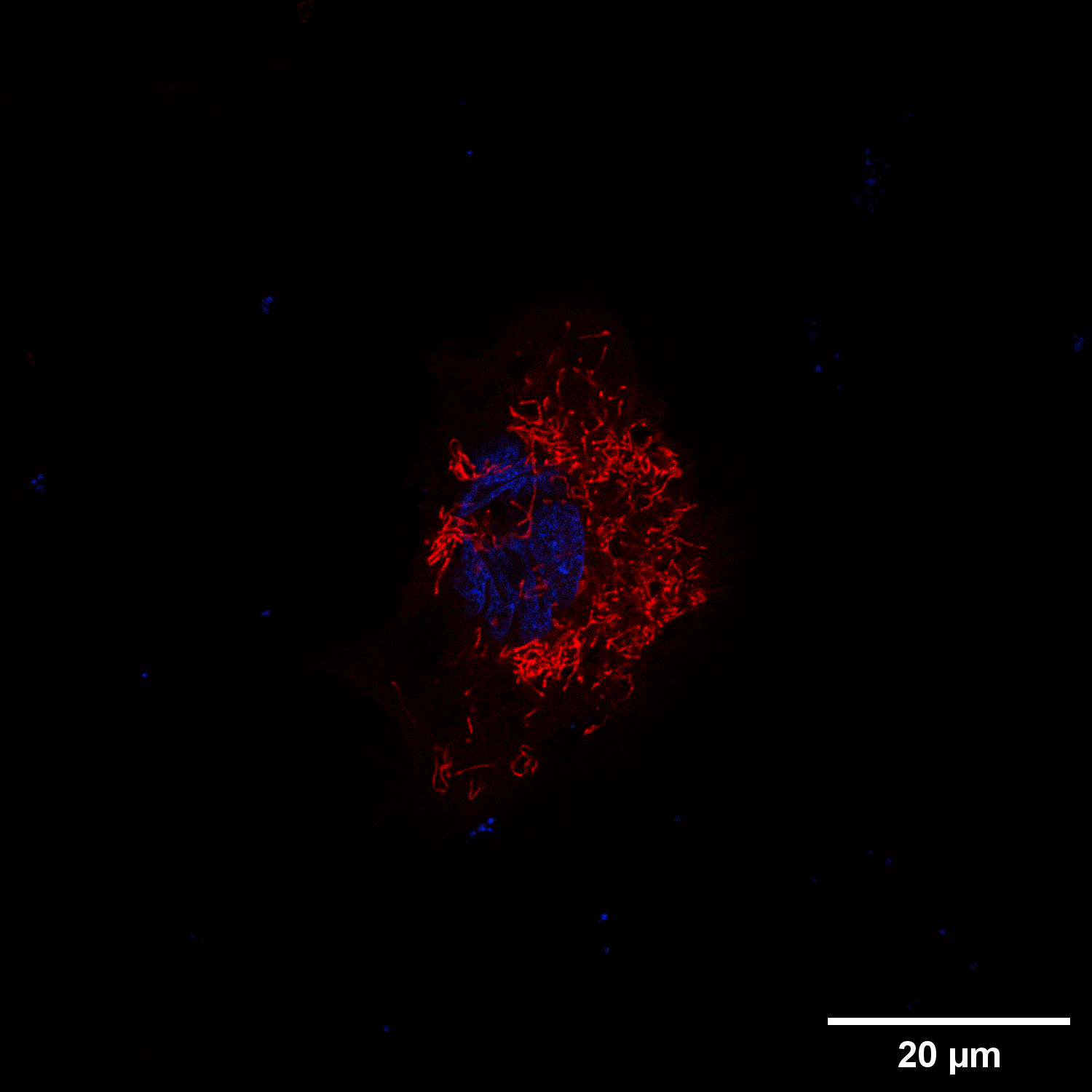

Supplement: Supplementary file 3 [file DataSheet4.zip › Mitotracker(1,2)/Mitotracker-1/Mitotracker-1═╝╞1⁄4/Control╫Θ/Con-1/1 RGB.tif]

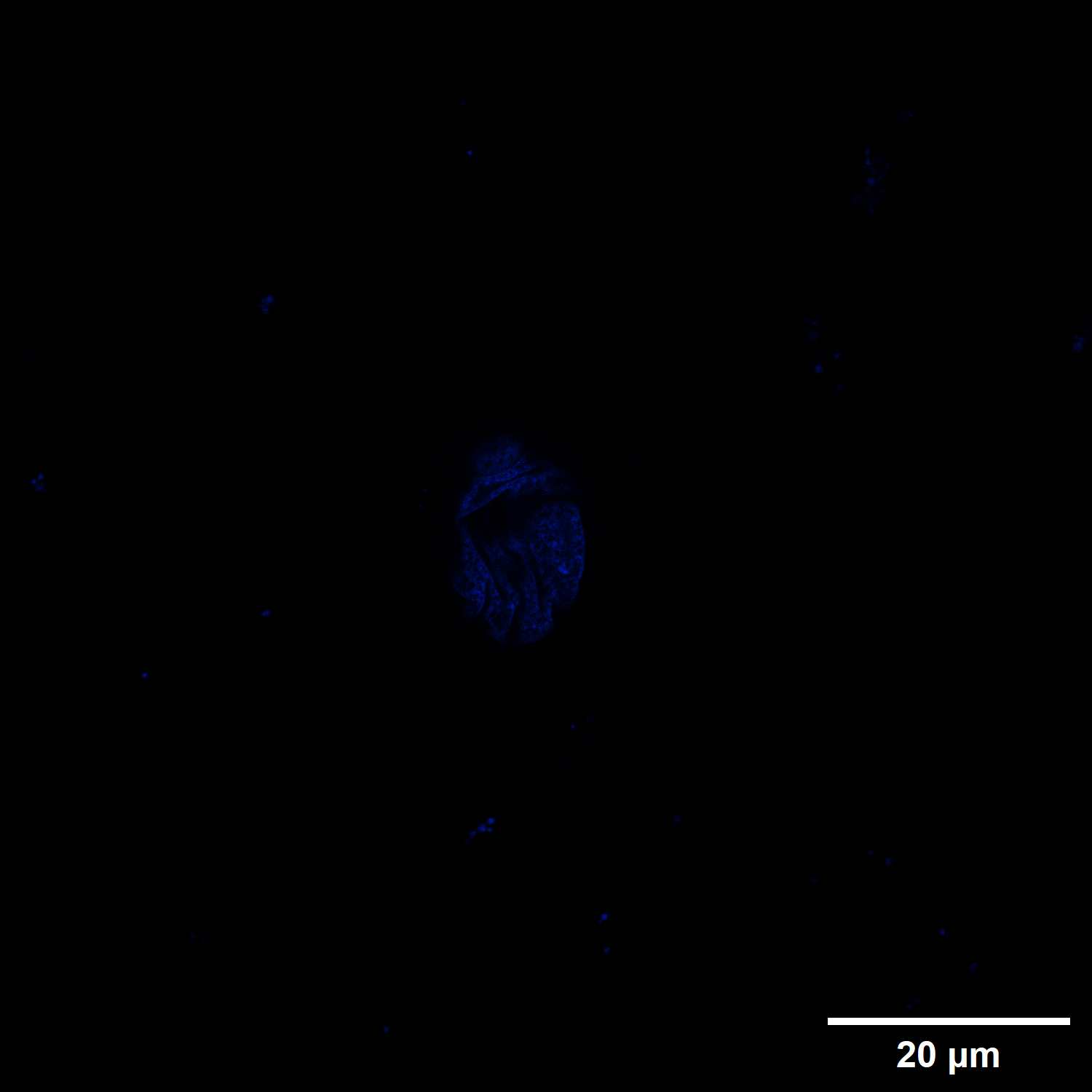

Supplement: Supplementary file 3 [file DataSheet4.zip › Mitotracker(1,2)/Mitotracker-1/Mitotracker-1═╝╞1⁄4/Control╫Θ/Con-1/1 RGB_SR405.tif]

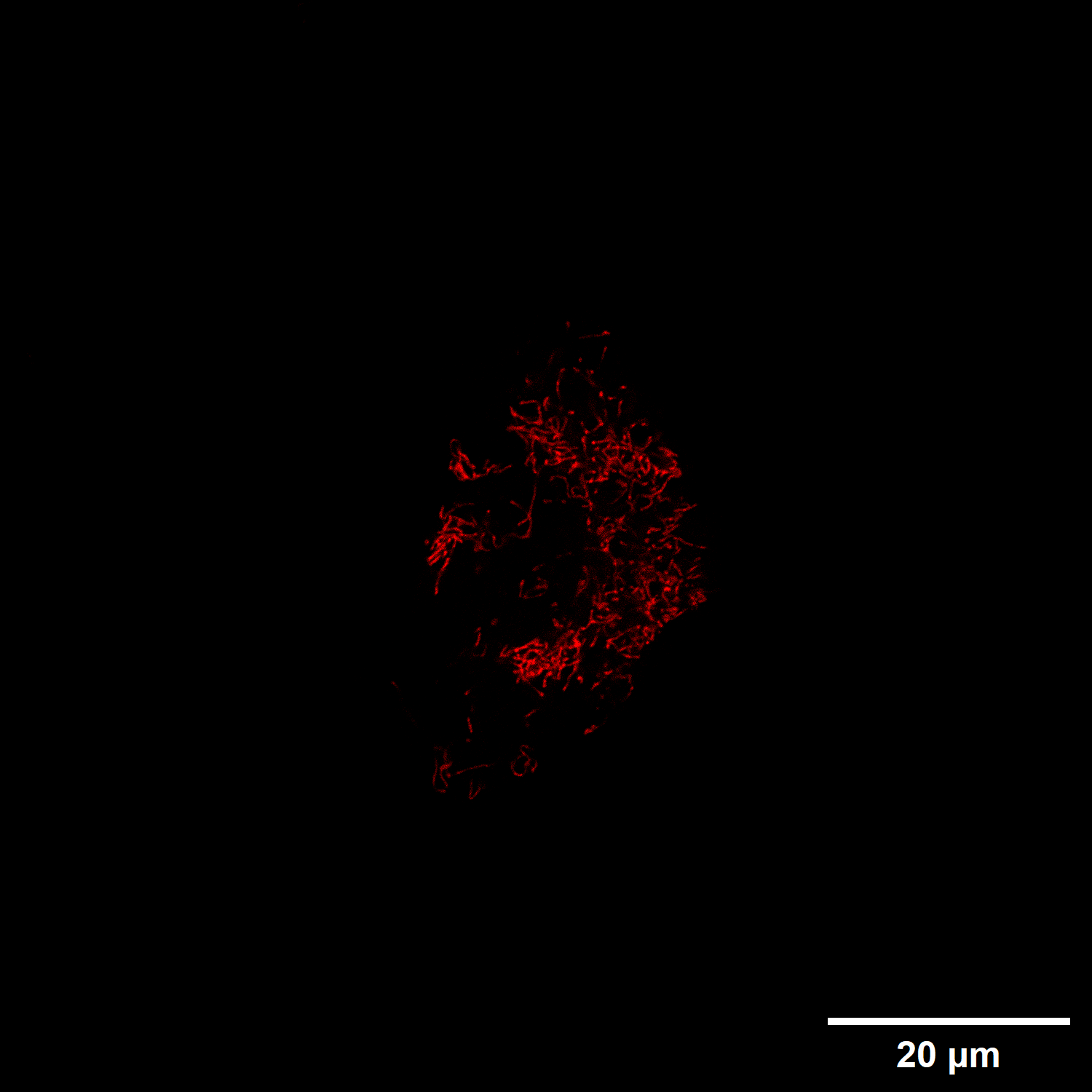

Supplement: Supplementary file 3 [file DataSheet4.zip › Mitotracker(1,2)/Mitotracker-1/Mitotracker-1═╝╞1⁄4/Control╫Θ/Con-1/1 RGB_SR561.tif]

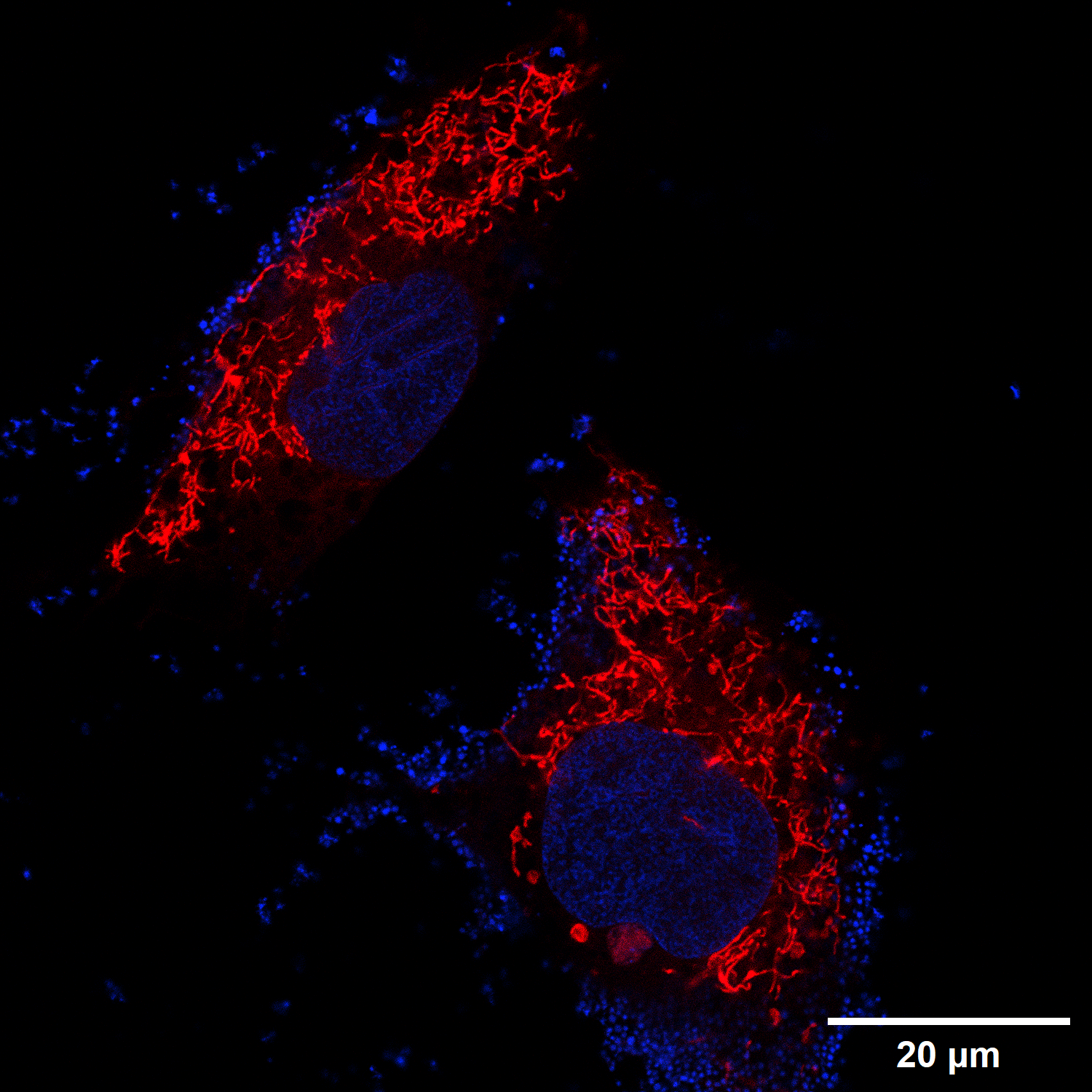

Supplement: Supplementary file 3 [file DataSheet4.zip › Mitotracker(1,2)/Mitotracker-1/Mitotracker-1═╝╞1⁄4/Control╫Θ/Con-2/2_RGB.tif]

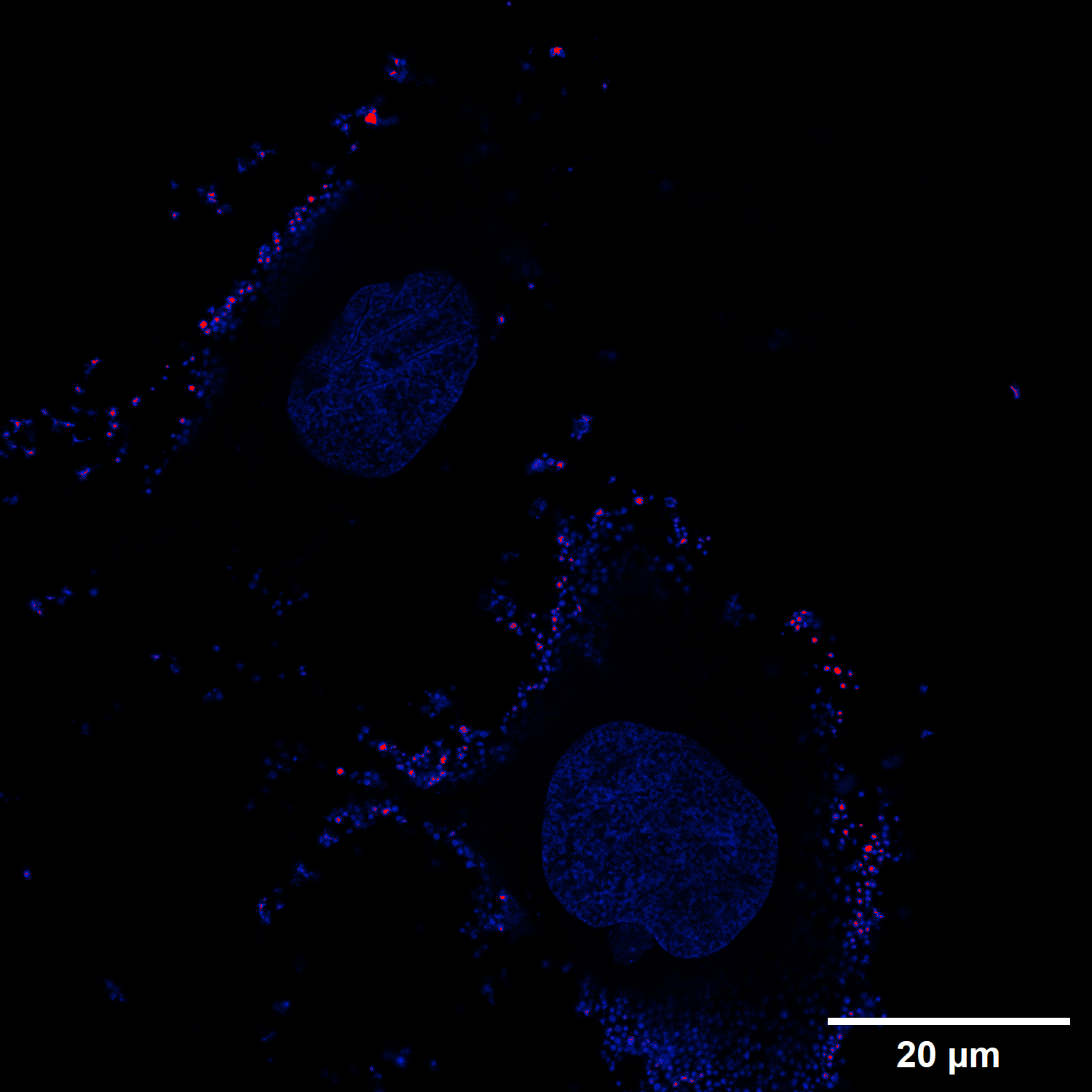

Supplement: Supplementary file 3 [file DataSheet4.zip › Mitotracker(1,2)/Mitotracker-1/Mitotracker-1═╝╞1⁄4/Control╫Θ/Con-2/2_RGB_SR405.tif]

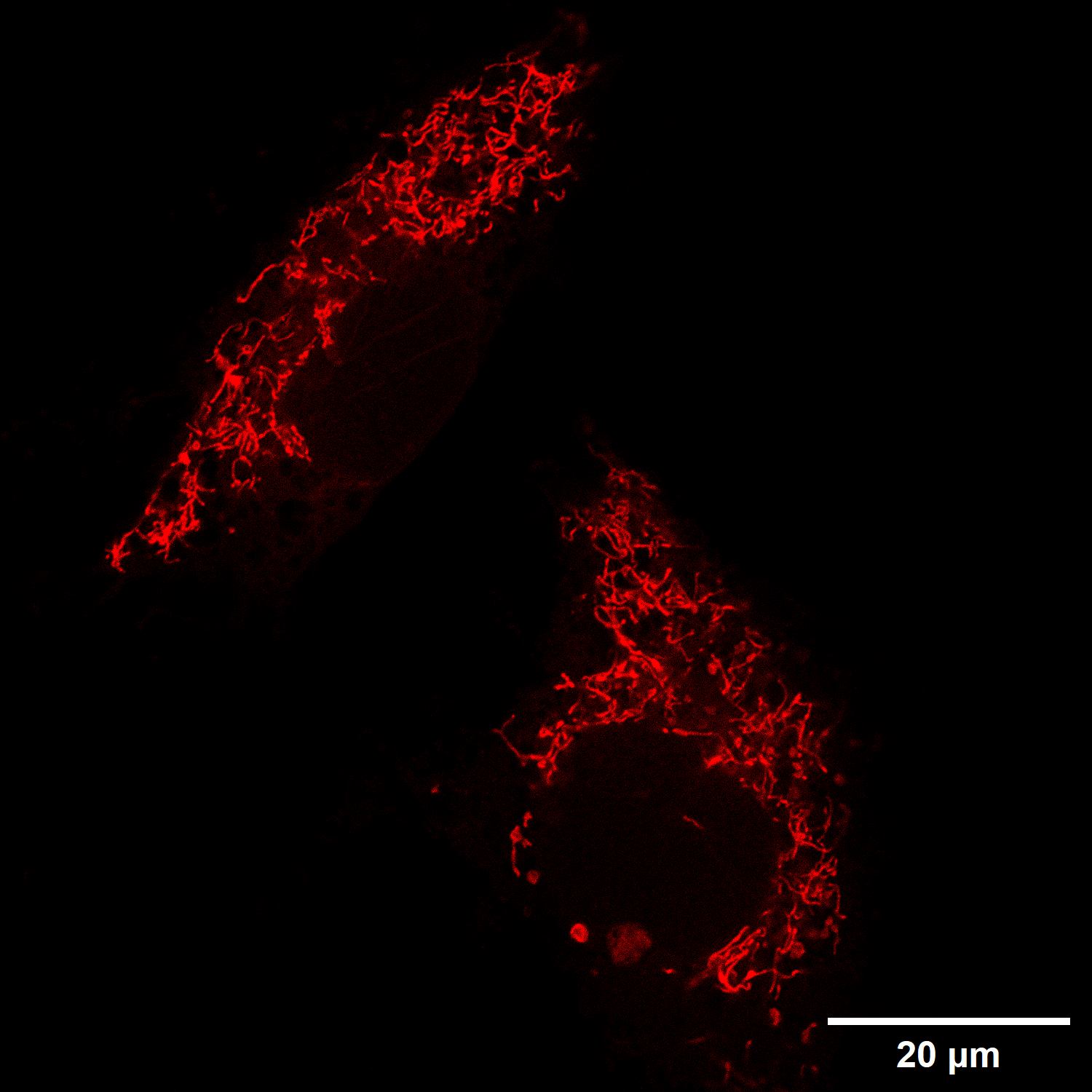

Supplement: Supplementary file 3 [file DataSheet4.zip › Mitotracker(1,2)/Mitotracker-1/Mitotracker-1═╝╞1⁄4/Control╫Θ/Con-2/2_RGB_SR561.tif]

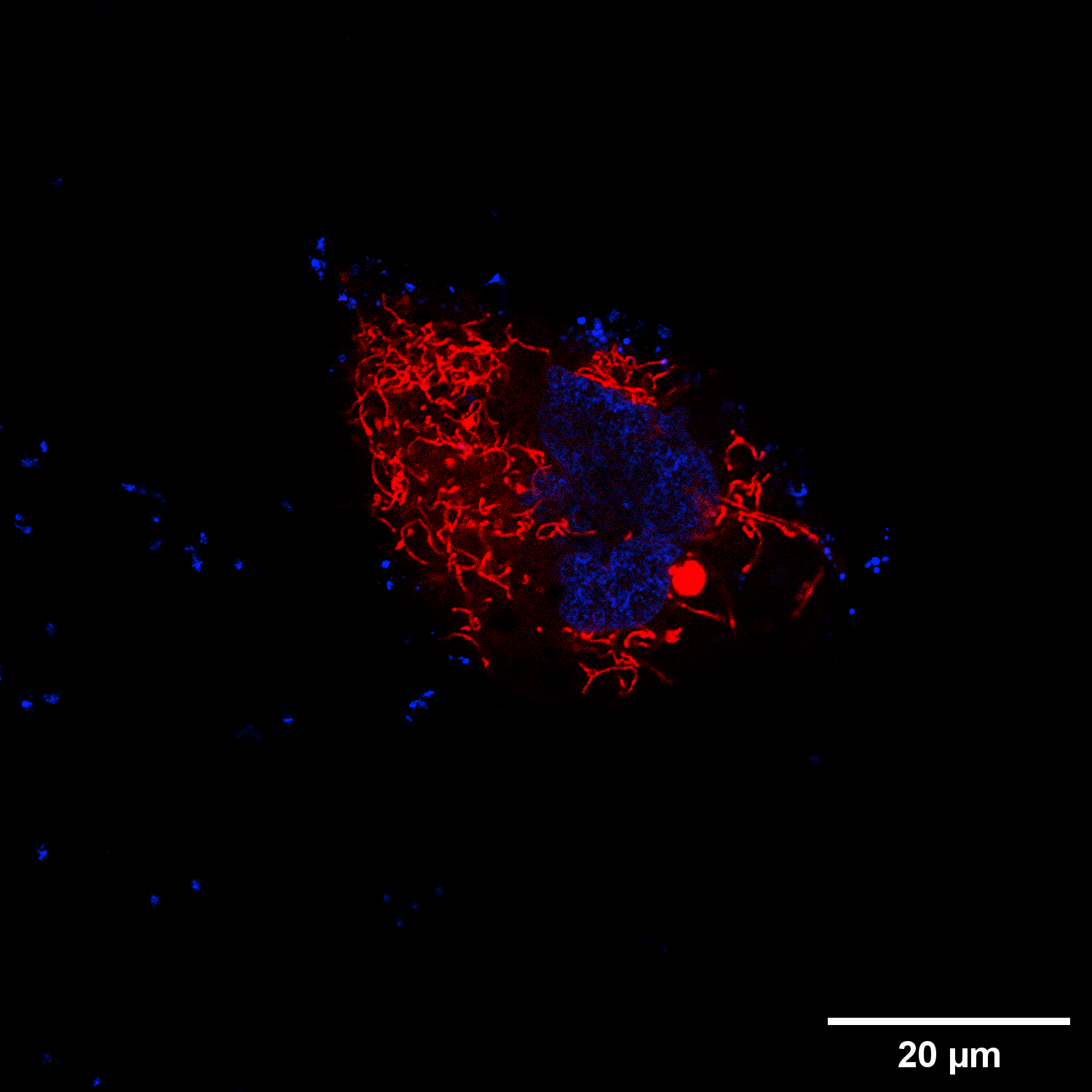

Supplement: Supplementary file 3 [file DataSheet4.zip › Mitotracker(1,2)/Mitotracker-1/Mitotracker-1═╝╞1⁄4/Control╫Θ/Con-3/3_RGB.tif]

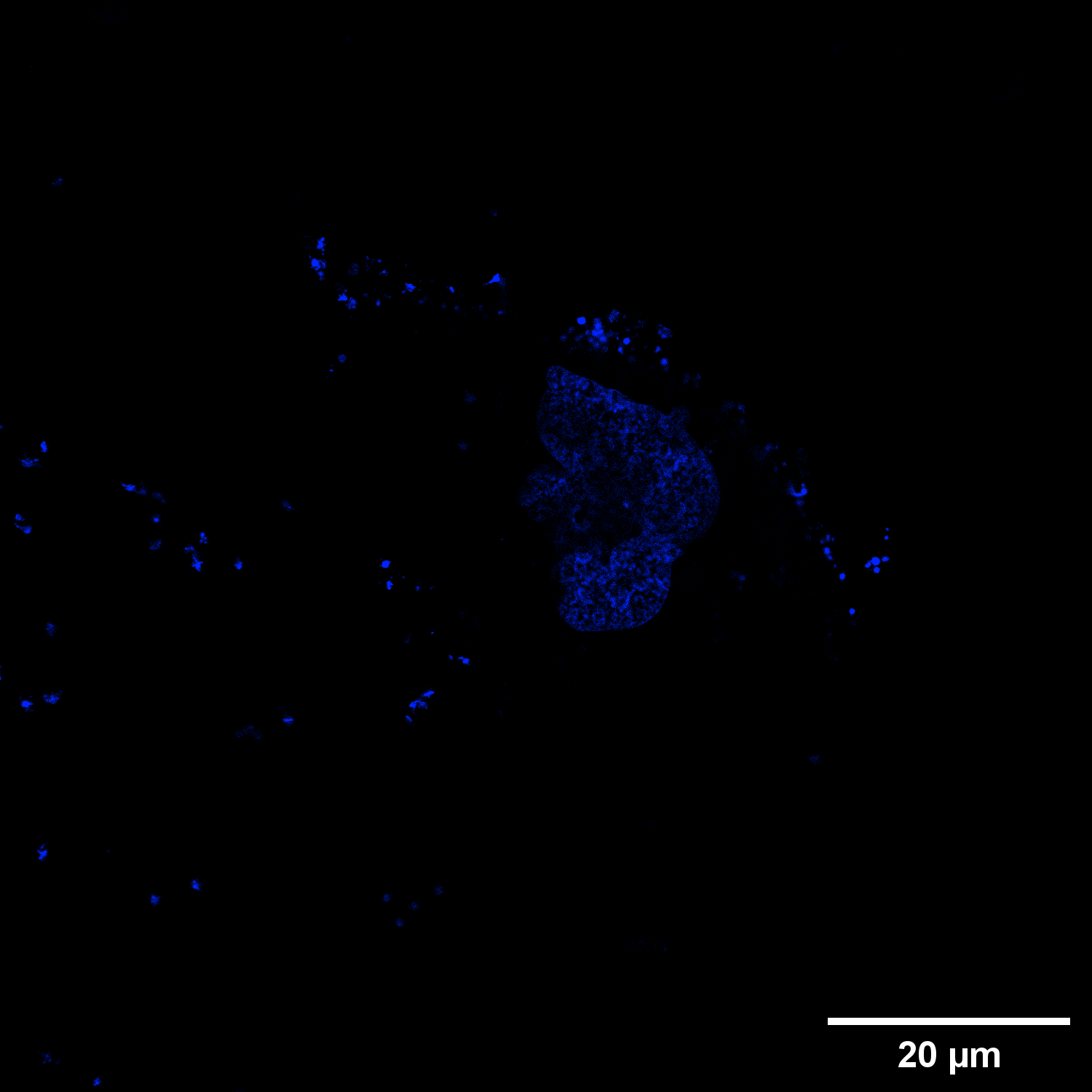

Supplement: Supplementary file 3 [file DataSheet4.zip › Mitotracker(1,2)/Mitotracker-1/Mitotracker-1═╝╞1⁄4/Control╫Θ/Con-3/3_RGB_SR405.tif]

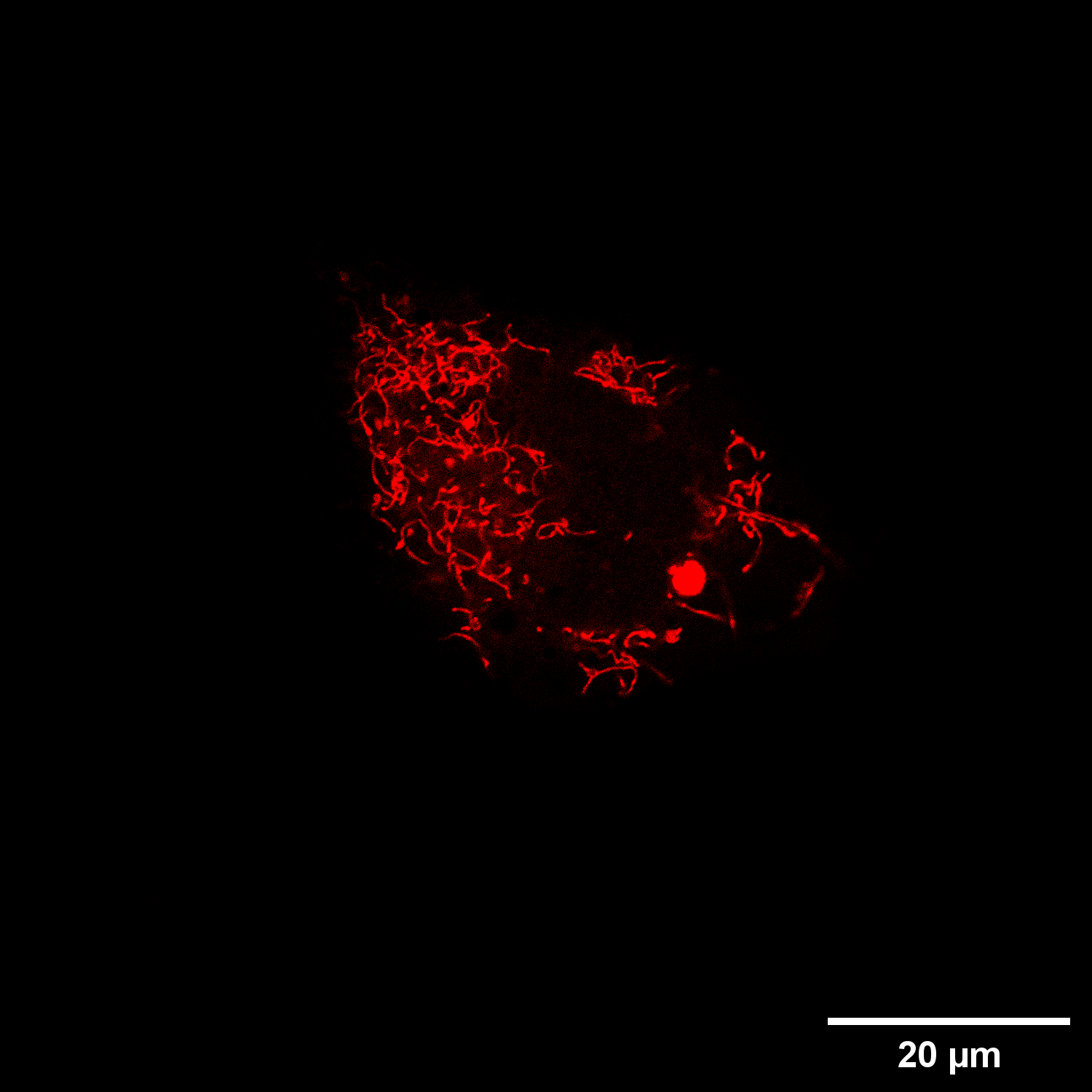

Supplement: Supplementary file 3 [file DataSheet4.zip › Mitotracker(1,2)/Mitotracker-1/Mitotracker-1═╝╞1⁄4/Control╫Θ/Con-3/3_RGB_SR561.tif]

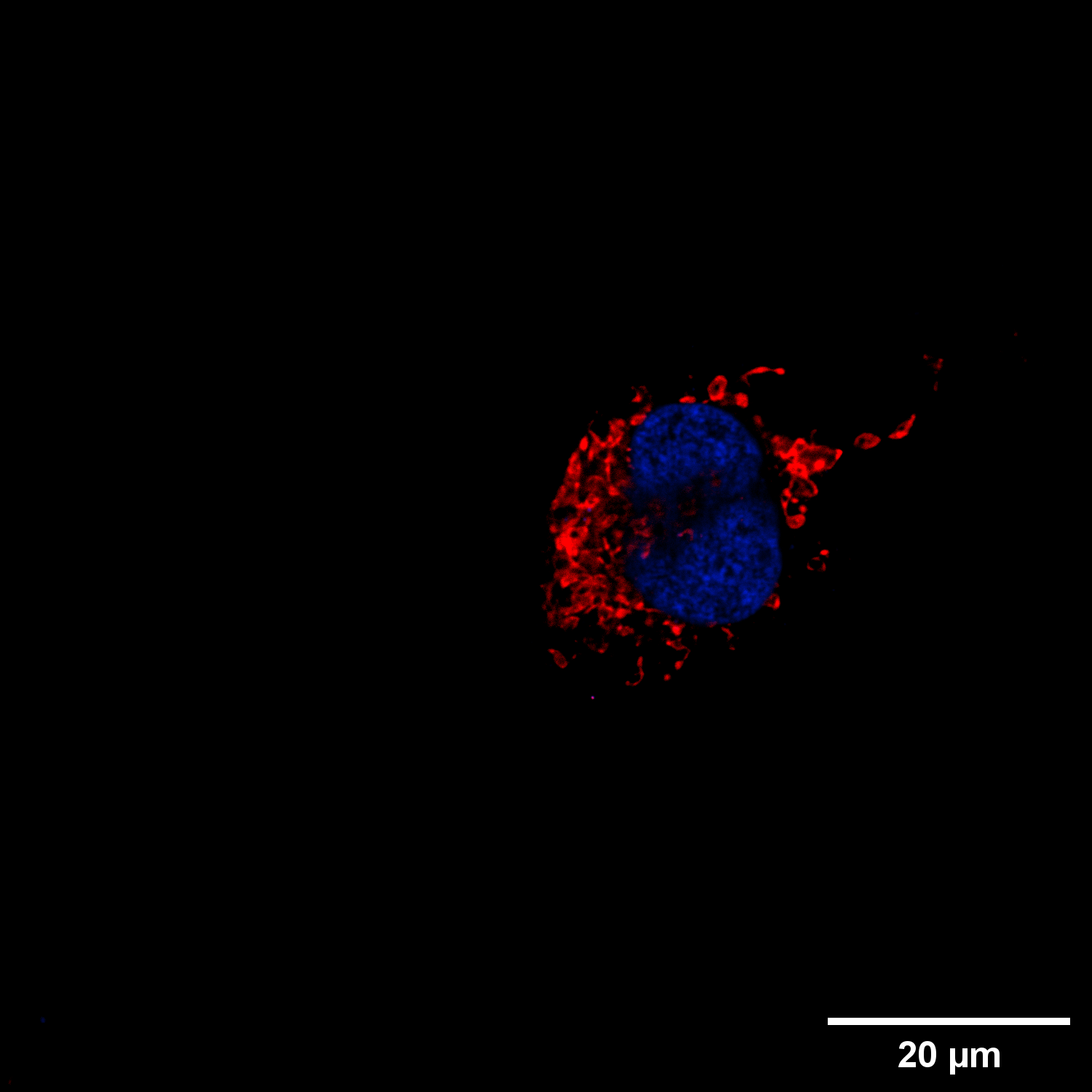

Supplement: Supplementary file 3 [file DataSheet4.zip › Mitotracker(1,2)/Mitotracker-1/Mitotracker-1═╝╞1⁄4/Iohexol 12h/Ioh 12h-1/1_RGB.tif]

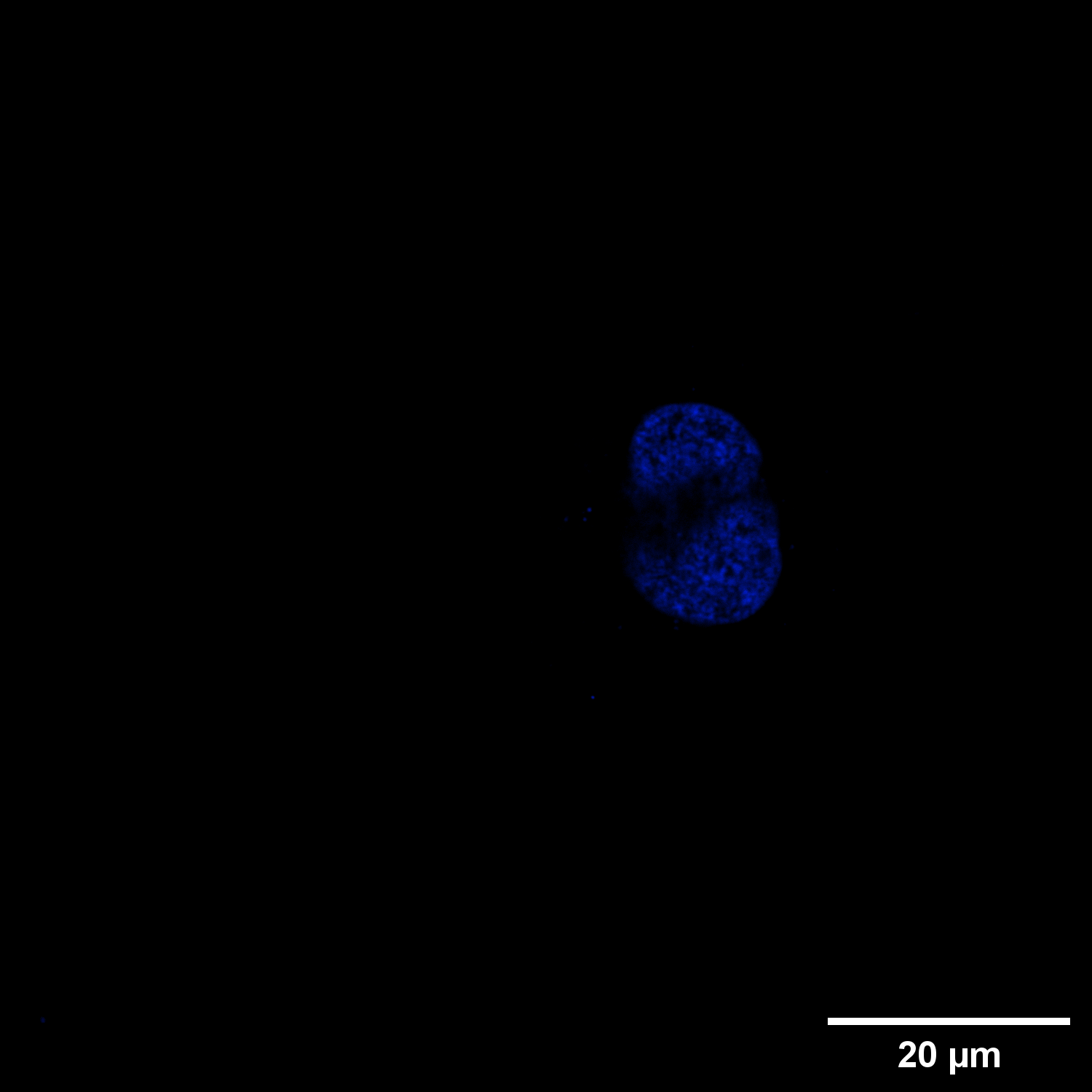

Supplement: Supplementary file 3 [file DataSheet4.zip › Mitotracker(1,2)/Mitotracker-1/Mitotracker-1═╝╞1⁄4/Iohexol 12h/Ioh 12h-1/1_RGB_SR405.tif]

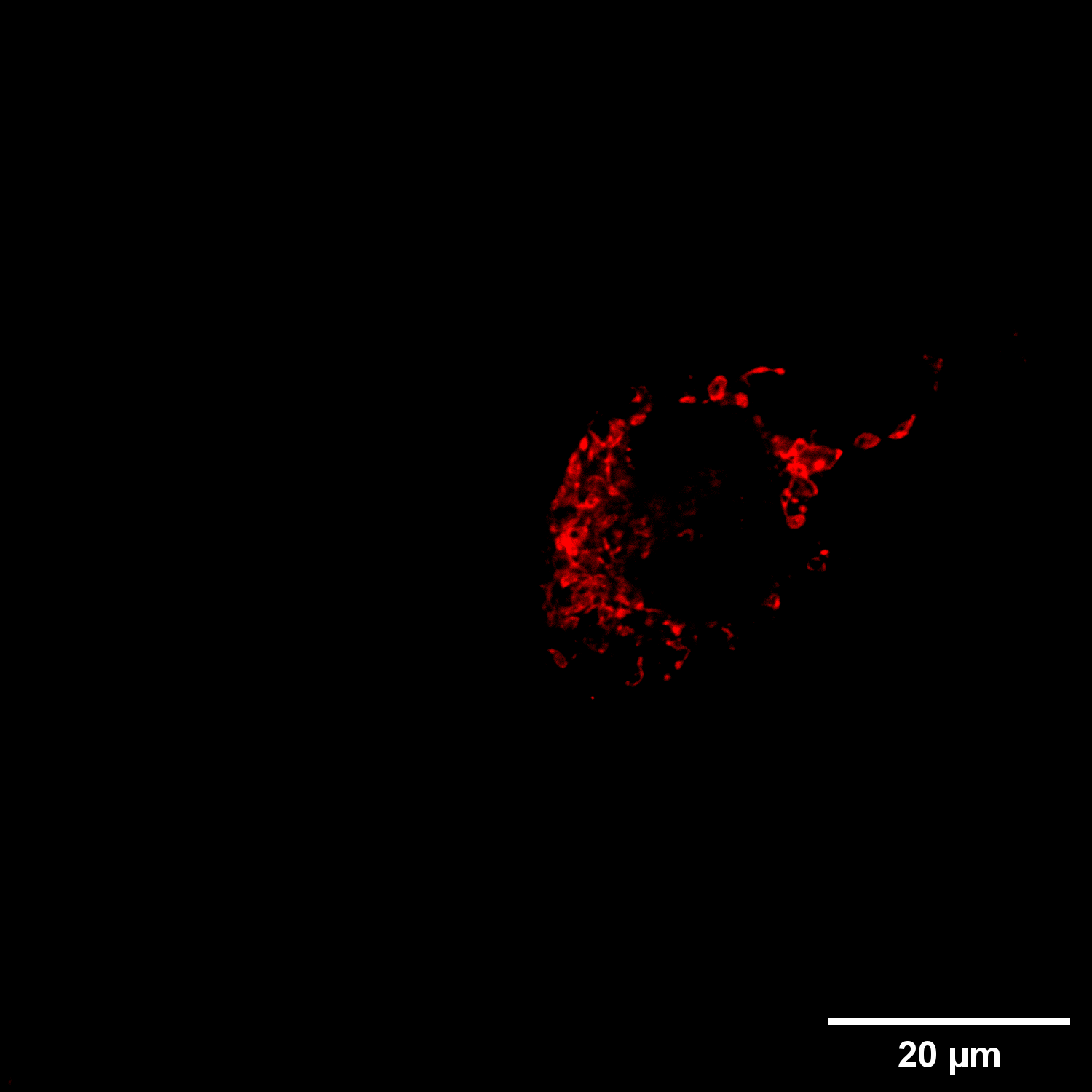

Supplement: Supplementary file 3 [file DataSheet4.zip › Mitotracker(1,2)/Mitotracker-1/Mitotracker-1═╝╞1⁄4/Iohexol 12h/Ioh 12h-1/1_RGB_SR561.tif]

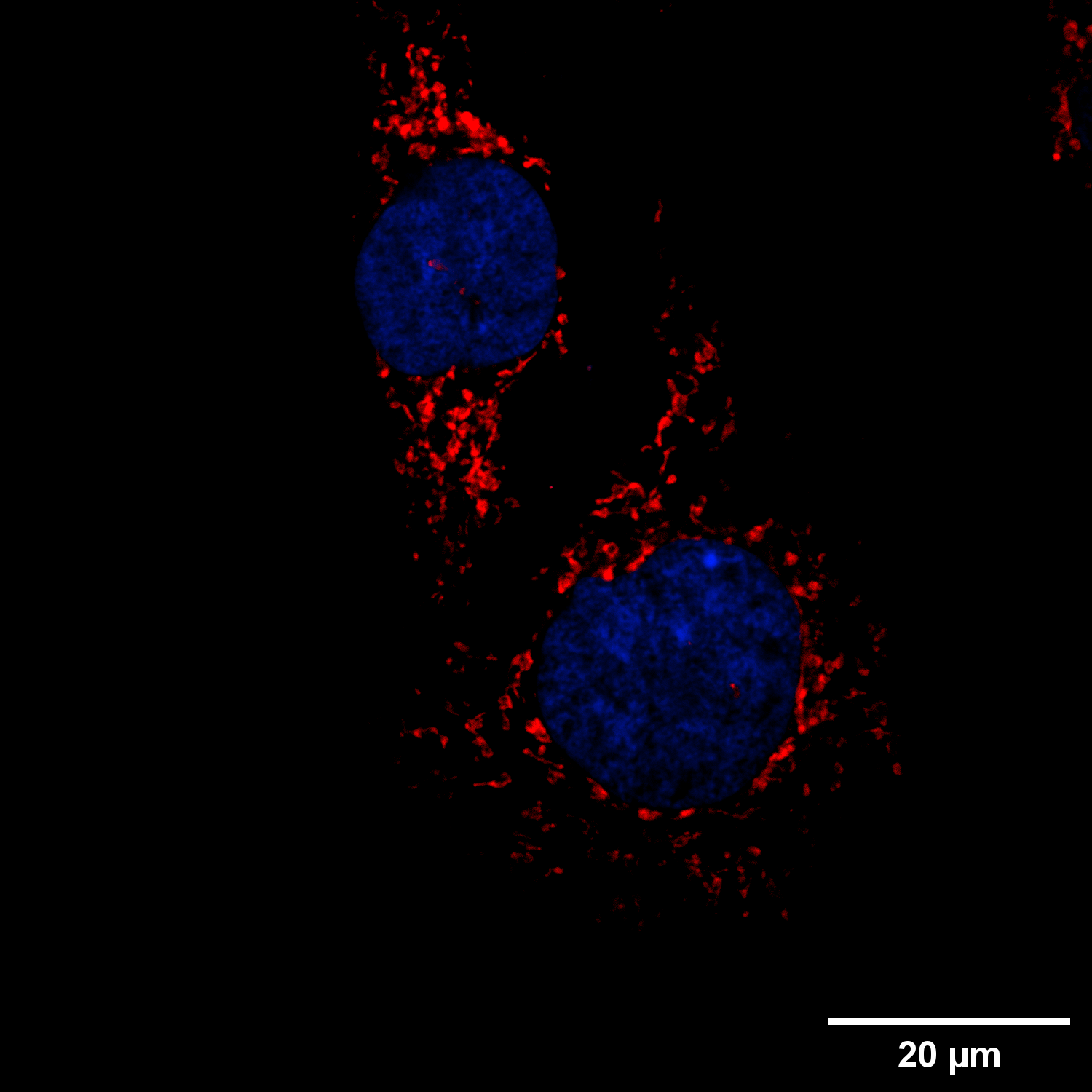

Supplement: Supplementary file 3 [file DataSheet4.zip › Mitotracker(1,2)/Mitotracker-1/Mitotracker-1═╝╞1⁄4/Iohexol 12h/Ioh 12h-2/2_RGB.tif]

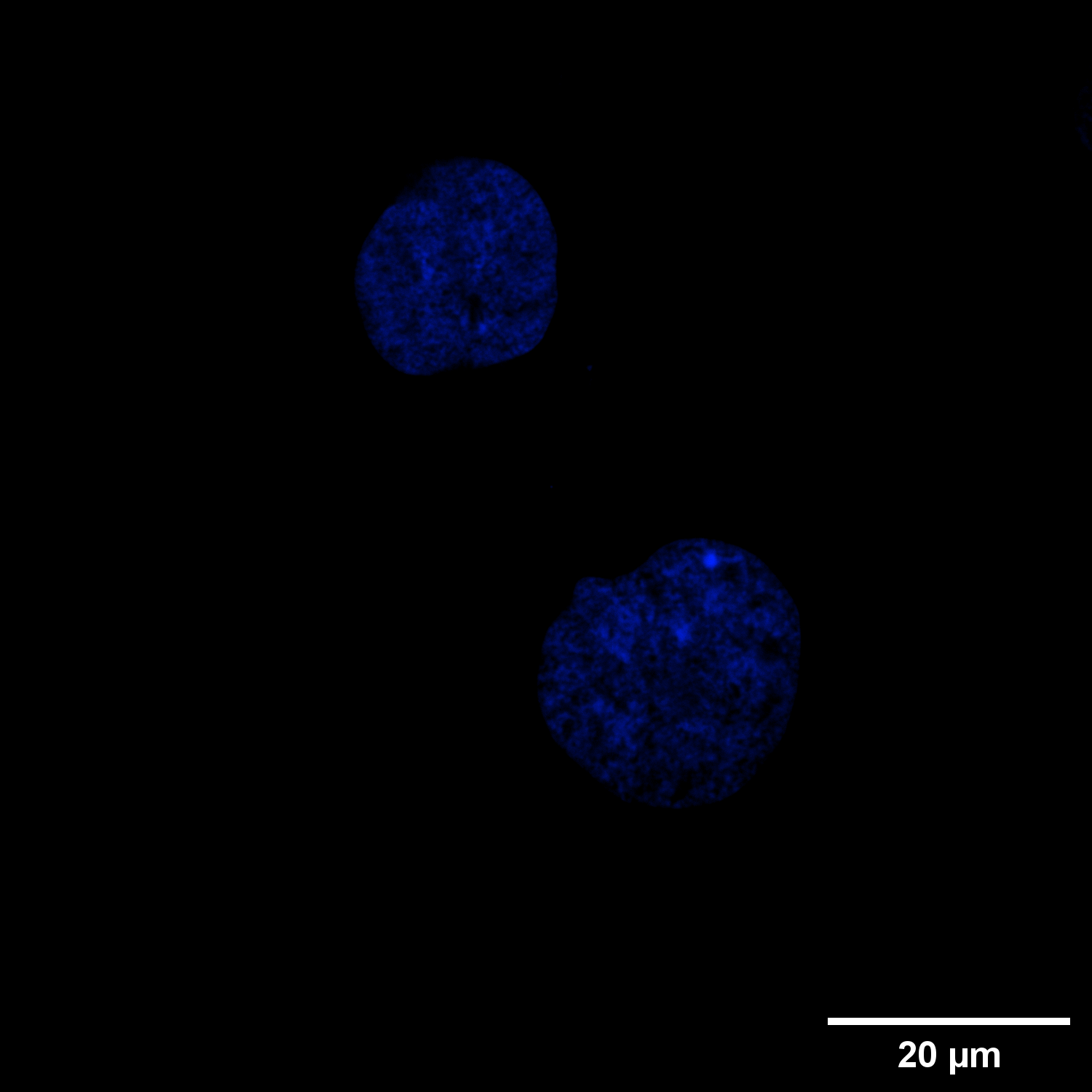

Supplement: Supplementary file 3 [file DataSheet4.zip › Mitotracker(1,2)/Mitotracker-1/Mitotracker-1═╝╞1⁄4/Iohexol 12h/Ioh 12h-2/2_RGB_SR405.tif]

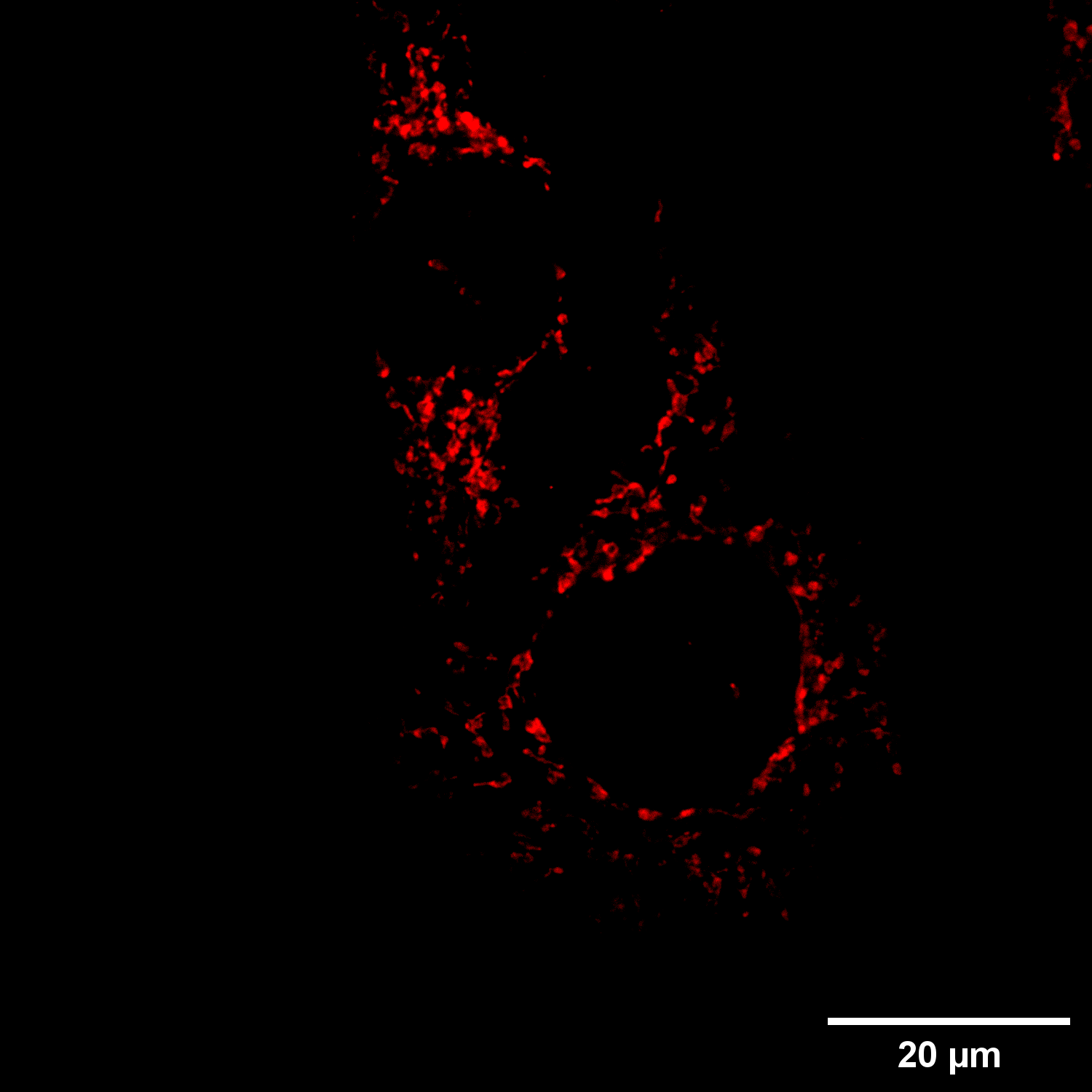

Supplement: Supplementary file 3 [file DataSheet4.zip › Mitotracker(1,2)/Mitotracker-1/Mitotracker-1═╝╞1⁄4/Iohexol 12h/Ioh 12h-2/2_RGB_SR561.tif]

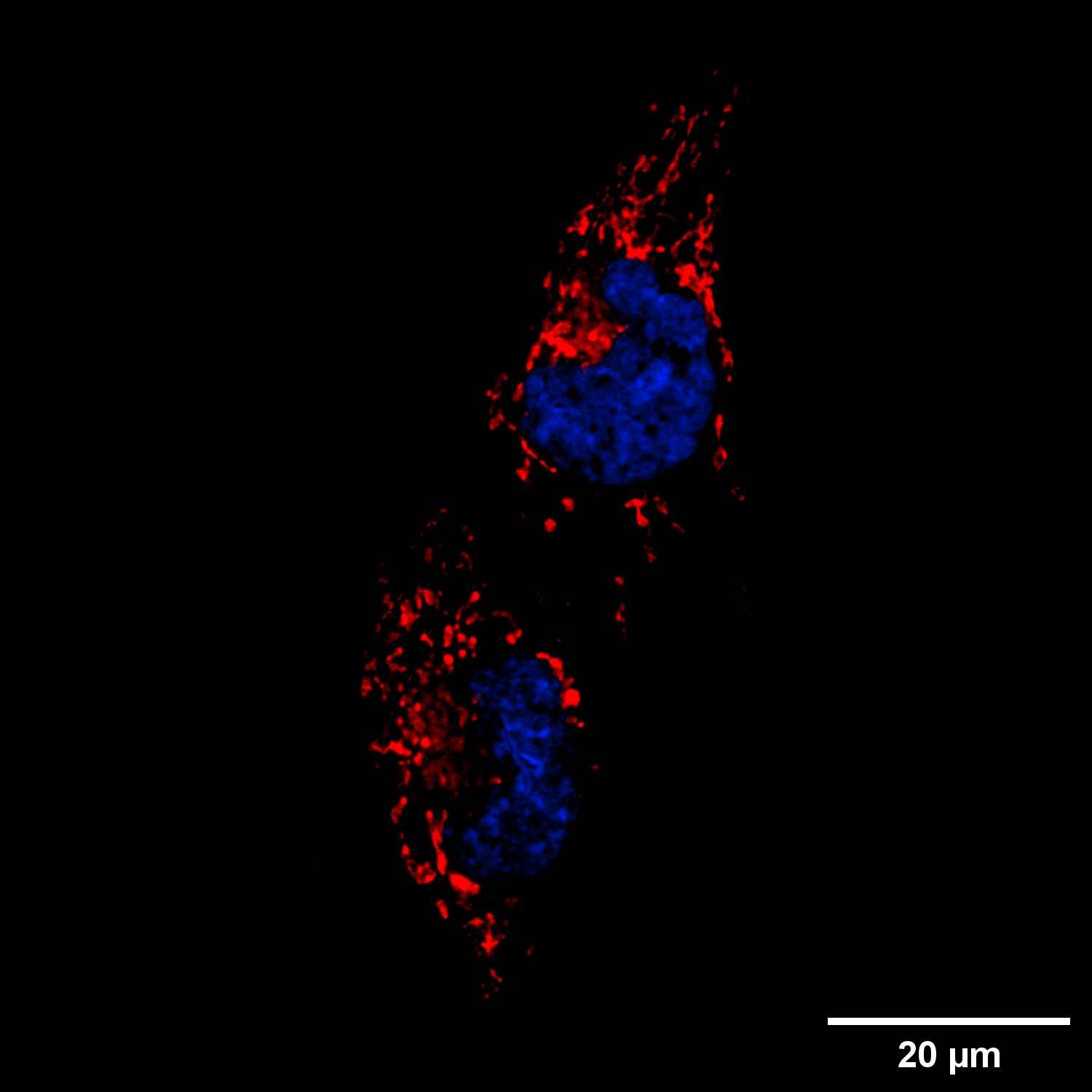

Supplement: Supplementary file 3 [file DataSheet4.zip › Mitotracker(1,2)/Mitotracker-1/Mitotracker-1═╝╞1⁄4/Iohexol 12h/Ioh 12h-3/3_RGB.tif]

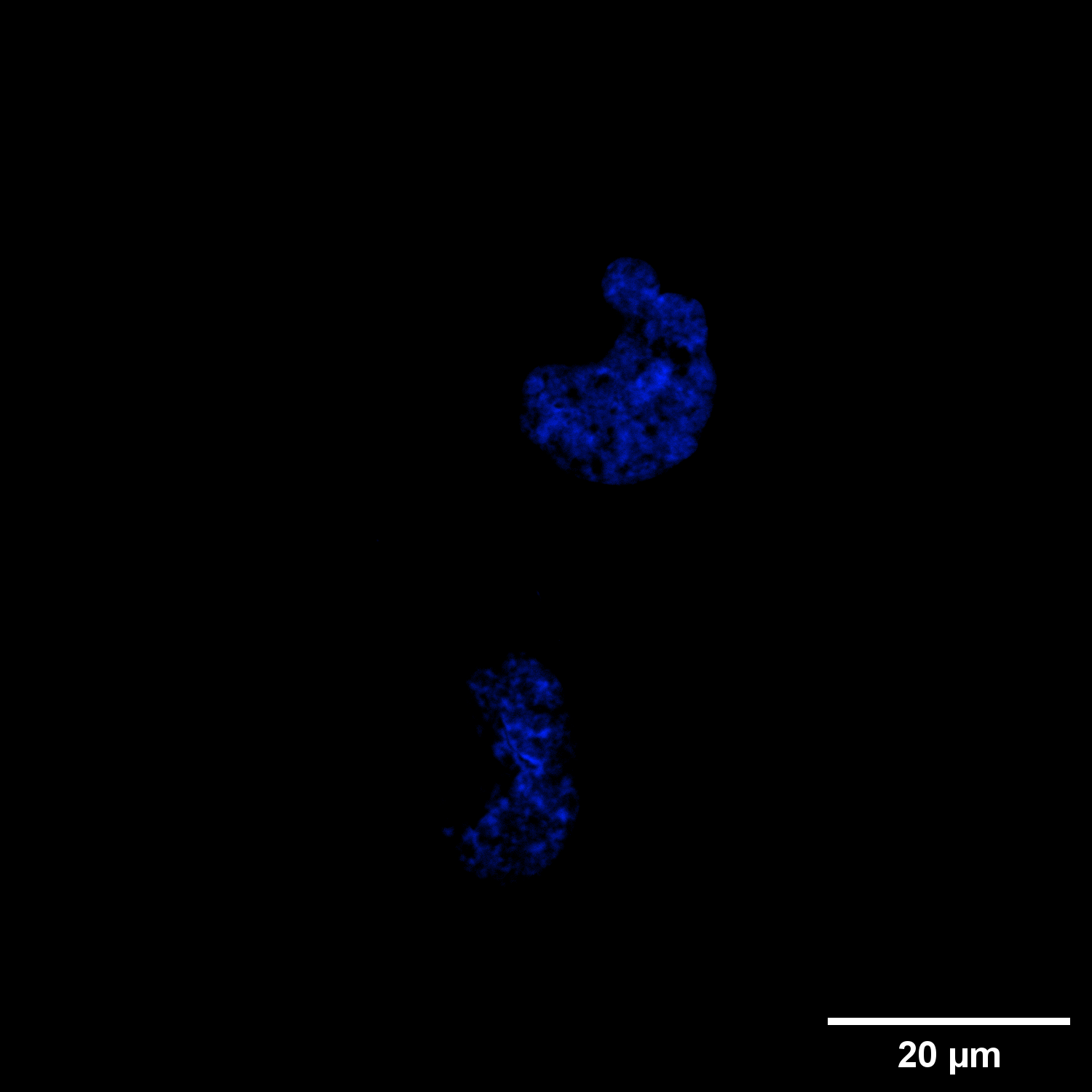

Supplement: Supplementary file 3 [file DataSheet4.zip › Mitotracker(1,2)/Mitotracker-1/Mitotracker-1═╝╞1⁄4/Iohexol 12h/Ioh 12h-3/3_RGB_SR405.tif]

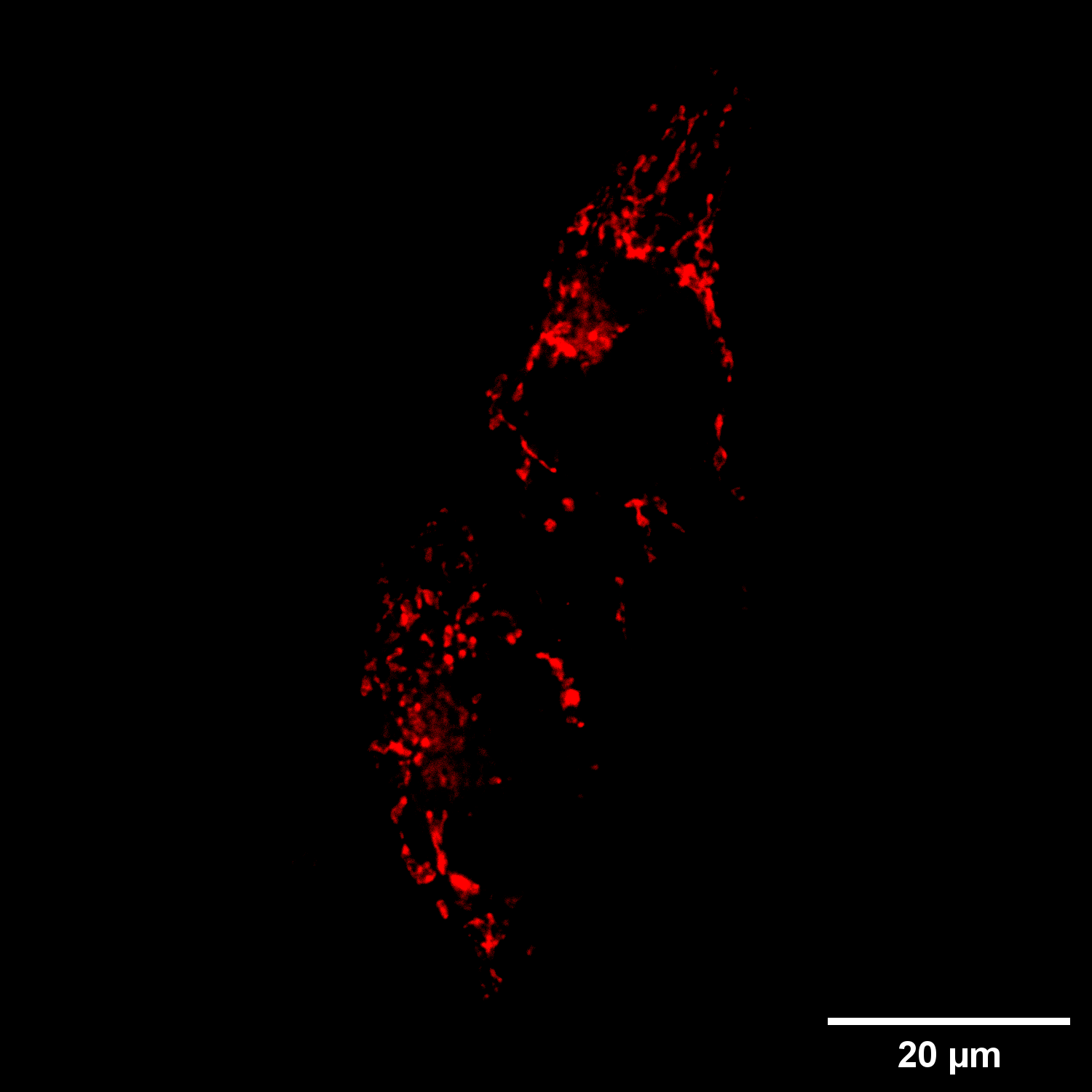

Supplement: Supplementary file 3 [file DataSheet4.zip › Mitotracker(1,2)/Mitotracker-1/Mitotracker-1═╝╞1⁄4/Iohexol 12h/Ioh 12h-3/3_RGB_SR561.tif]

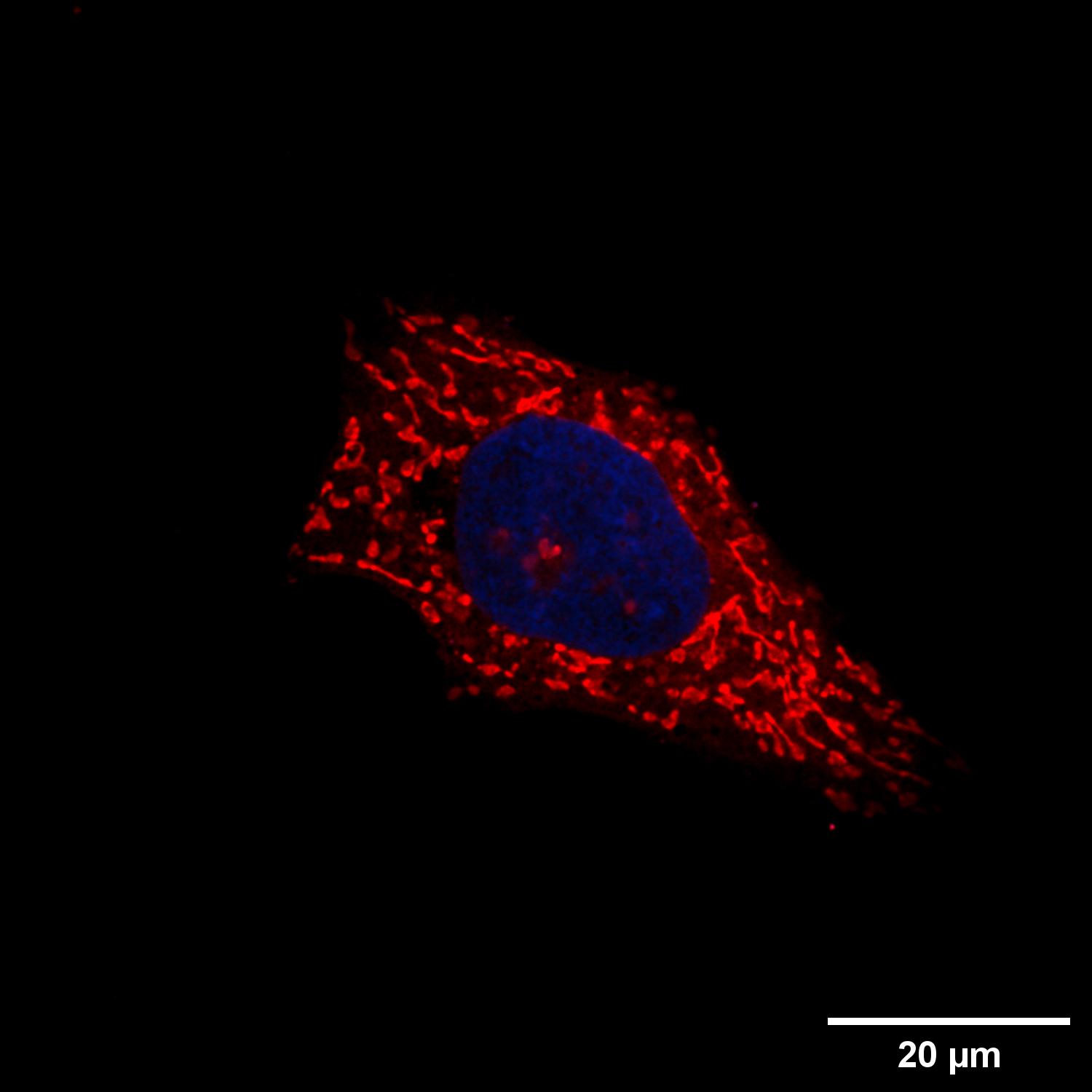

Supplement: Supplementary file 3 [file DataSheet4.zip › Mitotracker(1,2)/Mitotracker-1/Mitotracker-1═╝╞1⁄4/Iohexol 4h/Ioh 4h-1/1 RGB.tif]

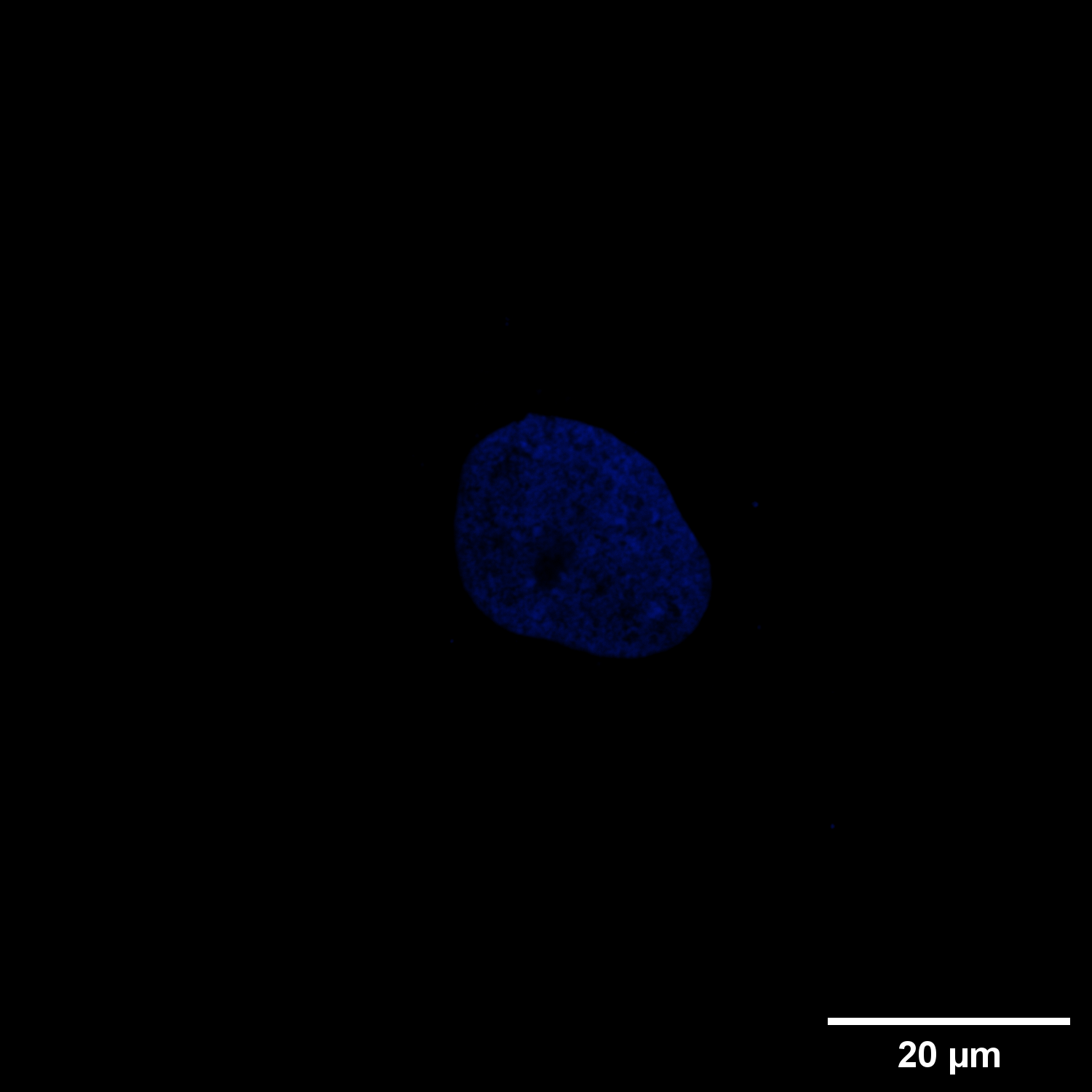

Supplement: Supplementary file 3 [file DataSheet4.zip › Mitotracker(1,2)/Mitotracker-1/Mitotracker-1═╝╞1⁄4/Iohexol 4h/Ioh 4h-1/1 RGB_SR405.tif]

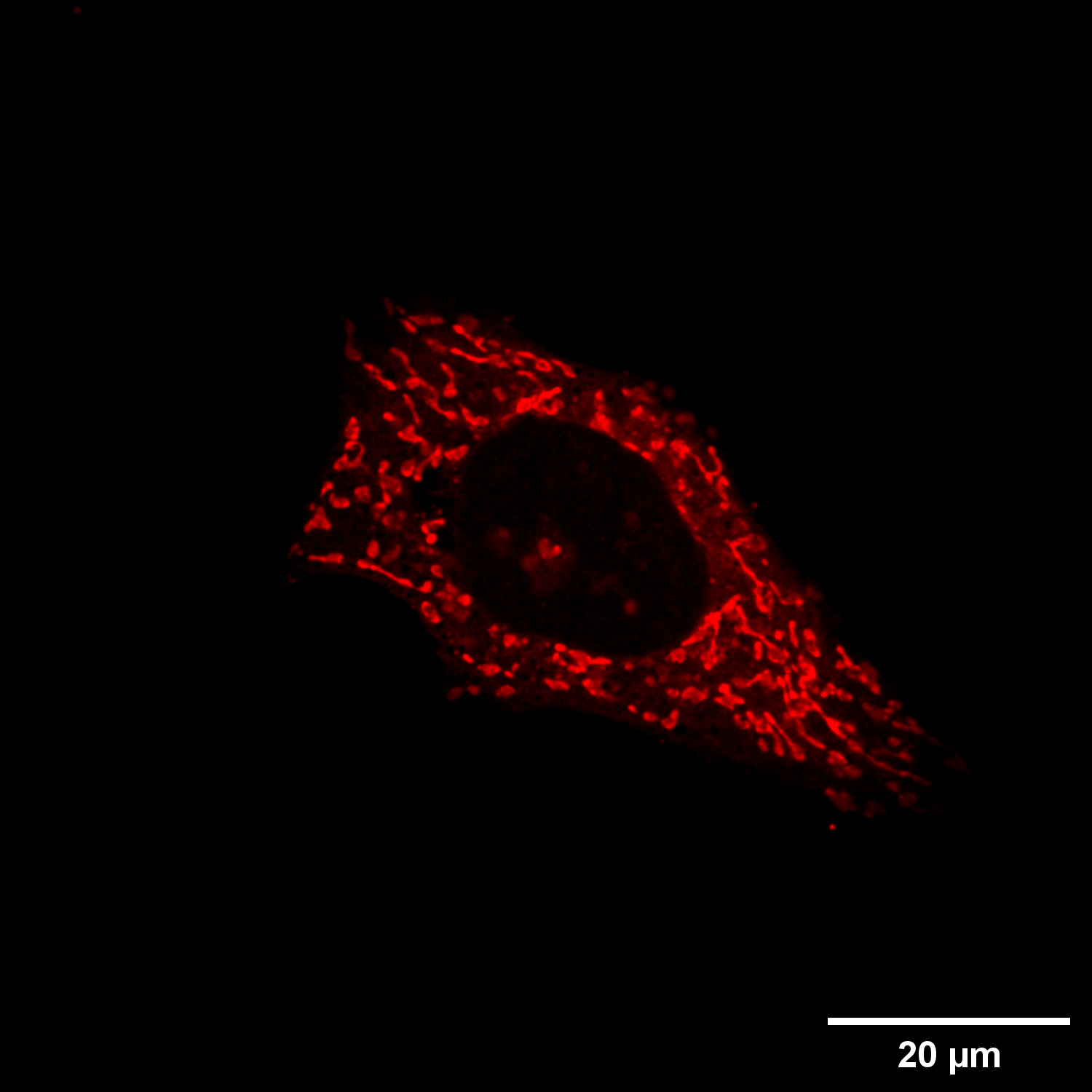

Supplement: Supplementary file 3 [file DataSheet4.zip › Mitotracker(1,2)/Mitotracker-1/Mitotracker-1═╝╞1⁄4/Iohexol 4h/Ioh 4h-1/1 RGB_SR561.tif]

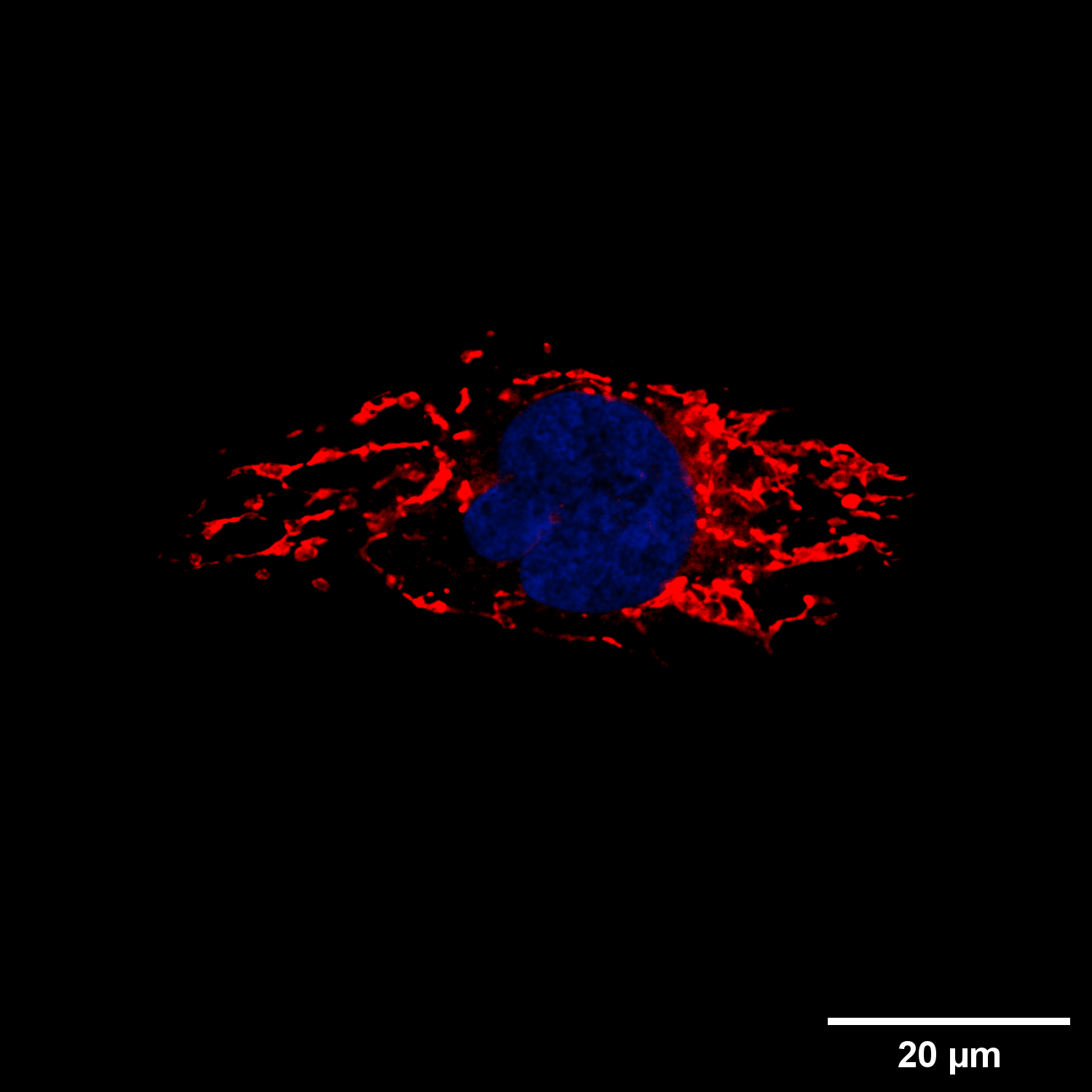

Supplement: Supplementary file 3 [file DataSheet4.zip › Mitotracker(1,2)/Mitotracker-1/Mitotracker-1═╝╞1⁄4/Iohexol 4h/Ioh 4h-2/2_RGB.tif]

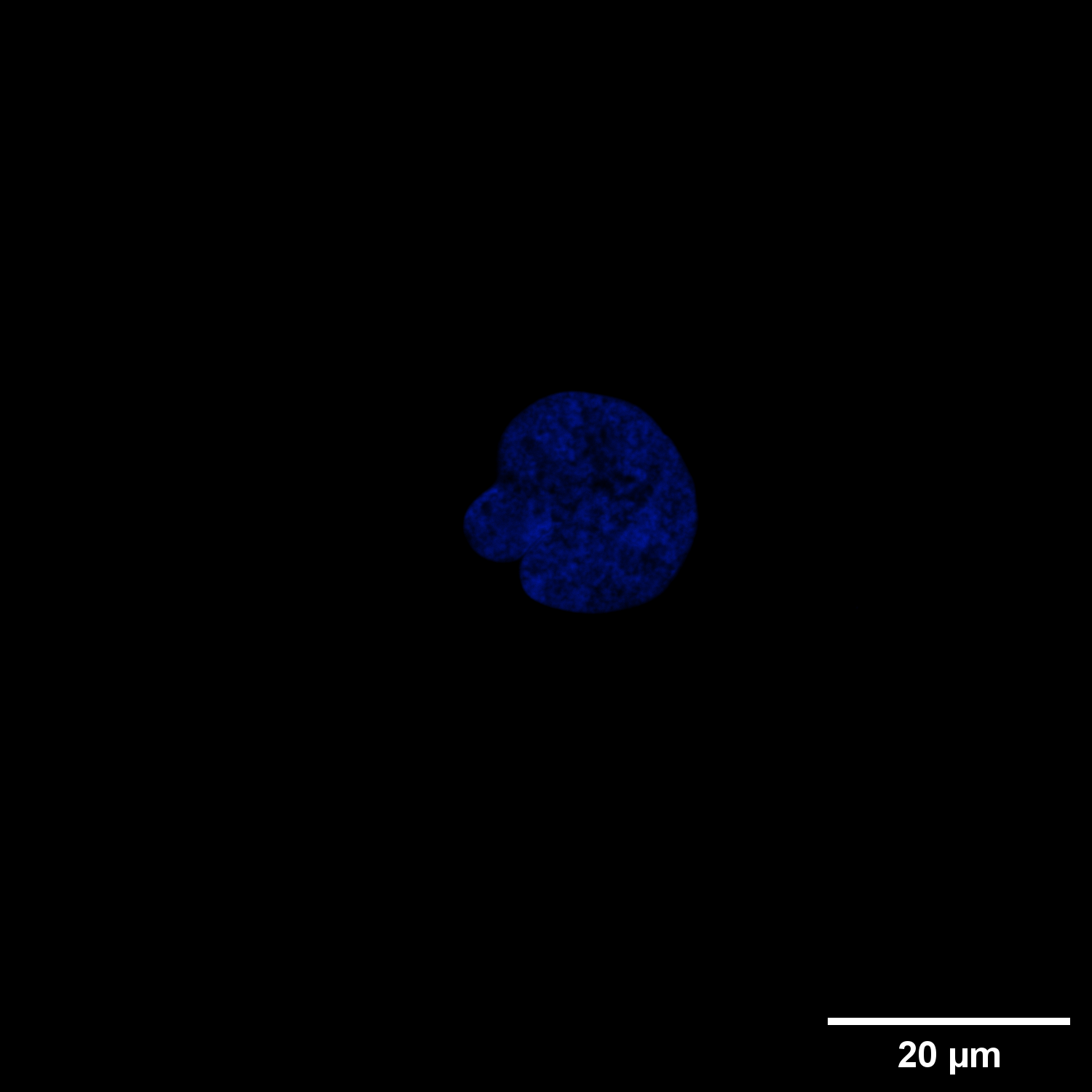

Supplement: Supplementary file 3 [file DataSheet4.zip › Mitotracker(1,2)/Mitotracker-1/Mitotracker-1═╝╞1⁄4/Iohexol 4h/Ioh 4h-2/2_RGB_SR405.tif]

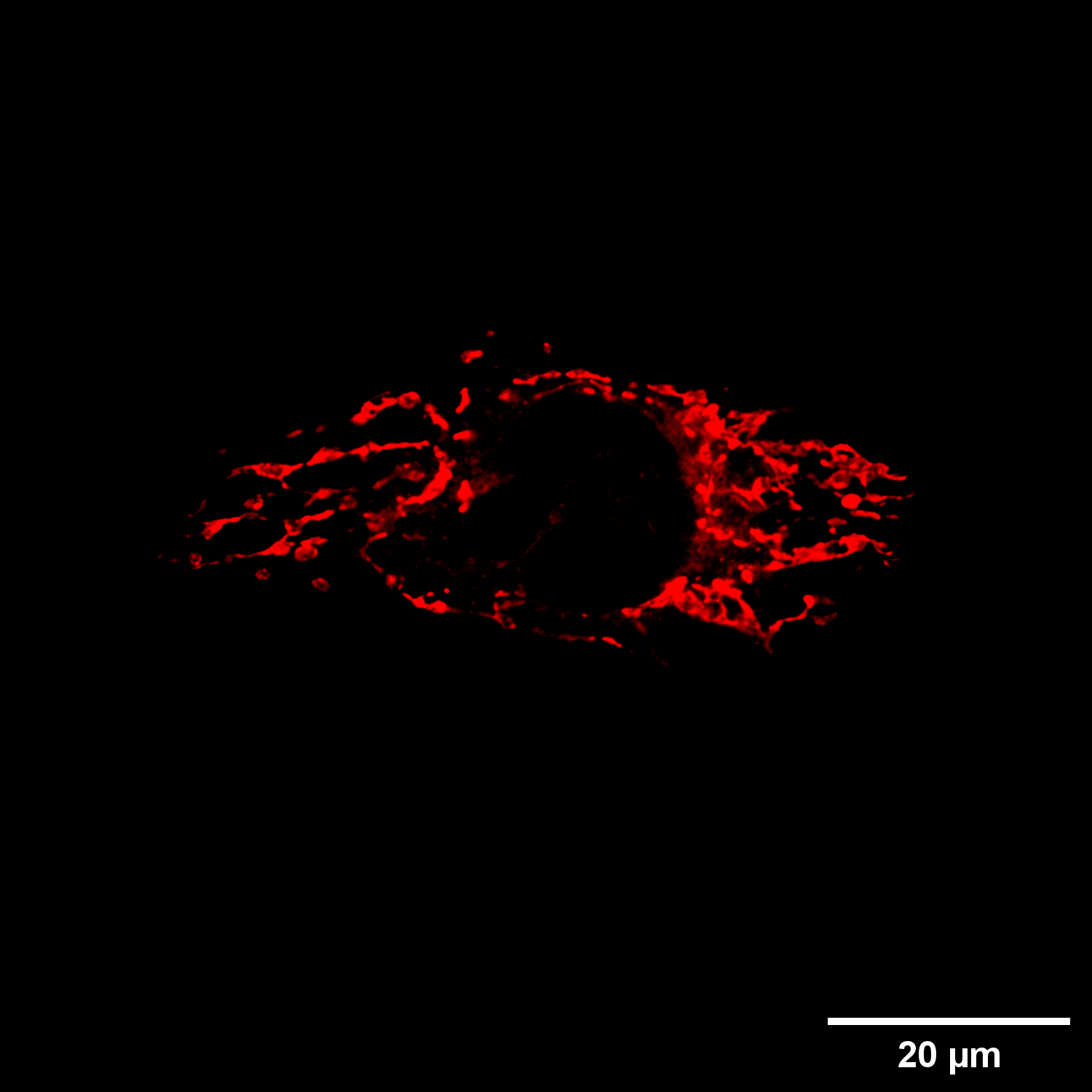

Supplement: Supplementary file 3 [file DataSheet4.zip › Mitotracker(1,2)/Mitotracker-1/Mitotracker-1═╝╞1⁄4/Iohexol 4h/Ioh 4h-2/2_RGB_SR561.tif]

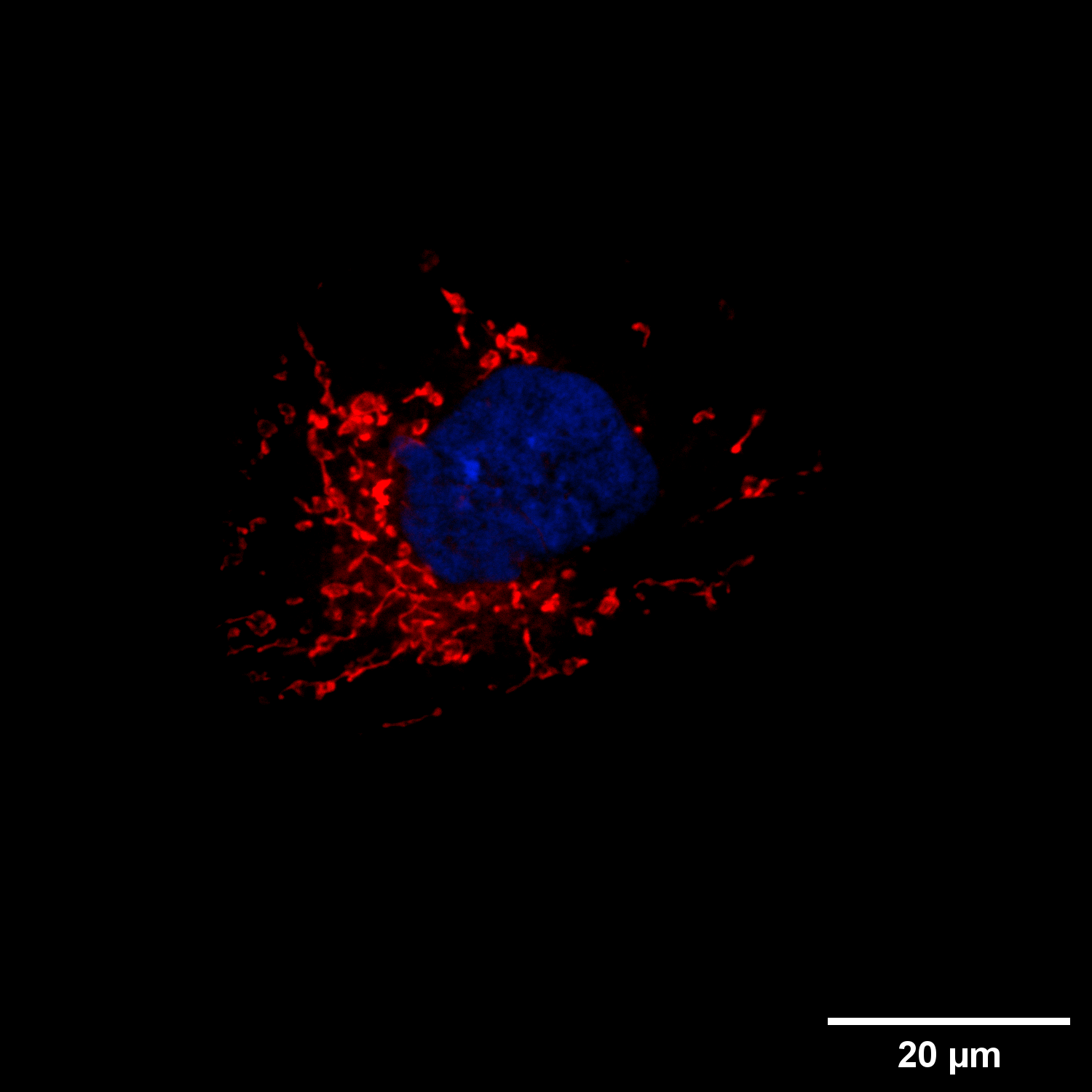

Supplement: Supplementary file 3 [file DataSheet4.zip › Mitotracker(1,2)/Mitotracker-1/Mitotracker-1═╝╞1⁄4/Iohexol 4h/Ioh 4h-3/3_RGB.tif]

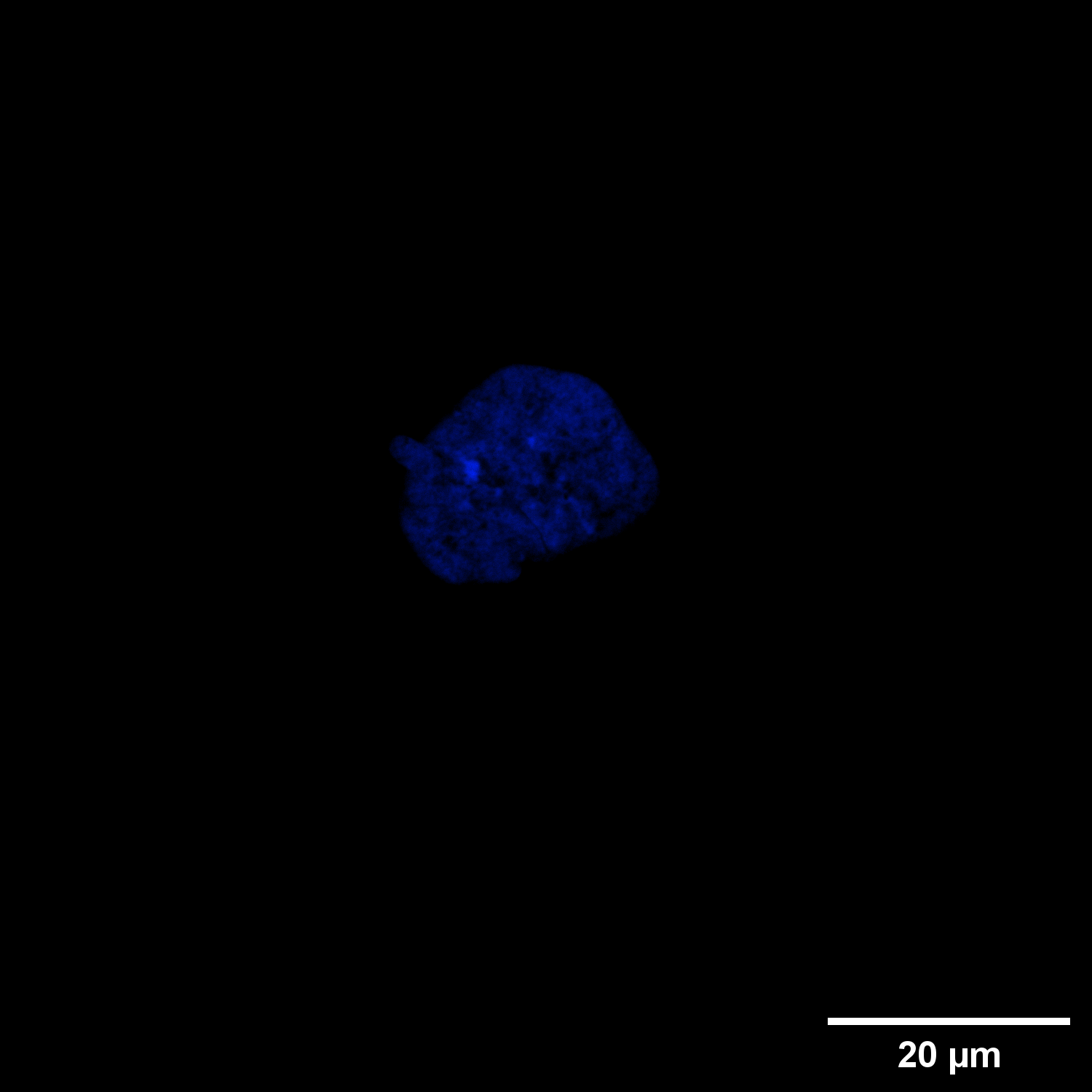

Supplement: Supplementary file 3 [file DataSheet4.zip › Mitotracker(1,2)/Mitotracker-1/Mitotracker-1═╝╞1⁄4/Iohexol 4h/Ioh 4h-3/3_RGB_SR405.tif]

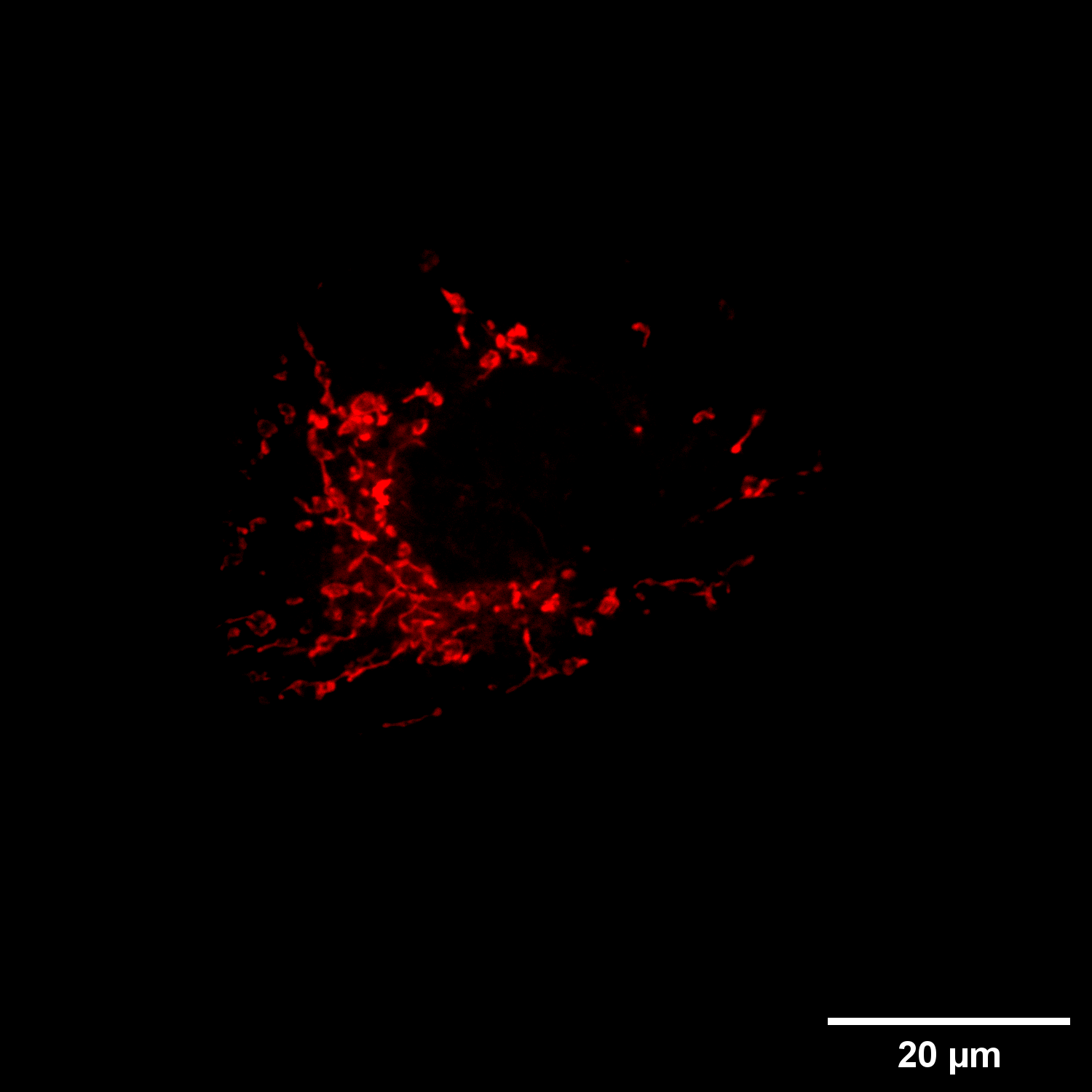

Supplement: Supplementary file 3 [file DataSheet4.zip › Mitotracker(1,2)/Mitotracker-1/Mitotracker-1═╝╞1⁄4/Iohexol 4h/Ioh 4h-3/3_RGB_SR561.tif]

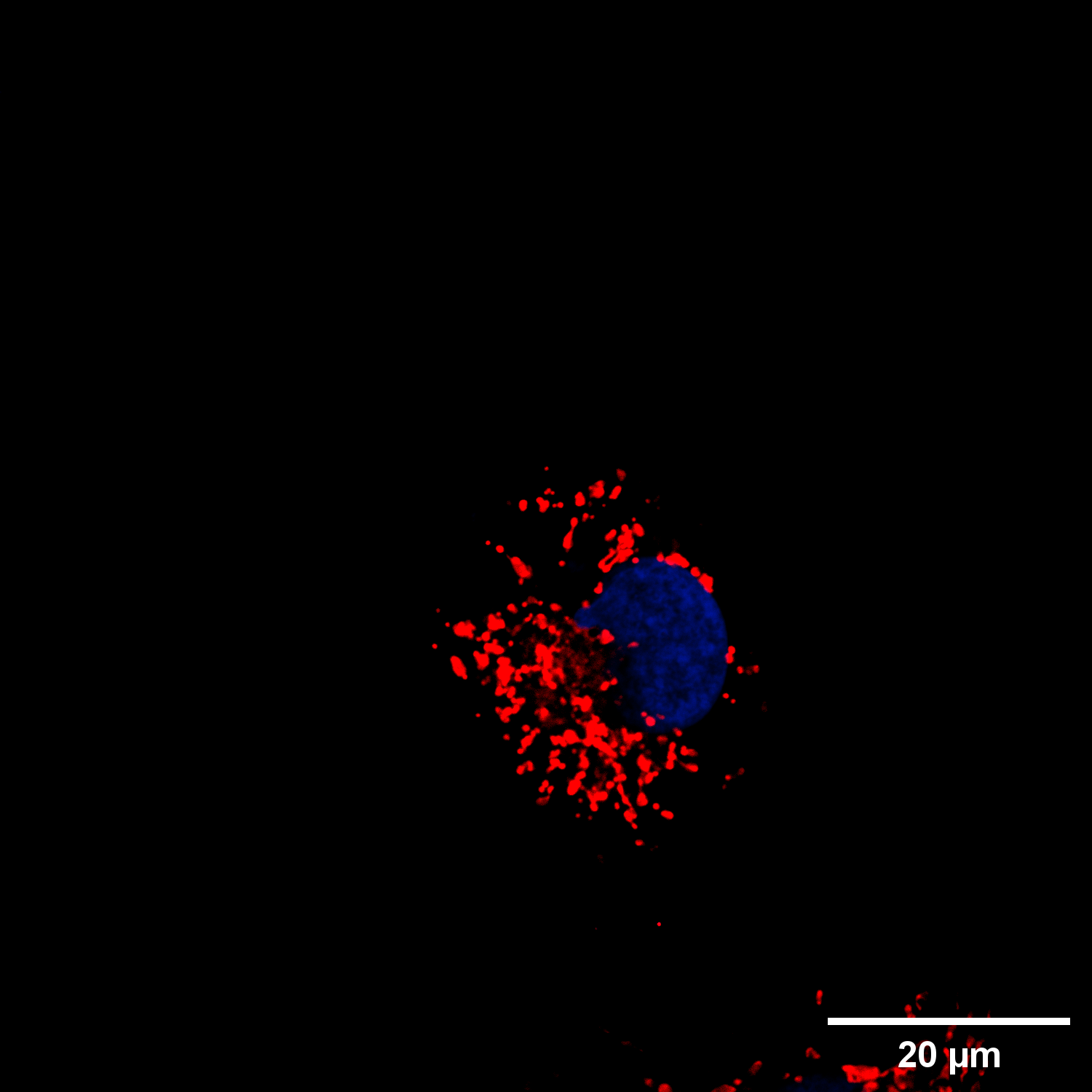

Supplement: Supplementary file 3 [file DataSheet4.zip › Mitotracker(1,2)/Mitotracker-1/Mitotracker-1═╝╞1⁄4/Iohexol 8h/Ioh 8h-1/1_RGB.tif]

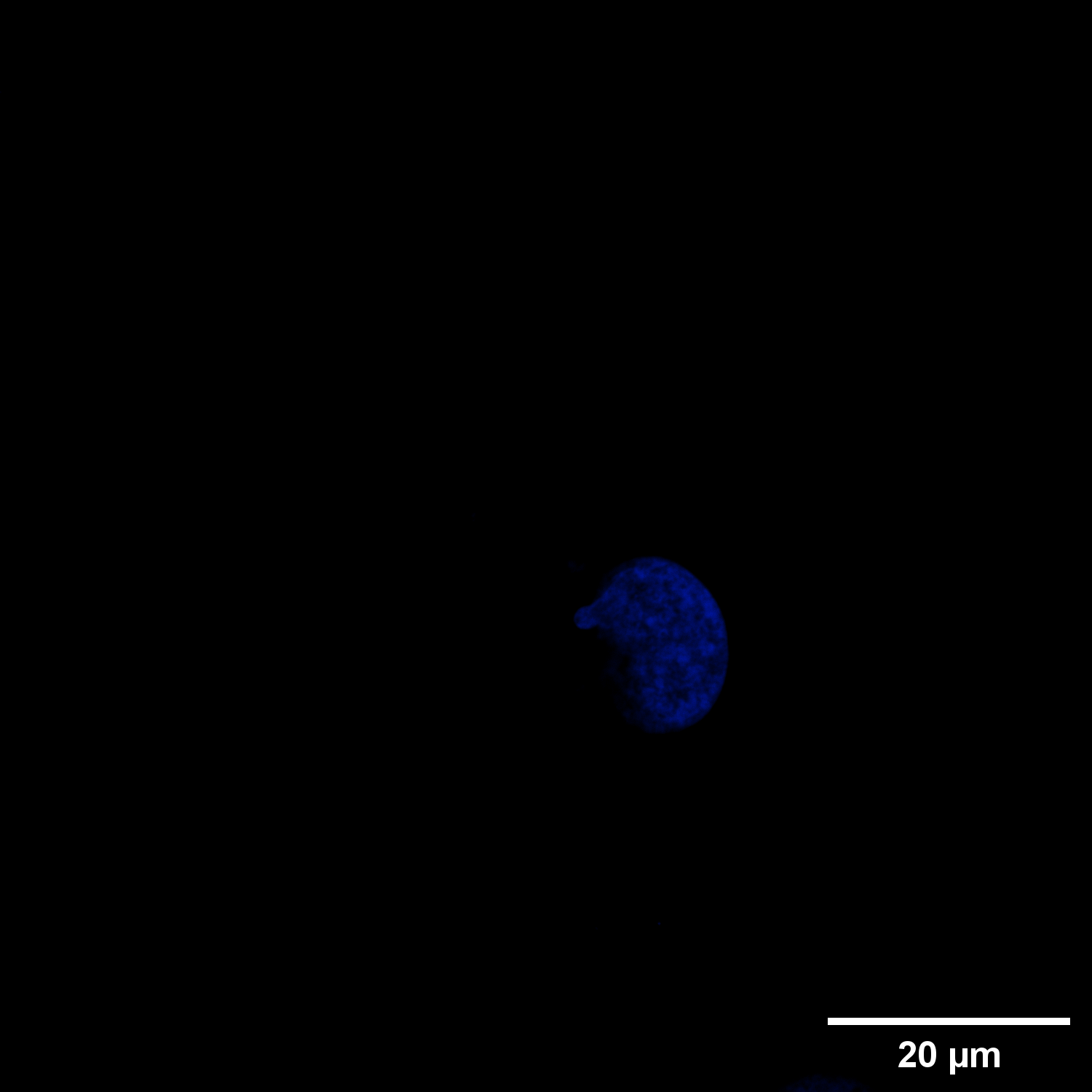

Supplement: Supplementary file 3 [file DataSheet4.zip › Mitotracker(1,2)/Mitotracker-1/Mitotracker-1═╝╞1⁄4/Iohexol 8h/Ioh 8h-1/1_RGB_SR405.tif]

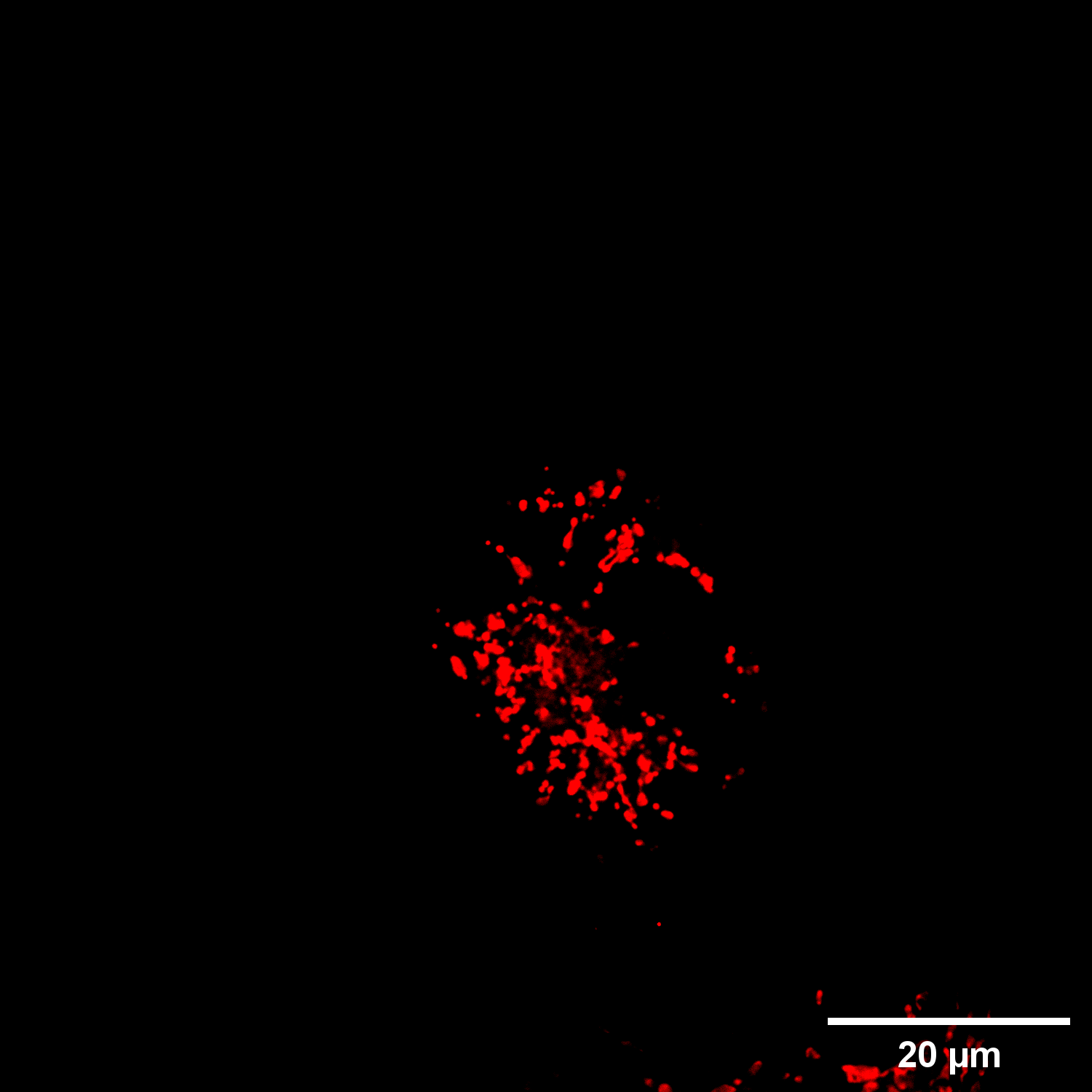

Supplement: Supplementary file 3 [file DataSheet4.zip › Mitotracker(1,2)/Mitotracker-1/Mitotracker-1═╝╞1⁄4/Iohexol 8h/Ioh 8h-1/1_RGB_SR561.tif]

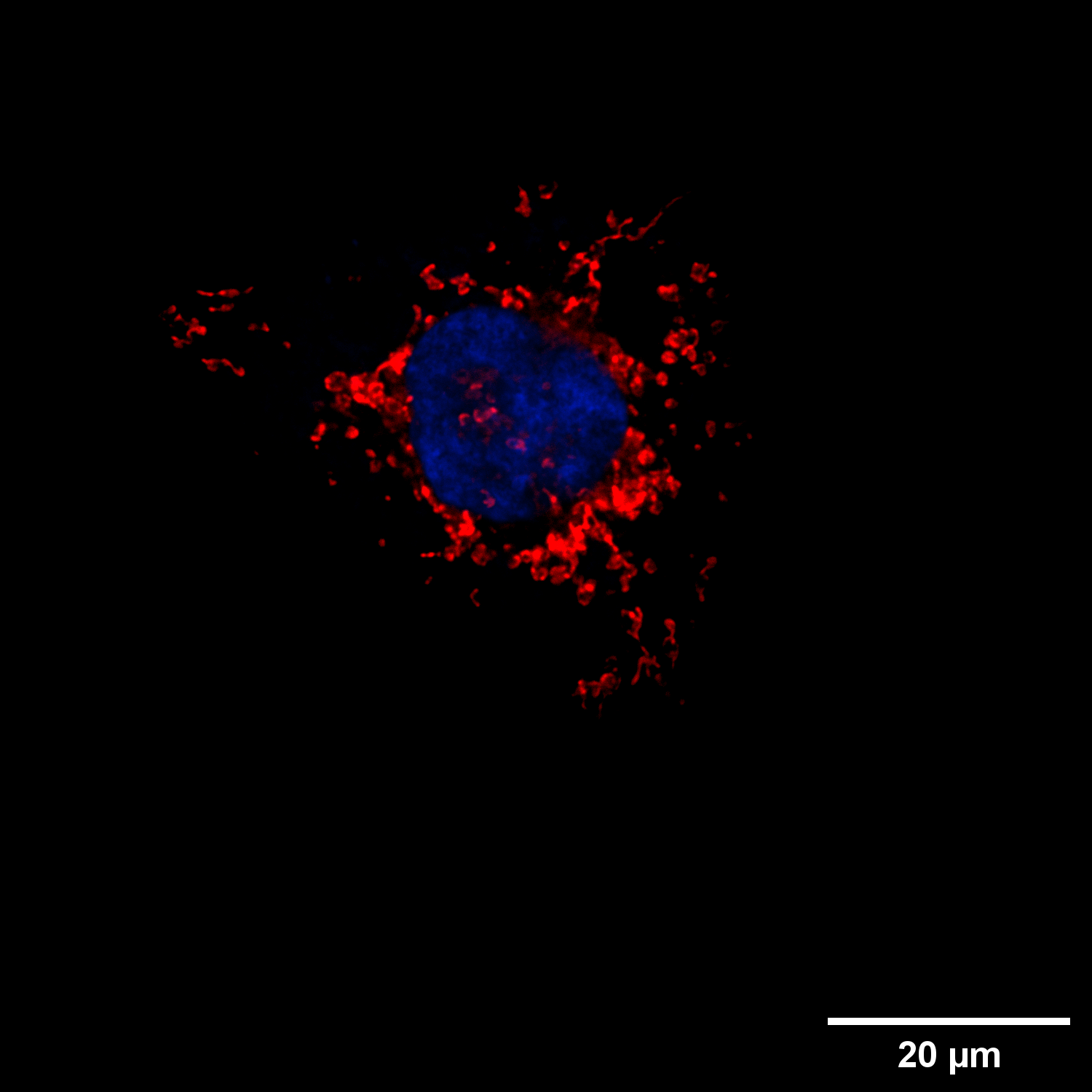

Supplement: Supplementary file 3 [file DataSheet4.zip › Mitotracker(1,2)/Mitotracker-1/Mitotracker-1═╝╞1⁄4/Iohexol 8h/Ioh 8h-2/2_RGB.tif]

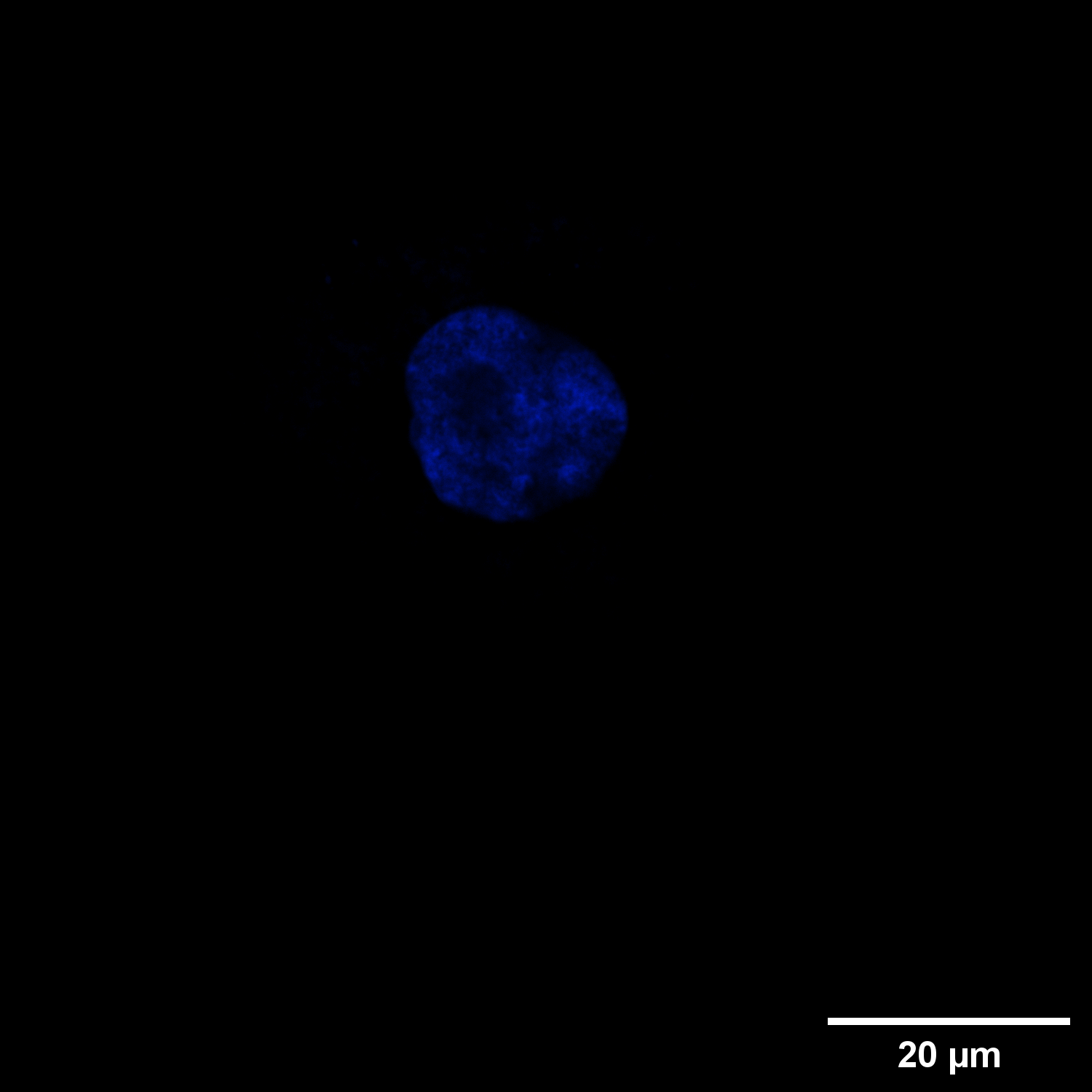

Supplement: Supplementary file 3 [file DataSheet4.zip › Mitotracker(1,2)/Mitotracker-1/Mitotracker-1═╝╞1⁄4/Iohexol 8h/Ioh 8h-2/2_RGB_SR405.tif]

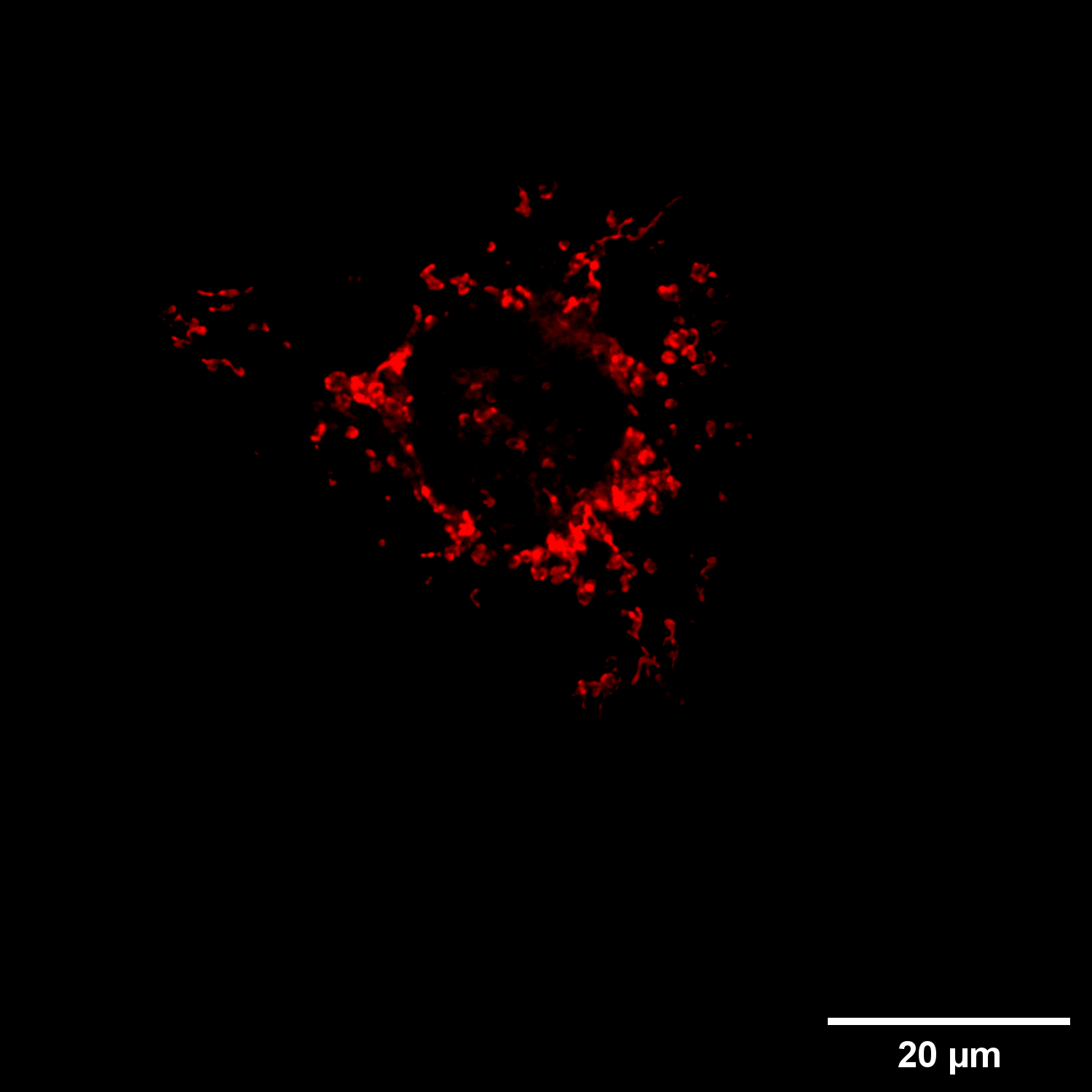

Supplement: Supplementary file 3 [file DataSheet4.zip › Mitotracker(1,2)/Mitotracker-1/Mitotracker-1═╝╞1⁄4/Iohexol 8h/Ioh 8h-2/2_RGB_SR561.tif]

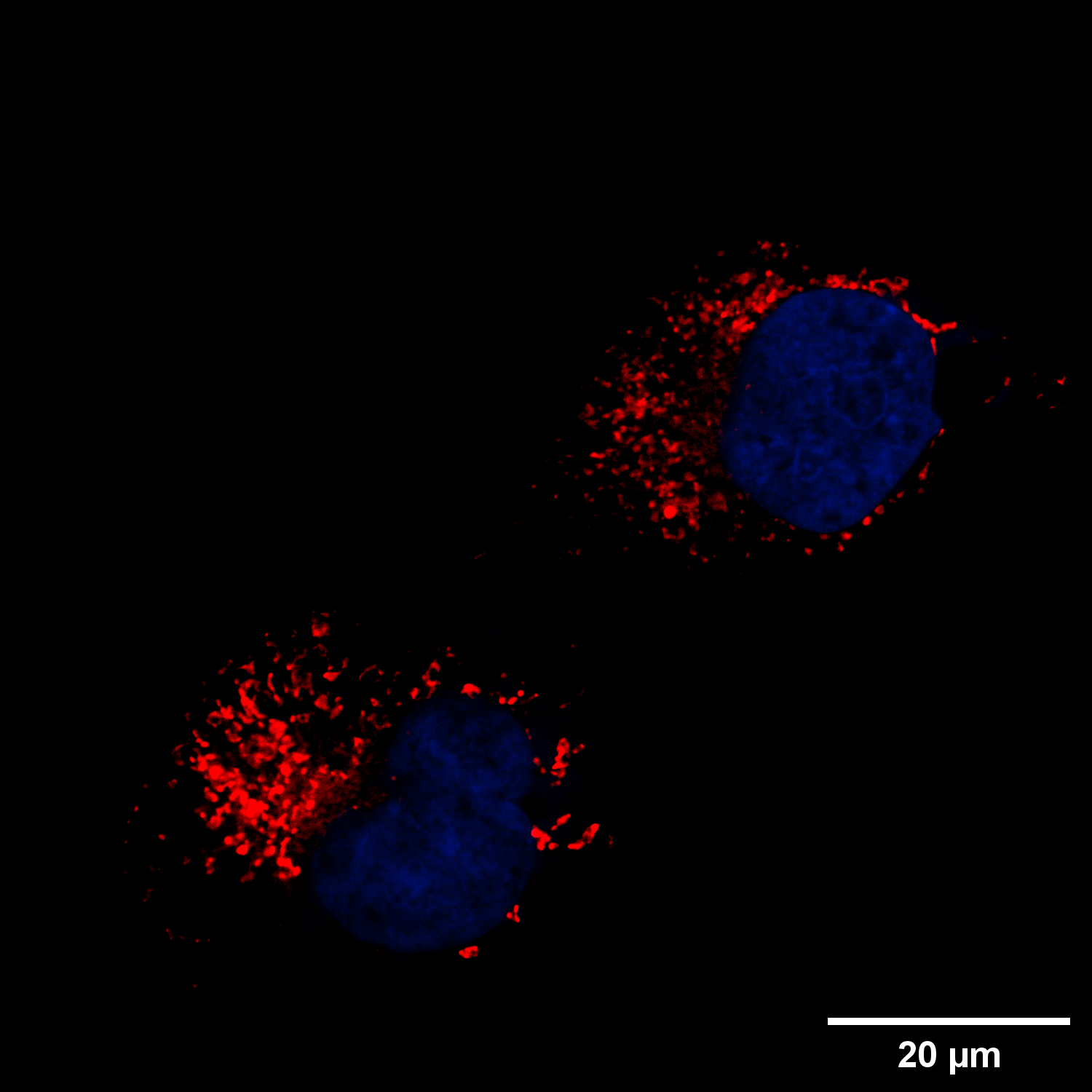

Supplement: Supplementary file 3 [file DataSheet4.zip › Mitotracker(1,2)/Mitotracker-1/Mitotracker-1═╝╞1⁄4/Iohexol 8h/Ioh 8h-3/3_RGB.tif]

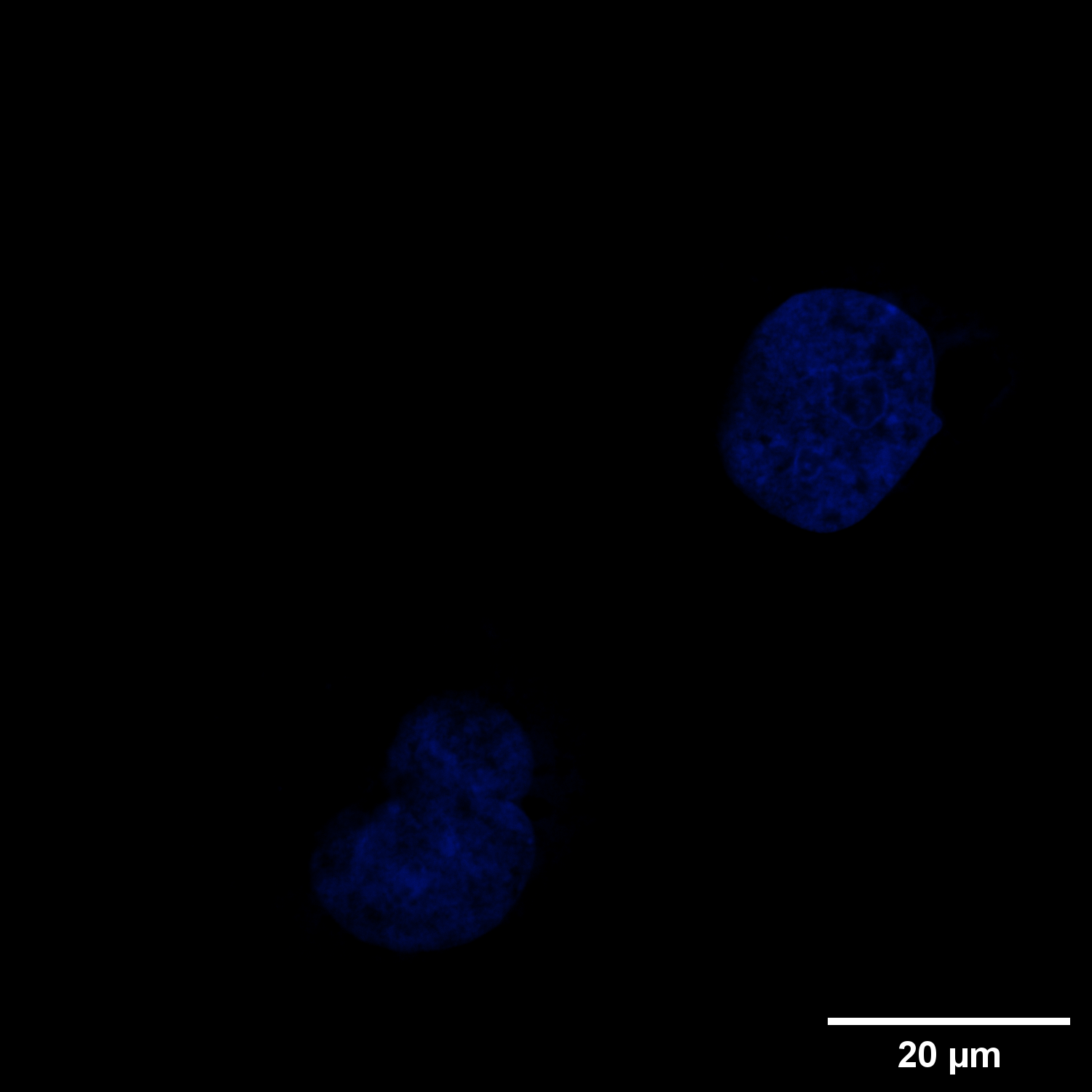

Supplement: Supplementary file 3 [file DataSheet4.zip › Mitotracker(1,2)/Mitotracker-1/Mitotracker-1═╝╞1⁄4/Iohexol 8h/Ioh 8h-3/3_RGB_SR405.tif]

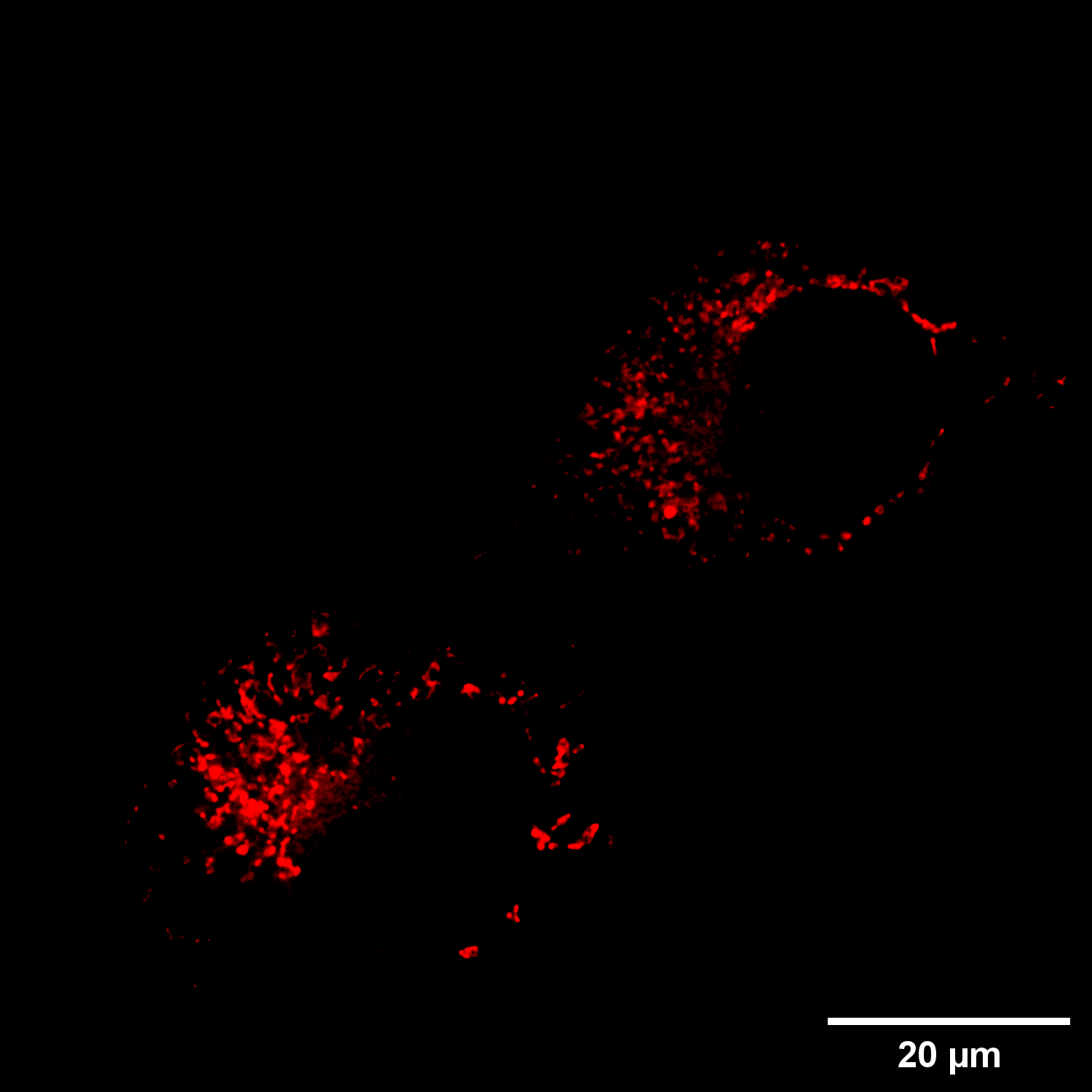

Supplement: Supplementary file 3 [file DataSheet4.zip › Mitotracker(1,2)/Mitotracker-1/Mitotracker-1═╝╞1⁄4/Iohexol 8h/Ioh 8h-3/3_RGB_SR561.tif]

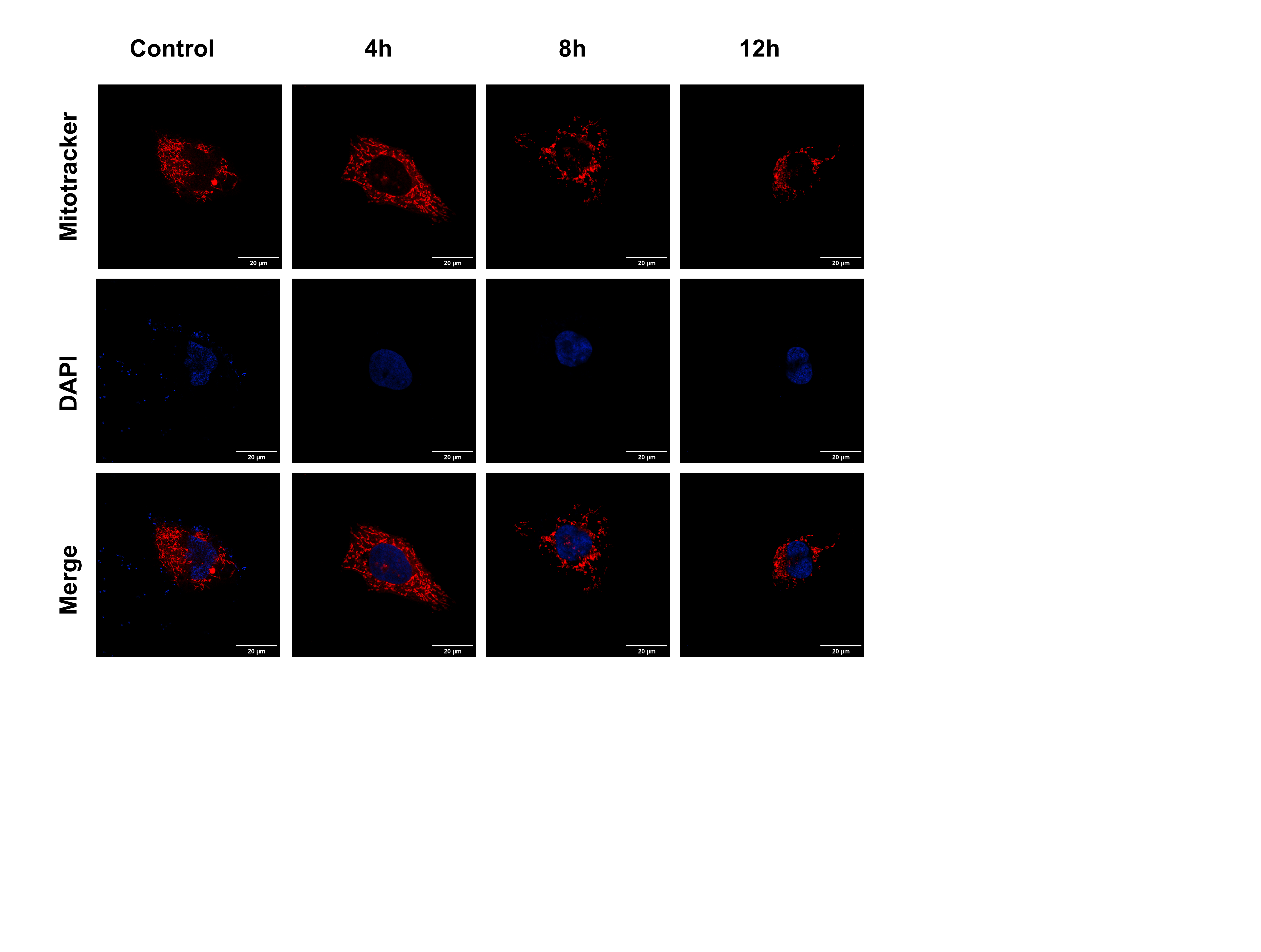

Supplement: Supplementary file 3 [file DataSheet4.zip › Mitotracker(1,2)/Mitotracker-1/Mitotracker-1═╝╞1⁄4/mitotracker-1║╧═╝.tif]

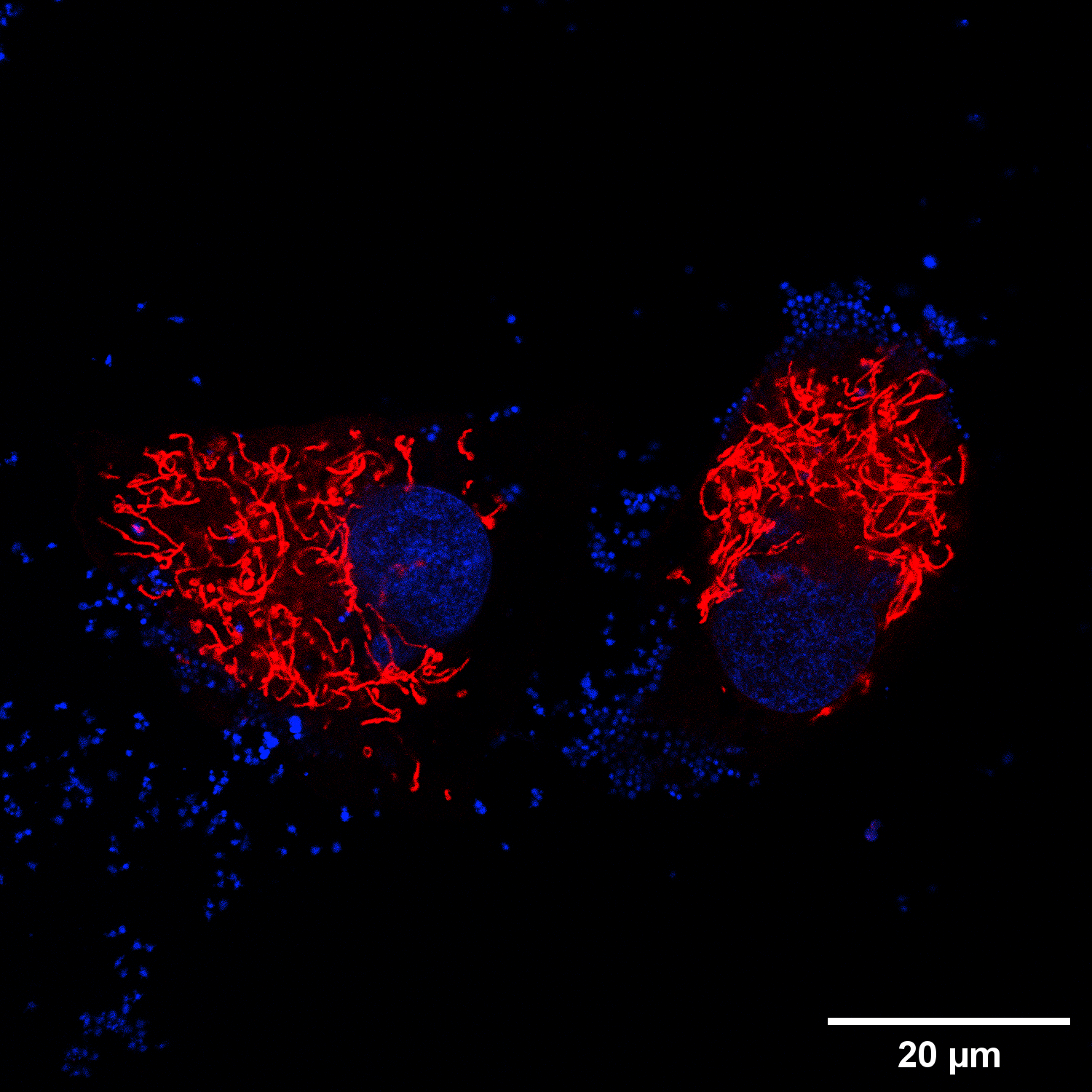

Supplement: Supplementary file 3 [file DataSheet4.zip › Mitotracker(1,2)/Mitotracker-2/Mitotracker-2═╝╞1⁄4/Control/Con-1/1_RGB.tif]

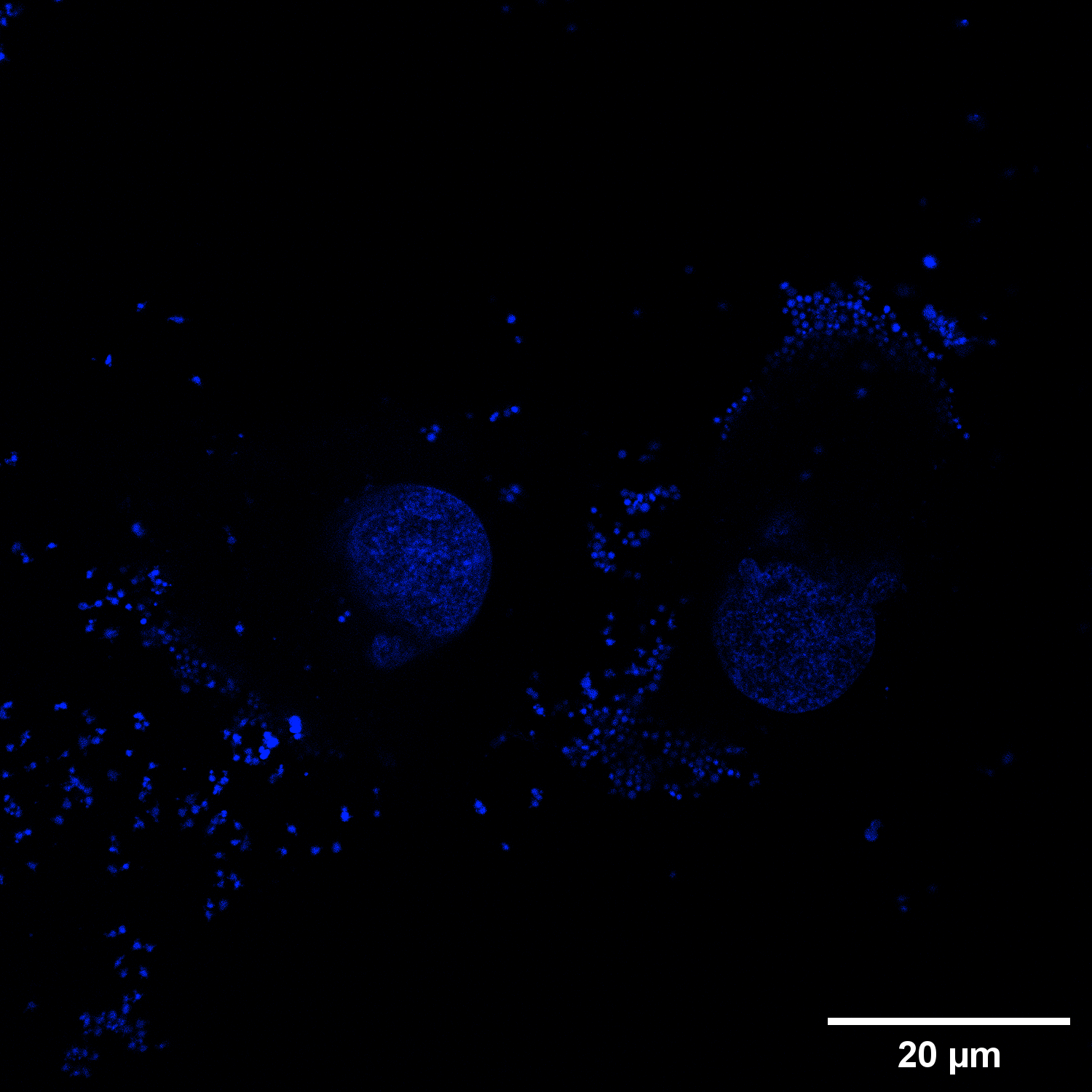

Supplement: Supplementary file 3 [file DataSheet4.zip › Mitotracker(1,2)/Mitotracker-2/Mitotracker-2═╝╞1⁄4/Control/Con-1/1_RGB_SR405.tif]

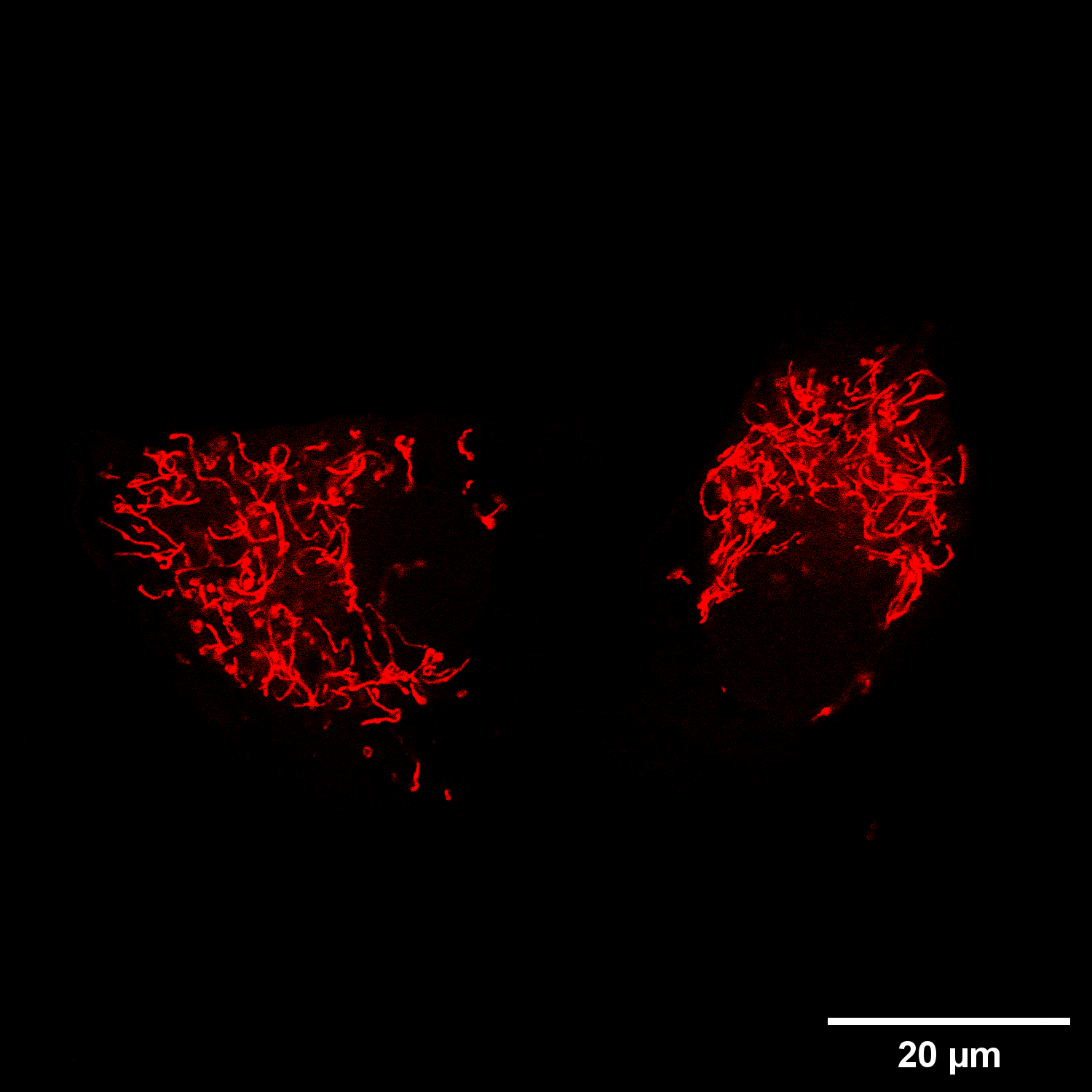

Supplement: Supplementary file 3 [file DataSheet4.zip › Mitotracker(1,2)/Mitotracker-2/Mitotracker-2═╝╞1⁄4/Control/Con-1/1_RGB_SR561.tif]

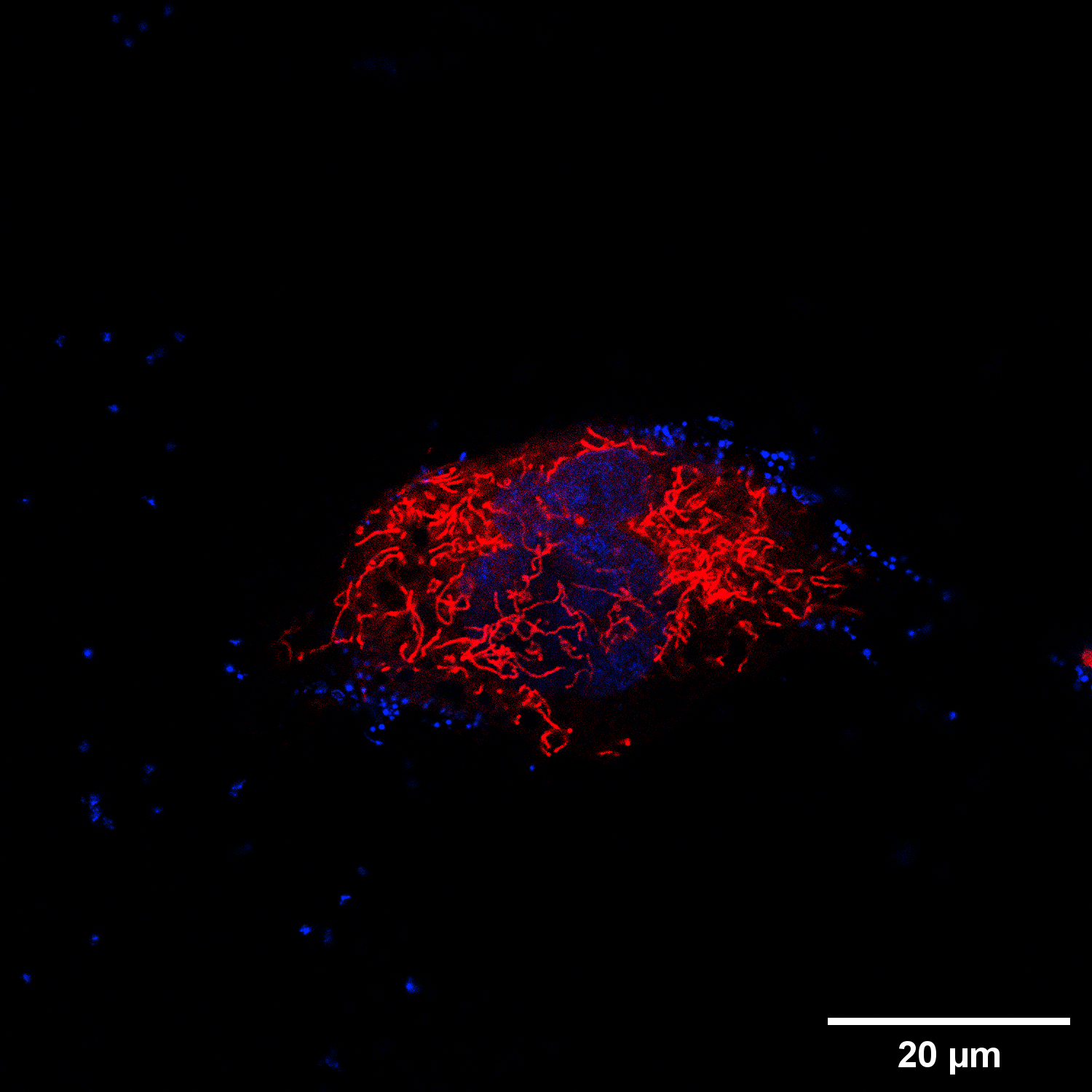

Supplement: Supplementary file 3 [file DataSheet4.zip › Mitotracker(1,2)/Mitotracker-2/Mitotracker-2═╝╞1⁄4/Control/Con-2/2_RGB.tif]
