# Supplementary figures and images for: Mitochondrial calcium uniporter-mediated mitochondrial dynamics imbalance contributes to contrast medium-induced renal tubular cell injury (part 1 of 4)
Source: Front Mol Biosci. 2026 Jun 29;13:1848361. doi: 10.3389/fmolb.2026.1848361 (PMC13357276; doi:10.3389/fmolb.2026.1848361)

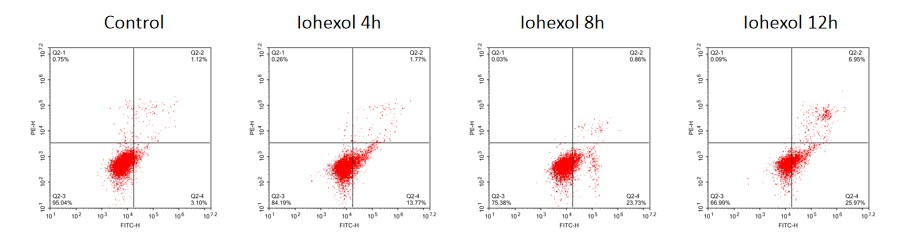

Supplement: Supplementary file 1 [file DataSheet3.zip › Flow Cytometry Assay(1,2)/Flow Cytometry Assay-1/╧╕░√╡≥═÷-1/╡≥═÷ 1/1.jpg]

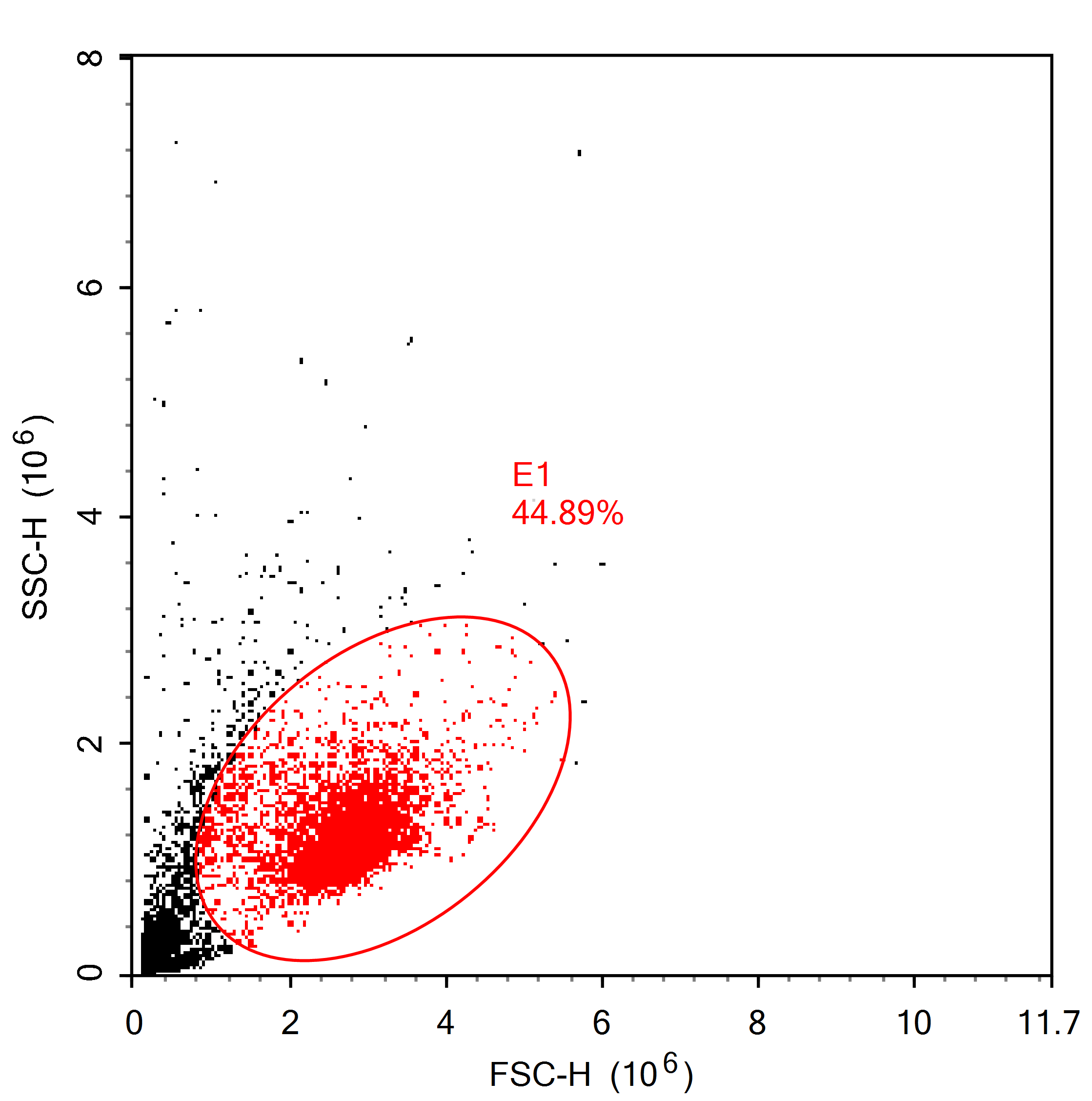

Supplement: Supplementary file 1 [file DataSheet3.zip › Flow Cytometry Assay(1,2)/Flow Cytometry Assay-1/╧╕░√╡≥═÷-1/╡≥═÷ 1/═╝╞1⁄4/12h 1/═╝1.tiff]

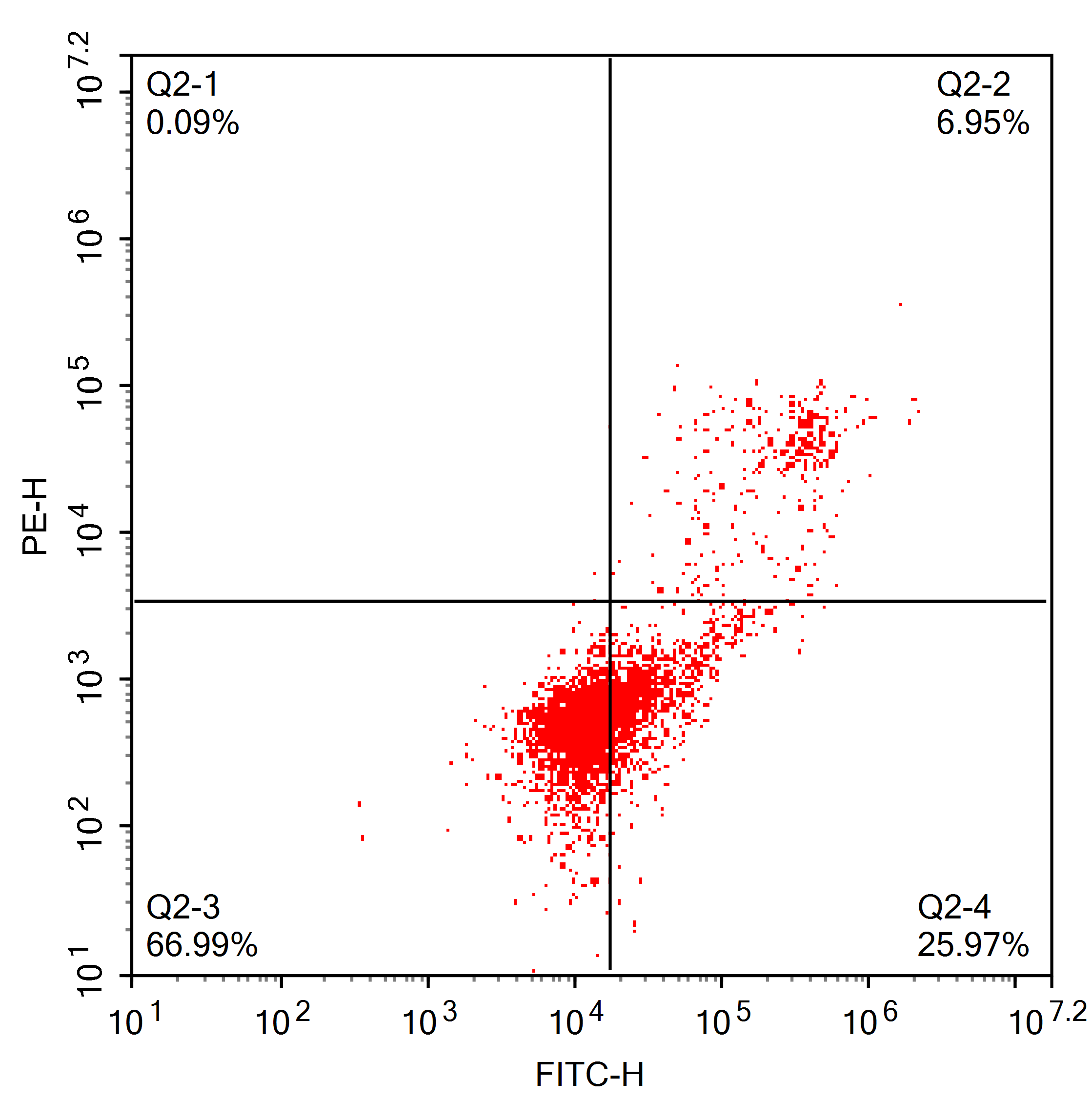

Supplement: Supplementary file 1 [file DataSheet3.zip › Flow Cytometry Assay(1,2)/Flow Cytometry Assay-1/╧╕░√╡≥═÷-1/╡≥═÷ 1/═╝╞1⁄4/12h 1/═╝2.tiff]

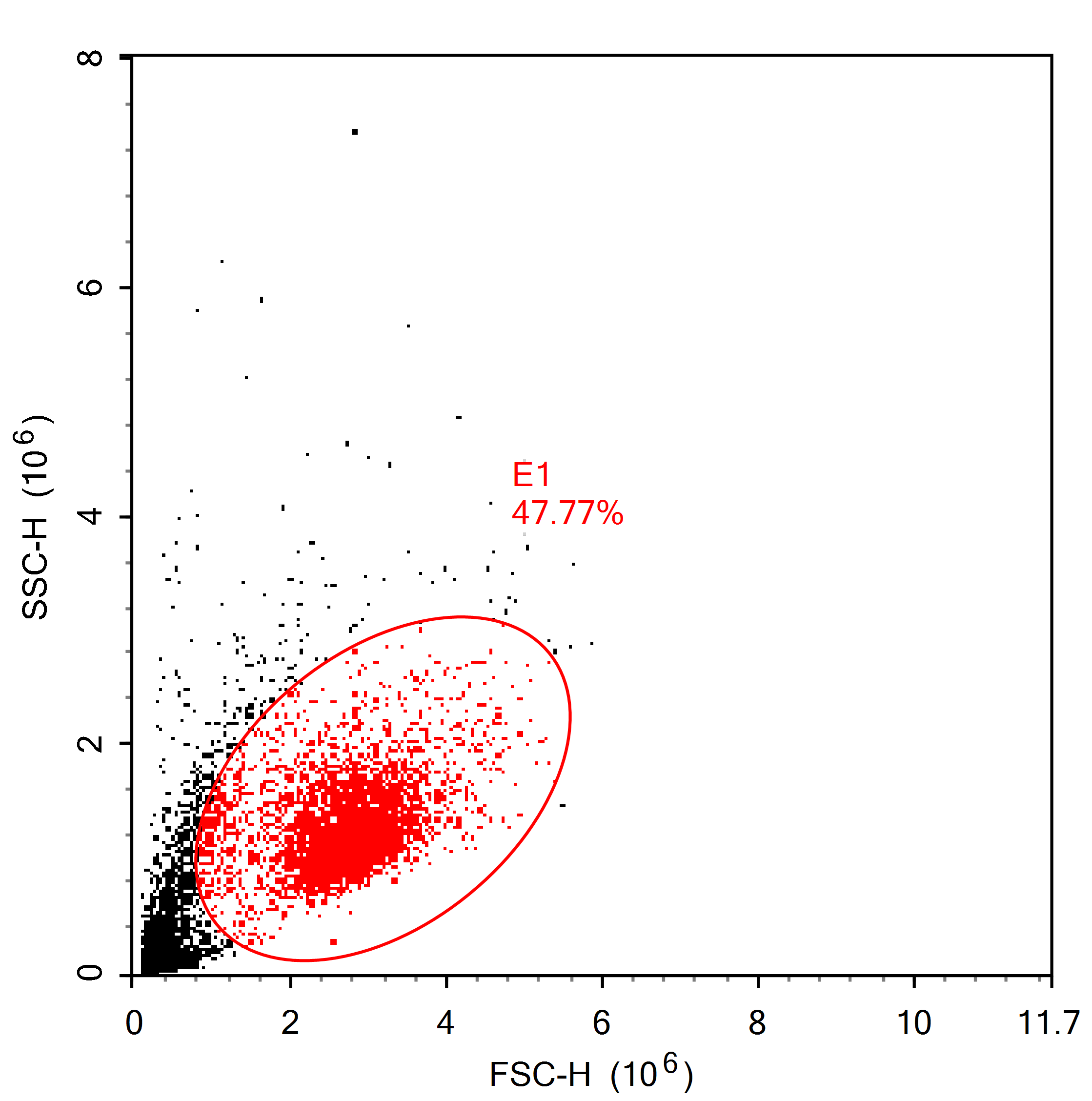

Supplement: Supplementary file 1 [file DataSheet3.zip › Flow Cytometry Assay(1,2)/Flow Cytometry Assay-1/╧╕░√╡≥═÷-1/╡≥═÷ 1/═╝╞1⁄4/12h 2/═╝1.tiff]

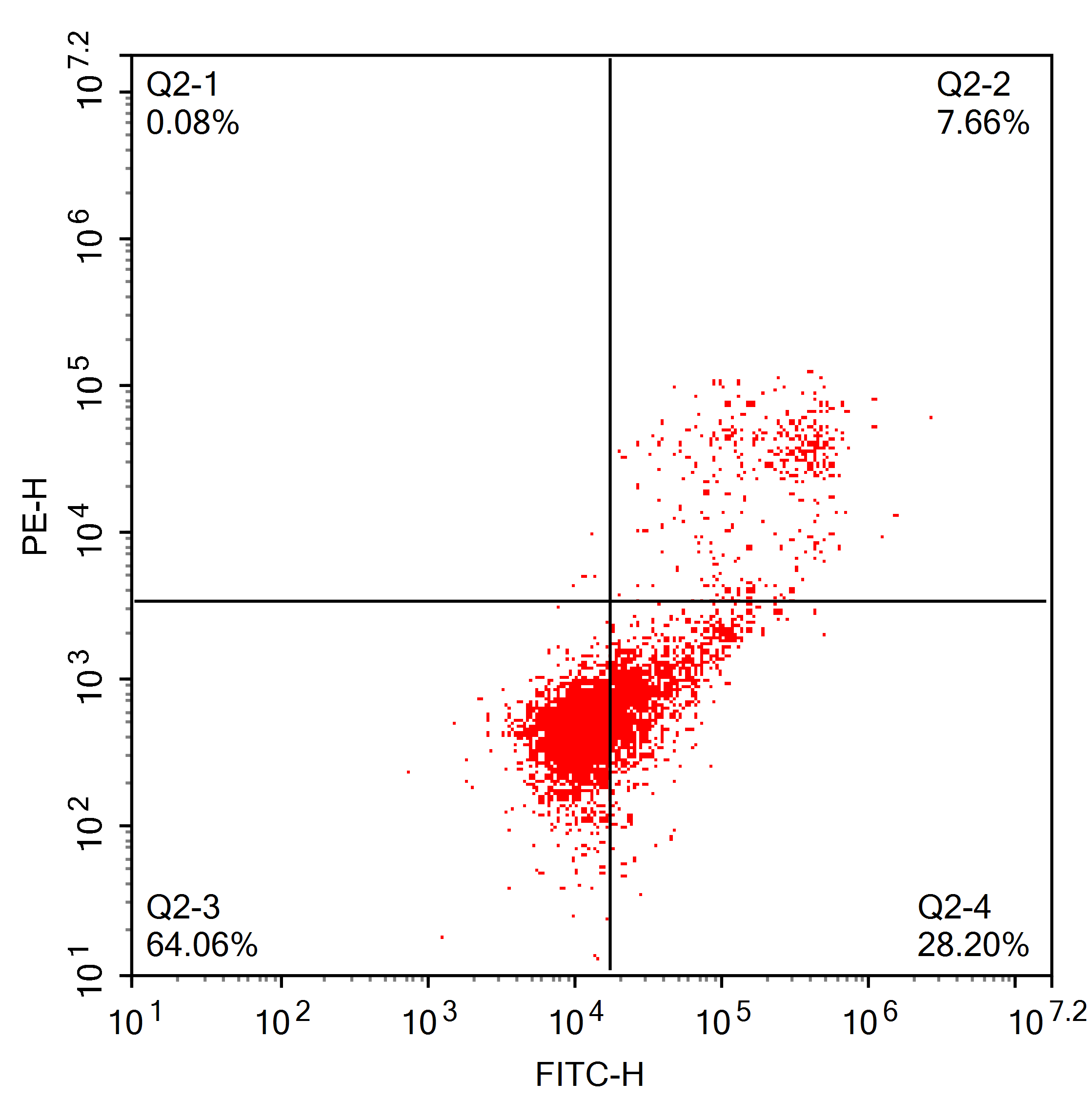

Supplement: Supplementary file 1 [file DataSheet3.zip › Flow Cytometry Assay(1,2)/Flow Cytometry Assay-1/╧╕░√╡≥═÷-1/╡≥═÷ 1/═╝╞1⁄4/12h 2/═╝2.tiff]

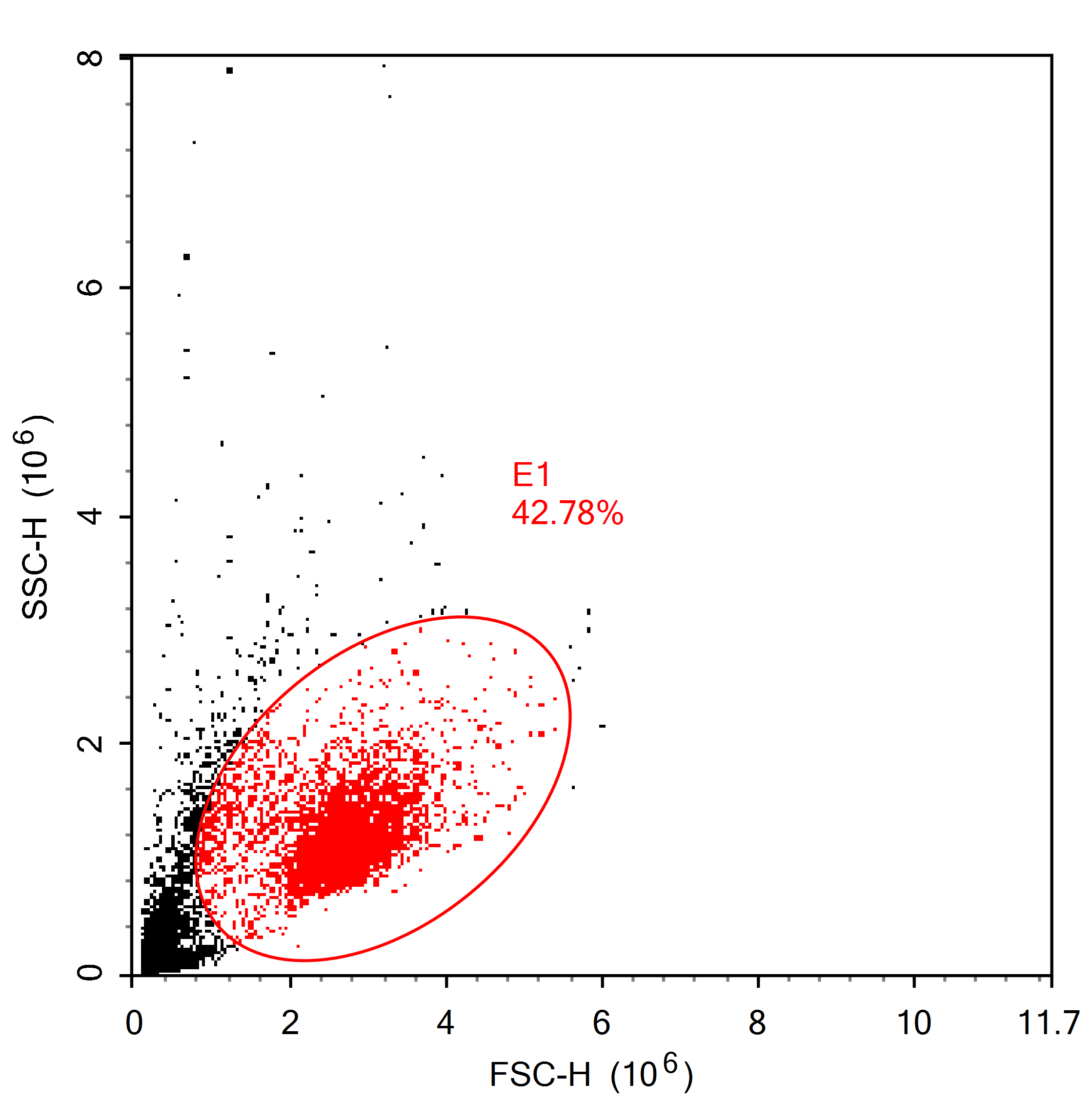

Supplement: Supplementary file 1 [file DataSheet3.zip › Flow Cytometry Assay(1,2)/Flow Cytometry Assay-1/╧╕░√╡≥═÷-1/╡≥═÷ 1/═╝╞1⁄4/12h 3/═╝1.tiff]

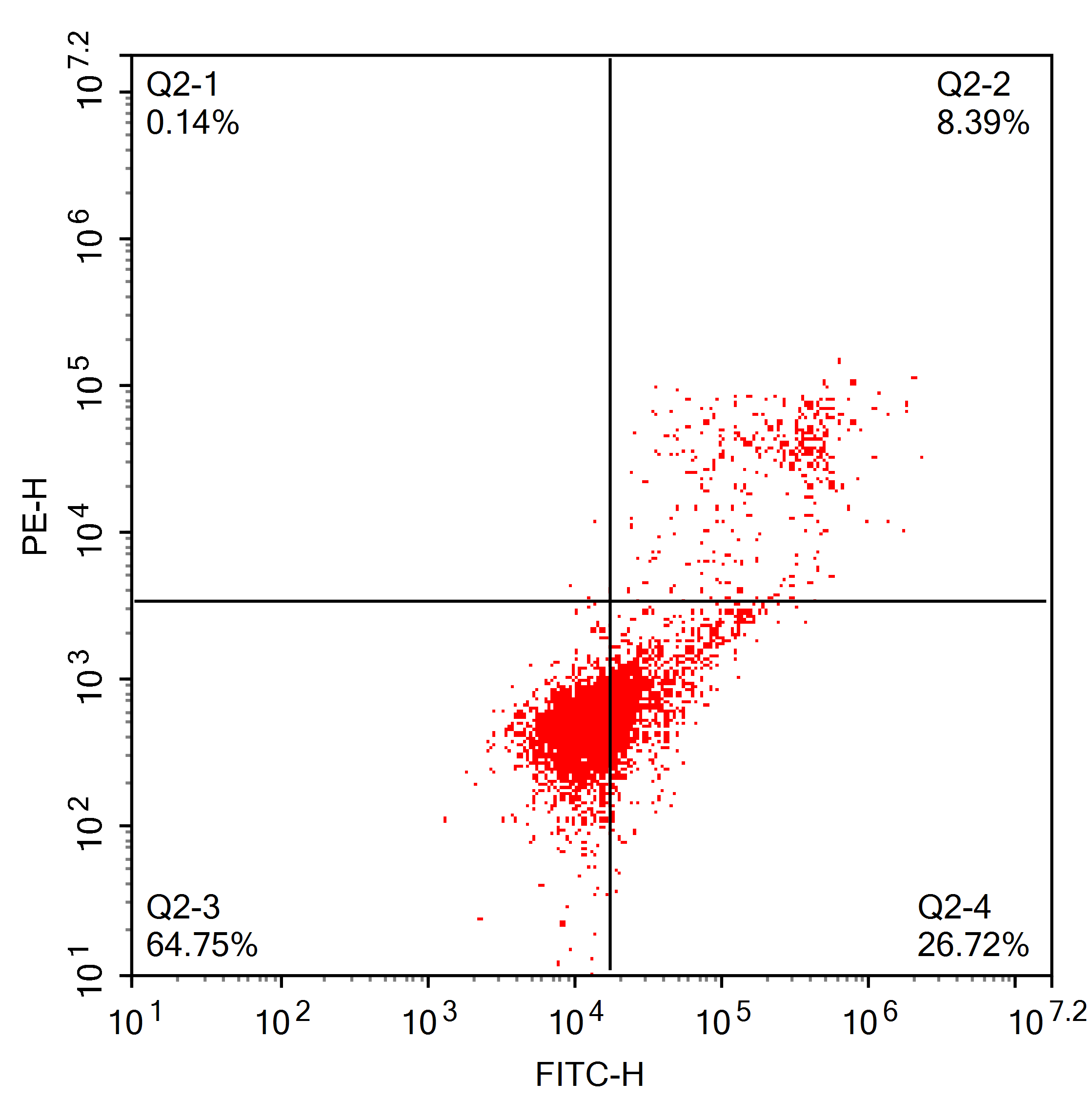

Supplement: Supplementary file 1 [file DataSheet3.zip › Flow Cytometry Assay(1,2)/Flow Cytometry Assay-1/╧╕░√╡≥═÷-1/╡≥═÷ 1/═╝╞1⁄4/12h 3/═╝2.tiff]

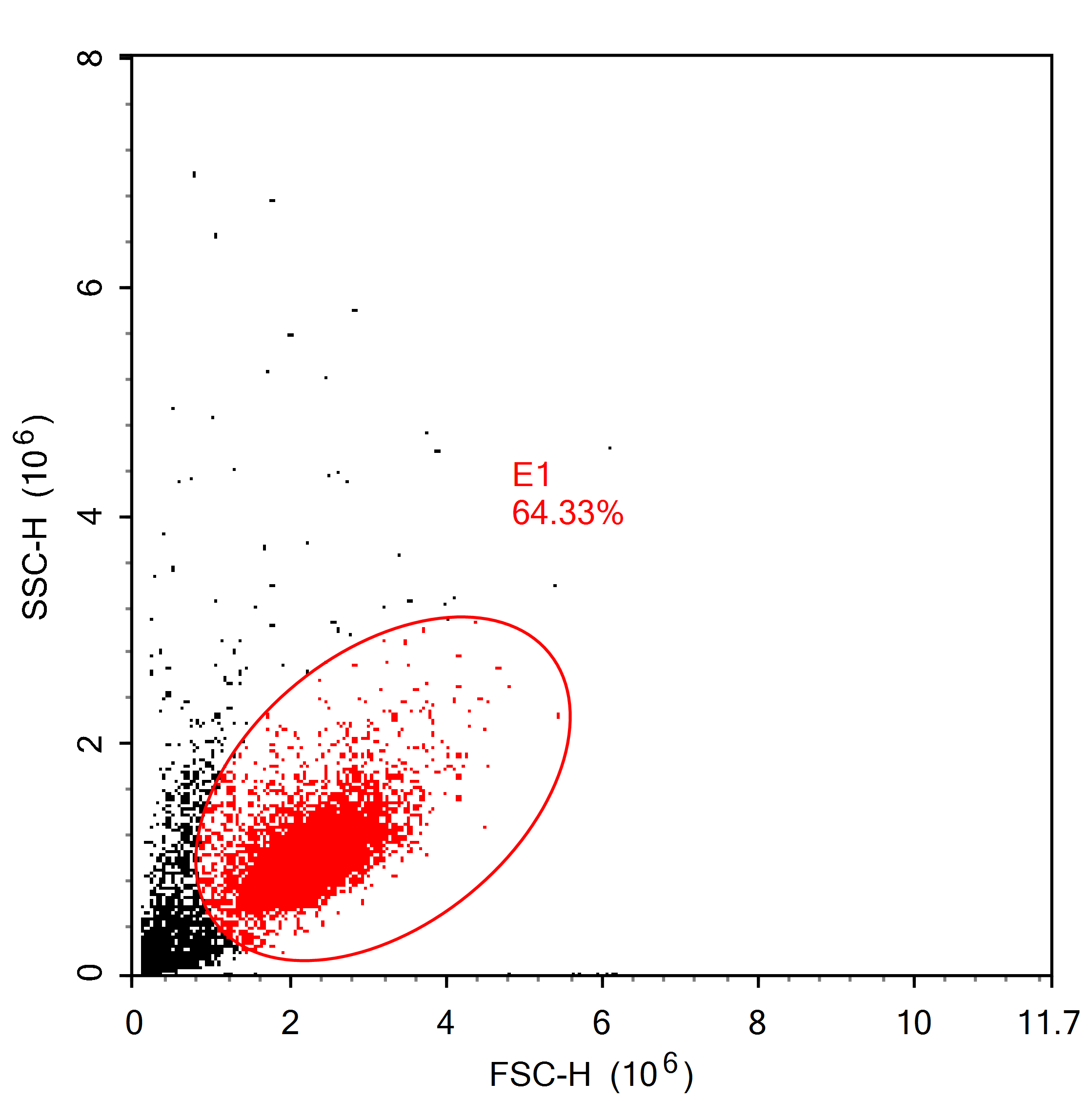

Supplement: Supplementary file 1 [file DataSheet3.zip › Flow Cytometry Assay(1,2)/Flow Cytometry Assay-1/╧╕░√╡≥═÷-1/╡≥═÷ 1/═╝╞1⁄4/4h 1/═╝1.tiff]

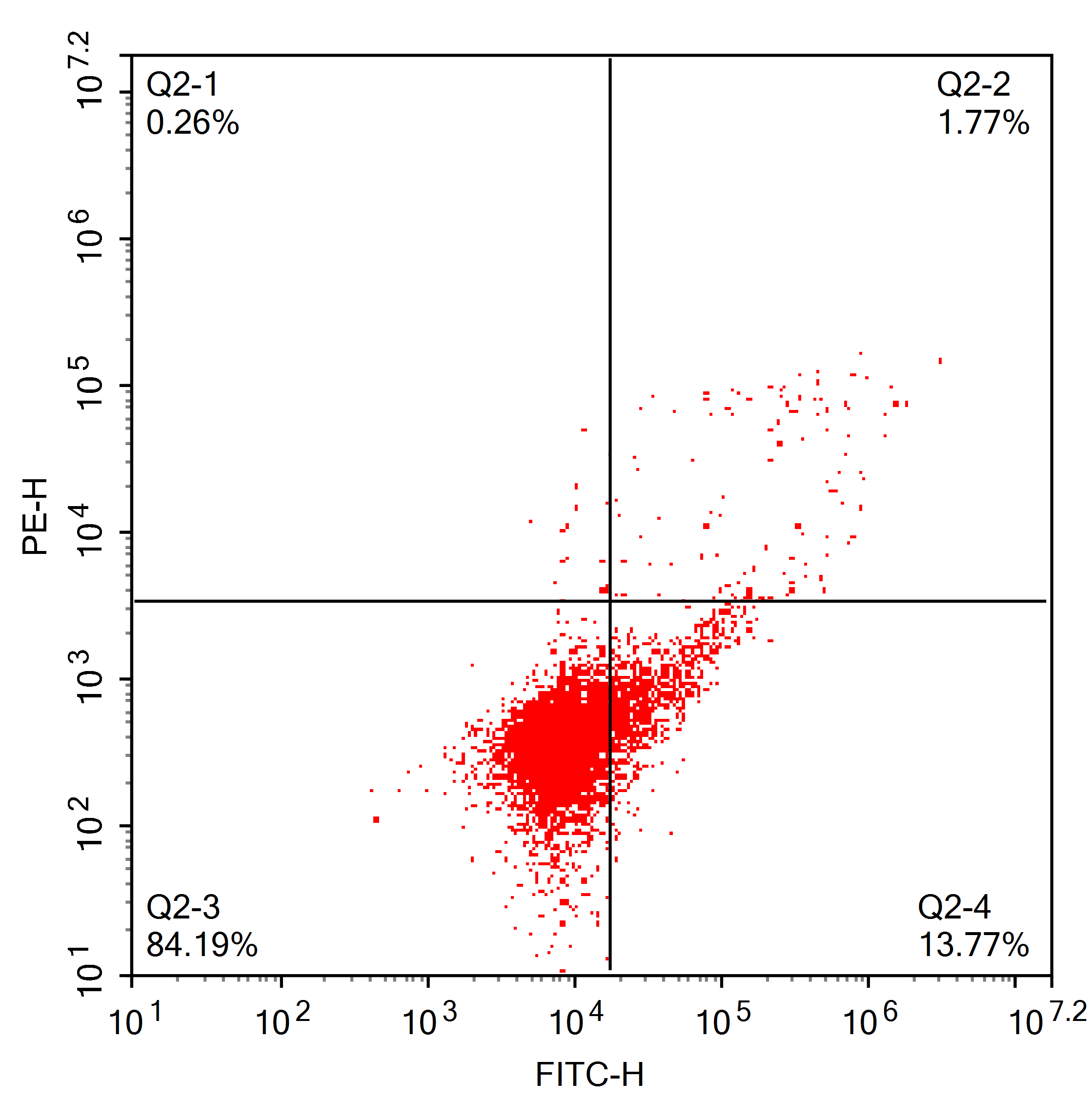

Supplement: Supplementary file 1 [file DataSheet3.zip › Flow Cytometry Assay(1,2)/Flow Cytometry Assay-1/╧╕░√╡≥═÷-1/╡≥═÷ 1/═╝╞1⁄4/4h 1/═╝2.tiff]

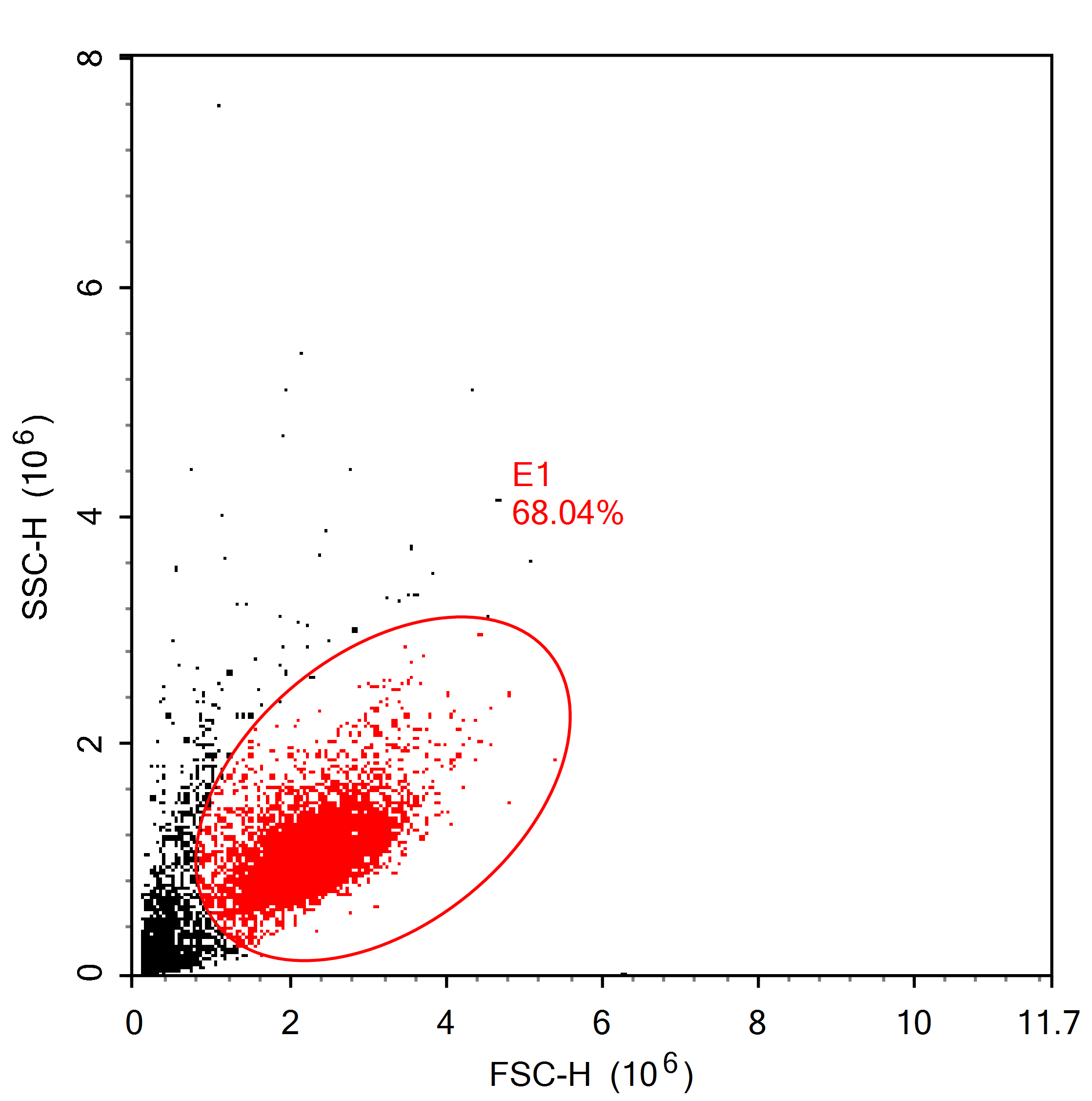

Supplement: Supplementary file 1 [file DataSheet3.zip › Flow Cytometry Assay(1,2)/Flow Cytometry Assay-1/╧╕░√╡≥═÷-1/╡≥═÷ 1/═╝╞1⁄4/4h 2/═╝1.tiff]

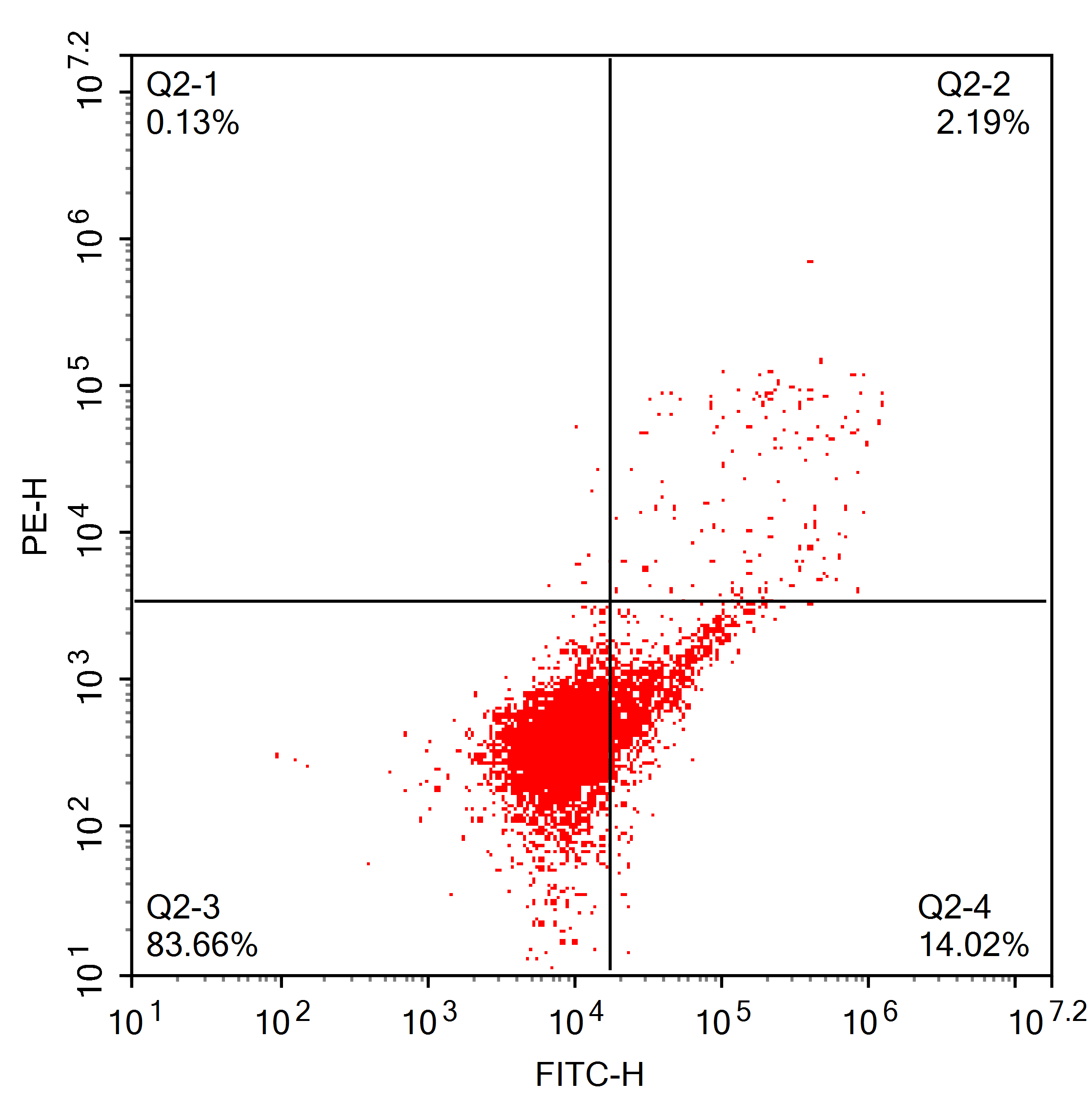

Supplement: Supplementary file 1 [file DataSheet3.zip › Flow Cytometry Assay(1,2)/Flow Cytometry Assay-1/╧╕░√╡≥═÷-1/╡≥═÷ 1/═╝╞1⁄4/4h 2/═╝2.tiff]

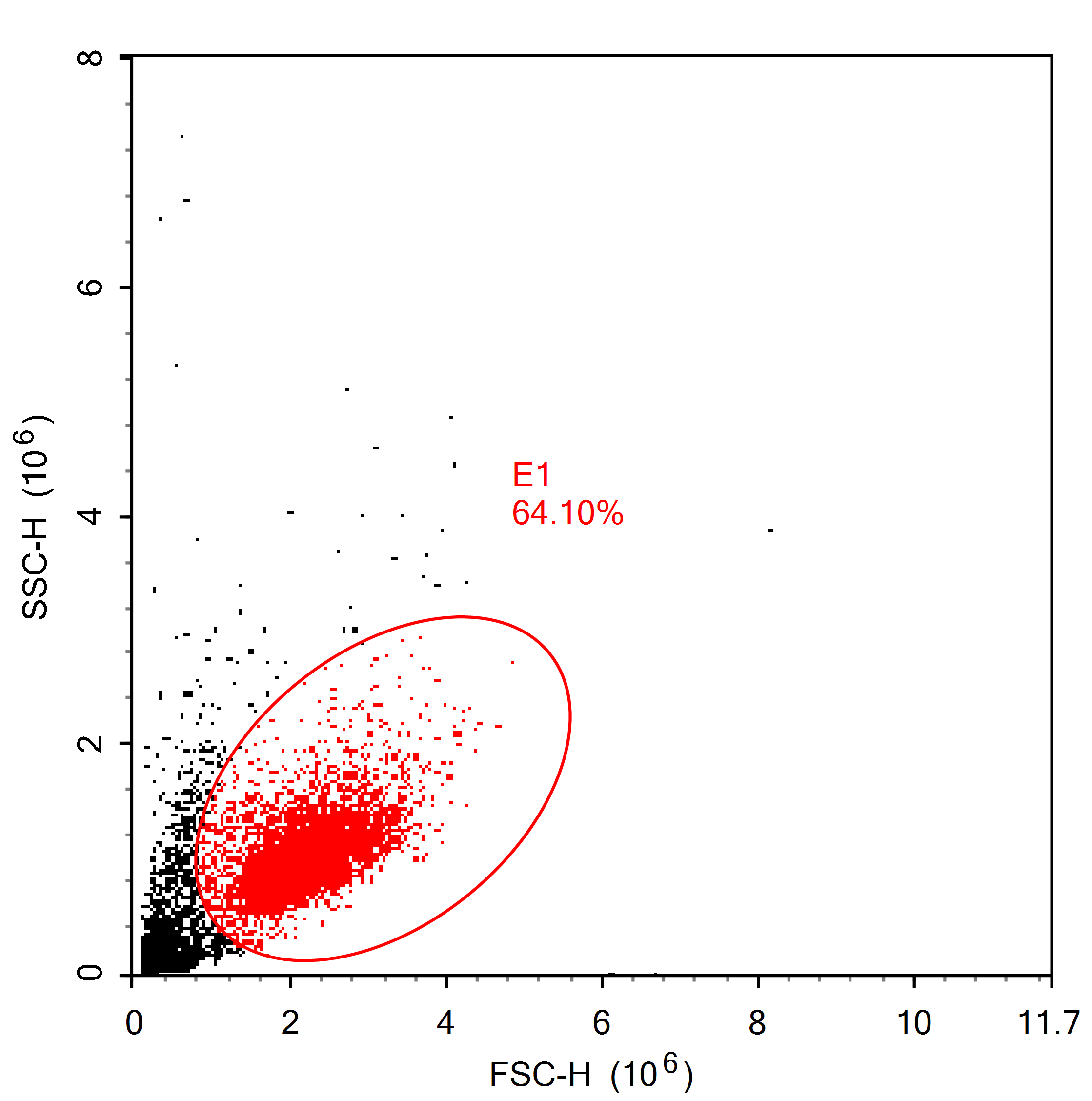

Supplement: Supplementary file 1 [file DataSheet3.zip › Flow Cytometry Assay(1,2)/Flow Cytometry Assay-1/╧╕░√╡≥═÷-1/╡≥═÷ 1/═╝╞1⁄4/4h 3/═╝1.tiff]

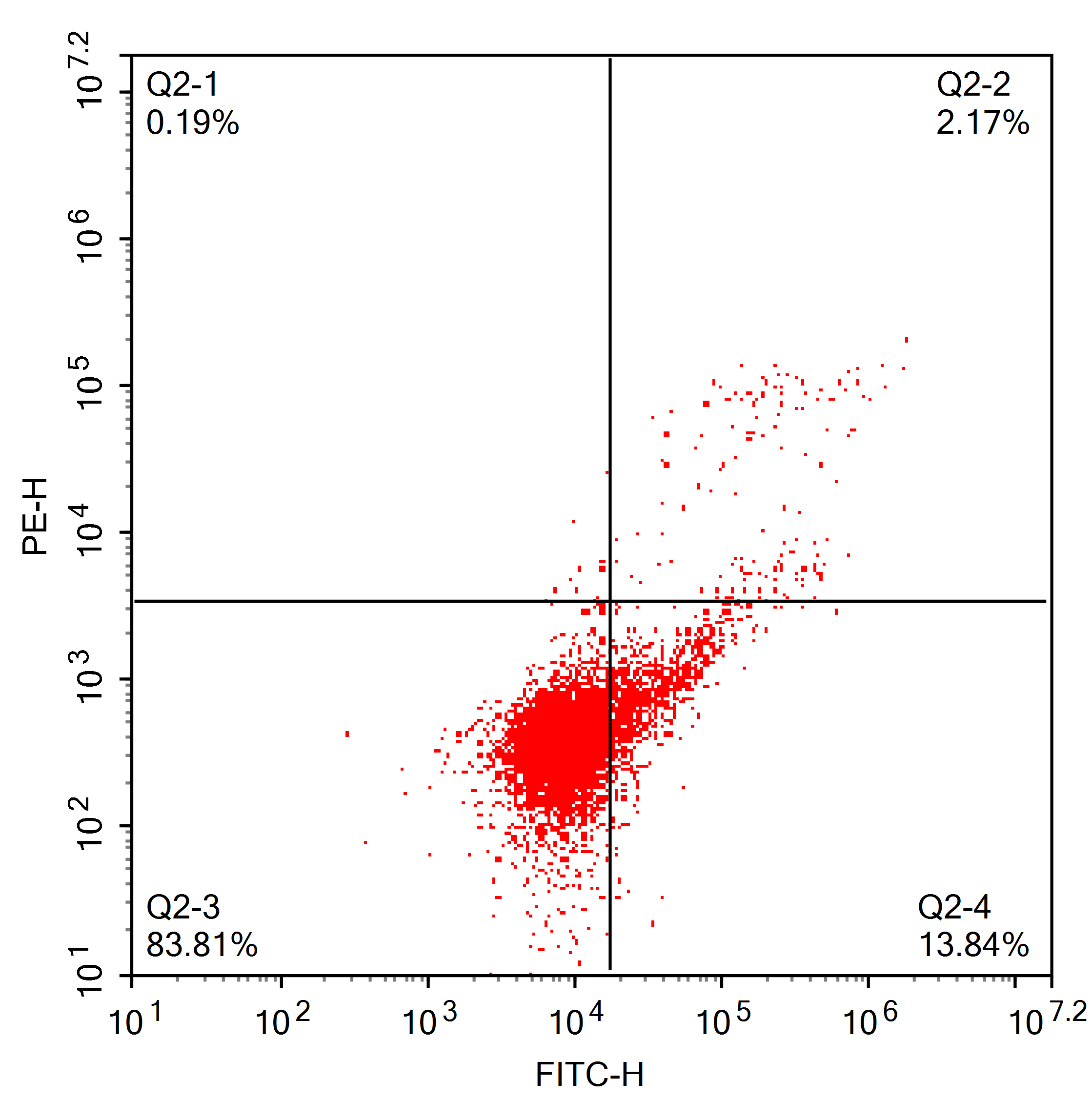

Supplement: Supplementary file 1 [file DataSheet3.zip › Flow Cytometry Assay(1,2)/Flow Cytometry Assay-1/╧╕░√╡≥═÷-1/╡≥═÷ 1/═╝╞1⁄4/4h 3/═╝2.tiff]

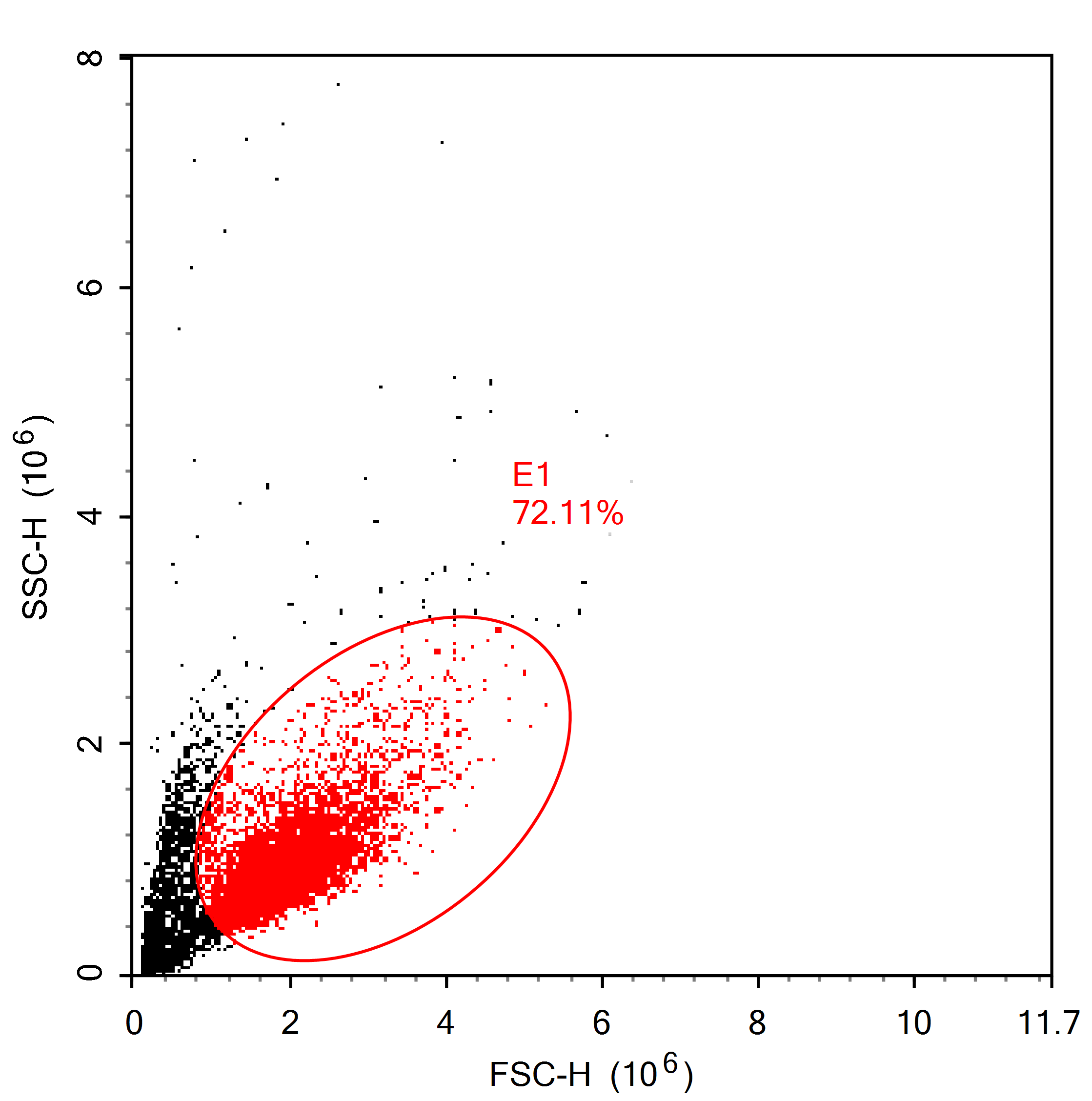

Supplement: Supplementary file 1 [file DataSheet3.zip › Flow Cytometry Assay(1,2)/Flow Cytometry Assay-1/╧╕░√╡≥═÷-1/╡≥═÷ 1/═╝╞1⁄4/8h 1/═╝1.tiff]

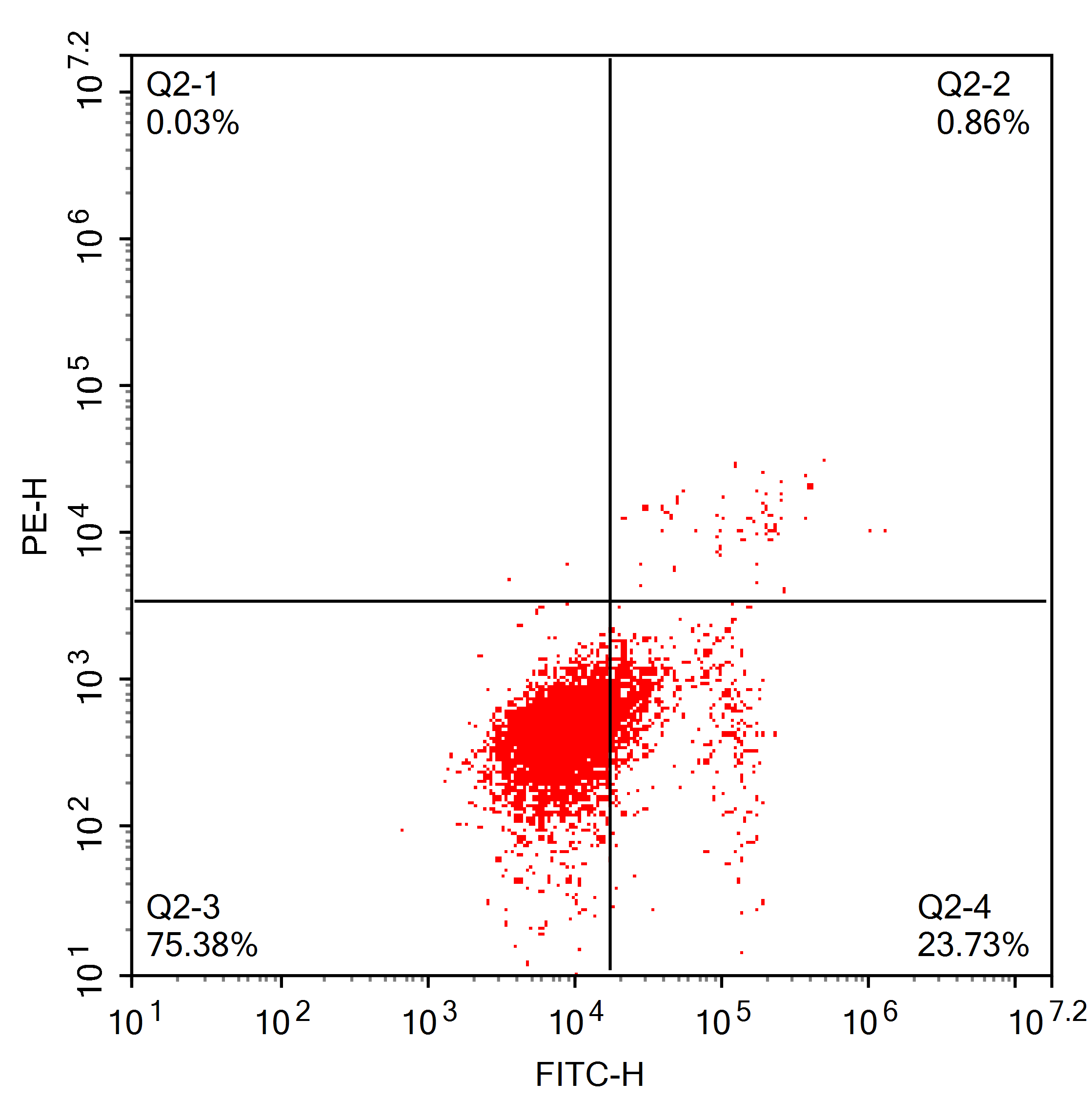

Supplement: Supplementary file 1 [file DataSheet3.zip › Flow Cytometry Assay(1,2)/Flow Cytometry Assay-1/╧╕░√╡≥═÷-1/╡≥═÷ 1/═╝╞1⁄4/8h 1/═╝2.tiff]

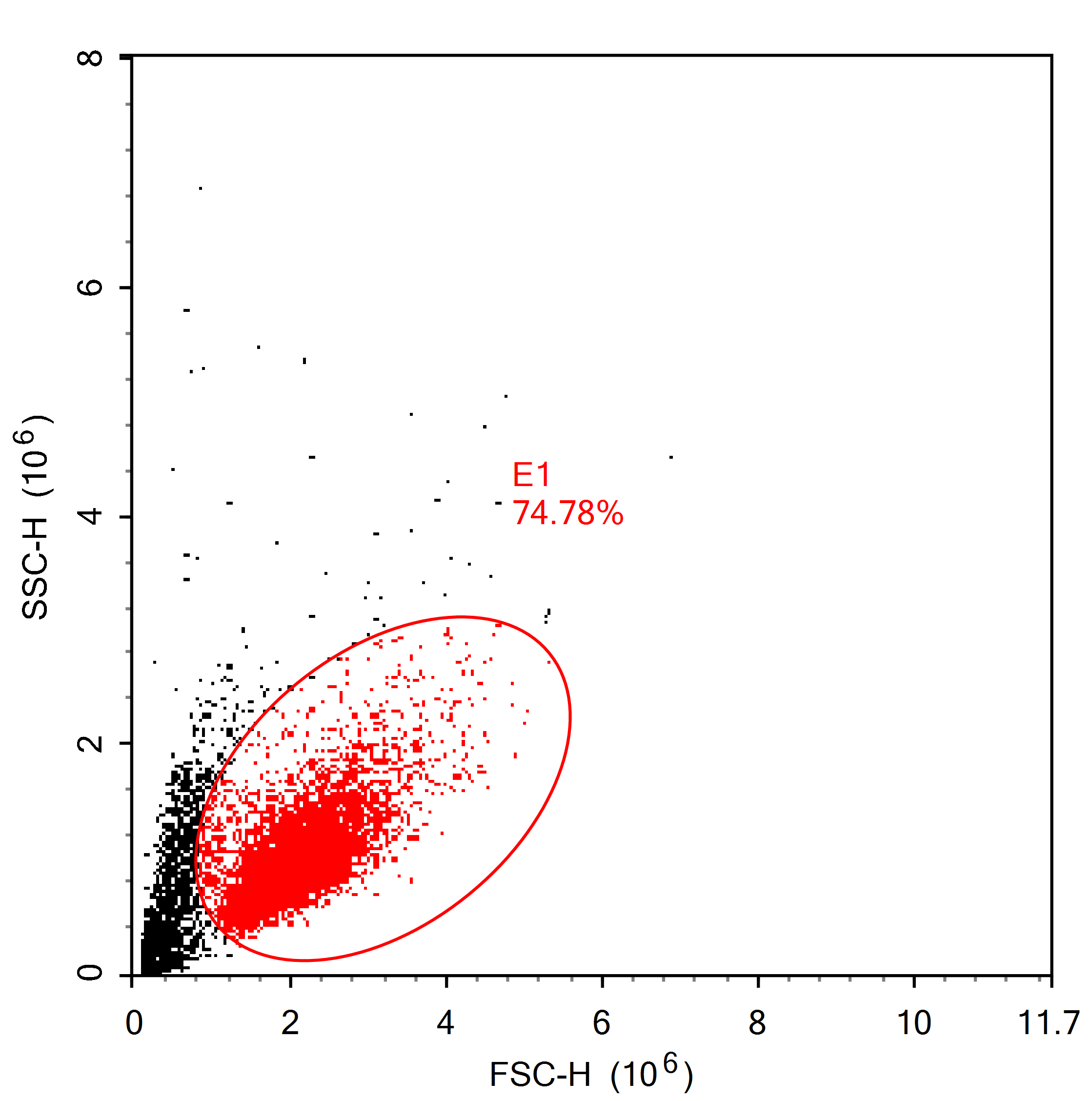

Supplement: Supplementary file 1 [file DataSheet3.zip › Flow Cytometry Assay(1,2)/Flow Cytometry Assay-1/╧╕░√╡≥═÷-1/╡≥═÷ 1/═╝╞1⁄4/8h 2/═╝1.tiff]

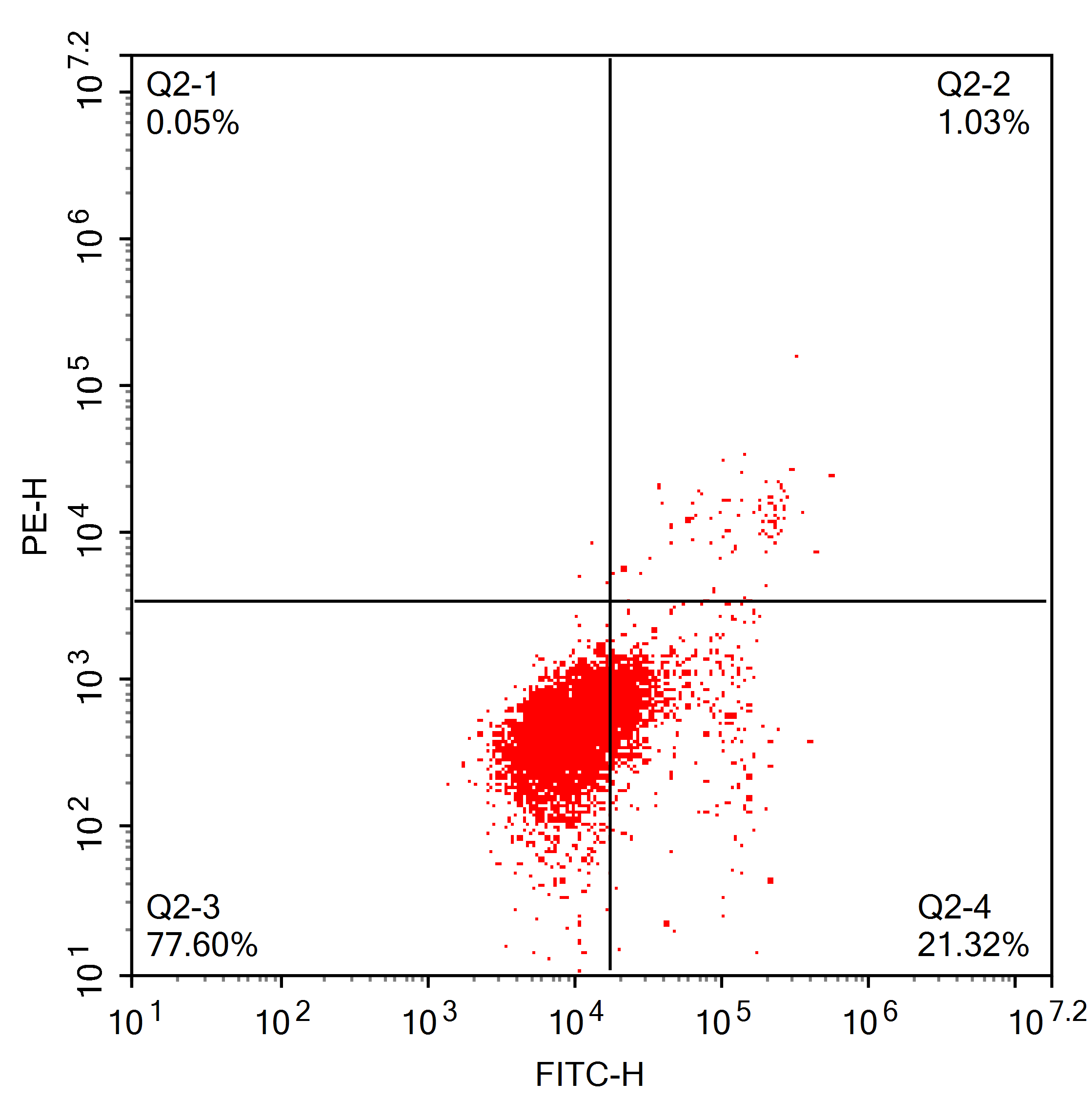

Supplement: Supplementary file 1 [file DataSheet3.zip › Flow Cytometry Assay(1,2)/Flow Cytometry Assay-1/╧╕░√╡≥═÷-1/╡≥═÷ 1/═╝╞1⁄4/8h 2/═╝2.tiff]

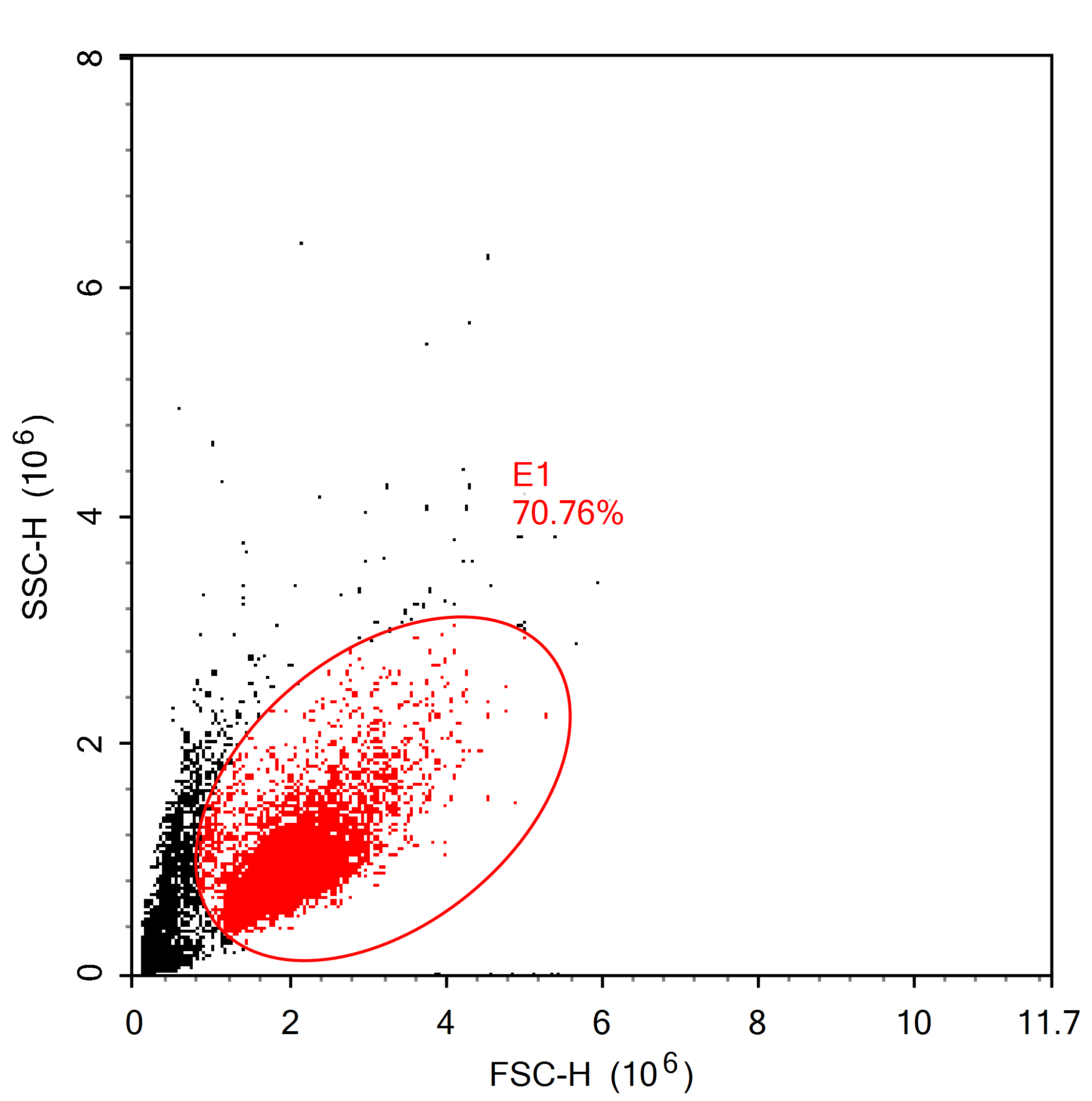

Supplement: Supplementary file 1 [file DataSheet3.zip › Flow Cytometry Assay(1,2)/Flow Cytometry Assay-1/╧╕░√╡≥═÷-1/╡≥═÷ 1/═╝╞1⁄4/8h 3/═╝1.tiff]

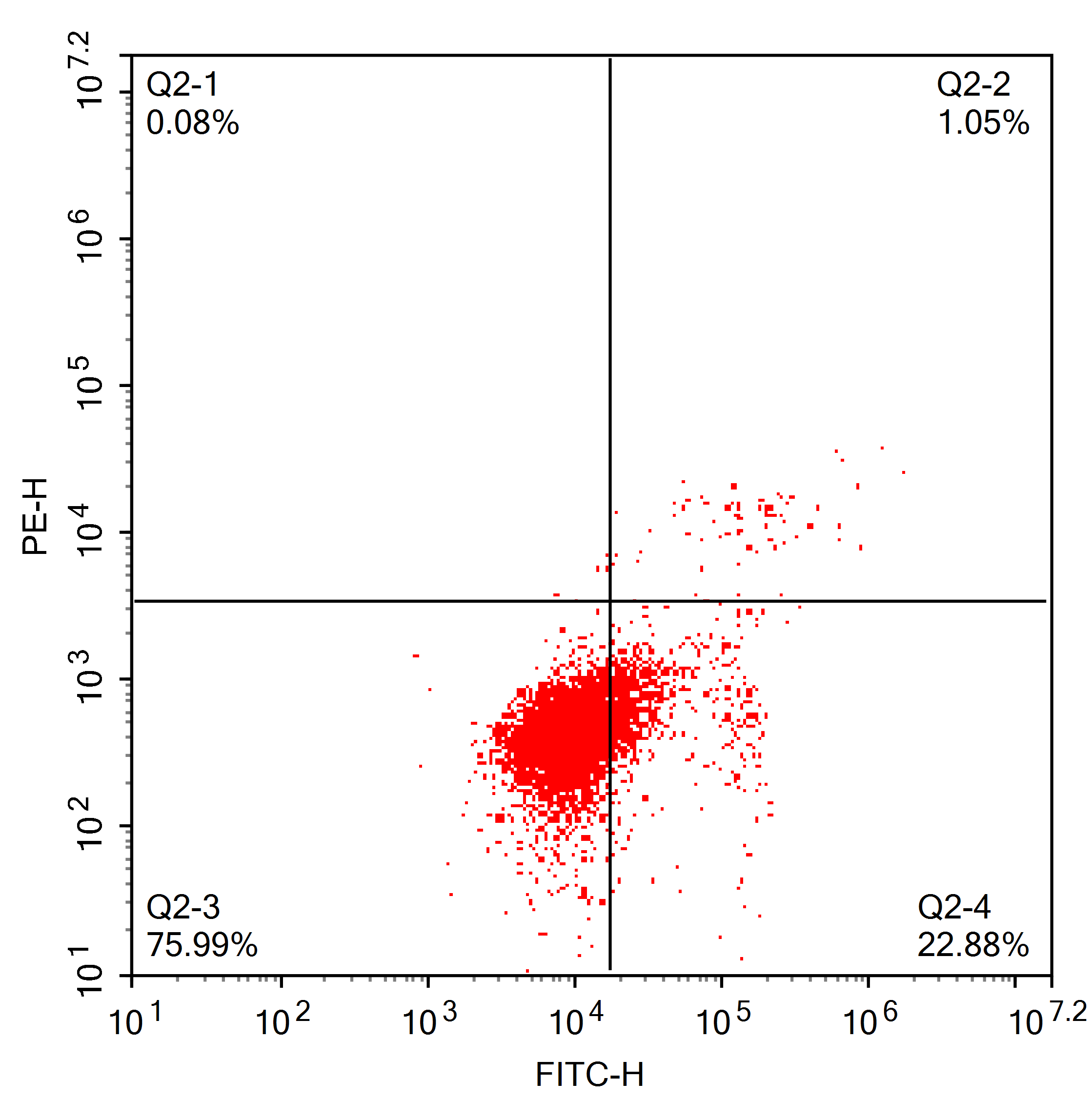

Supplement: Supplementary file 1 [file DataSheet3.zip › Flow Cytometry Assay(1,2)/Flow Cytometry Assay-1/╧╕░√╡≥═÷-1/╡≥═÷ 1/═╝╞1⁄4/8h 3/═╝2.tiff]

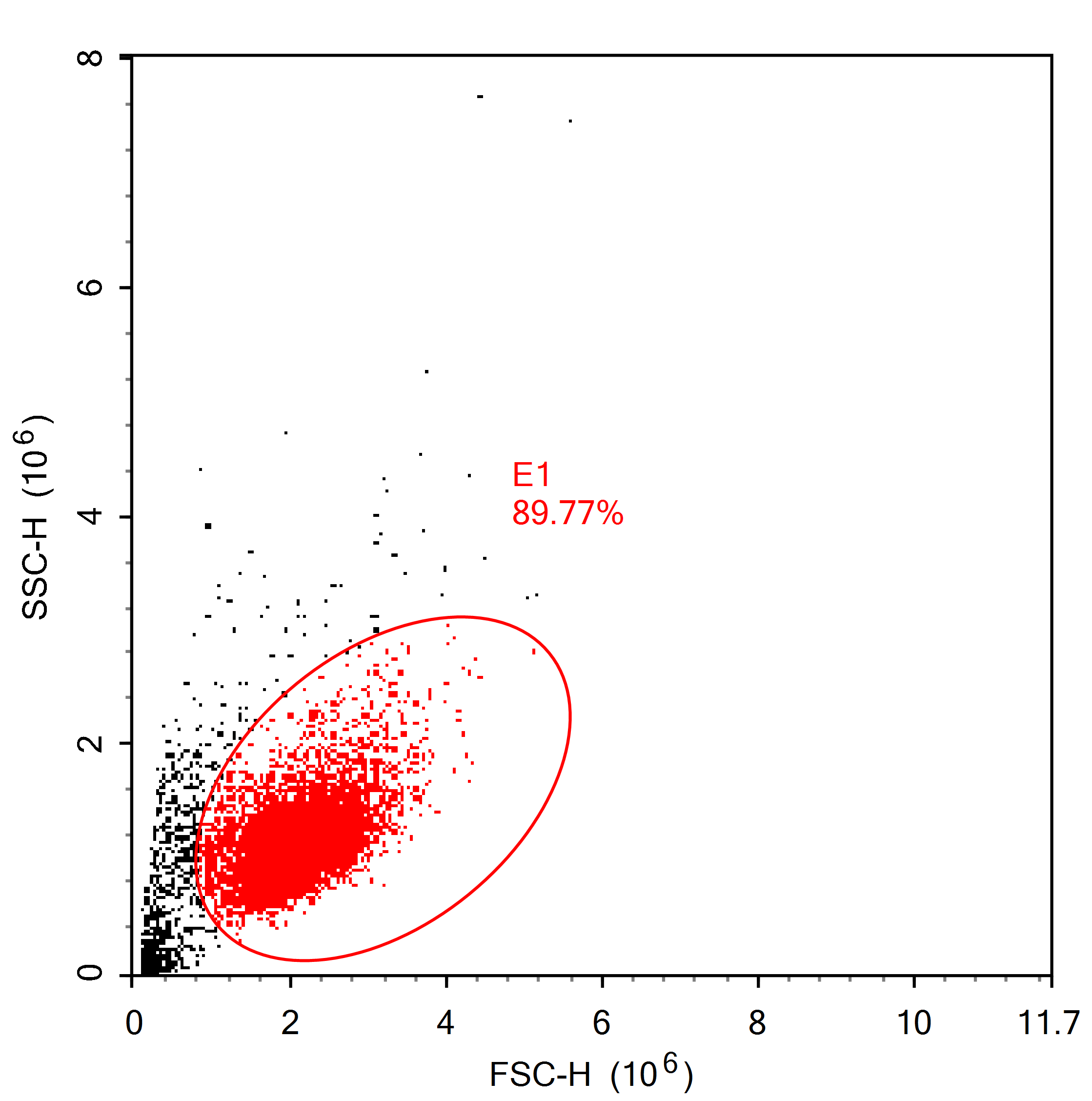

Supplement: Supplementary file 1 [file DataSheet3.zip › Flow Cytometry Assay(1,2)/Flow Cytometry Assay-1/╧╕░√╡≥═÷-1/╡≥═÷ 1/═╝╞1⁄4/Blank/═╝1.tiff]

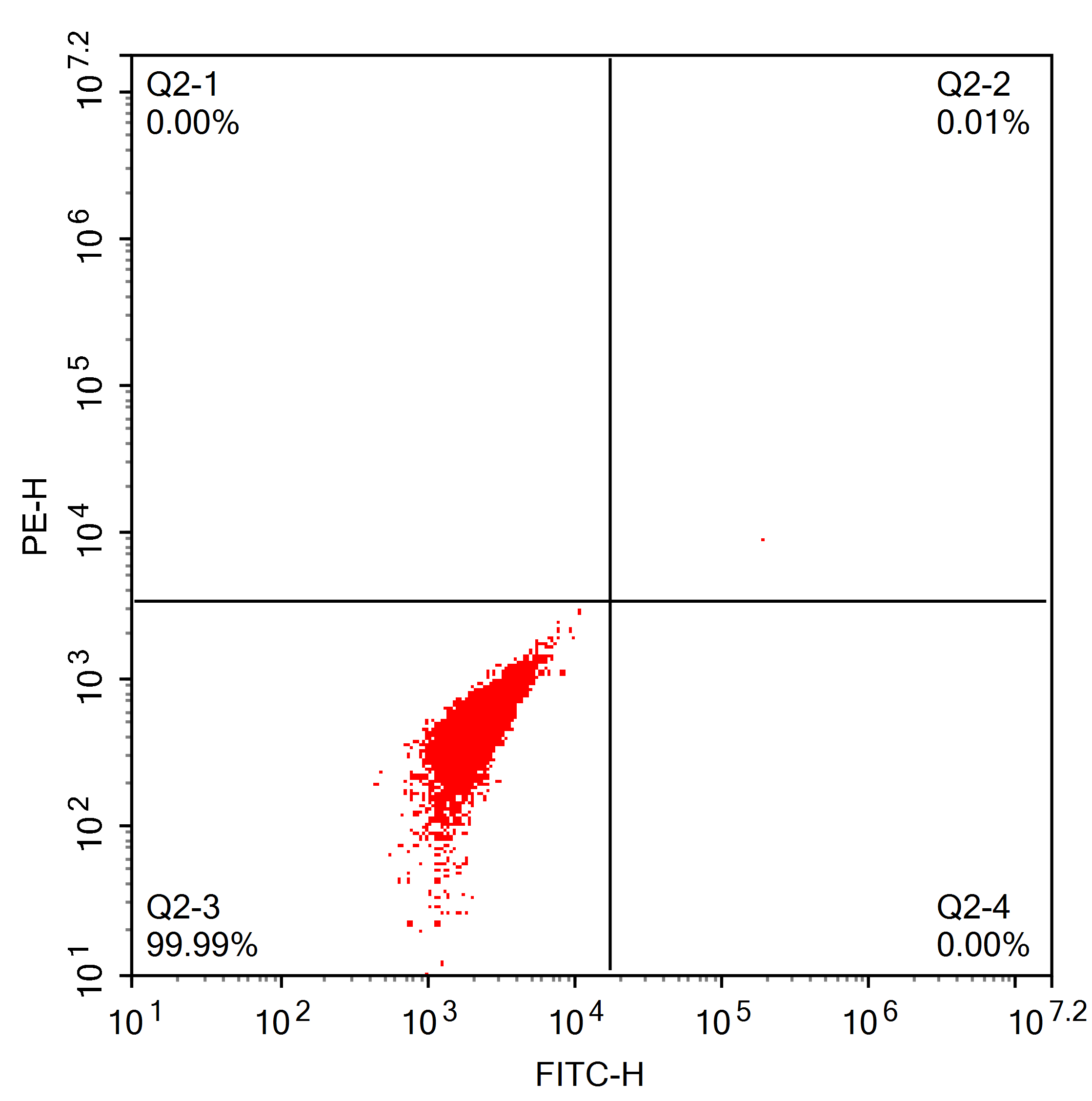

Supplement: Supplementary file 1 [file DataSheet3.zip › Flow Cytometry Assay(1,2)/Flow Cytometry Assay-1/╧╕░√╡≥═÷-1/╡≥═÷ 1/═╝╞1⁄4/Blank/═╝2.tiff]

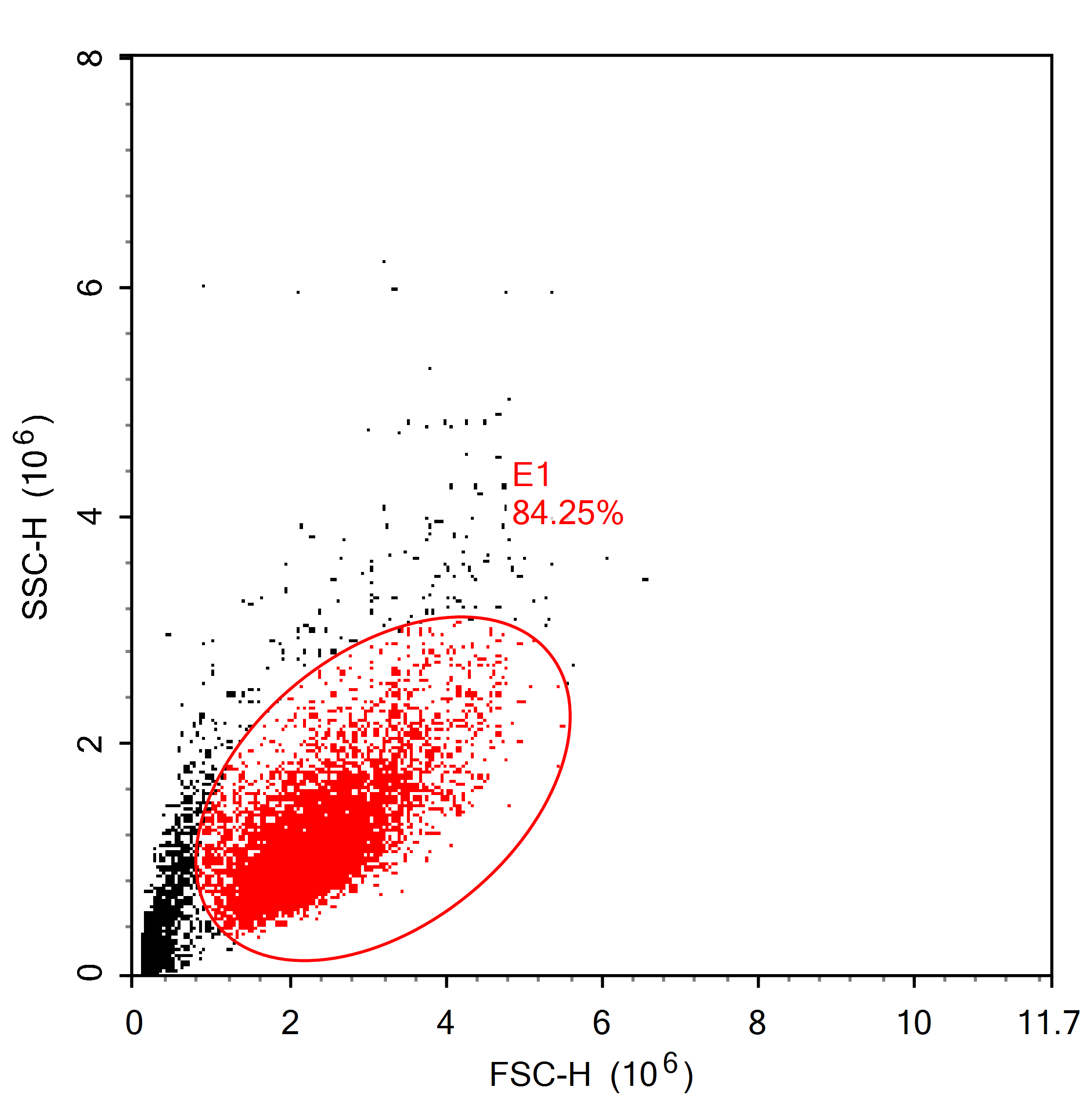

Supplement: Supplementary file 1 [file DataSheet3.zip › Flow Cytometry Assay(1,2)/Flow Cytometry Assay-1/╧╕░√╡≥═÷-1/╡≥═÷ 1/═╝╞1⁄4/Ctrl 1/═╝1.tiff]

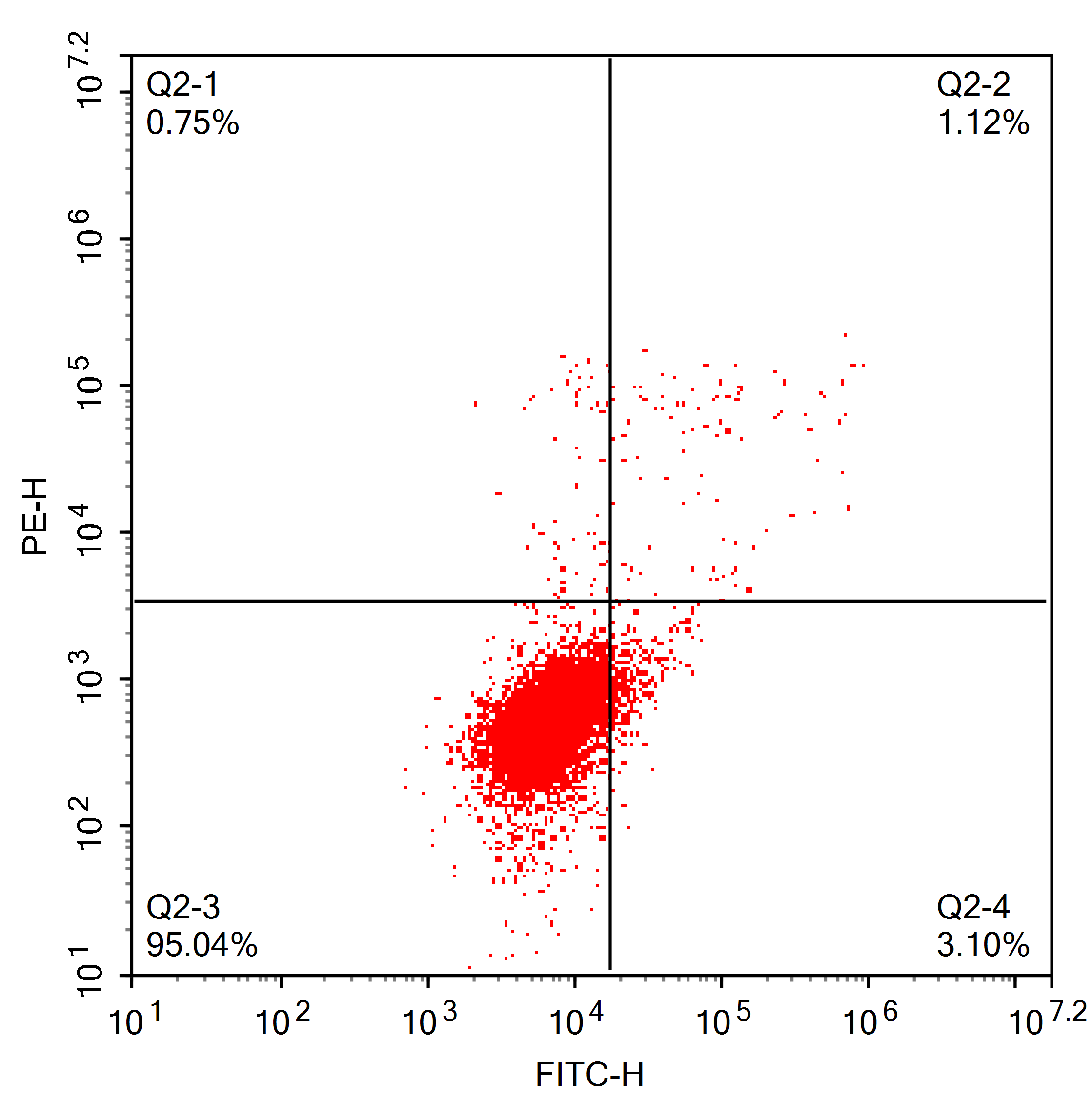

Supplement: Supplementary file 1 [file DataSheet3.zip › Flow Cytometry Assay(1,2)/Flow Cytometry Assay-1/╧╕░√╡≥═÷-1/╡≥═÷ 1/═╝╞1⁄4/Ctrl 1/═╝2.tiff]

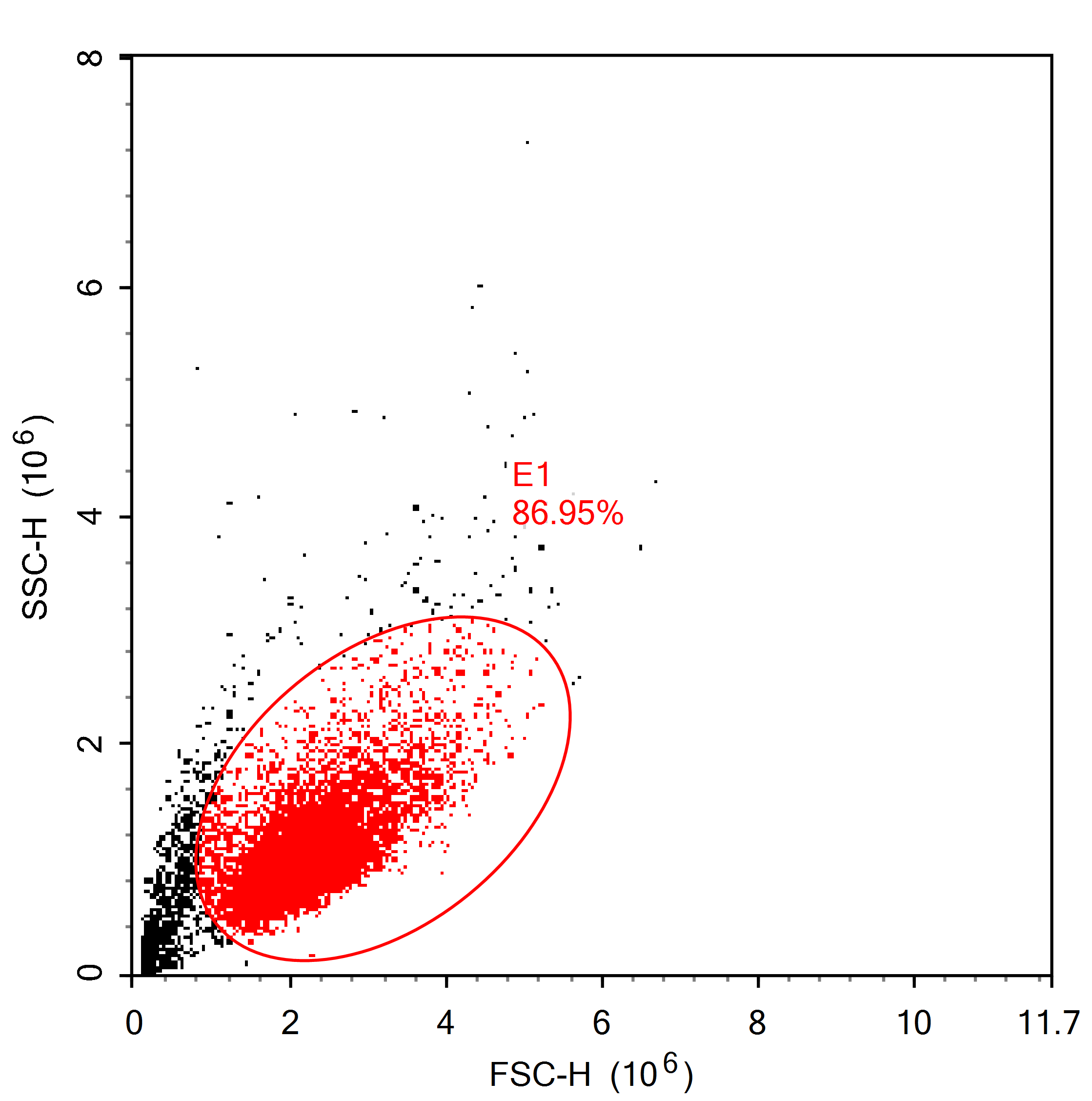

Supplement: Supplementary file 1 [file DataSheet3.zip › Flow Cytometry Assay(1,2)/Flow Cytometry Assay-1/╧╕░√╡≥═÷-1/╡≥═÷ 1/═╝╞1⁄4/Ctrl 2/═╝1.tiff]

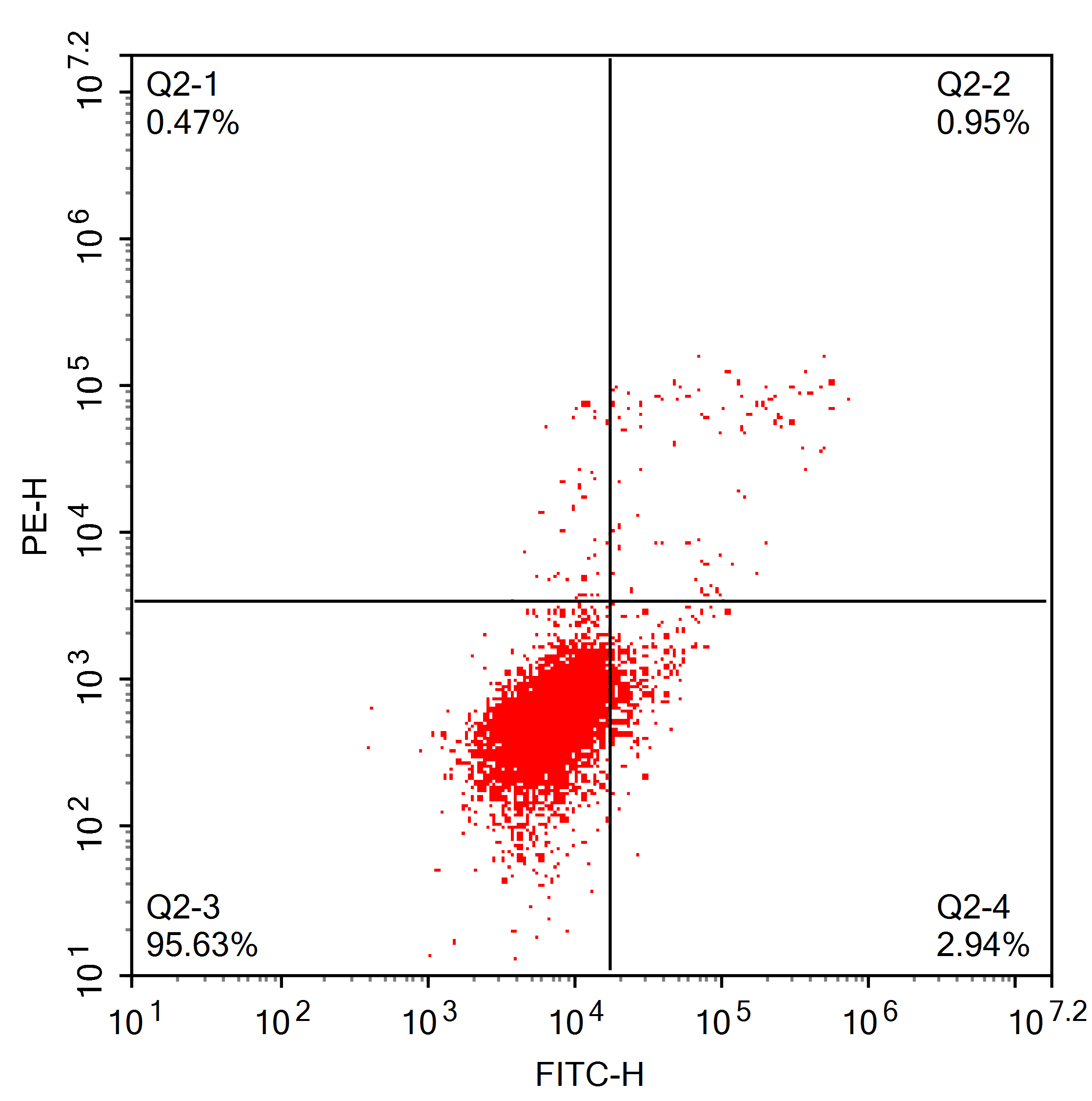

Supplement: Supplementary file 1 [file DataSheet3.zip › Flow Cytometry Assay(1,2)/Flow Cytometry Assay-1/╧╕░√╡≥═÷-1/╡≥═÷ 1/═╝╞1⁄4/Ctrl 2/═╝2.tiff]

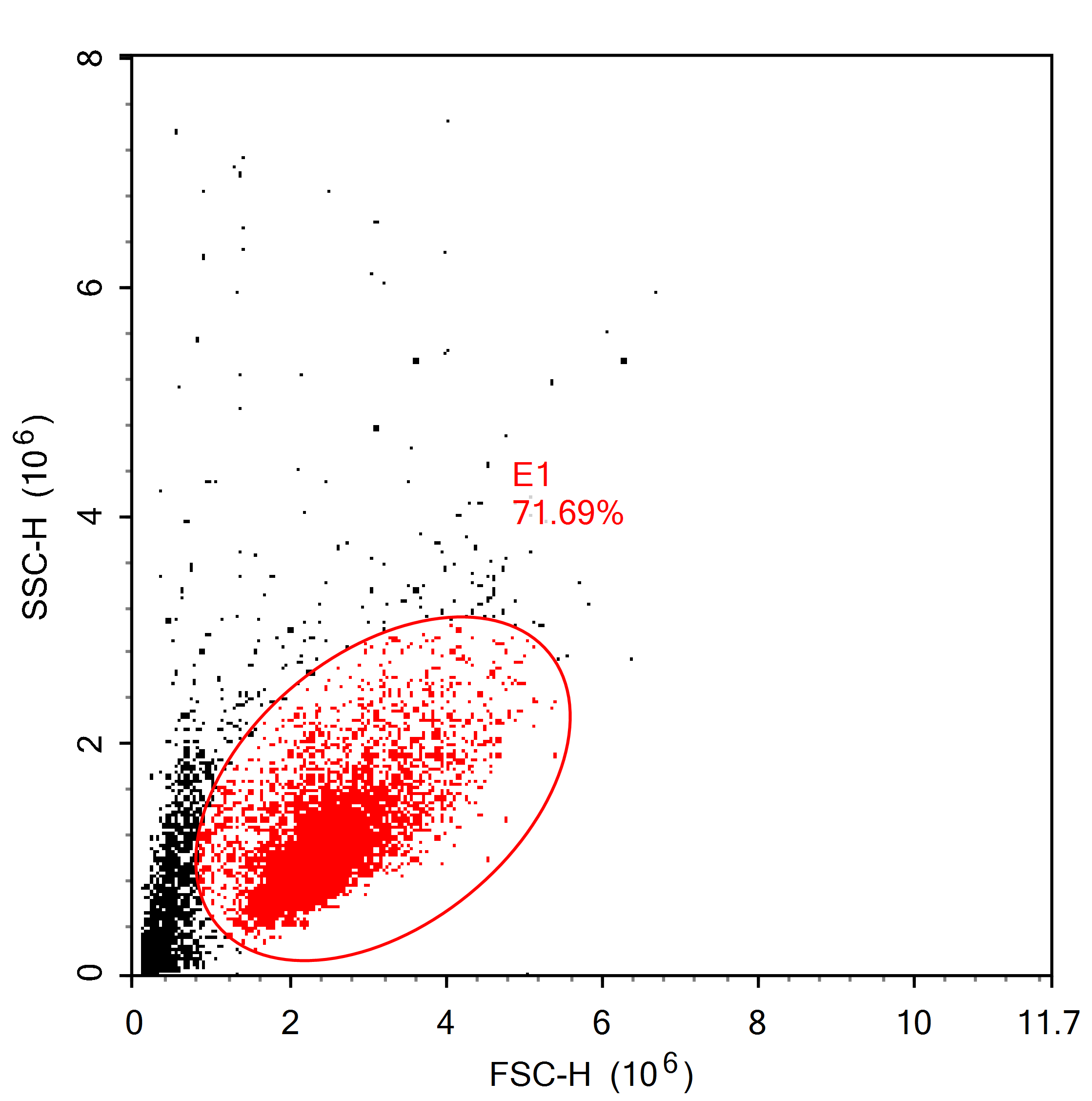

Supplement: Supplementary file 1 [file DataSheet3.zip › Flow Cytometry Assay(1,2)/Flow Cytometry Assay-1/╧╕░√╡≥═÷-1/╡≥═÷ 1/═╝╞1⁄4/Ctrl 3/═╝1.tiff]

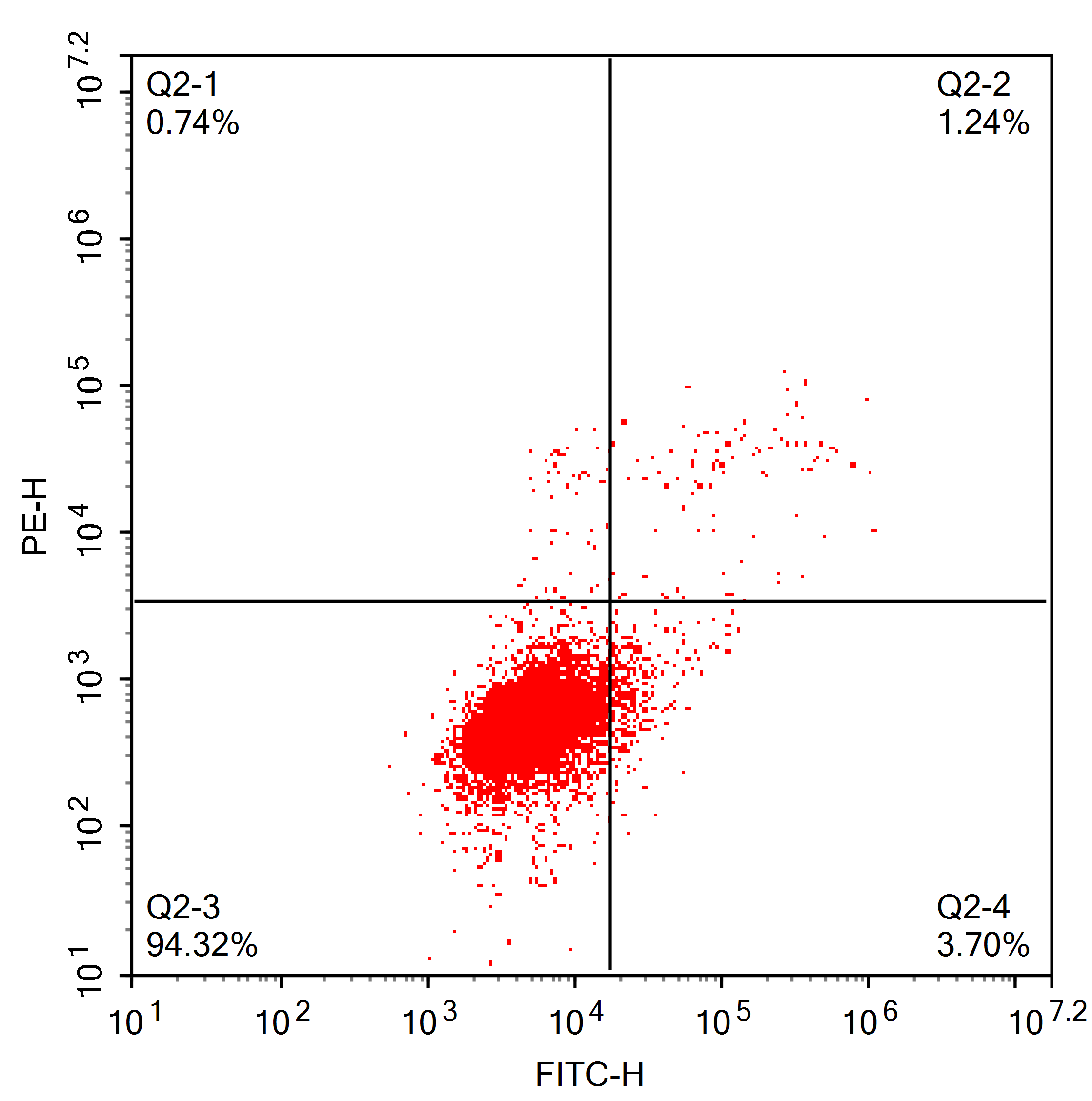

Supplement: Supplementary file 1 [file DataSheet3.zip › Flow Cytometry Assay(1,2)/Flow Cytometry Assay-1/╧╕░√╡≥═÷-1/╡≥═÷ 1/═╝╞1⁄4/Ctrl 3/═╝2.tiff]

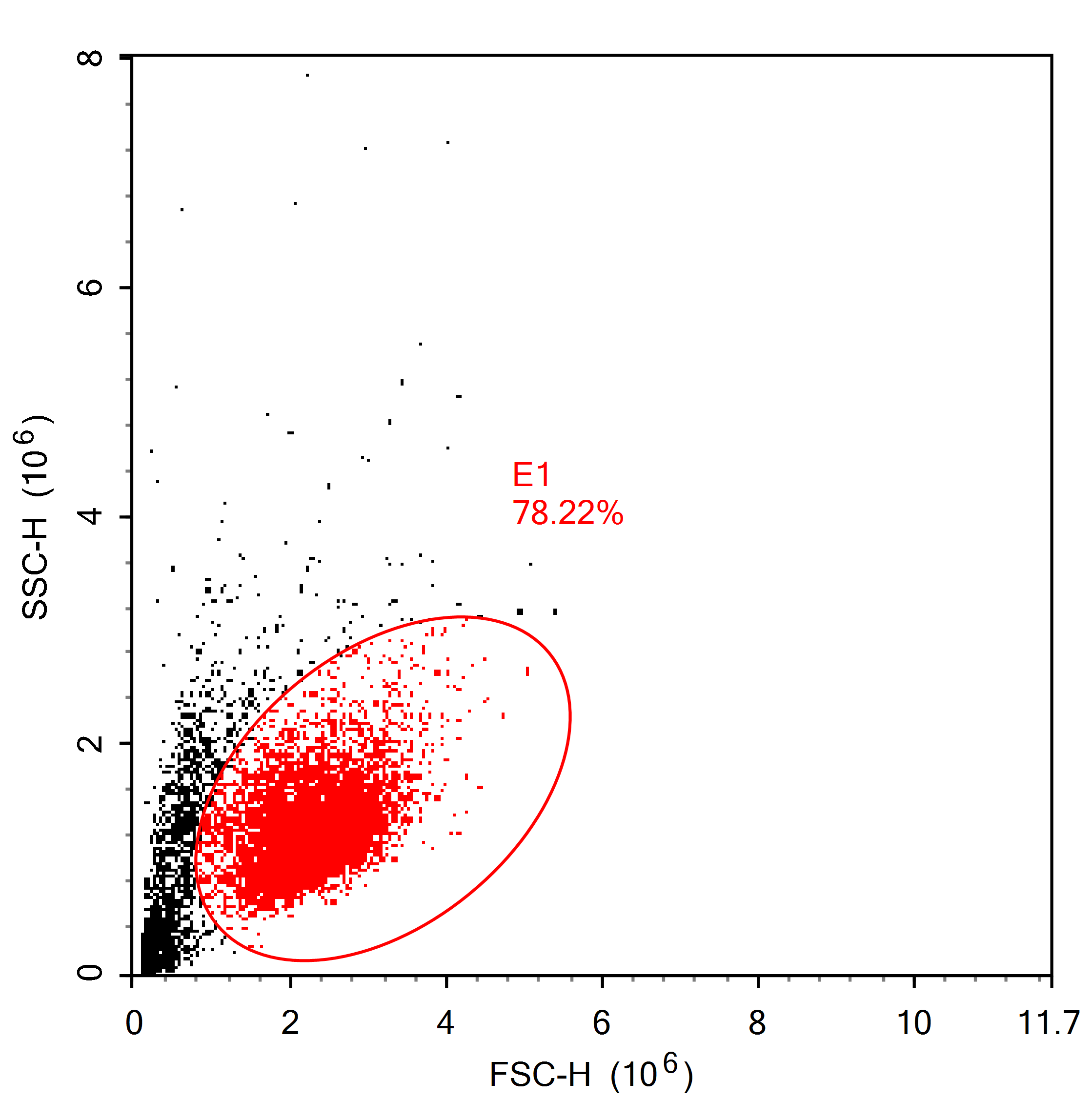

Supplement: Supplementary file 1 [file DataSheet3.zip › Flow Cytometry Assay(1,2)/Flow Cytometry Assay-1/╧╕░√╡≥═÷-1/╡≥═÷ 1/═╝╞1⁄4/FITC/═╝1.tiff]

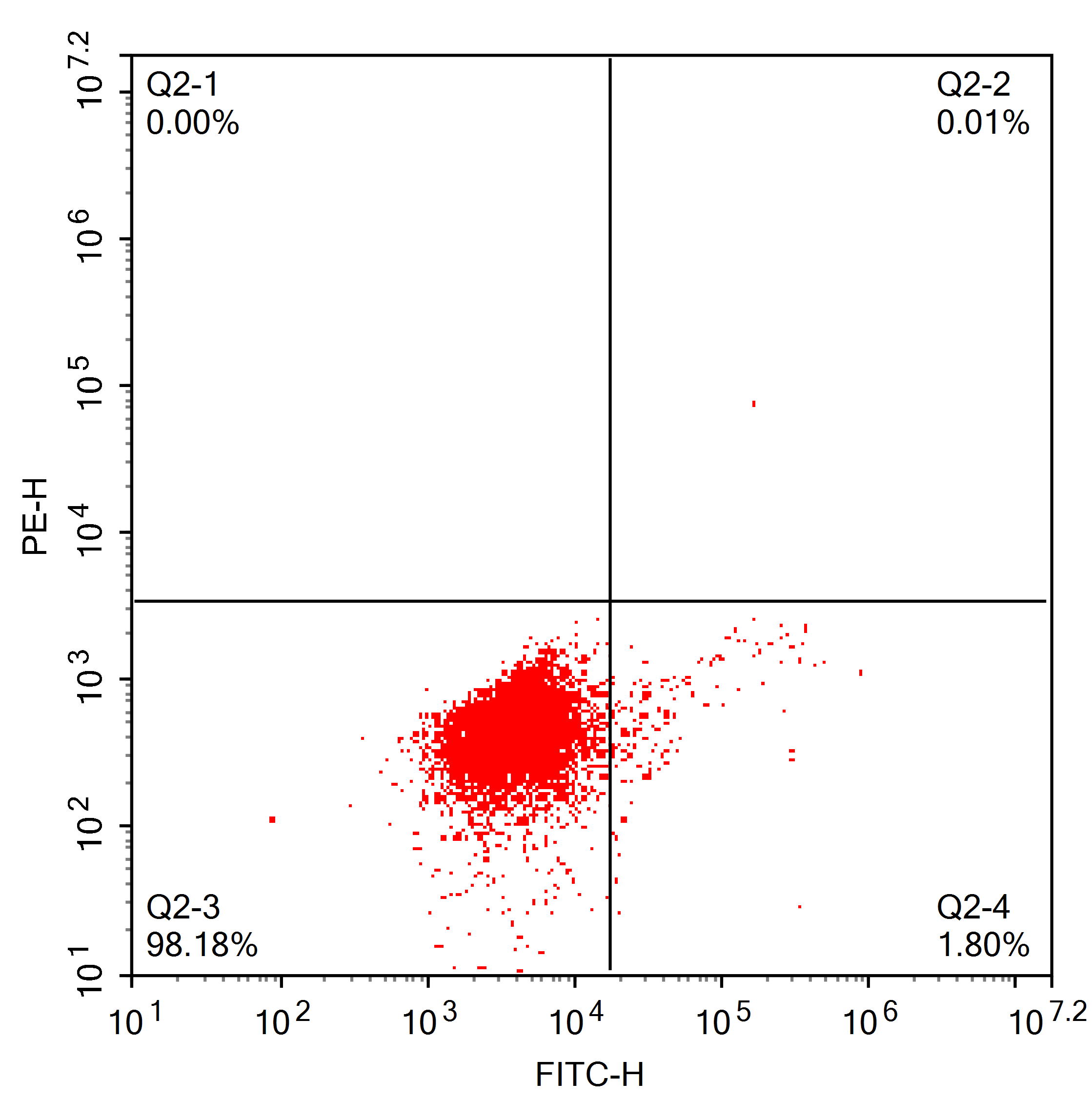

Supplement: Supplementary file 1 [file DataSheet3.zip › Flow Cytometry Assay(1,2)/Flow Cytometry Assay-1/╧╕░√╡≥═÷-1/╡≥═÷ 1/═╝╞1⁄4/FITC/═╝2.tiff]

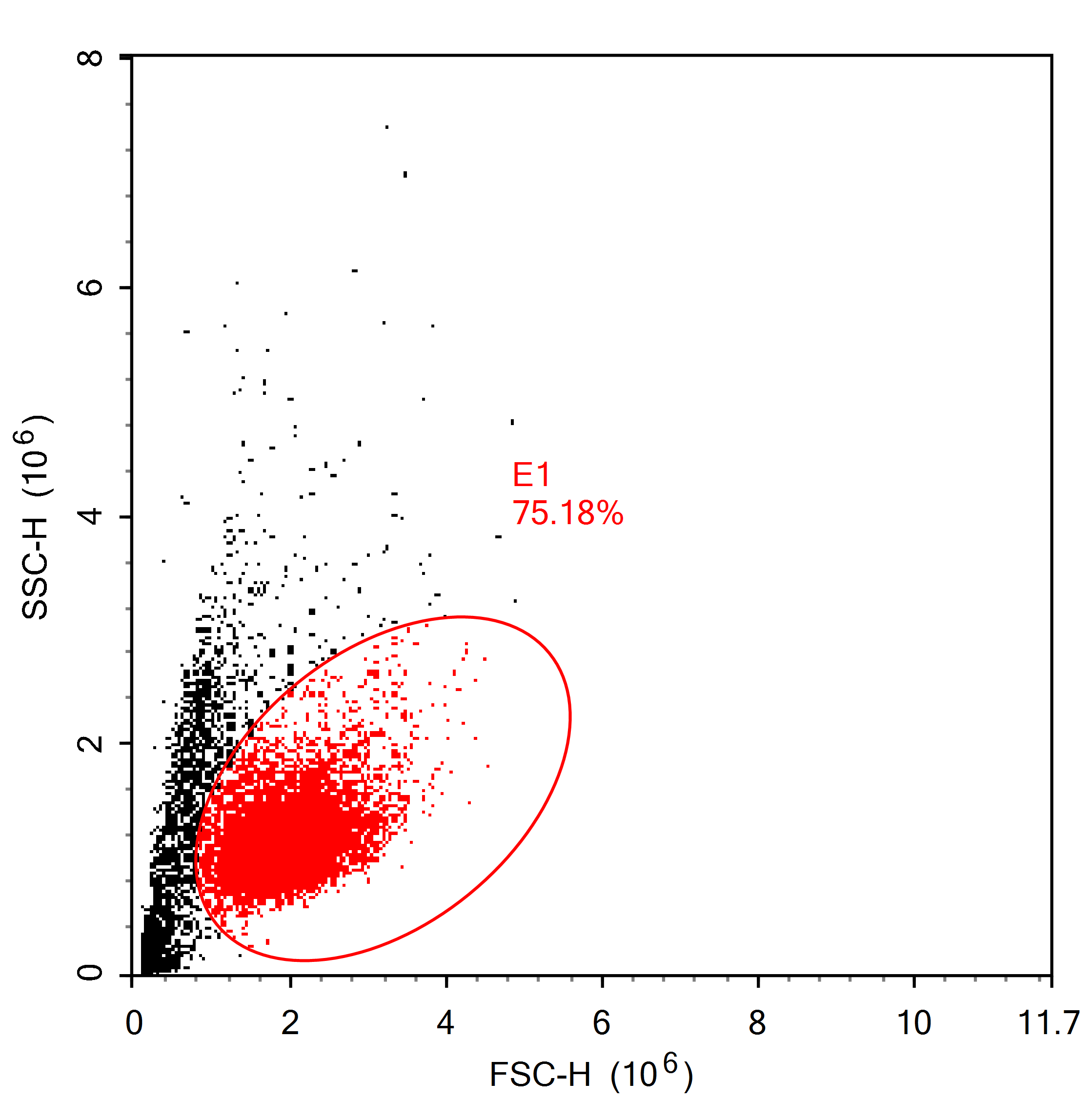

Supplement: Supplementary file 1 [file DataSheet3.zip › Flow Cytometry Assay(1,2)/Flow Cytometry Assay-1/╧╕░√╡≥═÷-1/╡≥═÷ 1/═╝╞1⁄4/PI/═╝1.tiff]

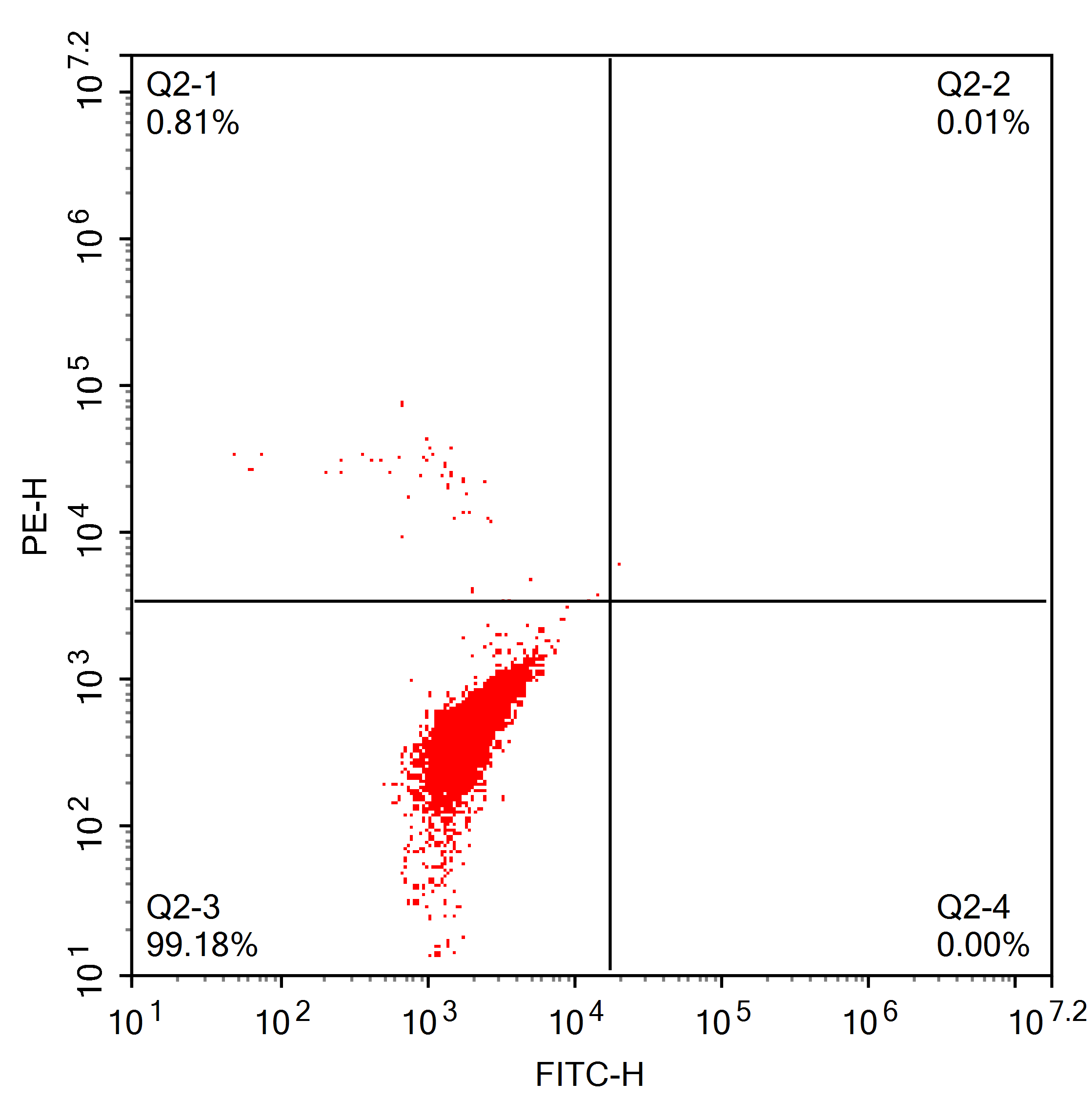

Supplement: Supplementary file 1 [file DataSheet3.zip › Flow Cytometry Assay(1,2)/Flow Cytometry Assay-1/╧╕░√╡≥═÷-1/╡≥═÷ 1/═╝╞1⁄4/PI/═╝2.tiff]

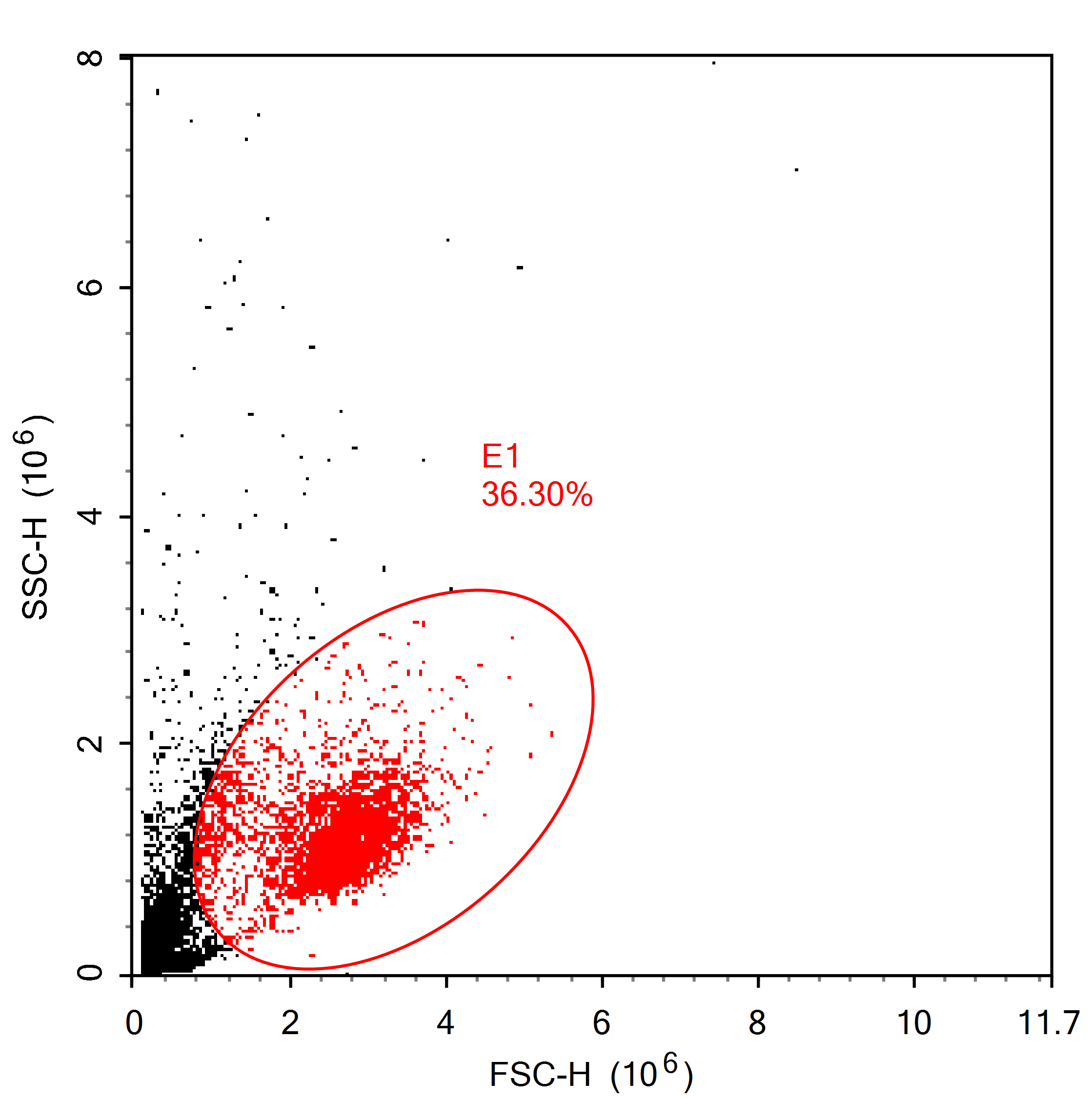

Supplement: Supplementary file 1 [file DataSheet3.zip › Flow Cytometry Assay(1,2)/Flow Cytometry Assay-1/╧╕░√╡≥═÷-1/╡≥═÷ 2/═╝╞1⁄4/12h 1/═╝1.tiff]

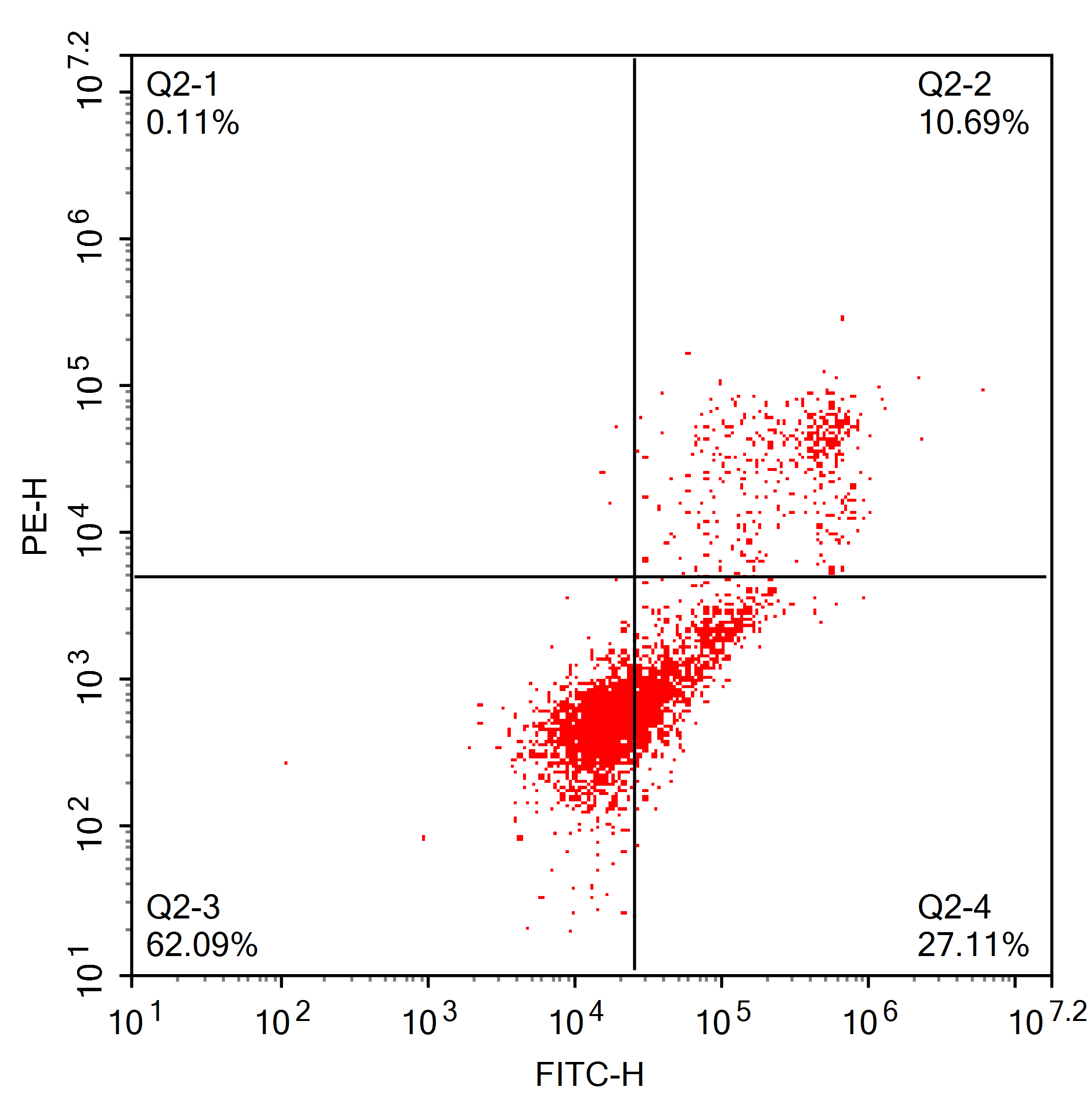

Supplement: Supplementary file 1 [file DataSheet3.zip › Flow Cytometry Assay(1,2)/Flow Cytometry Assay-1/╧╕░√╡≥═÷-1/╡≥═÷ 2/═╝╞1⁄4/12h 1/═╝2.tiff]

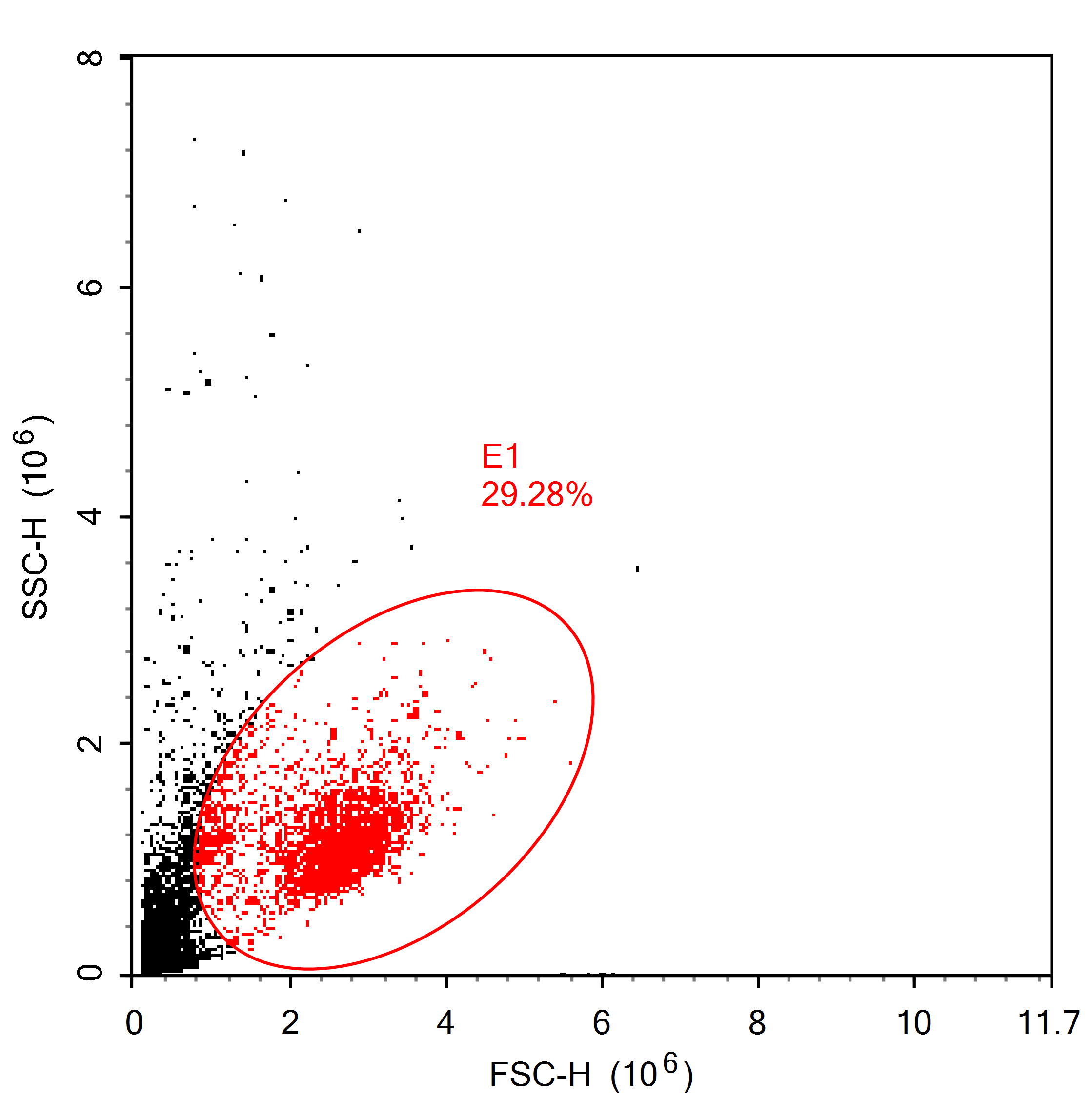

Supplement: Supplementary file 1 [file DataSheet3.zip › Flow Cytometry Assay(1,2)/Flow Cytometry Assay-1/╧╕░√╡≥═÷-1/╡≥═÷ 2/═╝╞1⁄4/12h 2/═╝1.tiff]

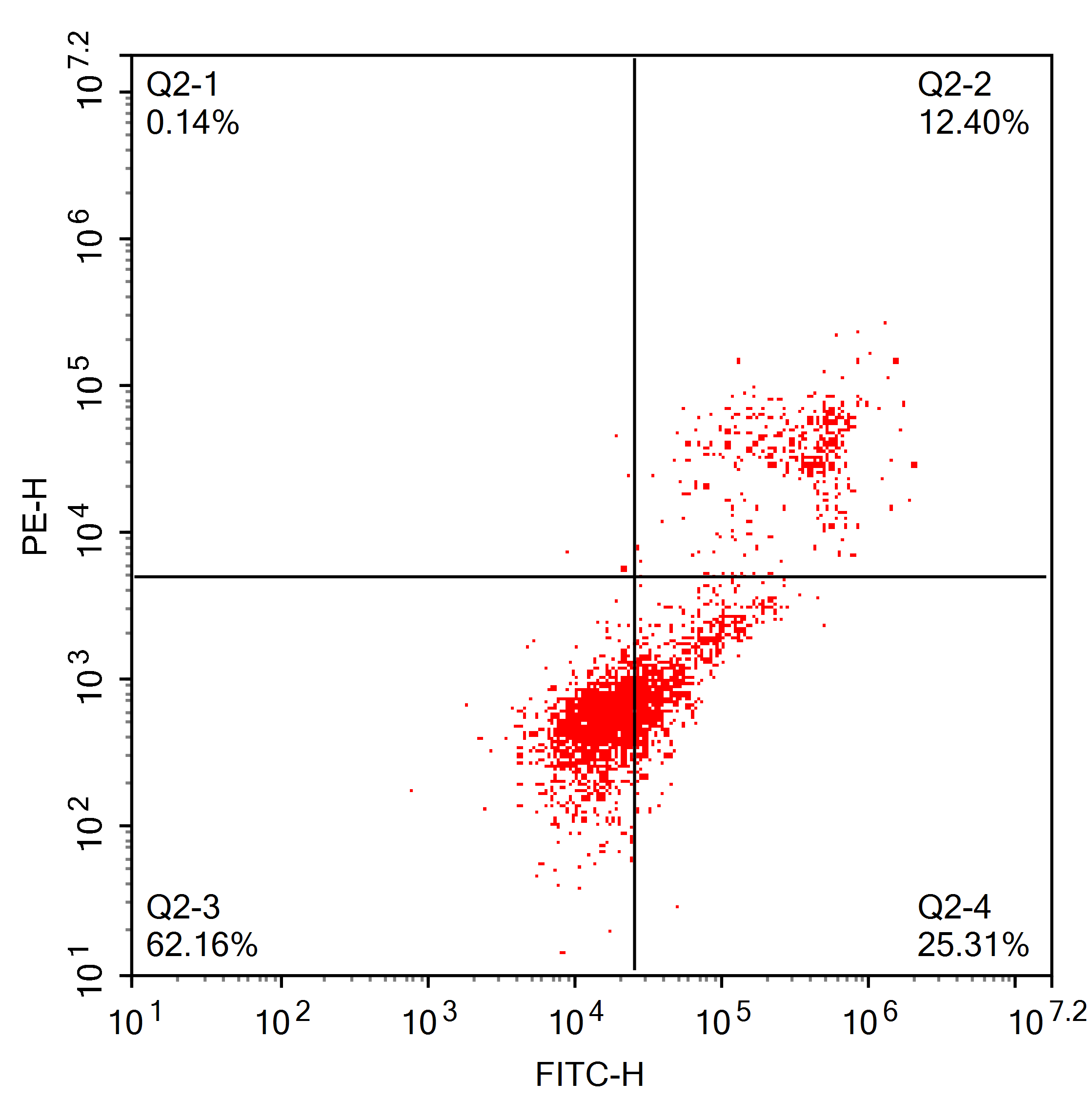

Supplement: Supplementary file 1 [file DataSheet3.zip › Flow Cytometry Assay(1,2)/Flow Cytometry Assay-1/╧╕░√╡≥═÷-1/╡≥═÷ 2/═╝╞1⁄4/12h 2/═╝2.tiff]

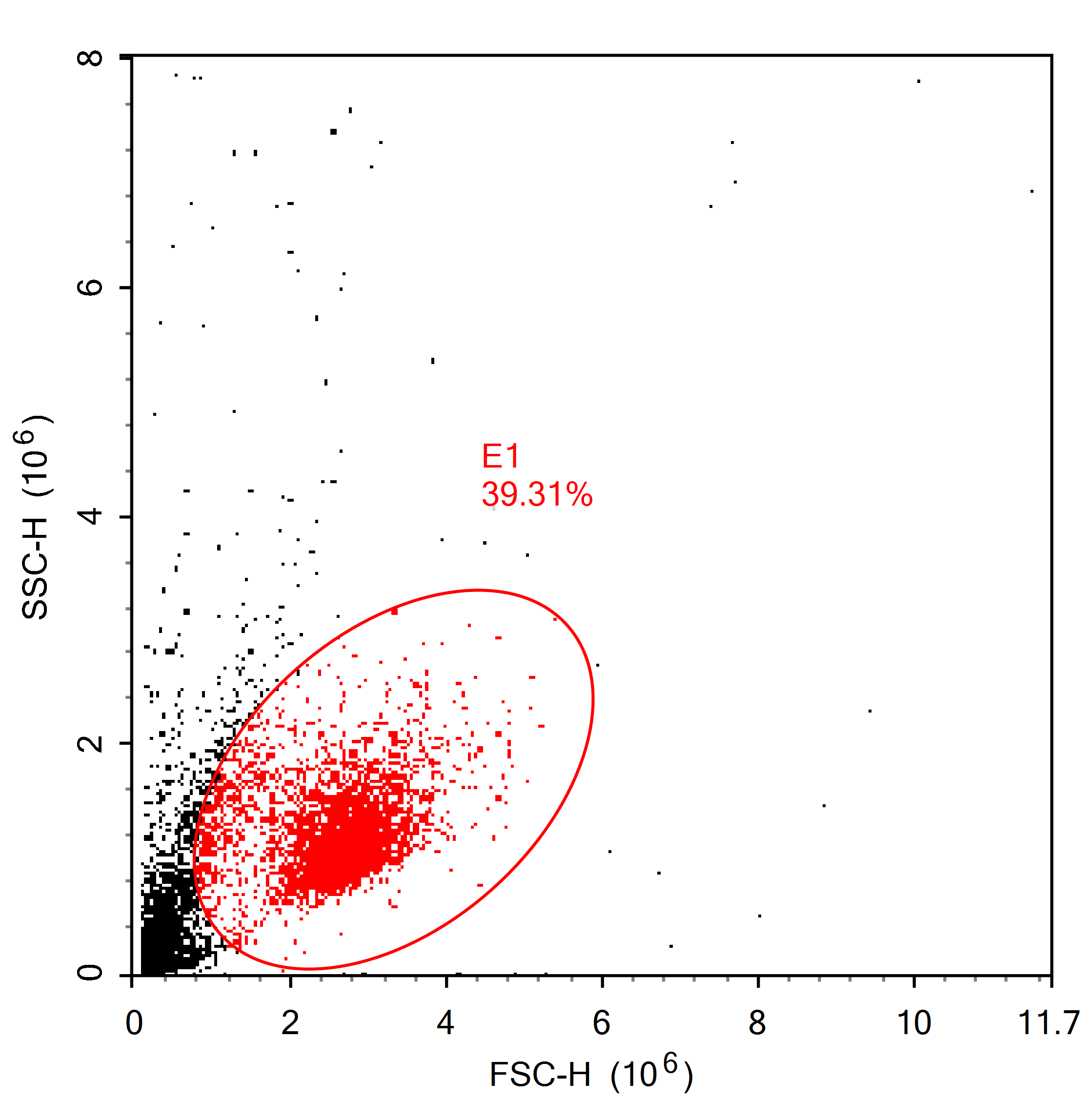

Supplement: Supplementary file 1 [file DataSheet3.zip › Flow Cytometry Assay(1,2)/Flow Cytometry Assay-1/╧╕░√╡≥═÷-1/╡≥═÷ 2/═╝╞1⁄4/12h 3/═╝1.tiff]

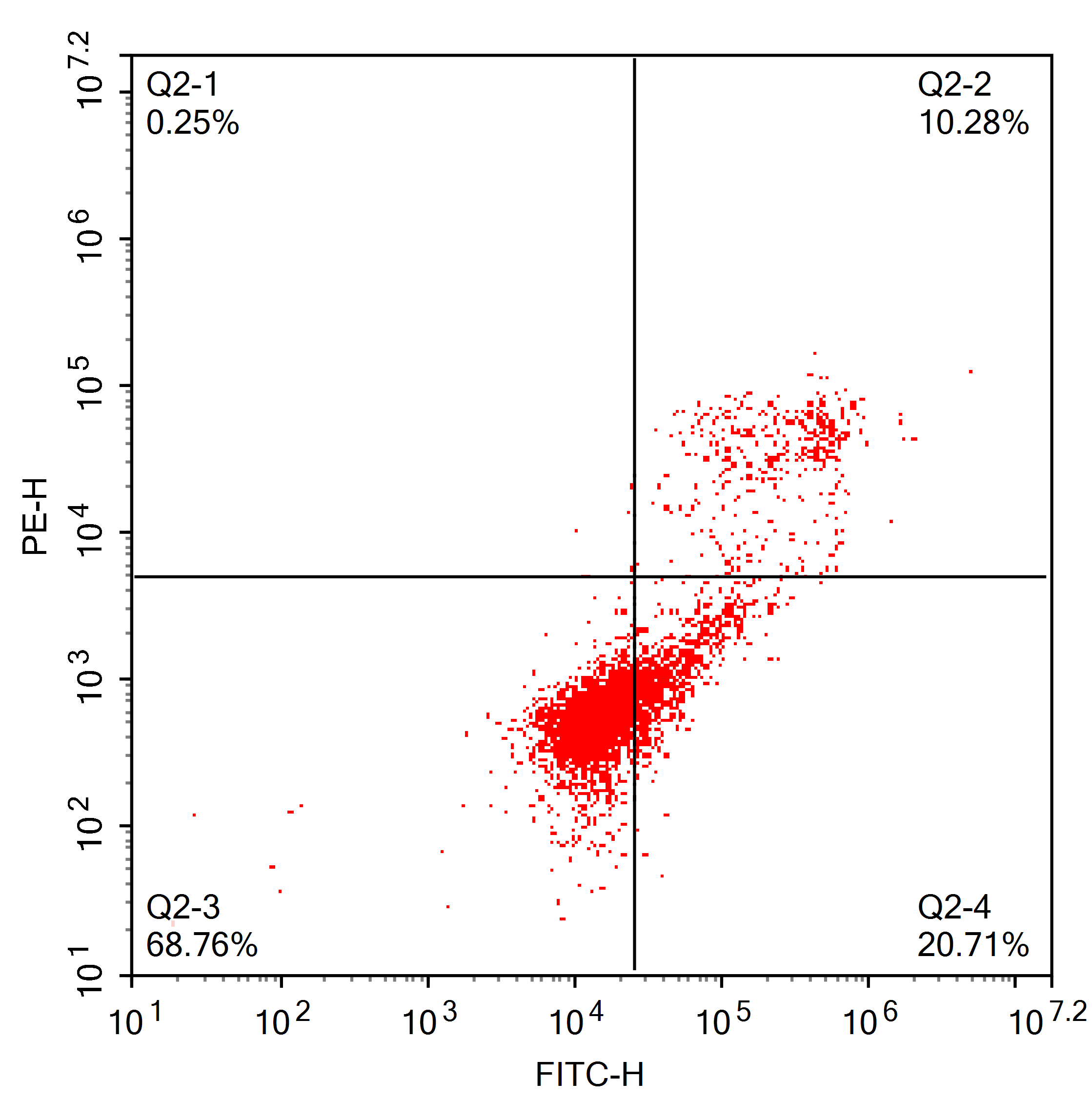

Supplement: Supplementary file 1 [file DataSheet3.zip › Flow Cytometry Assay(1,2)/Flow Cytometry Assay-1/╧╕░√╡≥═÷-1/╡≥═÷ 2/═╝╞1⁄4/12h 3/═╝2.tiff]

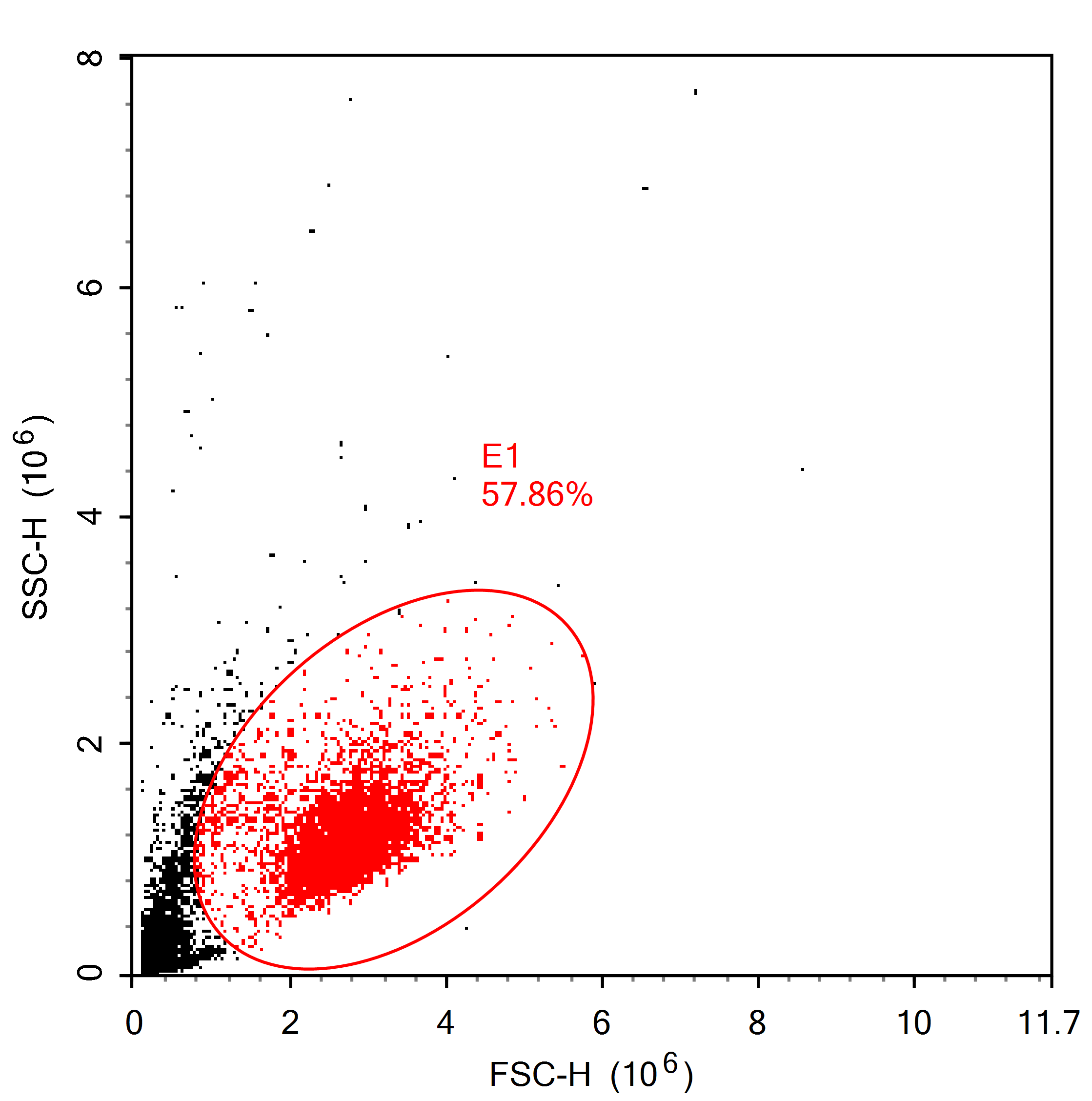

Supplement: Supplementary file 1 [file DataSheet3.zip › Flow Cytometry Assay(1,2)/Flow Cytometry Assay-1/╧╕░√╡≥═÷-1/╡≥═÷ 2/═╝╞1⁄4/4h 1/═╝1.tiff]

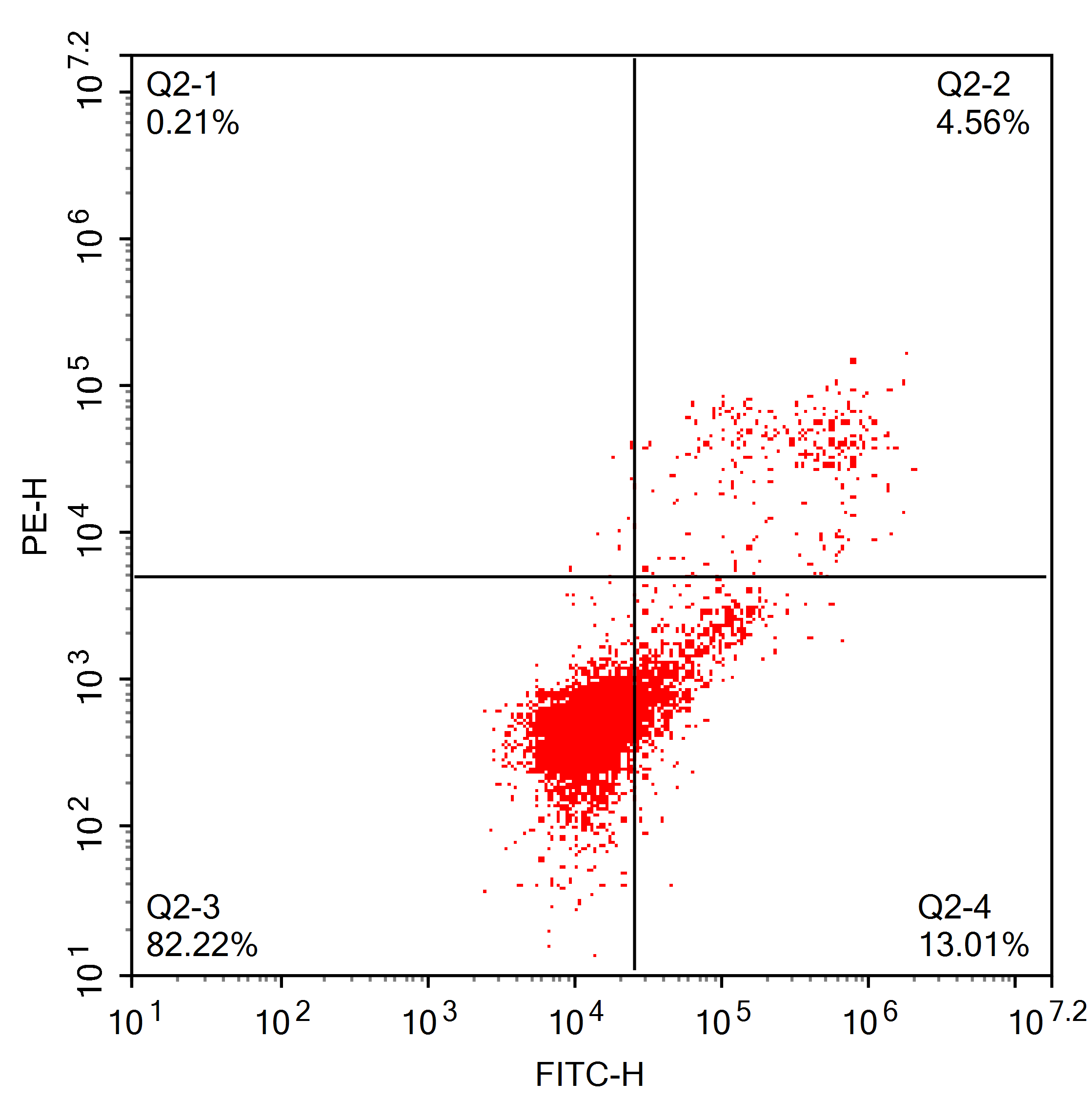

Supplement: Supplementary file 1 [file DataSheet3.zip › Flow Cytometry Assay(1,2)/Flow Cytometry Assay-1/╧╕░√╡≥═÷-1/╡≥═÷ 2/═╝╞1⁄4/4h 1/═╝2.tiff]

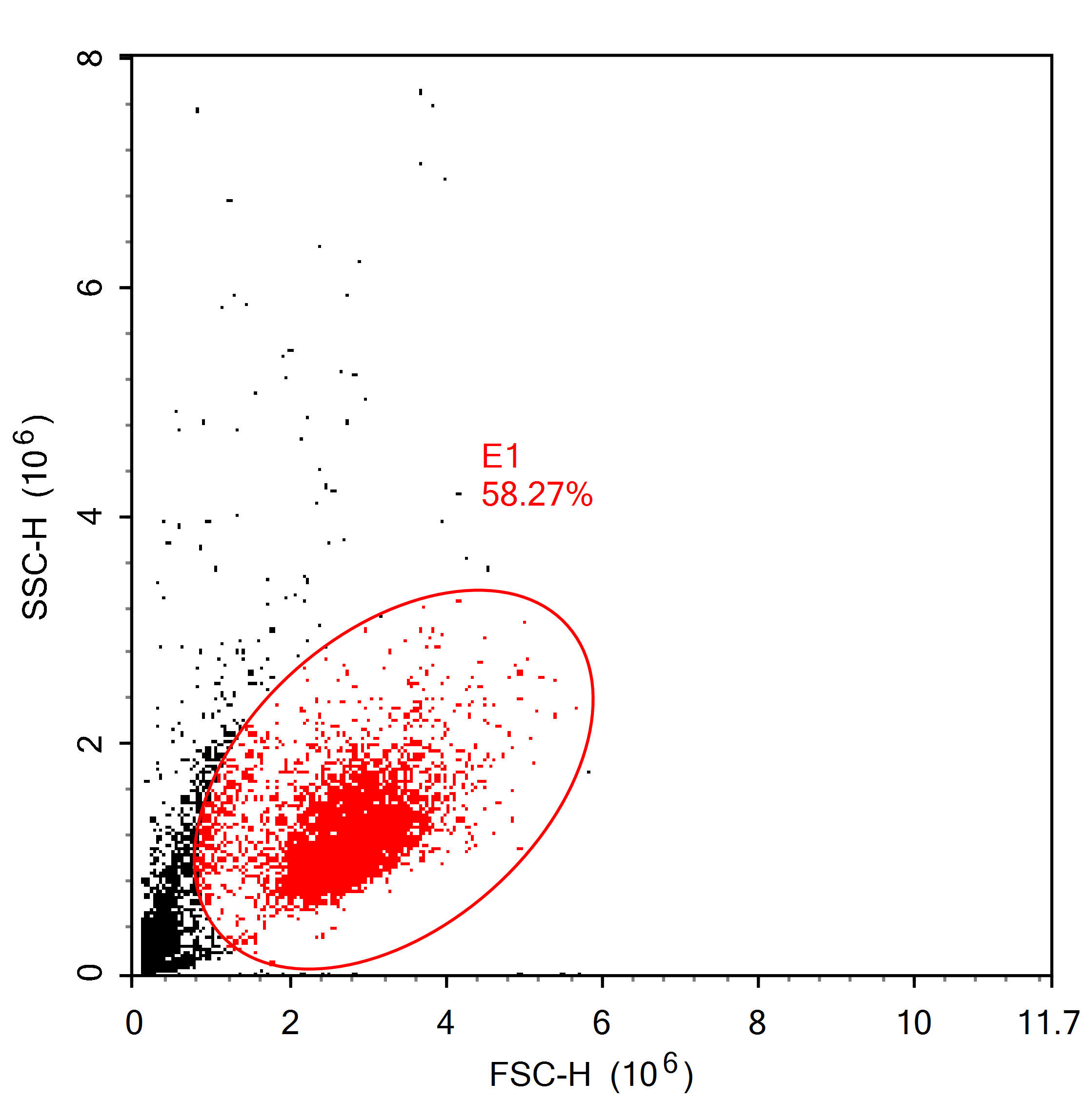

Supplement: Supplementary file 1 [file DataSheet3.zip › Flow Cytometry Assay(1,2)/Flow Cytometry Assay-1/╧╕░√╡≥═÷-1/╡≥═÷ 2/═╝╞1⁄4/4h 2/═╝1.tiff]

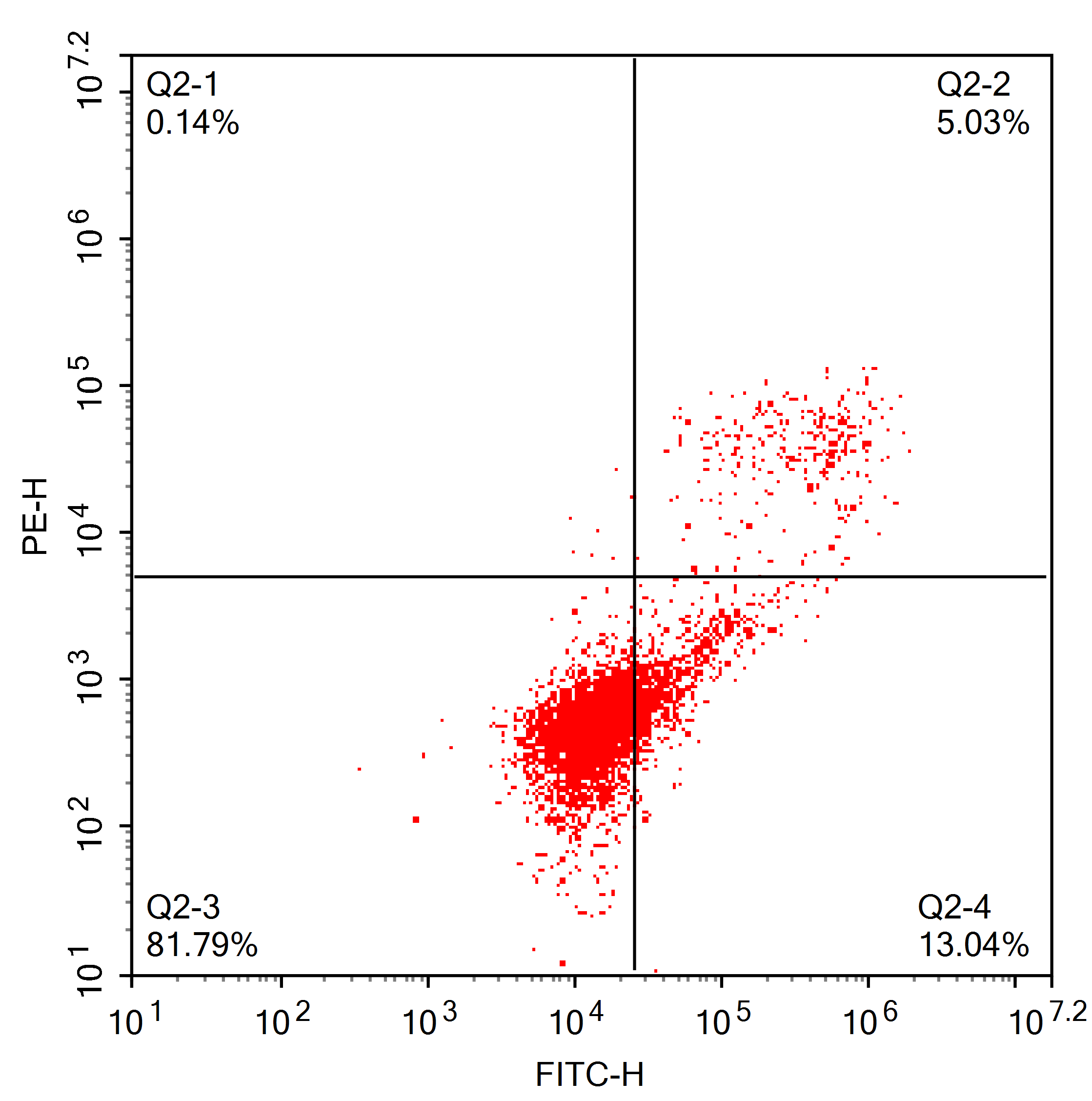

Supplement: Supplementary file 1 [file DataSheet3.zip › Flow Cytometry Assay(1,2)/Flow Cytometry Assay-1/╧╕░√╡≥═÷-1/╡≥═÷ 2/═╝╞1⁄4/4h 2/═╝2.tiff]

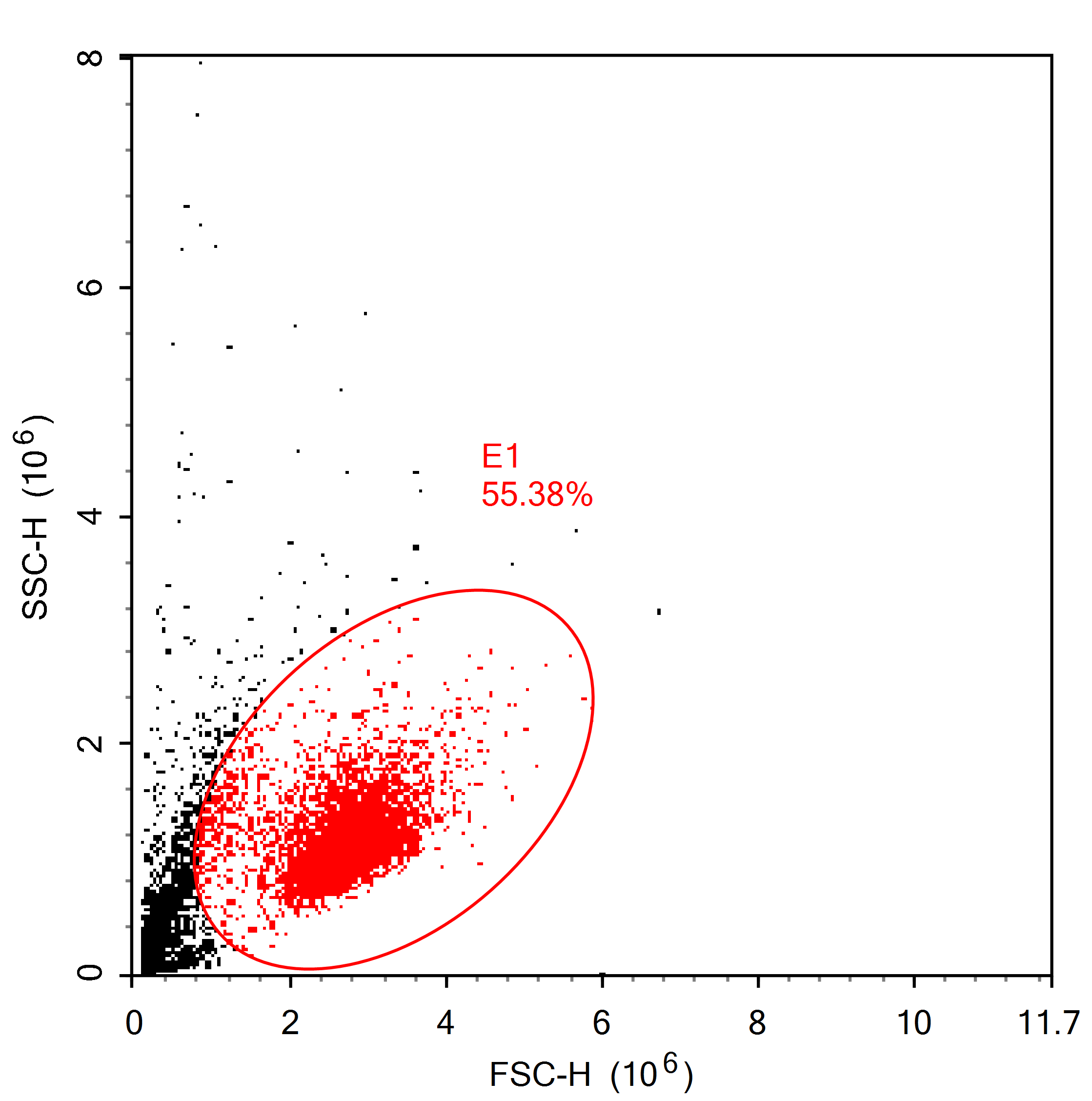

Supplement: Supplementary file 1 [file DataSheet3.zip › Flow Cytometry Assay(1,2)/Flow Cytometry Assay-1/╧╕░√╡≥═÷-1/╡≥═÷ 2/═╝╞1⁄4/4h 3/═╝1.tiff]

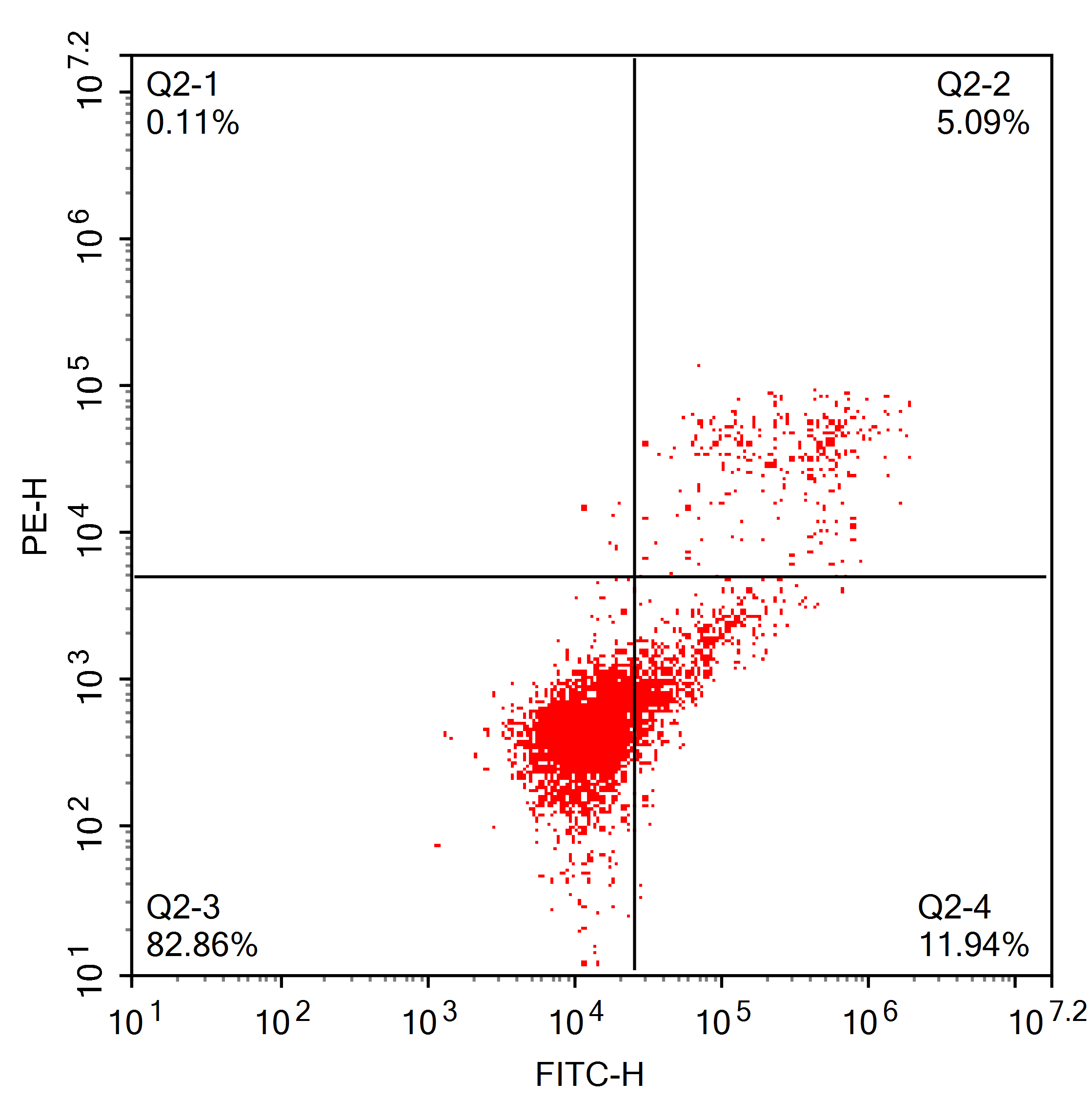

Supplement: Supplementary file 1 [file DataSheet3.zip › Flow Cytometry Assay(1,2)/Flow Cytometry Assay-1/╧╕░√╡≥═÷-1/╡≥═÷ 2/═╝╞1⁄4/4h 3/═╝2.tiff]

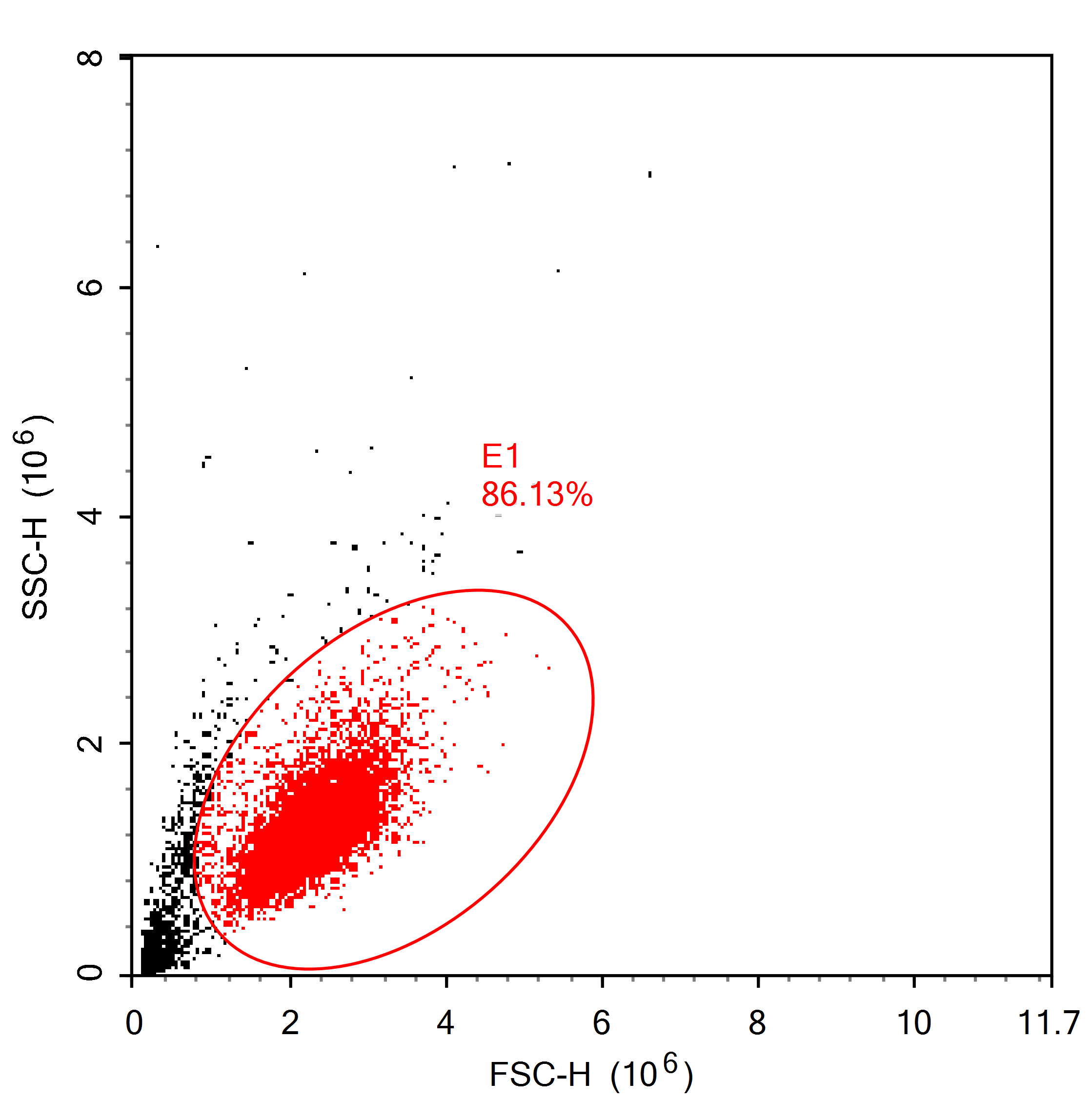

Supplement: Supplementary file 1 [file DataSheet3.zip › Flow Cytometry Assay(1,2)/Flow Cytometry Assay-1/╧╕░√╡≥═÷-1/╡≥═÷ 2/═╝╞1⁄4/8h 1/═╝1.tiff]

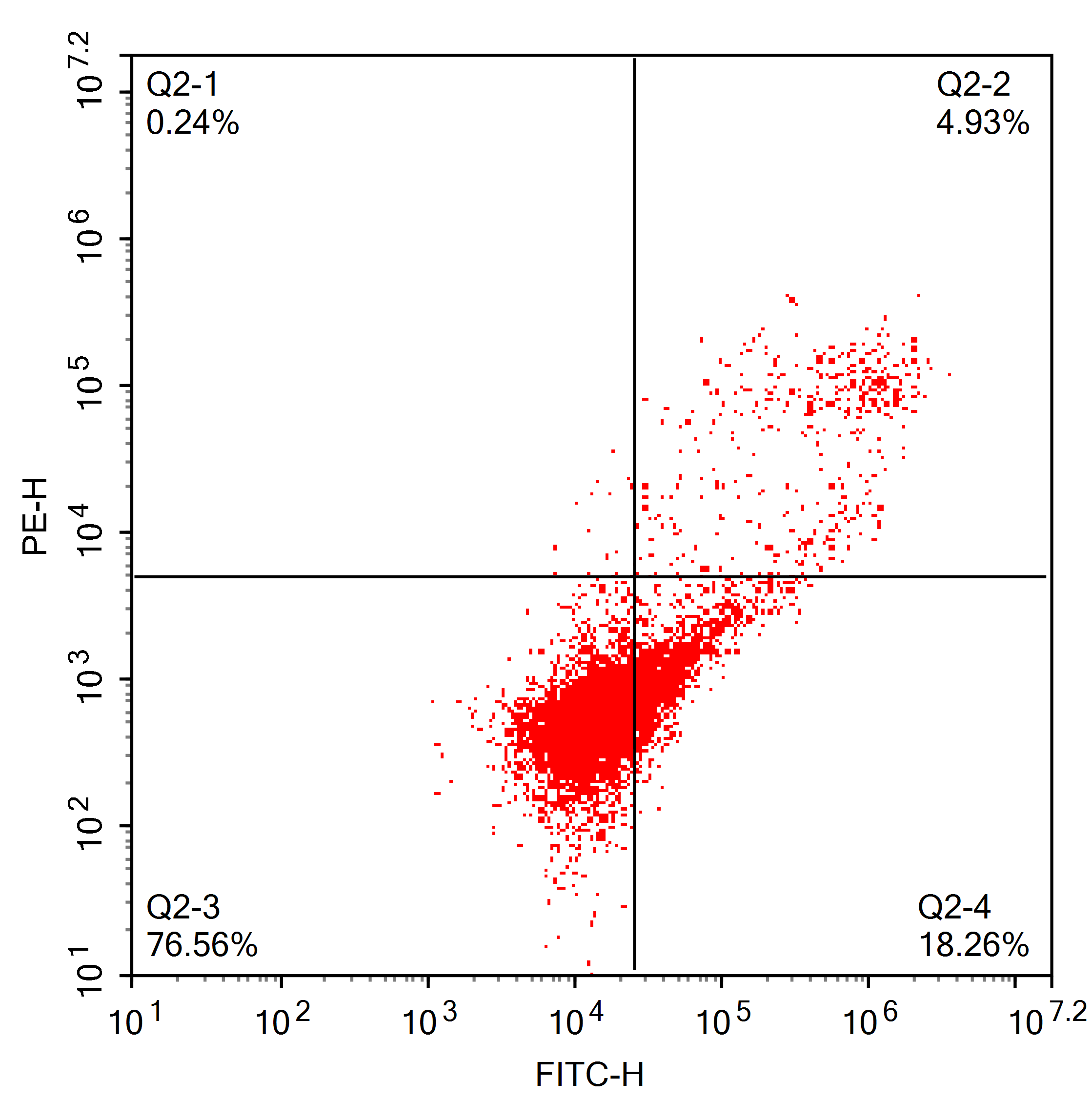

Supplement: Supplementary file 1 [file DataSheet3.zip › Flow Cytometry Assay(1,2)/Flow Cytometry Assay-1/╧╕░√╡≥═÷-1/╡≥═÷ 2/═╝╞1⁄4/8h 1/═╝2.tiff]

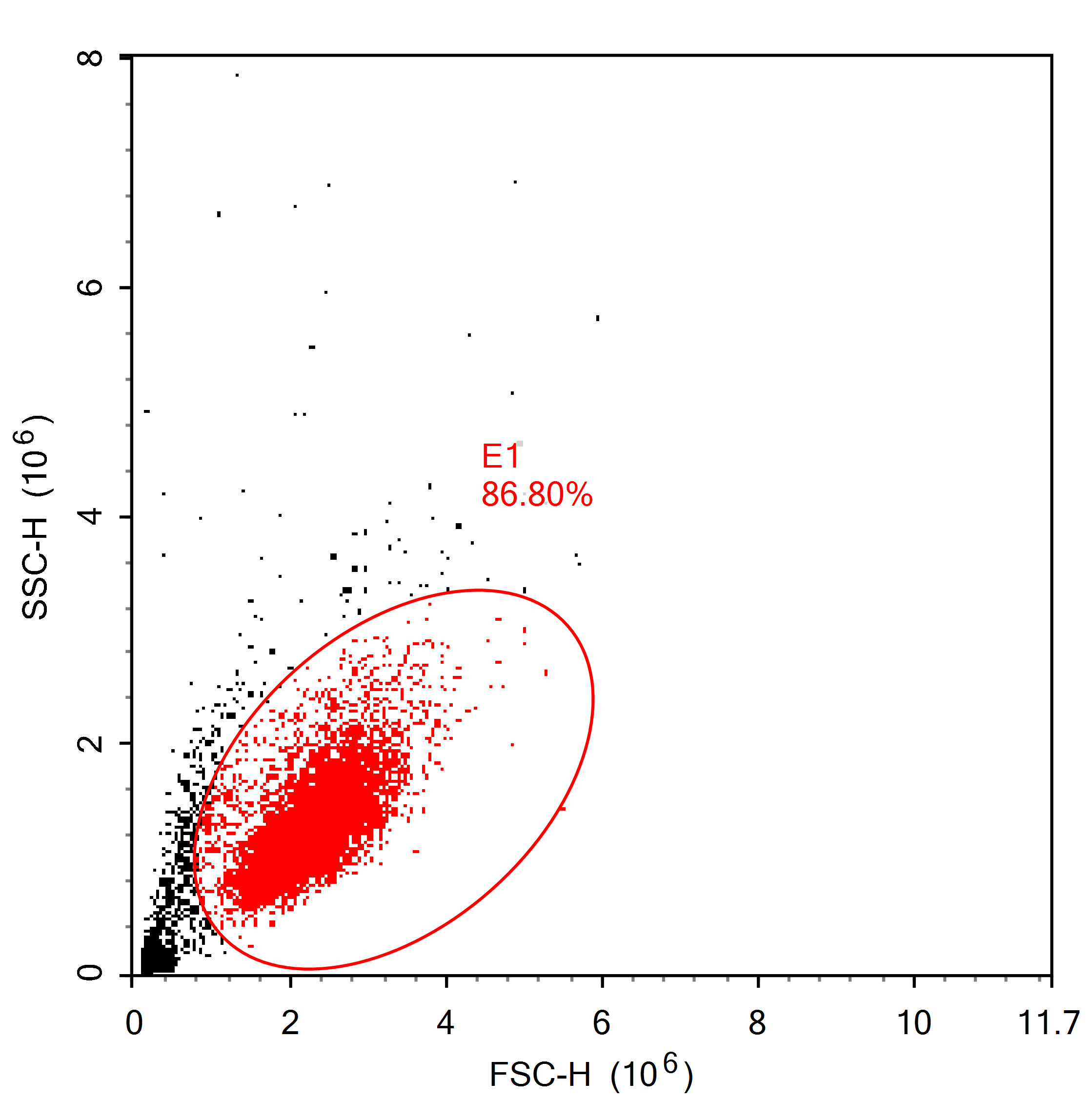

Supplement: Supplementary file 1 [file DataSheet3.zip › Flow Cytometry Assay(1,2)/Flow Cytometry Assay-1/╧╕░√╡≥═÷-1/╡≥═÷ 2/═╝╞1⁄4/8h 2/═╝1.tiff]

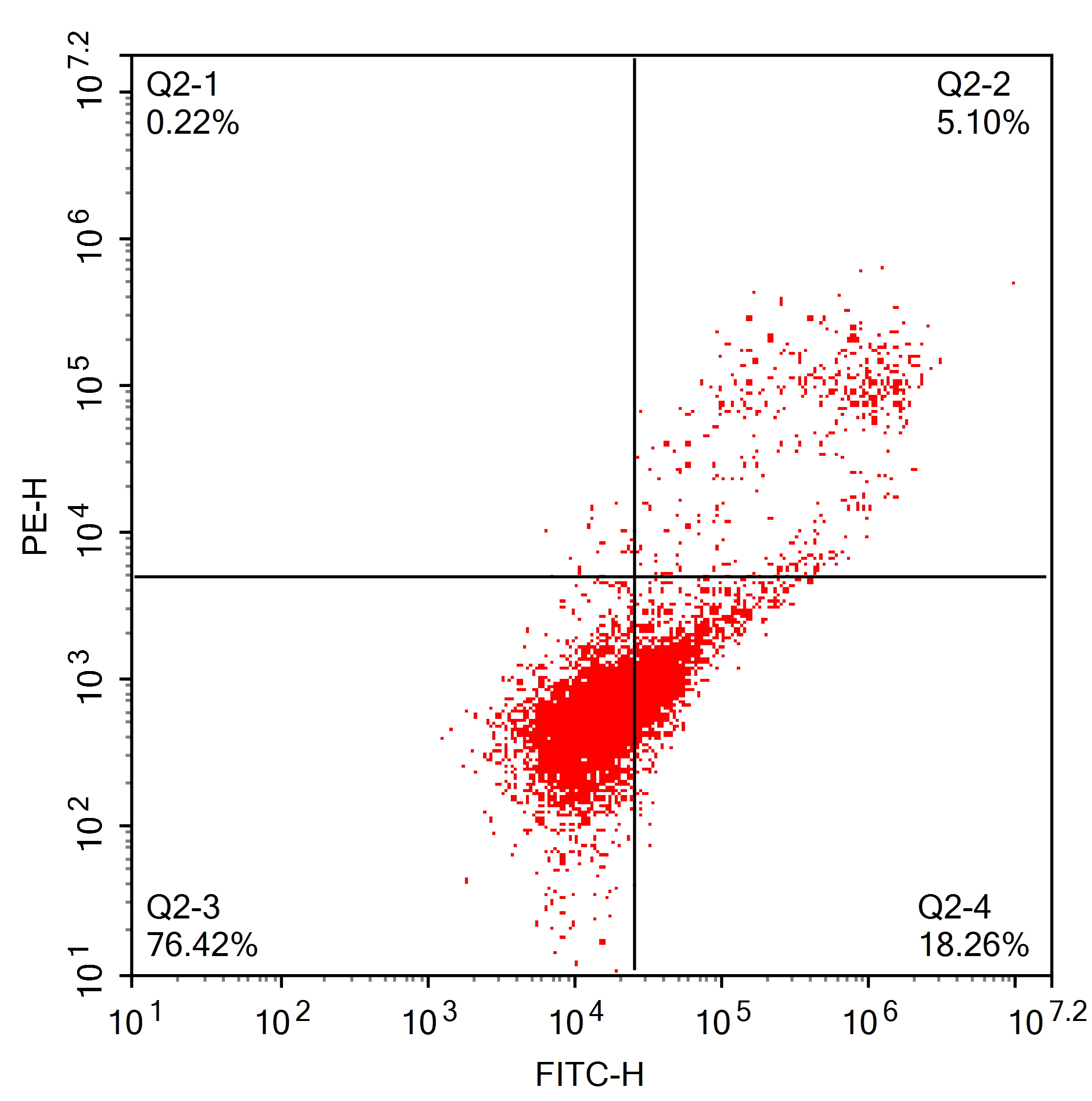

Supplement: Supplementary file 1 [file DataSheet3.zip › Flow Cytometry Assay(1,2)/Flow Cytometry Assay-1/╧╕░√╡≥═÷-1/╡≥═÷ 2/═╝╞1⁄4/8h 2/═╝2.tiff]

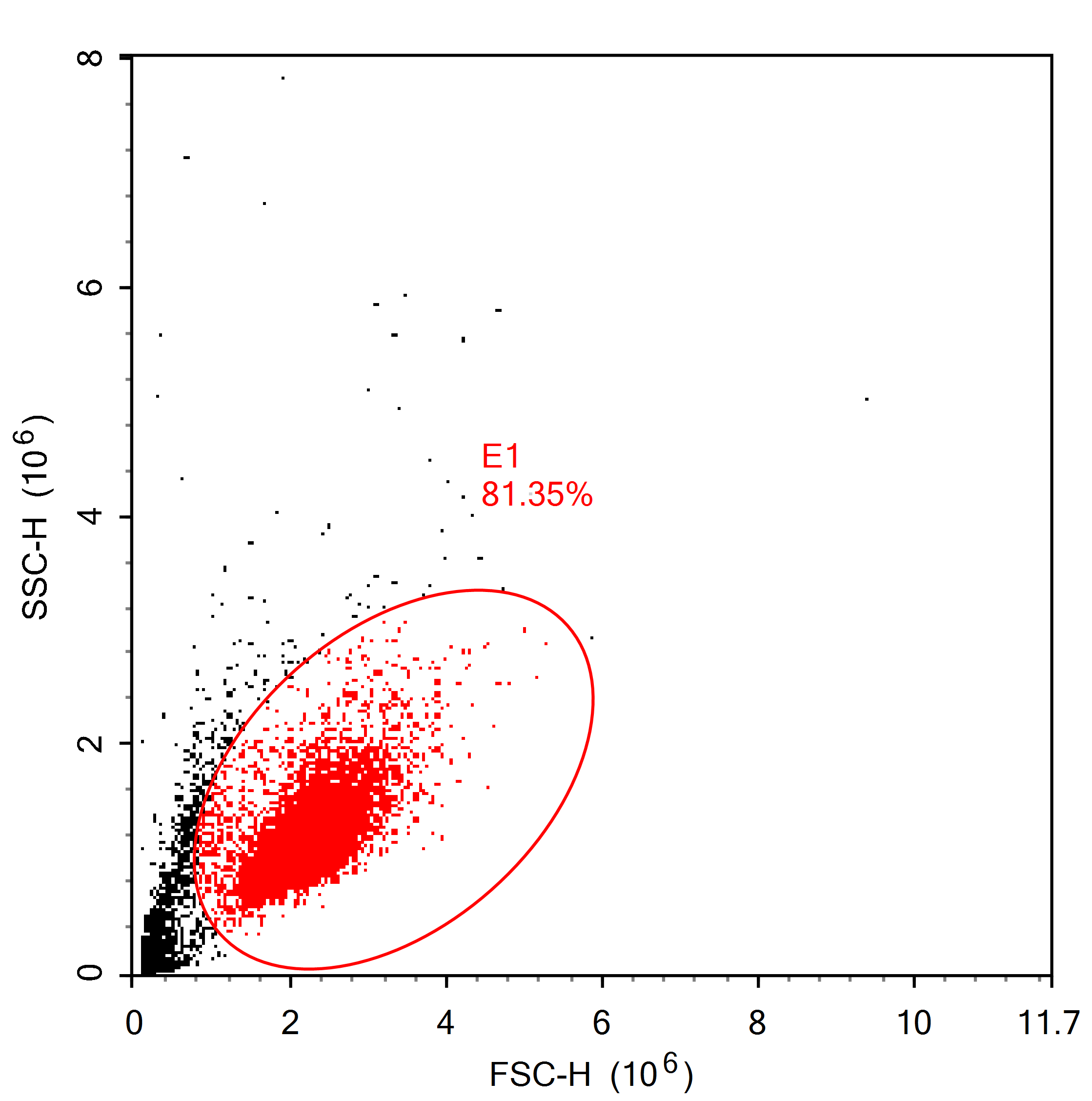

Supplement: Supplementary file 1 [file DataSheet3.zip › Flow Cytometry Assay(1,2)/Flow Cytometry Assay-1/╧╕░√╡≥═÷-1/╡≥═÷ 2/═╝╞1⁄4/8h 3/═╝1.tiff]

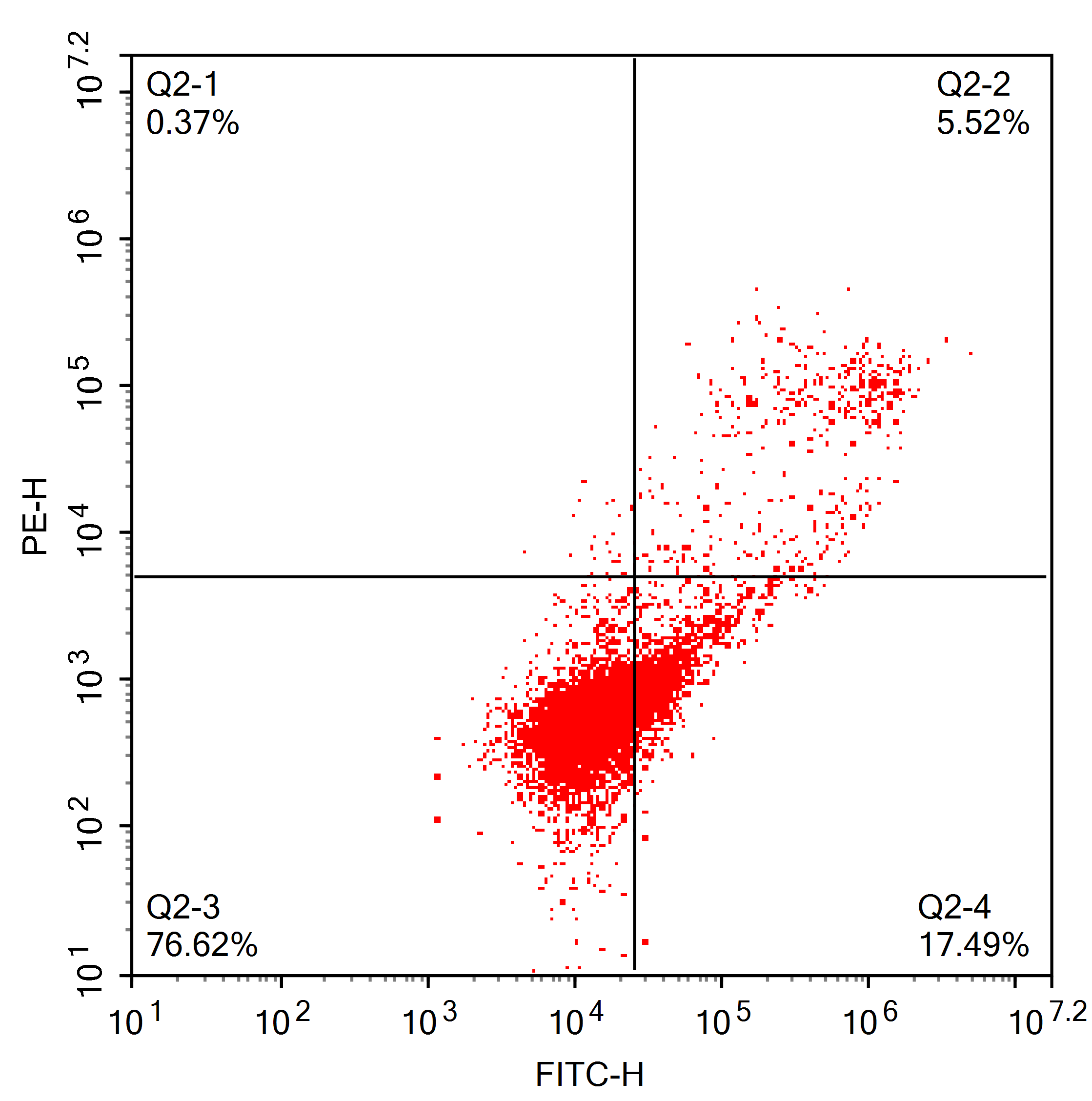

Supplement: Supplementary file 1 [file DataSheet3.zip › Flow Cytometry Assay(1,2)/Flow Cytometry Assay-1/╧╕░√╡≥═÷-1/╡≥═÷ 2/═╝╞1⁄4/8h 3/═╝2.tiff]

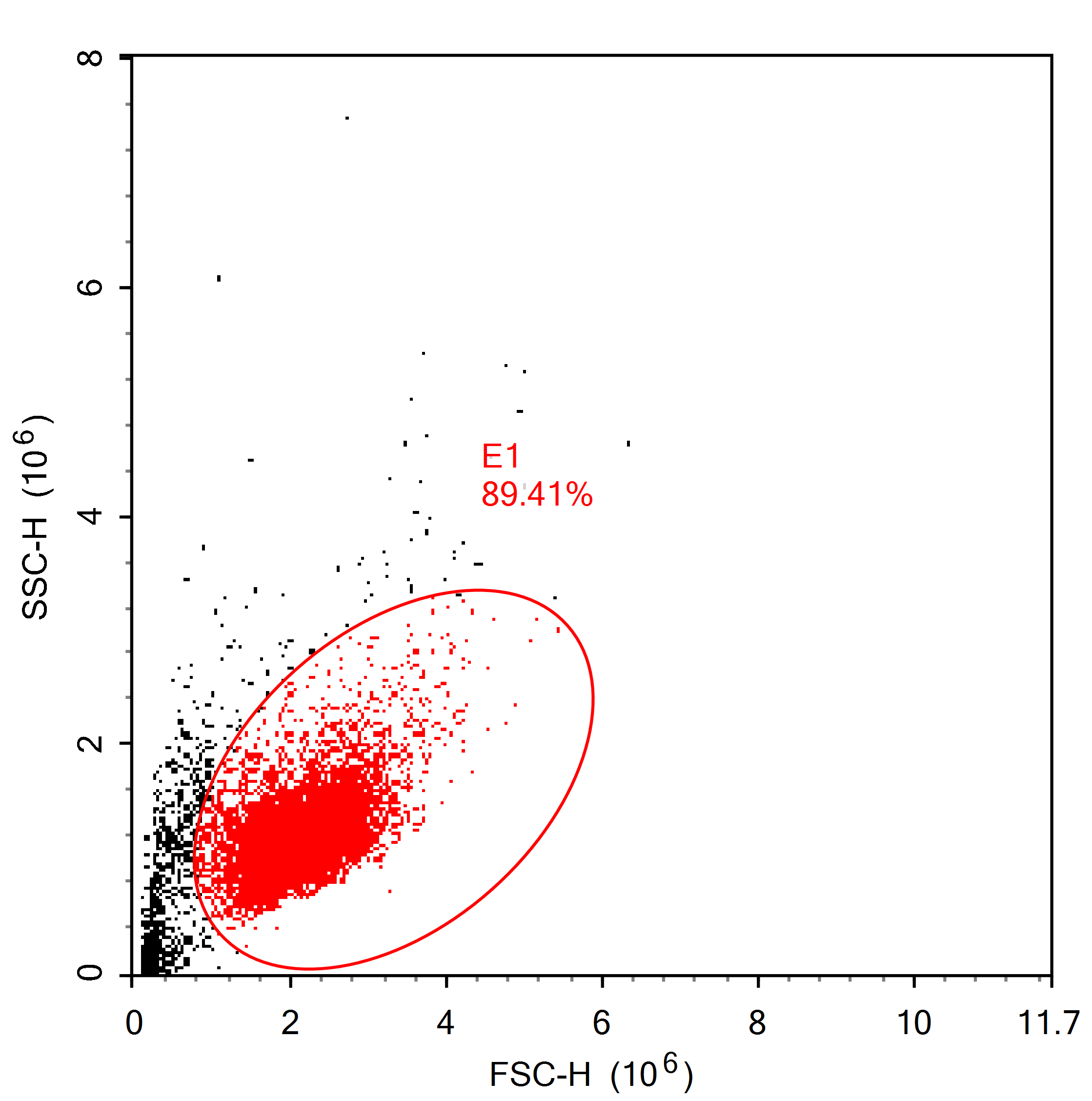

Supplement: Supplementary file 1 [file DataSheet3.zip › Flow Cytometry Assay(1,2)/Flow Cytometry Assay-1/╧╕░√╡≥═÷-1/╡≥═÷ 2/═╝╞1⁄4/blank/═╝1.tiff]

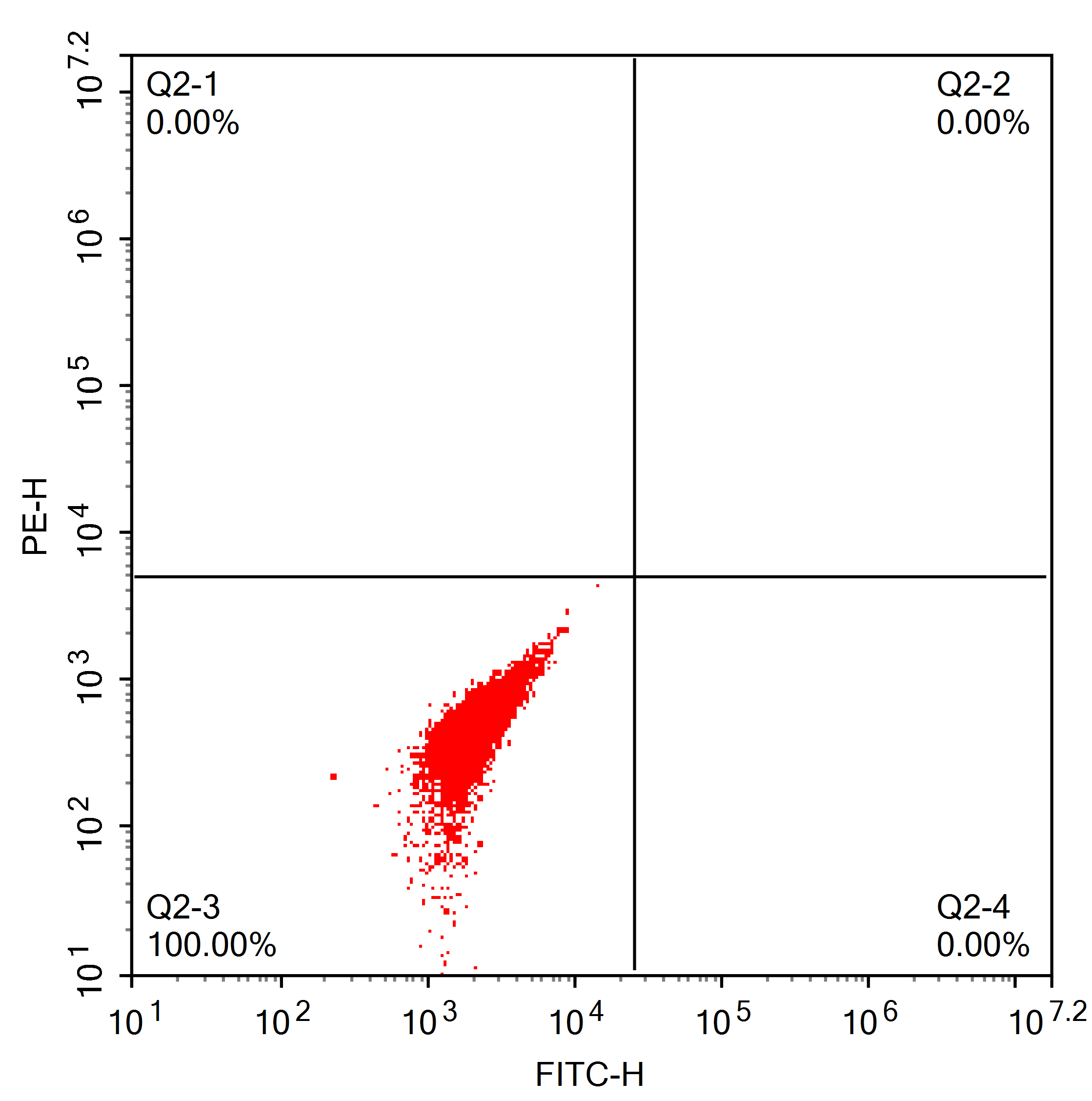

Supplement: Supplementary file 1 [file DataSheet3.zip › Flow Cytometry Assay(1,2)/Flow Cytometry Assay-1/╧╕░√╡≥═÷-1/╡≥═÷ 2/═╝╞1⁄4/blank/═╝2.tiff]

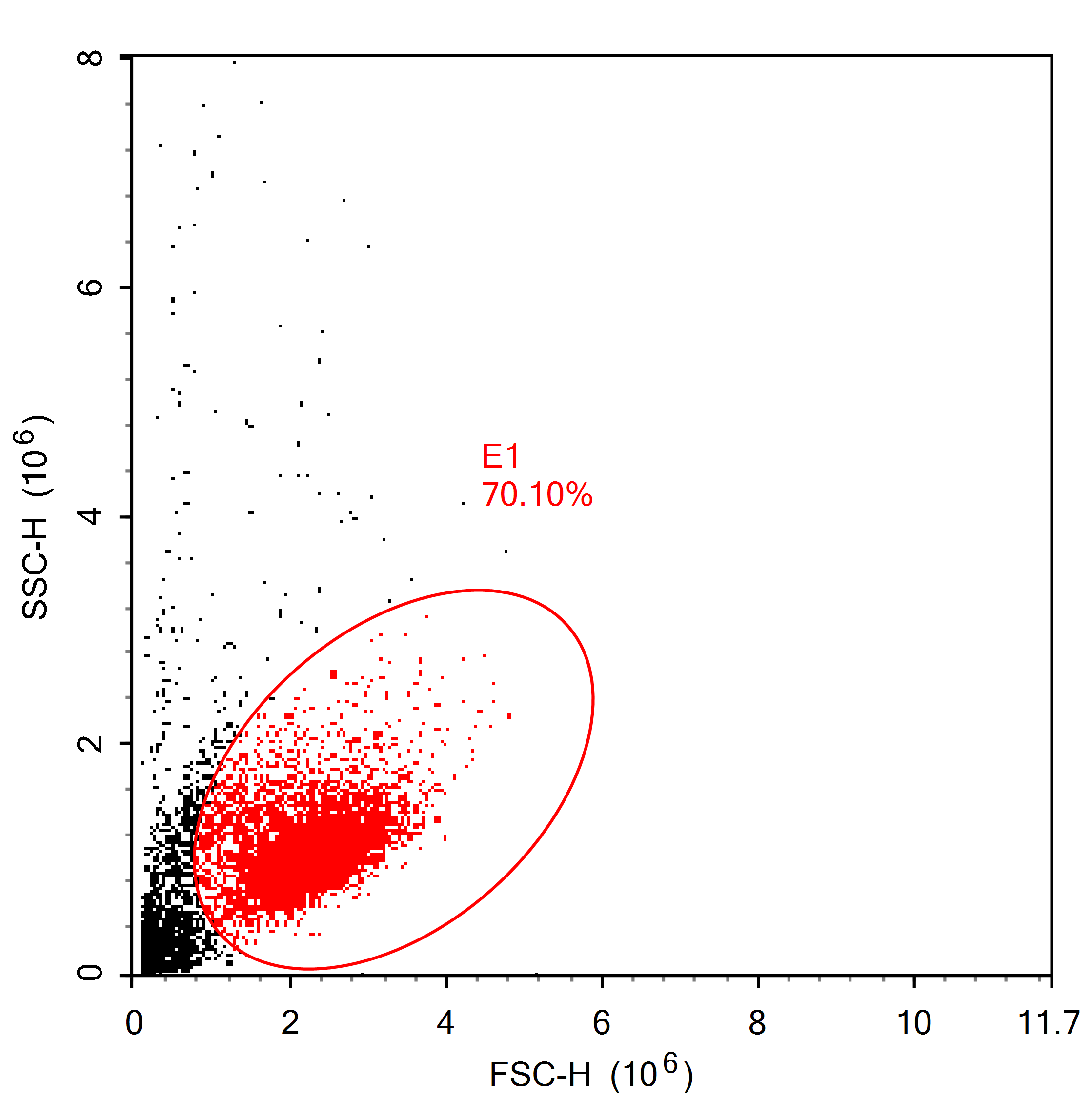

Supplement: Supplementary file 1 [file DataSheet3.zip › Flow Cytometry Assay(1,2)/Flow Cytometry Assay-1/╧╕░√╡≥═÷-1/╡≥═÷ 2/═╝╞1⁄4/Ctrl 1/═╝1.tiff]

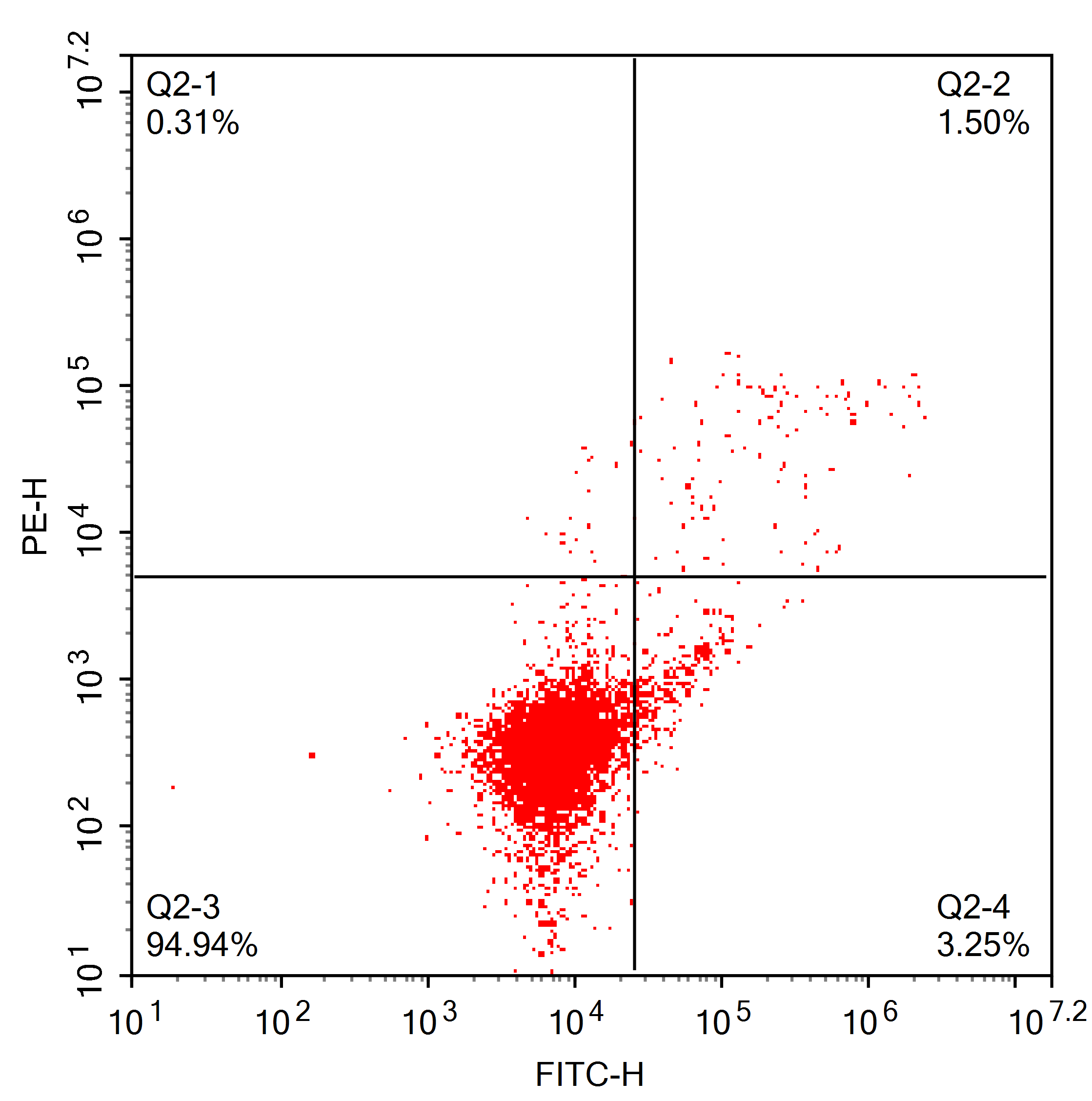

Supplement: Supplementary file 1 [file DataSheet3.zip › Flow Cytometry Assay(1,2)/Flow Cytometry Assay-1/╧╕░√╡≥═÷-1/╡≥═÷ 2/═╝╞1⁄4/Ctrl 1/═╝2.tiff]

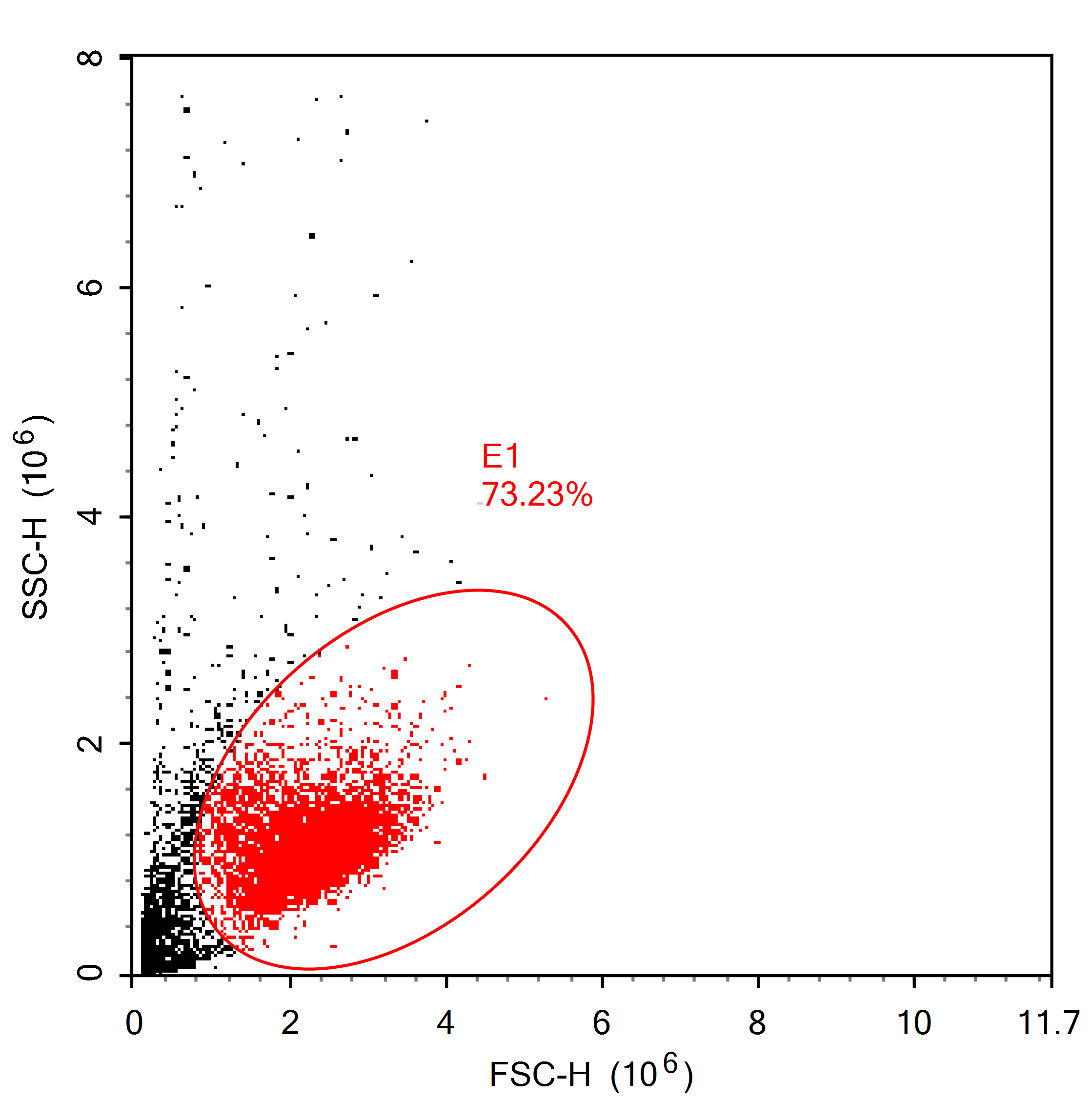

Supplement: Supplementary file 1 [file DataSheet3.zip › Flow Cytometry Assay(1,2)/Flow Cytometry Assay-1/╧╕░√╡≥═÷-1/╡≥═÷ 2/═╝╞1⁄4/Ctrl 2/═╝1.tiff]

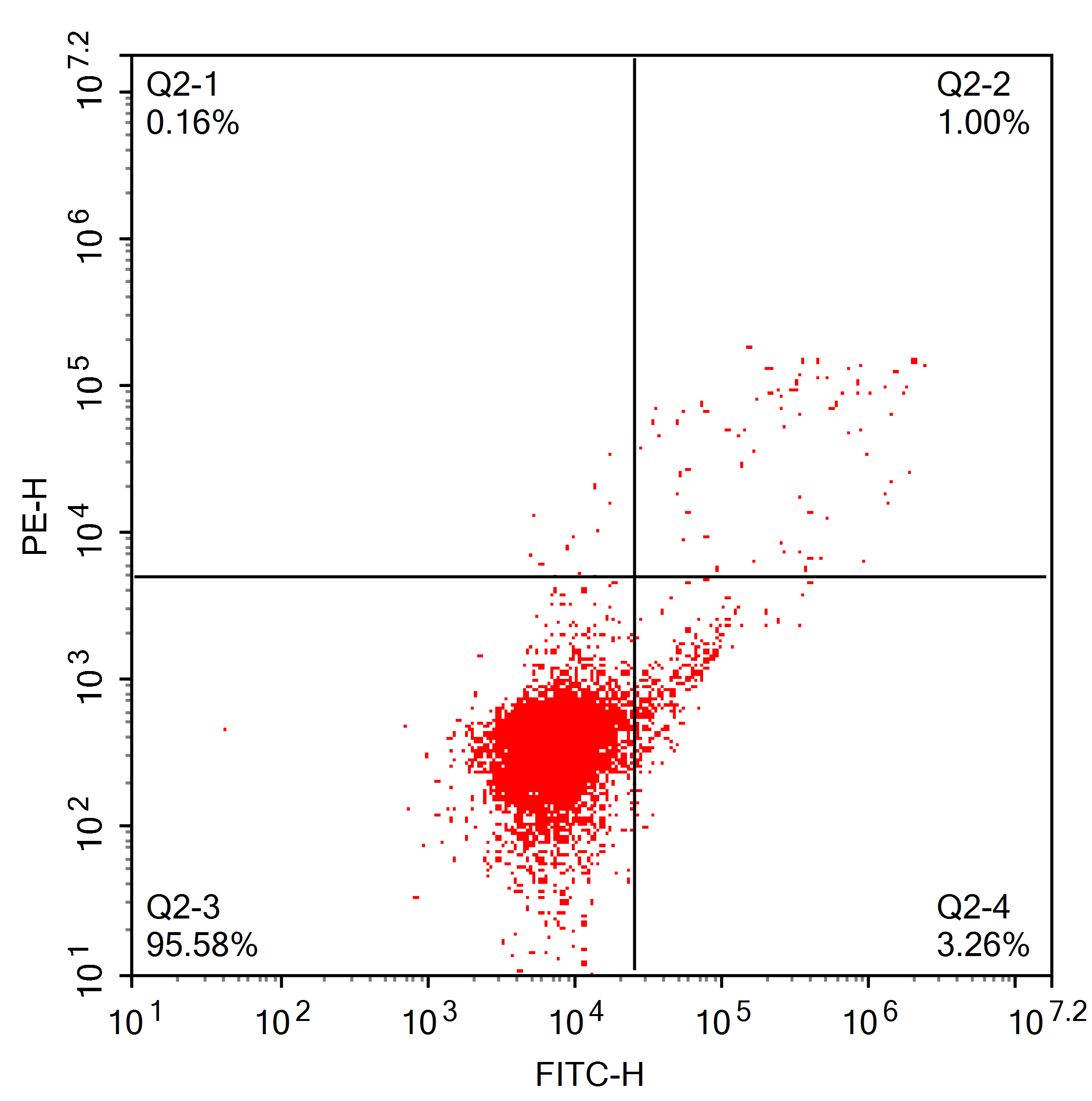

Supplement: Supplementary file 1 [file DataSheet3.zip › Flow Cytometry Assay(1,2)/Flow Cytometry Assay-1/╧╕░√╡≥═÷-1/╡≥═÷ 2/═╝╞1⁄4/Ctrl 2/═╝2.tiff]

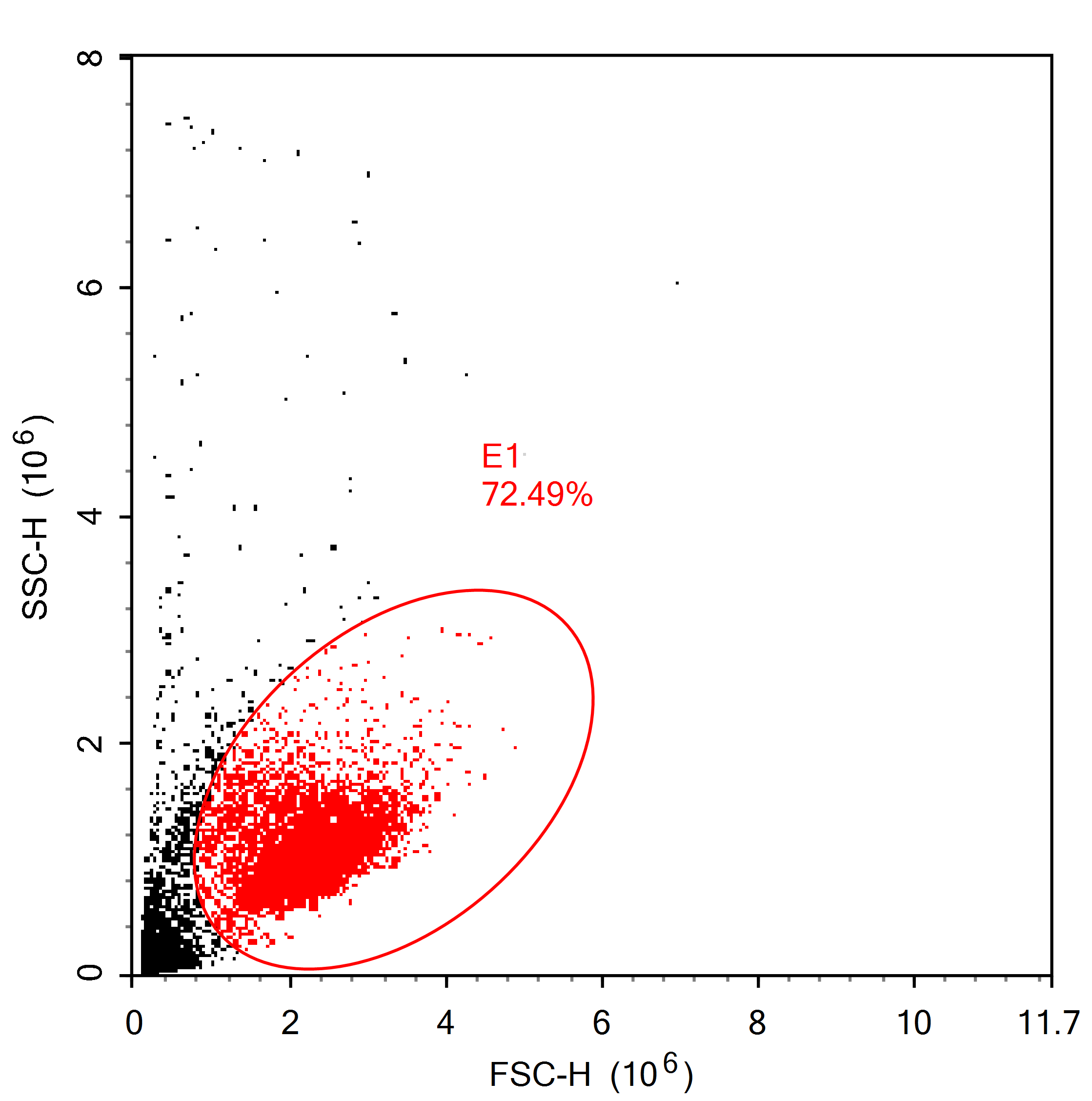

Supplement: Supplementary file 1 [file DataSheet3.zip › Flow Cytometry Assay(1,2)/Flow Cytometry Assay-1/╧╕░√╡≥═÷-1/╡≥═÷ 2/═╝╞1⁄4/Ctrl 3/═╝1.tiff]

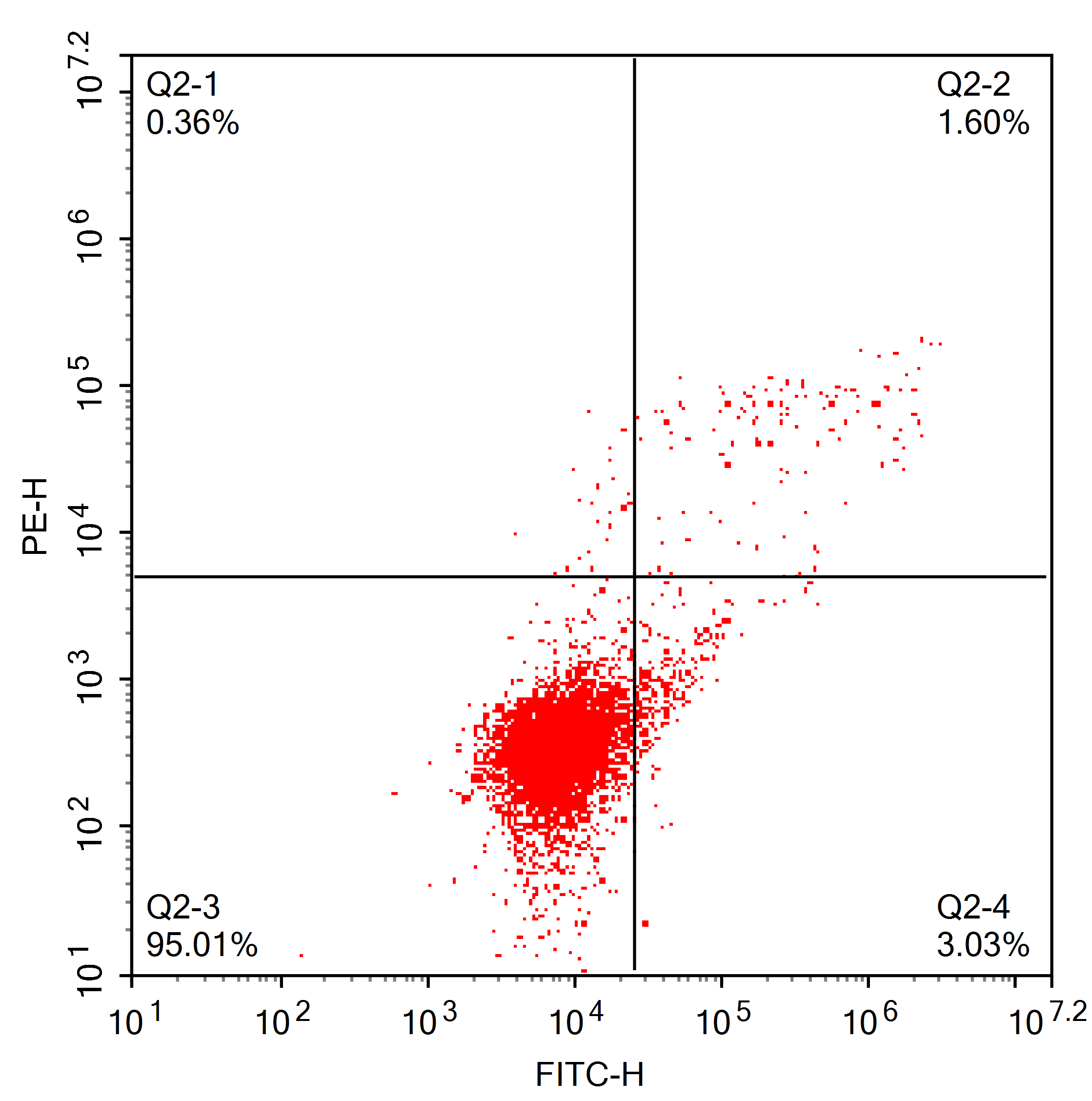

Supplement: Supplementary file 1 [file DataSheet3.zip › Flow Cytometry Assay(1,2)/Flow Cytometry Assay-1/╧╕░√╡≥═÷-1/╡≥═÷ 2/═╝╞1⁄4/Ctrl 3/═╝2.tiff]

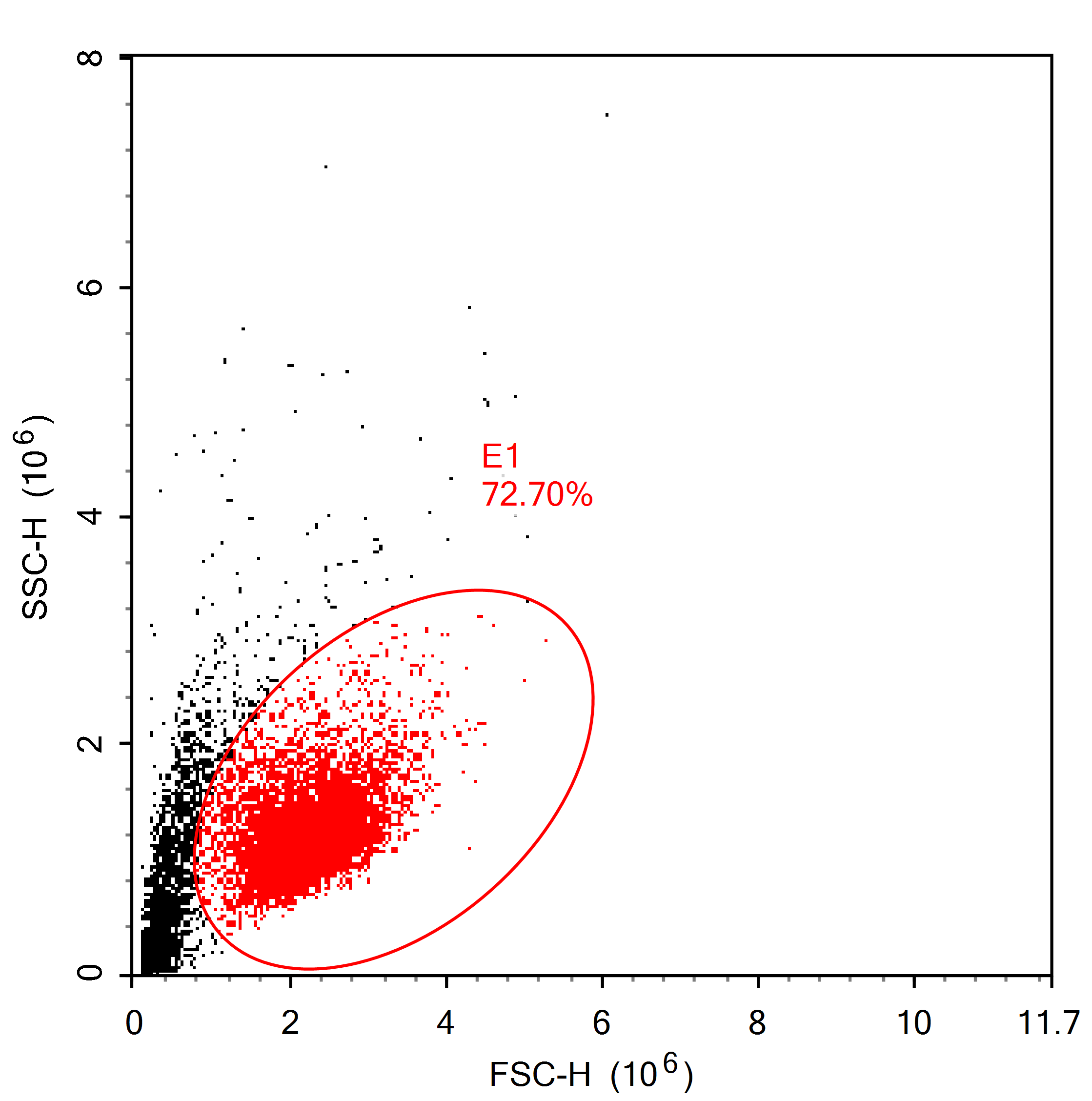

Supplement: Supplementary file 1 [file DataSheet3.zip › Flow Cytometry Assay(1,2)/Flow Cytometry Assay-1/╧╕░√╡≥═÷-1/╡≥═÷ 2/═╝╞1⁄4/FITC/═╝1.tiff]

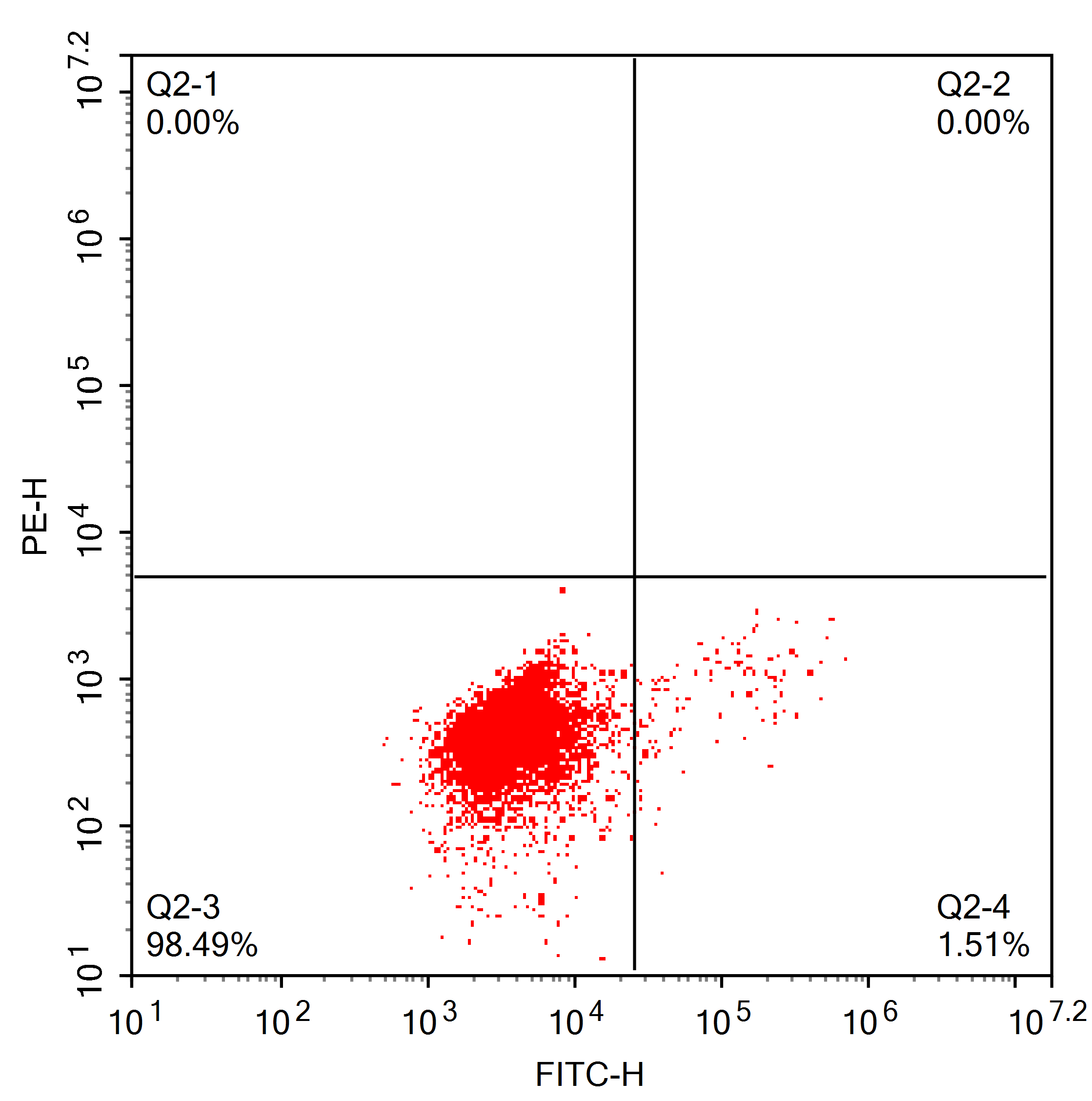

Supplement: Supplementary file 1 [file DataSheet3.zip › Flow Cytometry Assay(1,2)/Flow Cytometry Assay-1/╧╕░√╡≥═÷-1/╡≥═÷ 2/═╝╞1⁄4/FITC/═╝2.tiff]

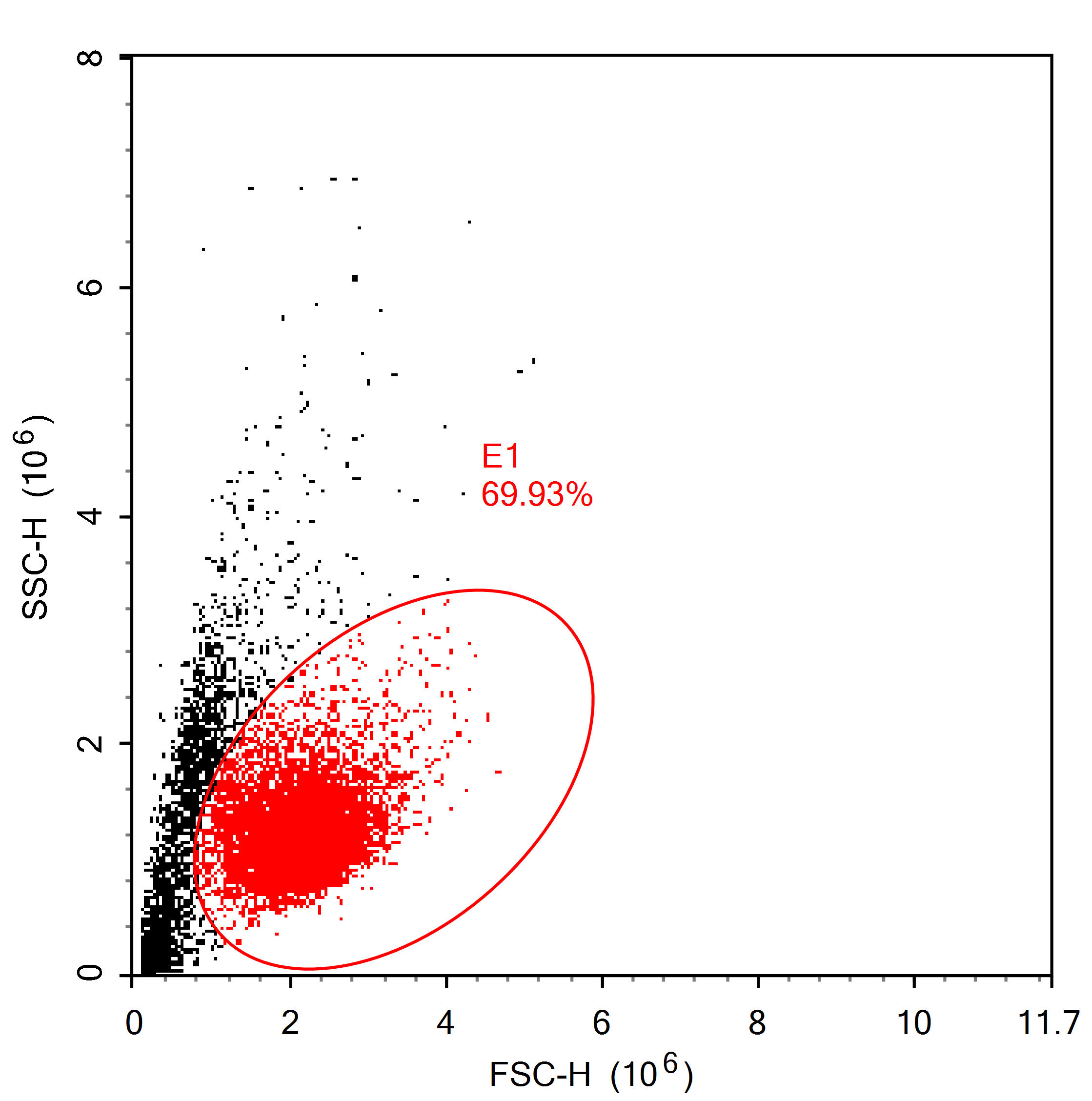

Supplement: Supplementary file 1 [file DataSheet3.zip › Flow Cytometry Assay(1,2)/Flow Cytometry Assay-1/╧╕░√╡≥═÷-1/╡≥═÷ 2/═╝╞1⁄4/PI/═╝1.tiff]

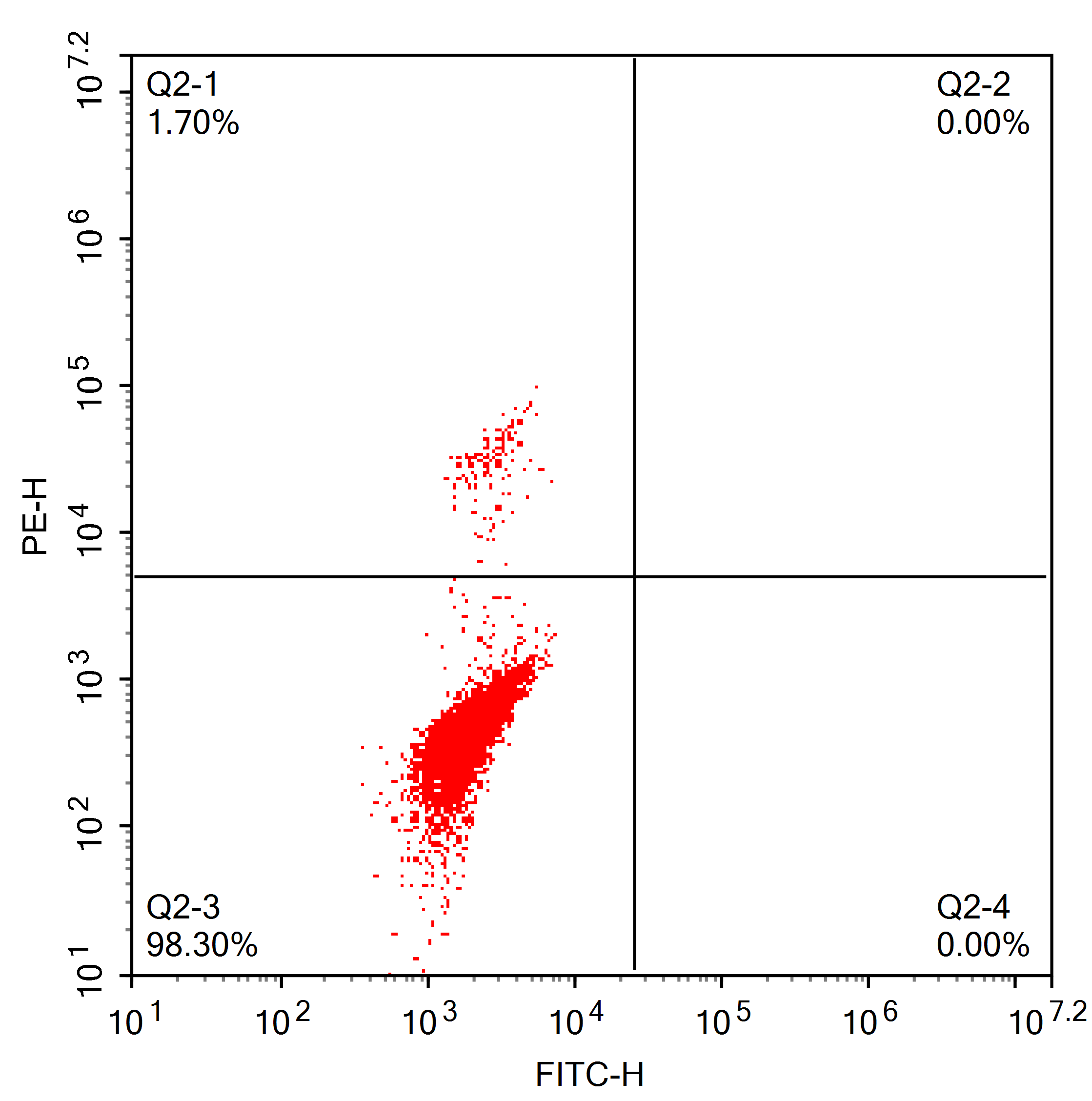

Supplement: Supplementary file 1 [file DataSheet3.zip › Flow Cytometry Assay(1,2)/Flow Cytometry Assay-1/╧╕░√╡≥═÷-1/╡≥═÷ 2/═╝╞1⁄4/PI/═╝2.tiff]

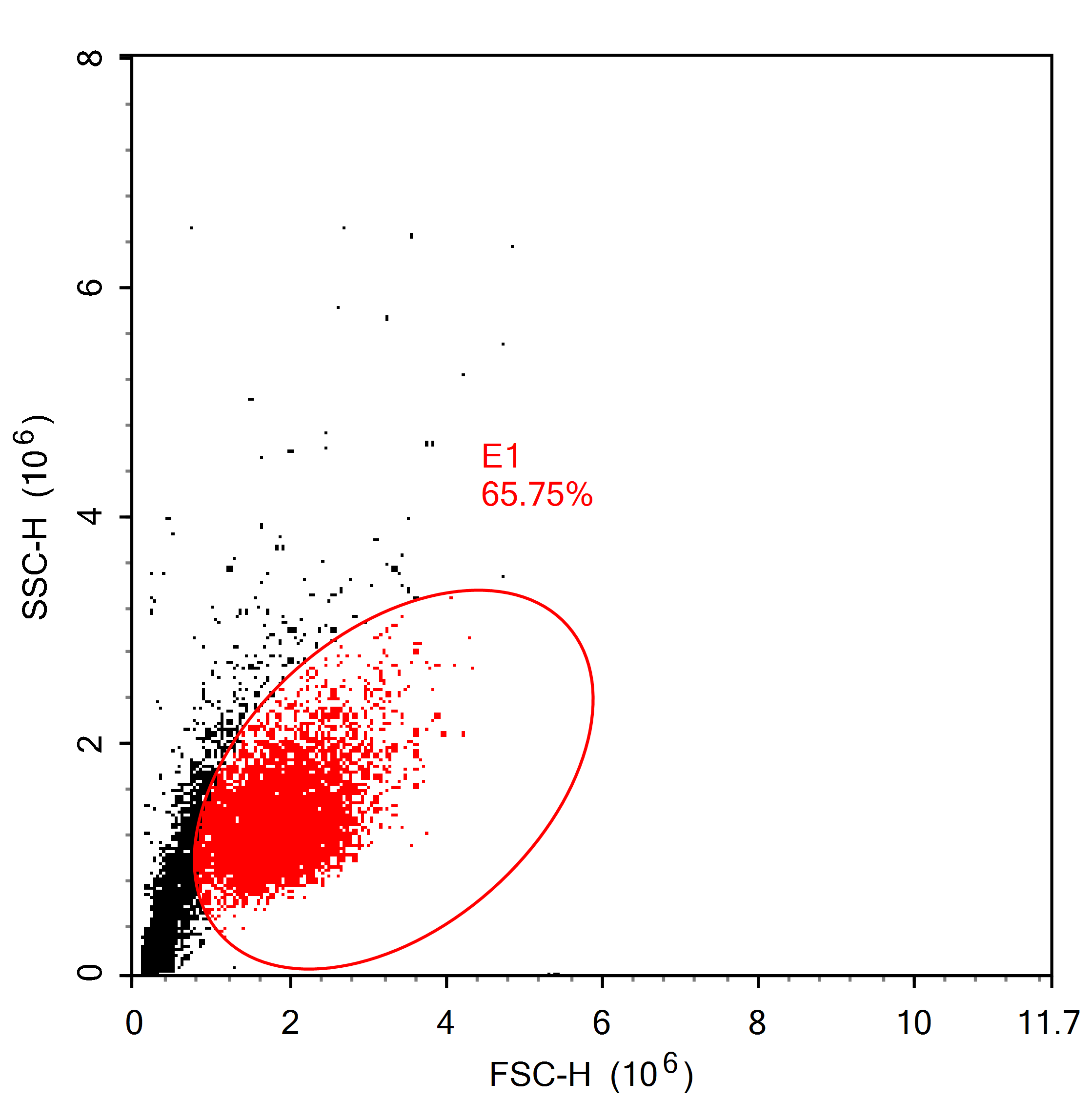

Supplement: Supplementary file 1 [file DataSheet3.zip › Flow Cytometry Assay(1,2)/Flow Cytometry Assay-1/╧╕░√╡≥═÷-1/╡≥═÷ 3/═╝╞1⁄4/12h 1/═╝1.tiff]

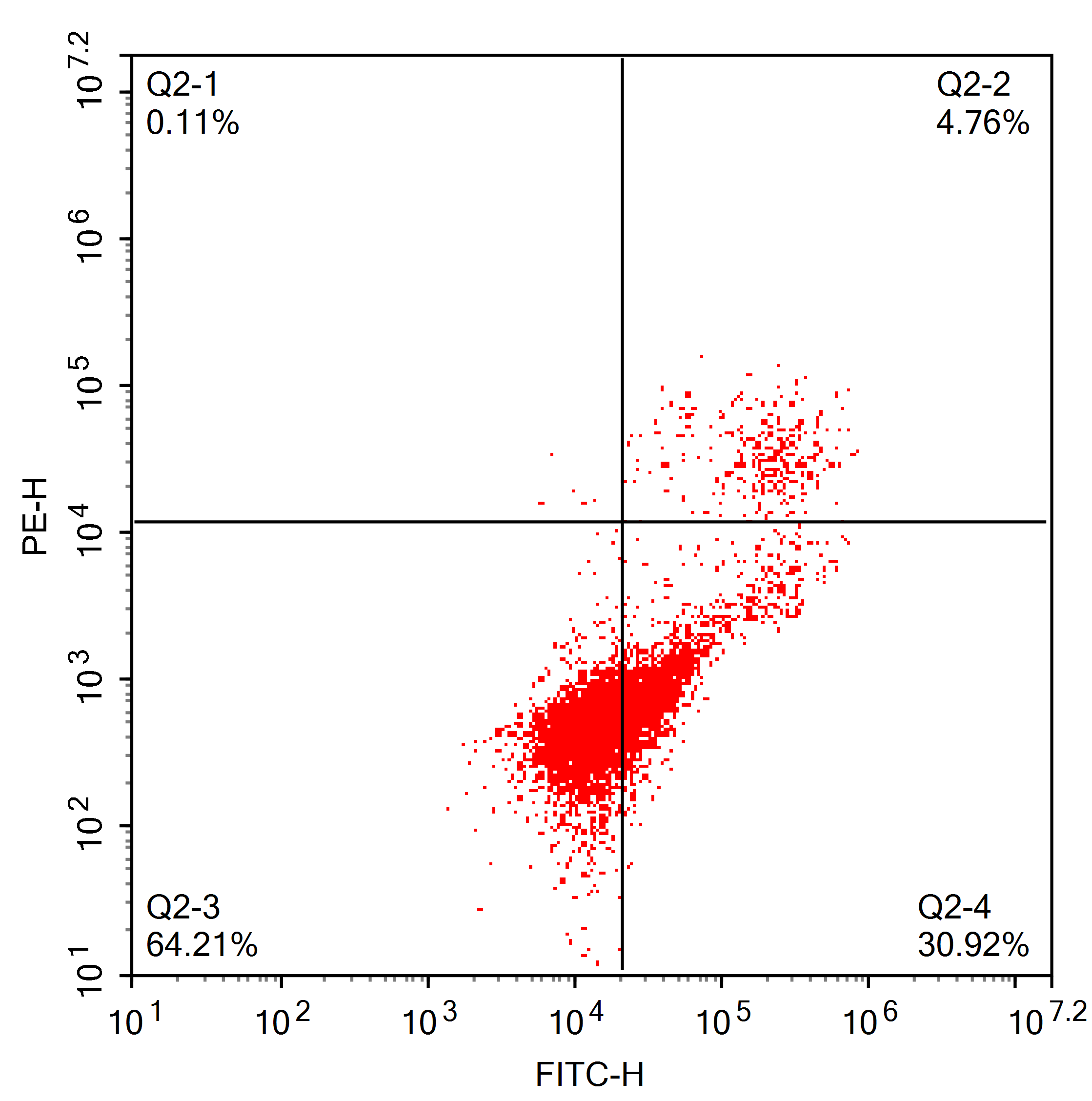

Supplement: Supplementary file 1 [file DataSheet3.zip › Flow Cytometry Assay(1,2)/Flow Cytometry Assay-1/╧╕░√╡≥═÷-1/╡≥═÷ 3/═╝╞1⁄4/12h 1/═╝2.tiff]

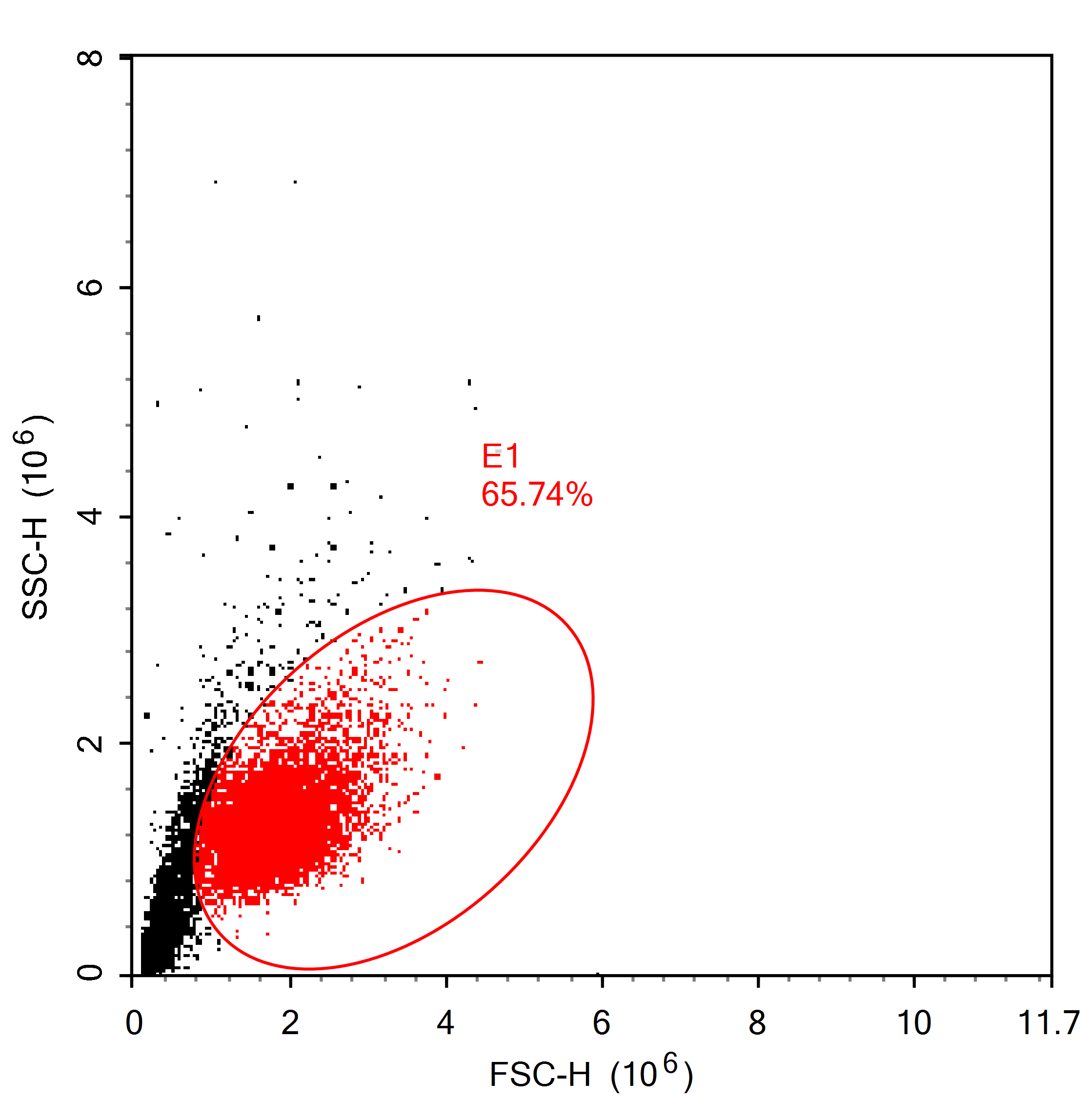

Supplement: Supplementary file 1 [file DataSheet3.zip › Flow Cytometry Assay(1,2)/Flow Cytometry Assay-1/╧╕░√╡≥═÷-1/╡≥═÷ 3/═╝╞1⁄4/12h 2/═╝1.tiff]

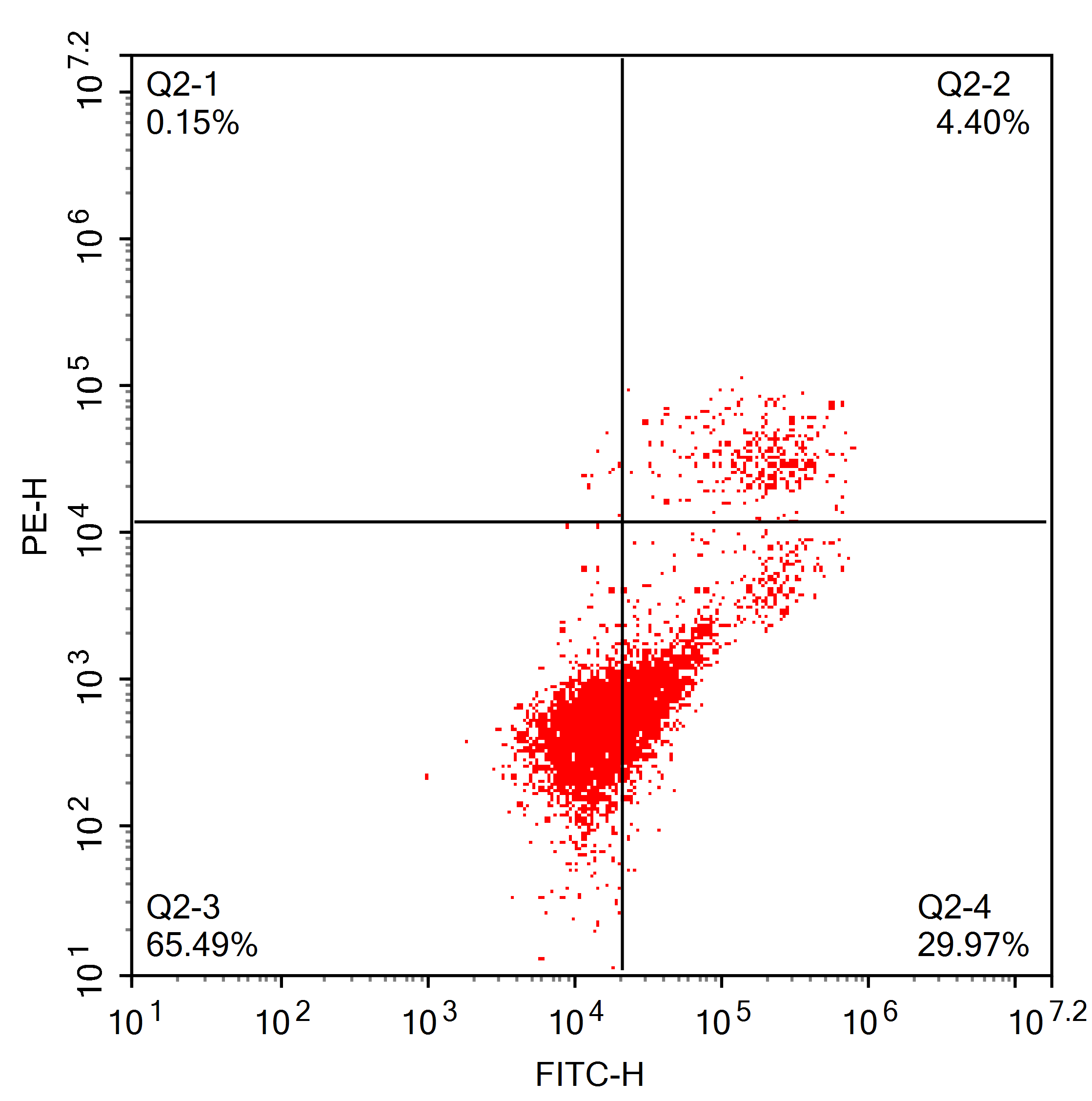

Supplement: Supplementary file 1 [file DataSheet3.zip › Flow Cytometry Assay(1,2)/Flow Cytometry Assay-1/╧╕░√╡≥═÷-1/╡≥═÷ 3/═╝╞1⁄4/12h 2/═╝2.tiff]

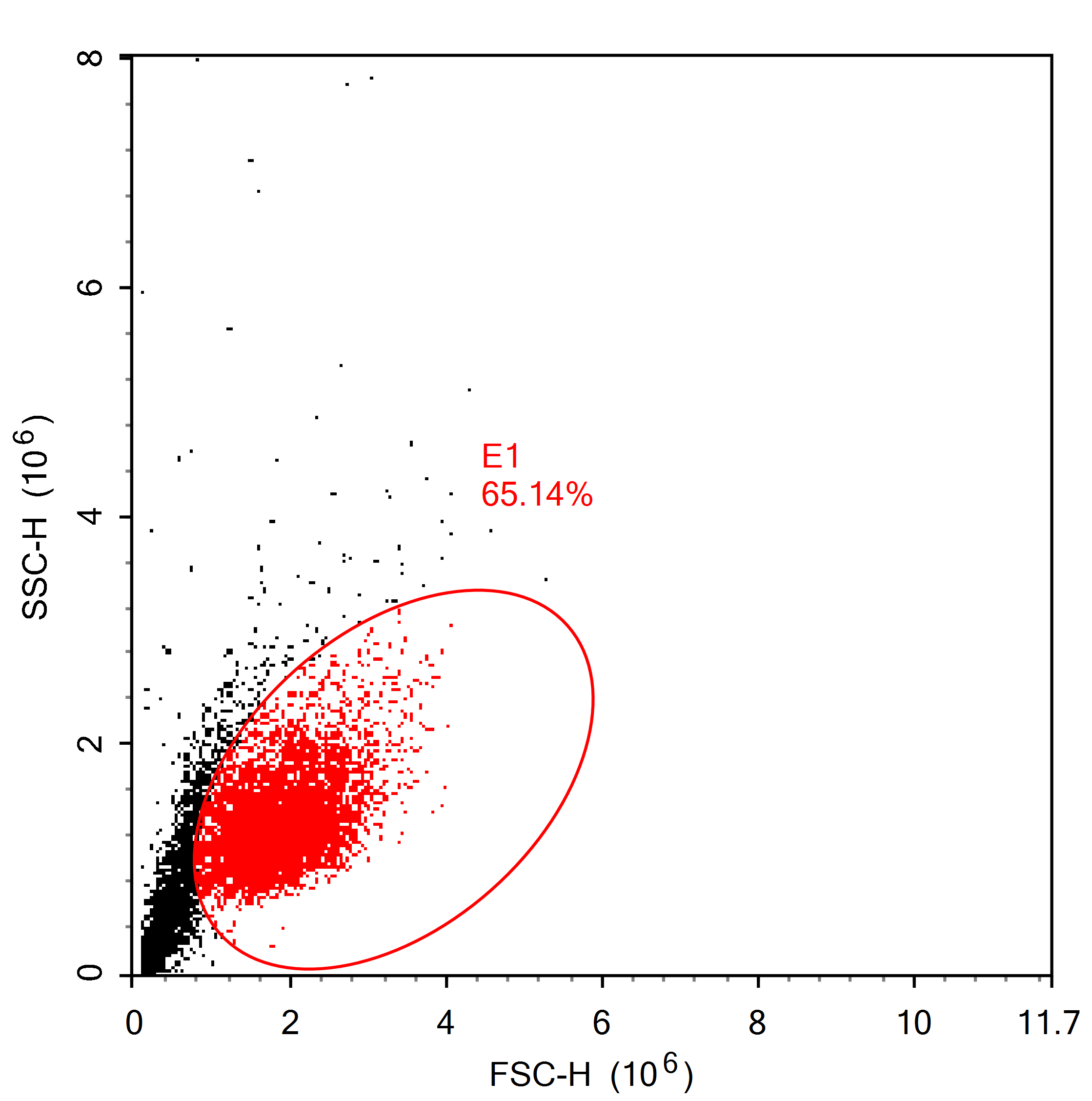

Supplement: Supplementary file 1 [file DataSheet3.zip › Flow Cytometry Assay(1,2)/Flow Cytometry Assay-1/╧╕░√╡≥═÷-1/╡≥═÷ 3/═╝╞1⁄4/12h 3/═╝1.tiff]

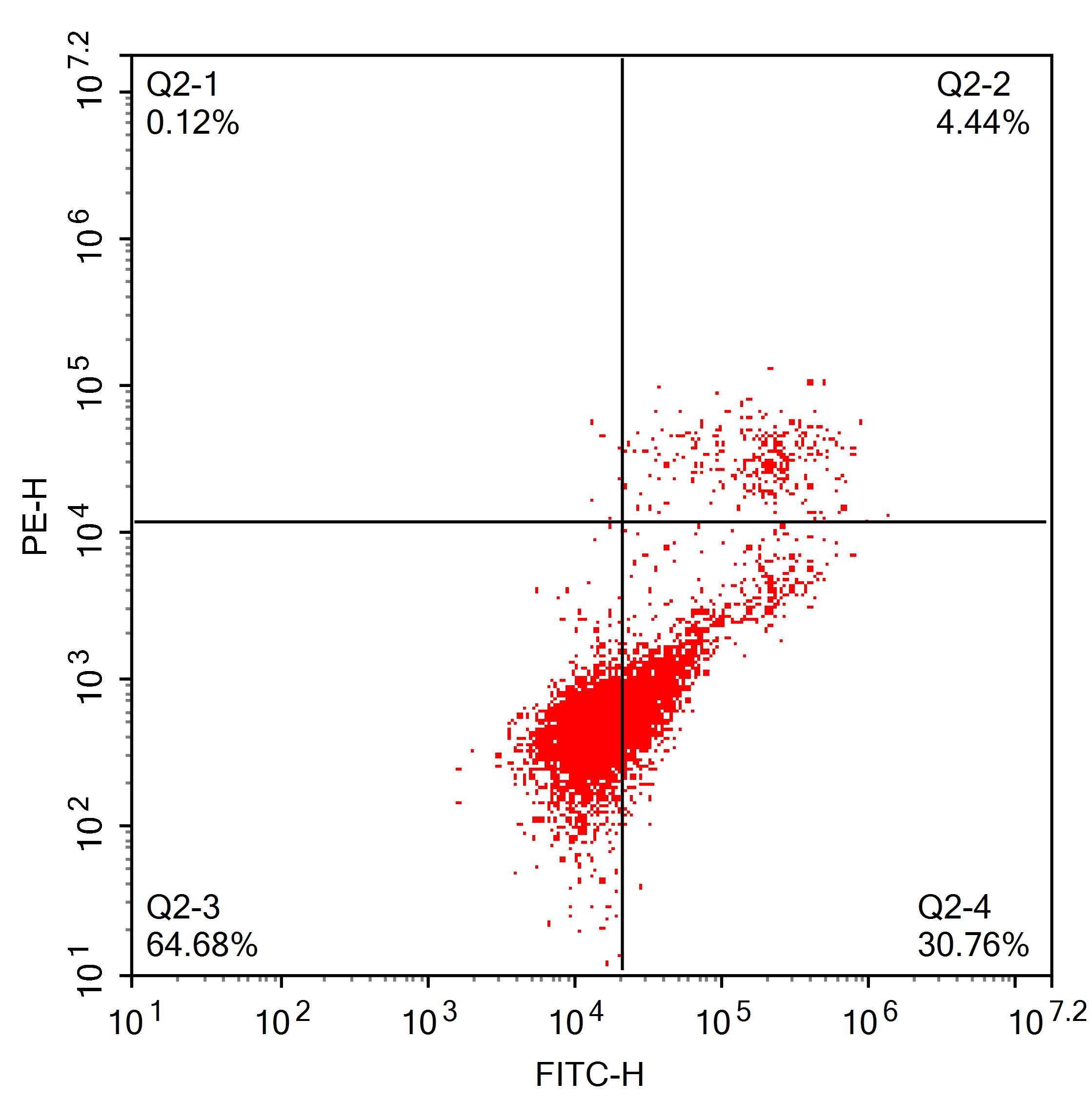

Supplement: Supplementary file 1 [file DataSheet3.zip › Flow Cytometry Assay(1,2)/Flow Cytometry Assay-1/╧╕░√╡≥═÷-1/╡≥═÷ 3/═╝╞1⁄4/12h 3/═╝2.tiff]

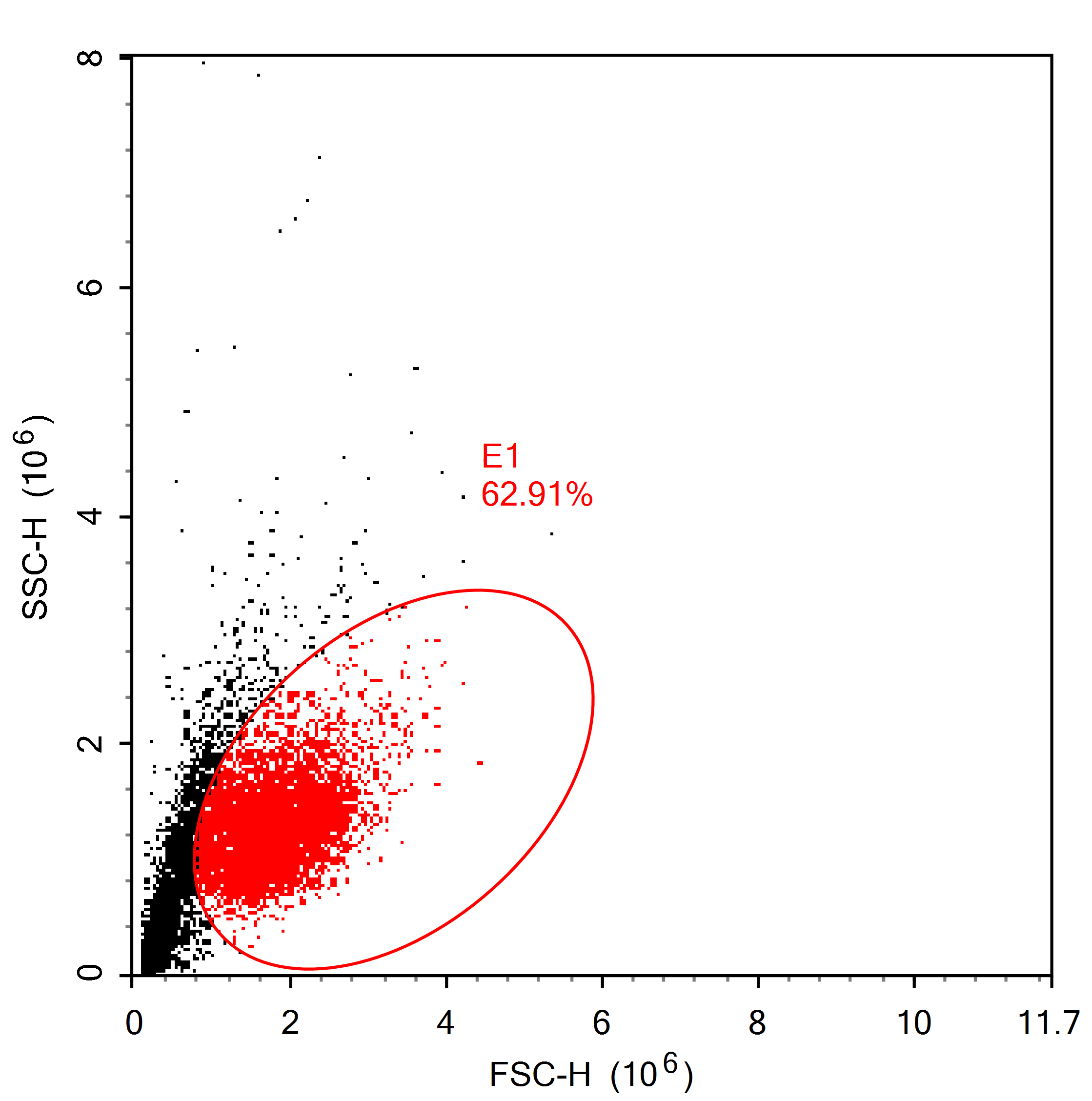

Supplement: Supplementary file 1 [file DataSheet3.zip › Flow Cytometry Assay(1,2)/Flow Cytometry Assay-1/╧╕░√╡≥═÷-1/╡≥═÷ 3/═╝╞1⁄4/4h 1/═╝1.tiff]

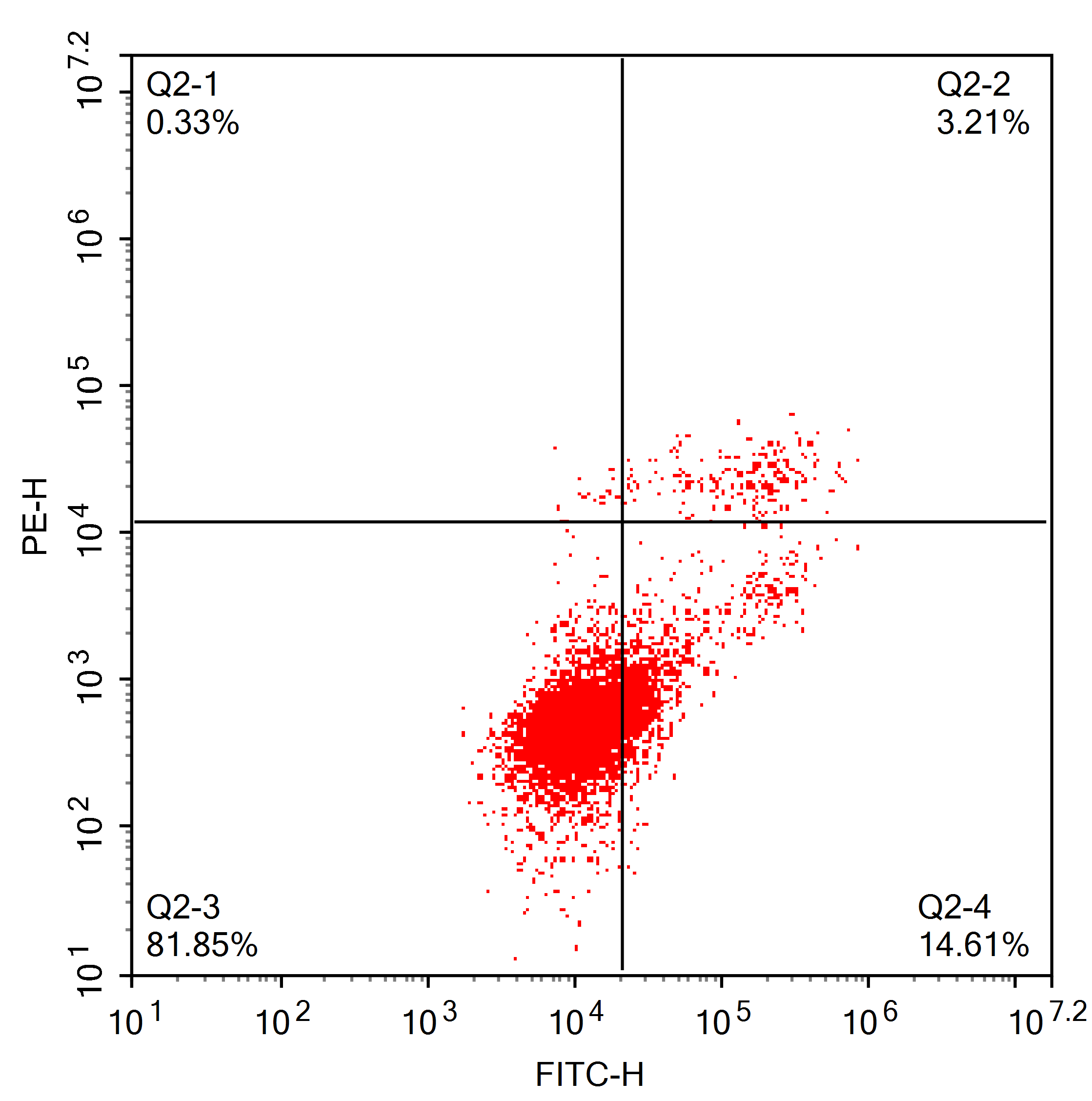

Supplement: Supplementary file 1 [file DataSheet3.zip › Flow Cytometry Assay(1,2)/Flow Cytometry Assay-1/╧╕░√╡≥═÷-1/╡≥═÷ 3/═╝╞1⁄4/4h 1/═╝2.tiff]

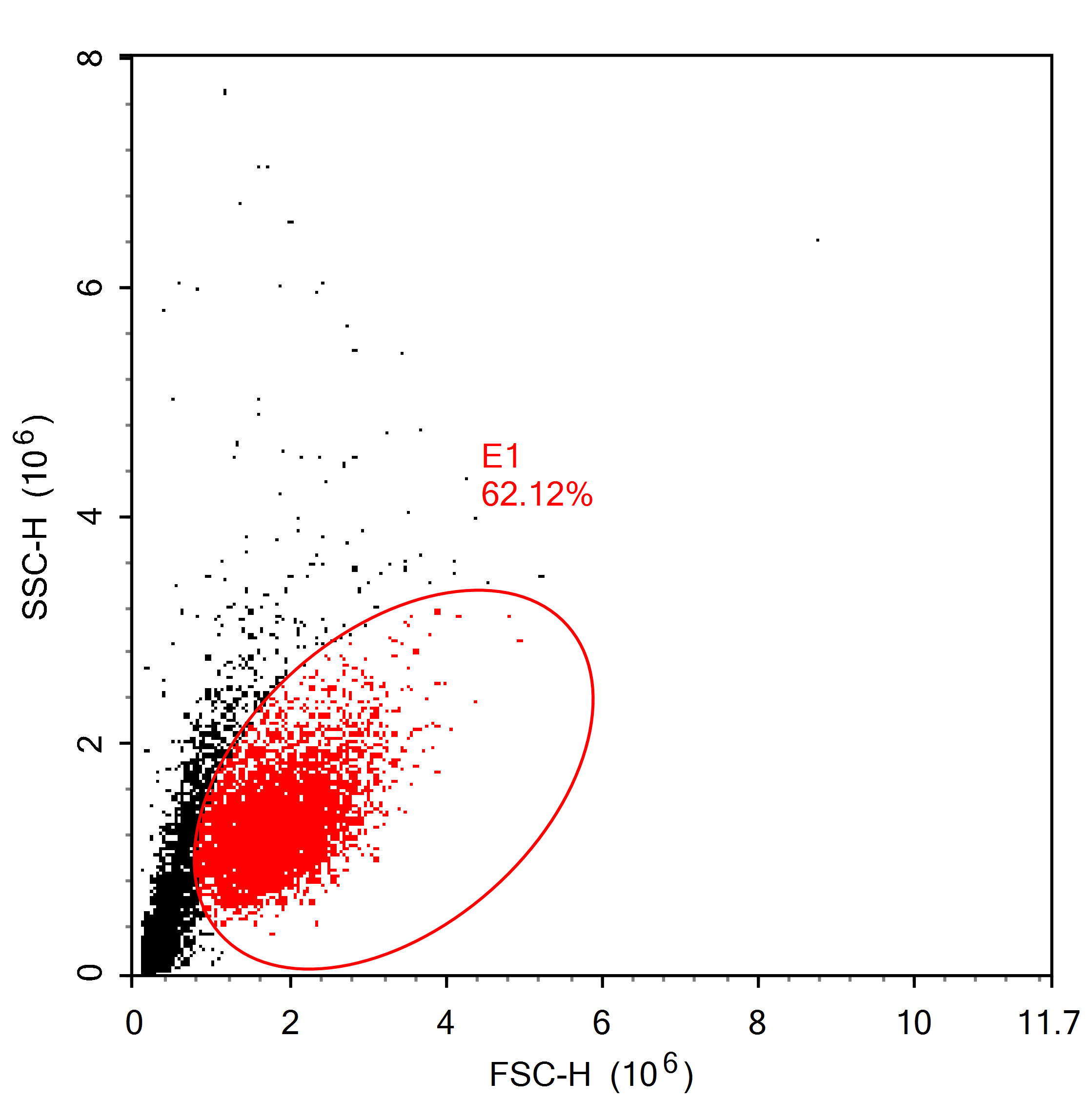

Supplement: Supplementary file 1 [file DataSheet3.zip › Flow Cytometry Assay(1,2)/Flow Cytometry Assay-1/╧╕░√╡≥═÷-1/╡≥═÷ 3/═╝╞1⁄4/4h 2/═╝1.tiff]

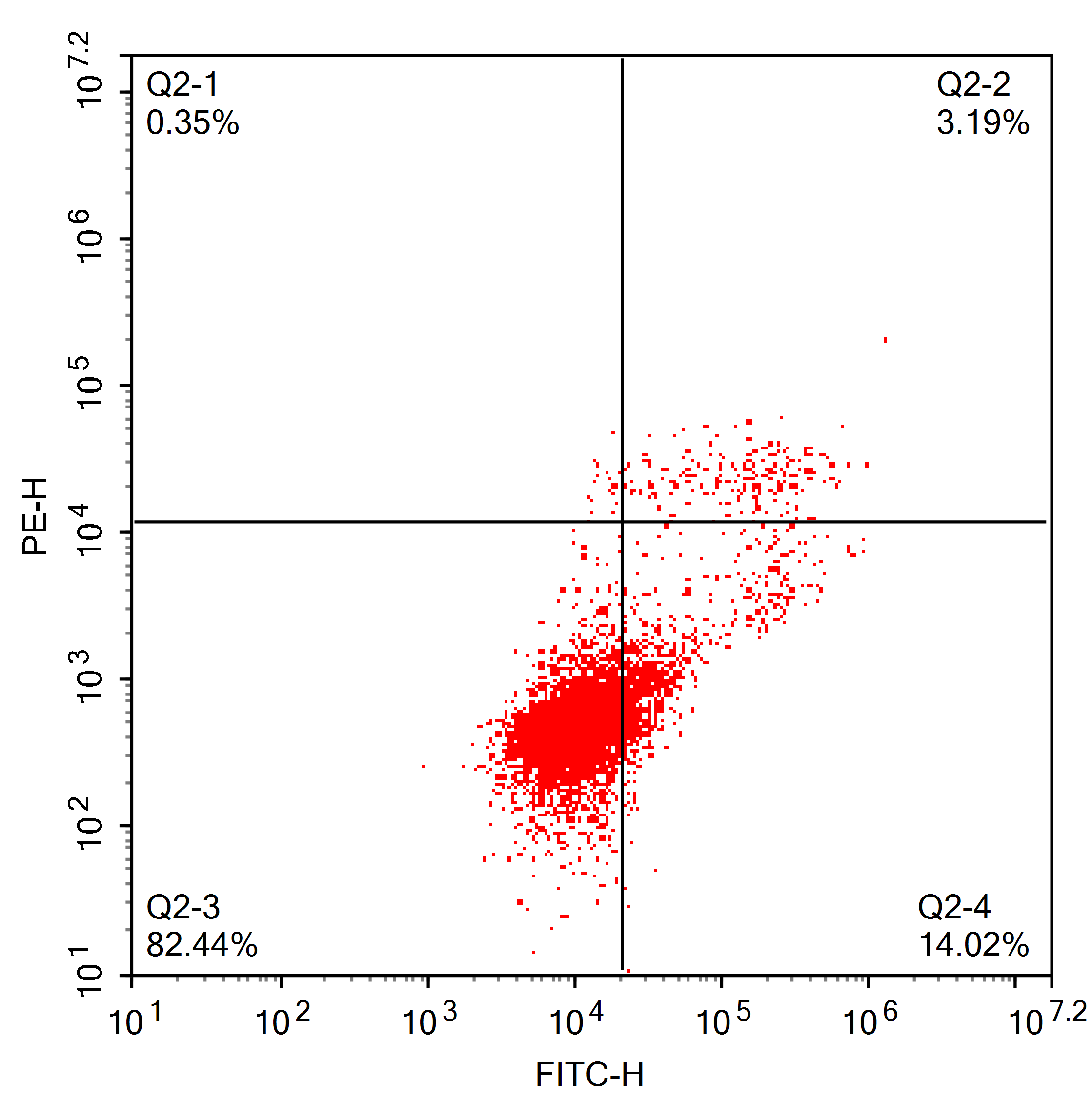

Supplement: Supplementary file 1 [file DataSheet3.zip › Flow Cytometry Assay(1,2)/Flow Cytometry Assay-1/╧╕░√╡≥═÷-1/╡≥═÷ 3/═╝╞1⁄4/4h 2/═╝2.tiff]

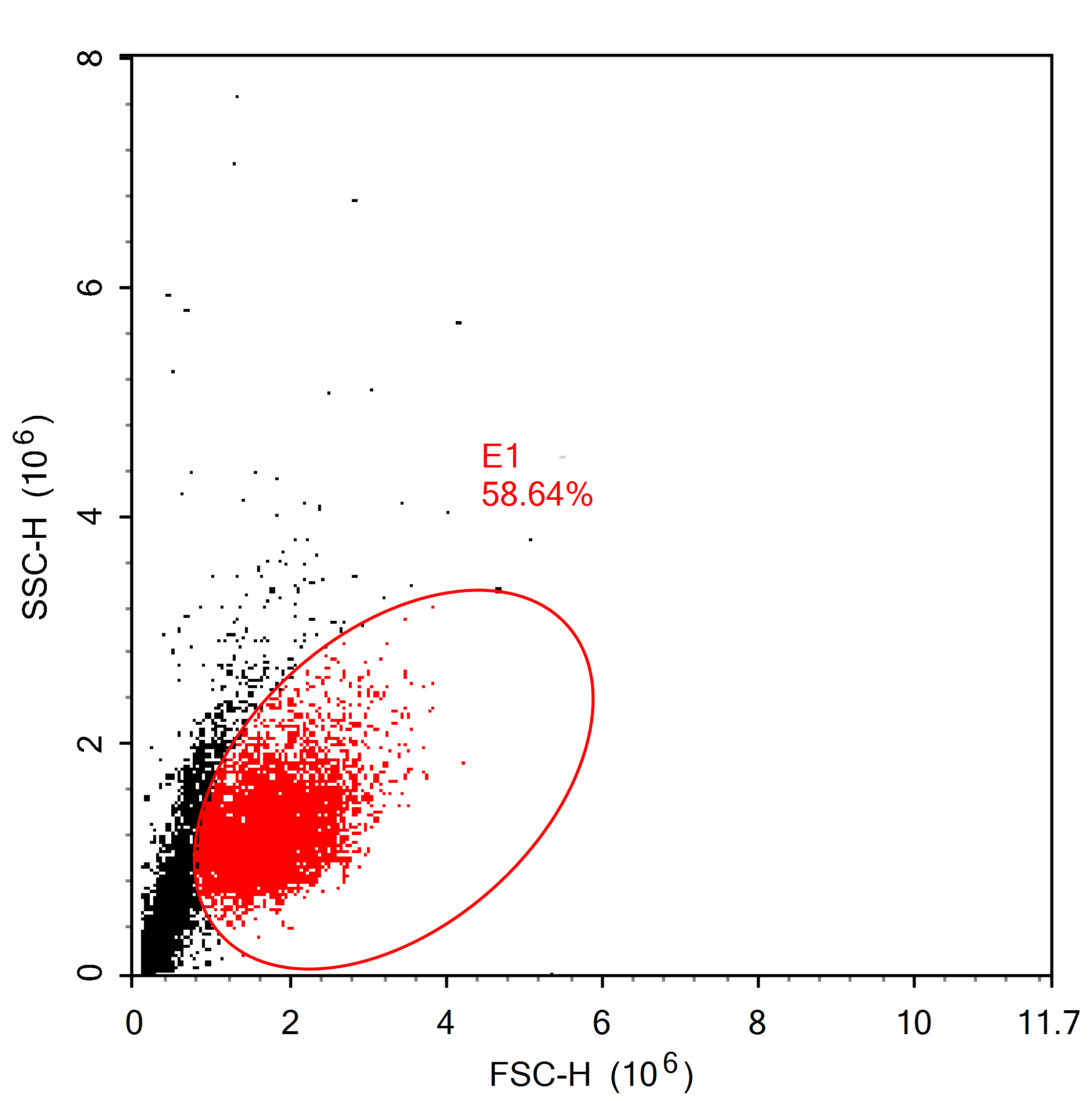

Supplement: Supplementary file 1 [file DataSheet3.zip › Flow Cytometry Assay(1,2)/Flow Cytometry Assay-1/╧╕░√╡≥═÷-1/╡≥═÷ 3/═╝╞1⁄4/4h 3/═╝1.tiff]

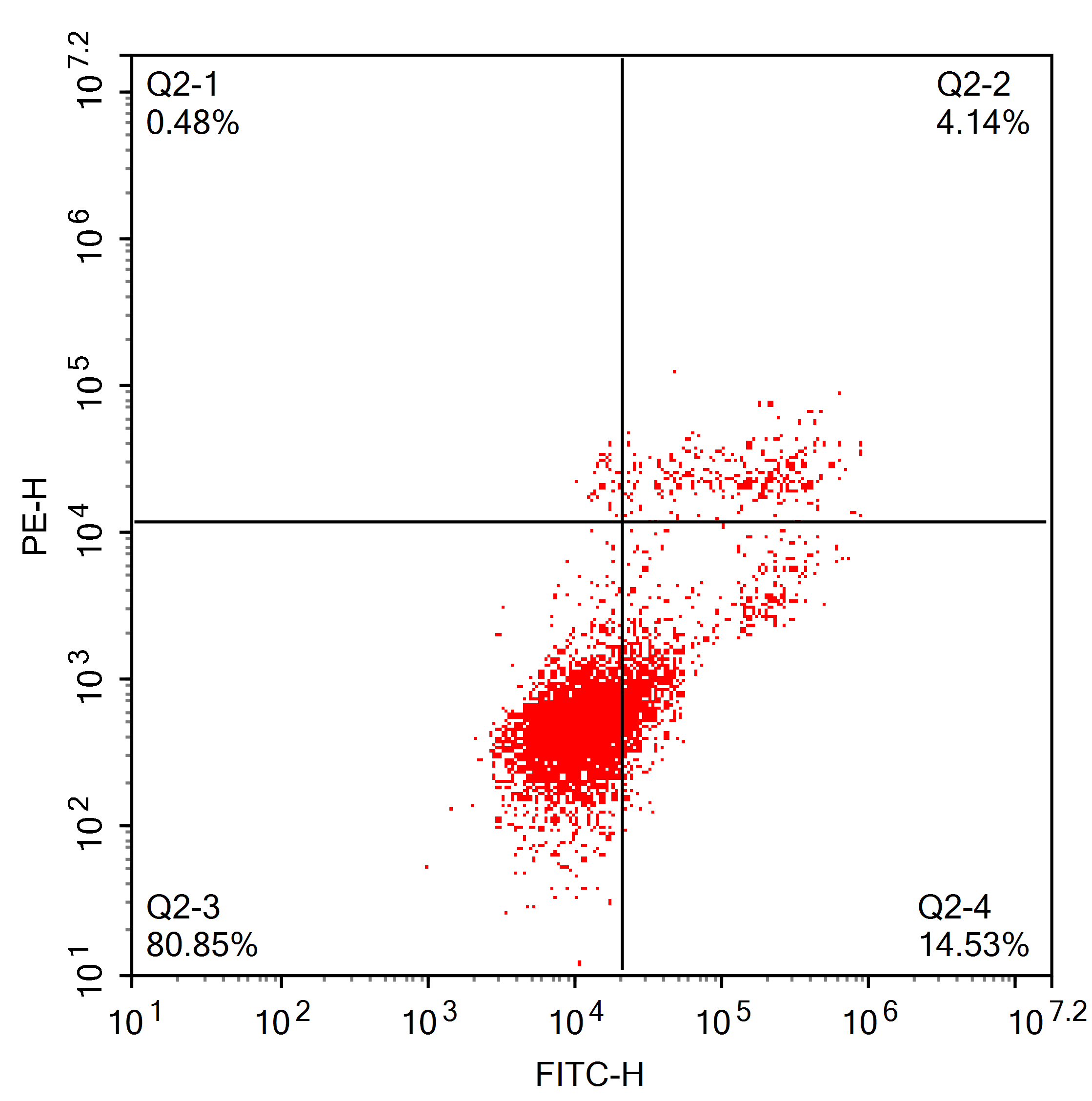

Supplement: Supplementary file 1 [file DataSheet3.zip › Flow Cytometry Assay(1,2)/Flow Cytometry Assay-1/╧╕░√╡≥═÷-1/╡≥═÷ 3/═╝╞1⁄4/4h 3/═╝2.tiff]

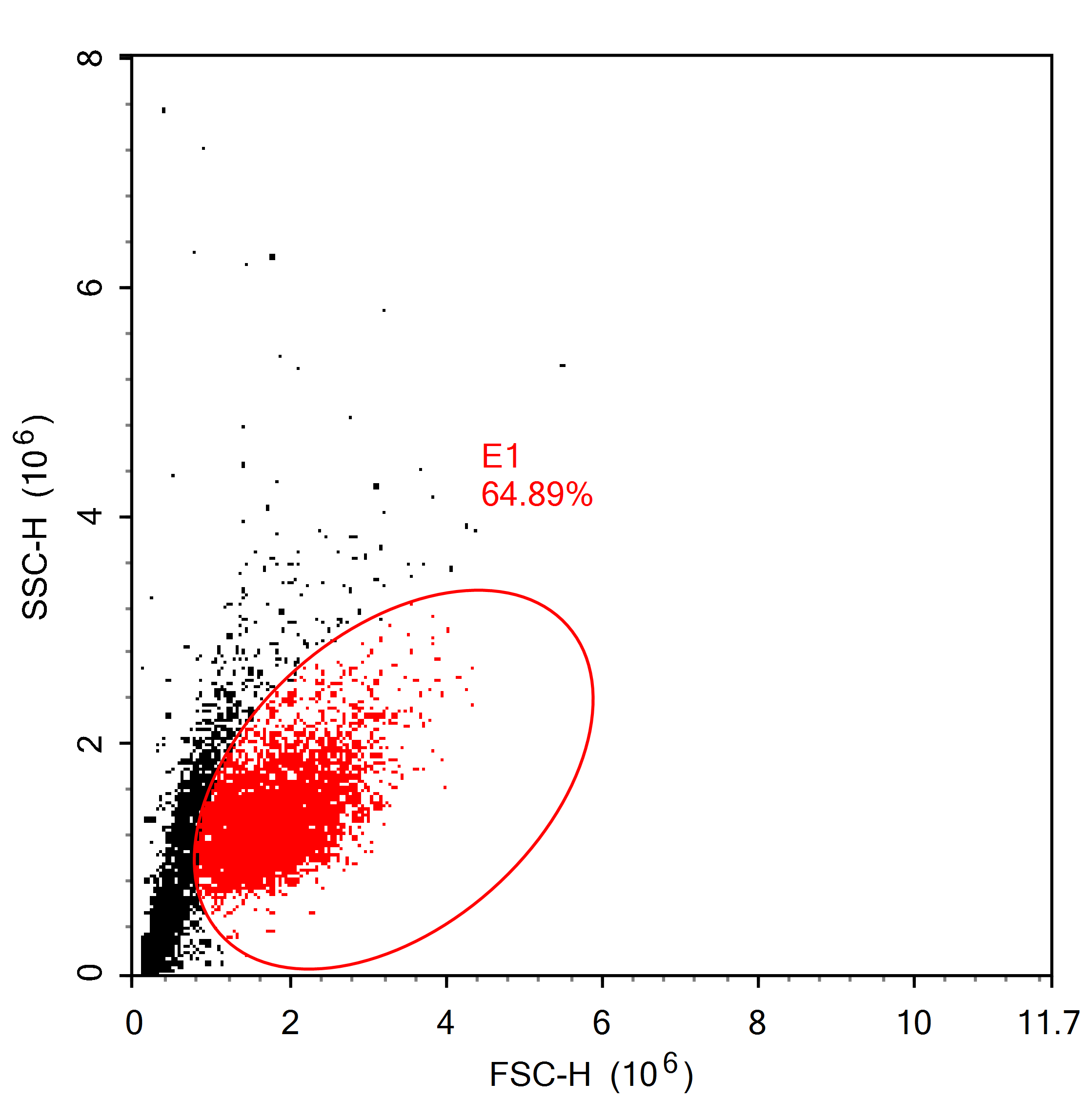

Supplement: Supplementary file 1 [file DataSheet3.zip › Flow Cytometry Assay(1,2)/Flow Cytometry Assay-1/╧╕░√╡≥═÷-1/╡≥═÷ 3/═╝╞1⁄4/8h 1/═╝1.tiff]

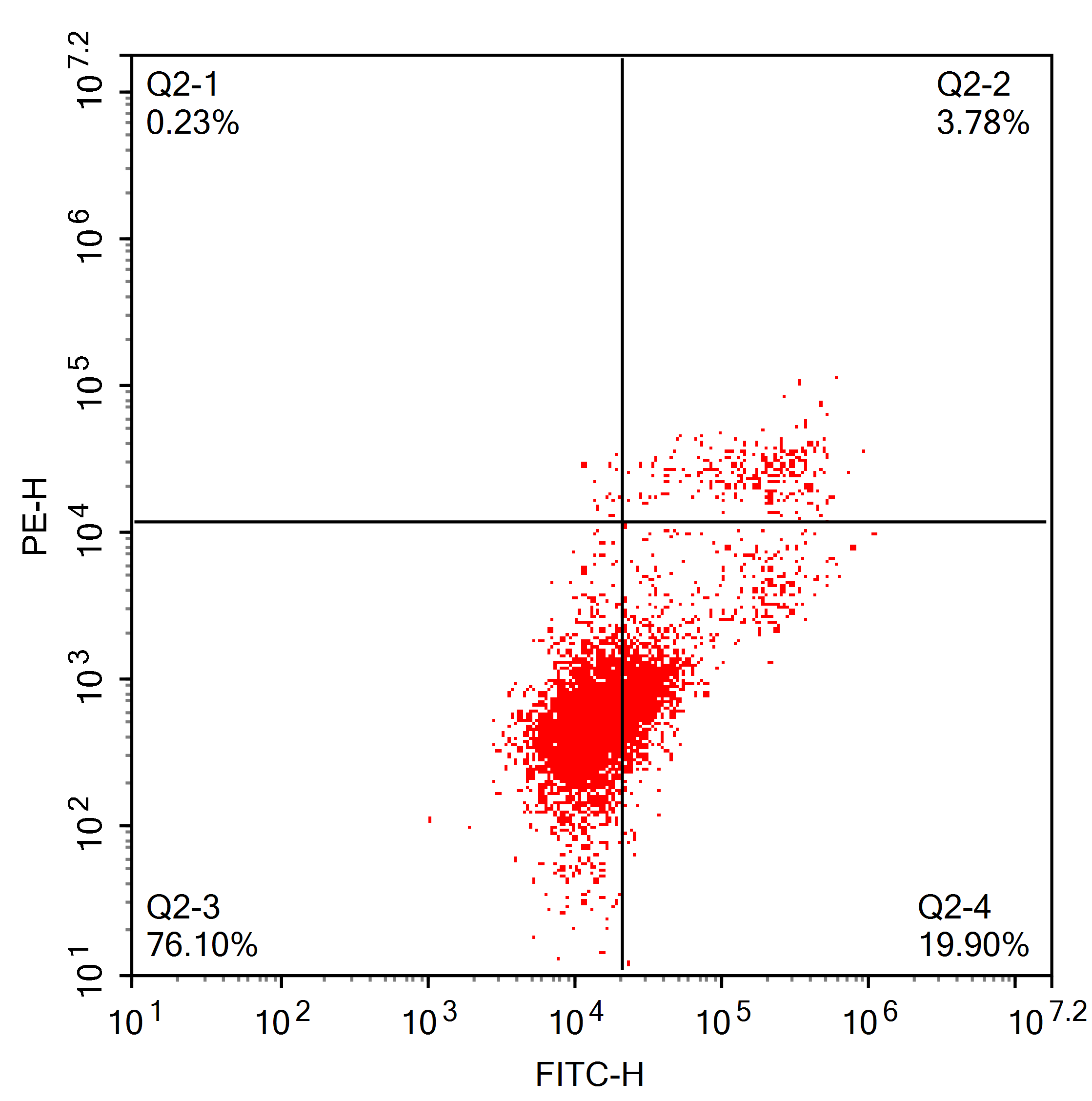

Supplement: Supplementary file 1 [file DataSheet3.zip › Flow Cytometry Assay(1,2)/Flow Cytometry Assay-1/╧╕░√╡≥═÷-1/╡≥═÷ 3/═╝╞1⁄4/8h 1/═╝2.tiff]

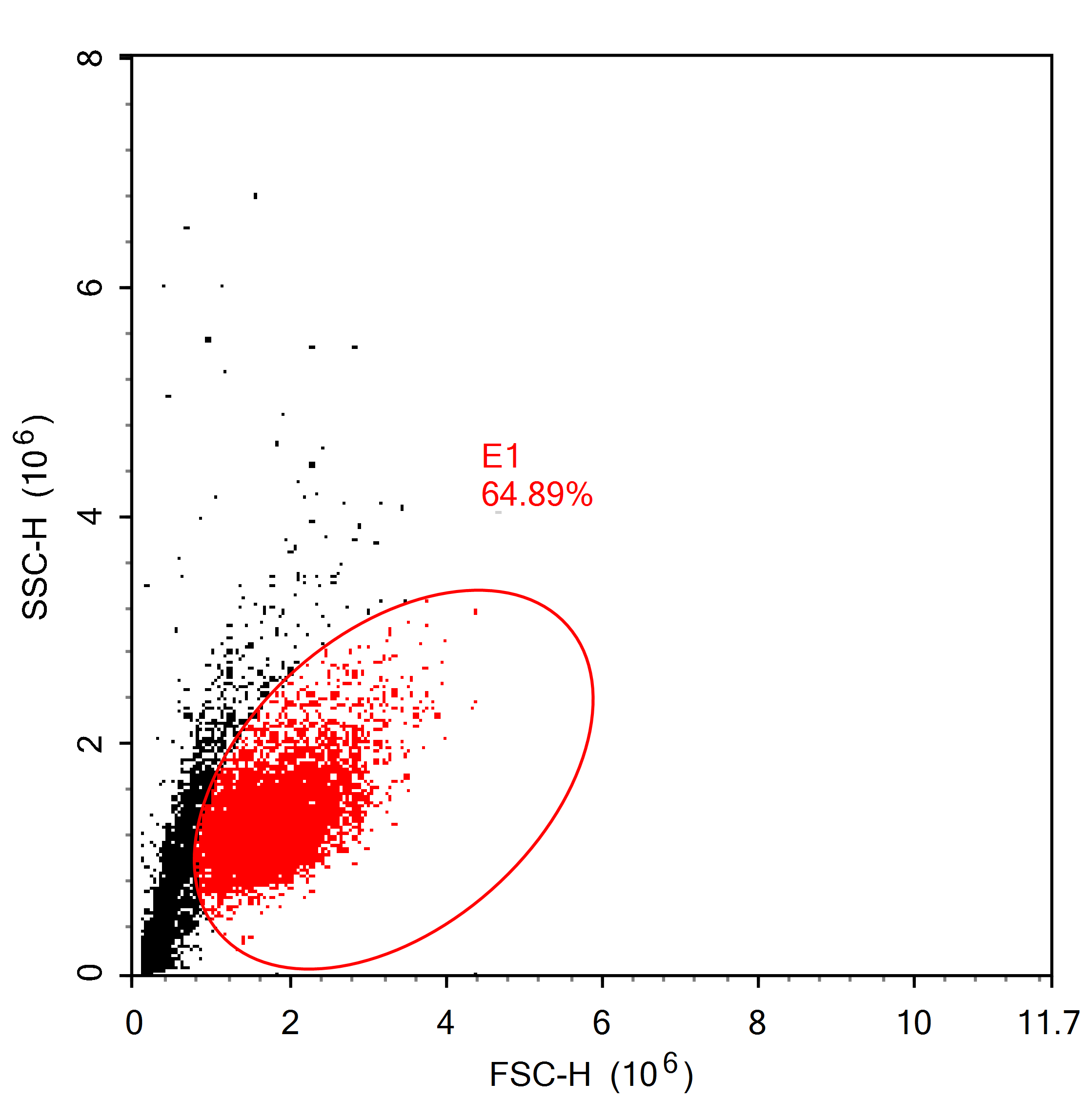

Supplement: Supplementary file 1 [file DataSheet3.zip › Flow Cytometry Assay(1,2)/Flow Cytometry Assay-1/╧╕░√╡≥═÷-1/╡≥═÷ 3/═╝╞1⁄4/8h 2/═╝1.tiff]

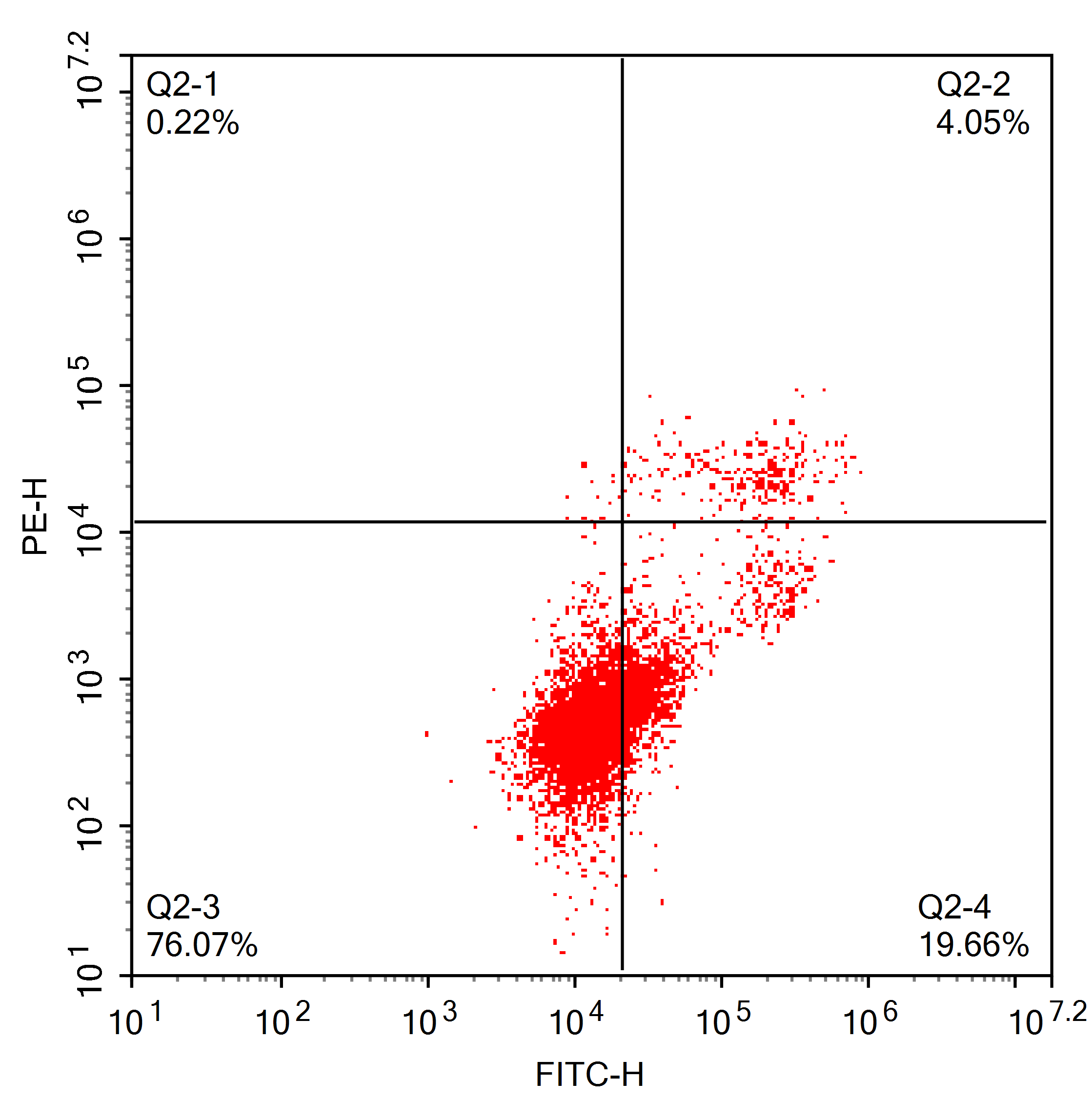

Supplement: Supplementary file 1 [file DataSheet3.zip › Flow Cytometry Assay(1,2)/Flow Cytometry Assay-1/╧╕░√╡≥═÷-1/╡≥═÷ 3/═╝╞1⁄4/8h 2/═╝2.tiff]

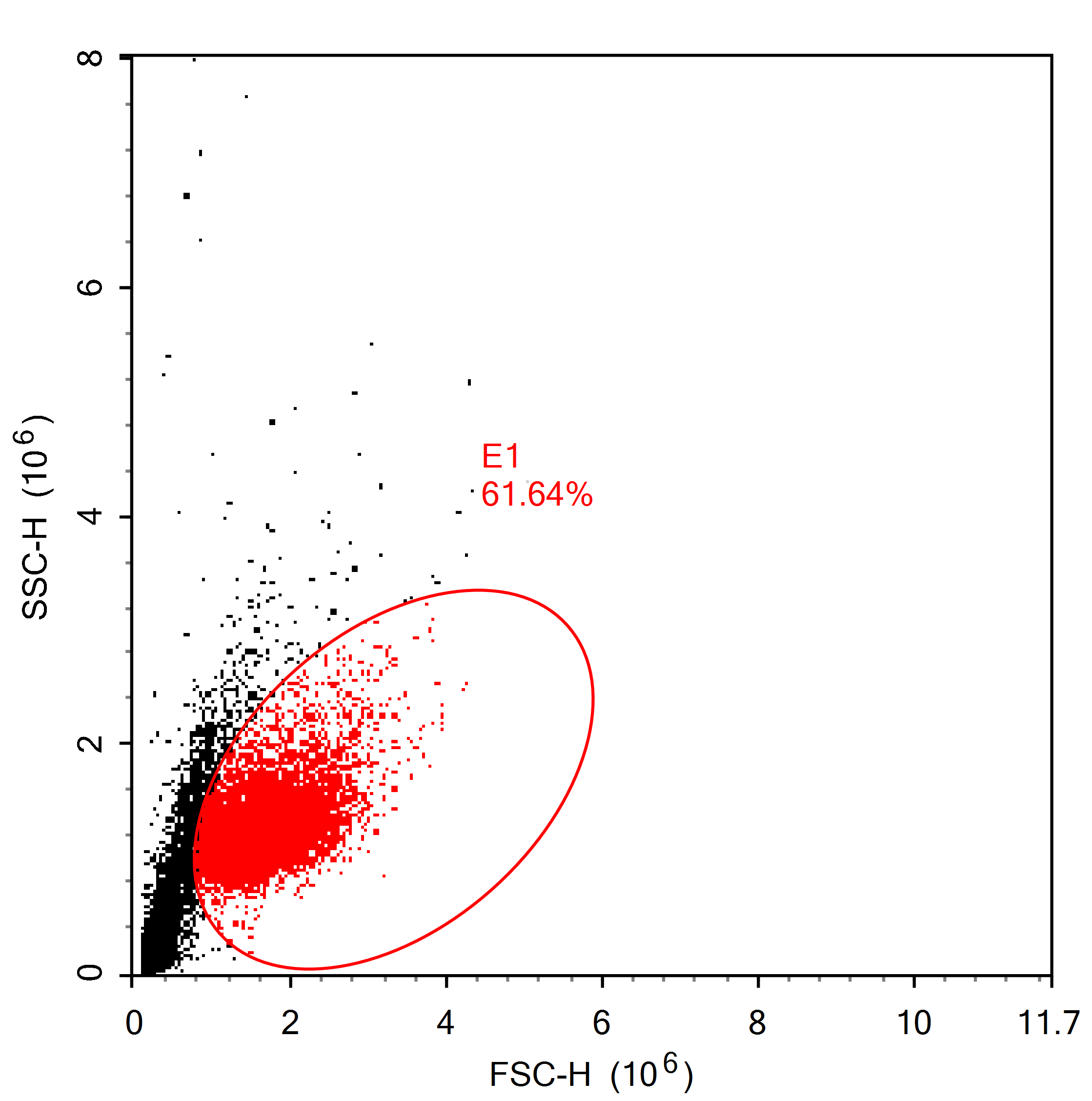

Supplement: Supplementary file 1 [file DataSheet3.zip › Flow Cytometry Assay(1,2)/Flow Cytometry Assay-1/╧╕░√╡≥═÷-1/╡≥═÷ 3/═╝╞1⁄4/8h 3/═╝1.tiff]

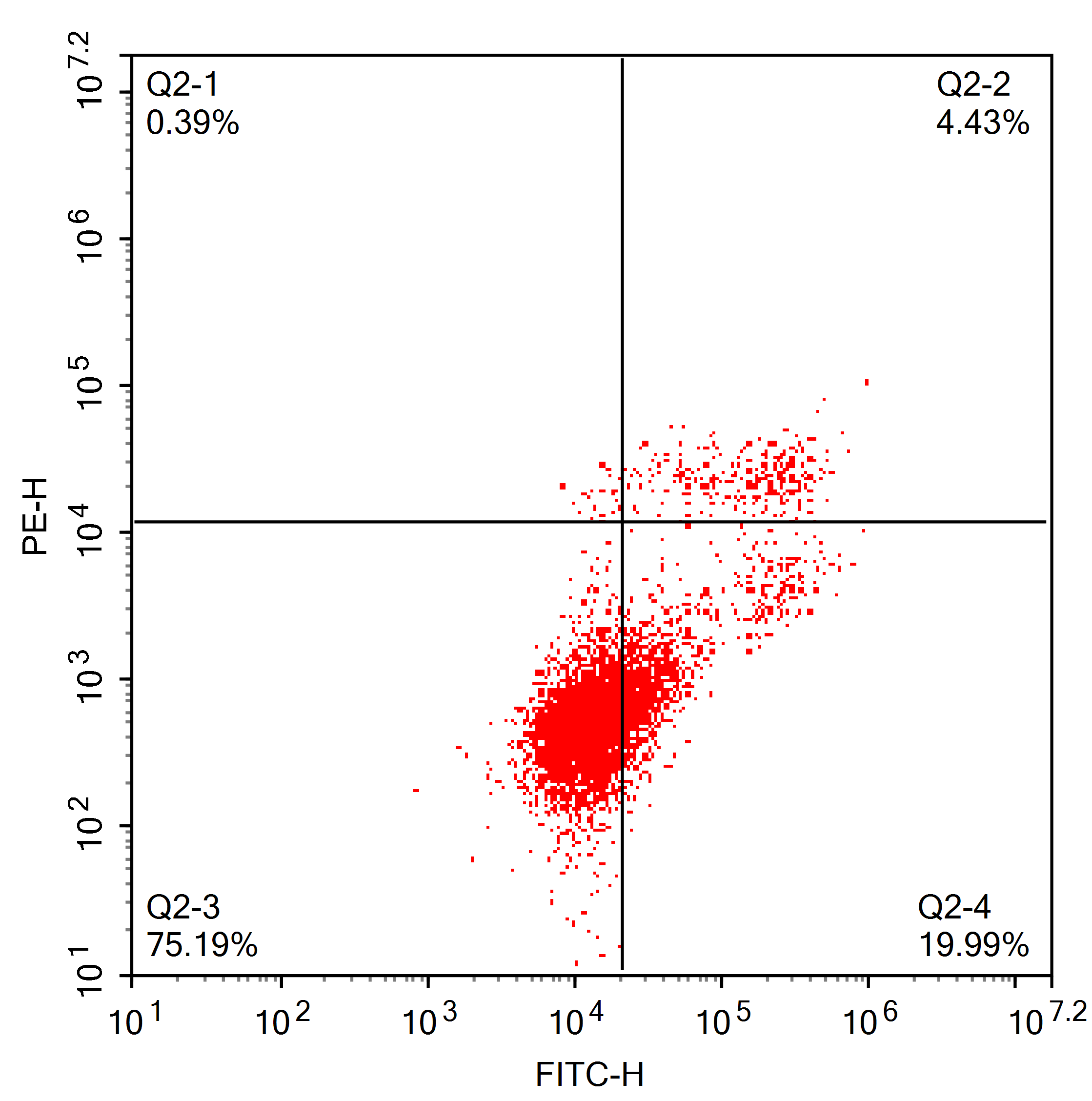

Supplement: Supplementary file 1 [file DataSheet3.zip › Flow Cytometry Assay(1,2)/Flow Cytometry Assay-1/╧╕░√╡≥═÷-1/╡≥═÷ 3/═╝╞1⁄4/8h 3/═╝2.tiff]

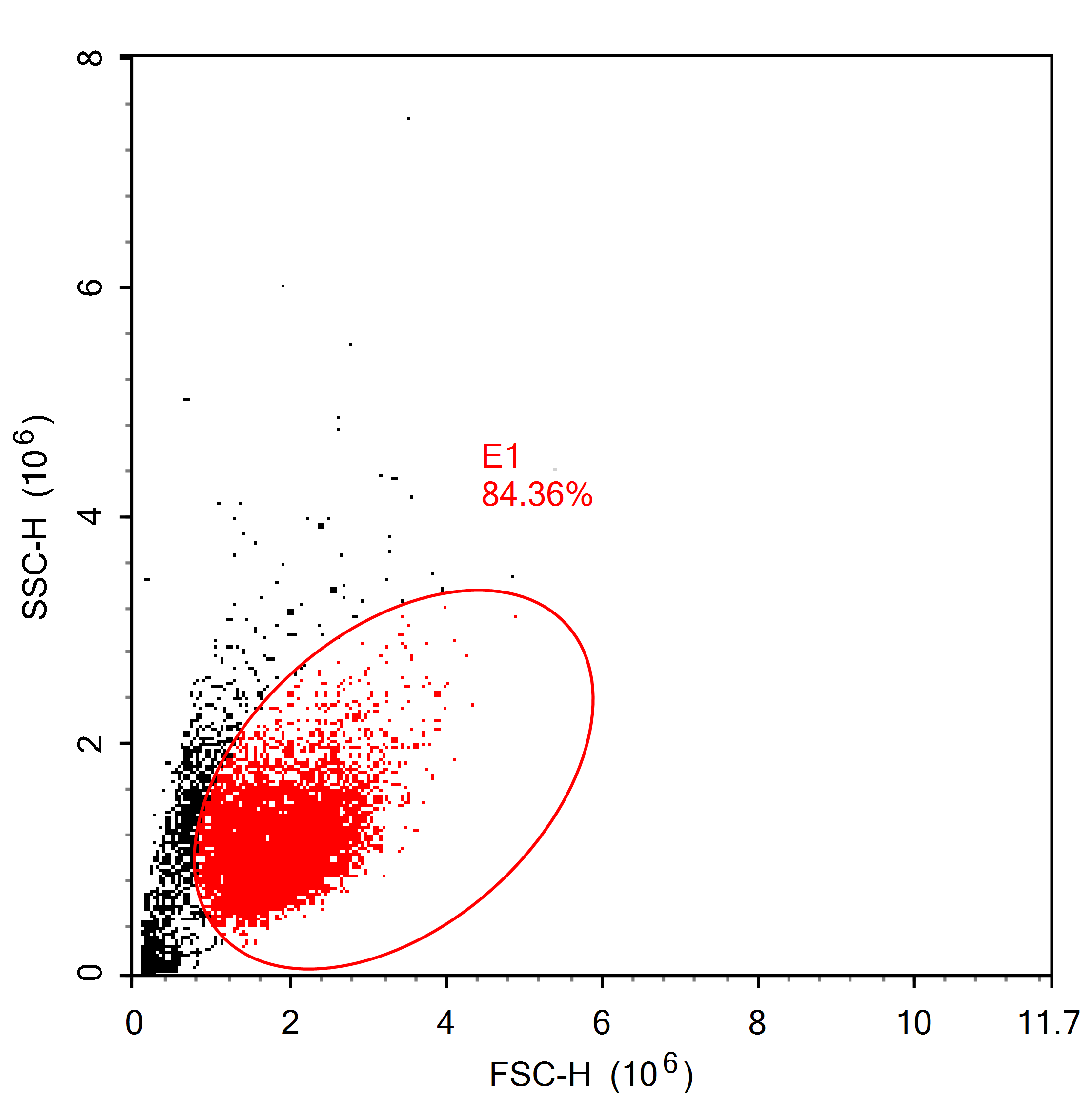

Supplement: Supplementary file 1 [file DataSheet3.zip › Flow Cytometry Assay(1,2)/Flow Cytometry Assay-1/╧╕░√╡≥═÷-1/╡≥═÷ 3/═╝╞1⁄4/Blank/═╝1.tiff]

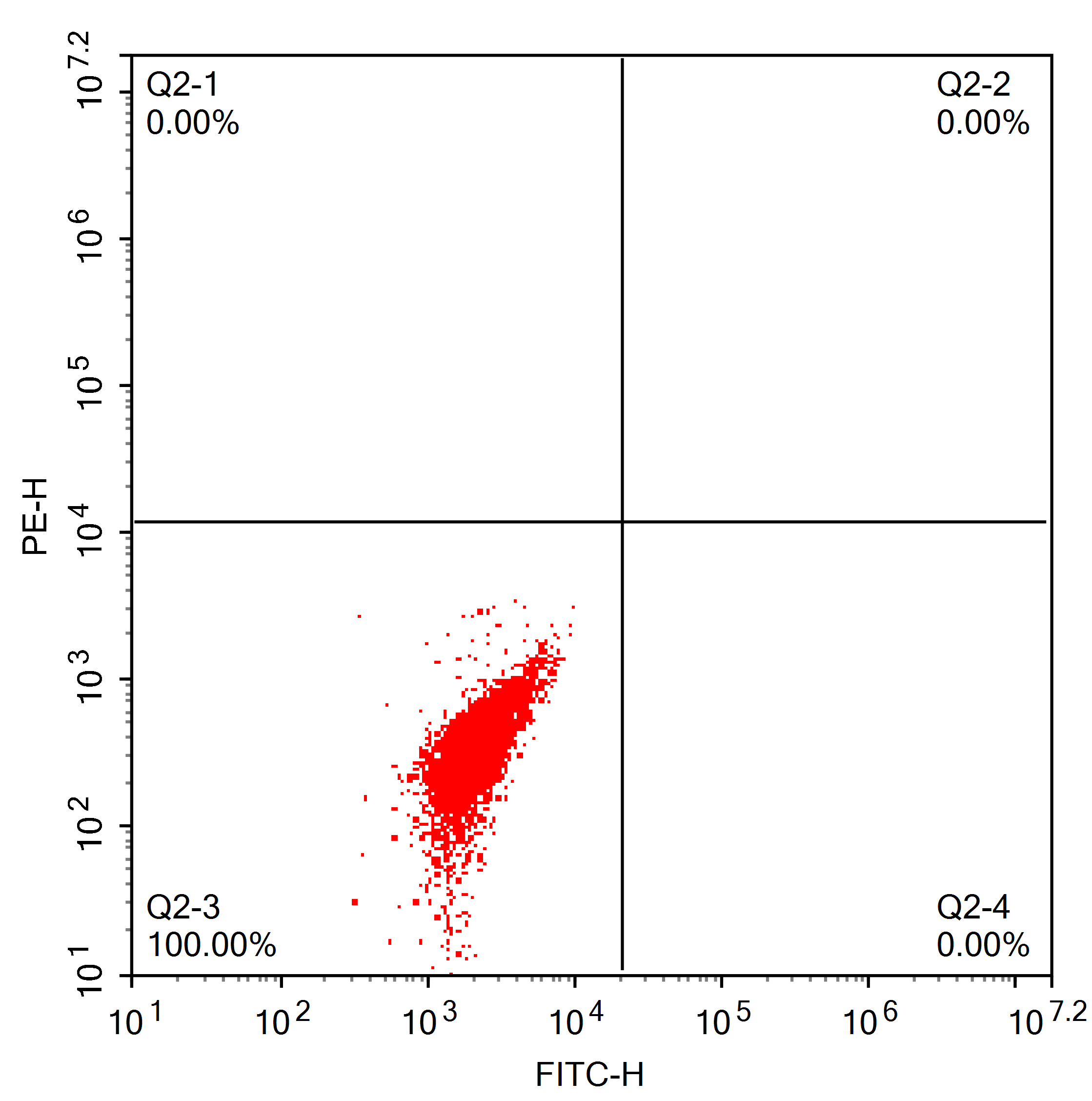

Supplement: Supplementary file 1 [file DataSheet3.zip › Flow Cytometry Assay(1,2)/Flow Cytometry Assay-1/╧╕░√╡≥═÷-1/╡≥═÷ 3/═╝╞1⁄4/Blank/═╝2.tiff]

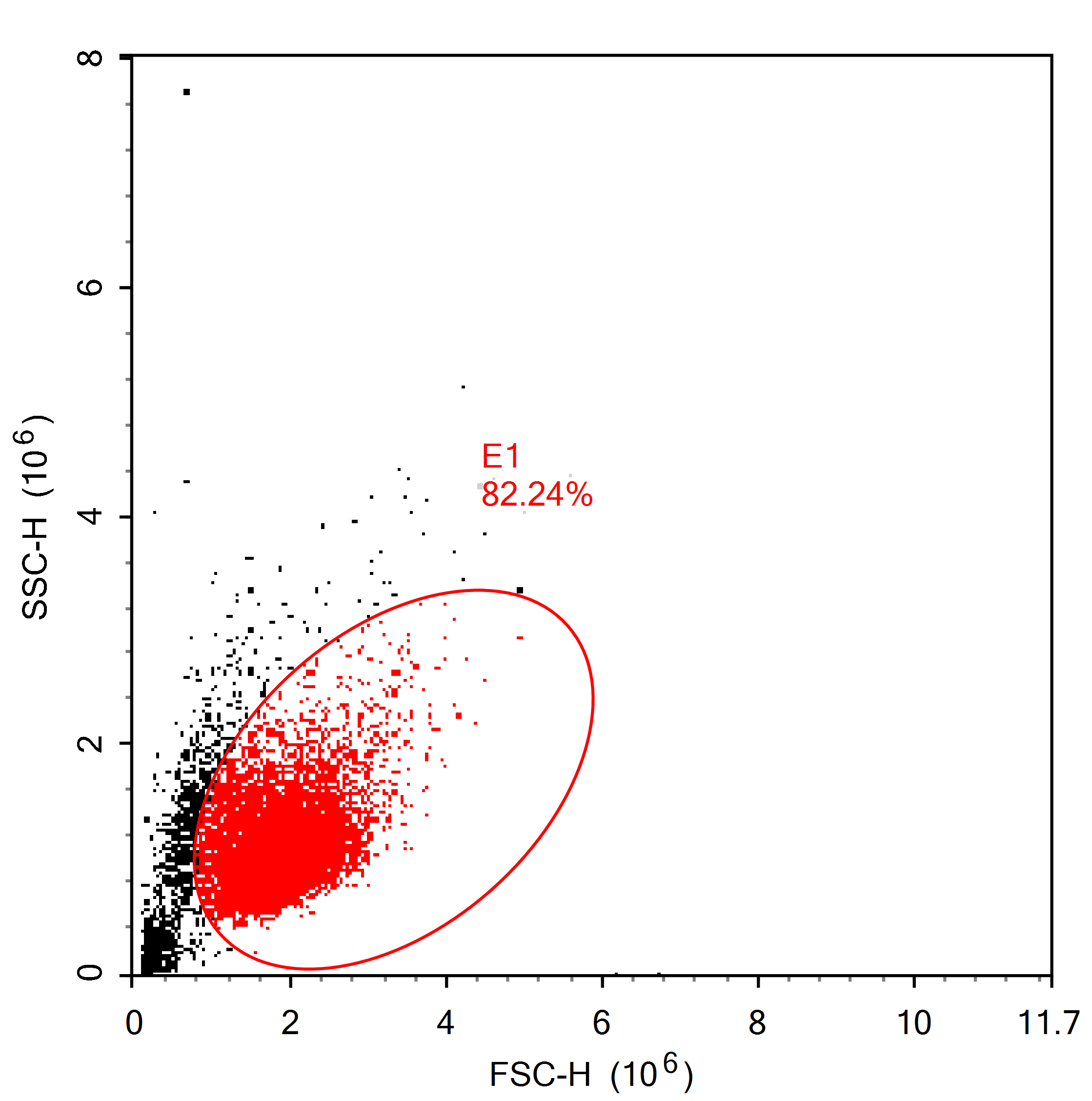

Supplement: Supplementary file 1 [file DataSheet3.zip › Flow Cytometry Assay(1,2)/Flow Cytometry Assay-1/╧╕░√╡≥═÷-1/╡≥═÷ 3/═╝╞1⁄4/Ctrl 1/═╝1.tiff]

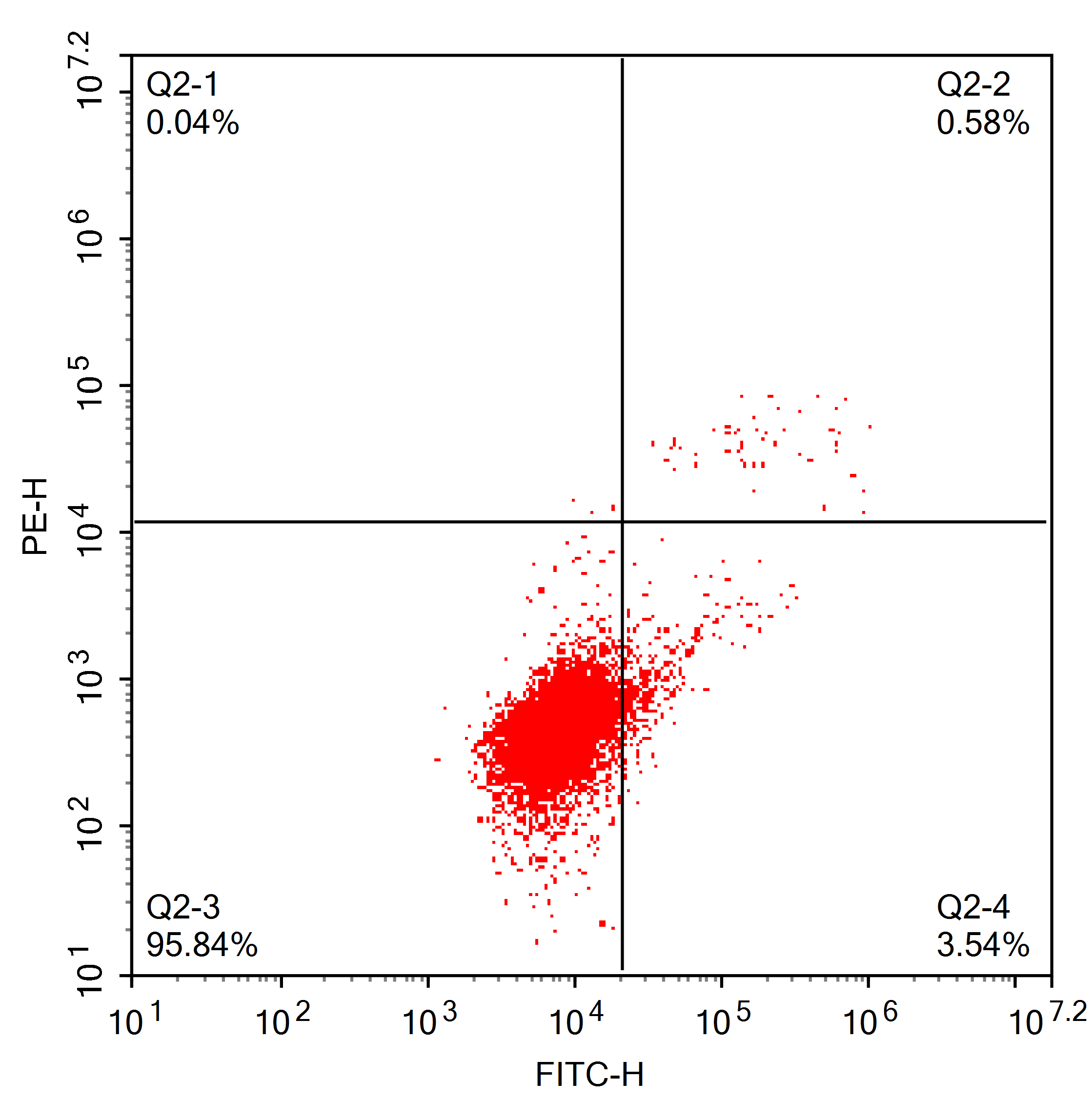

Supplement: Supplementary file 1 [file DataSheet3.zip › Flow Cytometry Assay(1,2)/Flow Cytometry Assay-1/╧╕░√╡≥═÷-1/╡≥═÷ 3/═╝╞1⁄4/Ctrl 1/═╝2.tiff]

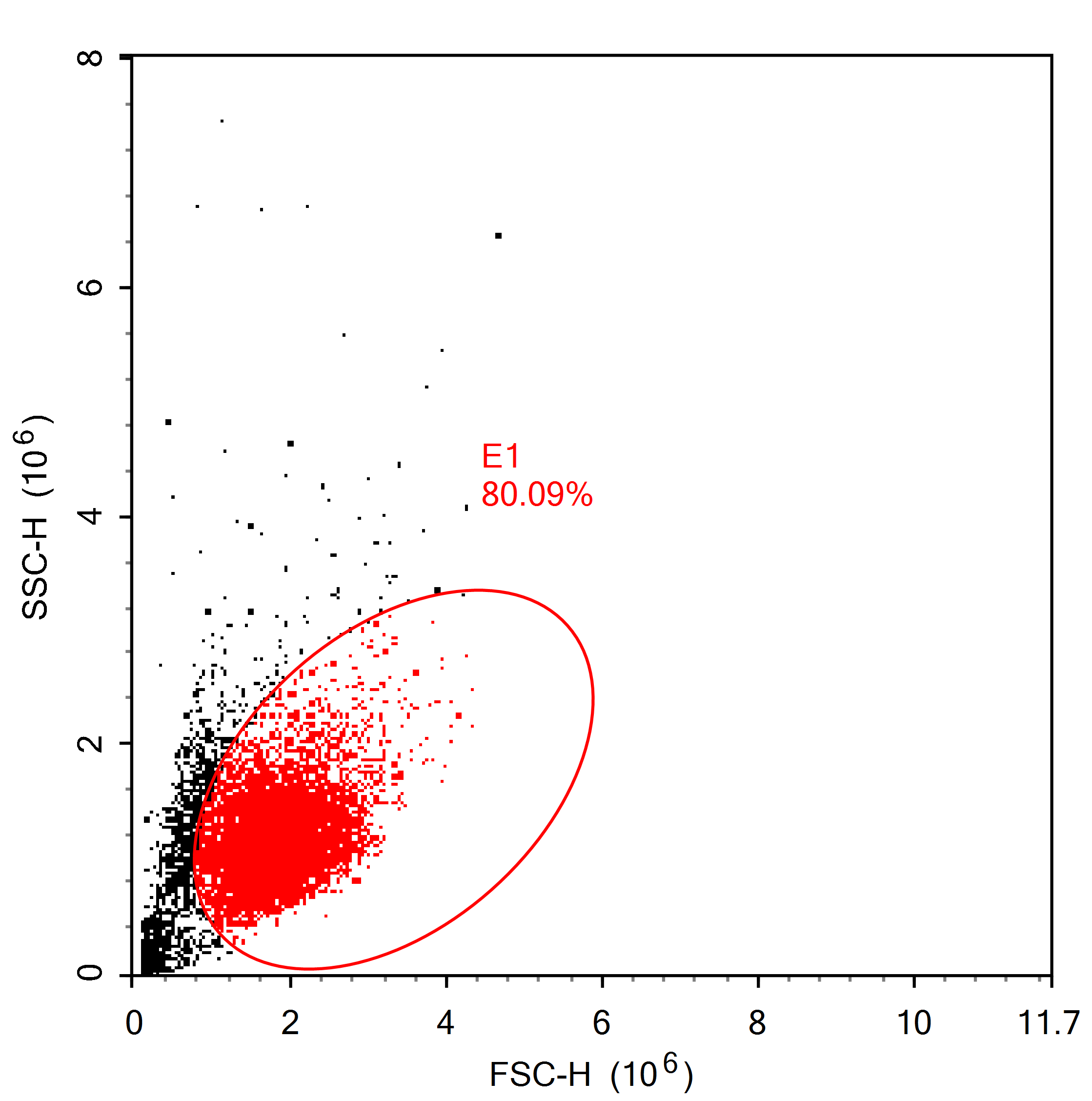

Supplement: Supplementary file 1 [file DataSheet3.zip › Flow Cytometry Assay(1,2)/Flow Cytometry Assay-1/╧╕░√╡≥═÷-1/╡≥═÷ 3/═╝╞1⁄4/Ctrl 2/═╝1.tiff]

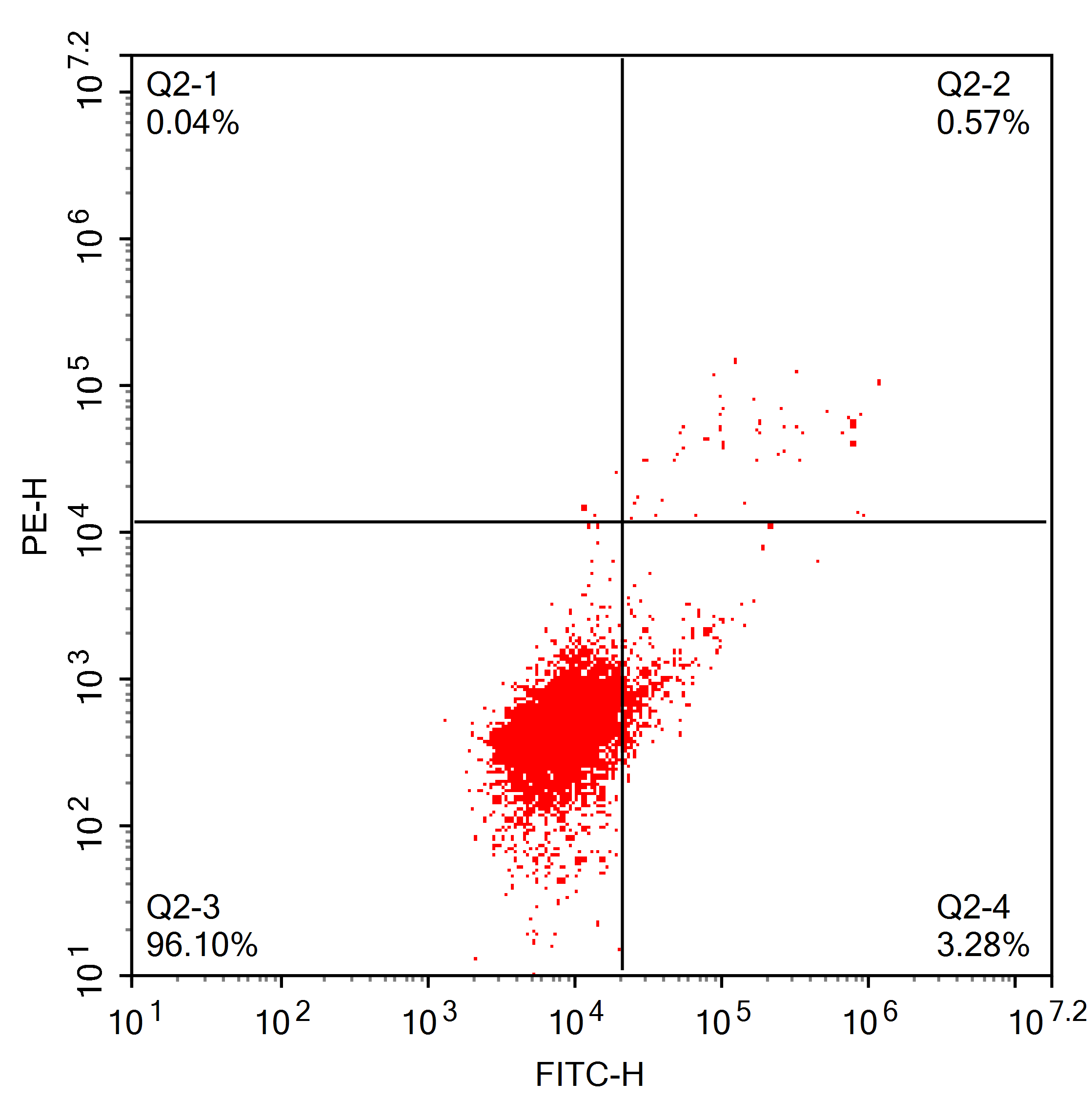

Supplement: Supplementary file 1 [file DataSheet3.zip › Flow Cytometry Assay(1,2)/Flow Cytometry Assay-1/╧╕░√╡≥═÷-1/╡≥═÷ 3/═╝╞1⁄4/Ctrl 2/═╝2.tiff]

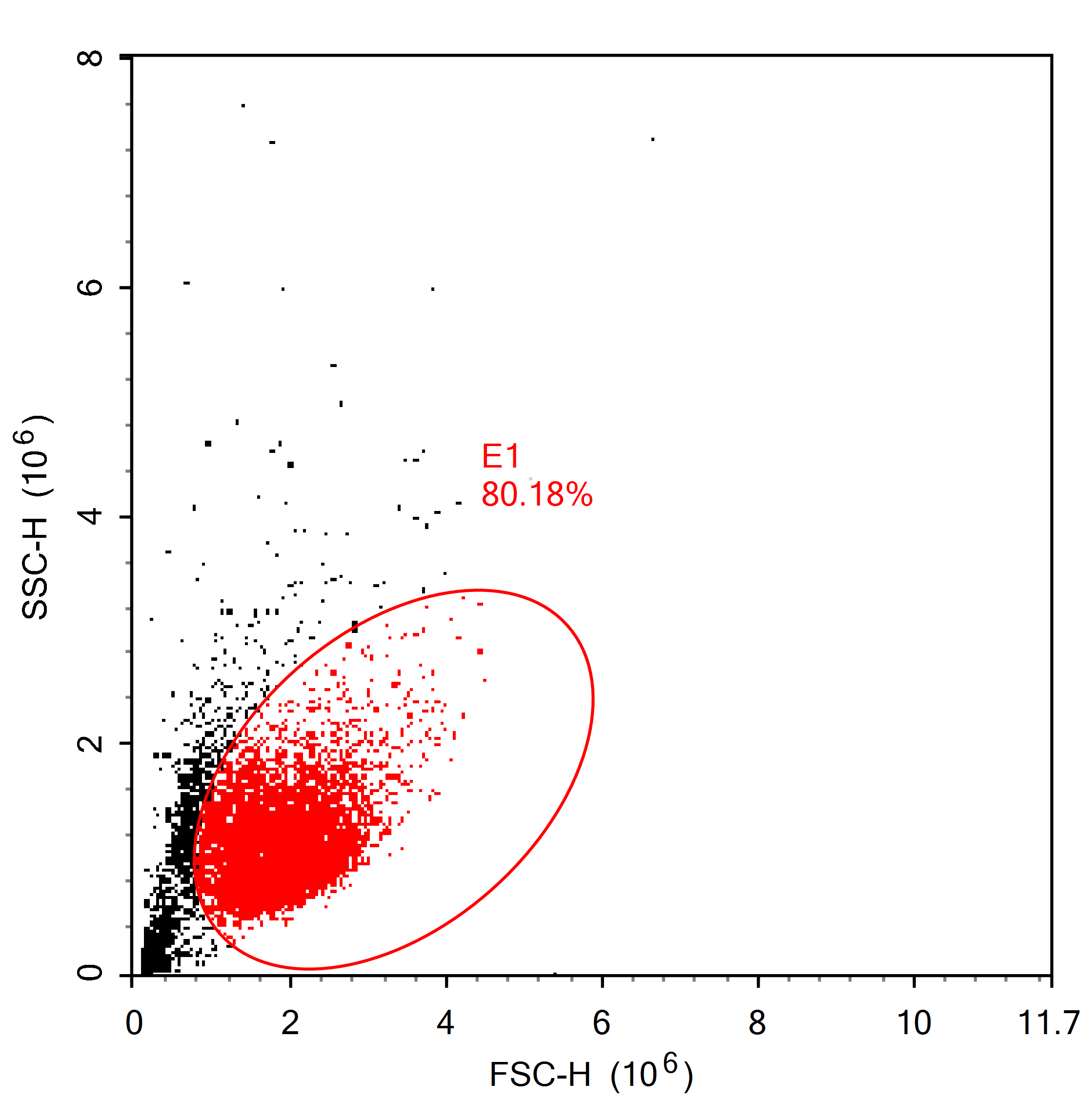

Supplement: Supplementary file 1 [file DataSheet3.zip › Flow Cytometry Assay(1,2)/Flow Cytometry Assay-1/╧╕░√╡≥═÷-1/╡≥═÷ 3/═╝╞1⁄4/Ctrl 3/═╝1.tiff]

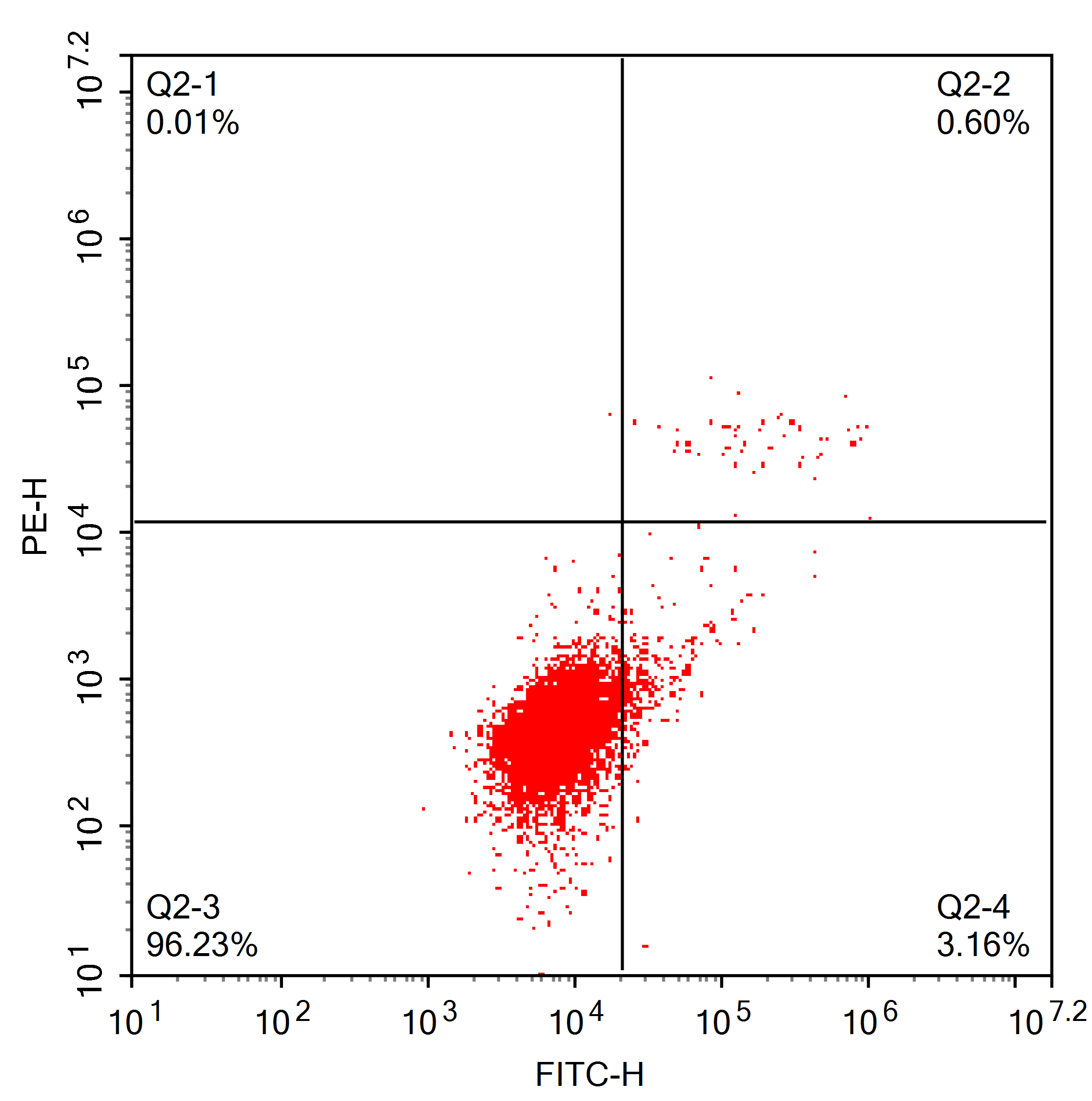

Supplement: Supplementary file 1 [file DataSheet3.zip › Flow Cytometry Assay(1,2)/Flow Cytometry Assay-1/╧╕░√╡≥═÷-1/╡≥═÷ 3/═╝╞1⁄4/Ctrl 3/═╝2.tiff]

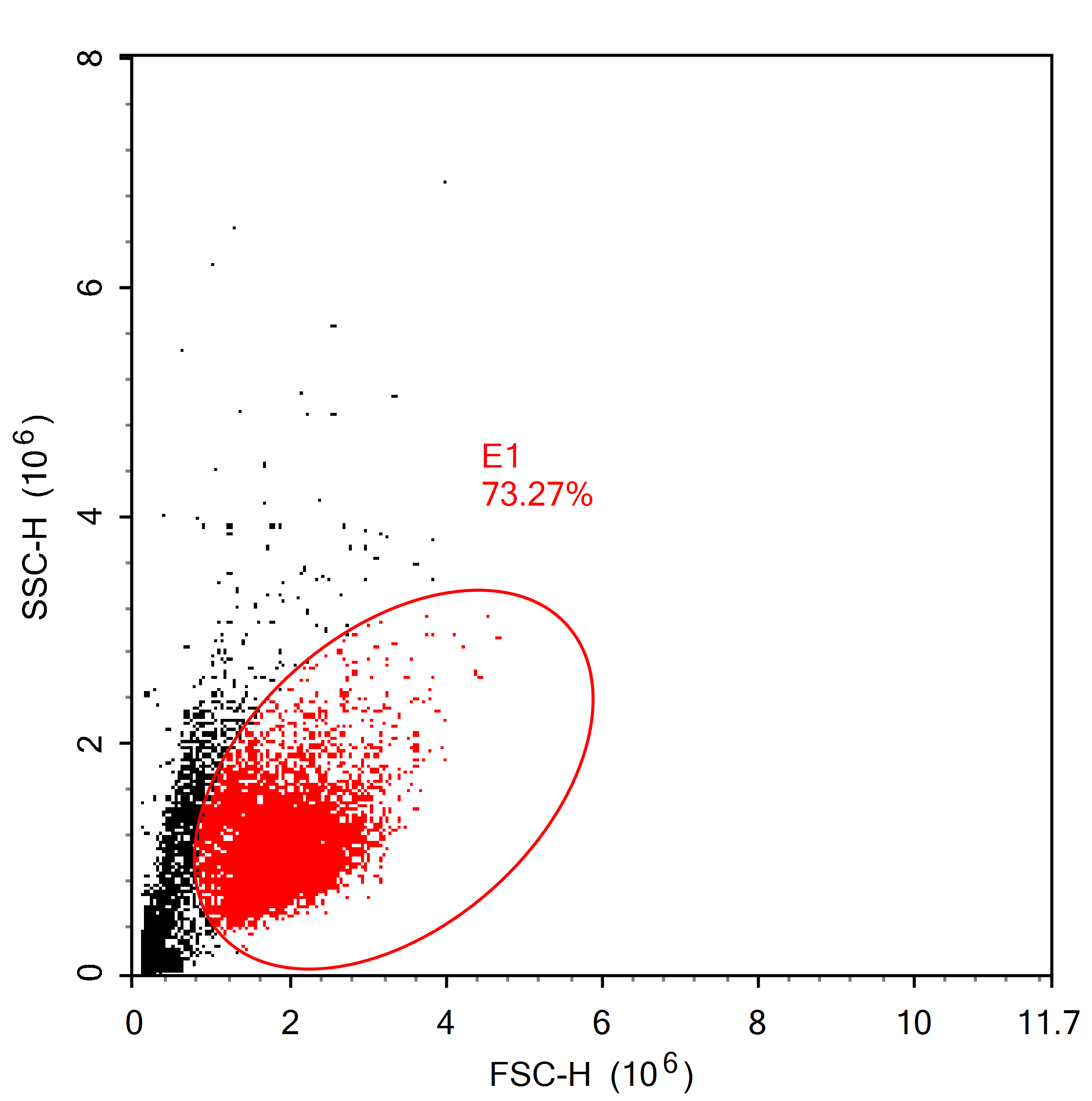

Supplement: Supplementary file 1 [file DataSheet3.zip › Flow Cytometry Assay(1,2)/Flow Cytometry Assay-1/╧╕░√╡≥═÷-1/╡≥═÷ 3/═╝╞1⁄4/FITC/═╝1.tiff]

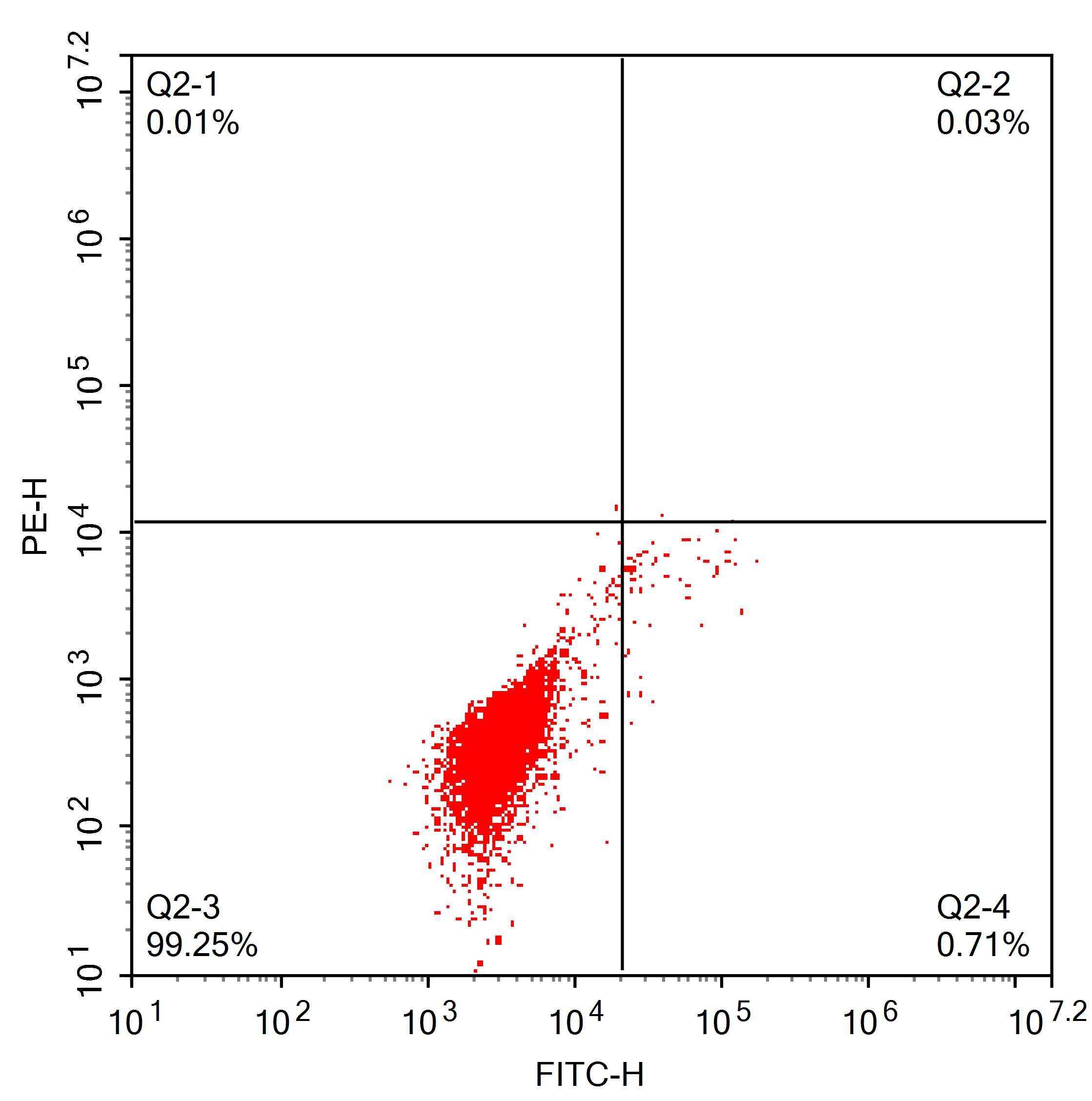

Supplement: Supplementary file 1 [file DataSheet3.zip › Flow Cytometry Assay(1,2)/Flow Cytometry Assay-1/╧╕░√╡≥═÷-1/╡≥═÷ 3/═╝╞1⁄4/FITC/═╝2.tiff]

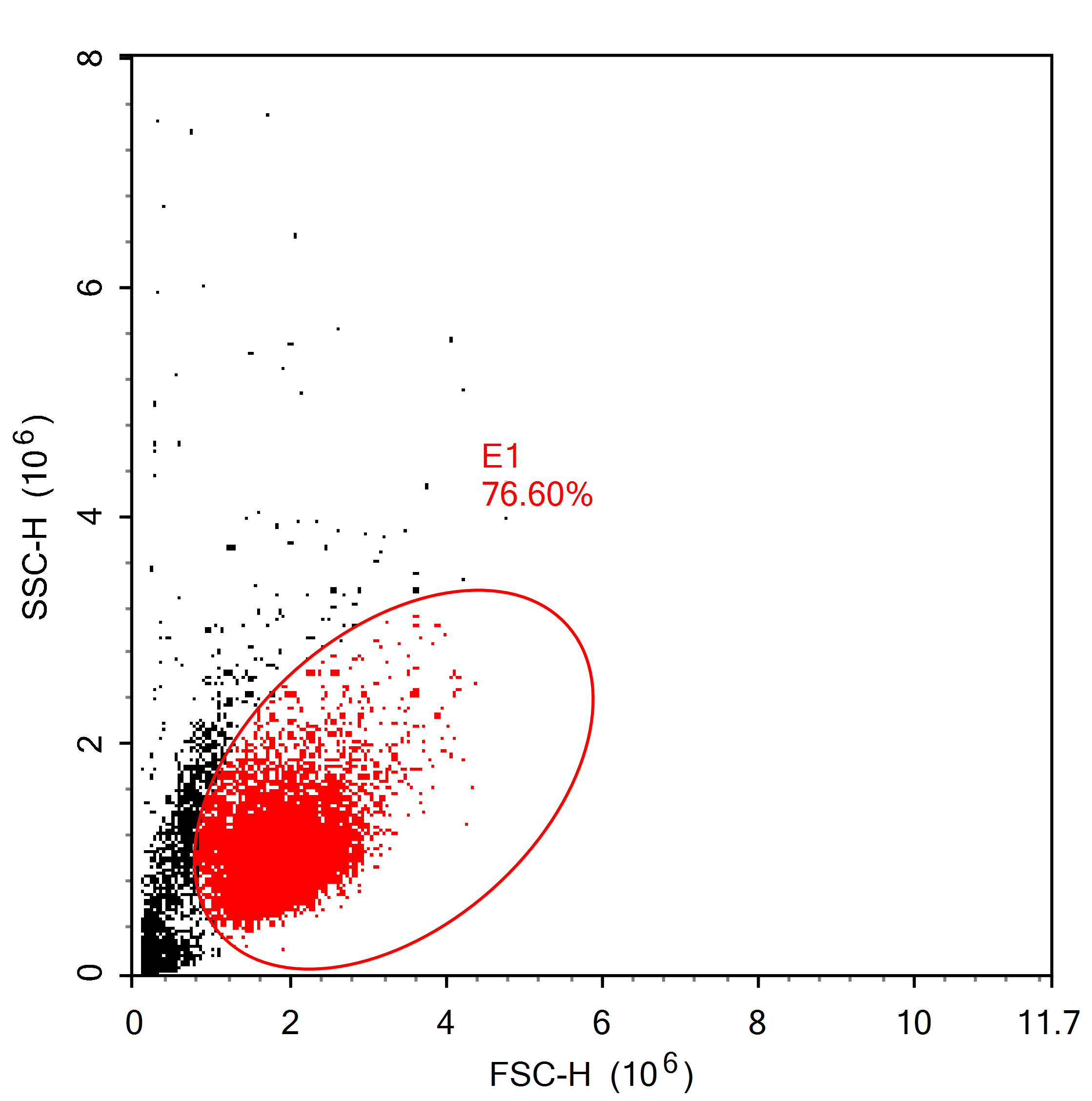

Supplement: Supplementary file 1 [file DataSheet3.zip › Flow Cytometry Assay(1,2)/Flow Cytometry Assay-1/╧╕░√╡≥═÷-1/╡≥═÷ 3/═╝╞1⁄4/PI/═╝1.tiff]

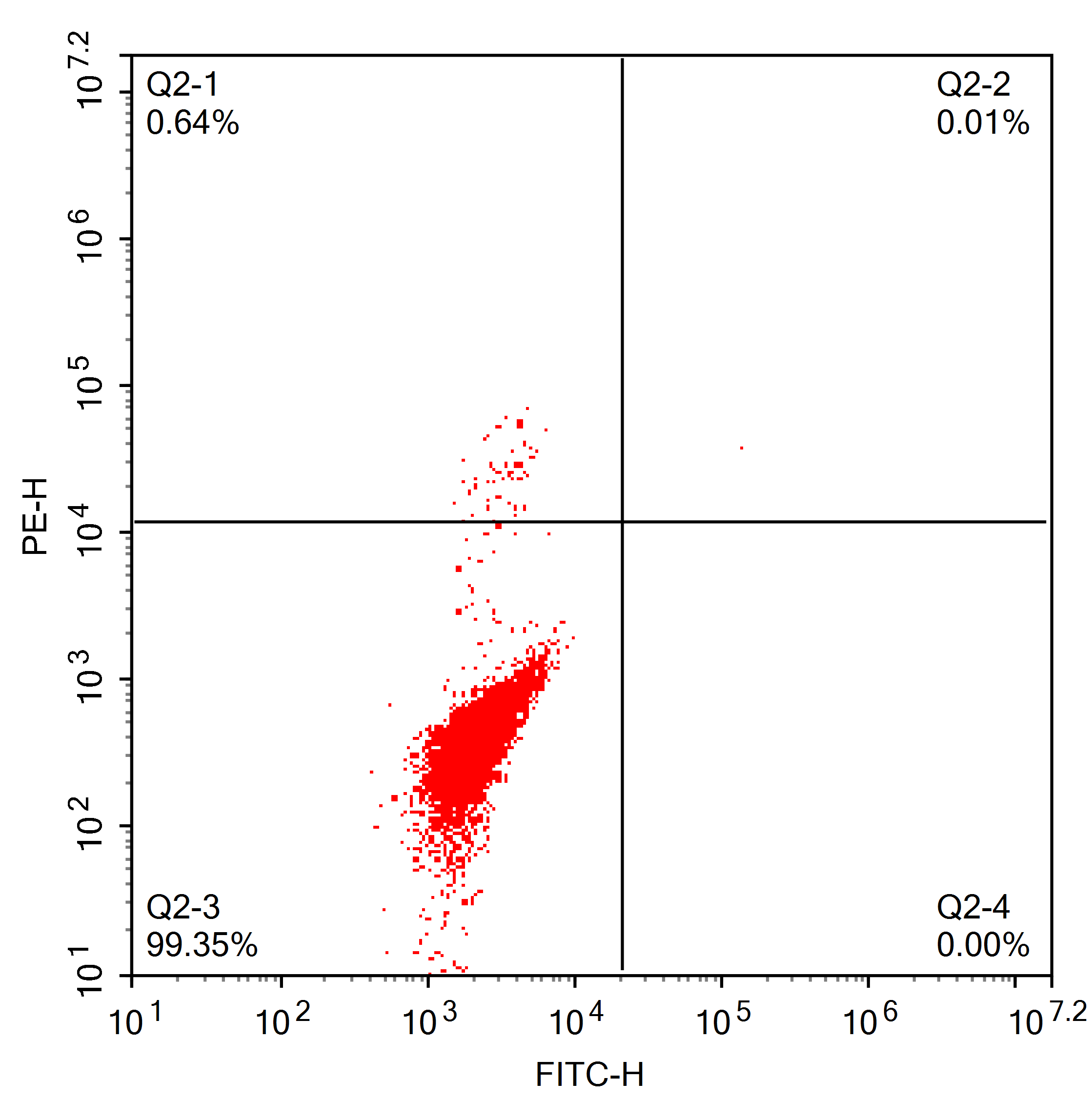

Supplement: Supplementary file 1 [file DataSheet3.zip › Flow Cytometry Assay(1,2)/Flow Cytometry Assay-1/╧╕░√╡≥═÷-1/╡≥═÷ 3/═╝╞1⁄4/PI/═╝2.tiff]

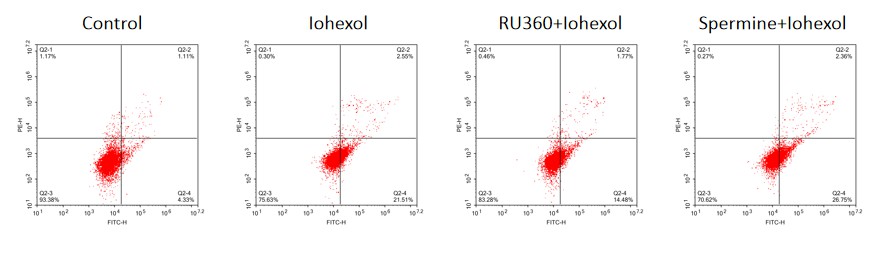

Supplement: Supplementary file 1 [file DataSheet3.zip › Flow Cytometry Assay(1,2)/Flow Cytometry Assay-2/╧╕░√╡≥═÷-2/HK-2 ╡≥═÷ 1/═╝╞1⁄4/1.jpg]

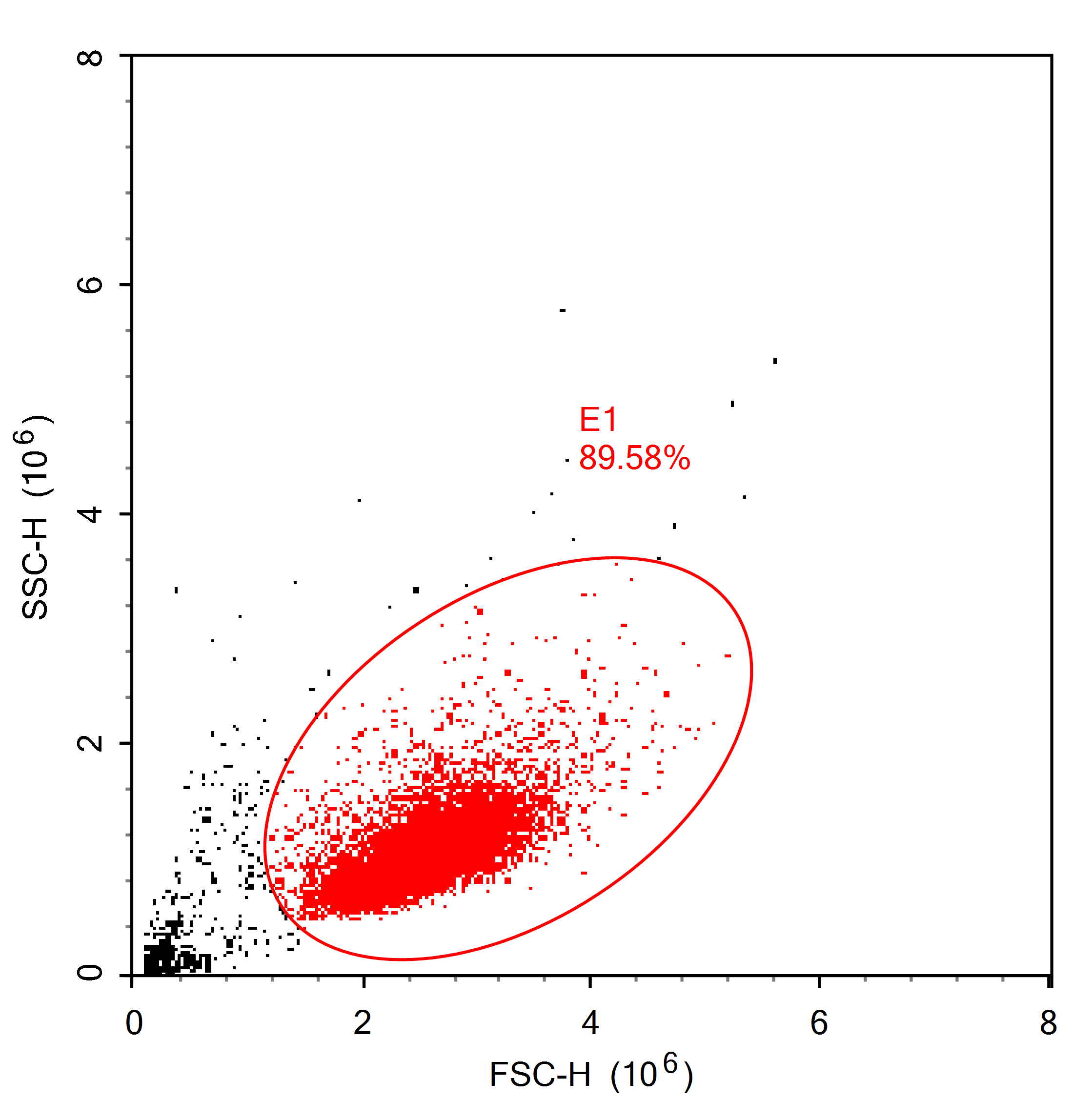

Supplement: Supplementary file 1 [file DataSheet3.zip › Flow Cytometry Assay(1,2)/Flow Cytometry Assay-2/╧╕░√╡≥═÷-2/HK-2 ╡≥═÷ 1/═╝╞1⁄4/Blank/═╝1.tiff]

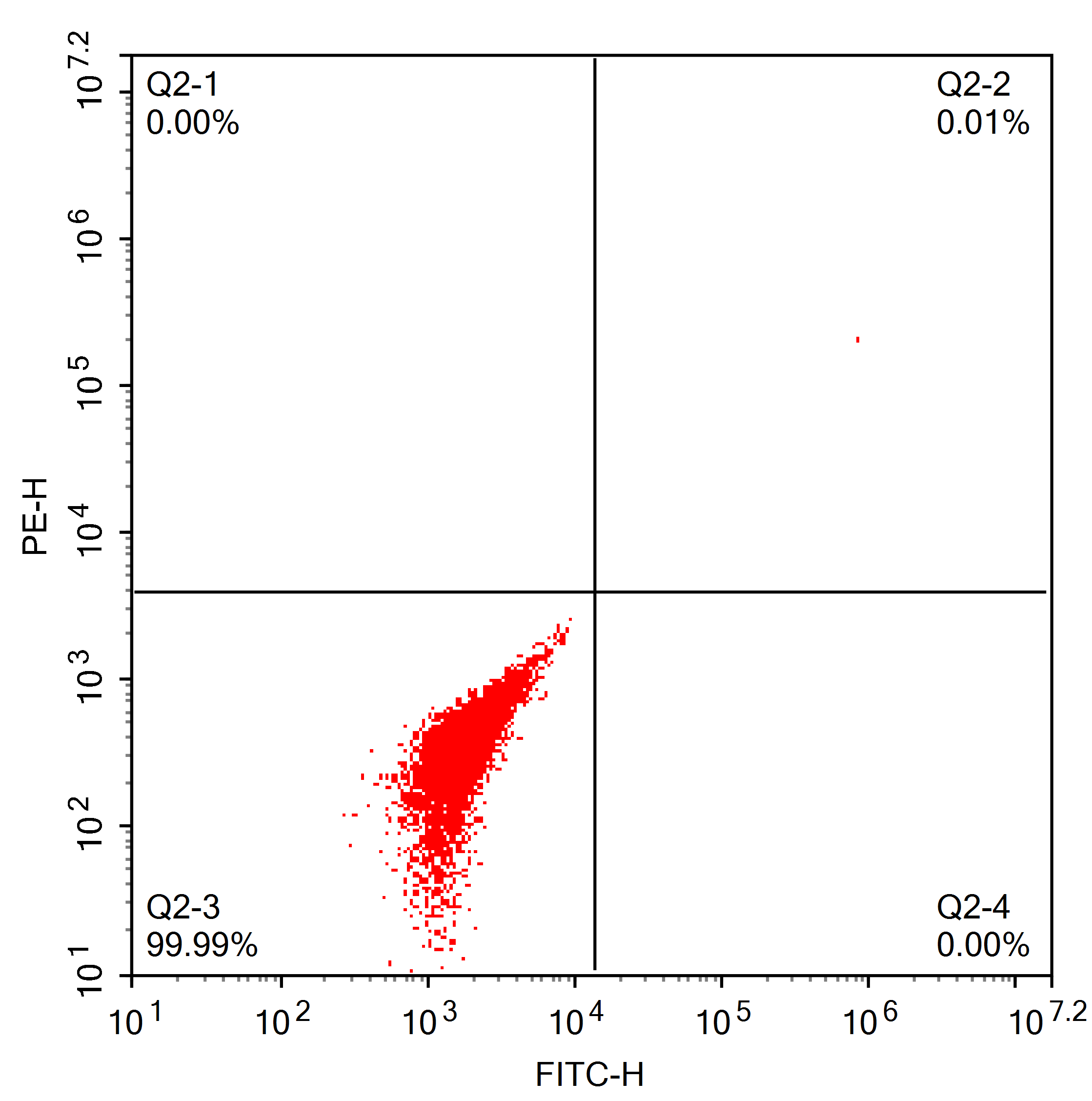

Supplement: Supplementary file 1 [file DataSheet3.zip › Flow Cytometry Assay(1,2)/Flow Cytometry Assay-2/╧╕░√╡≥═÷-2/HK-2 ╡≥═÷ 1/═╝╞1⁄4/Blank/═╝2.tiff]

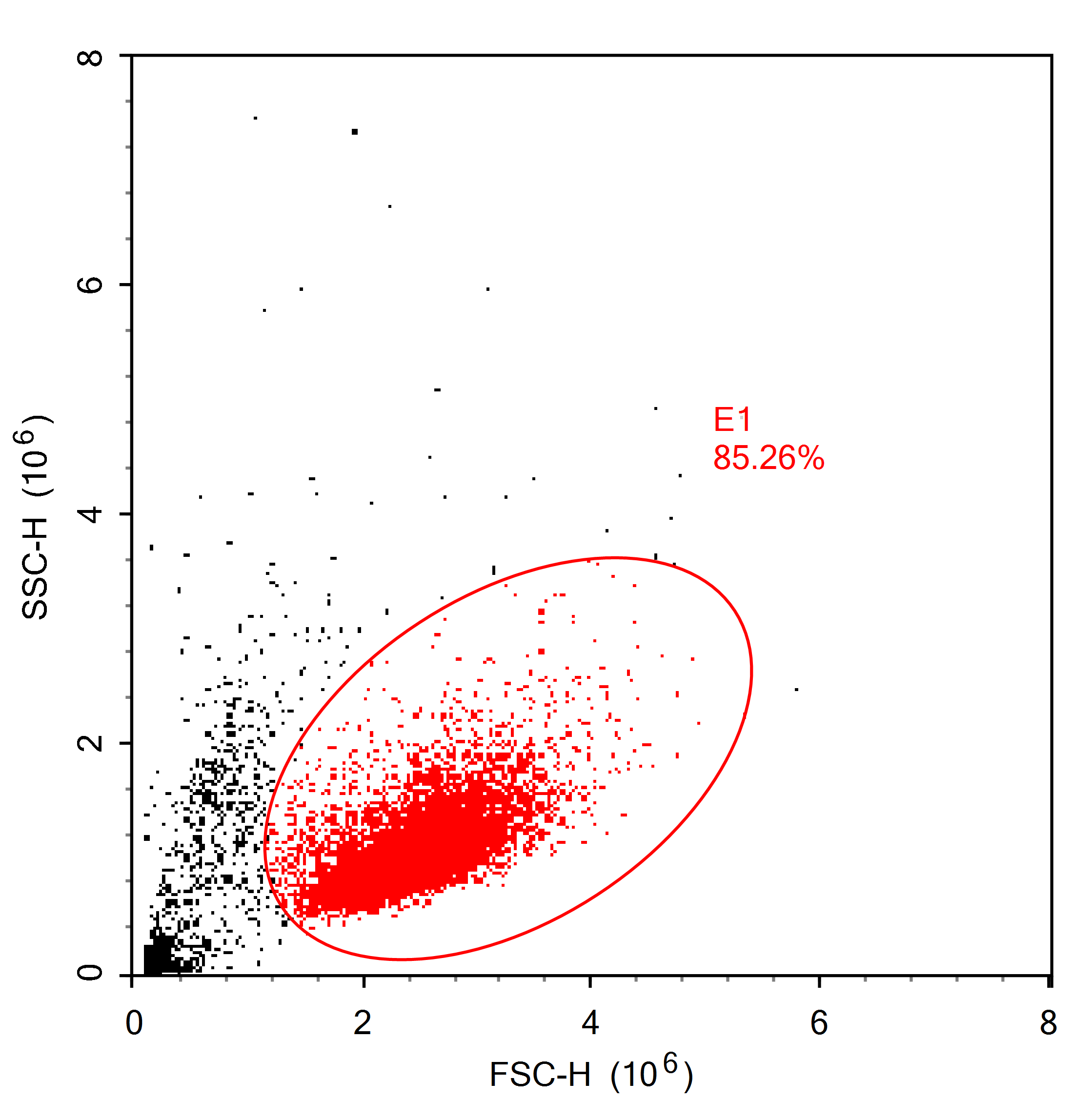

Supplement: Supplementary file 1 [file DataSheet3.zip › Flow Cytometry Assay(1,2)/Flow Cytometry Assay-2/╧╕░√╡≥═÷-2/HK-2 ╡≥═÷ 1/═╝╞1⁄4/Ctrl 1/═╝1.tiff]

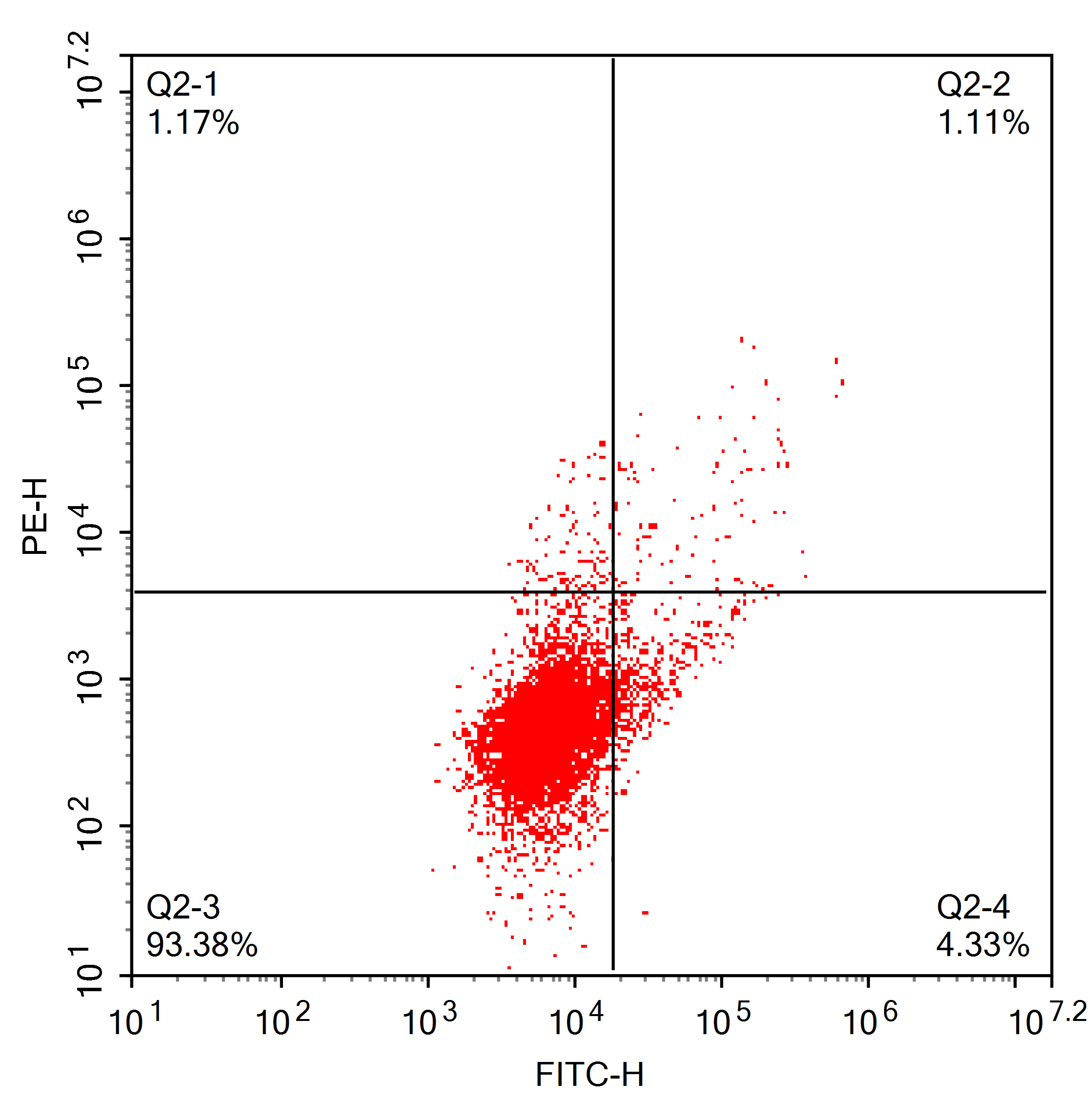

Supplement: Supplementary file 1 [file DataSheet3.zip › Flow Cytometry Assay(1,2)/Flow Cytometry Assay-2/╧╕░√╡≥═÷-2/HK-2 ╡≥═÷ 1/═╝╞1⁄4/Ctrl 1/═╝2.tiff]

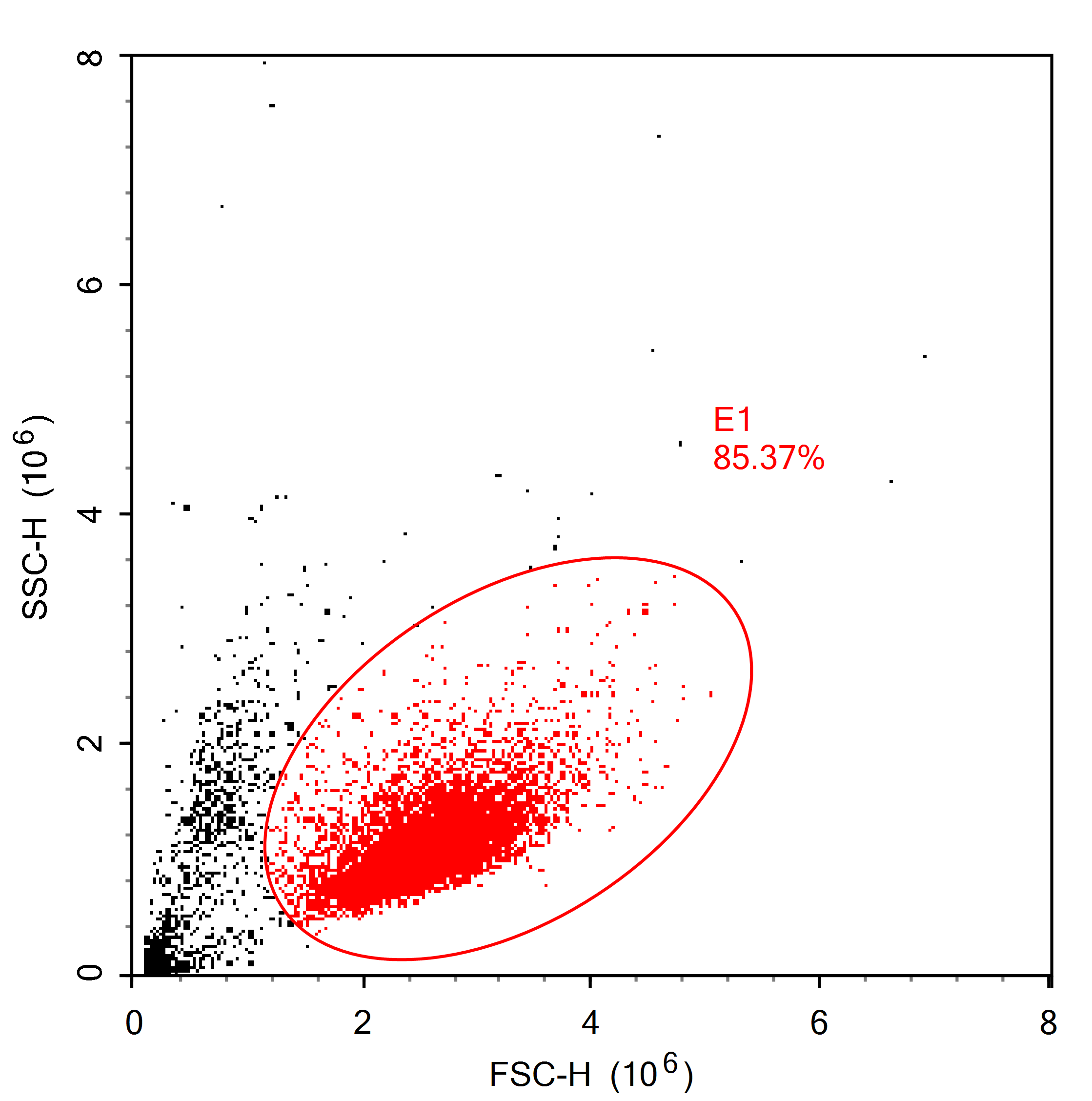

Supplement: Supplementary file 1 [file DataSheet3.zip › Flow Cytometry Assay(1,2)/Flow Cytometry Assay-2/╧╕░√╡≥═÷-2/HK-2 ╡≥═÷ 1/═╝╞1⁄4/Ctrl 2/═╝1.tiff]

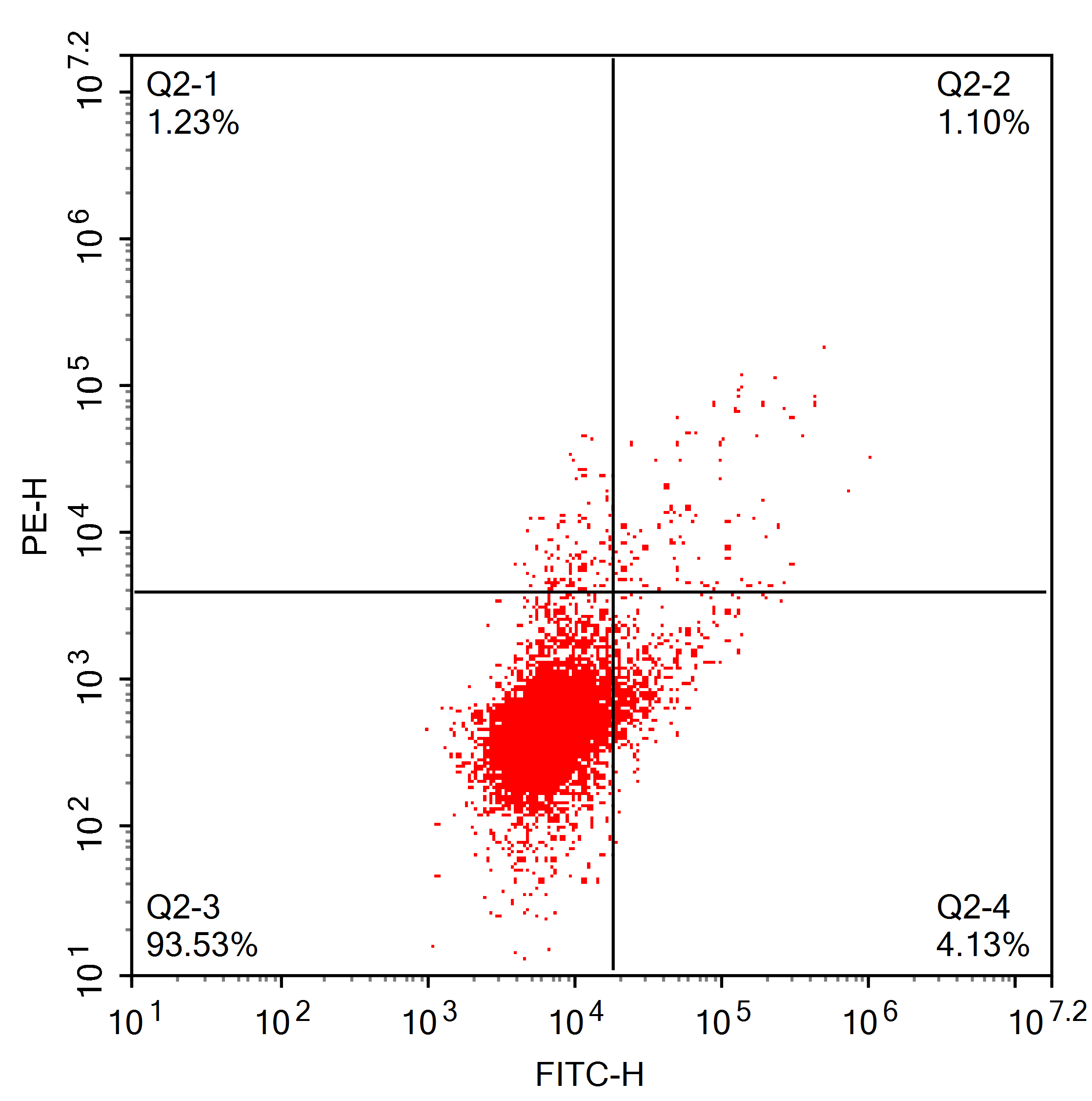

Supplement: Supplementary file 1 [file DataSheet3.zip › Flow Cytometry Assay(1,2)/Flow Cytometry Assay-2/╧╕░√╡≥═÷-2/HK-2 ╡≥═÷ 1/═╝╞1⁄4/Ctrl 2/═╝2.tiff]

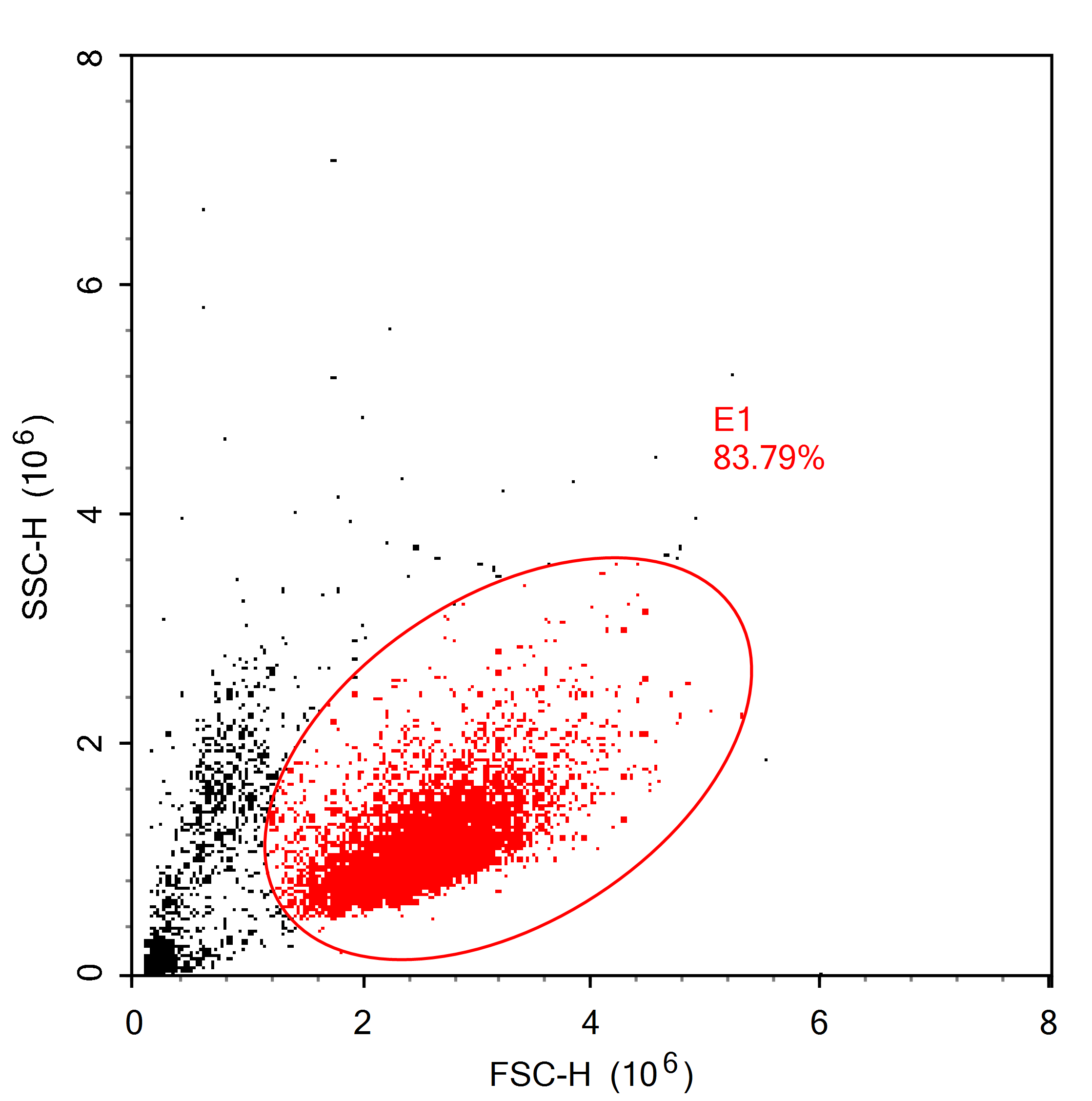

Supplement: Supplementary file 1 [file DataSheet3.zip › Flow Cytometry Assay(1,2)/Flow Cytometry Assay-2/╧╕░√╡≥═÷-2/HK-2 ╡≥═÷ 1/═╝╞1⁄4/Ctrl 3/═╝1.tiff]

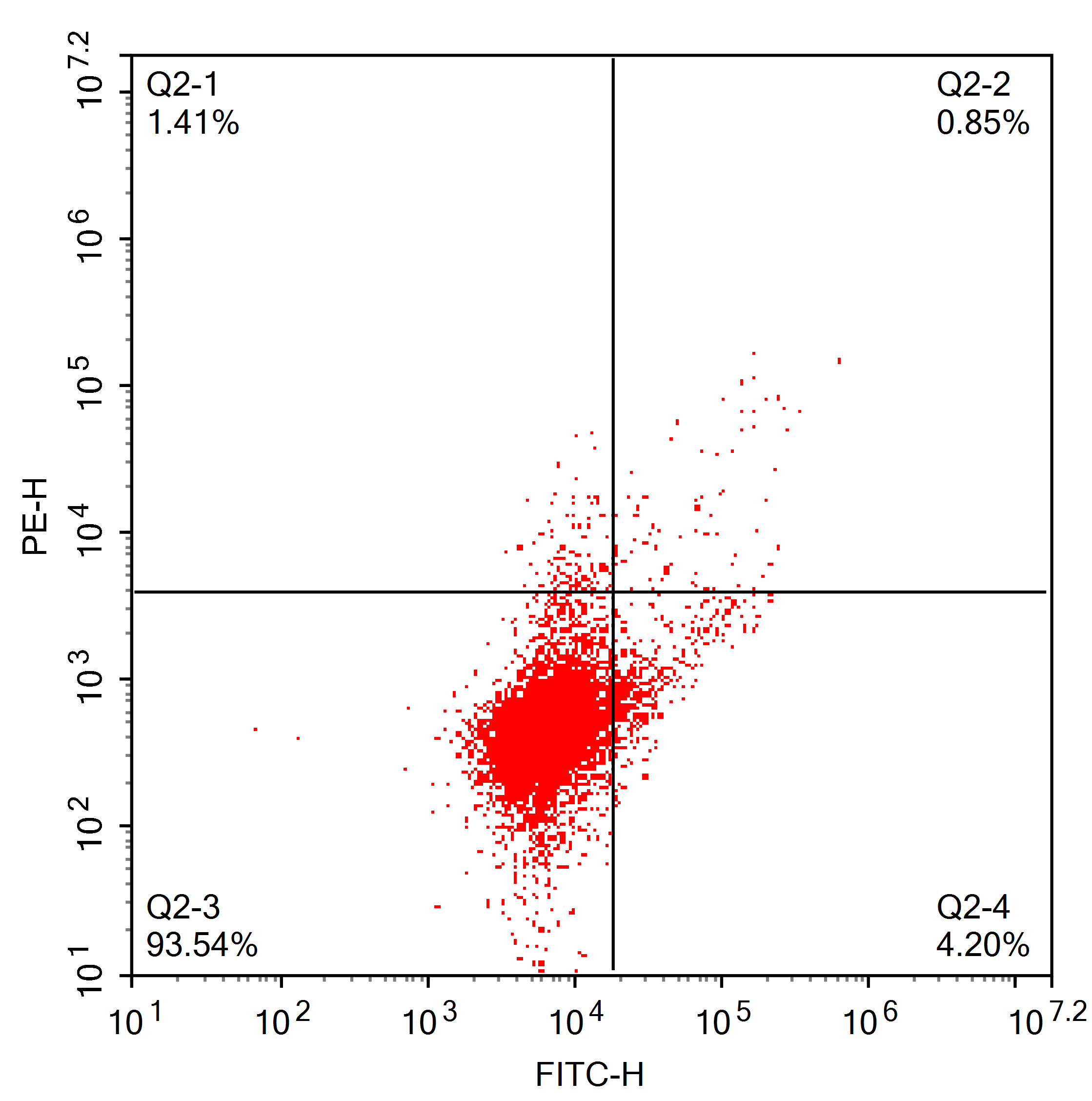

Supplement: Supplementary file 1 [file DataSheet3.zip › Flow Cytometry Assay(1,2)/Flow Cytometry Assay-2/╧╕░√╡≥═÷-2/HK-2 ╡≥═÷ 1/═╝╞1⁄4/Ctrl 3/═╝2.tiff]
