# Supplementary figures and images for: Mitochondrial calcium uniporter-mediated mitochondrial dynamics imbalance contributes to contrast medium-induced renal tubular cell injury (part 3 of 4)
Source: Front Mol Biosci. 2026 Jun 29;13:1848361. doi: 10.3389/fmolb.2026.1848361 (PMC13357276; doi:10.3389/fmolb.2026.1848361)

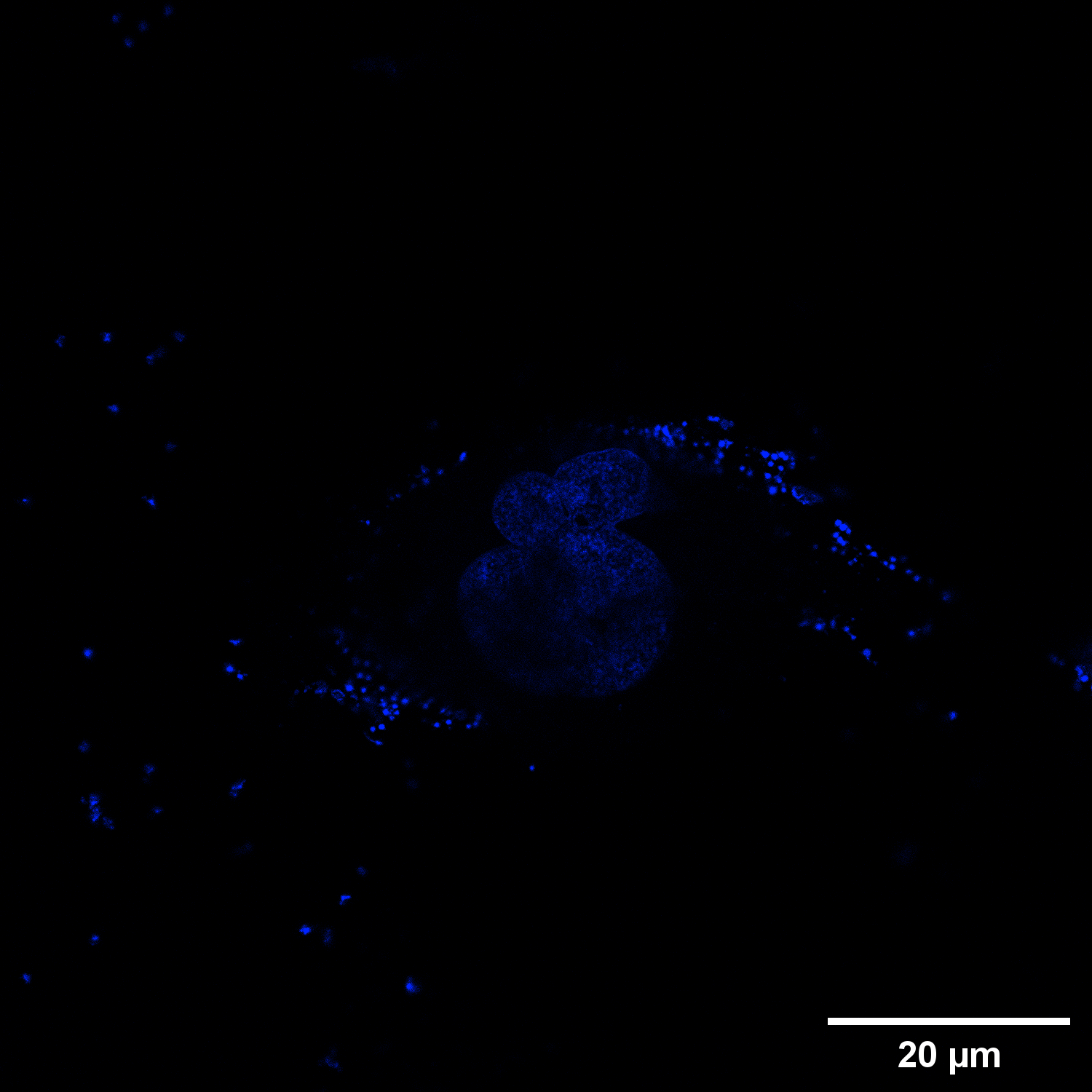

Supplement: Supplementary file 3 [file DataSheet4.zip › Mitotracker(1,2)/Mitotracker-2/Mitotracker-2═╝╞1⁄4/Control/Con-2/2_RGB_SR405.tif]

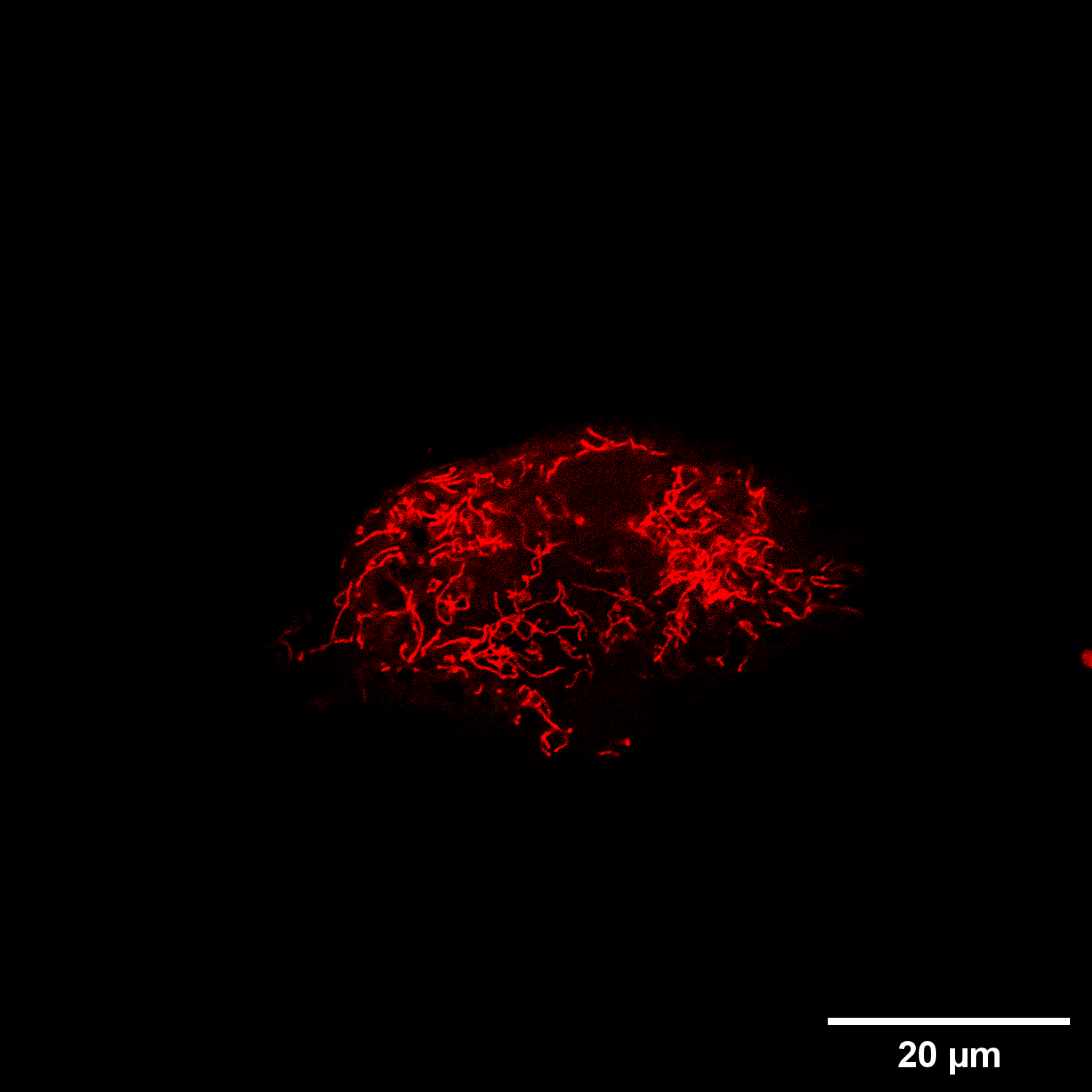

Supplement: Supplementary file 3 [file DataSheet4.zip › Mitotracker(1,2)/Mitotracker-2/Mitotracker-2═╝╞1⁄4/Control/Con-2/2_RGB_SR561.tif]

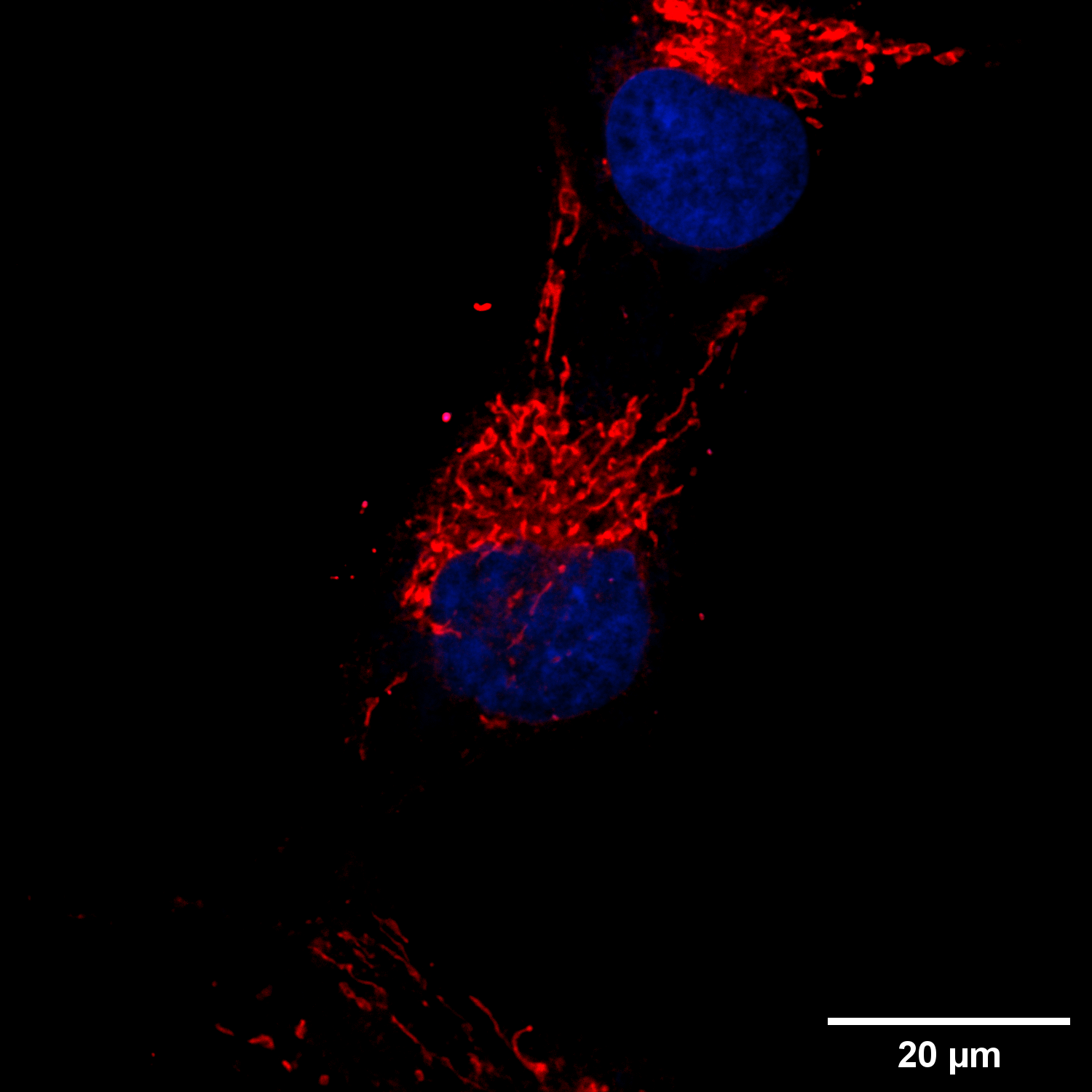

Supplement: Supplementary file 3 [file DataSheet4.zip › Mitotracker(1,2)/Mitotracker-2/Mitotracker-2═╝╞1⁄4/Control/Con-3/3_RGB.tif]

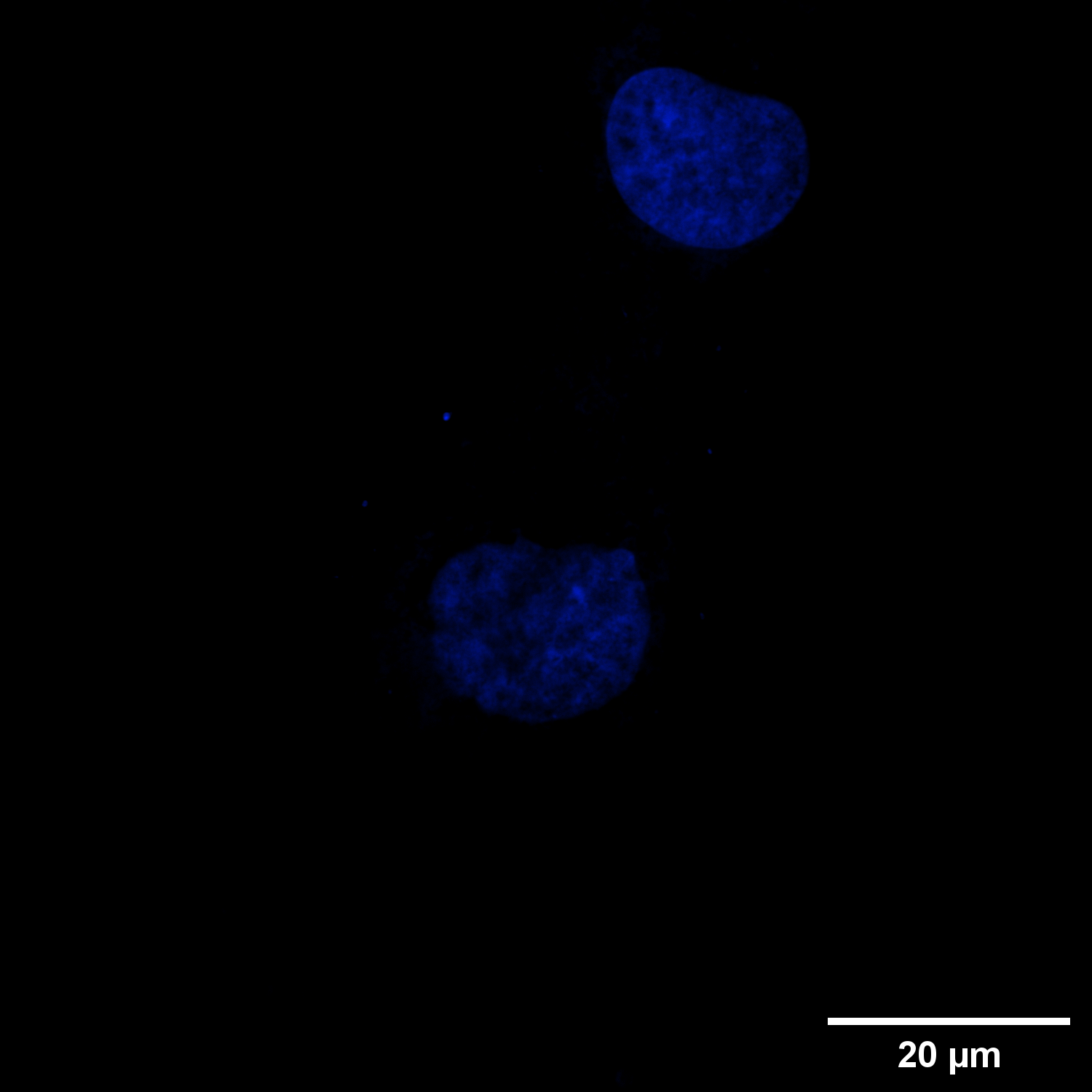

Supplement: Supplementary file 3 [file DataSheet4.zip › Mitotracker(1,2)/Mitotracker-2/Mitotracker-2═╝╞1⁄4/Control/Con-3/3_RGB_SR405.tif]

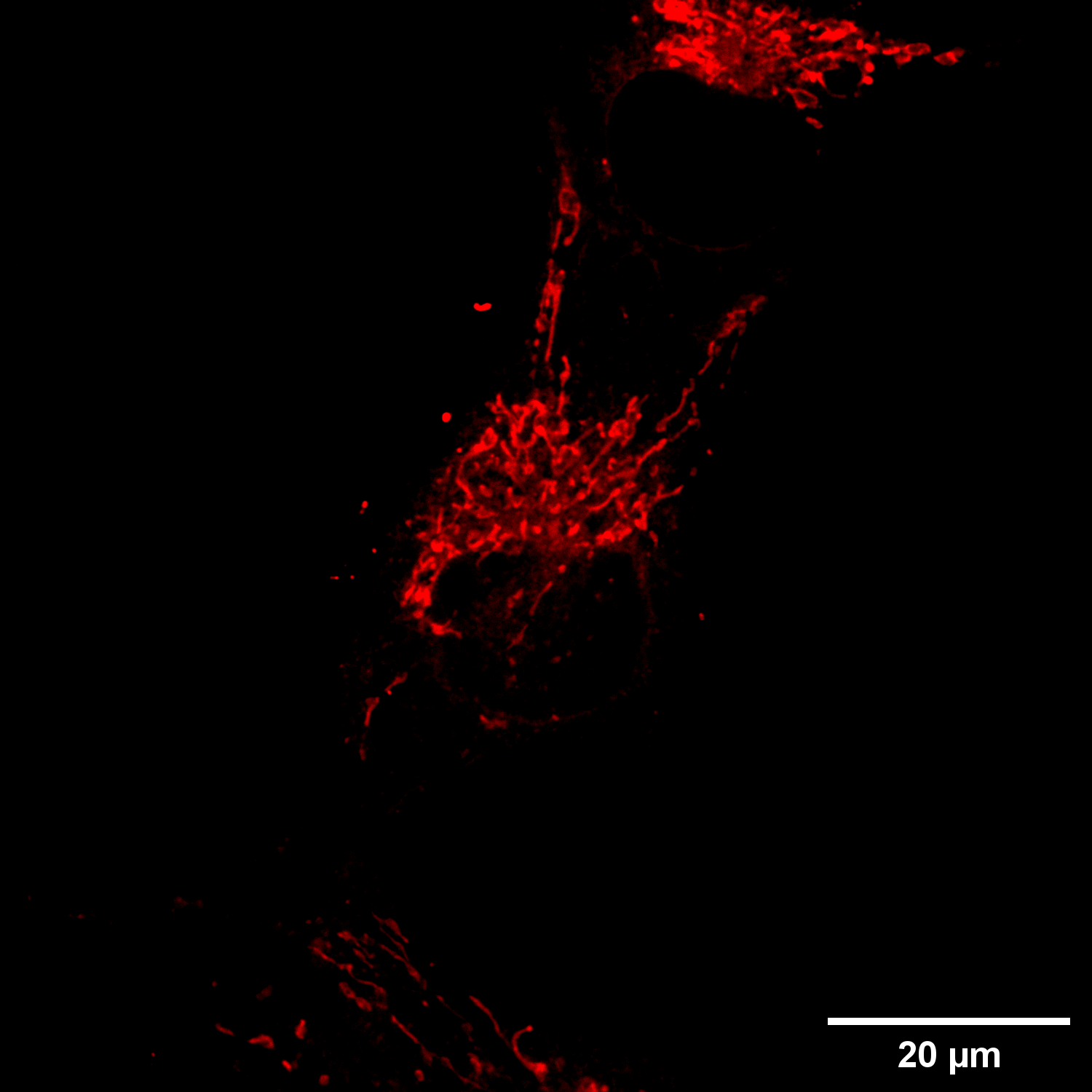

Supplement: Supplementary file 3 [file DataSheet4.zip › Mitotracker(1,2)/Mitotracker-2/Mitotracker-2═╝╞1⁄4/Control/Con-3/3_RGB_SR561.tif]

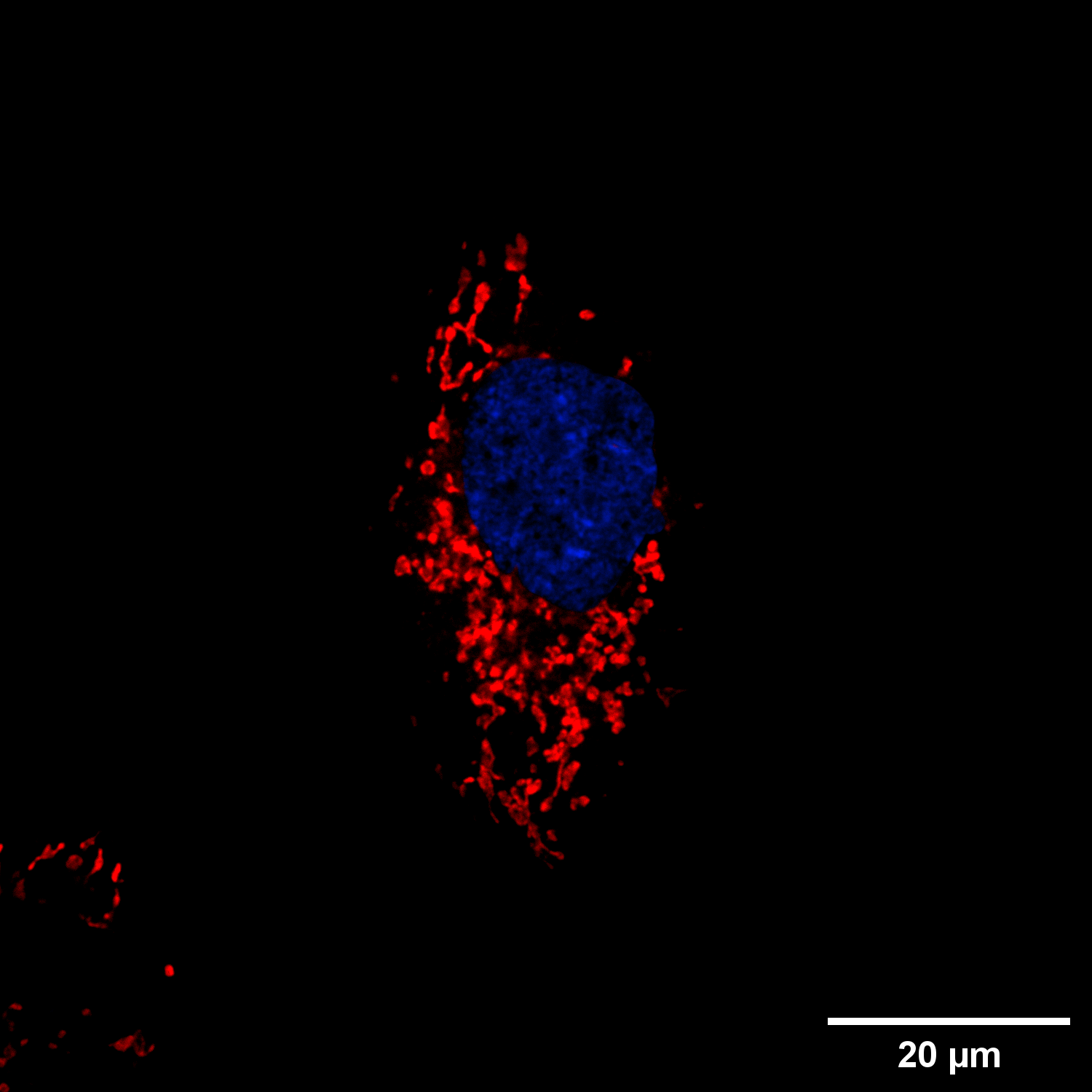

Supplement: Supplementary file 3 [file DataSheet4.zip › Mitotracker(1,2)/Mitotracker-2/Mitotracker-2═╝╞1⁄4/Iohexol/Ioh-1/1_RGB.tif]

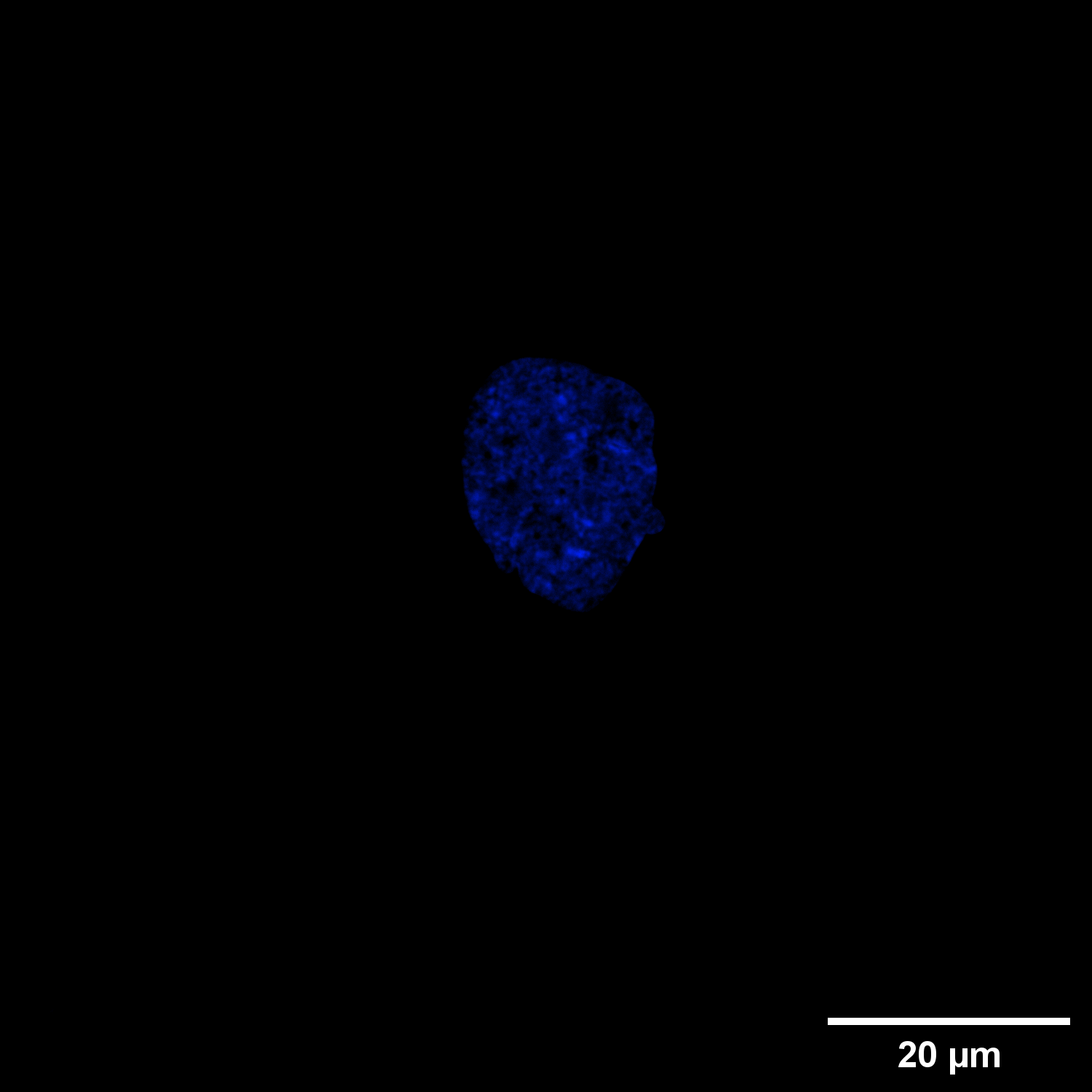

Supplement: Supplementary file 3 [file DataSheet4.zip › Mitotracker(1,2)/Mitotracker-2/Mitotracker-2═╝╞1⁄4/Iohexol/Ioh-1/1_RGB_SR405.tif]

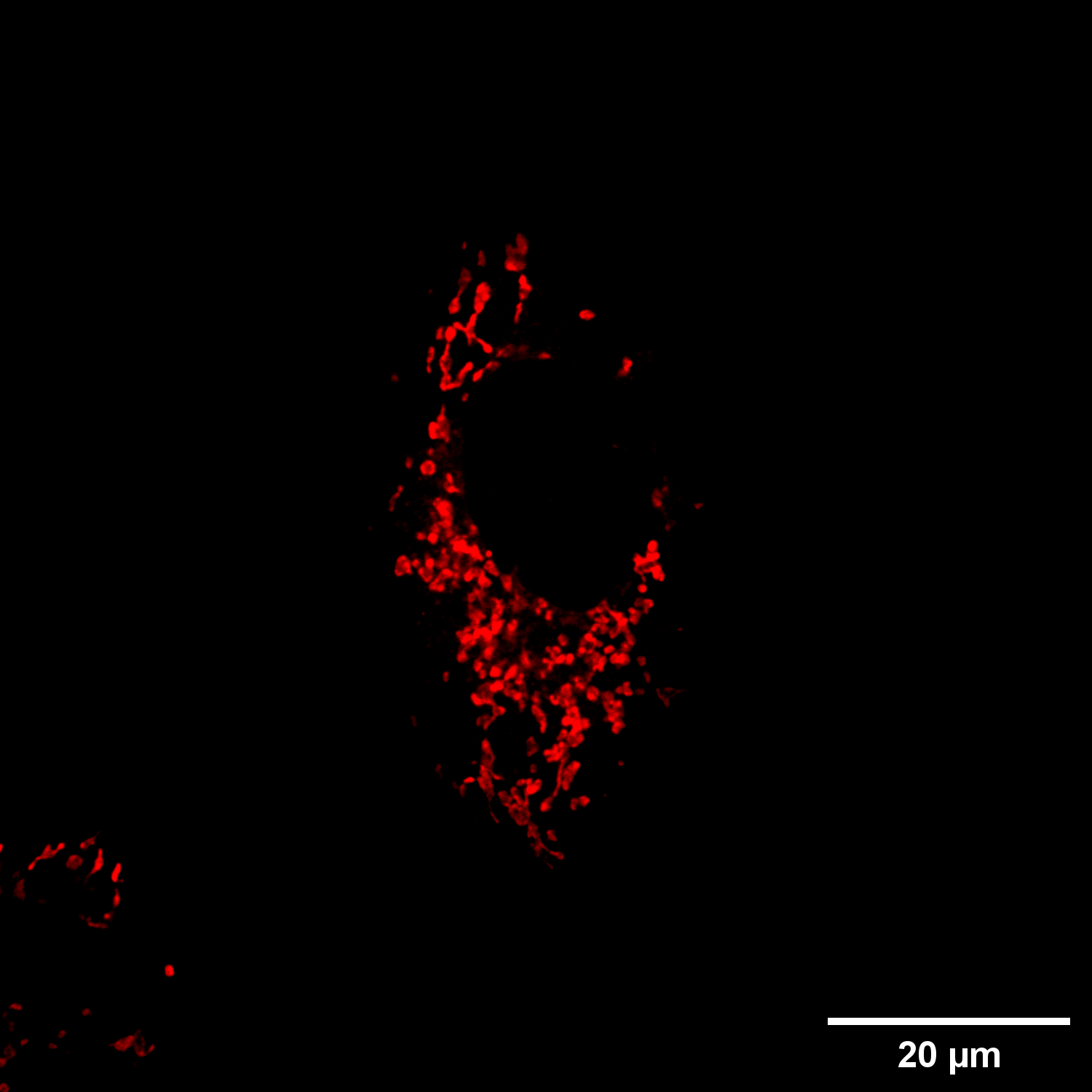

Supplement: Supplementary file 3 [file DataSheet4.zip › Mitotracker(1,2)/Mitotracker-2/Mitotracker-2═╝╞1⁄4/Iohexol/Ioh-1/1_RGB_SR561.tif]

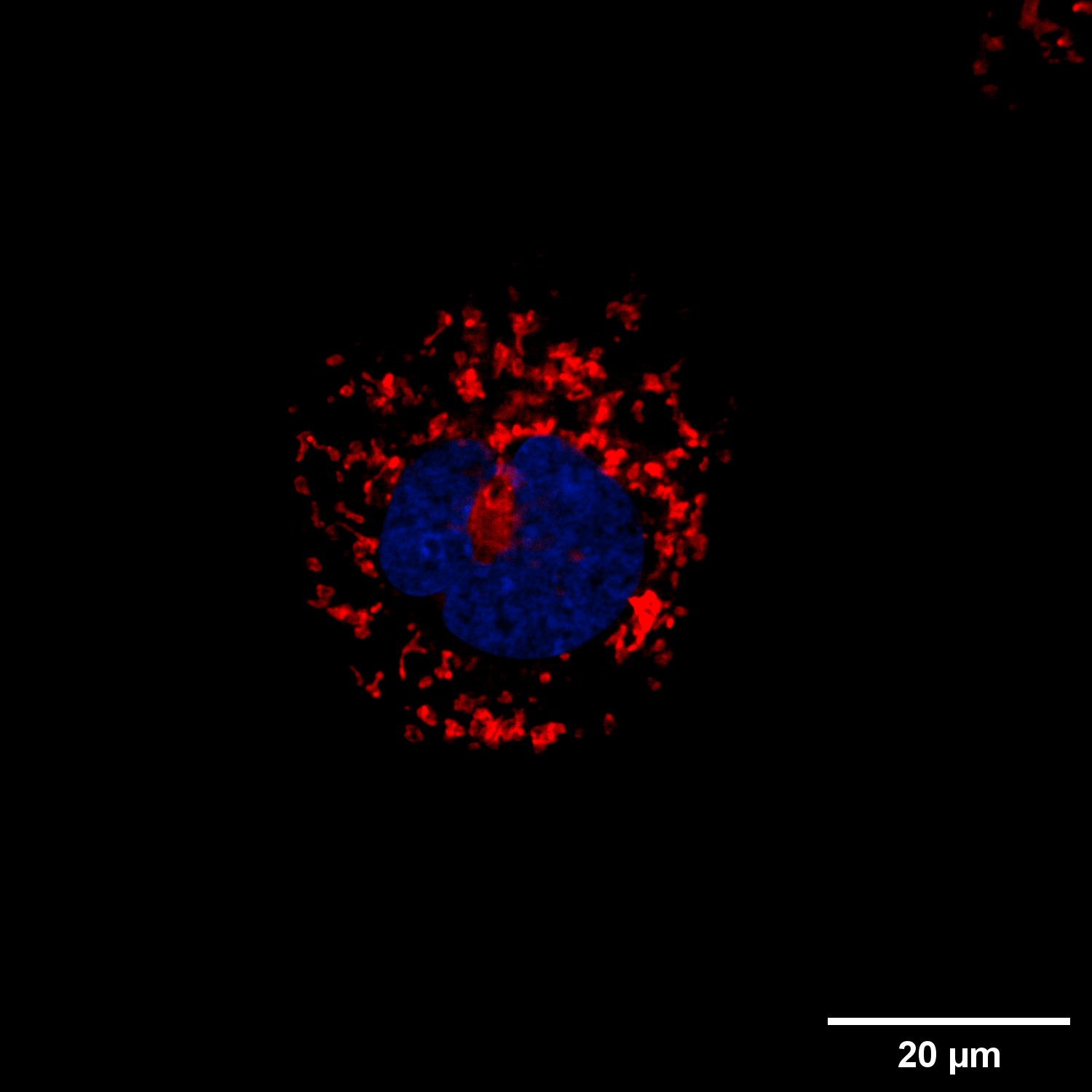

Supplement: Supplementary file 3 [file DataSheet4.zip › Mitotracker(1,2)/Mitotracker-2/Mitotracker-2═╝╞1⁄4/Iohexol/Ioh-2/2_RGB.tif]

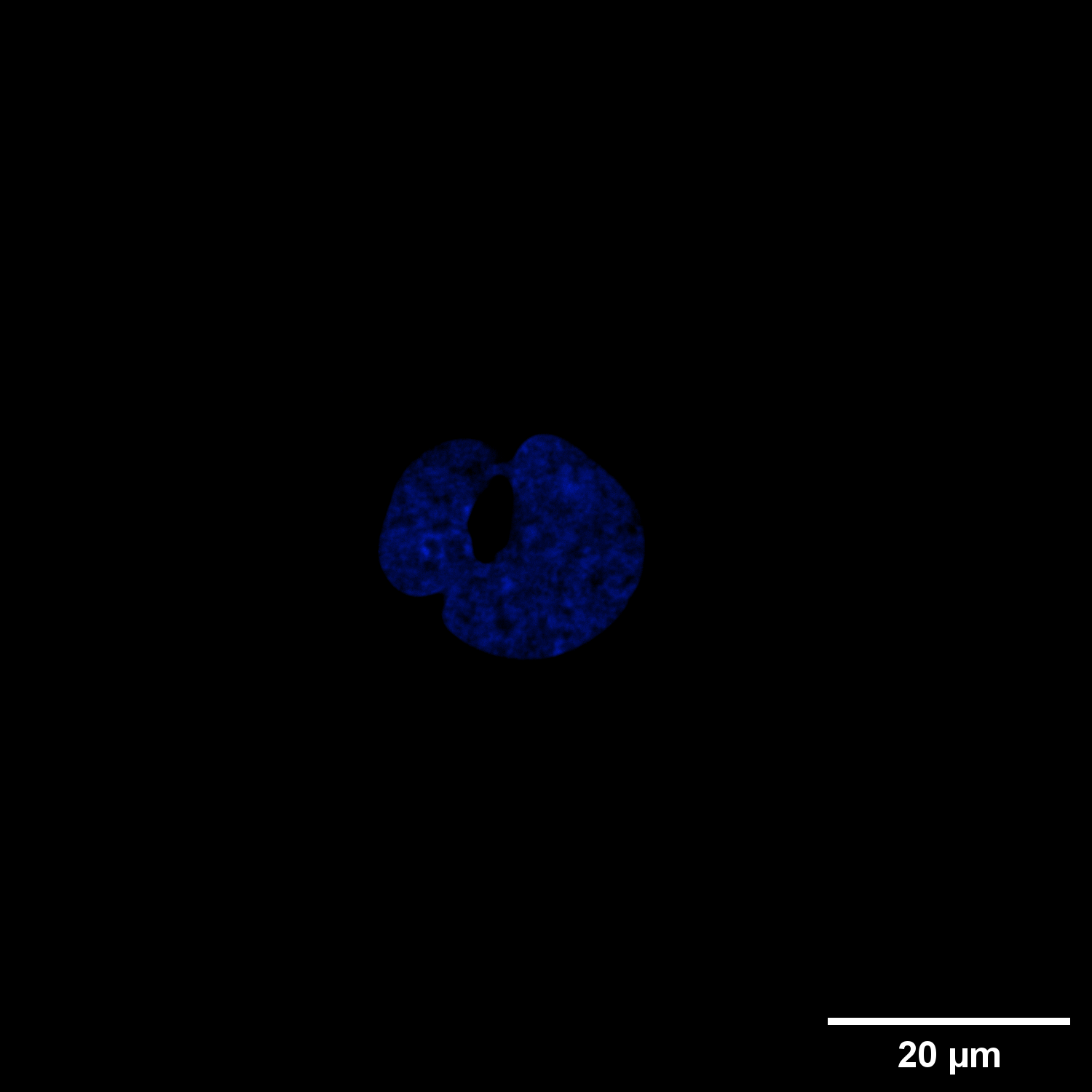

Supplement: Supplementary file 3 [file DataSheet4.zip › Mitotracker(1,2)/Mitotracker-2/Mitotracker-2═╝╞1⁄4/Iohexol/Ioh-2/2_RGB_SR405.tif]

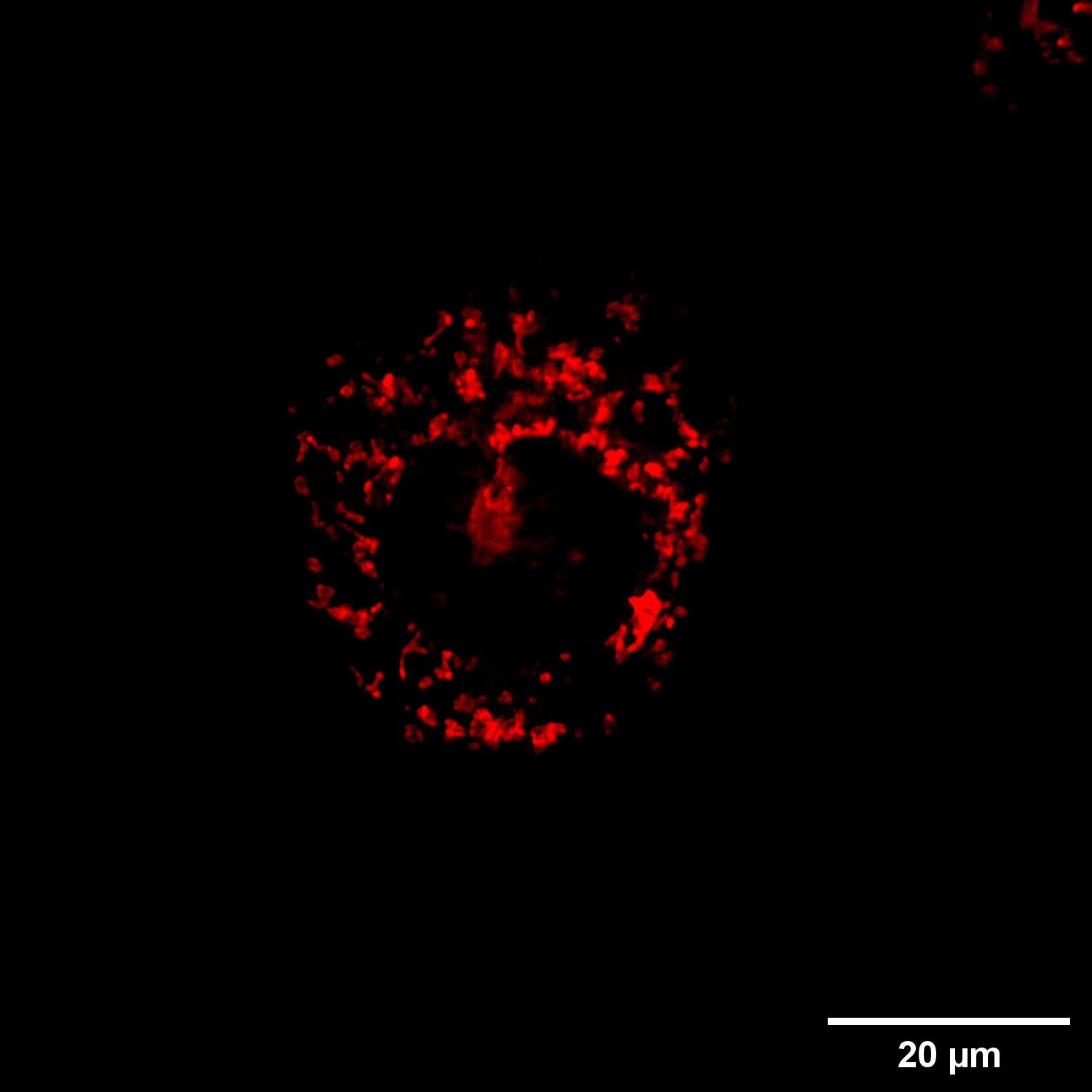

Supplement: Supplementary file 3 [file DataSheet4.zip › Mitotracker(1,2)/Mitotracker-2/Mitotracker-2═╝╞1⁄4/Iohexol/Ioh-2/2_RGB_SR561.tif]

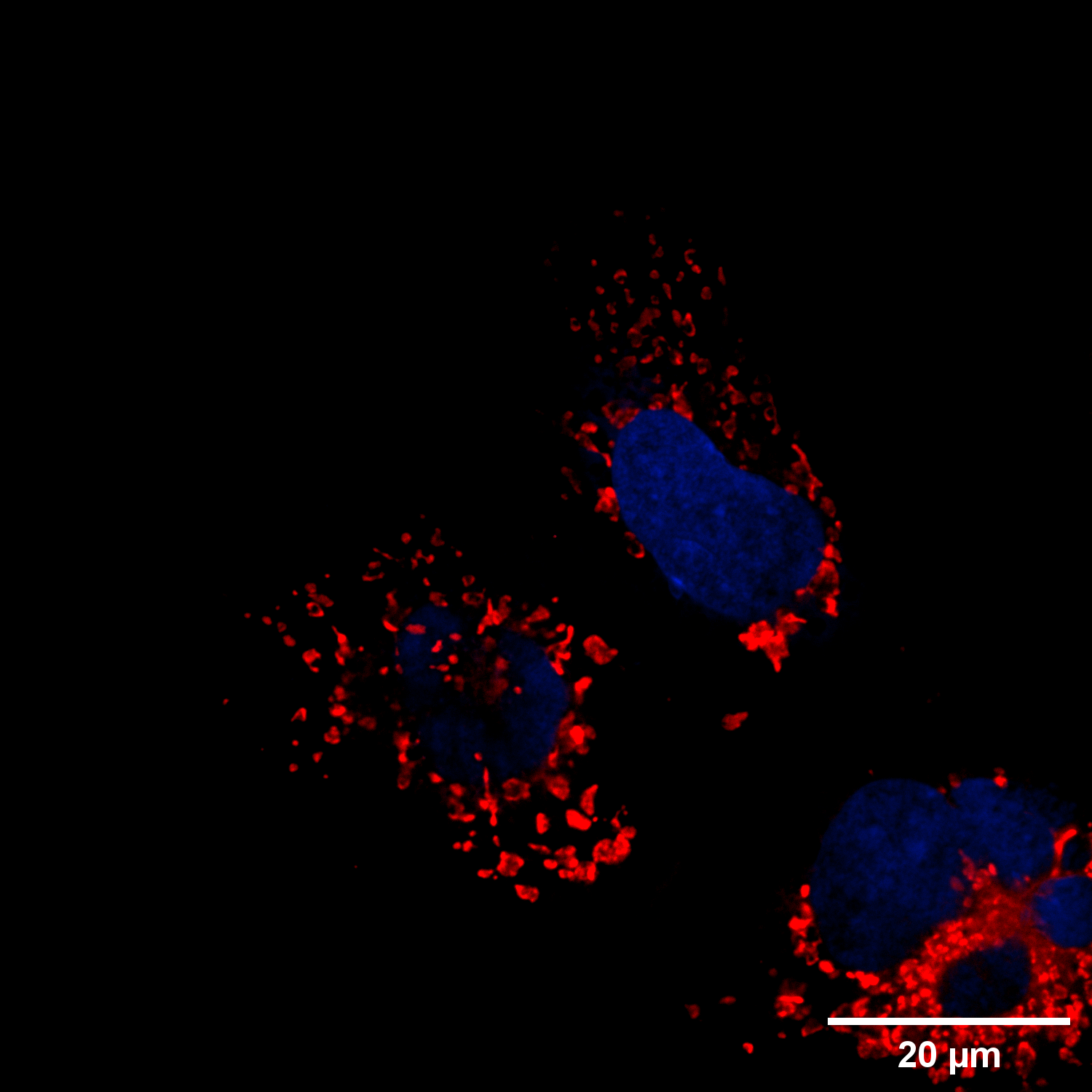

Supplement: Supplementary file 3 [file DataSheet4.zip › Mitotracker(1,2)/Mitotracker-2/Mitotracker-2═╝╞1⁄4/Iohexol/Ioh-3/3_RGB.tif]

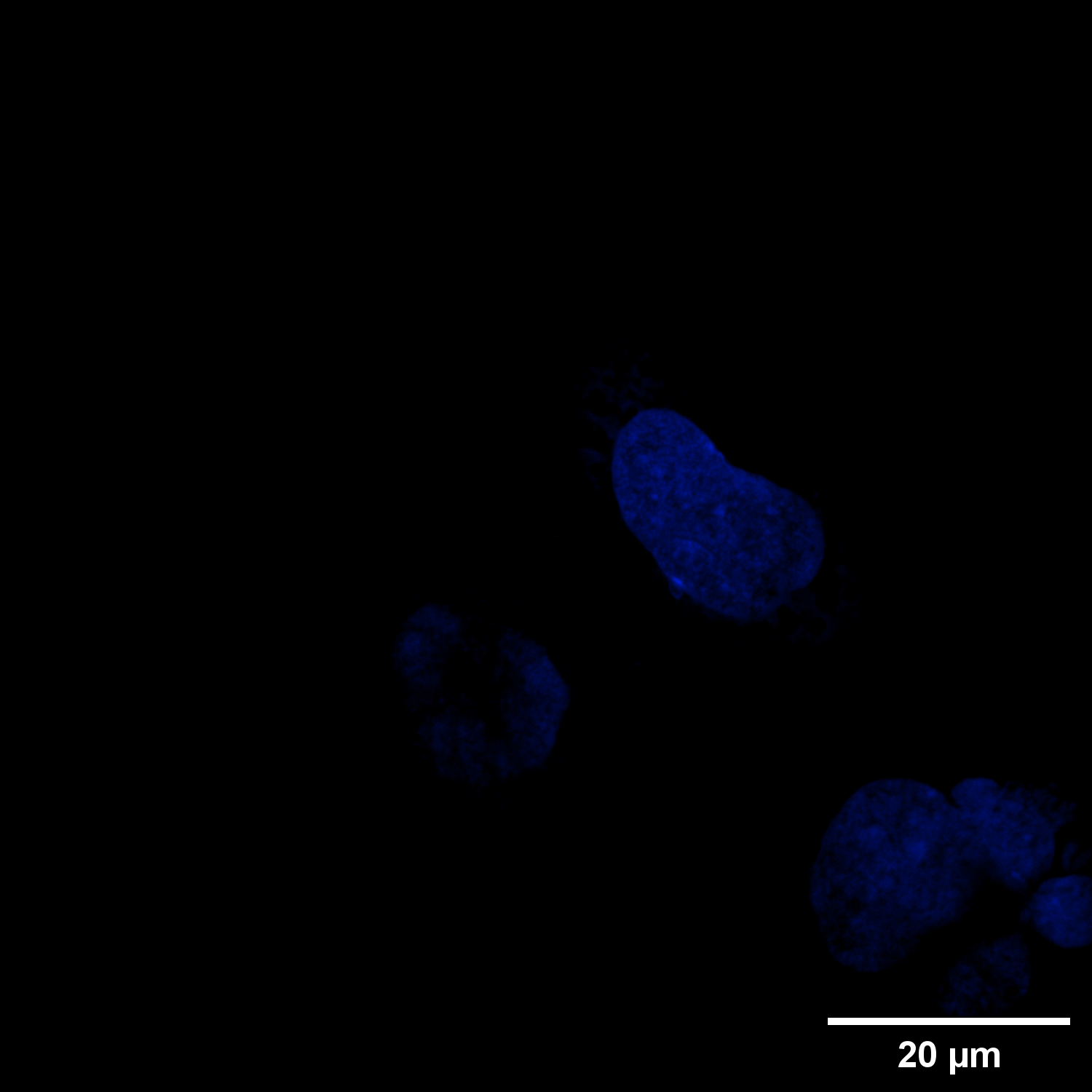

Supplement: Supplementary file 3 [file DataSheet4.zip › Mitotracker(1,2)/Mitotracker-2/Mitotracker-2═╝╞1⁄4/Iohexol/Ioh-3/3_RGB_SR405.tif]

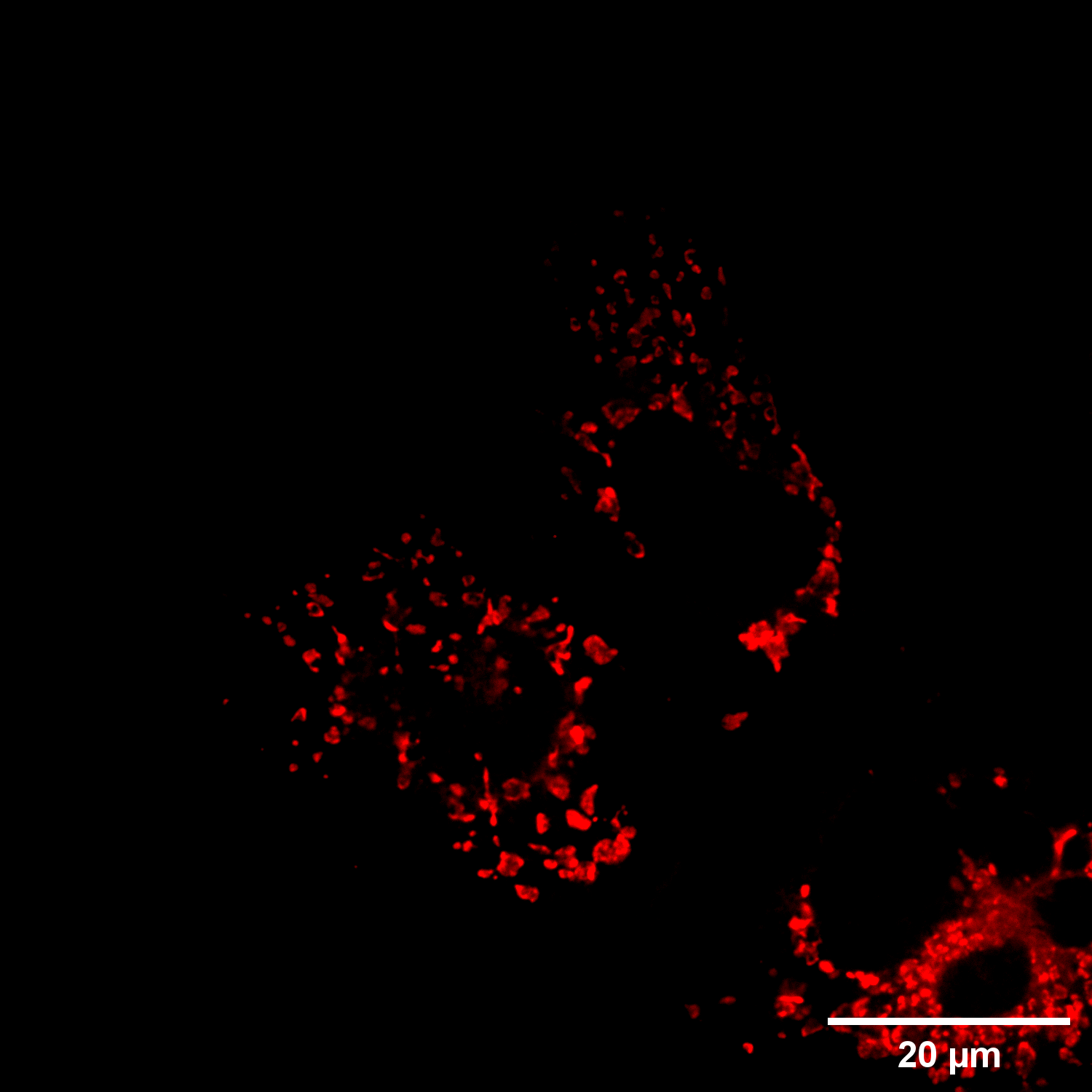

Supplement: Supplementary file 3 [file DataSheet4.zip › Mitotracker(1,2)/Mitotracker-2/Mitotracker-2═╝╞1⁄4/Iohexol/Ioh-3/3_RGB_SR561.tif]

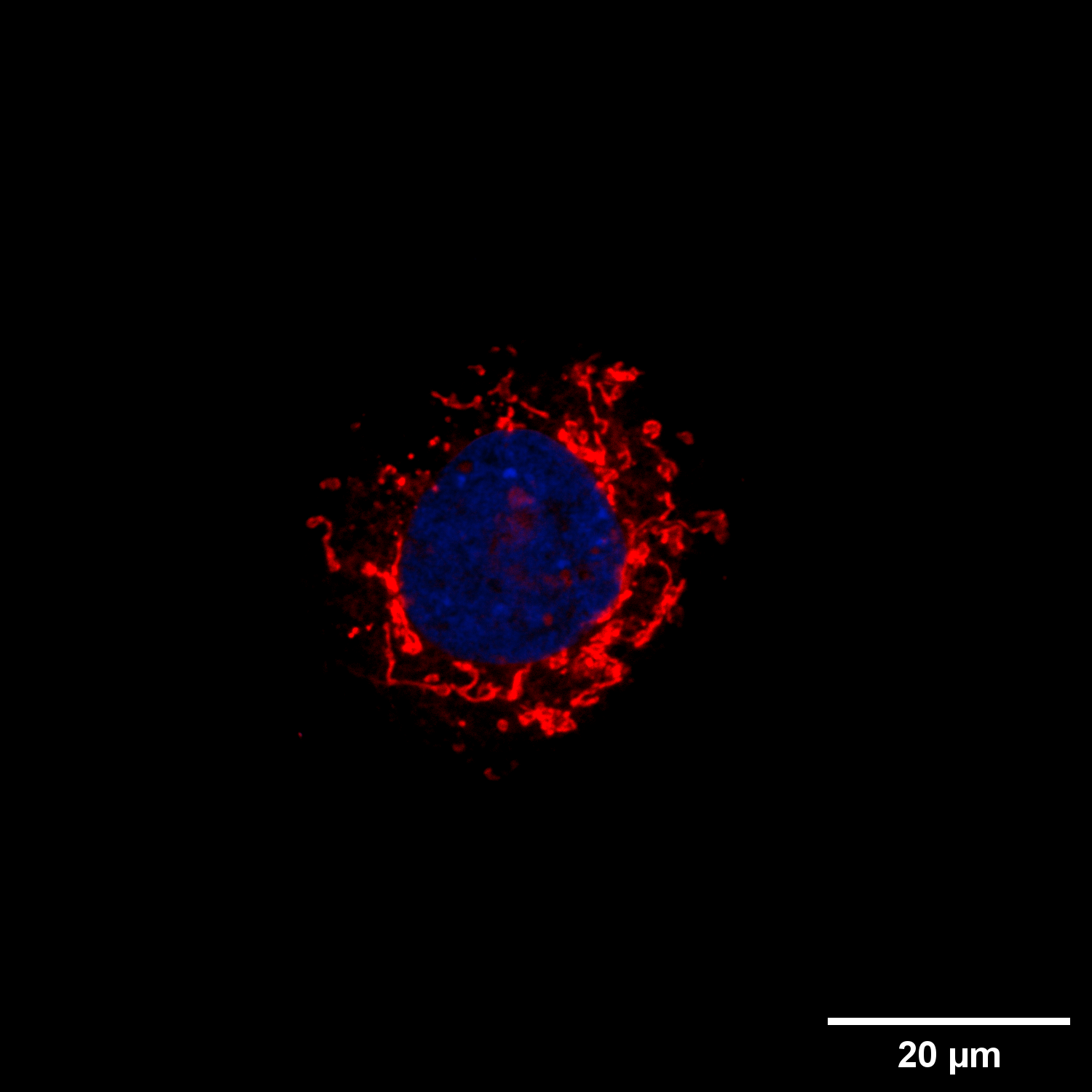

Supplement: Supplementary file 3 [file DataSheet4.zip › Mitotracker(1,2)/Mitotracker-2/Mitotracker-2═╝╞1⁄4/Iohexol+RU360/Ioh+RU360-1/1_RGB.tif]

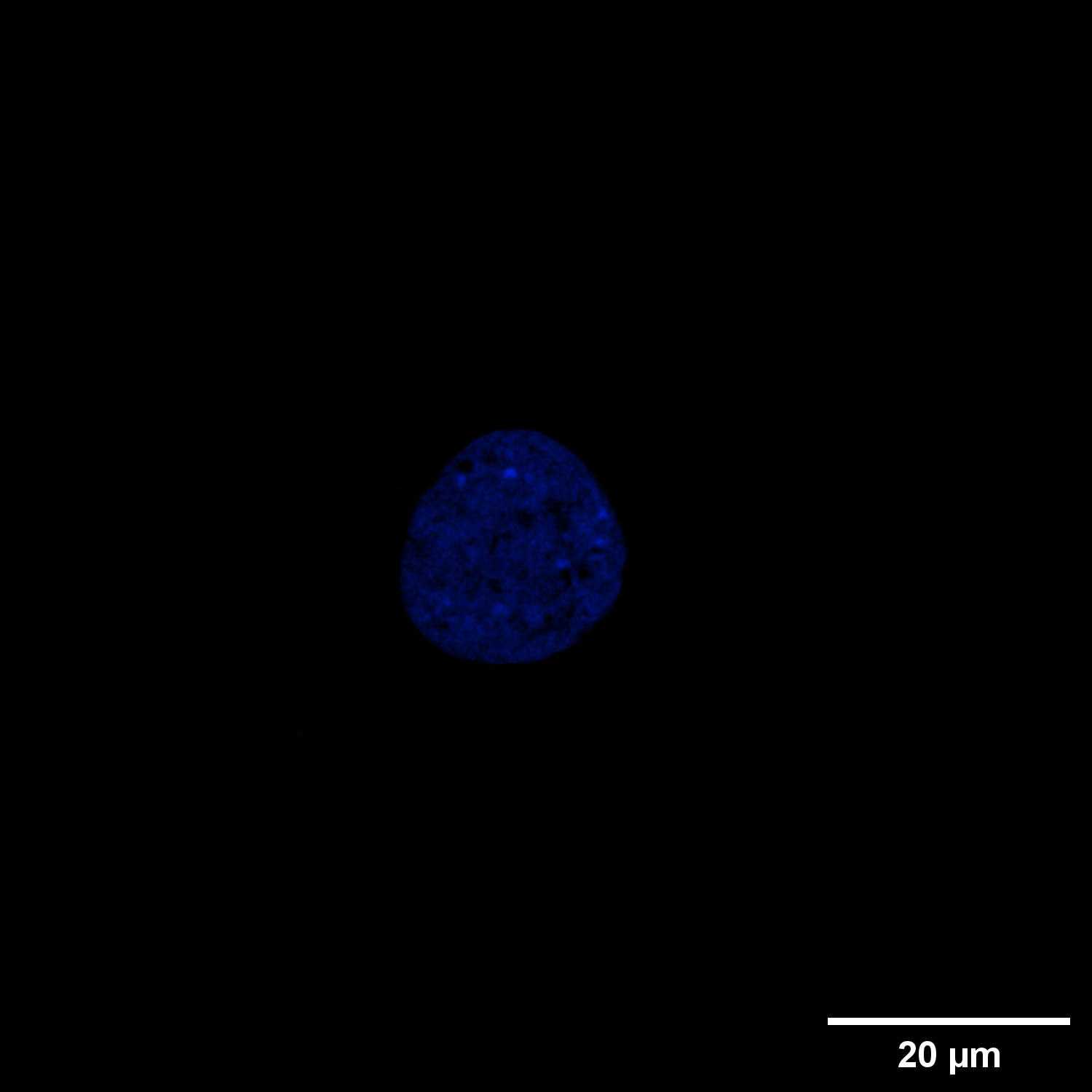

Supplement: Supplementary file 3 [file DataSheet4.zip › Mitotracker(1,2)/Mitotracker-2/Mitotracker-2═╝╞1⁄4/Iohexol+RU360/Ioh+RU360-1/1_RGB_SR405.tif]

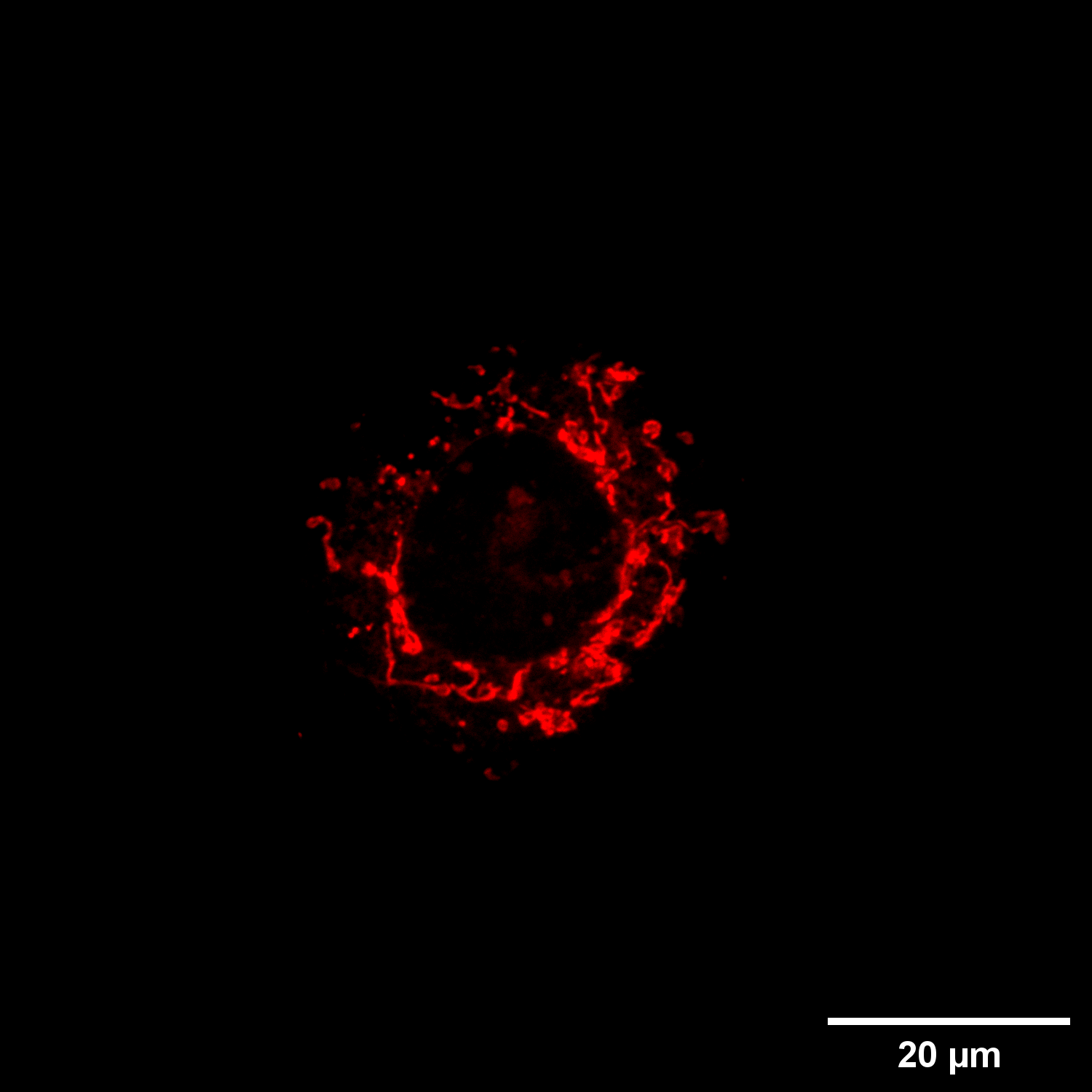

Supplement: Supplementary file 3 [file DataSheet4.zip › Mitotracker(1,2)/Mitotracker-2/Mitotracker-2═╝╞1⁄4/Iohexol+RU360/Ioh+RU360-1/1_RGB_SR561.tif]

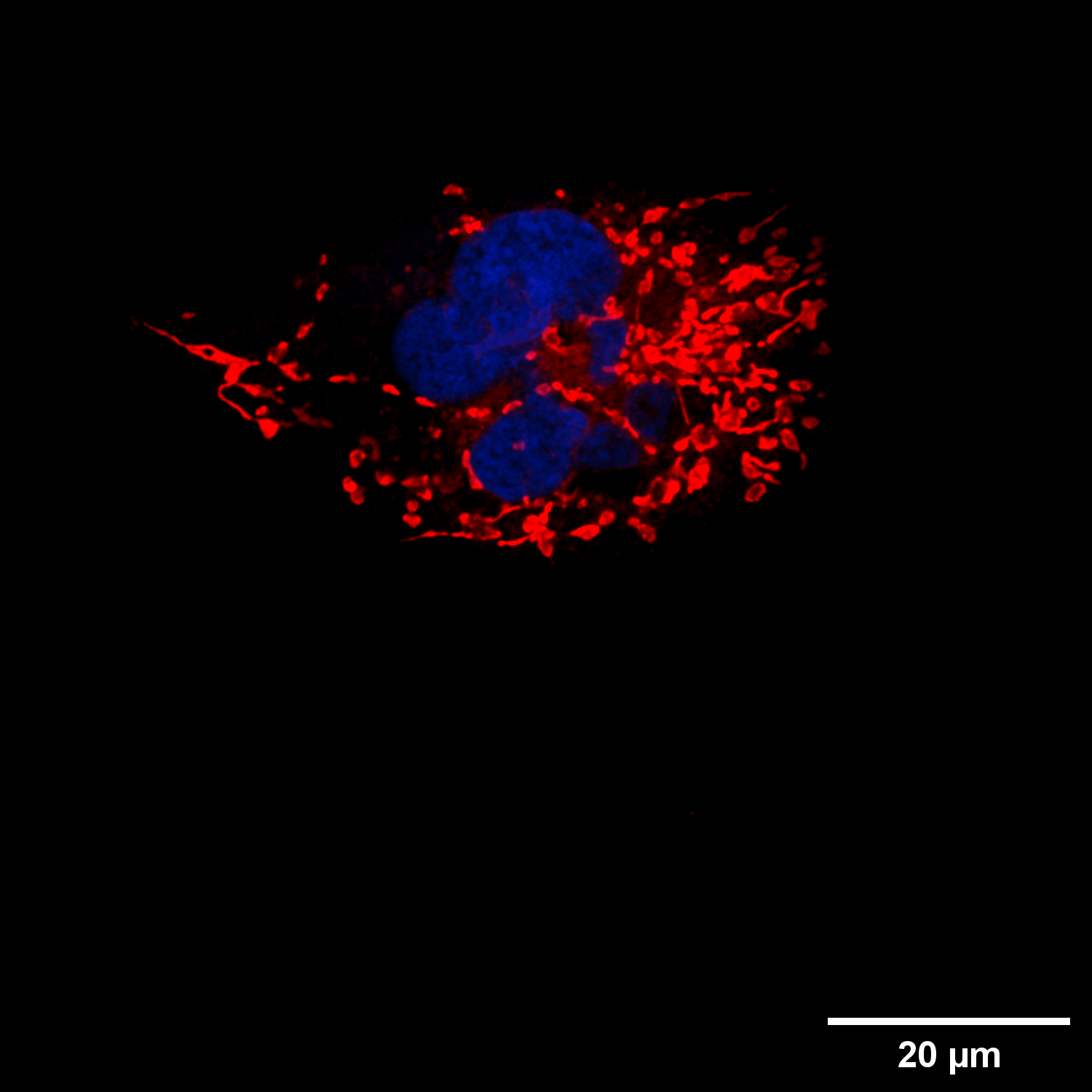

Supplement: Supplementary file 3 [file DataSheet4.zip › Mitotracker(1,2)/Mitotracker-2/Mitotracker-2═╝╞1⁄4/Iohexol+RU360/Ioh+RU360-2/2_RGB.tif]

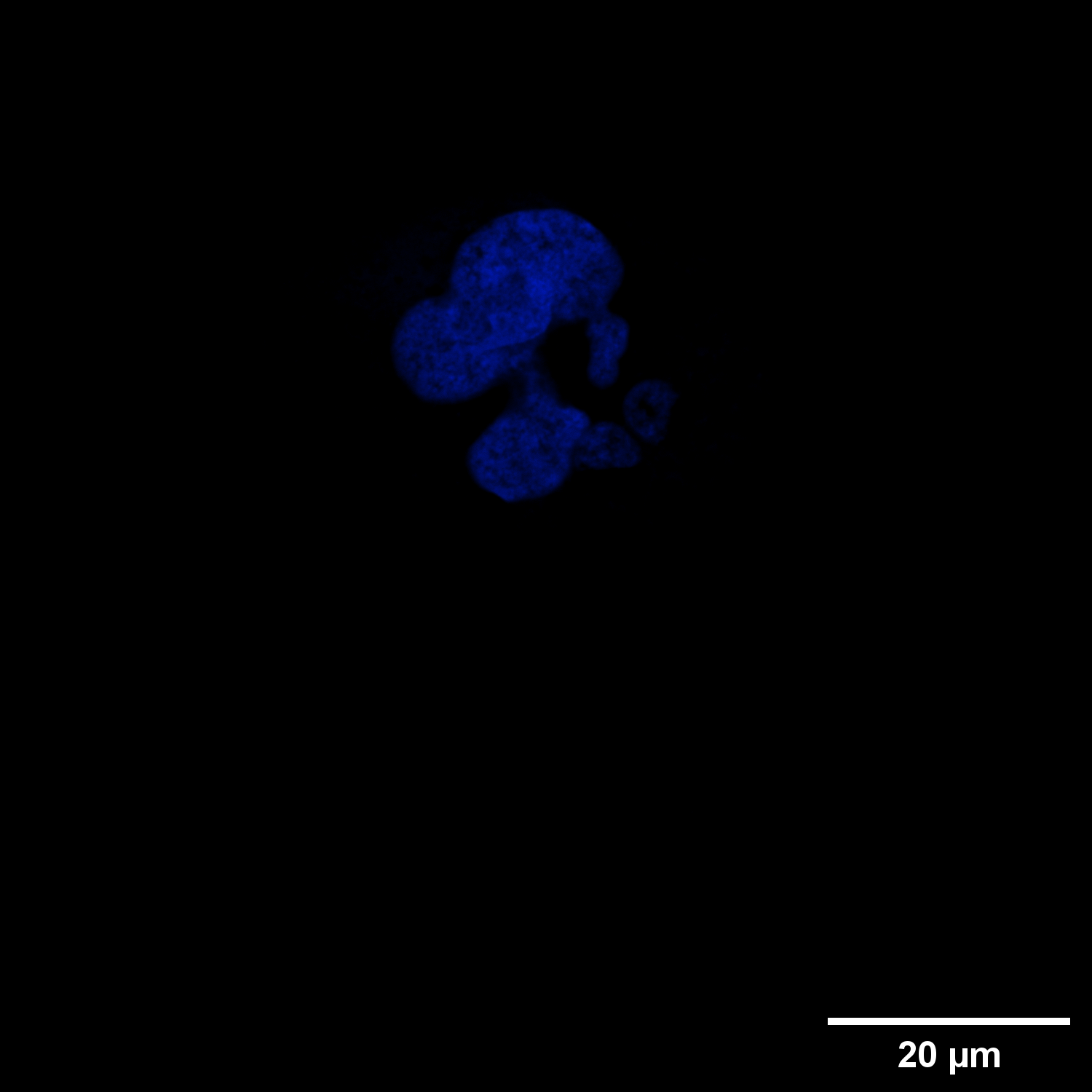

Supplement: Supplementary file 3 [file DataSheet4.zip › Mitotracker(1,2)/Mitotracker-2/Mitotracker-2═╝╞1⁄4/Iohexol+RU360/Ioh+RU360-2/2_RGB_SR405.tif]

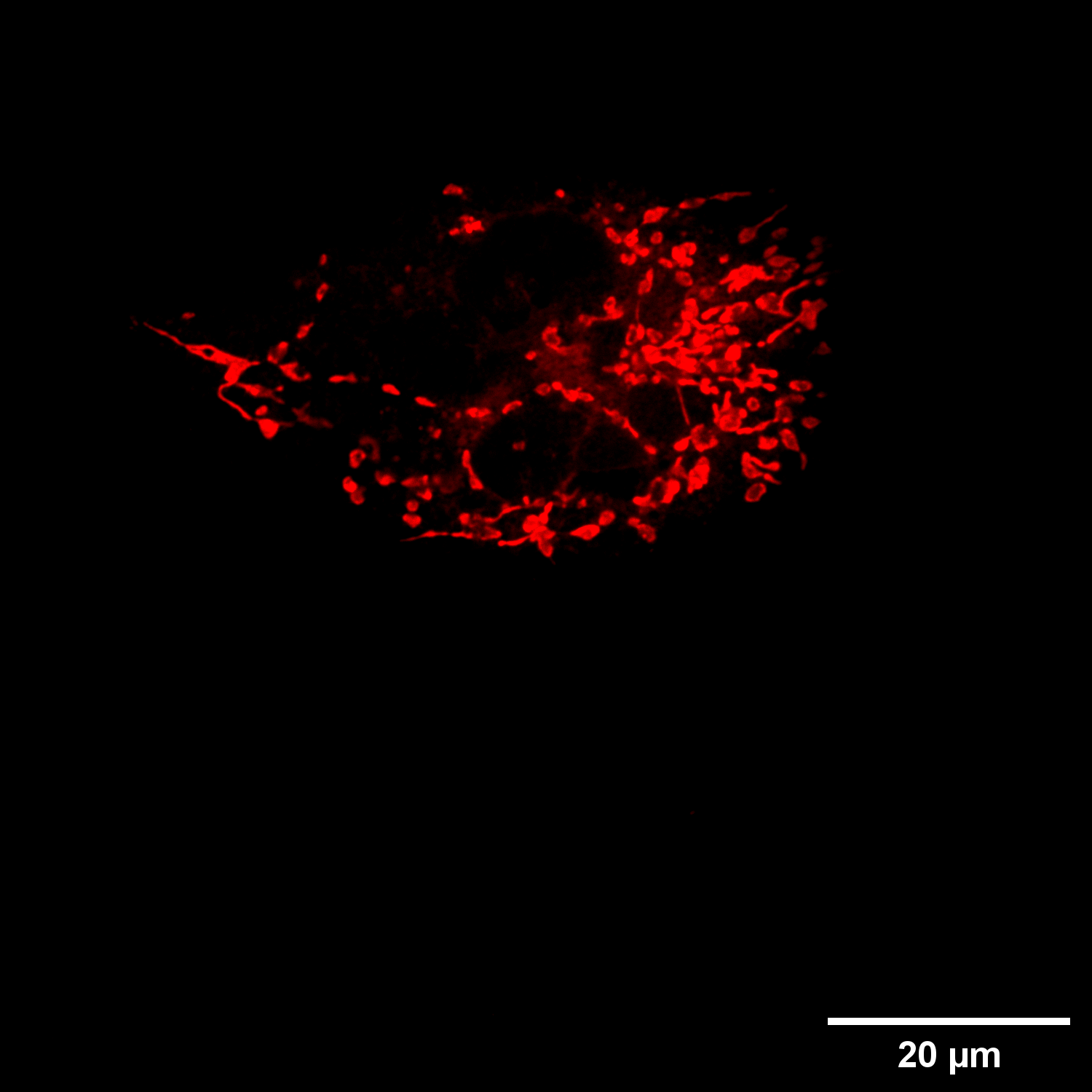

Supplement: Supplementary file 3 [file DataSheet4.zip › Mitotracker(1,2)/Mitotracker-2/Mitotracker-2═╝╞1⁄4/Iohexol+RU360/Ioh+RU360-2/2_RGB_SR561.tif]

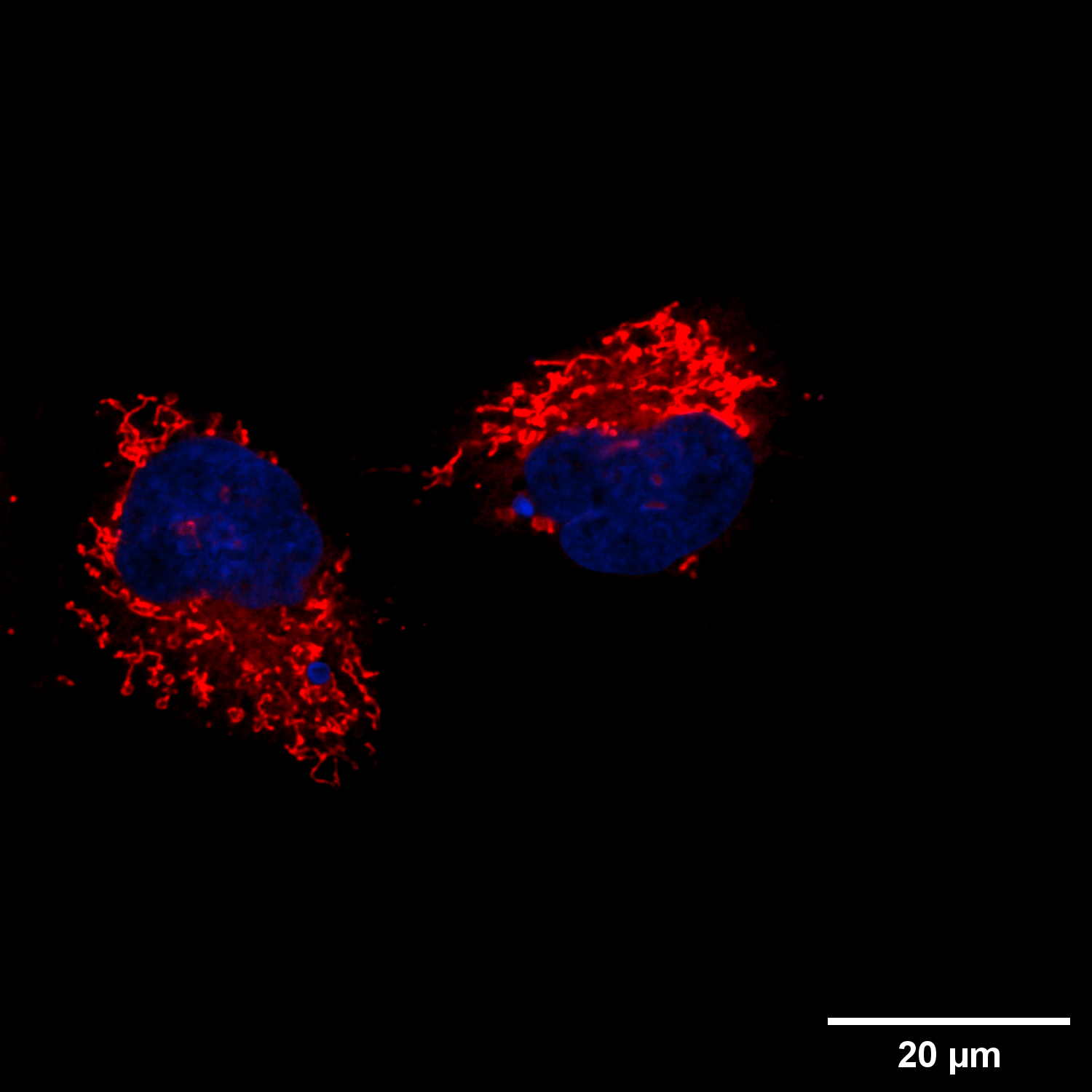

Supplement: Supplementary file 3 [file DataSheet4.zip › Mitotracker(1,2)/Mitotracker-2/Mitotracker-2═╝╞1⁄4/Iohexol+RU360/Ioh+RU360-3/3_RGB.tif]

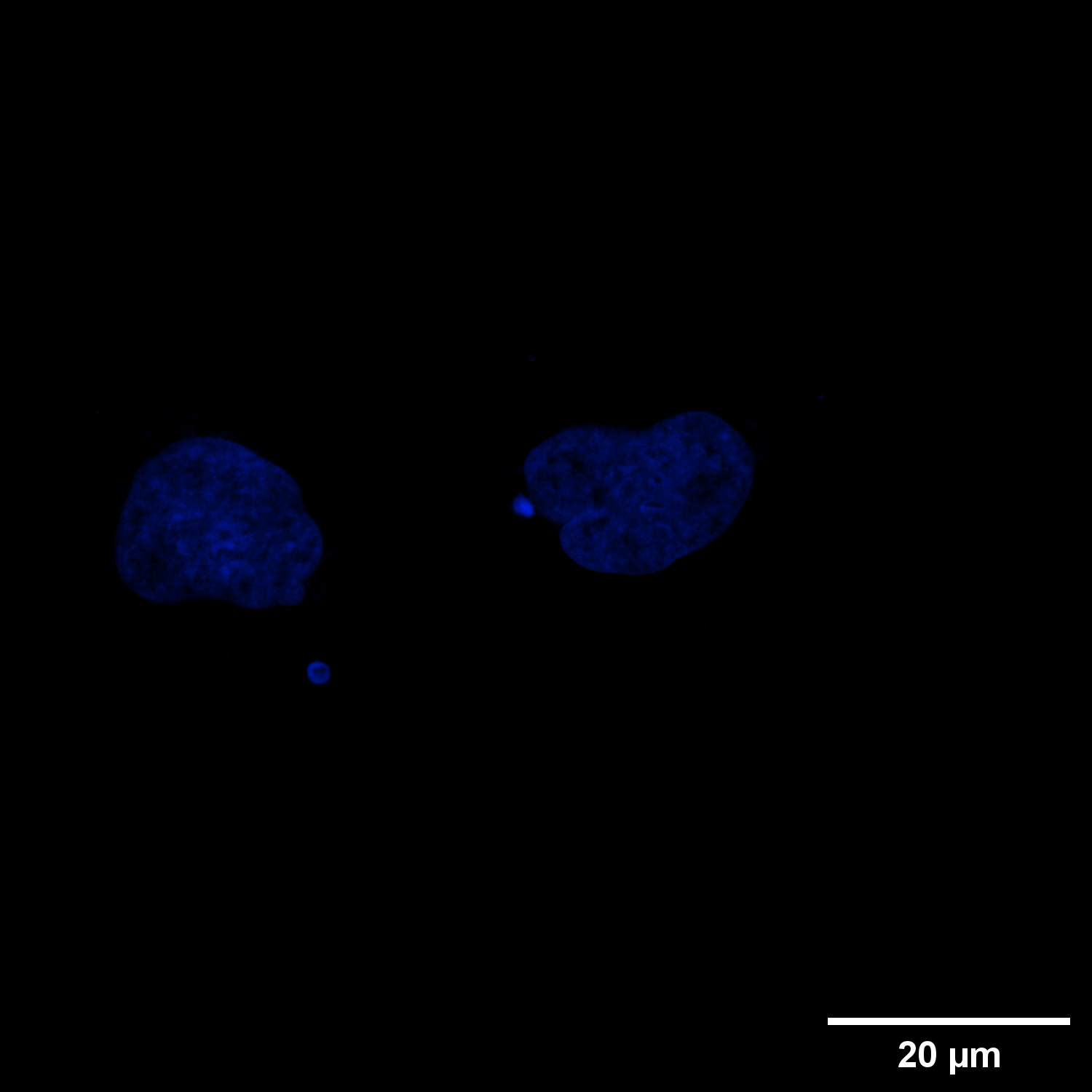

Supplement: Supplementary file 3 [file DataSheet4.zip › Mitotracker(1,2)/Mitotracker-2/Mitotracker-2═╝╞1⁄4/Iohexol+RU360/Ioh+RU360-3/3_RGB_SR405.tif]

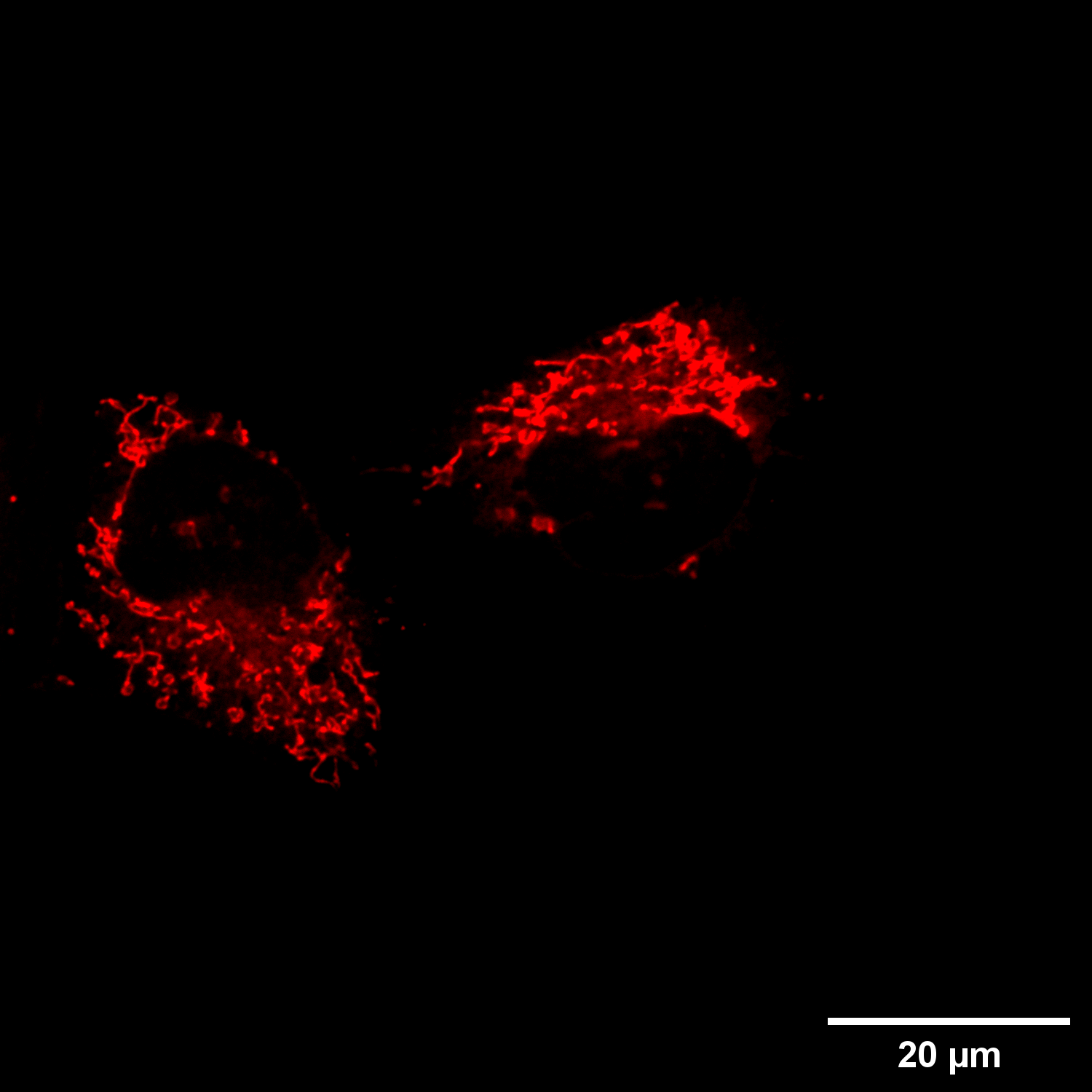

Supplement: Supplementary file 3 [file DataSheet4.zip › Mitotracker(1,2)/Mitotracker-2/Mitotracker-2═╝╞1⁄4/Iohexol+RU360/Ioh+RU360-3/3_RGB_SR561.tif]

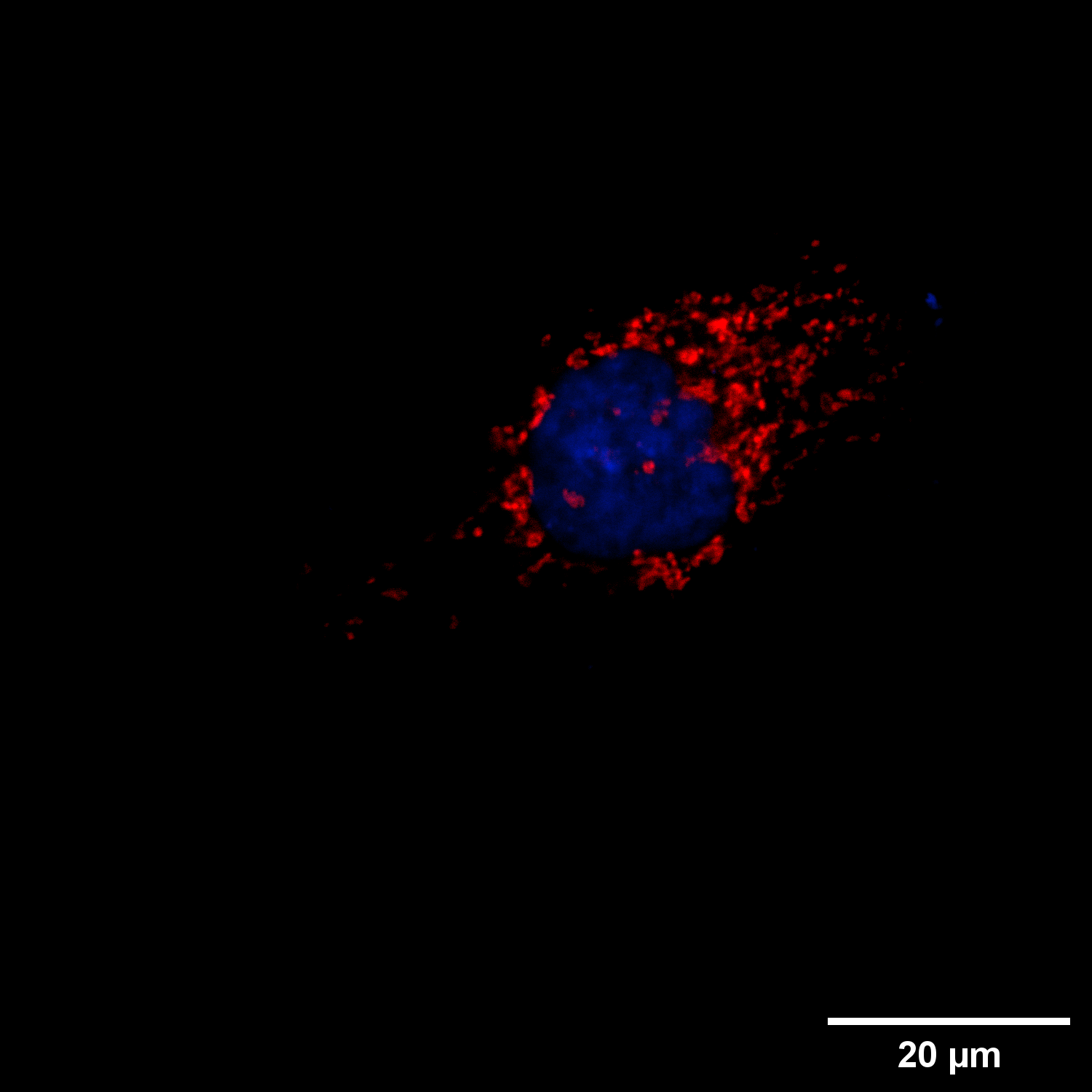

Supplement: Supplementary file 3 [file DataSheet4.zip › Mitotracker(1,2)/Mitotracker-2/Mitotracker-2═╝╞1⁄4/Iohexol+Spermine/Ioh+Spermine-1/1_RGB.tif]

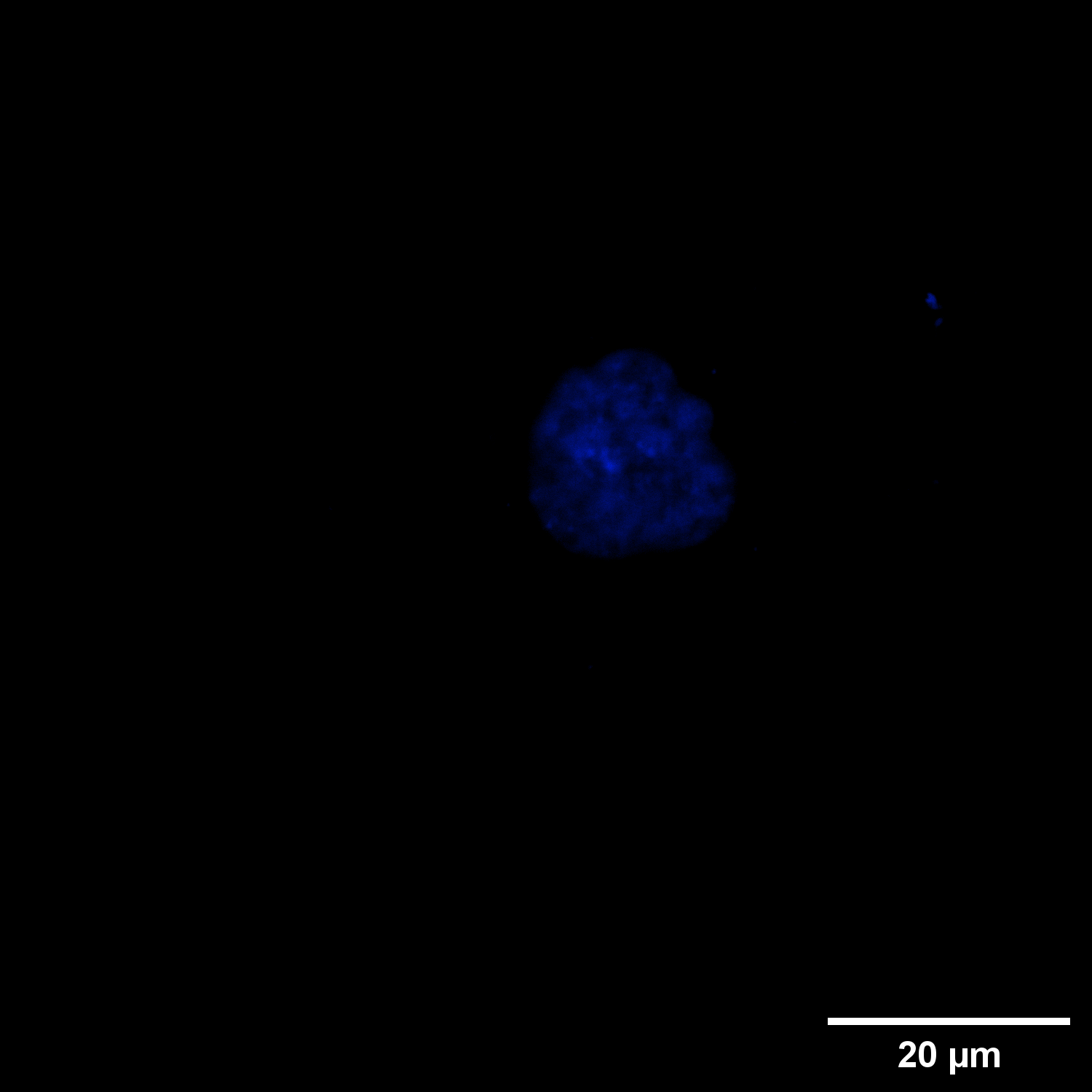

Supplement: Supplementary file 3 [file DataSheet4.zip › Mitotracker(1,2)/Mitotracker-2/Mitotracker-2═╝╞1⁄4/Iohexol+Spermine/Ioh+Spermine-1/1_RGB_SR405.tif]

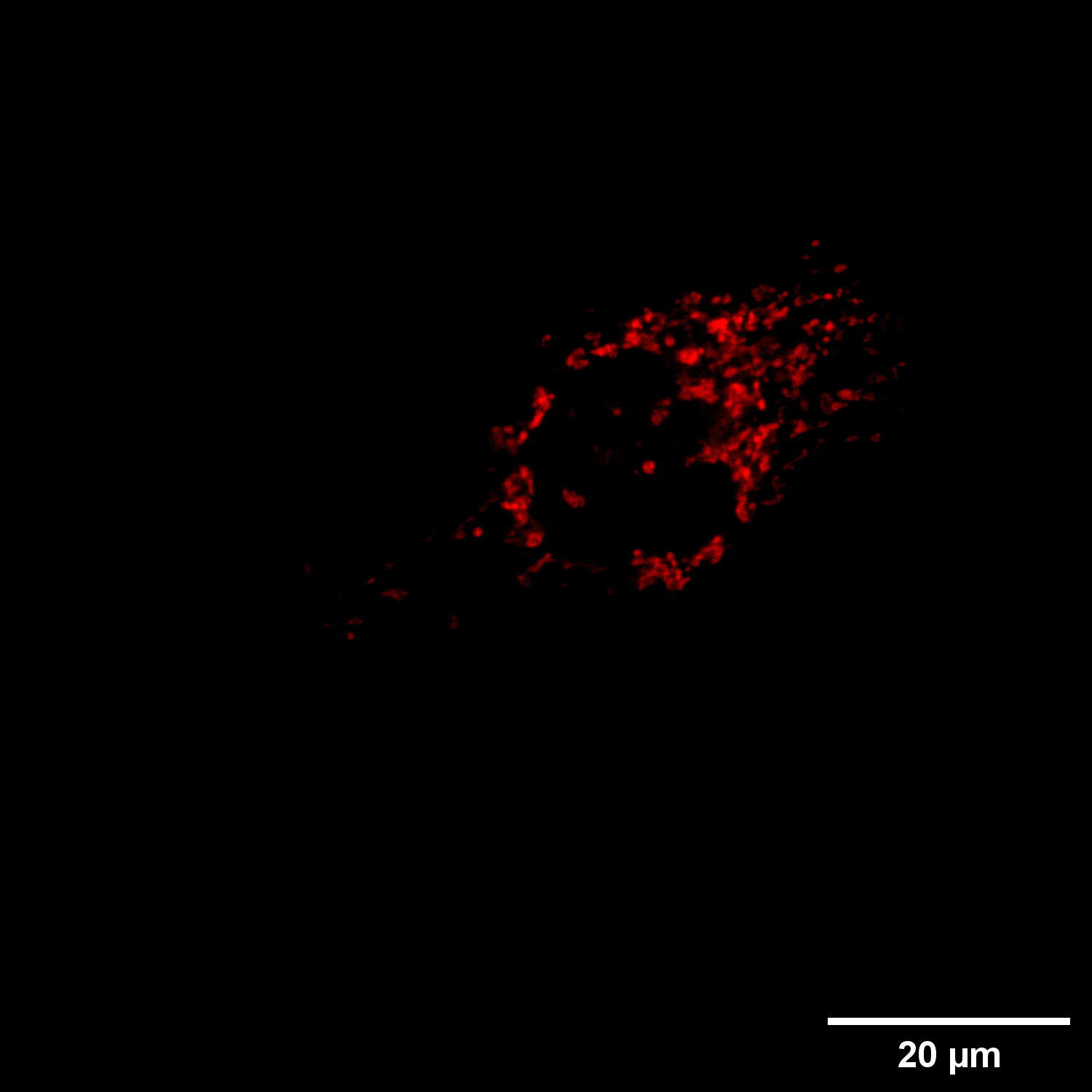

Supplement: Supplementary file 3 [file DataSheet4.zip › Mitotracker(1,2)/Mitotracker-2/Mitotracker-2═╝╞1⁄4/Iohexol+Spermine/Ioh+Spermine-1/1_RGB_SR561.tif]

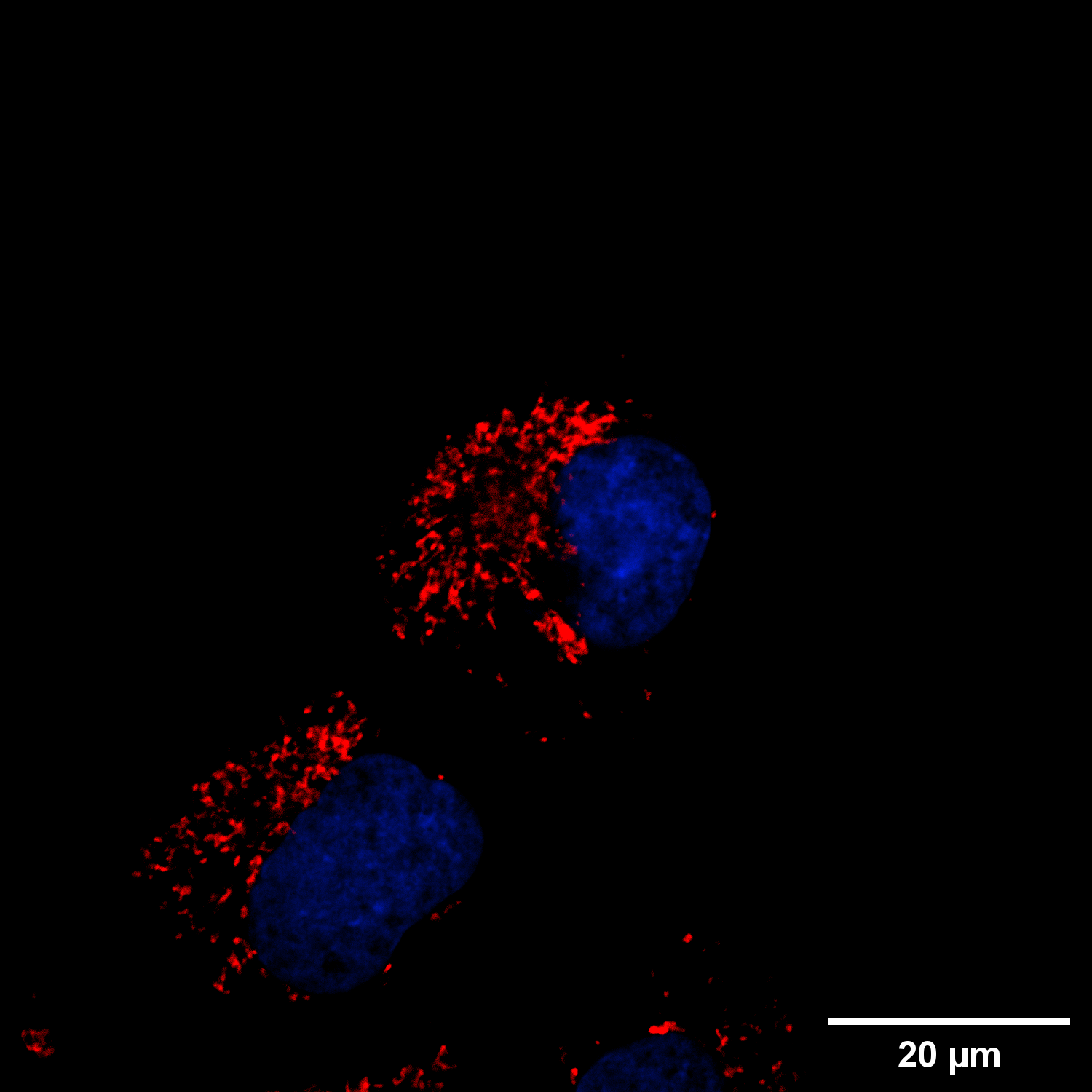

Supplement: Supplementary file 3 [file DataSheet4.zip › Mitotracker(1,2)/Mitotracker-2/Mitotracker-2═╝╞1⁄4/Iohexol+Spermine/Ioh+Spermine-2/2_RGB.tif]

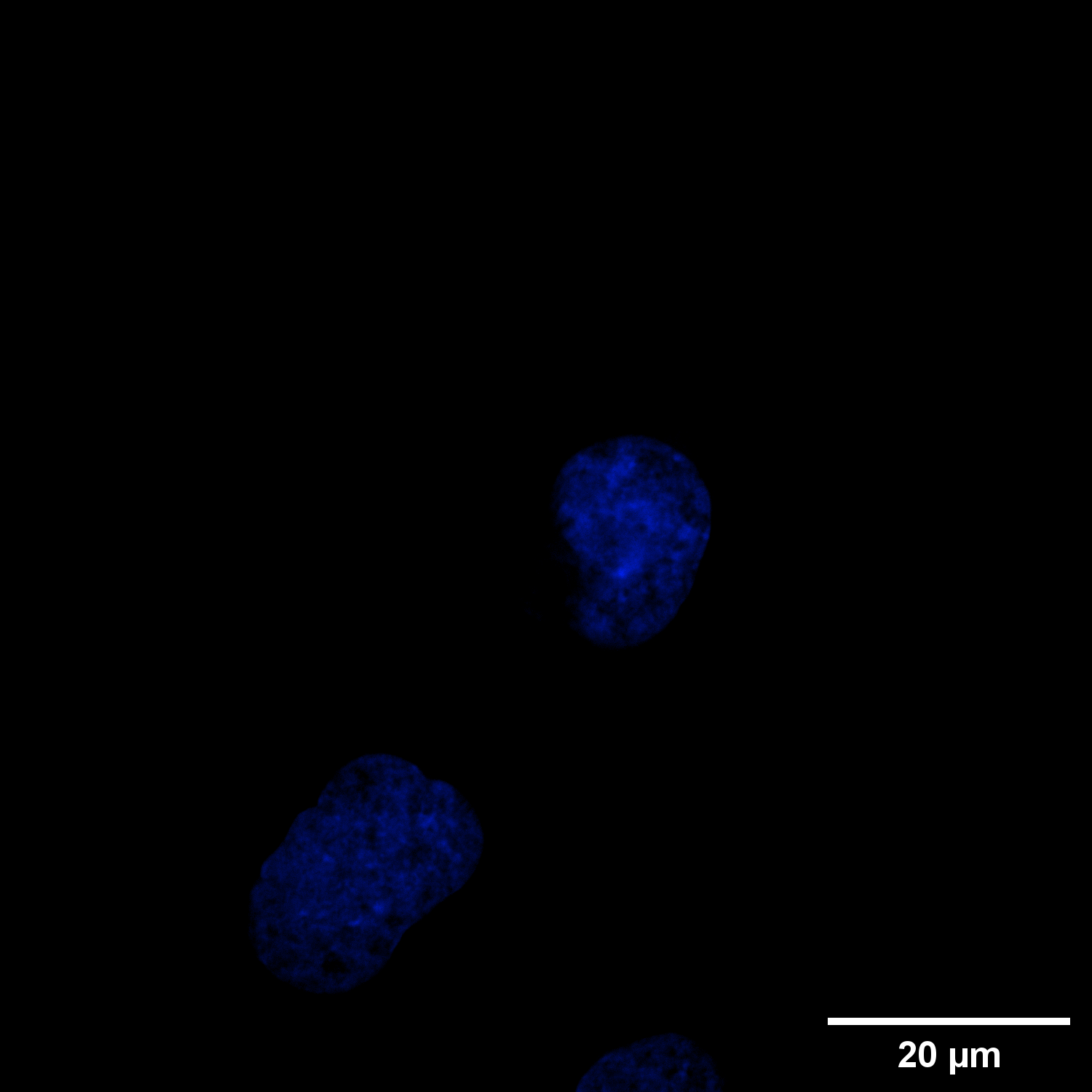

Supplement: Supplementary file 3 [file DataSheet4.zip › Mitotracker(1,2)/Mitotracker-2/Mitotracker-2═╝╞1⁄4/Iohexol+Spermine/Ioh+Spermine-2/2_RGB_SR405.tif]

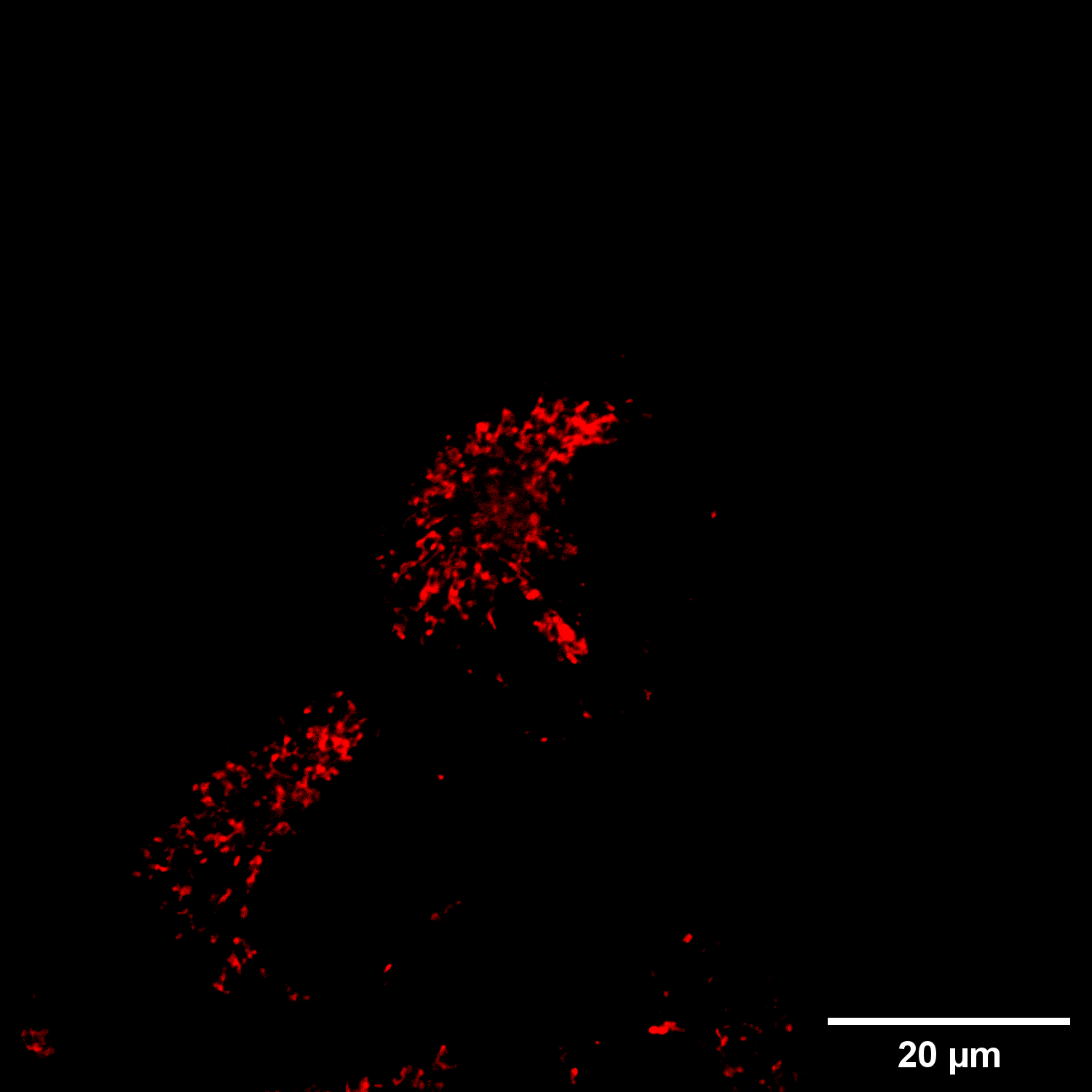

Supplement: Supplementary file 3 [file DataSheet4.zip › Mitotracker(1,2)/Mitotracker-2/Mitotracker-2═╝╞1⁄4/Iohexol+Spermine/Ioh+Spermine-2/2_RGB_SR561.tif]

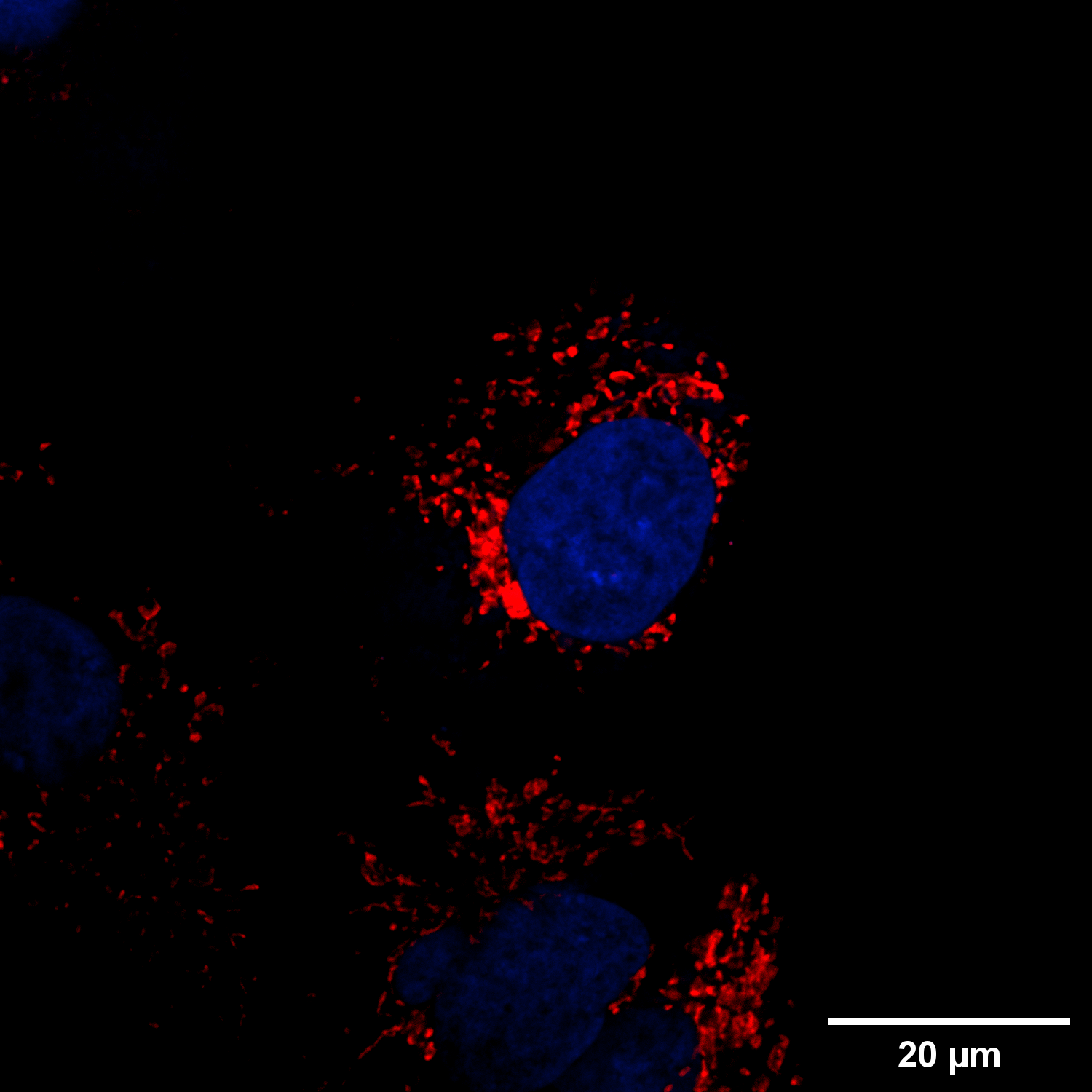

Supplement: Supplementary file 3 [file DataSheet4.zip › Mitotracker(1,2)/Mitotracker-2/Mitotracker-2═╝╞1⁄4/Iohexol+Spermine/Ioh+Spermine-3/3_RGB.tif]

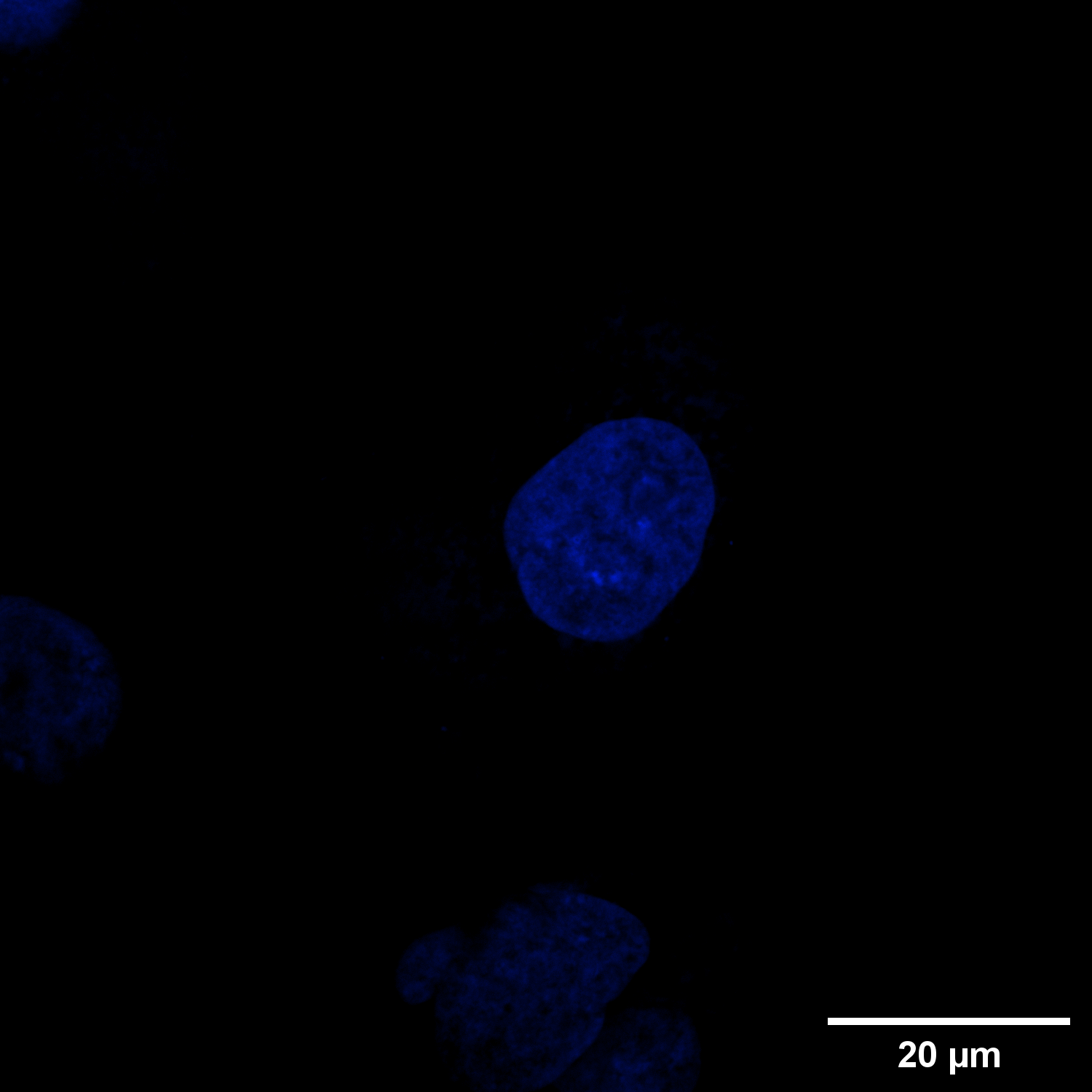

Supplement: Supplementary file 3 [file DataSheet4.zip › Mitotracker(1,2)/Mitotracker-2/Mitotracker-2═╝╞1⁄4/Iohexol+Spermine/Ioh+Spermine-3/3_RGB_SR405.tif]

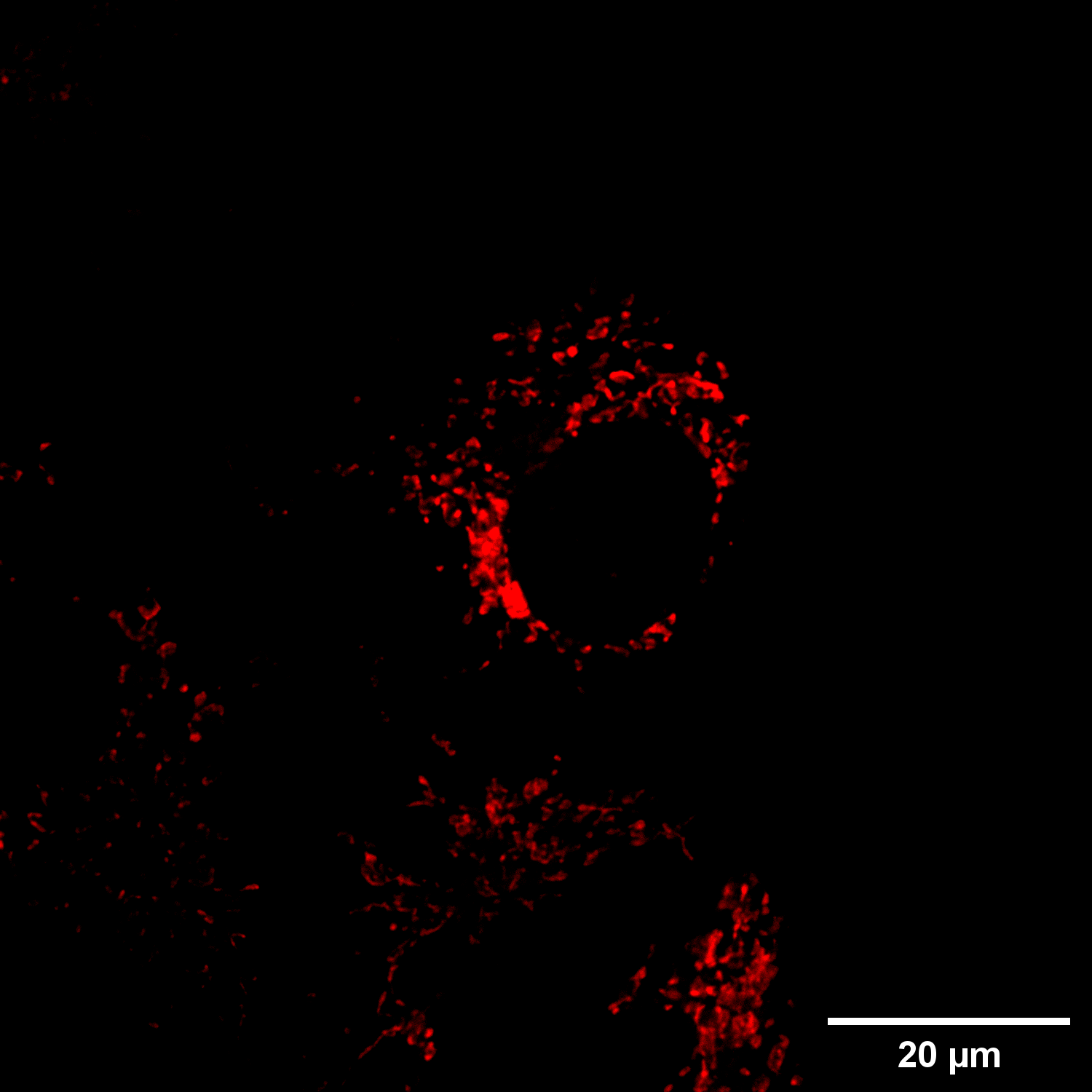

Supplement: Supplementary file 3 [file DataSheet4.zip › Mitotracker(1,2)/Mitotracker-2/Mitotracker-2═╝╞1⁄4/Iohexol+Spermine/Ioh+Spermine-3/3_RGB_SR561.tif]

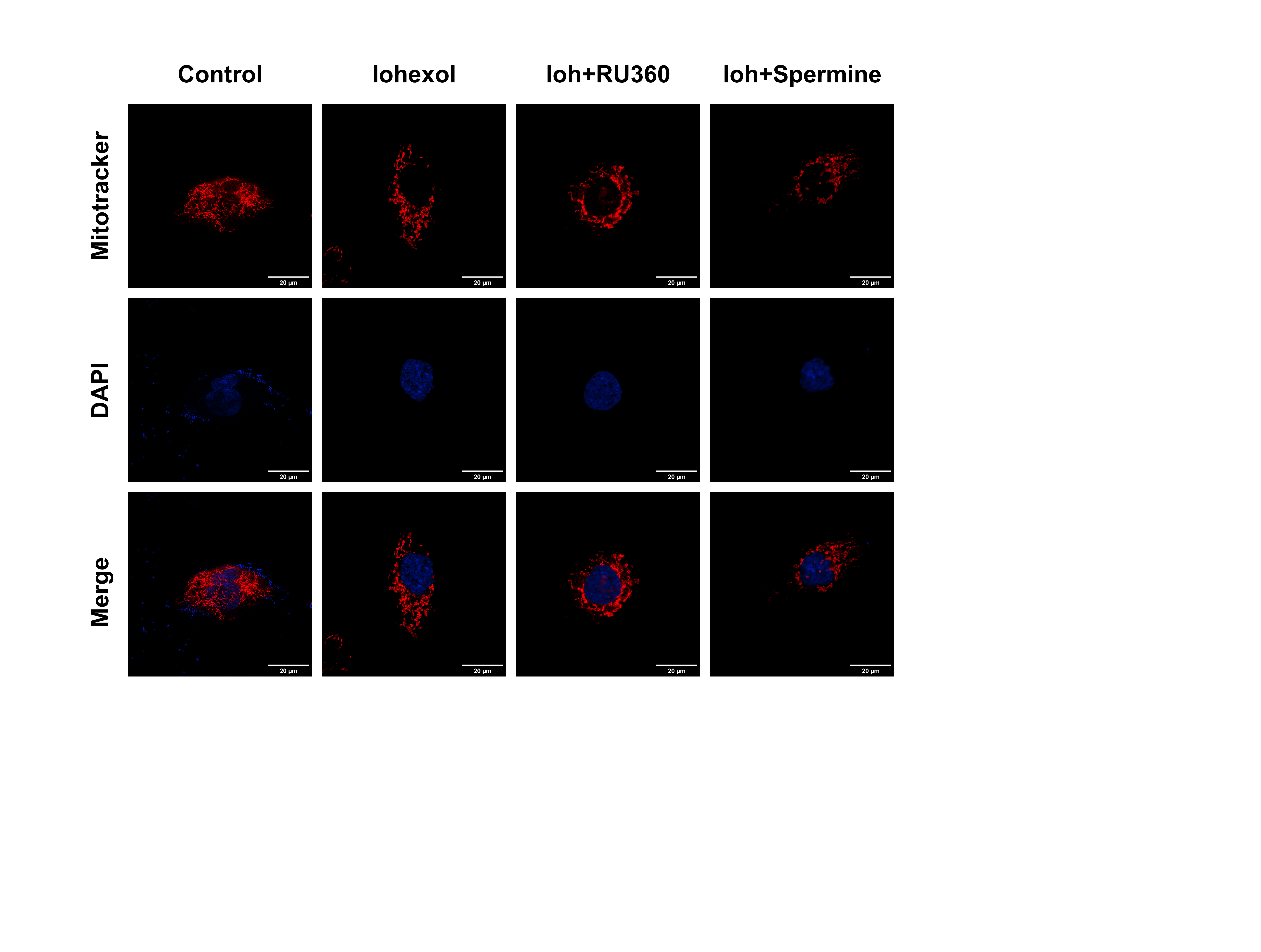

Supplement: Supplementary file 3 [file DataSheet4.zip › Mitotracker(1,2)/Mitotracker-2/Mitotracker-2═╝╞1⁄4/mitotracker-2║╧═╝.tif]

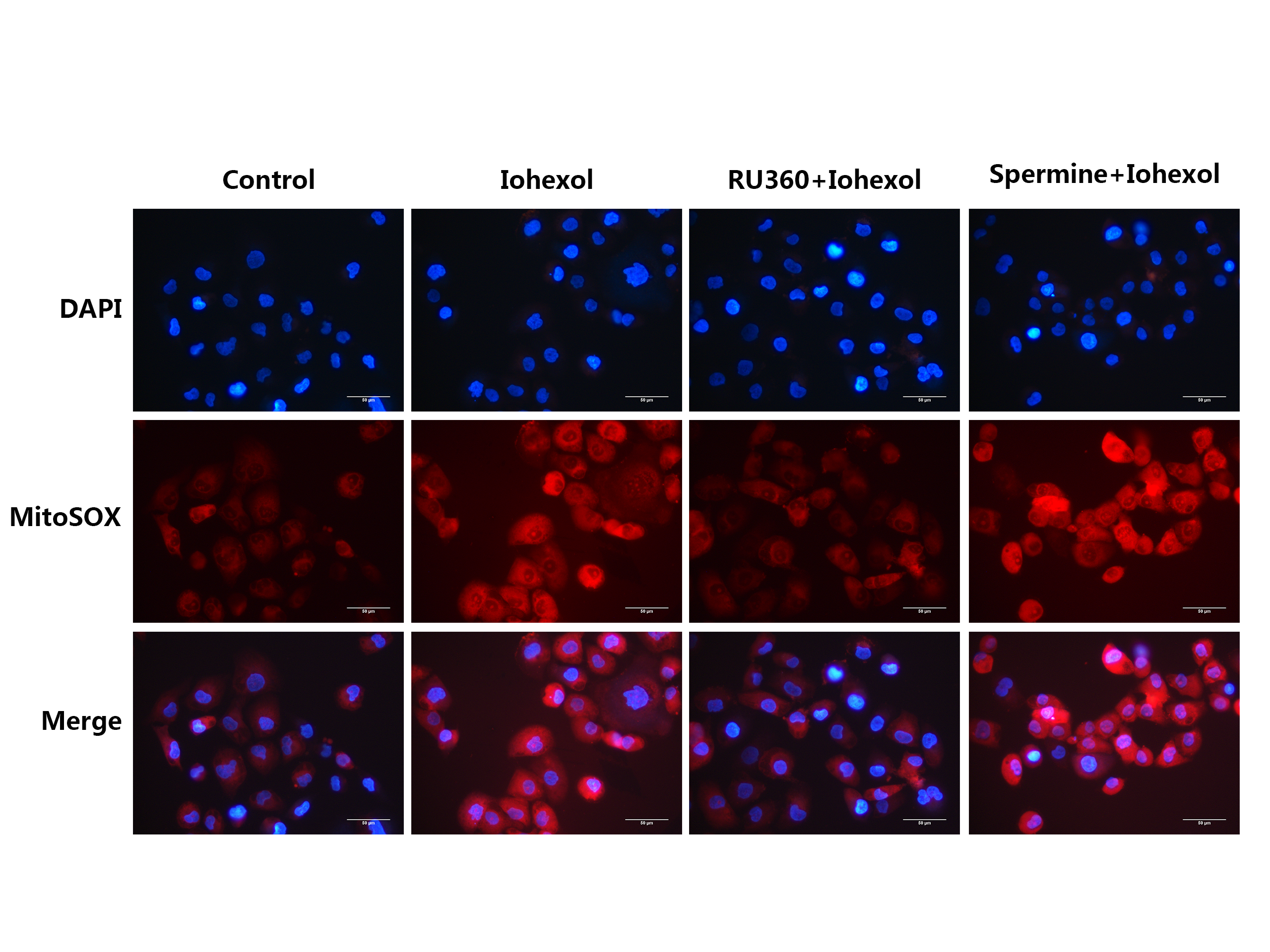

Supplement: Supplementary file 5 [file DataSheet6.zip › MitoSOX-2/MitoSOX═╝╞1⁄4/2 MitoSOX.tif]

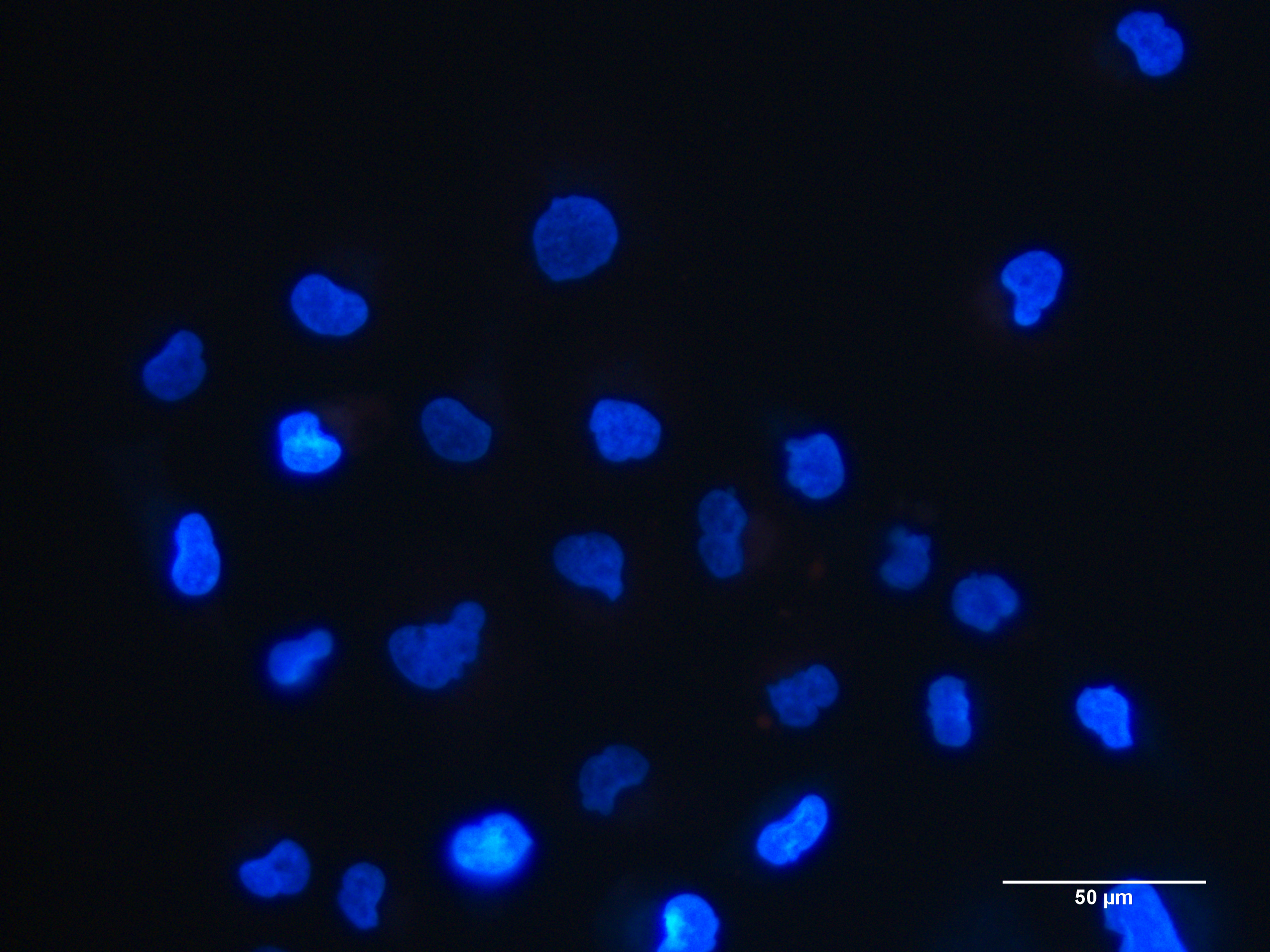

Supplement: Supplementary file 5 [file DataSheet6.zip › MitoSOX-2/MitoSOX═╝╞1⁄4/Control 1 DAPI.tif]

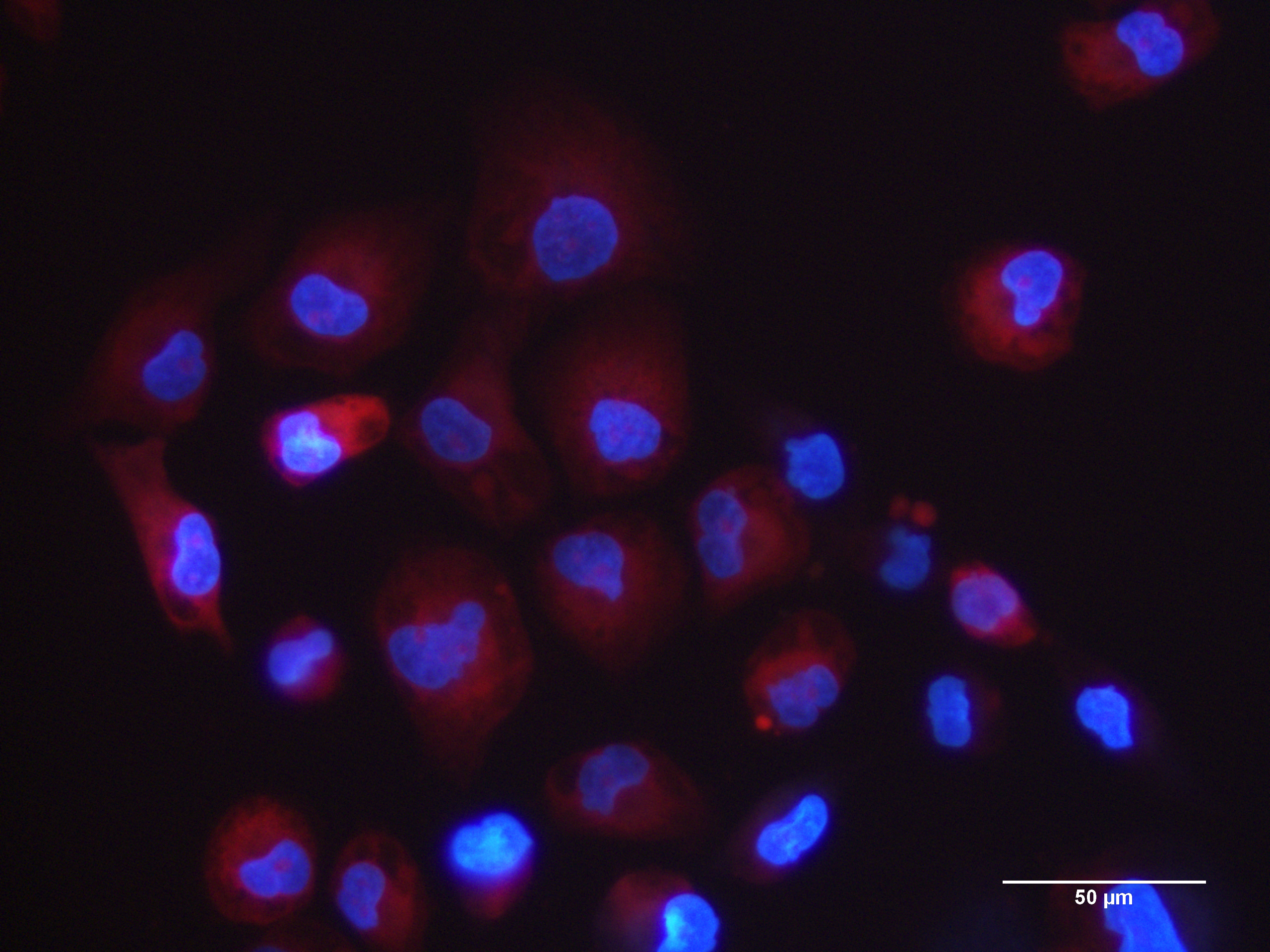

Supplement: Supplementary file 5 [file DataSheet6.zip › MitoSOX-2/MitoSOX═╝╞1⁄4/Control 1 merge.tif]

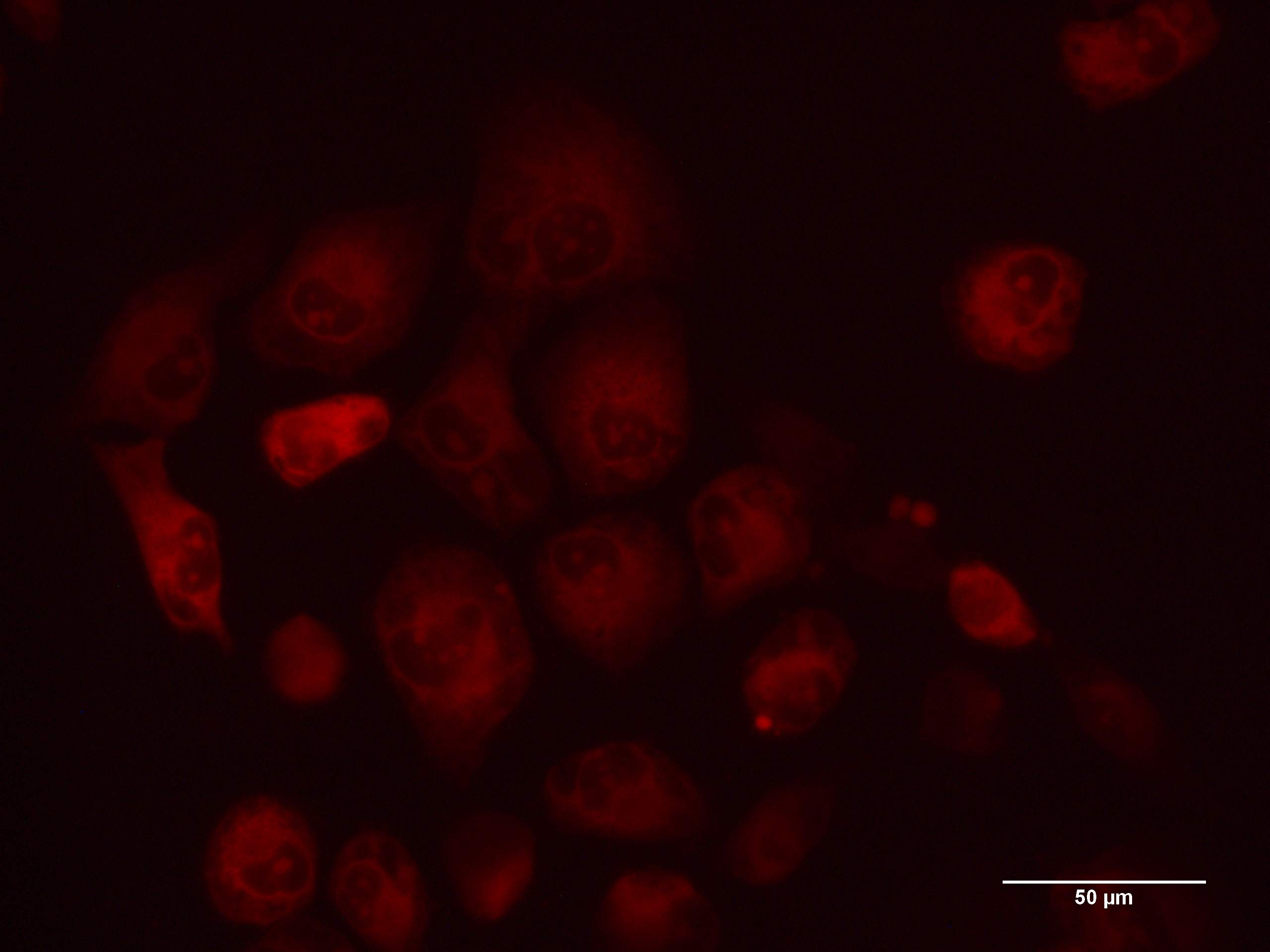

Supplement: Supplementary file 5 [file DataSheet6.zip › MitoSOX-2/MitoSOX═╝╞1⁄4/Control 1 MitoSOX.tif]

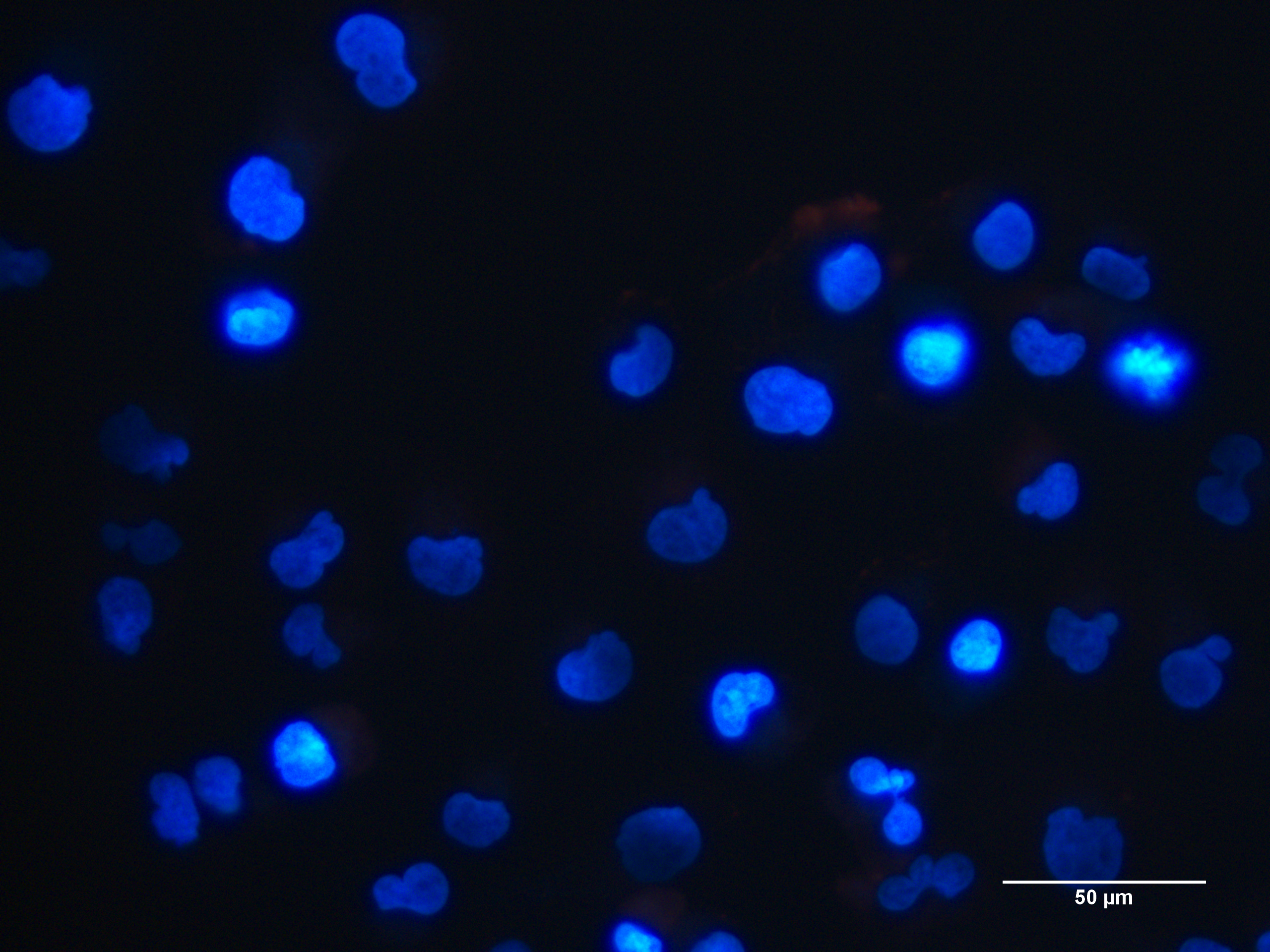

Supplement: Supplementary file 5 [file DataSheet6.zip › MitoSOX-2/MitoSOX═╝╞1⁄4/Control 2 DAPI.tif]

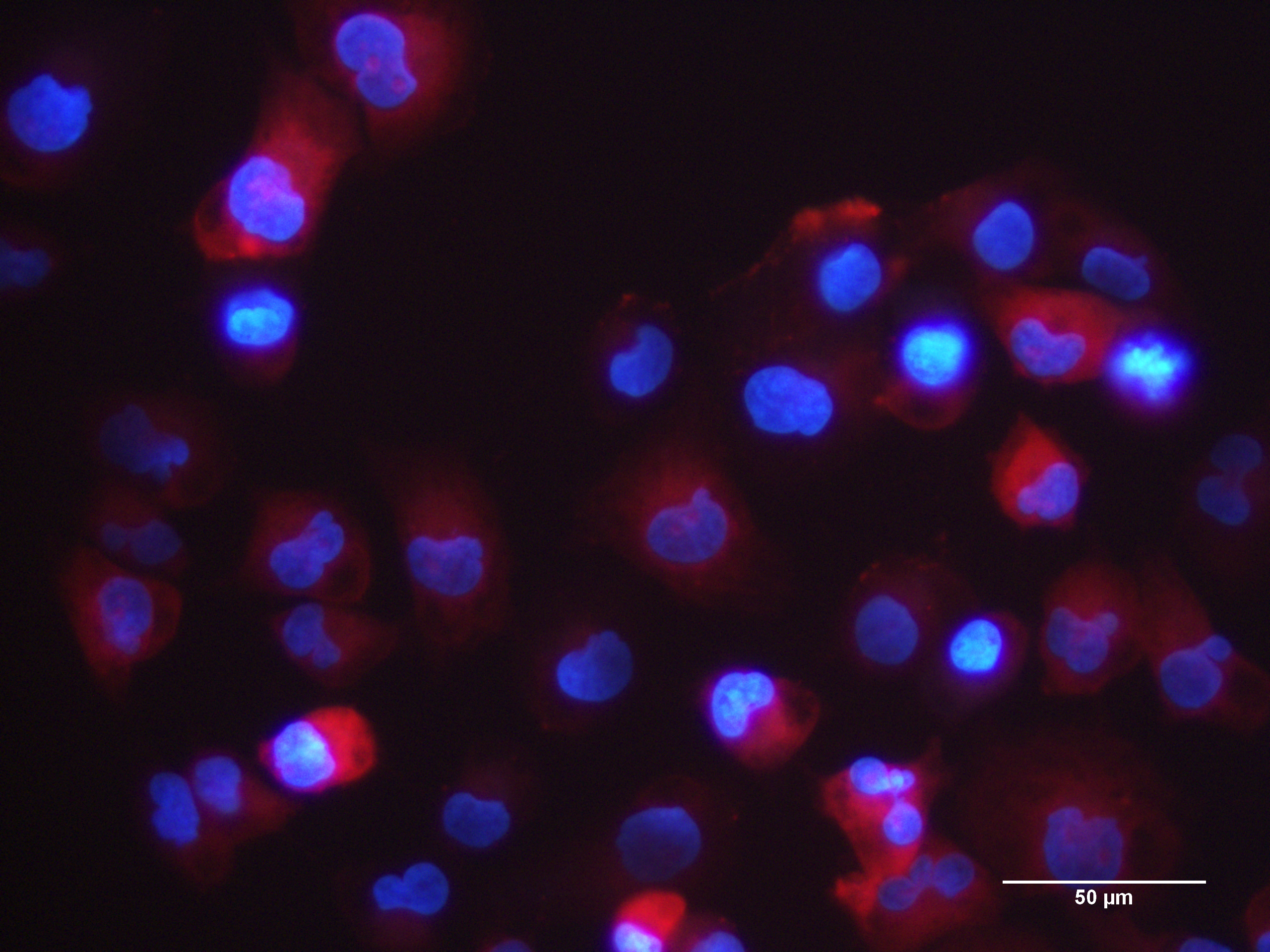

Supplement: Supplementary file 5 [file DataSheet6.zip › MitoSOX-2/MitoSOX═╝╞1⁄4/Control 2 merge.tif]

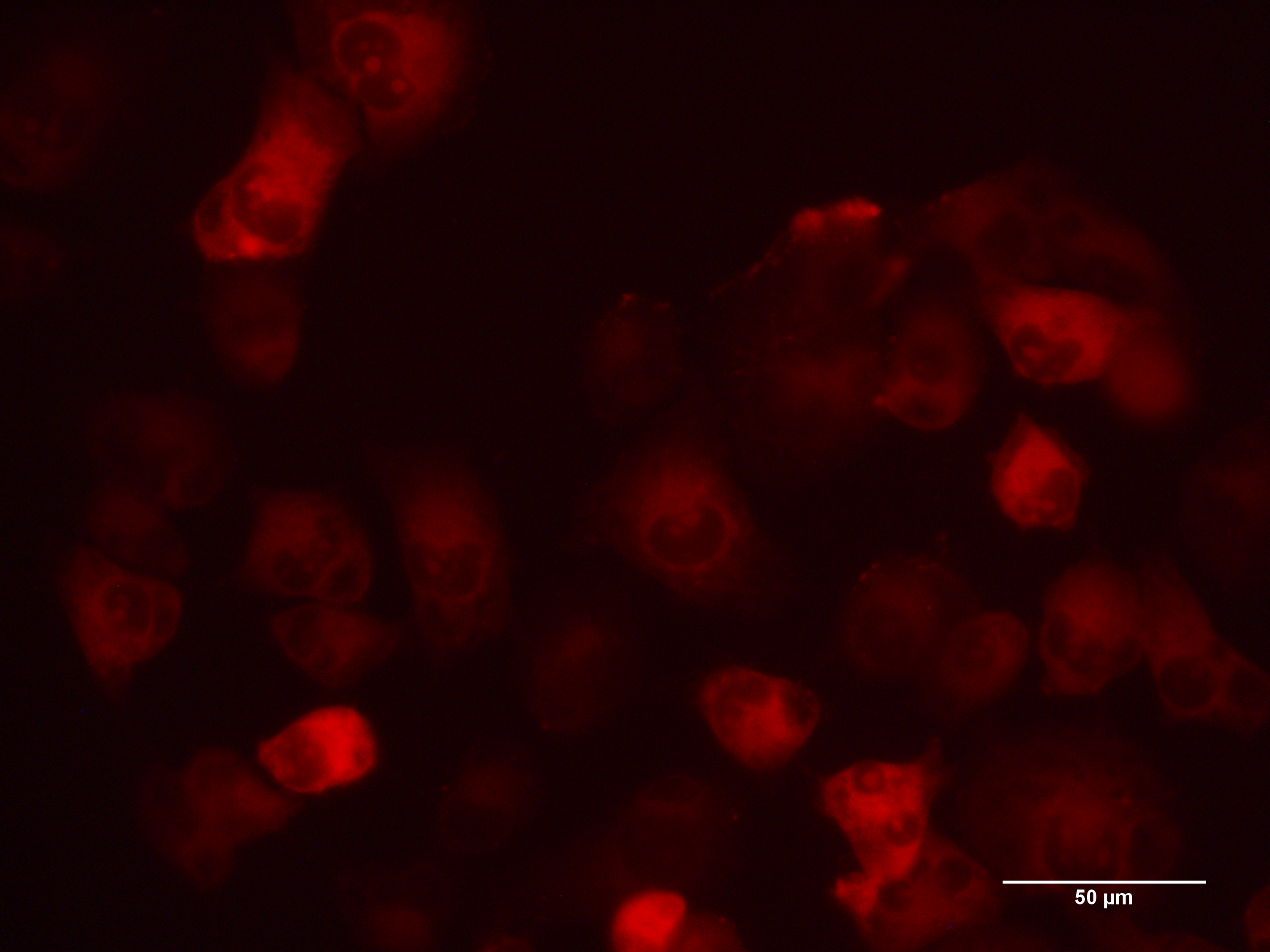

Supplement: Supplementary file 5 [file DataSheet6.zip › MitoSOX-2/MitoSOX═╝╞1⁄4/Control 2 MitoSOX.tif]

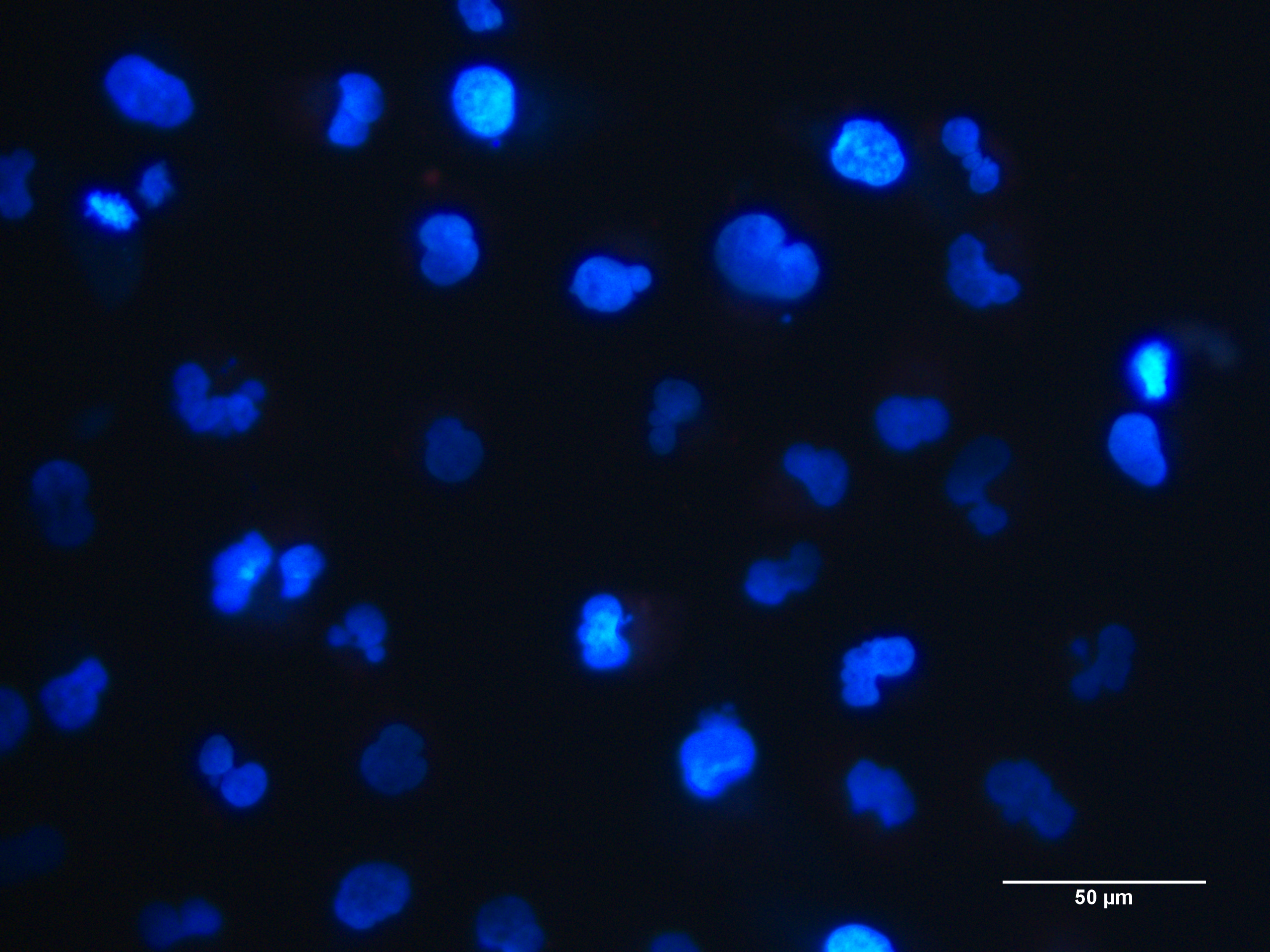

Supplement: Supplementary file 5 [file DataSheet6.zip › MitoSOX-2/MitoSOX═╝╞1⁄4/Control 3 DAPI.tif]

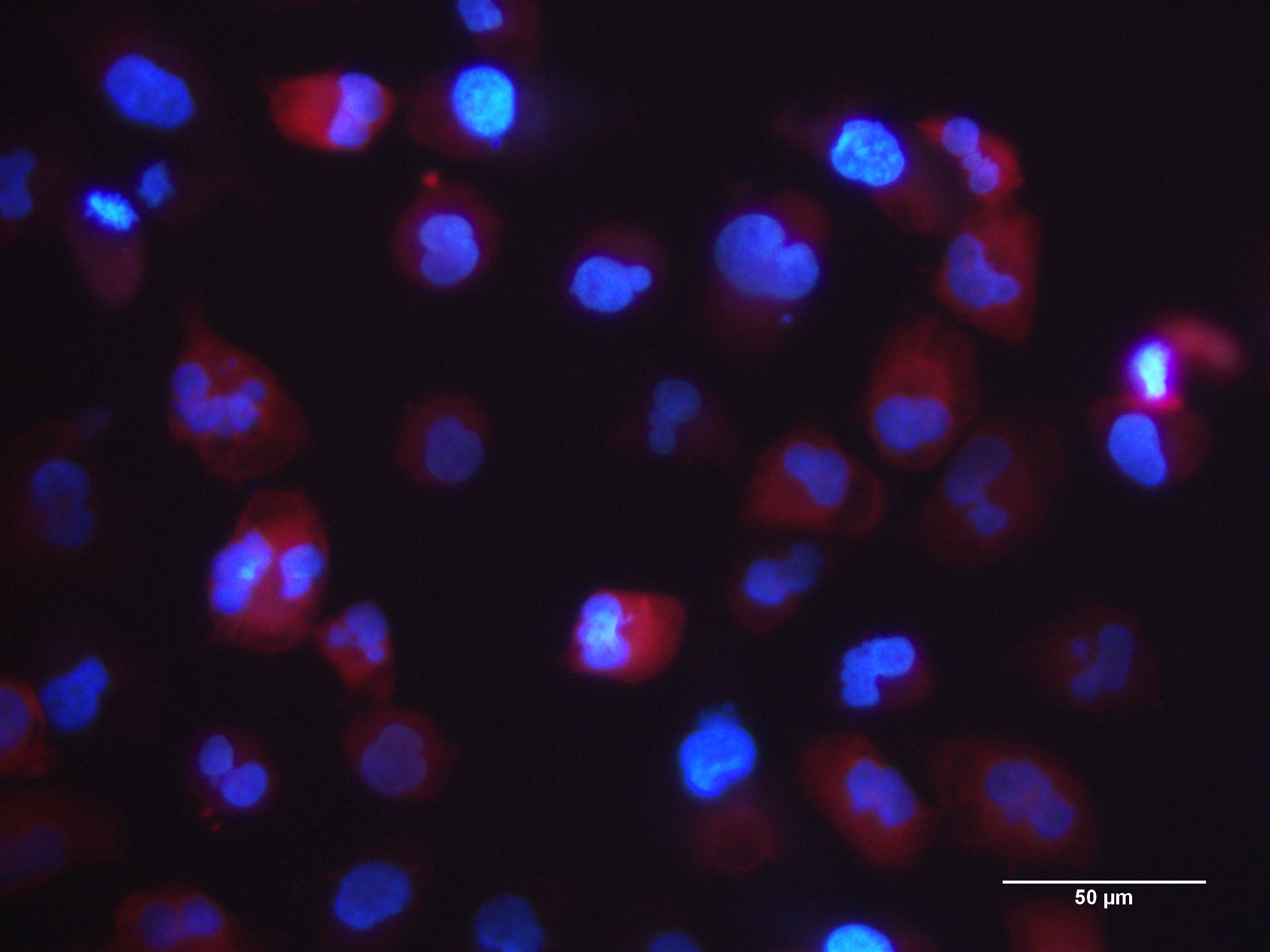

Supplement: Supplementary file 5 [file DataSheet6.zip › MitoSOX-2/MitoSOX═╝╞1⁄4/Control 3 merge.tif]

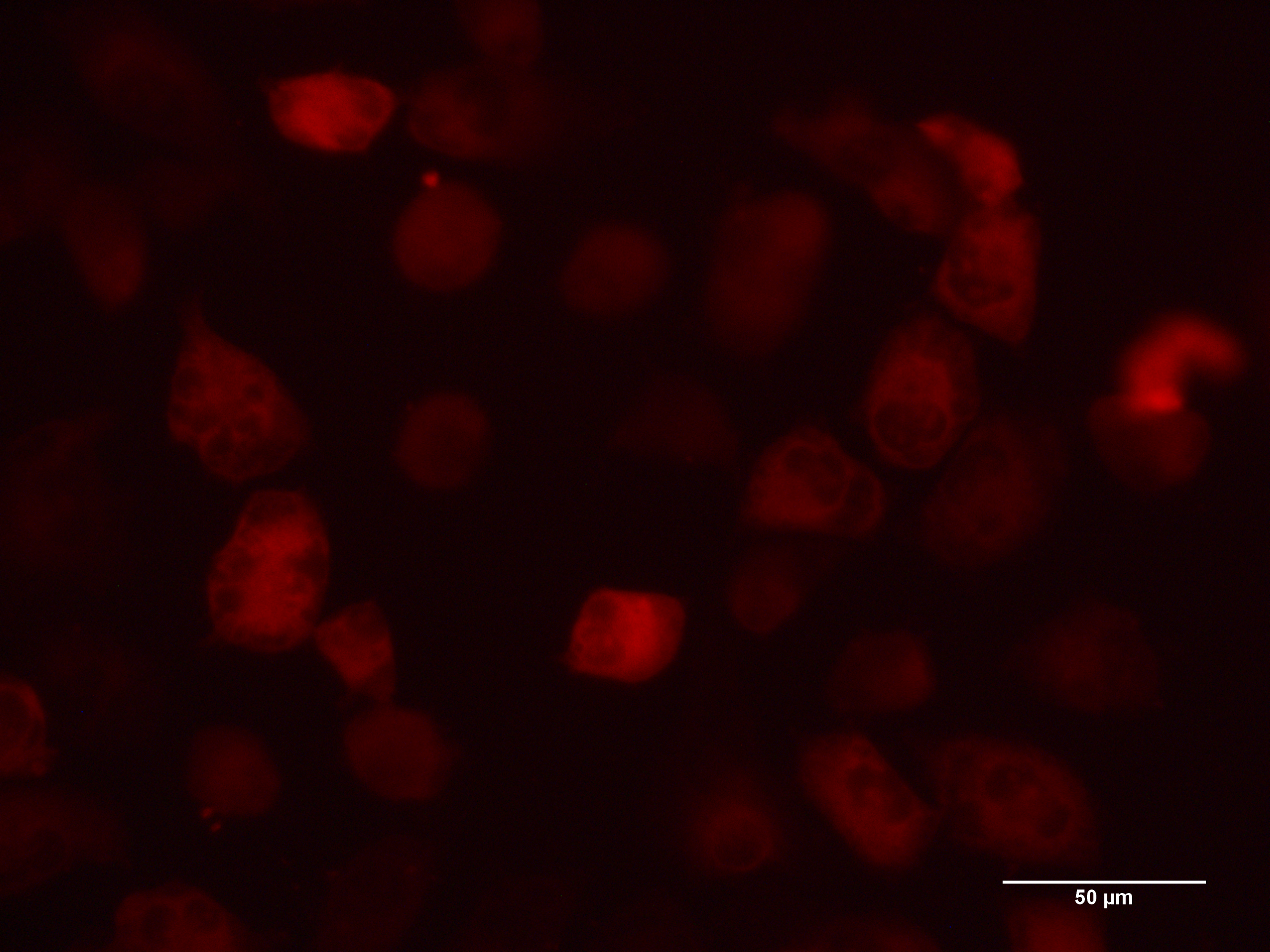

Supplement: Supplementary file 5 [file DataSheet6.zip › MitoSOX-2/MitoSOX═╝╞1⁄4/Control 3 MitoSOX.tif]

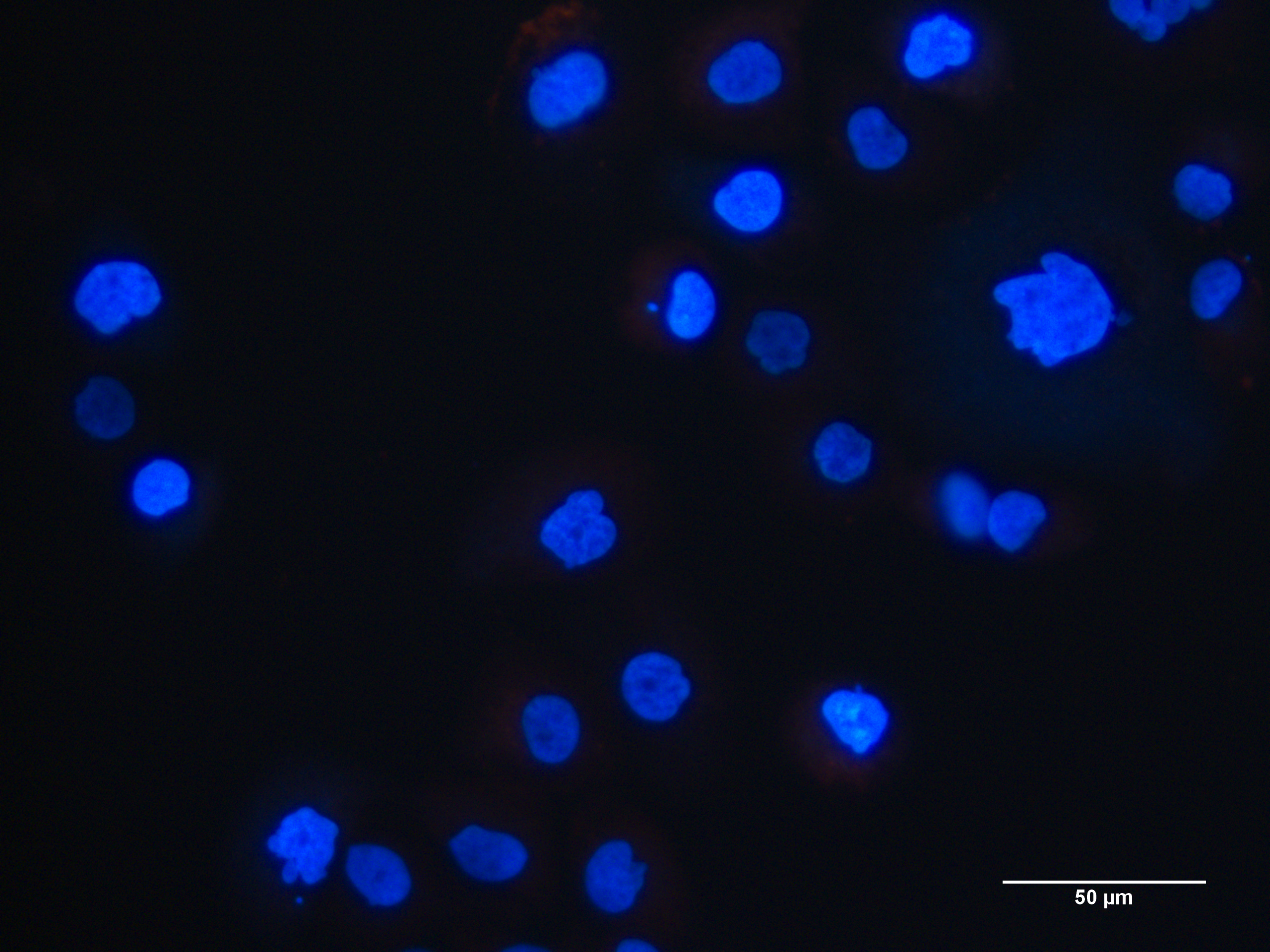

Supplement: Supplementary file 5 [file DataSheet6.zip › MitoSOX-2/MitoSOX═╝╞1⁄4/Iohexol 1 DAPI.tif]

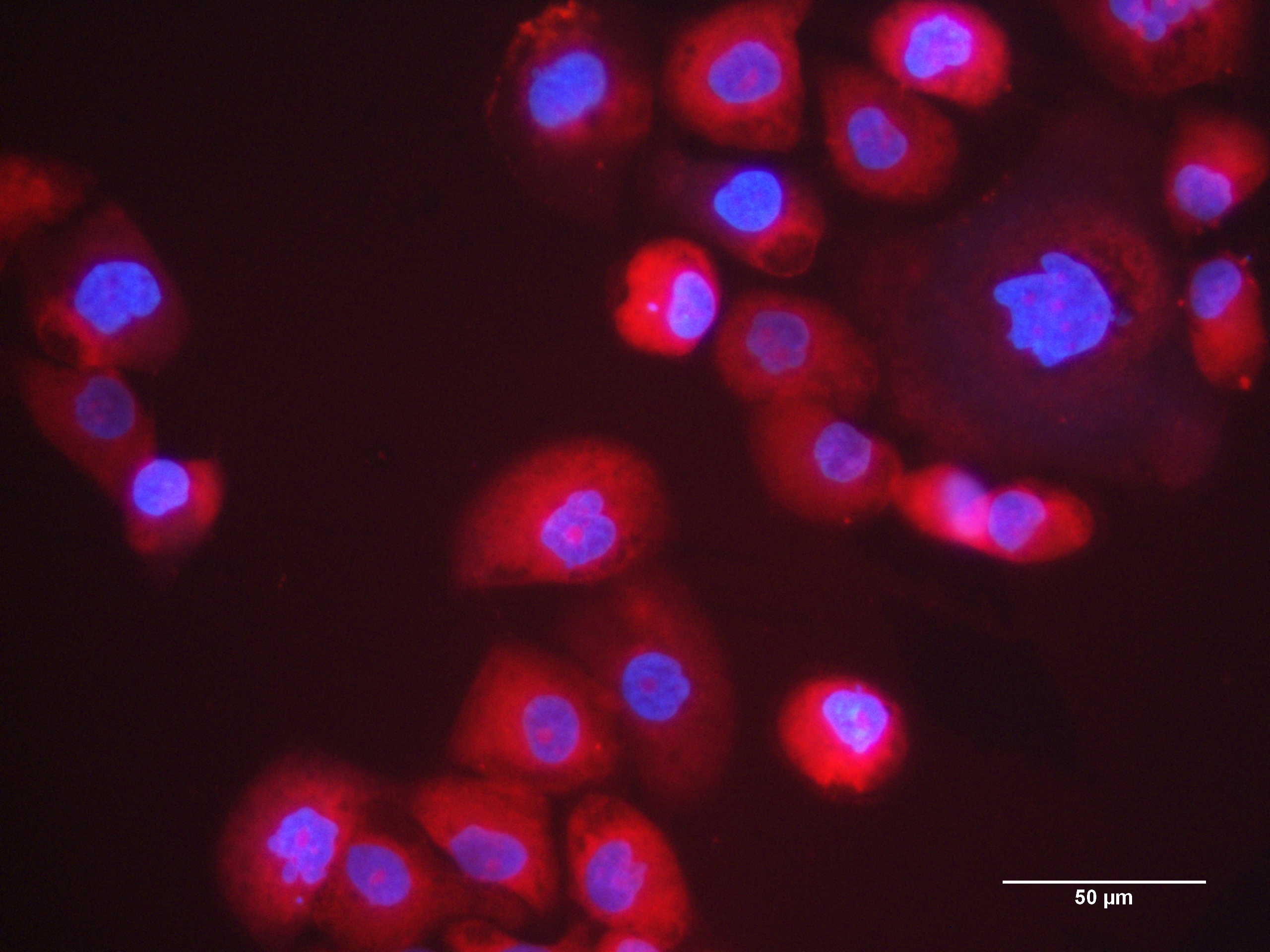

Supplement: Supplementary file 5 [file DataSheet6.zip › MitoSOX-2/MitoSOX═╝╞1⁄4/Iohexol 1 merge.tif]

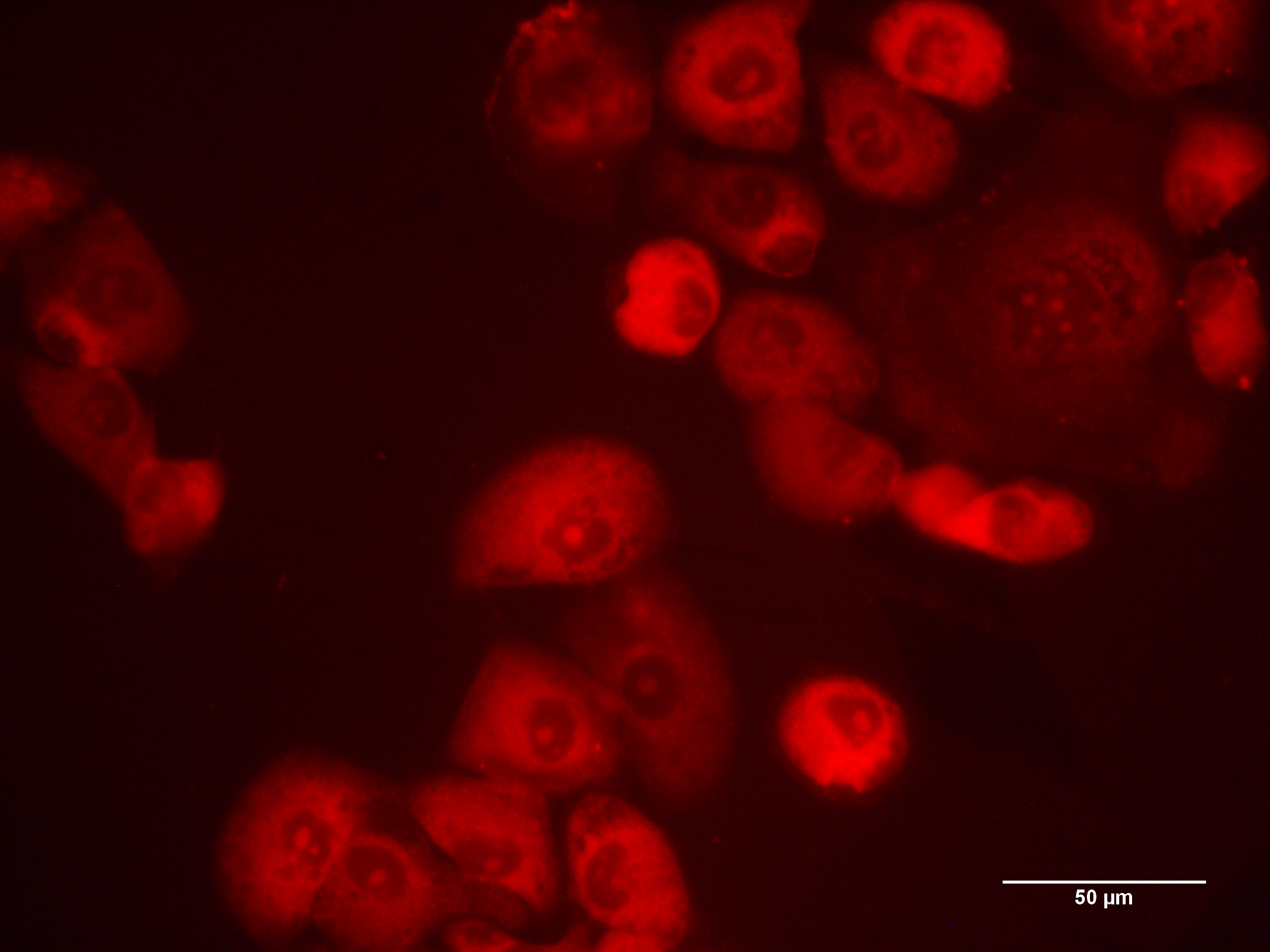

Supplement: Supplementary file 5 [file DataSheet6.zip › MitoSOX-2/MitoSOX═╝╞1⁄4/Iohexol 1 MitoSOX.tif]

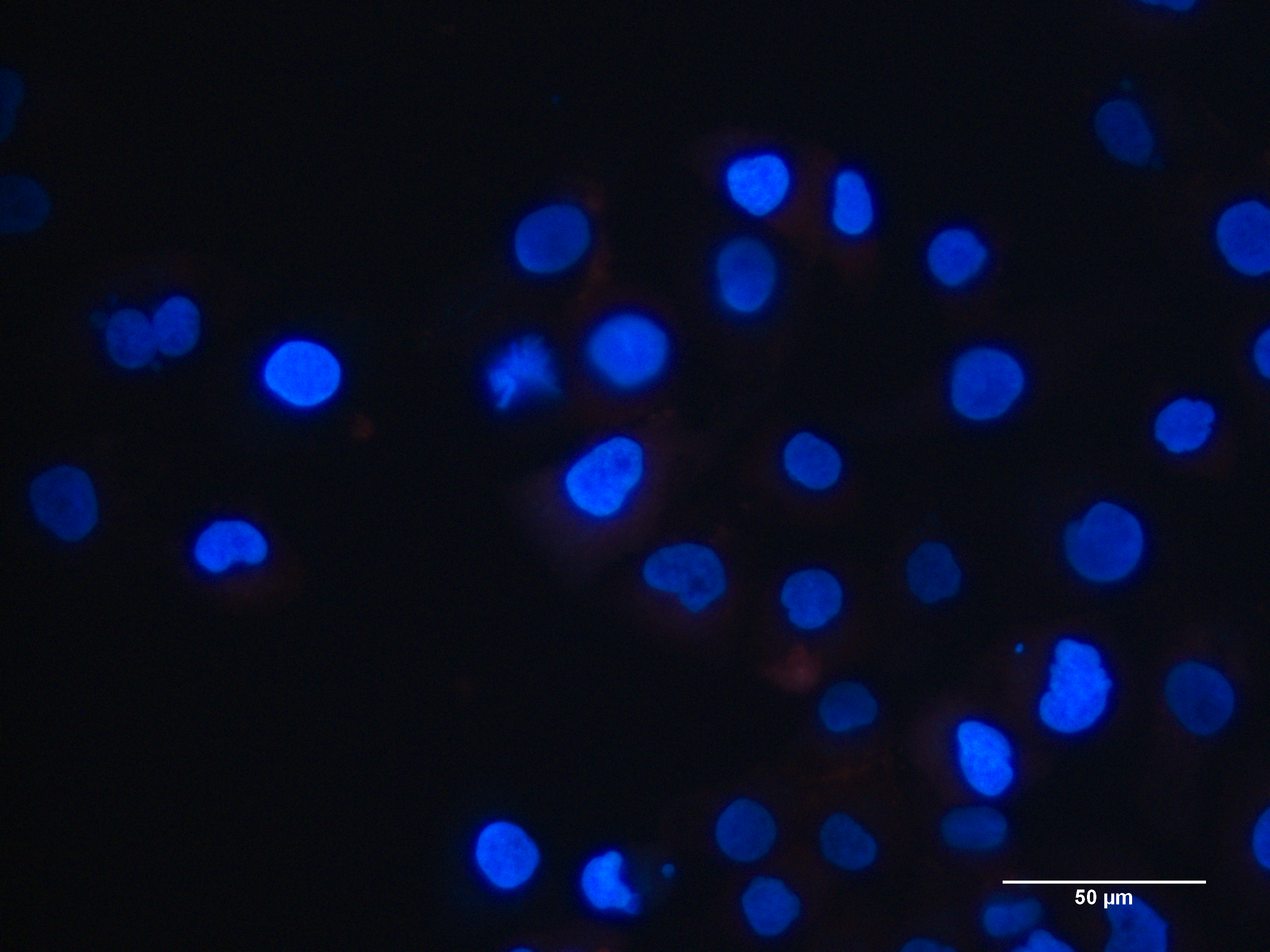

Supplement: Supplementary file 5 [file DataSheet6.zip › MitoSOX-2/MitoSOX═╝╞1⁄4/Iohexol 2 DAPI.tif]

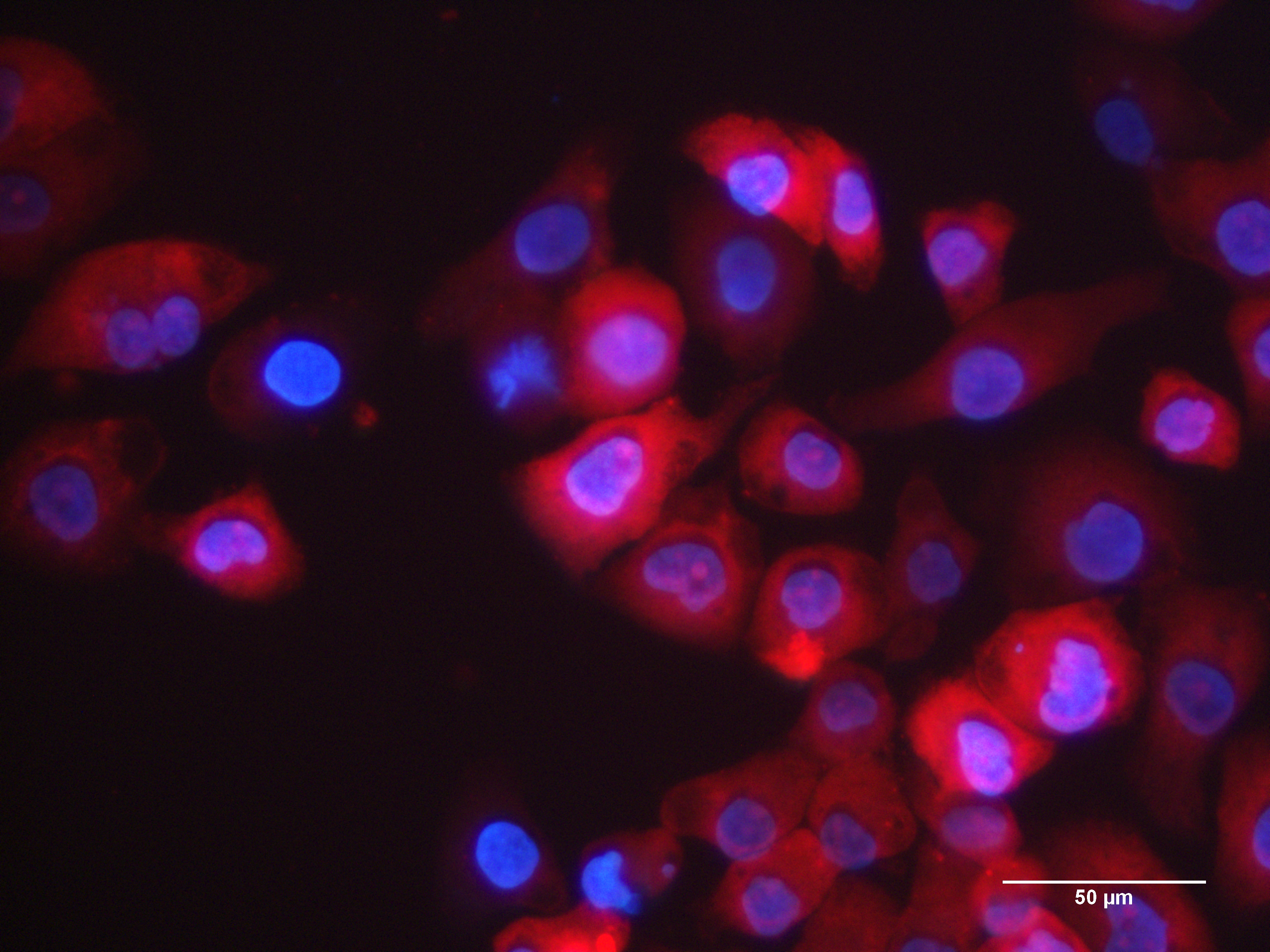

Supplement: Supplementary file 5 [file DataSheet6.zip › MitoSOX-2/MitoSOX═╝╞1⁄4/Iohexol 2 merge.tif]

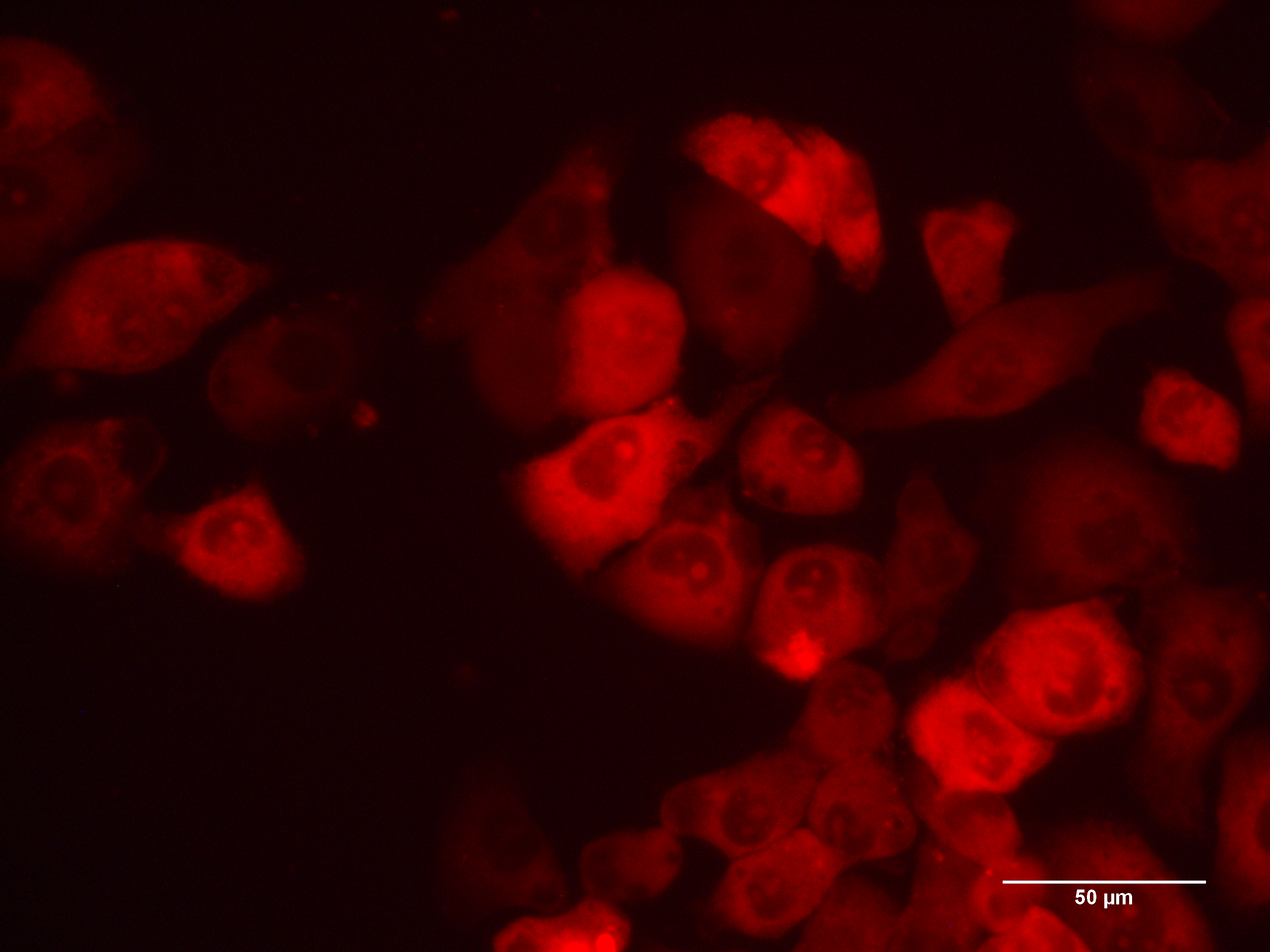

Supplement: Supplementary file 5 [file DataSheet6.zip › MitoSOX-2/MitoSOX═╝╞1⁄4/Iohexol 2 MitoSOX.tif]

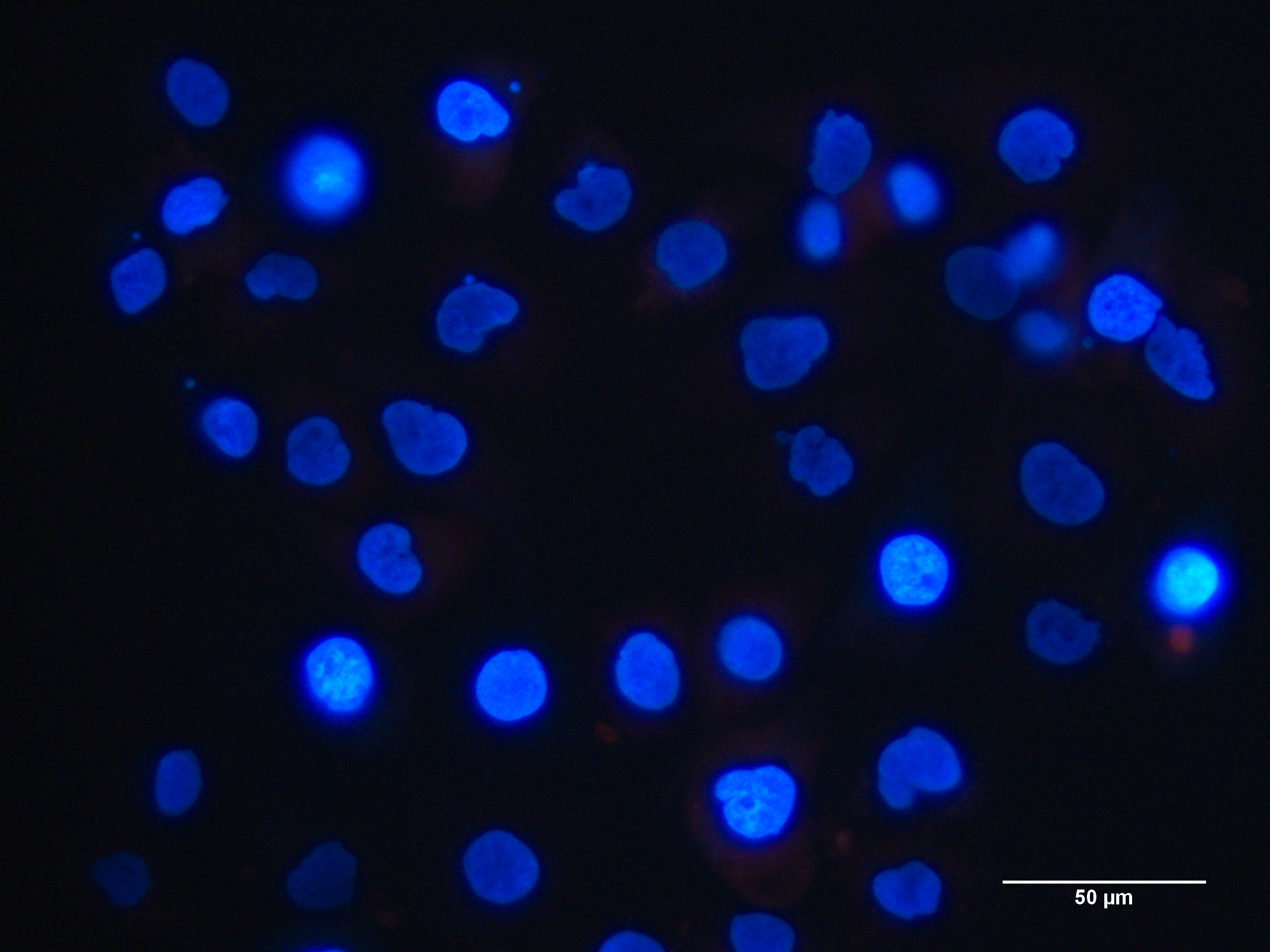

Supplement: Supplementary file 5 [file DataSheet6.zip › MitoSOX-2/MitoSOX═╝╞1⁄4/Iohexol 3 DAPI.tif]

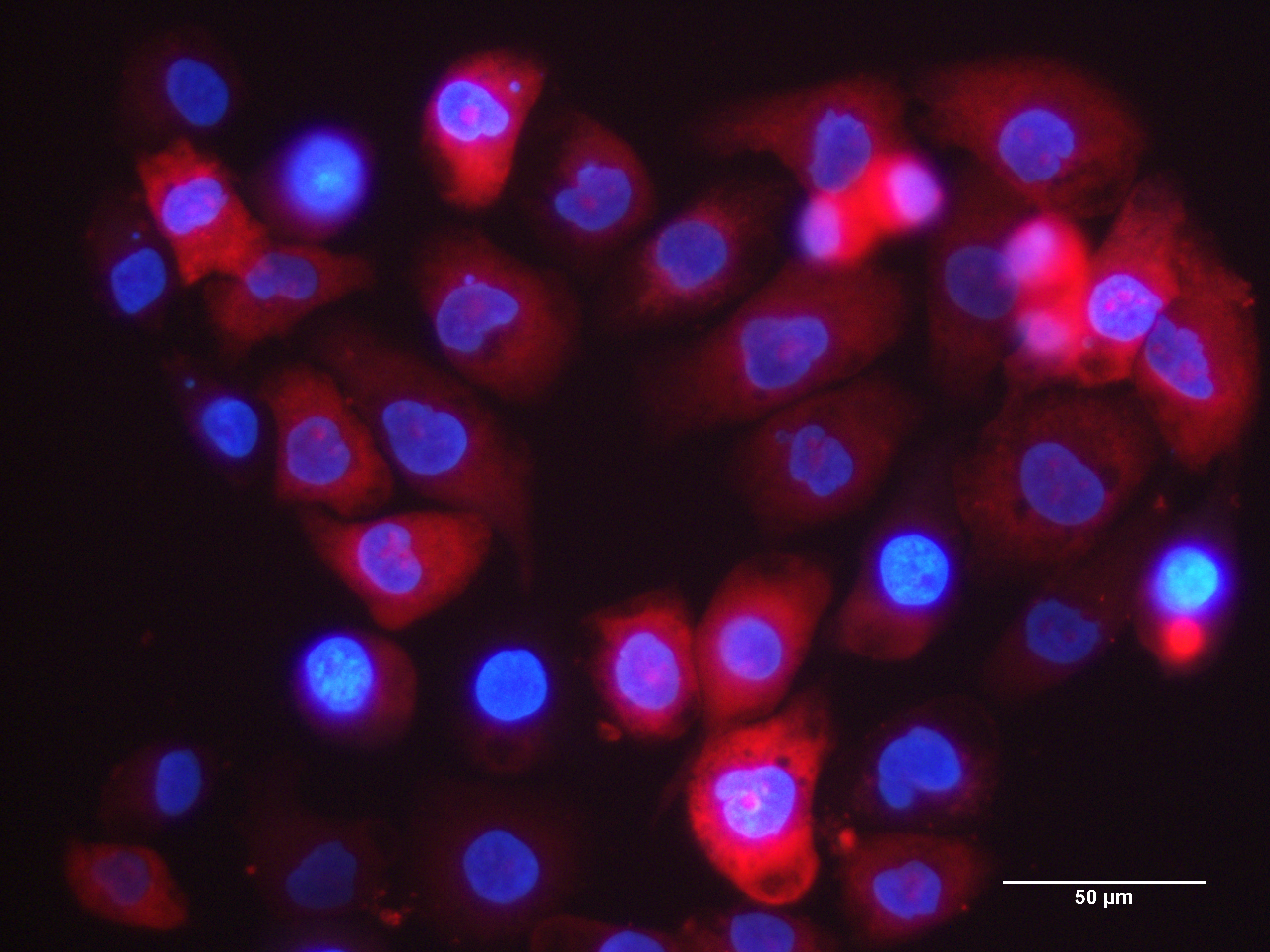

Supplement: Supplementary file 5 [file DataSheet6.zip › MitoSOX-2/MitoSOX═╝╞1⁄4/Iohexol 3 merge.tif]

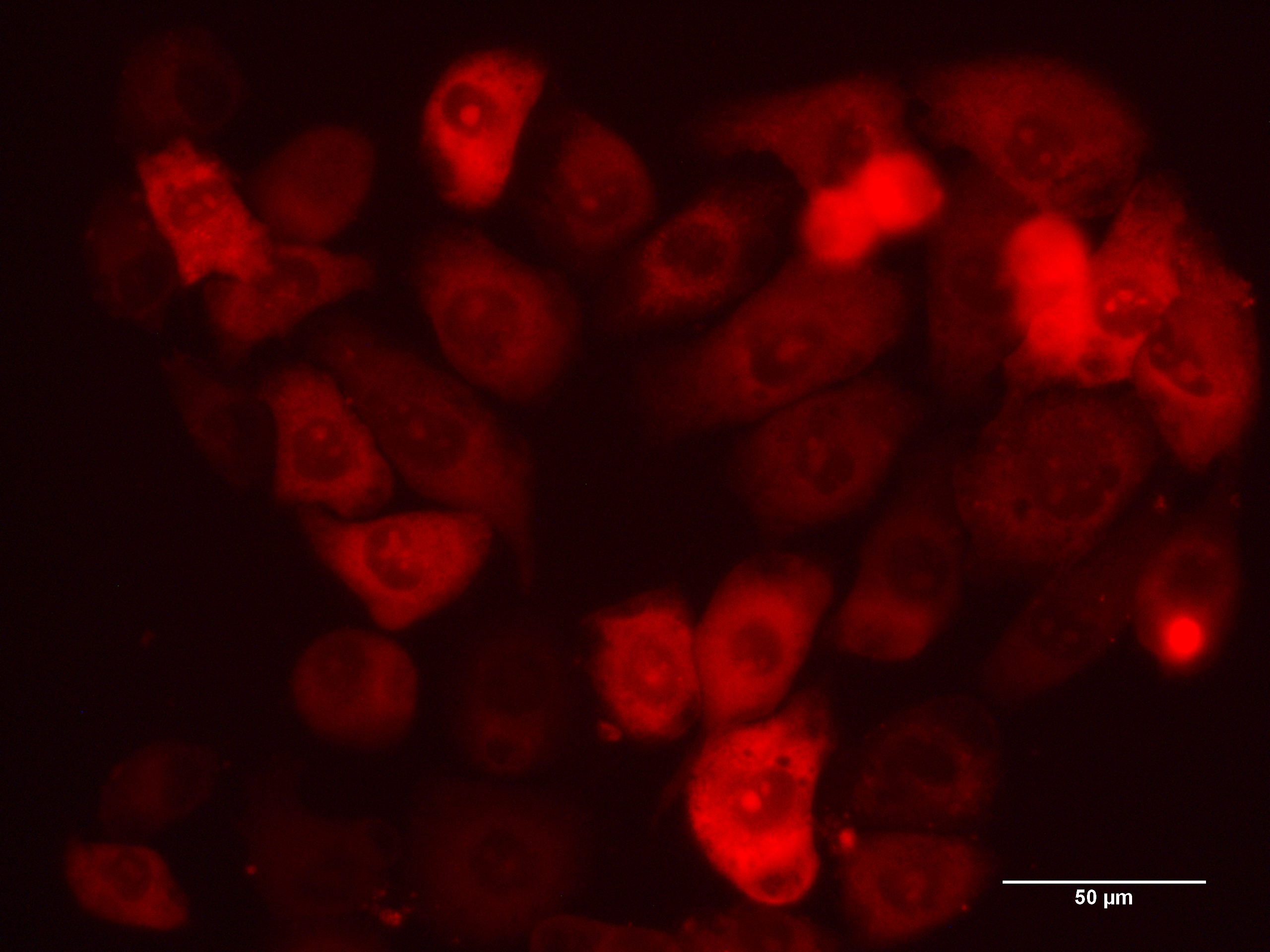

Supplement: Supplementary file 5 [file DataSheet6.zip › MitoSOX-2/MitoSOX═╝╞1⁄4/Iohexol 3 MitoSOX.tif]

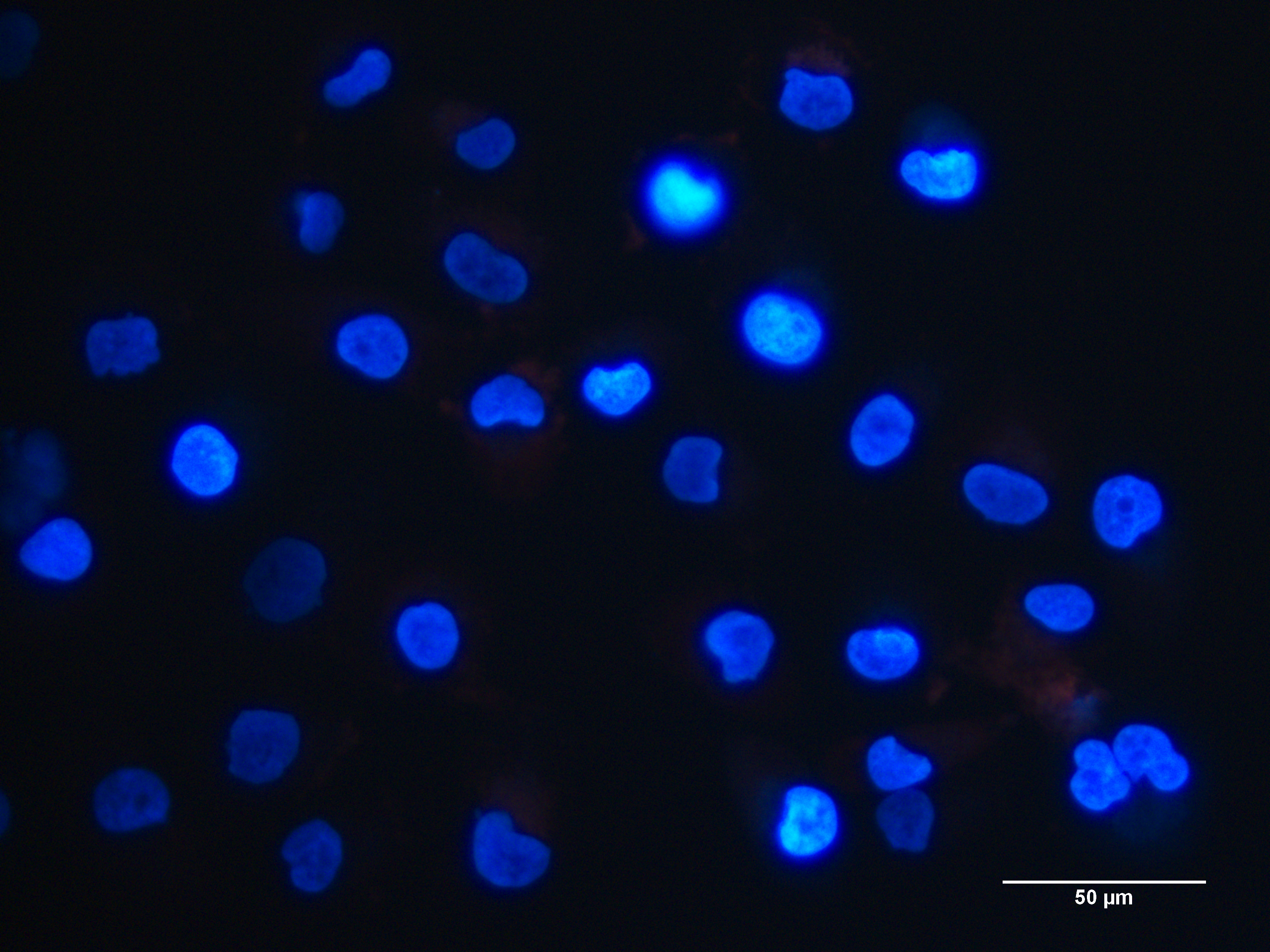

Supplement: Supplementary file 5 [file DataSheet6.zip › MitoSOX-2/MitoSOX═╝╞1⁄4/RU360_Iohexol 1 DAPI.tif]

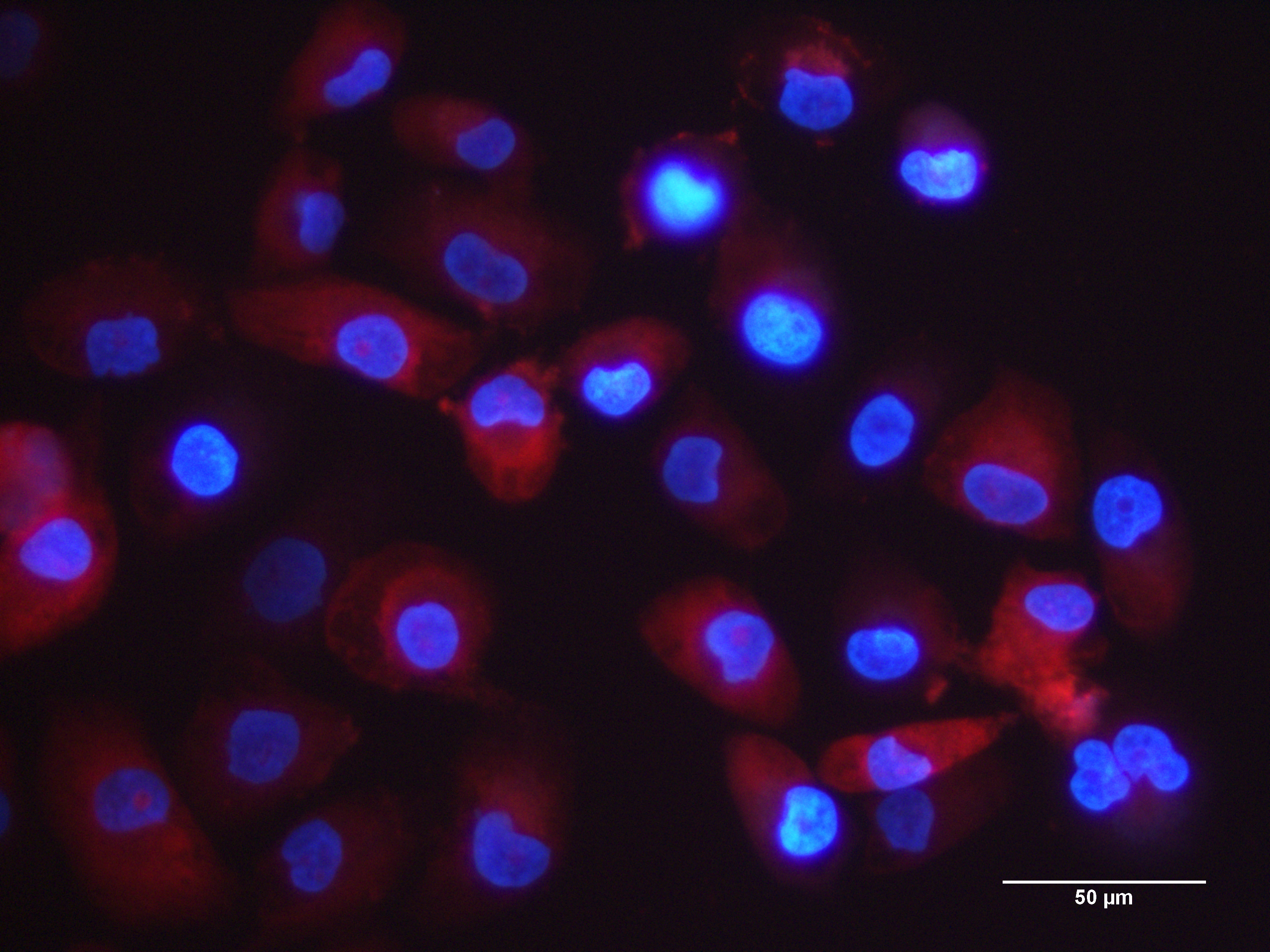

Supplement: Supplementary file 5 [file DataSheet6.zip › MitoSOX-2/MitoSOX═╝╞1⁄4/RU360_Iohexol 1 merge.tif]

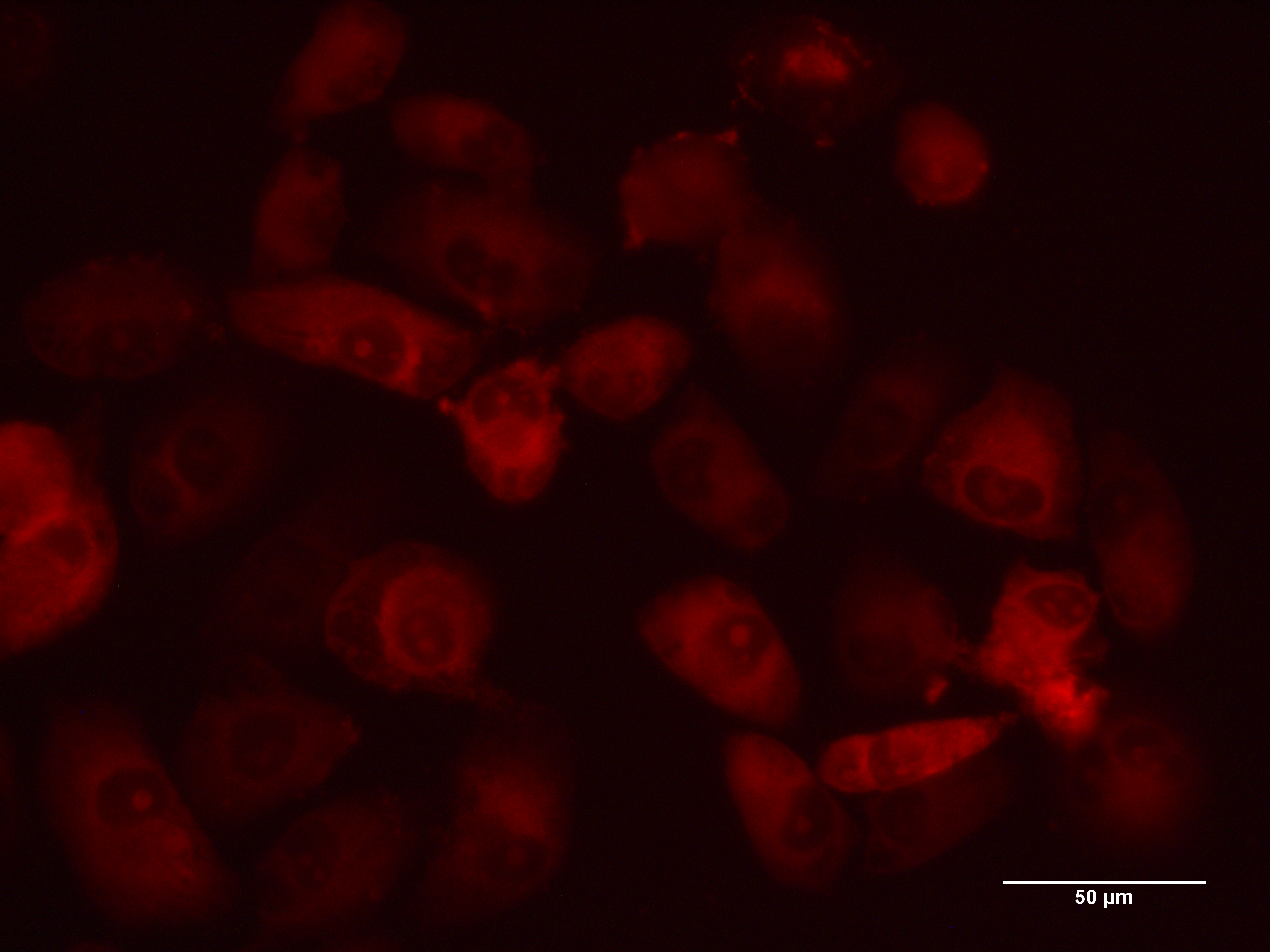

Supplement: Supplementary file 5 [file DataSheet6.zip › MitoSOX-2/MitoSOX═╝╞1⁄4/RU360_Iohexol 1 MitoSOX.tif]

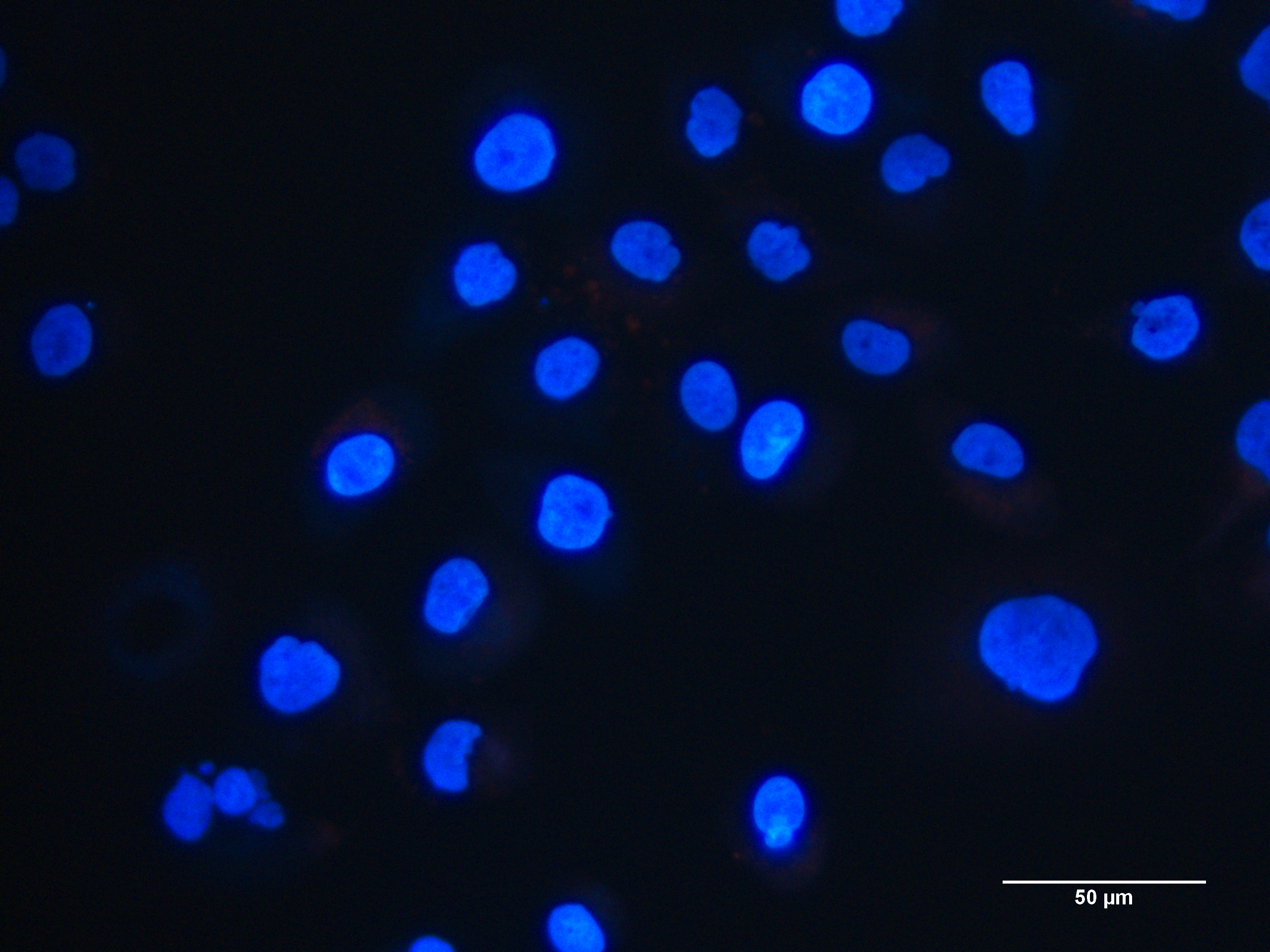

Supplement: Supplementary file 5 [file DataSheet6.zip › MitoSOX-2/MitoSOX═╝╞1⁄4/RU360_Iohexol 2 DAPI.tif]

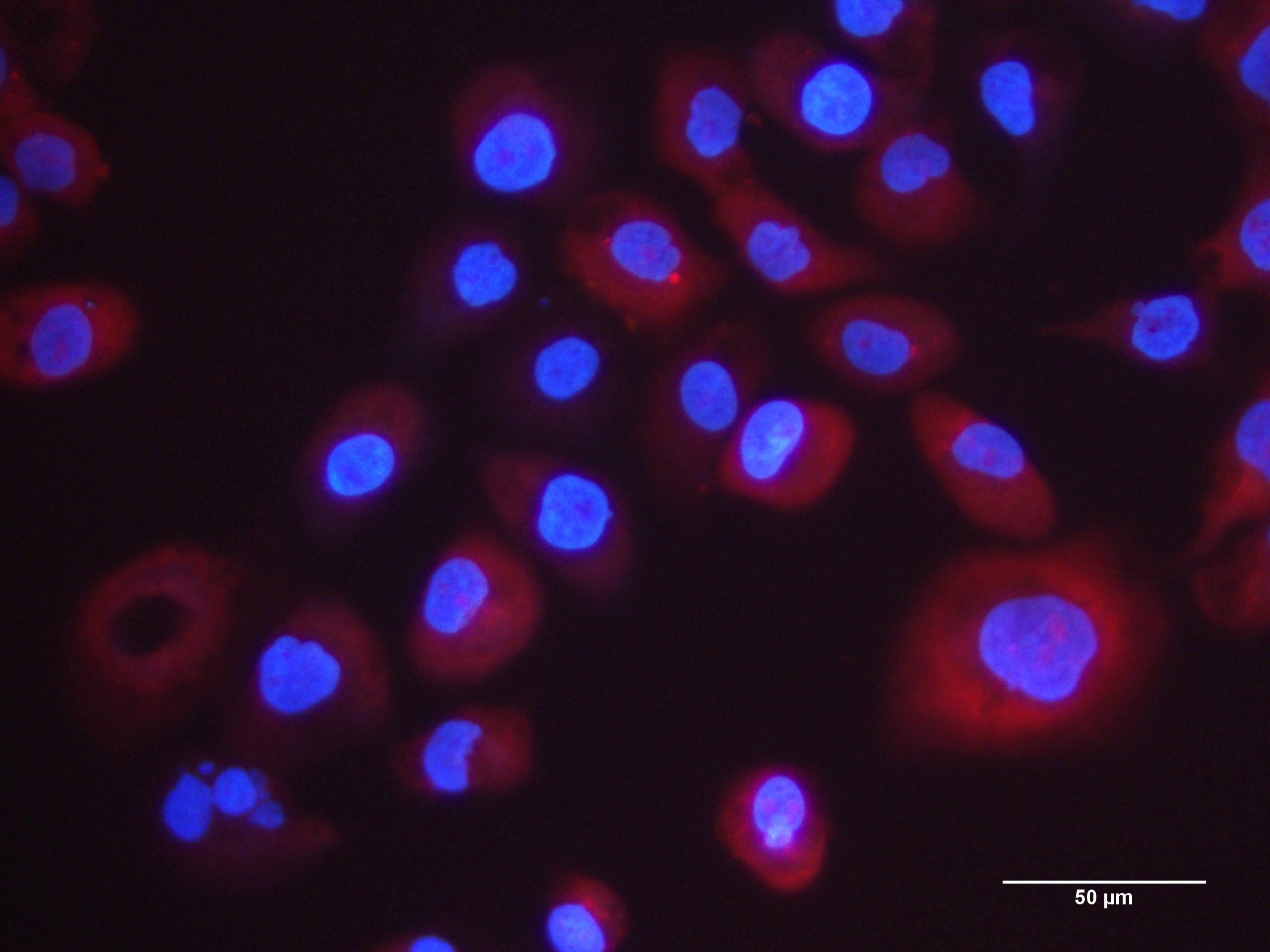

Supplement: Supplementary file 5 [file DataSheet6.zip › MitoSOX-2/MitoSOX═╝╞1⁄4/RU360_Iohexol 2 merge.tif]

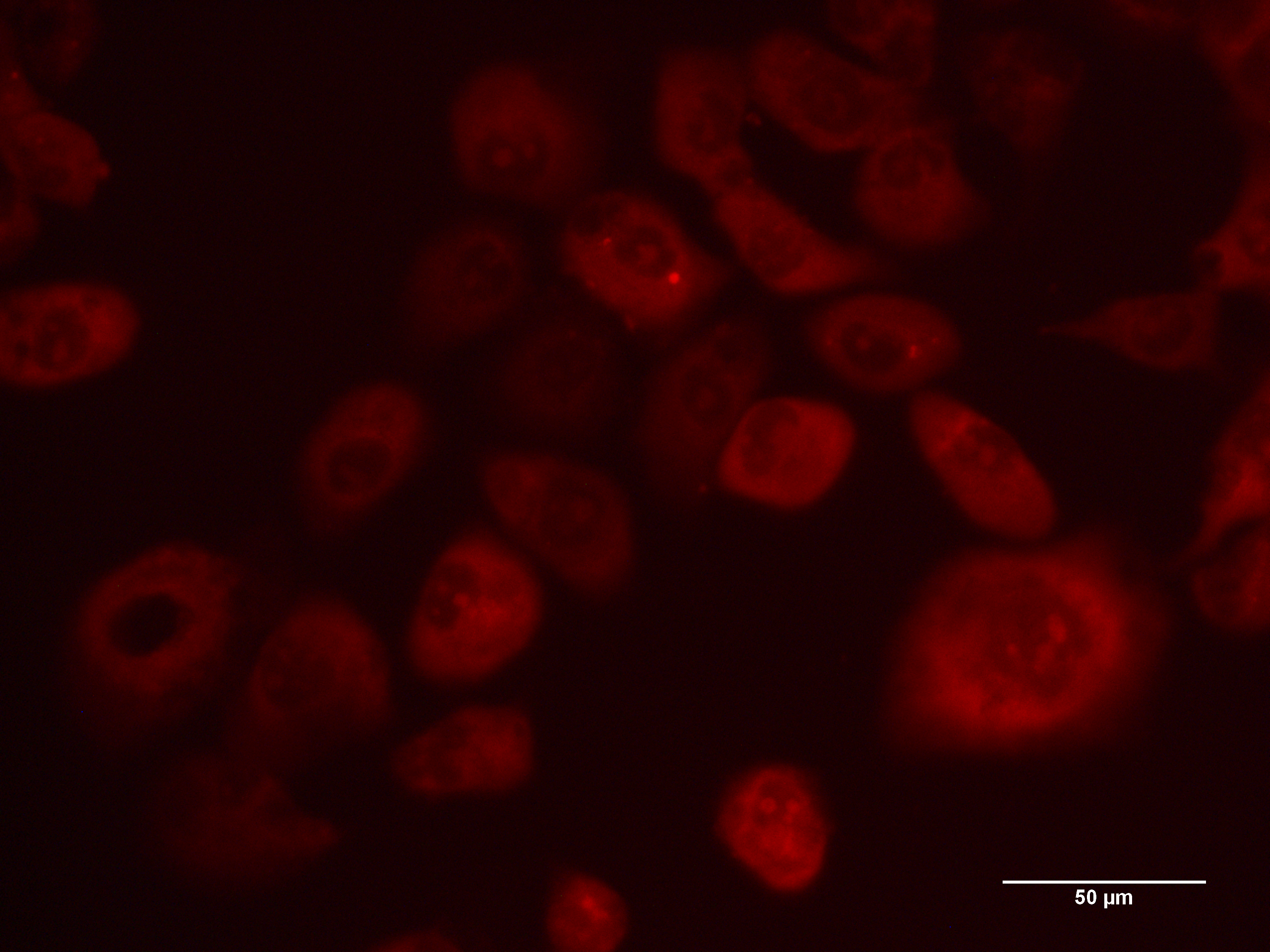

Supplement: Supplementary file 5 [file DataSheet6.zip › MitoSOX-2/MitoSOX═╝╞1⁄4/RU360_Iohexol 2 MitoSOX.tif]

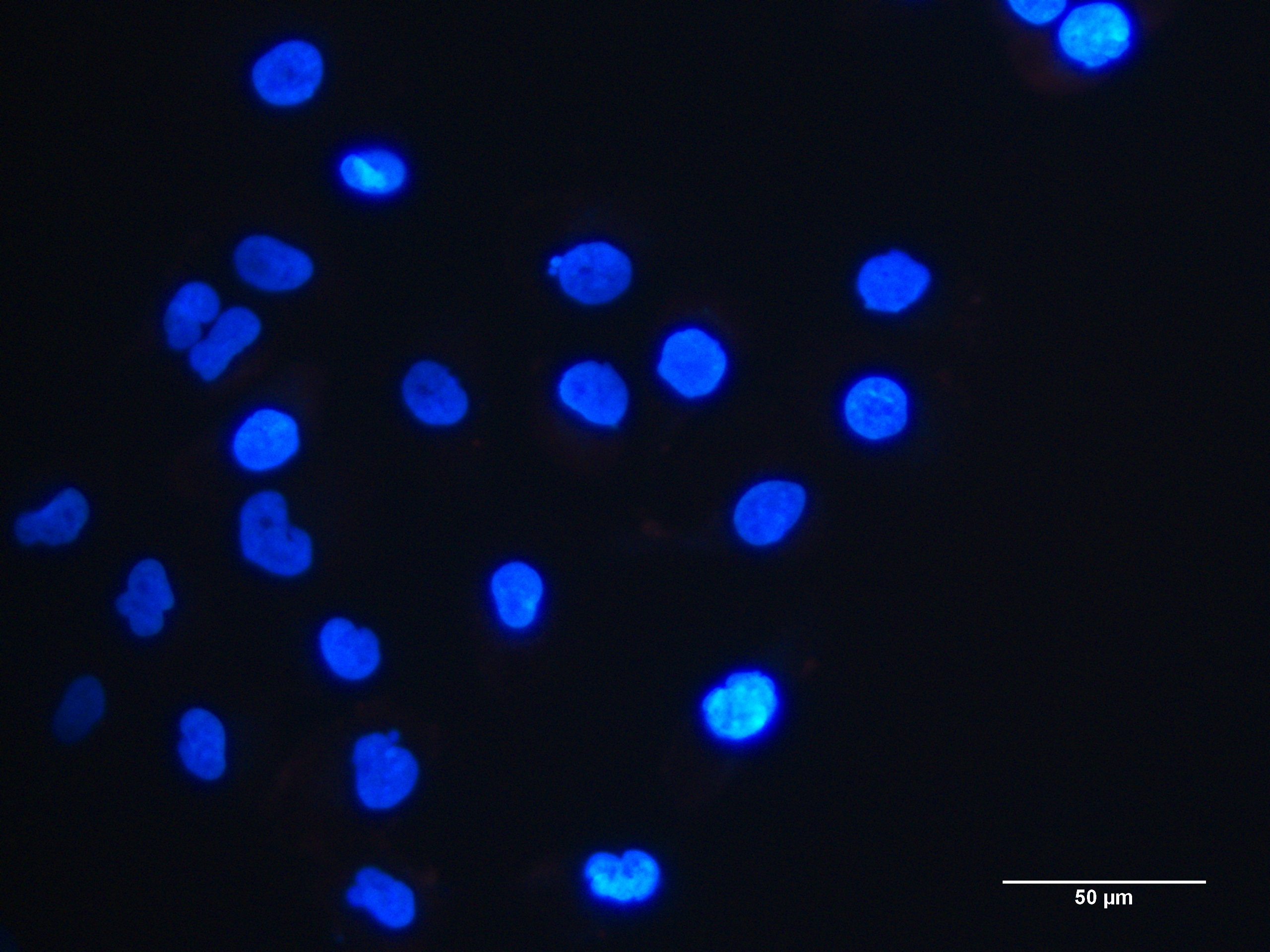

Supplement: Supplementary file 5 [file DataSheet6.zip › MitoSOX-2/MitoSOX═╝╞1⁄4/RU360_Iohexol 3 DAPI.tif]

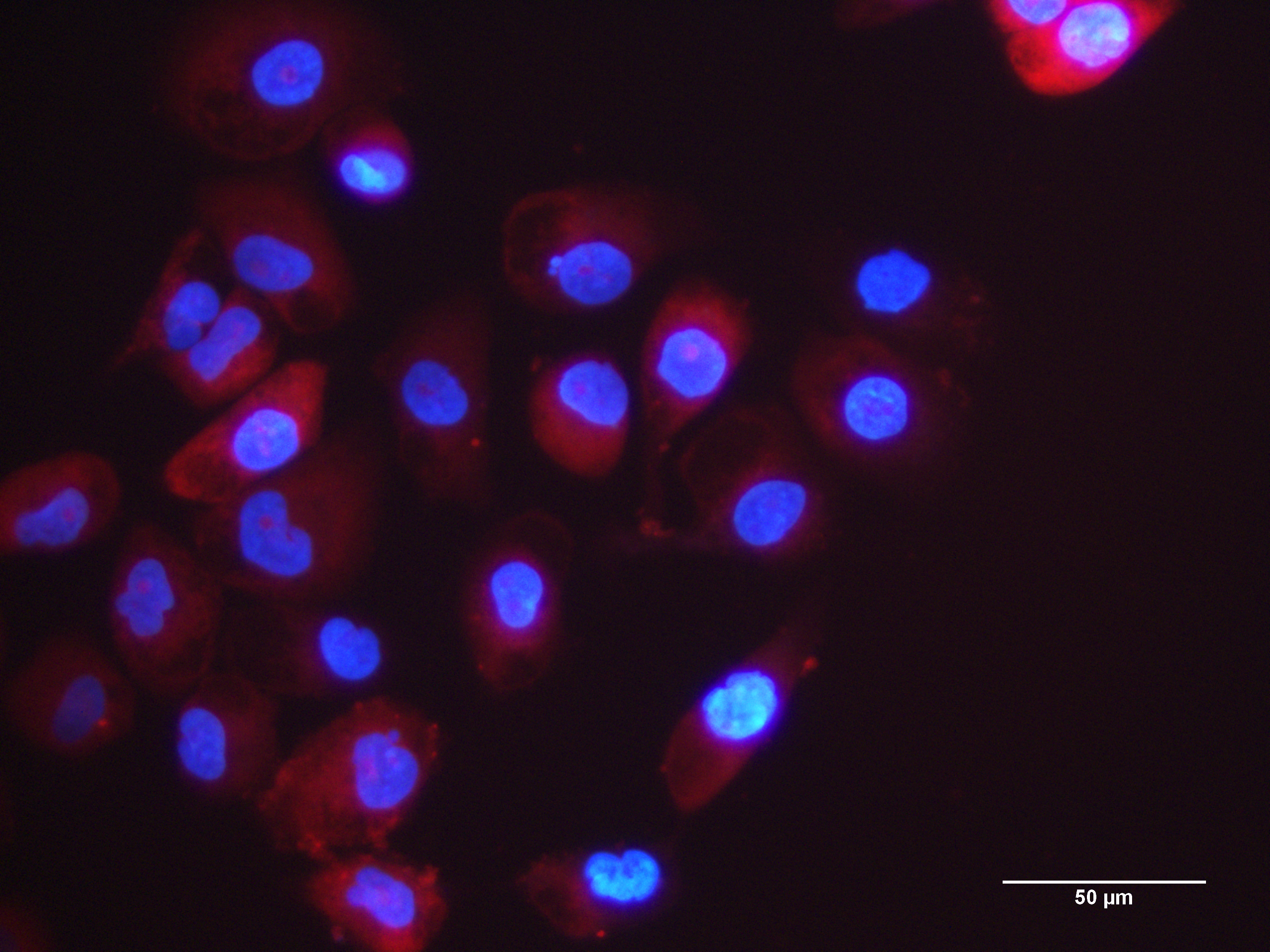

Supplement: Supplementary file 5 [file DataSheet6.zip › MitoSOX-2/MitoSOX═╝╞1⁄4/RU360_Iohexol 3 merge.tif]

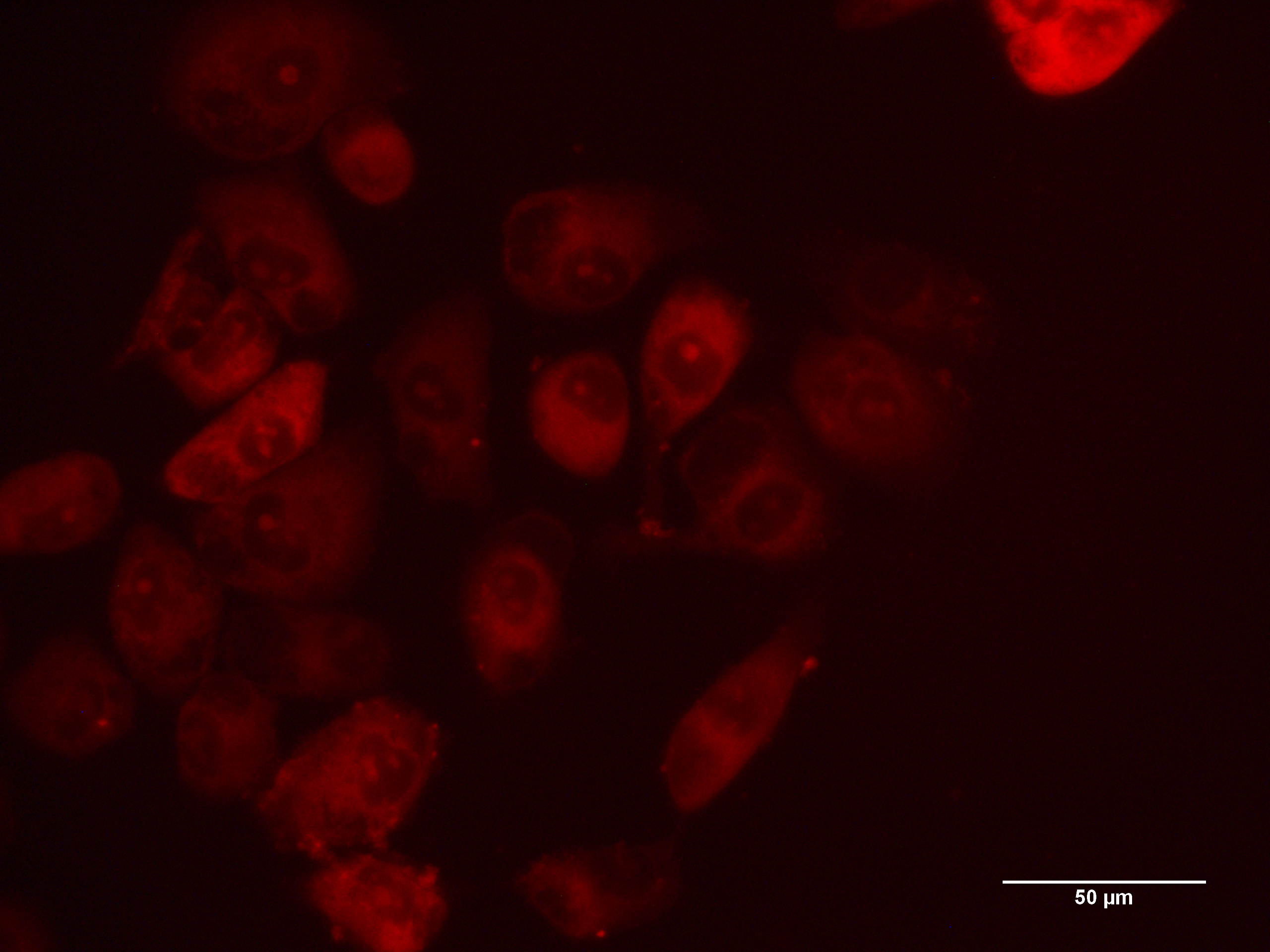

Supplement: Supplementary file 5 [file DataSheet6.zip › MitoSOX-2/MitoSOX═╝╞1⁄4/RU360_Iohexol 3 MitoSOX.tif]

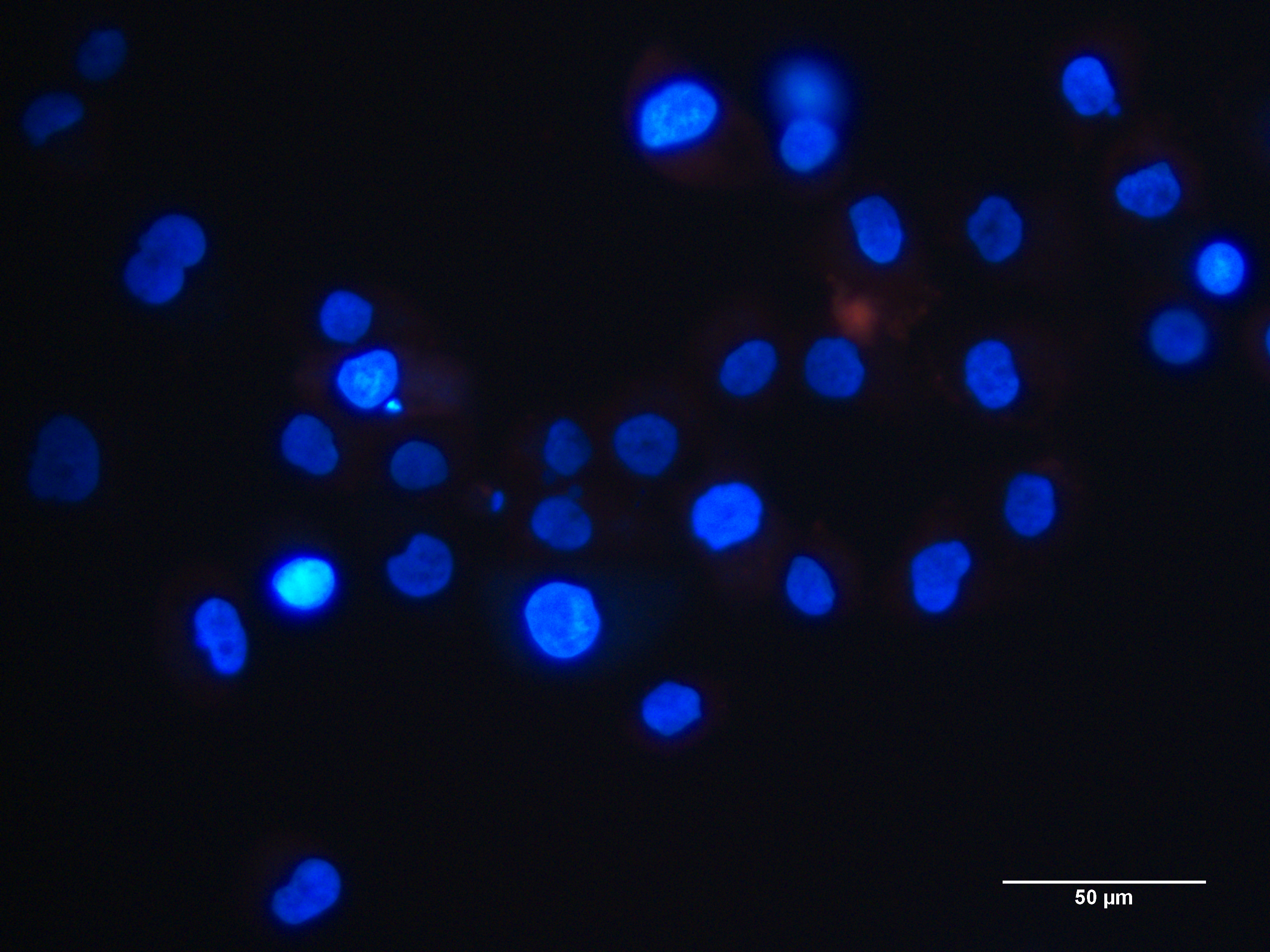

Supplement: Supplementary file 5 [file DataSheet6.zip › MitoSOX-2/MitoSOX═╝╞1⁄4/Spermine_Iohexol 1 DAPI.tif]

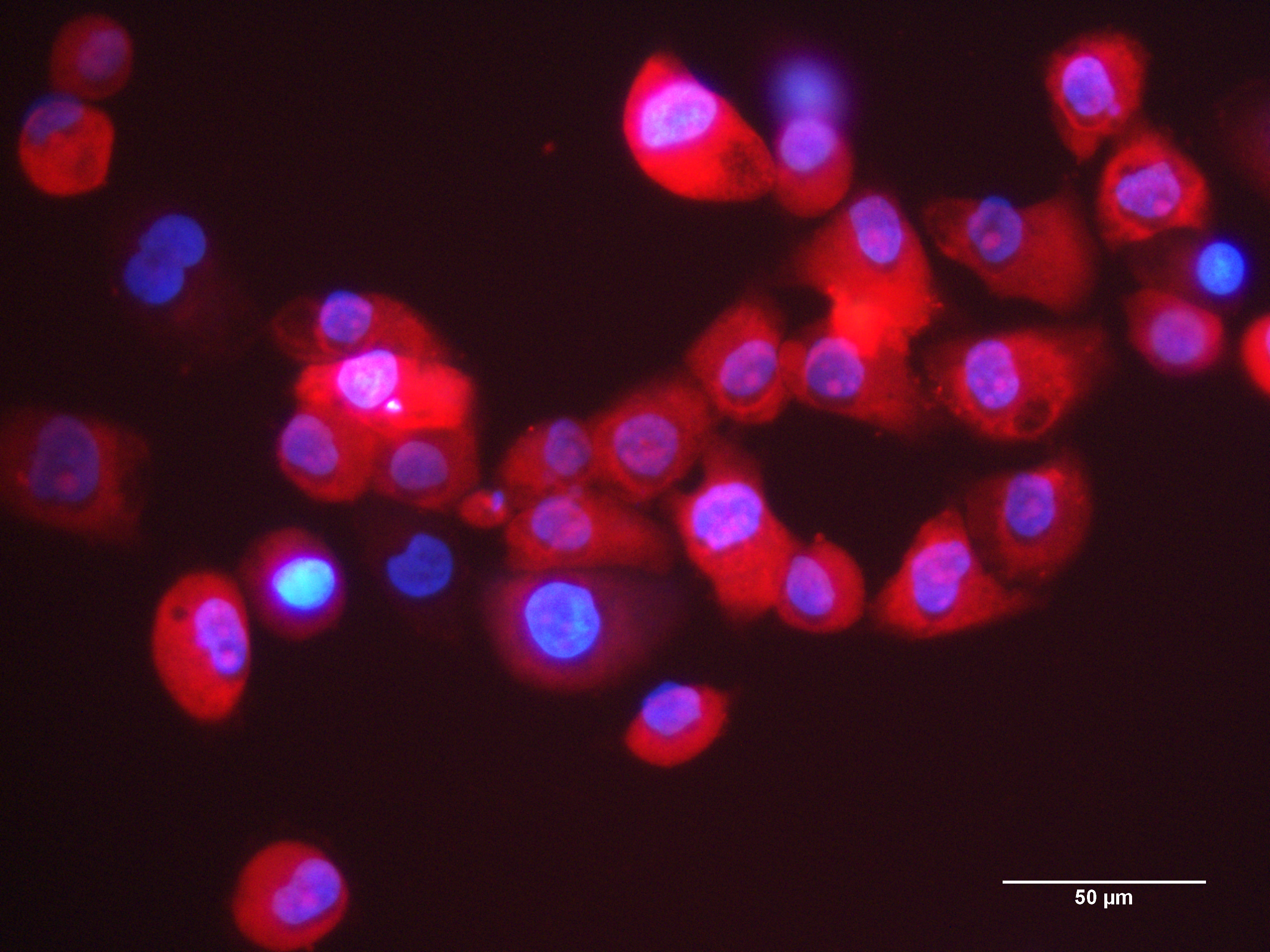

Supplement: Supplementary file 5 [file DataSheet6.zip › MitoSOX-2/MitoSOX═╝╞1⁄4/Spermine_Iohexol 1 merge.tif]

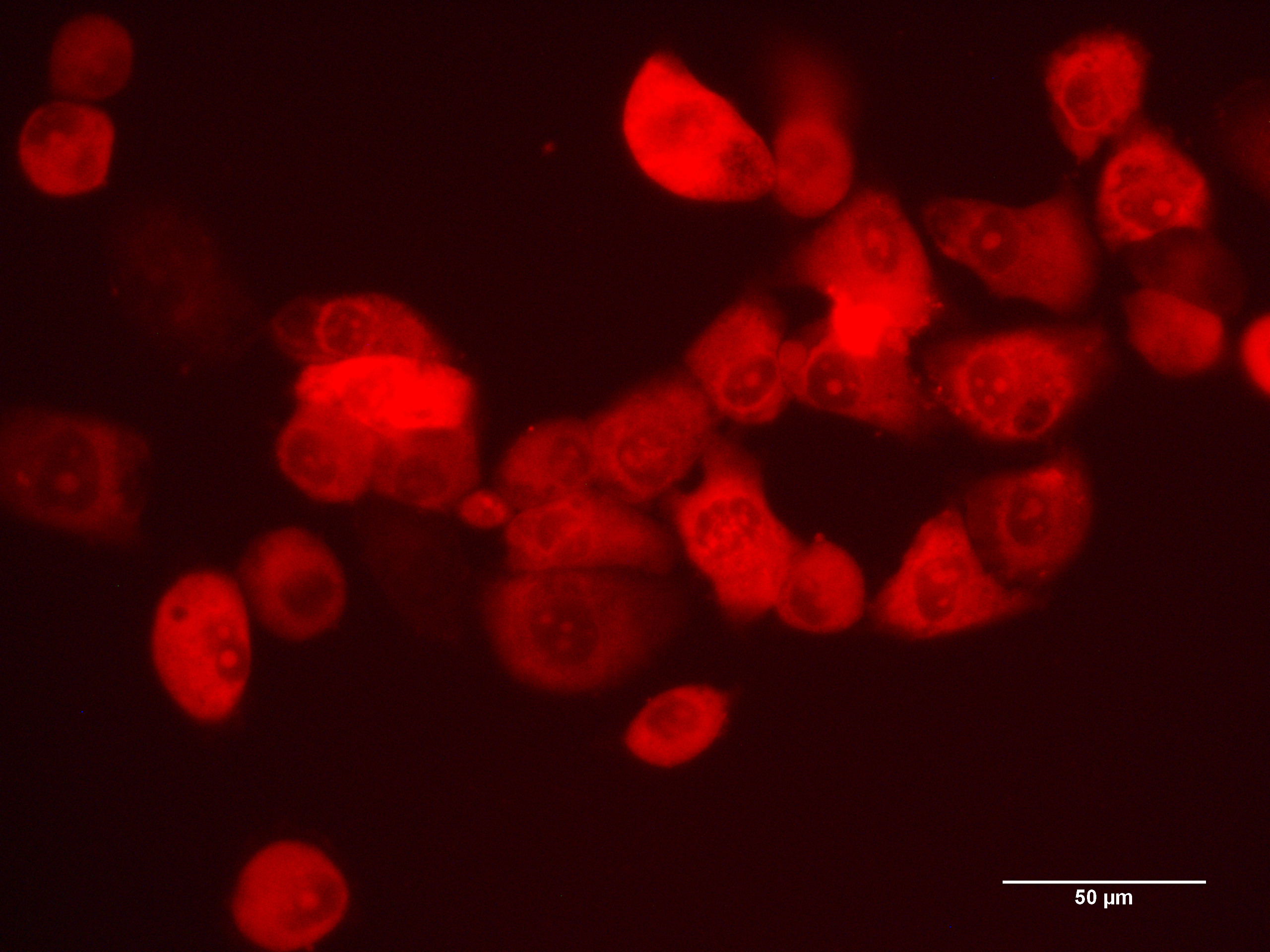

Supplement: Supplementary file 5 [file DataSheet6.zip › MitoSOX-2/MitoSOX═╝╞1⁄4/Spermine_Iohexol 1 MitoSOX.tif]

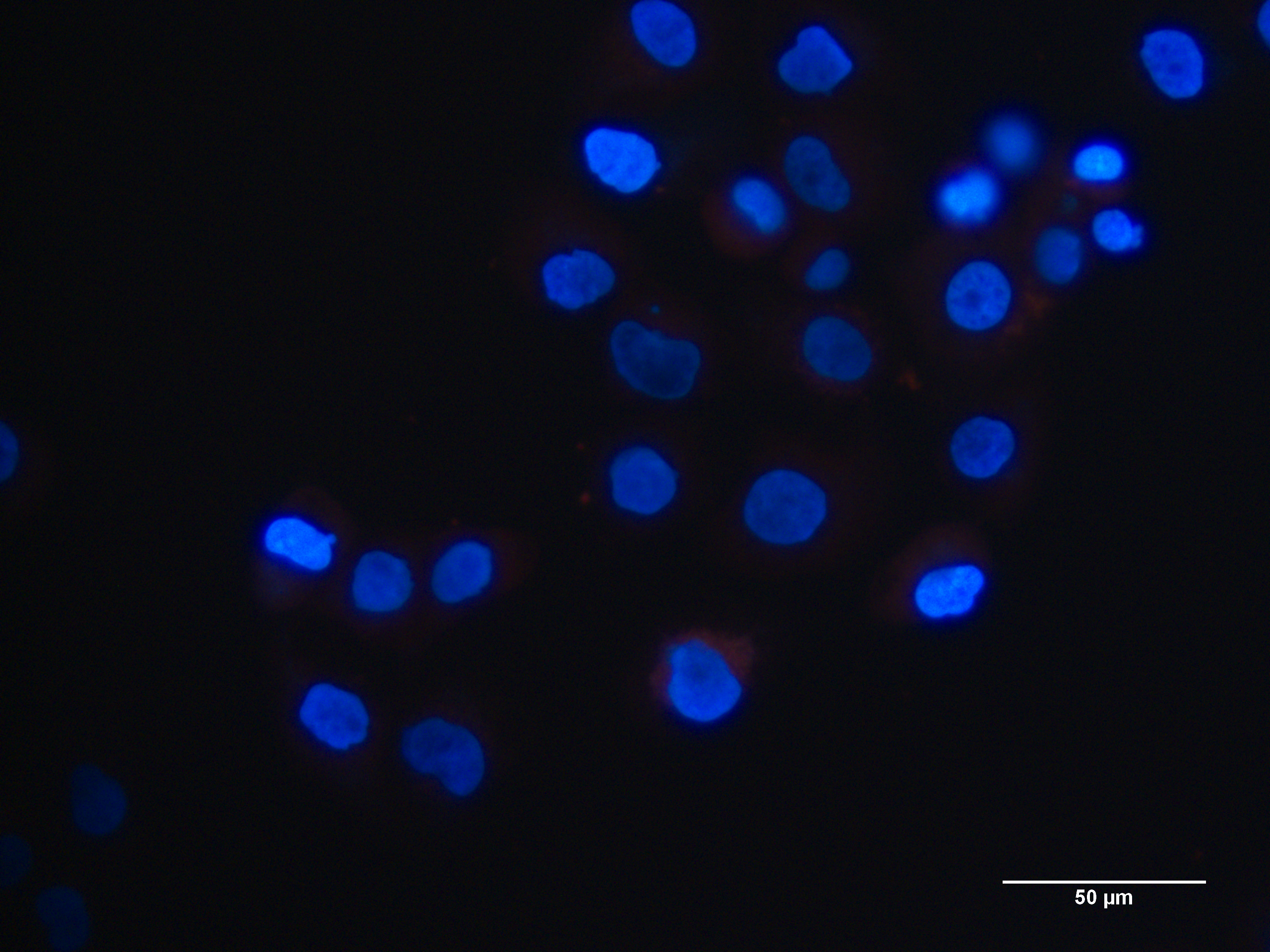

Supplement: Supplementary file 5 [file DataSheet6.zip › MitoSOX-2/MitoSOX═╝╞1⁄4/Spermine_Iohexol 2 DAPI.tif]

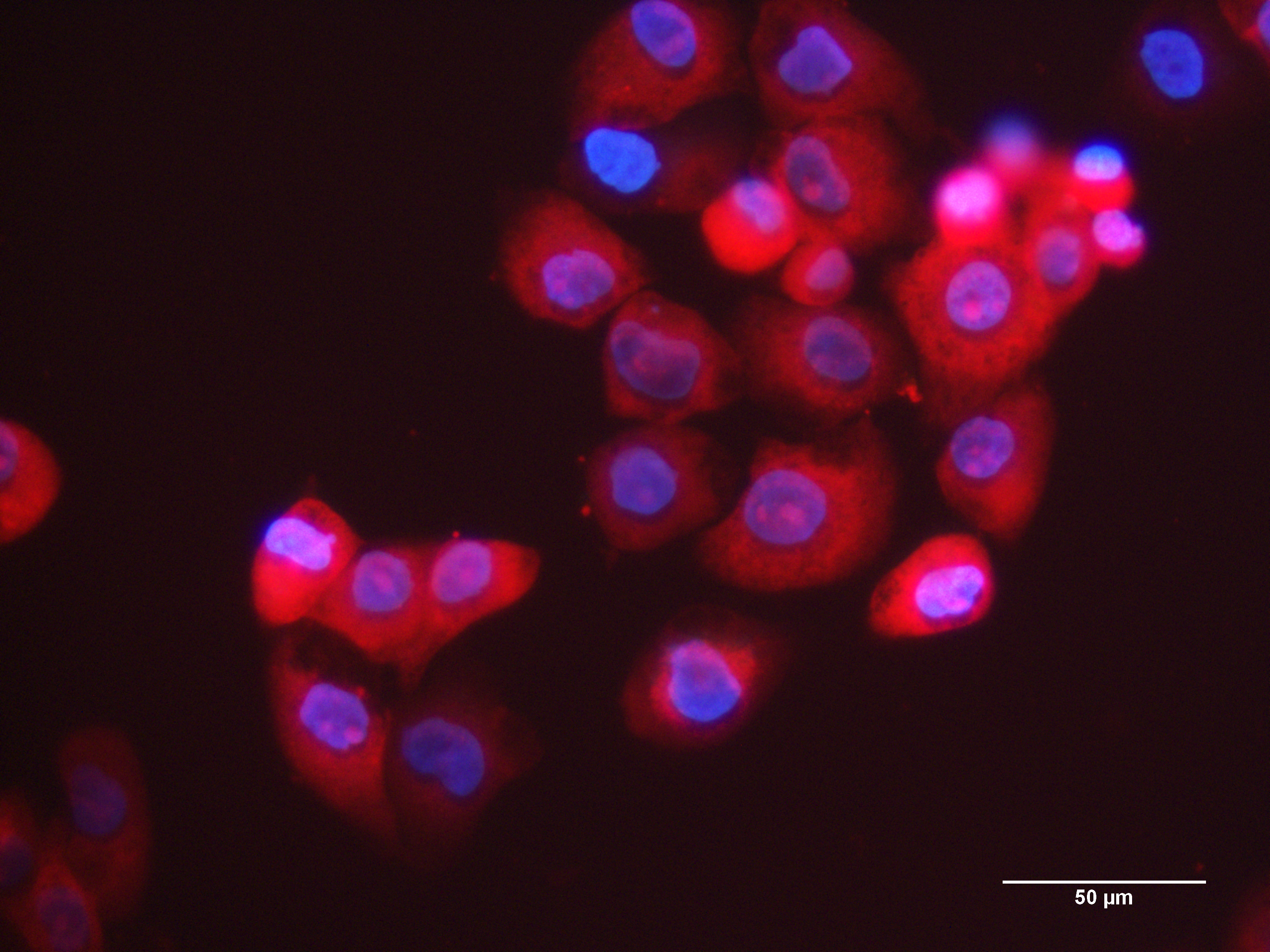

Supplement: Supplementary file 5 [file DataSheet6.zip › MitoSOX-2/MitoSOX═╝╞1⁄4/Spermine_Iohexol 2 merge.tif]

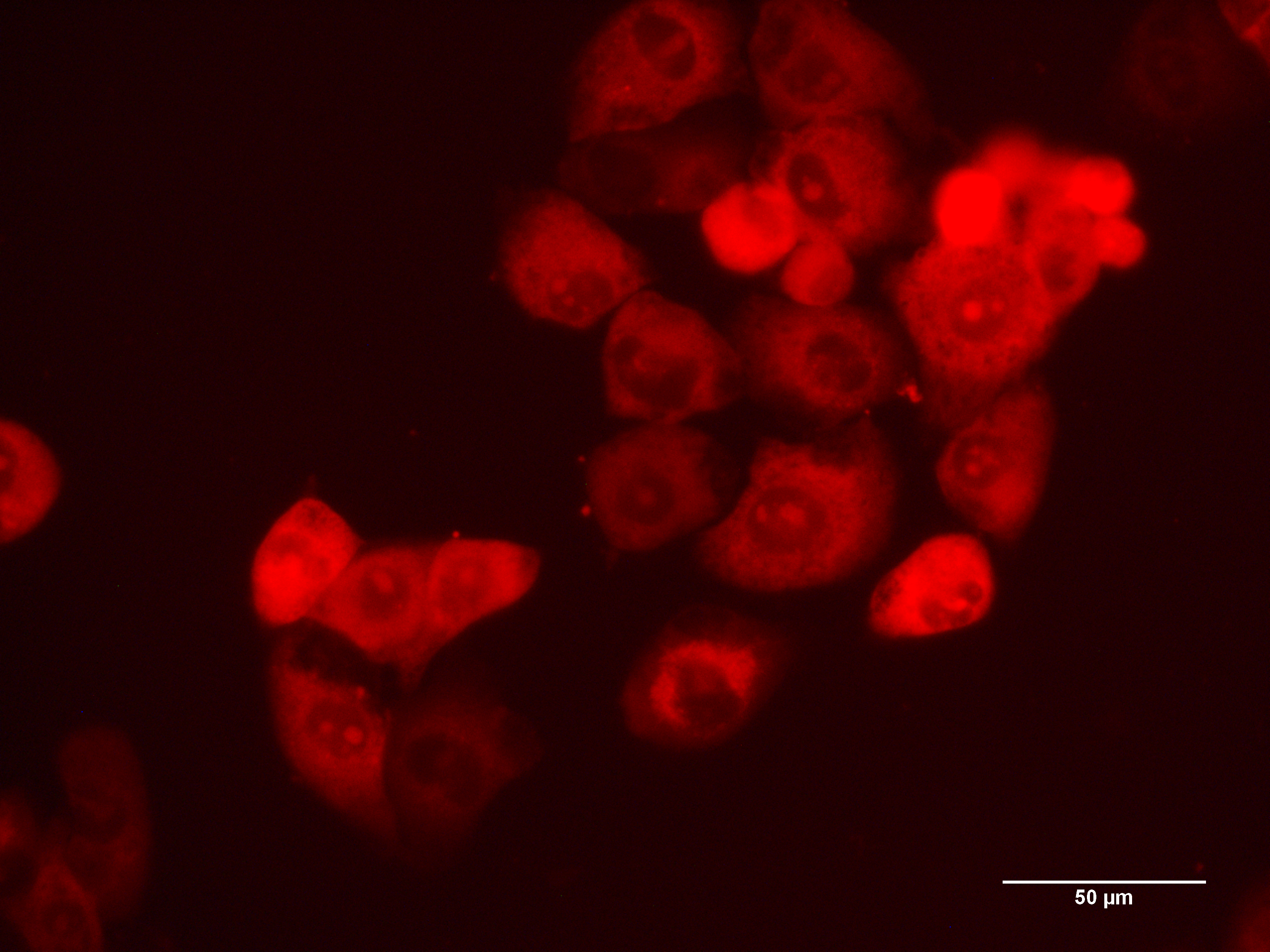

Supplement: Supplementary file 5 [file DataSheet6.zip › MitoSOX-2/MitoSOX═╝╞1⁄4/Spermine_Iohexol 2 MitoSOX.tif]

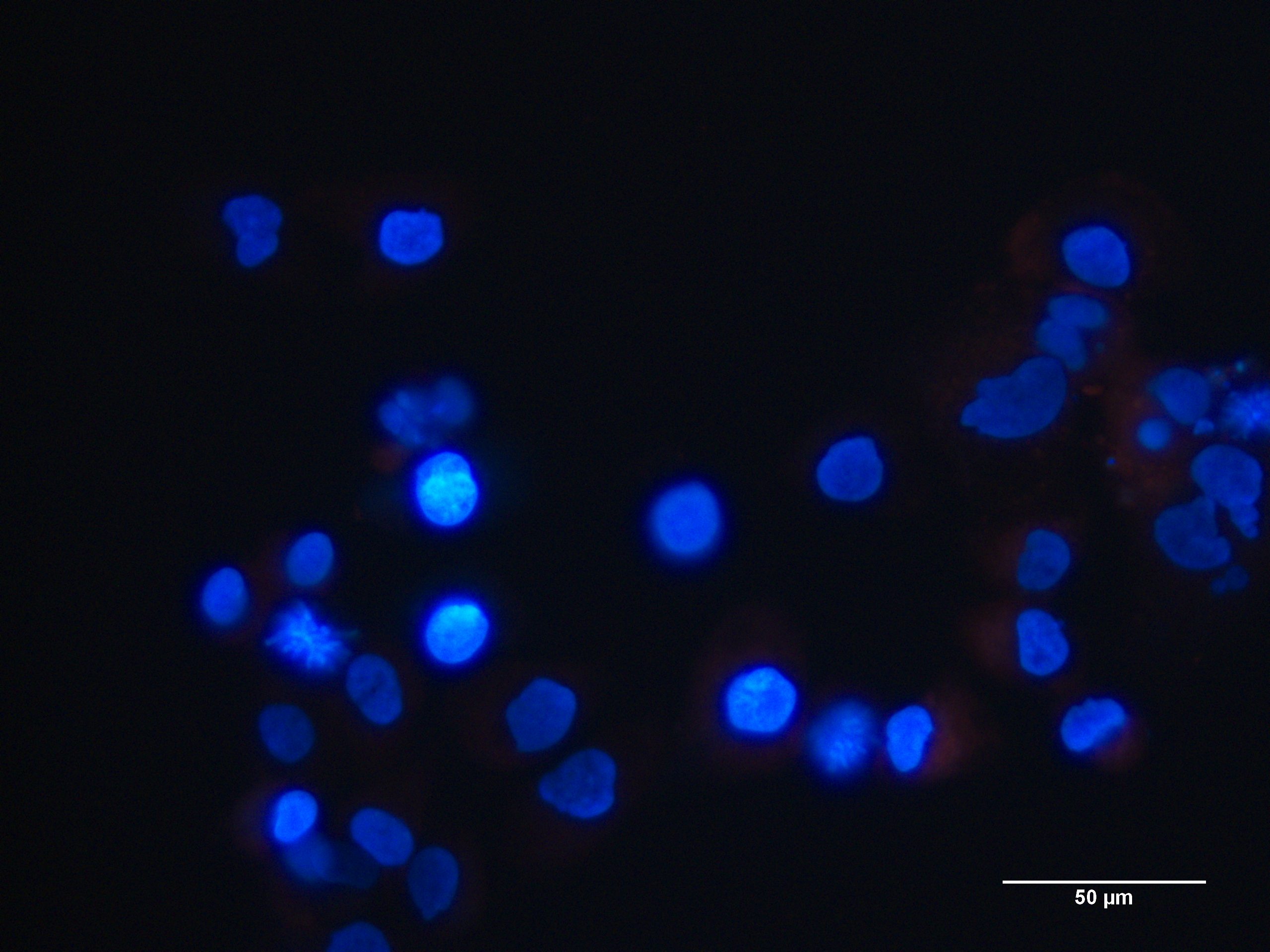

Supplement: Supplementary file 5 [file DataSheet6.zip › MitoSOX-2/MitoSOX═╝╞1⁄4/Spermine_Iohexol 3 DAPI.tif]

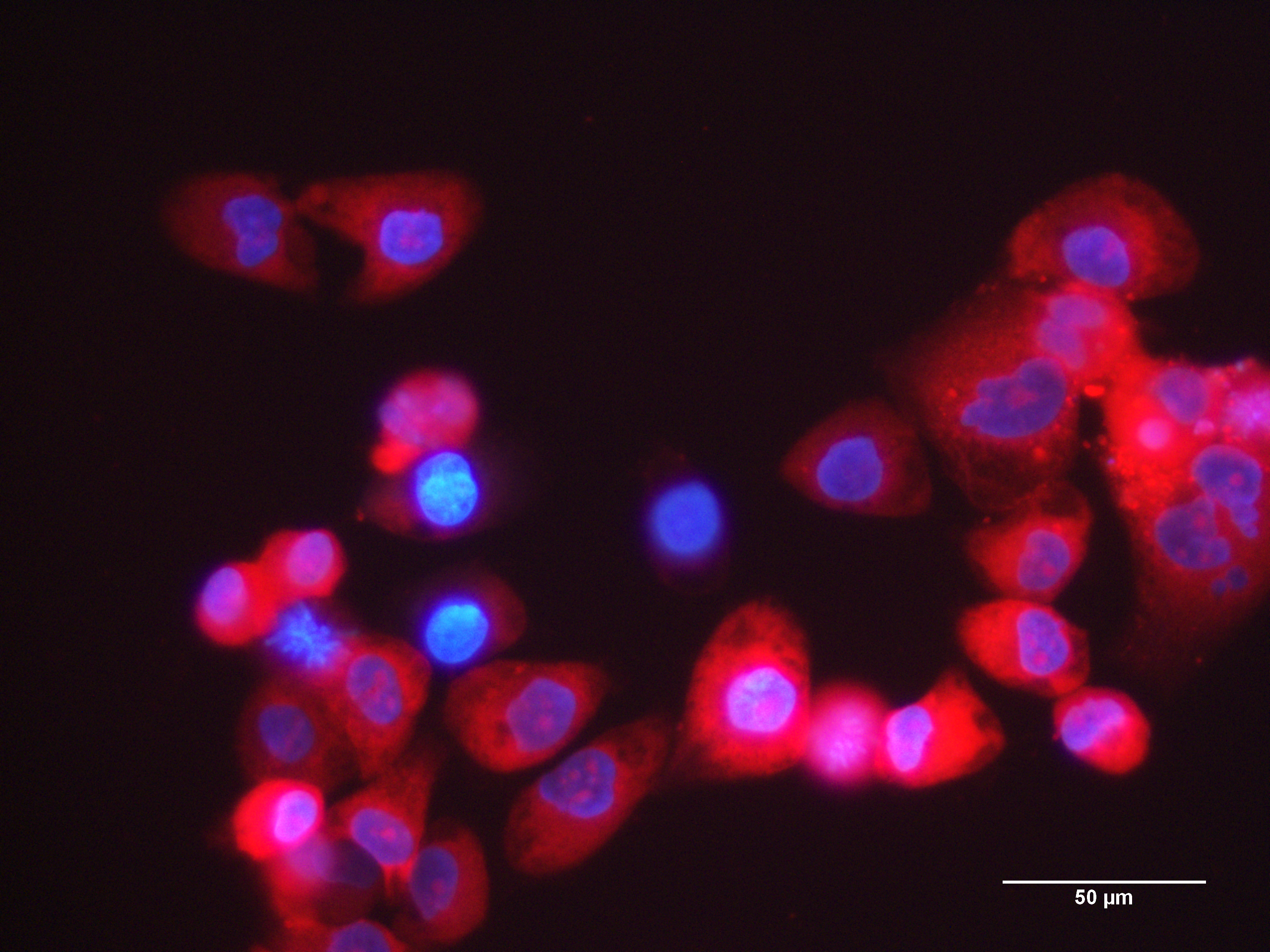

Supplement: Supplementary file 5 [file DataSheet6.zip › MitoSOX-2/MitoSOX═╝╞1⁄4/Spermine_Iohexol 3 merge.tif]

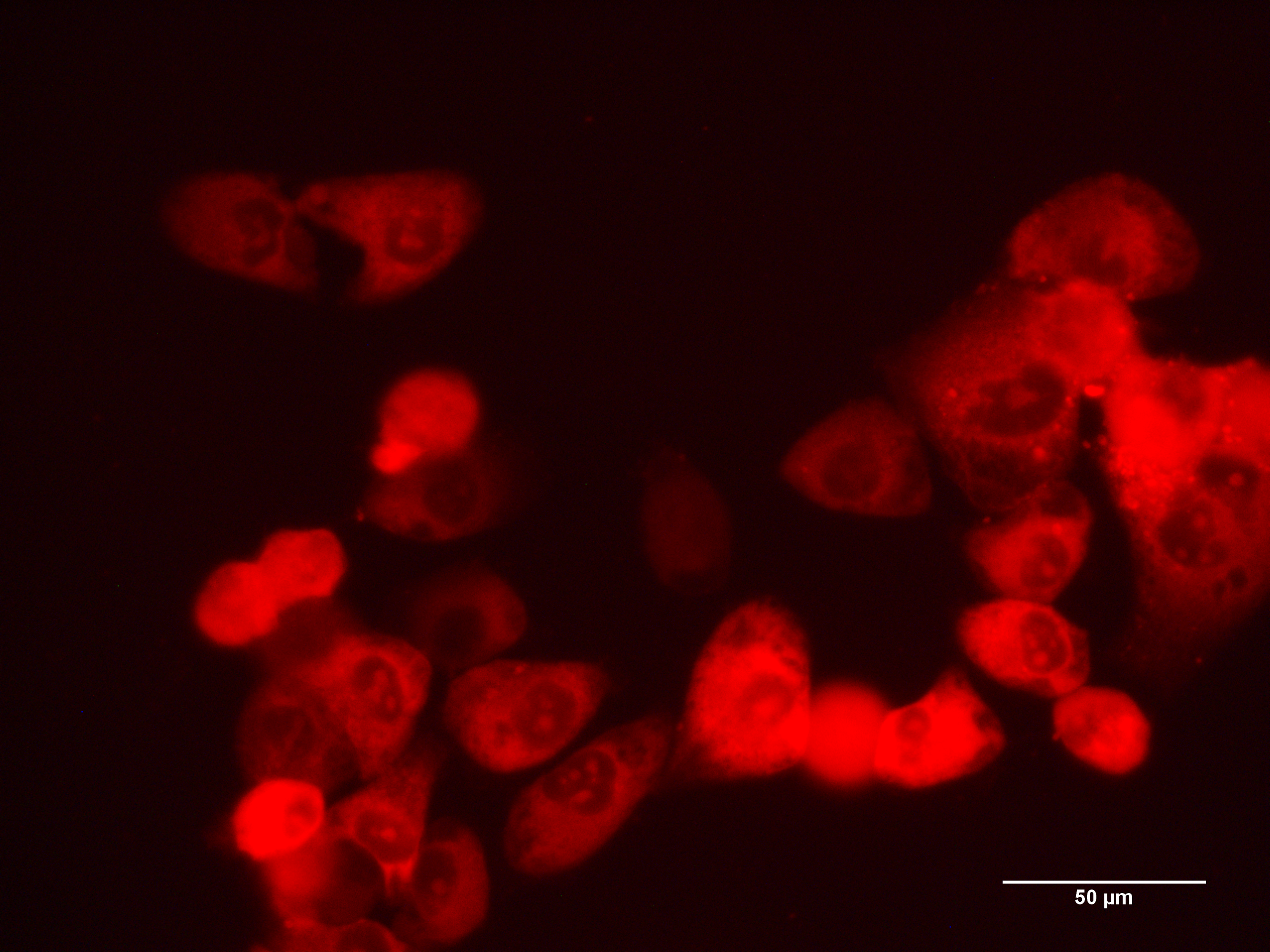

Supplement: Supplementary file 5 [file DataSheet6.zip › MitoSOX-2/MitoSOX═╝╞1⁄4/Spermine_Iohexol 3 MitoSOX.tif]

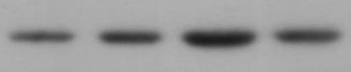

Supplement: Supplementary file 6 [file DataSheet2.zip › WB(1,2)/WB-1/DRP1+MCU+Tubulin/DRP1 1.jpg]

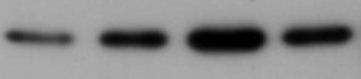

Supplement: Supplementary file 6 [file DataSheet2.zip › WB(1,2)/WB-1/DRP1+MCU+Tubulin/DRP1 2.jpg]

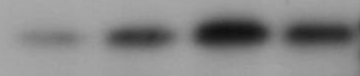

Supplement: Supplementary file 6 [file DataSheet2.zip › WB(1,2)/WB-1/DRP1+MCU+Tubulin/DRP1 3.jpg]

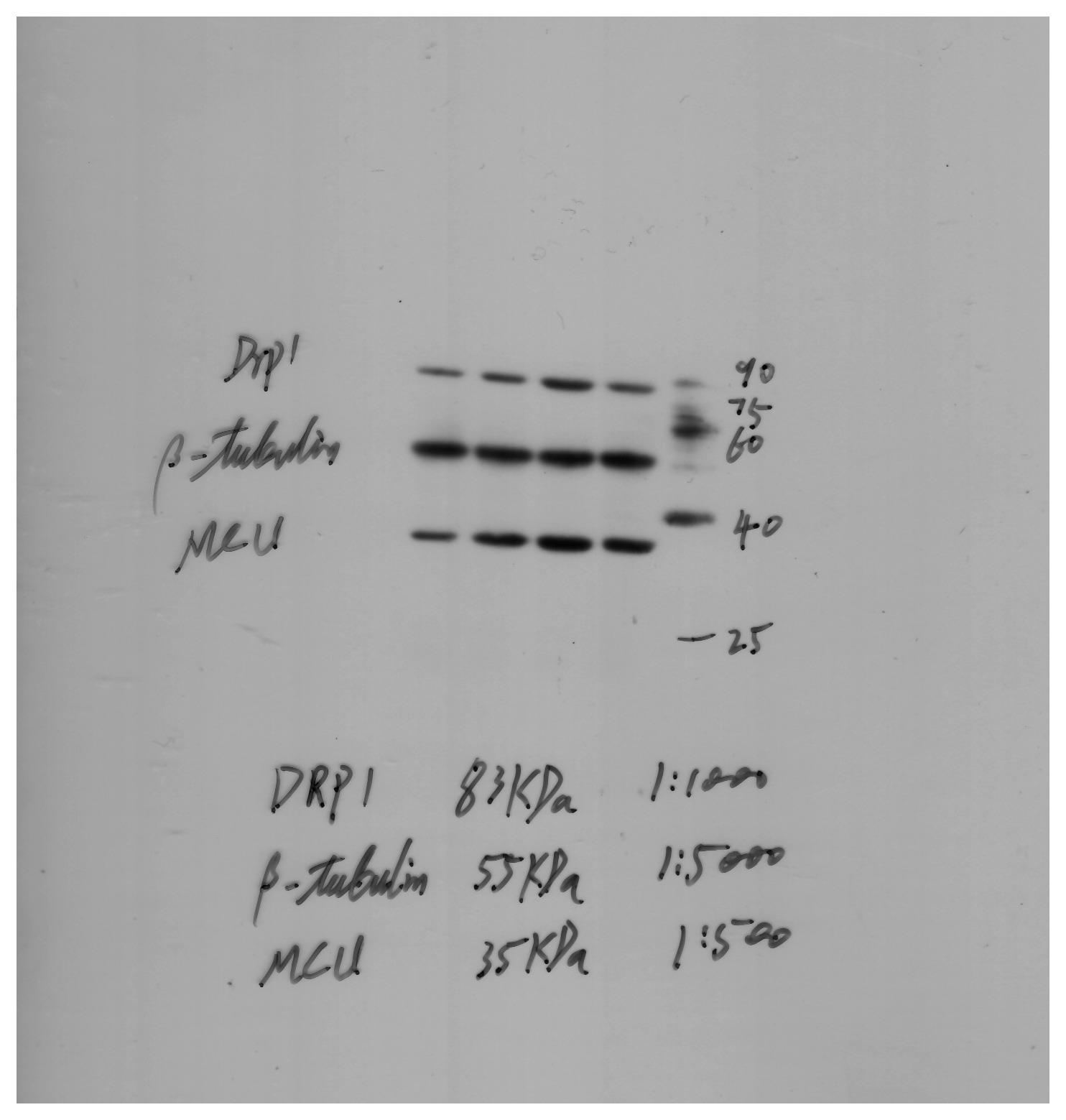

Supplement: Supplementary file 6 [file DataSheet2.zip › WB(1,2)/WB-1/DRP1+MCU+Tubulin/DRP1_MCU_Tubulin 1.jpg]

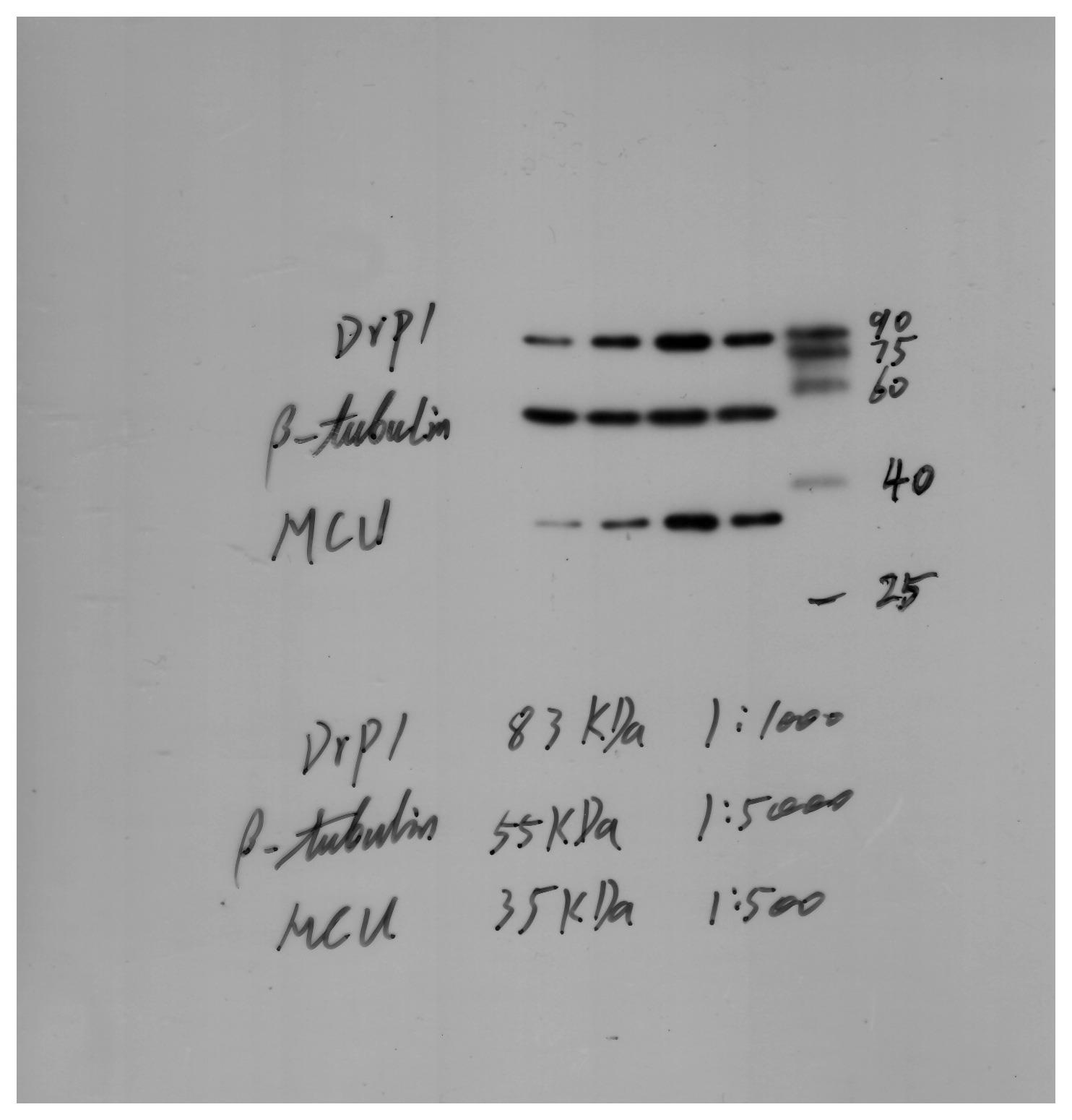

Supplement: Supplementary file 6 [file DataSheet2.zip › WB(1,2)/WB-1/DRP1+MCU+Tubulin/DRP1_MCU_Tubulin 2.jpg]

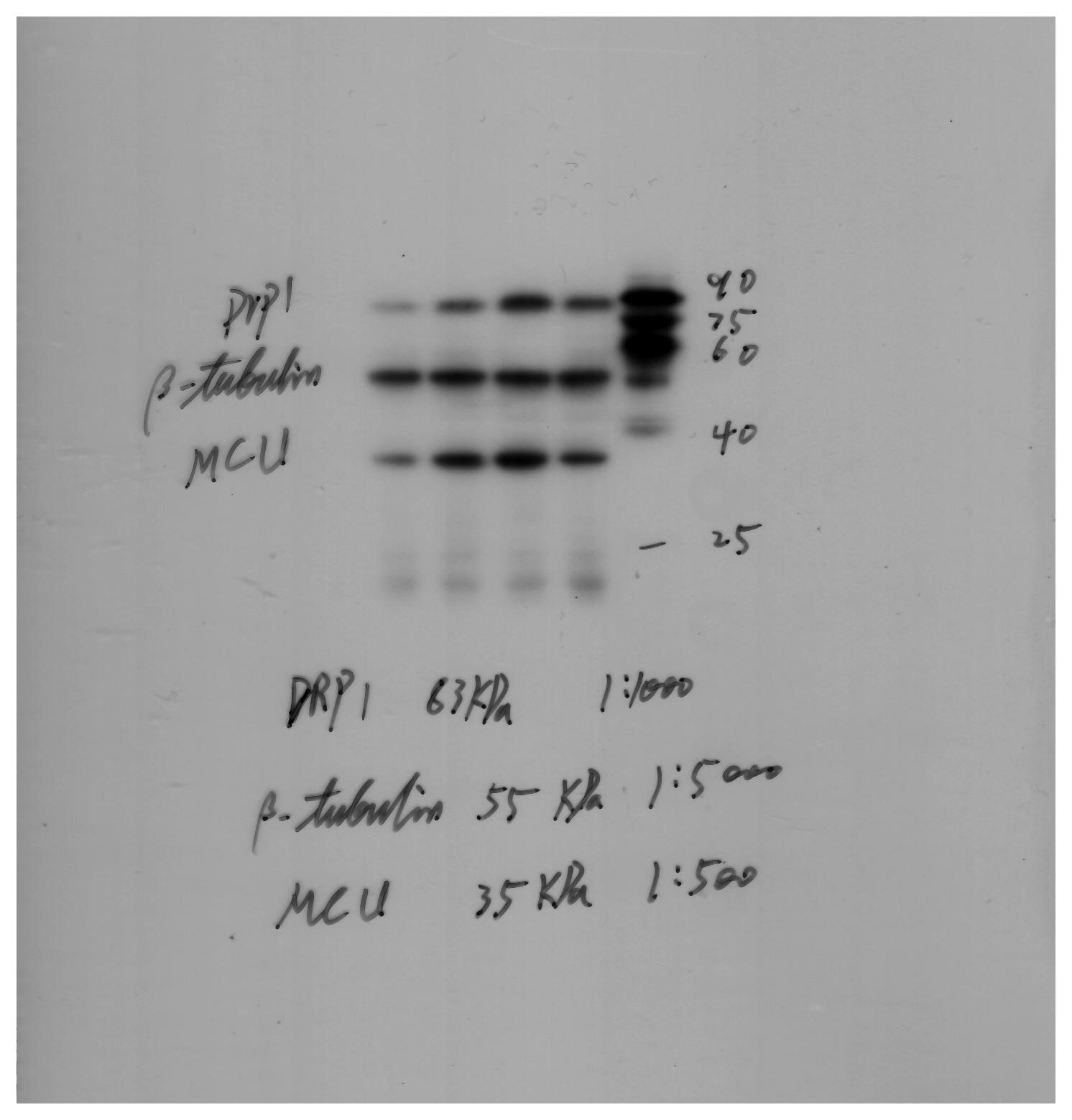

Supplement: Supplementary file 6 [file DataSheet2.zip › WB(1,2)/WB-1/DRP1+MCU+Tubulin/DRP1_MCU_Tubulin 3.jpg]

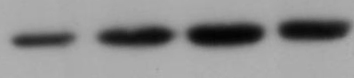

Supplement: Supplementary file 6 [file DataSheet2.zip › WB(1,2)/WB-1/DRP1+MCU+Tubulin/MCU 1.jpg]

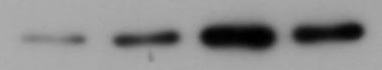

Supplement: Supplementary file 6 [file DataSheet2.zip › WB(1,2)/WB-1/DRP1+MCU+Tubulin/MCU 2.jpg]

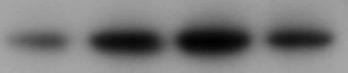

Supplement: Supplementary file 6 [file DataSheet2.zip › WB(1,2)/WB-1/DRP1+MCU+Tubulin/MCU 3.jpg]

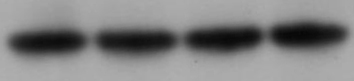

Supplement: Supplementary file 6 [file DataSheet2.zip › WB(1,2)/WB-1/DRP1+MCU+Tubulin/Tubulin 1.jpg]

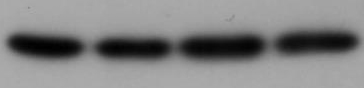

Supplement: Supplementary file 6 [file DataSheet2.zip › WB(1,2)/WB-1/DRP1+MCU+Tubulin/Tubulin 2.jpg]

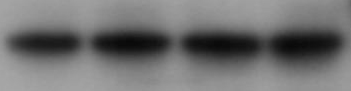

Supplement: Supplementary file 6 [file DataSheet2.zip › WB(1,2)/WB-1/DRP1+MCU+Tubulin/Tubulin 3.jpg]

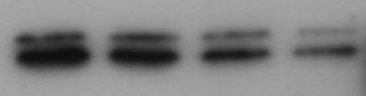

Supplement: Supplementary file 6 [file DataSheet2.zip › WB(1,2)/WB-1/OPA1+Tubulin/OPA1 1.jpg]

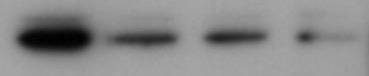

Supplement: Supplementary file 6 [file DataSheet2.zip › WB(1,2)/WB-1/OPA1+Tubulin/OPA1 2.jpg]

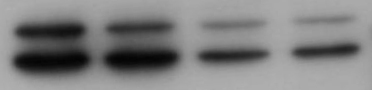

Supplement: Supplementary file 6 [file DataSheet2.zip › WB(1,2)/WB-1/OPA1+Tubulin/OPA1 3.jpg]

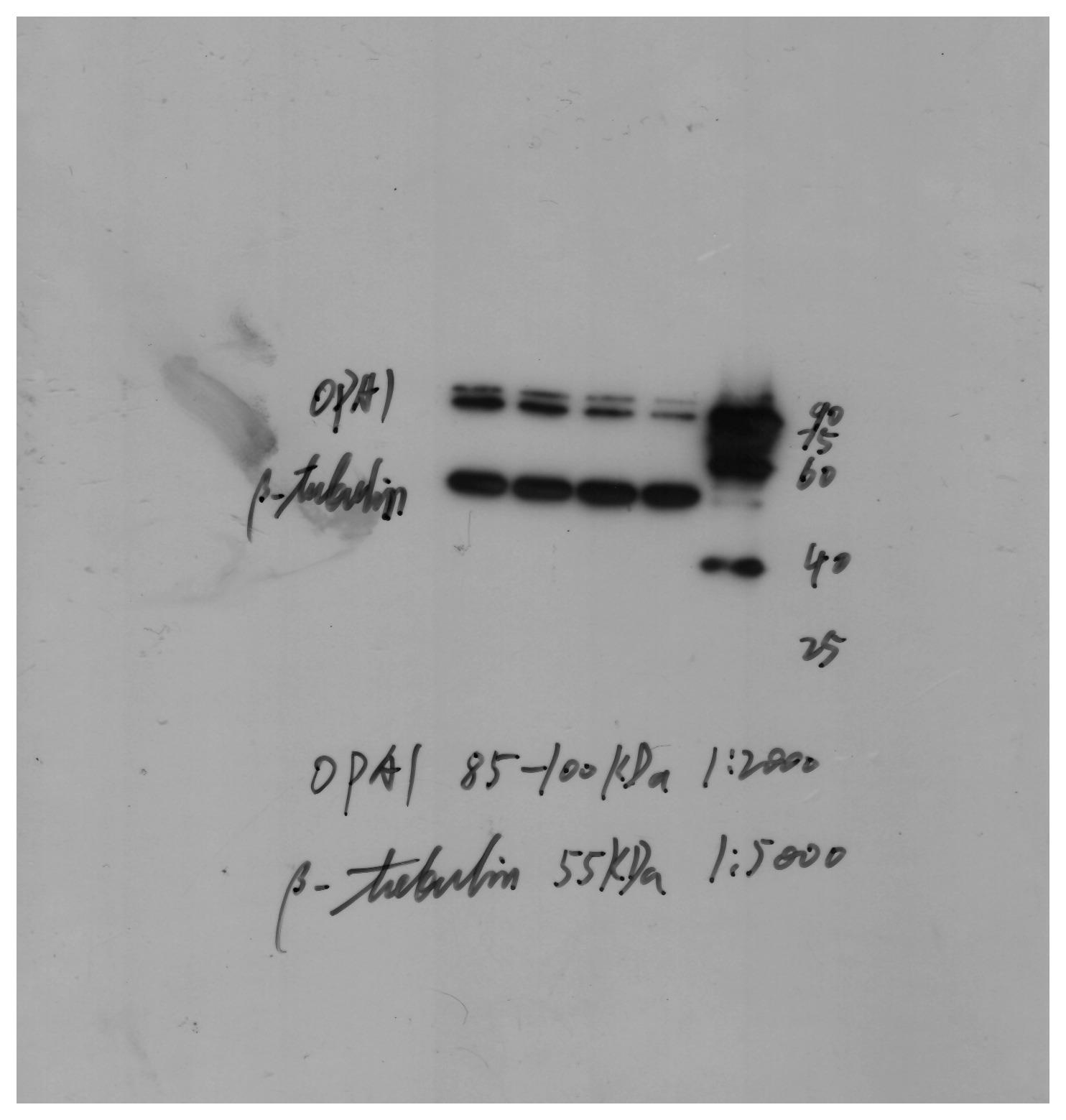

Supplement: Supplementary file 6 [file DataSheet2.zip › WB(1,2)/WB-1/OPA1+Tubulin/OPA1_Tubulin 1.jpg]

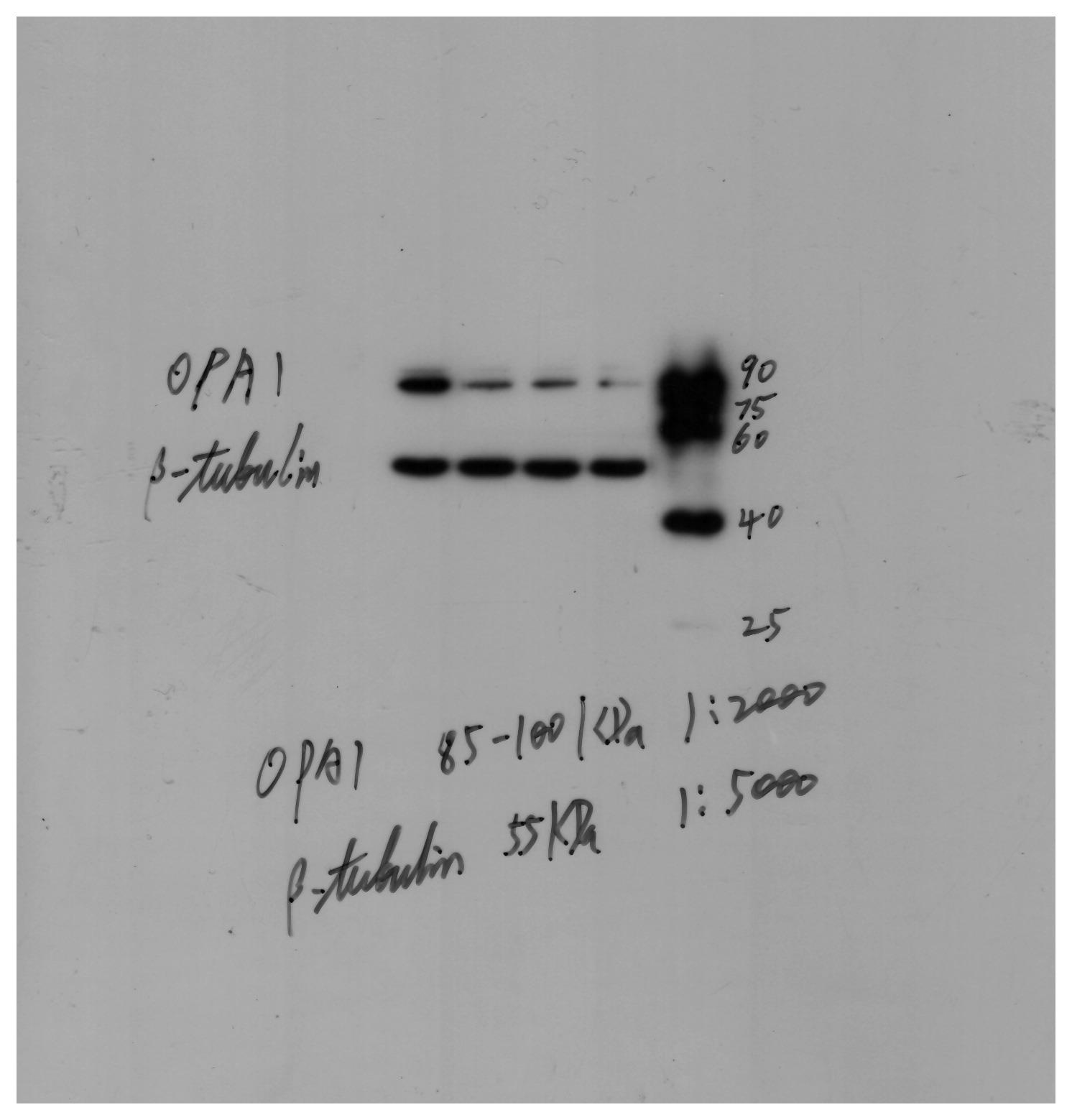

Supplement: Supplementary file 6 [file DataSheet2.zip › WB(1,2)/WB-1/OPA1+Tubulin/OPA1_Tubulin 2.jpg]

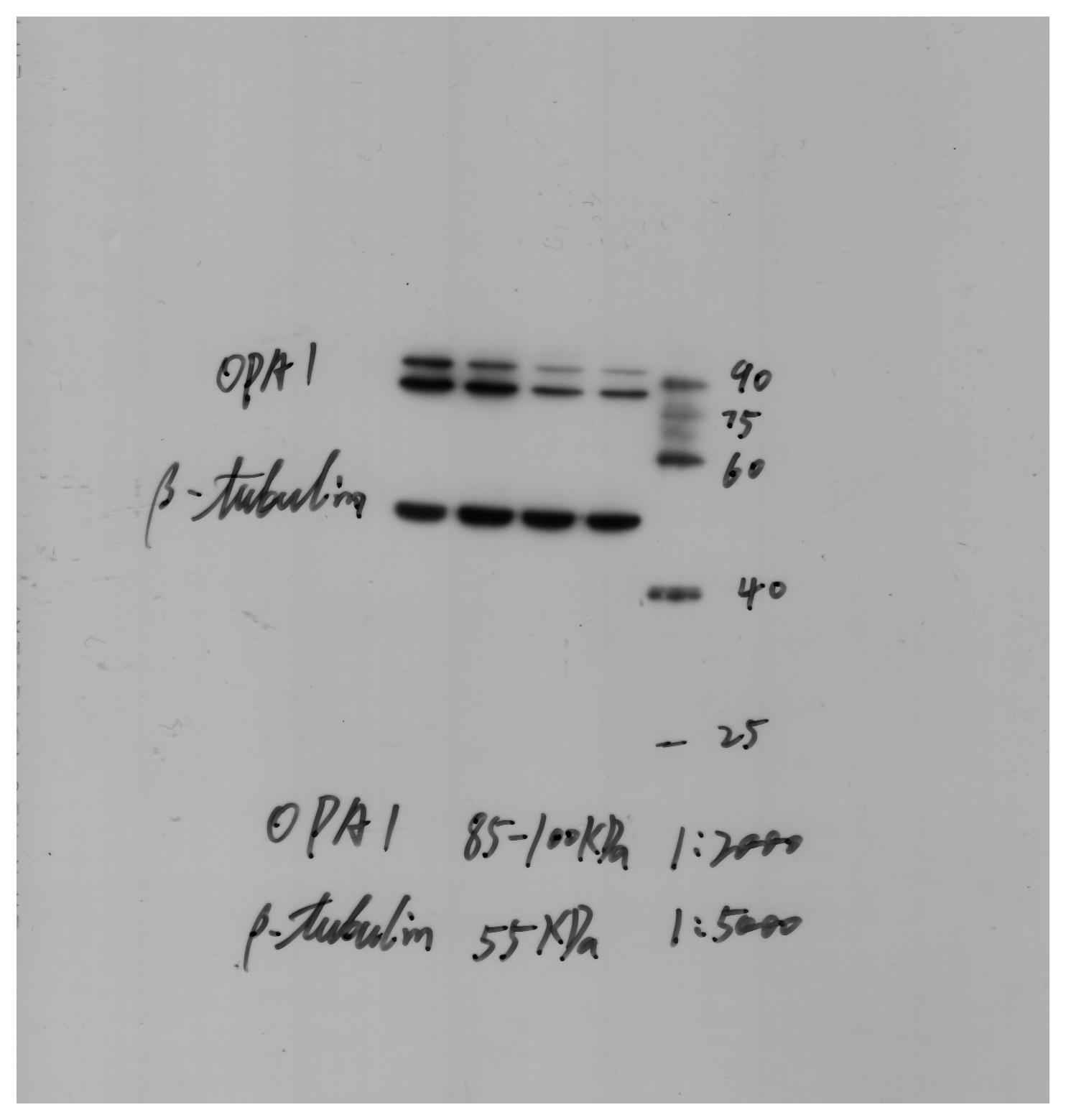

Supplement: Supplementary file 6 [file DataSheet2.zip › WB(1,2)/WB-1/OPA1+Tubulin/OPA1_Tubulin 3.jpg]

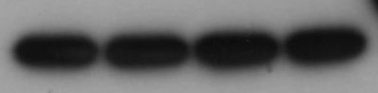

Supplement: Supplementary file 6 [file DataSheet2.zip › WB(1,2)/WB-1/OPA1+Tubulin/Tubulin 1.jpg]

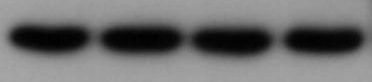

Supplement: Supplementary file 6 [file DataSheet2.zip › WB(1,2)/WB-1/OPA1+Tubulin/Tubulin 2.jpg]

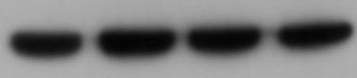

Supplement: Supplementary file 6 [file DataSheet2.zip › WB(1,2)/WB-1/OPA1+Tubulin/Tubulin 3.jpg]

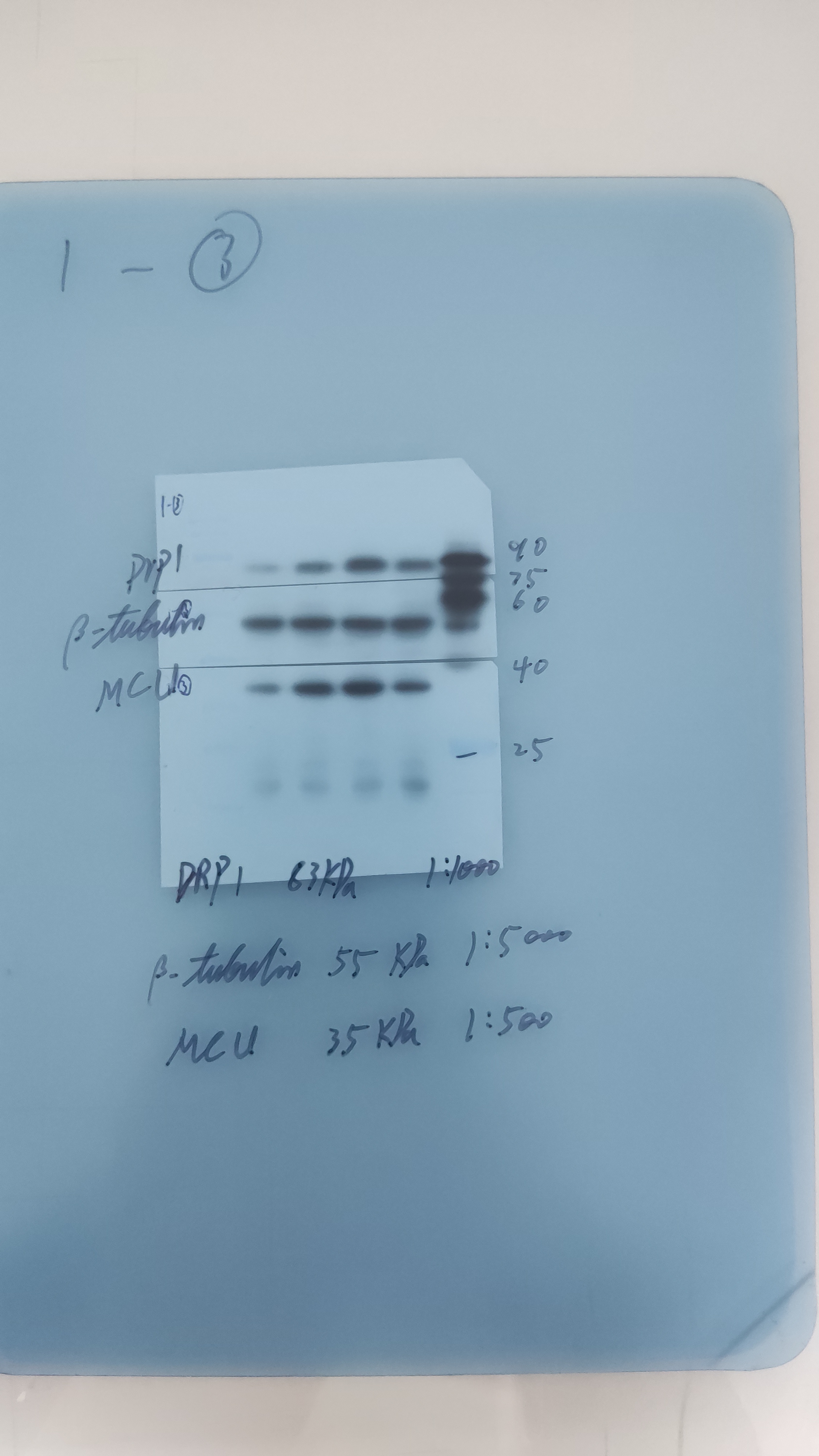

Supplement: Supplementary file 6 [file DataSheet2.zip › WB(1,2)/WB-1/╜║╞1⁄4/1.jpg]

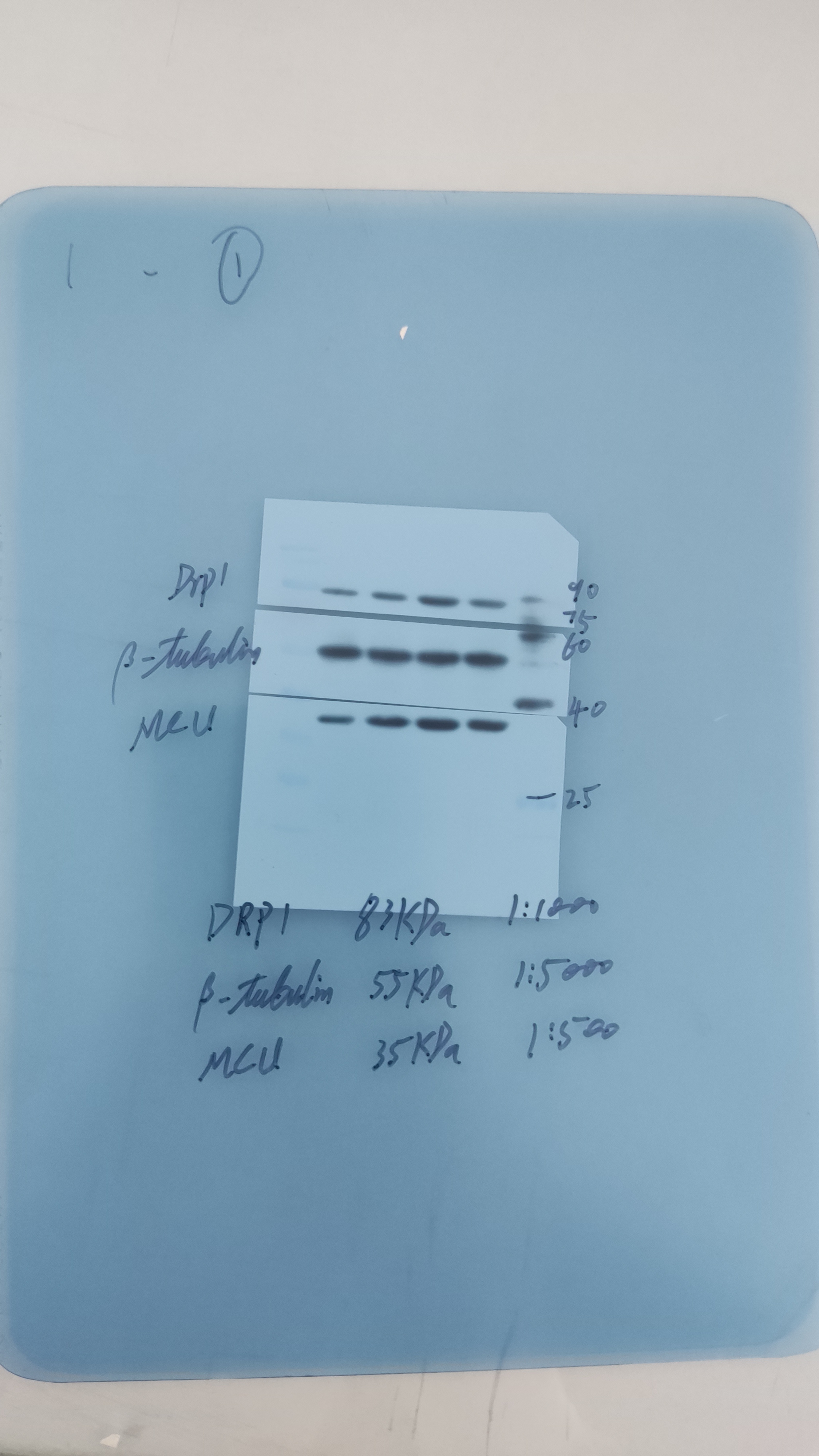

Supplement: Supplementary file 6 [file DataSheet2.zip › WB(1,2)/WB-1/╜║╞1⁄4/2.jpg]

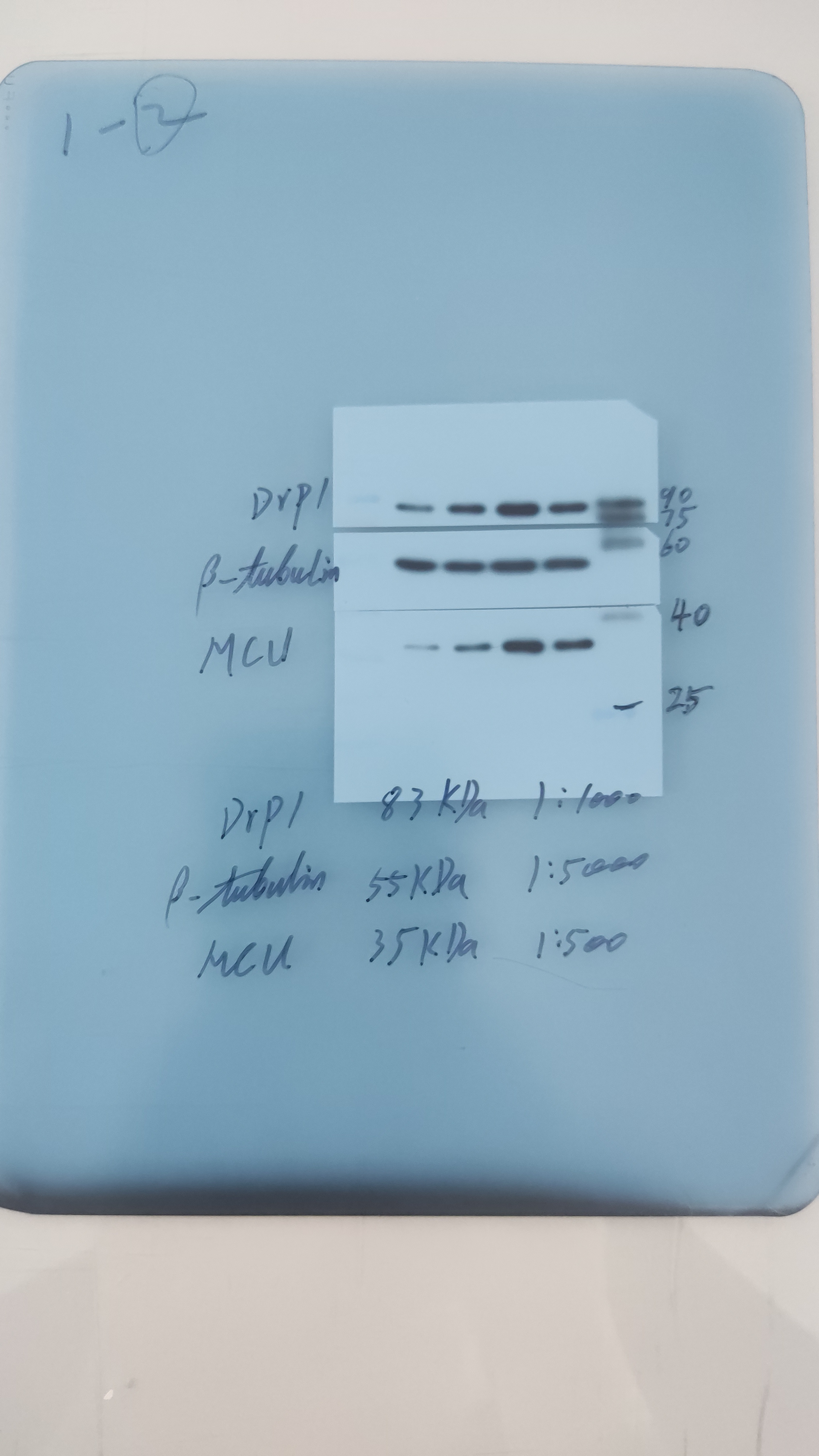

Supplement: Supplementary file 6 [file DataSheet2.zip › WB(1,2)/WB-1/╜║╞1⁄4/3.jpg]

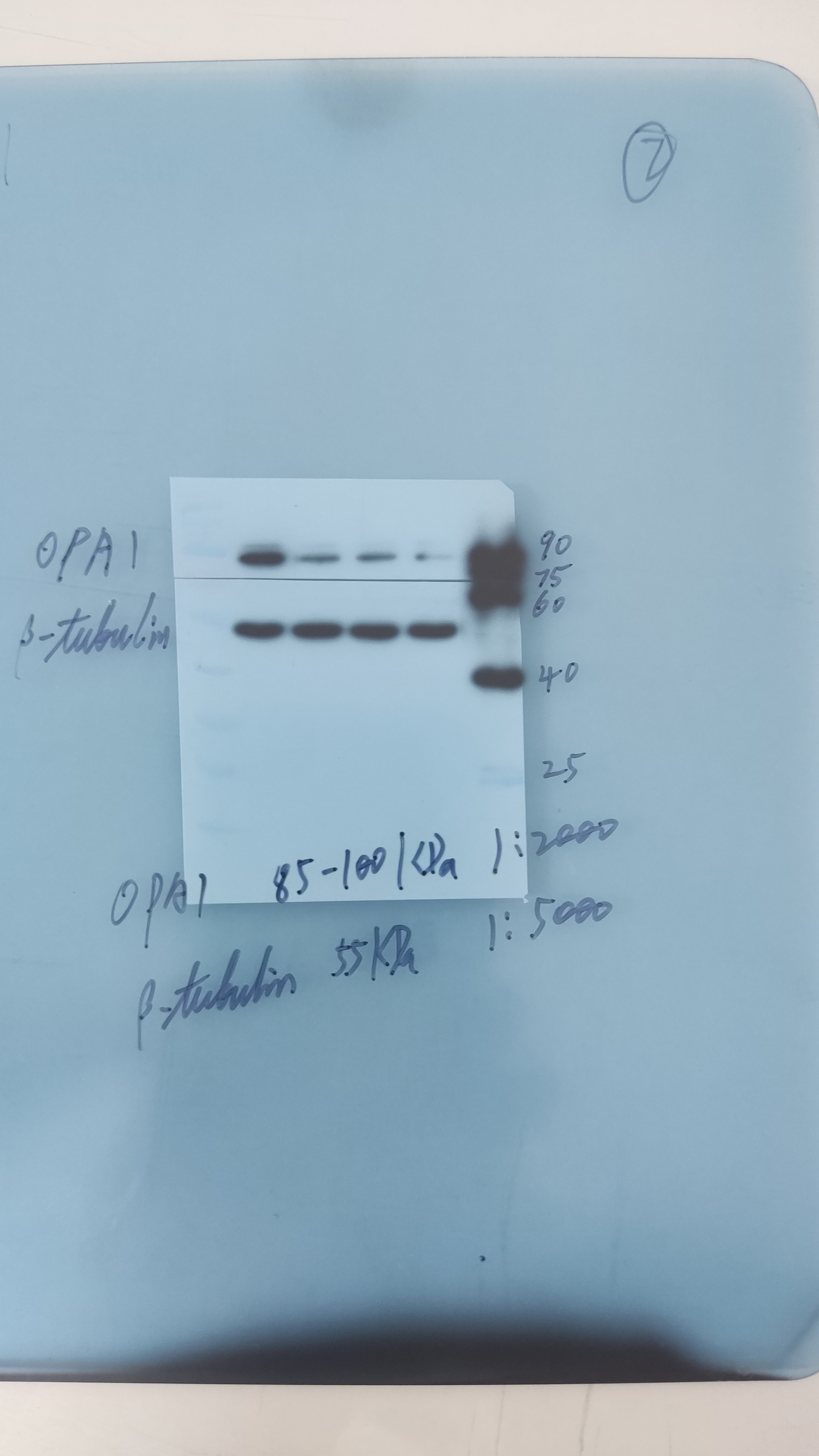

Supplement: Supplementary file 6 [file DataSheet2.zip › WB(1,2)/WB-1/╜║╞1⁄4/4.jpg]

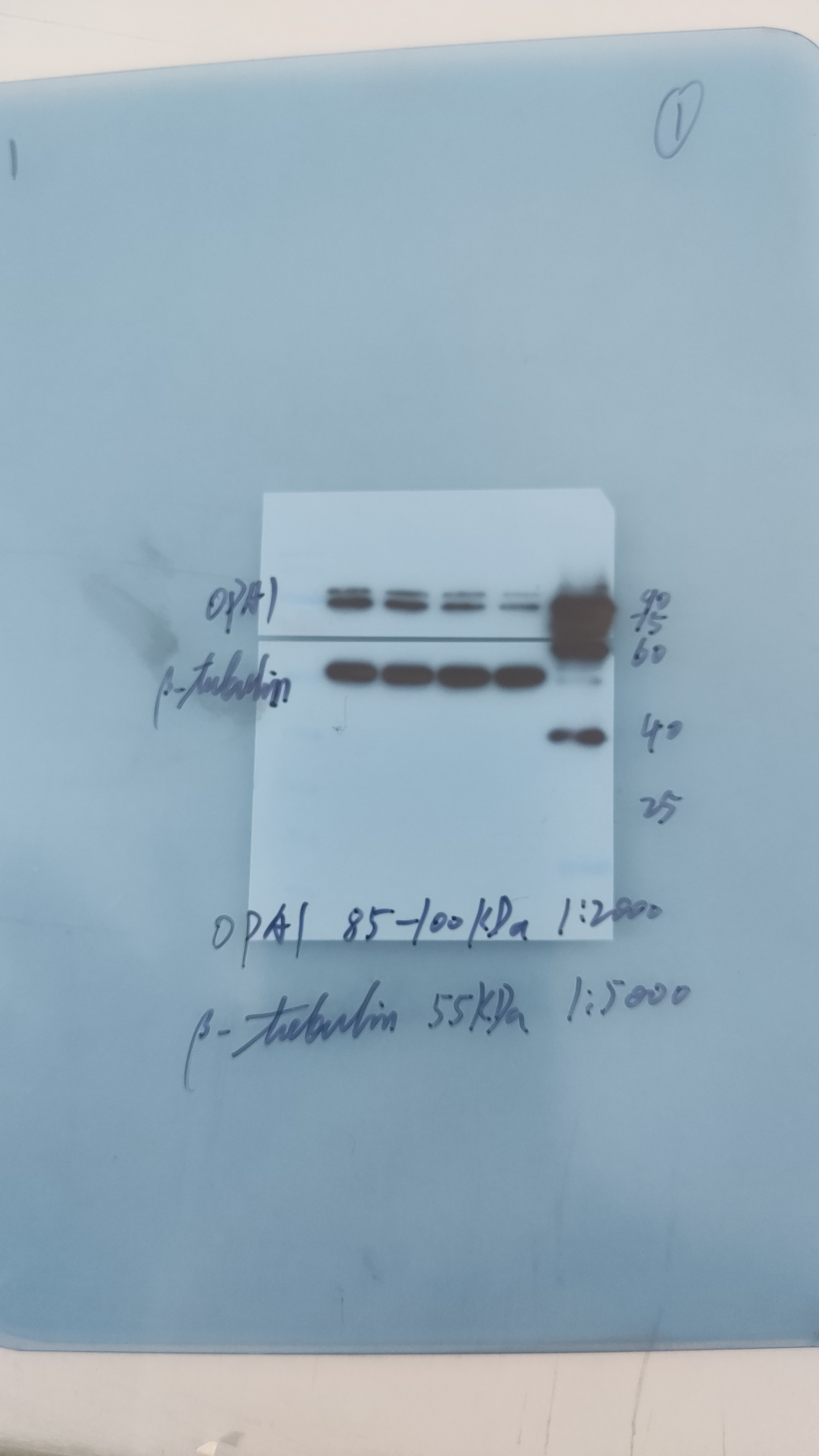

Supplement: Supplementary file 6 [file DataSheet2.zip › WB(1,2)/WB-1/╜║╞1⁄4/5.jpg]

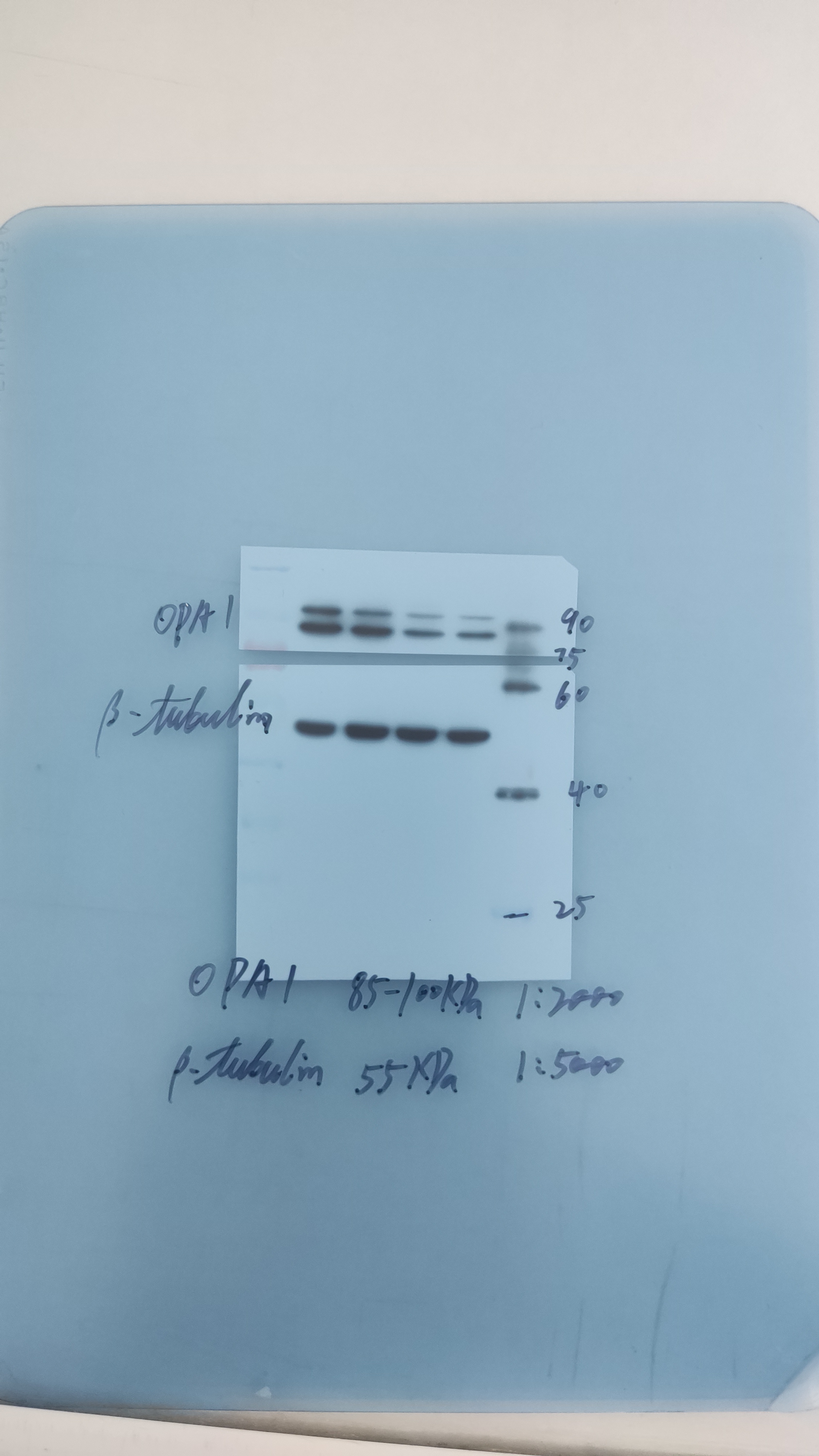

Supplement: Supplementary file 6 [file DataSheet2.zip › WB(1,2)/WB-1/╜║╞1⁄4/6.jpg]

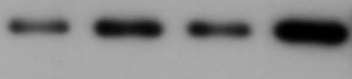

Supplement: Supplementary file 6 [file DataSheet2.zip › WB(1,2)/WB-2/DRP1+MCU+Tubulin/DRP1 1.jpg]

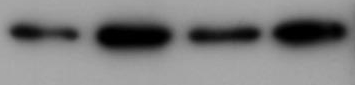

Supplement: Supplementary file 6 [file DataSheet2.zip › WB(1,2)/WB-2/DRP1+MCU+Tubulin/DRP1 2.jpg]

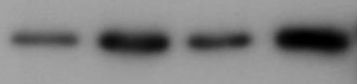

Supplement: Supplementary file 6 [file DataSheet2.zip › WB(1,2)/WB-2/DRP1+MCU+Tubulin/DRP1 3.jpg]
