# Supplementary figures and images for: Mitochondrial calcium uniporter-mediated mitochondrial dynamics imbalance contributes to contrast medium-induced renal tubular cell injury (part 4 of 4)
Source: Front Mol Biosci. 2026 Jun 29;13:1848361. doi: 10.3389/fmolb.2026.1848361 (PMC13357276; doi:10.3389/fmolb.2026.1848361)

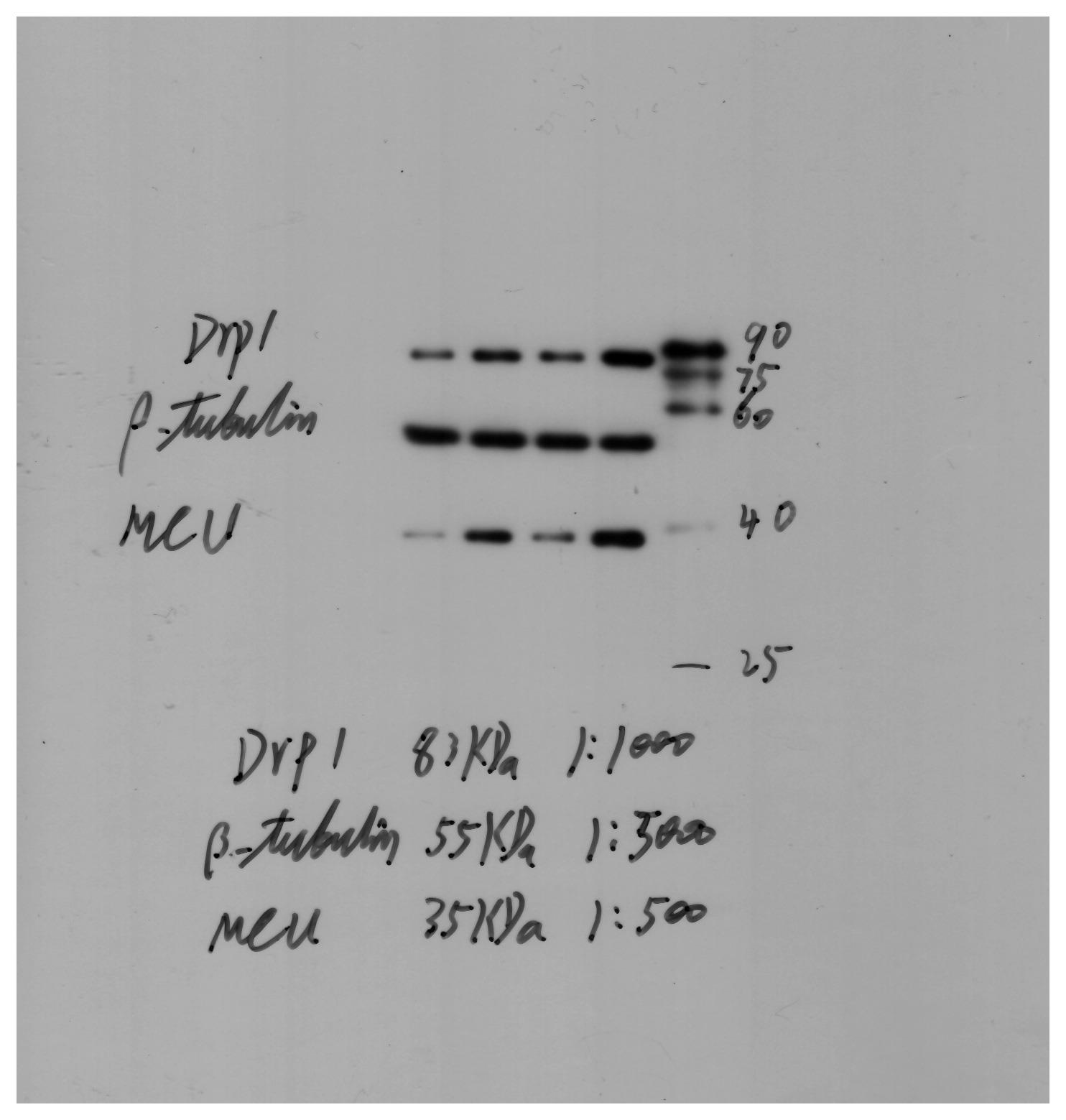

Supplement: Supplementary file 6 [file DataSheet2.zip › WB(1,2)/WB-2/DRP1+MCU+Tubulin/DRP1_MCU_Tubulin 1.jpg]

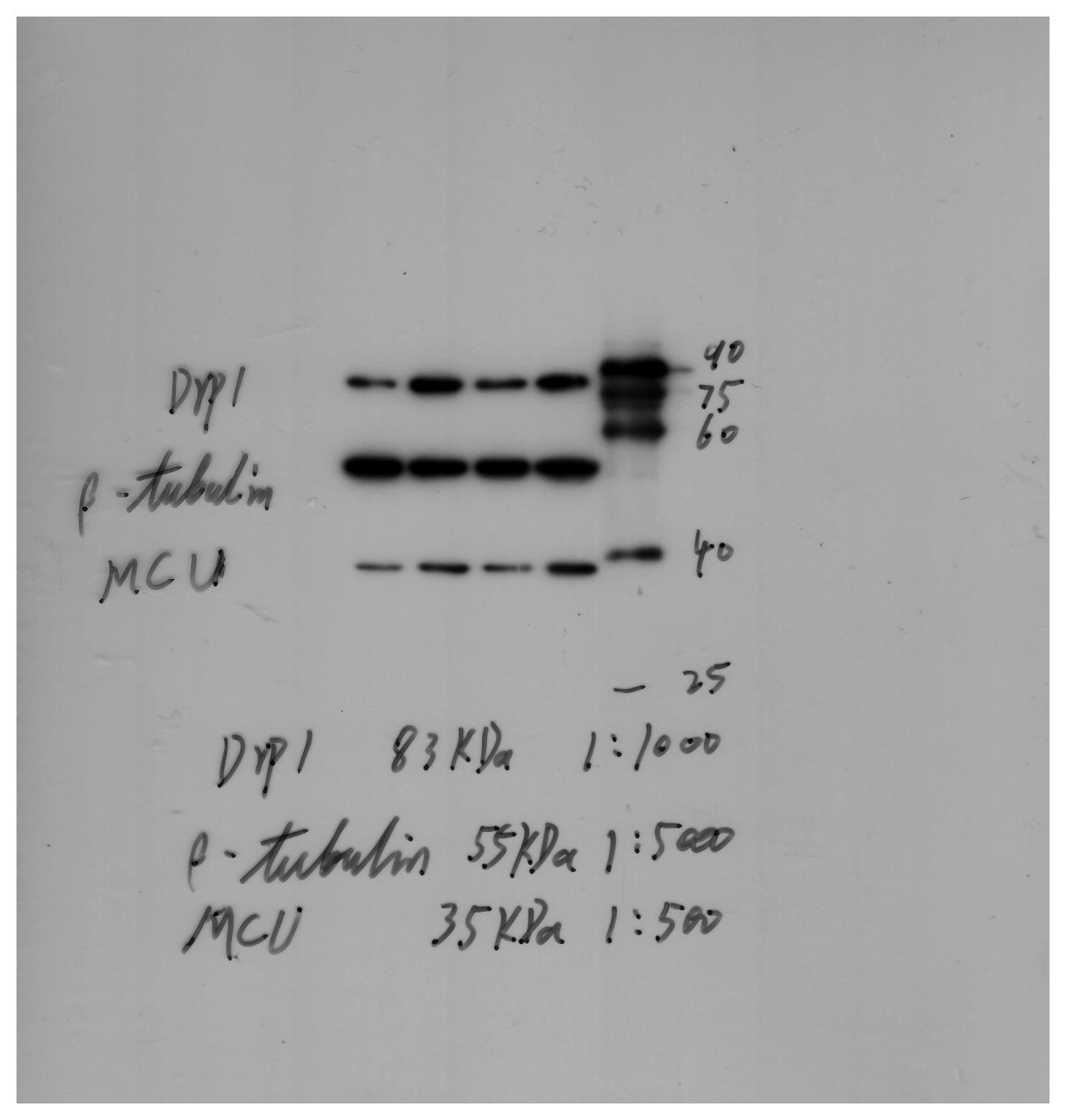

Supplement: Supplementary file 6 [file DataSheet2.zip › WB(1,2)/WB-2/DRP1+MCU+Tubulin/DRP1_MCU_Tubulin 2.jpg]

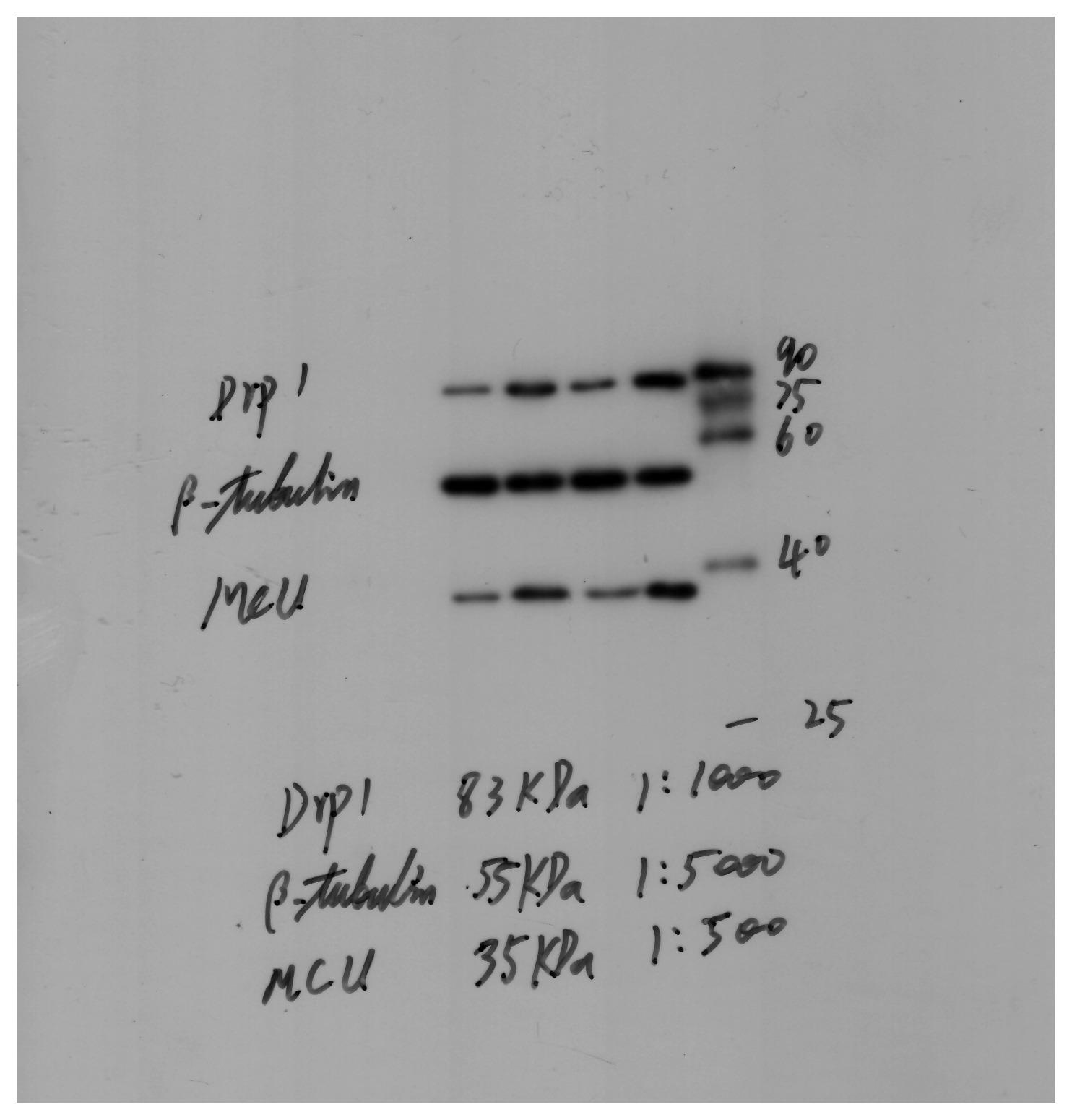

Supplement: Supplementary file 6 [file DataSheet2.zip › WB(1,2)/WB-2/DRP1+MCU+Tubulin/DRP1_MCU_Tubulin 3.jpg]

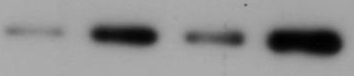

Supplement: Supplementary file 6 [file DataSheet2.zip › WB(1,2)/WB-2/DRP1+MCU+Tubulin/MCU 1.jpg]

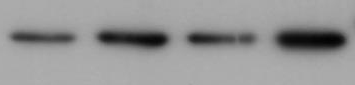

Supplement: Supplementary file 6 [file DataSheet2.zip › WB(1,2)/WB-2/DRP1+MCU+Tubulin/MCU 2.jpg]

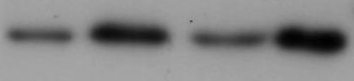

Supplement: Supplementary file 6 [file DataSheet2.zip › WB(1,2)/WB-2/DRP1+MCU+Tubulin/MCU 3.jpg]

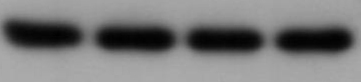

Supplement: Supplementary file 6 [file DataSheet2.zip › WB(1,2)/WB-2/DRP1+MCU+Tubulin/Tubulin 1.jpg]

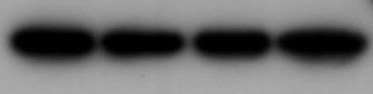

Supplement: Supplementary file 6 [file DataSheet2.zip › WB(1,2)/WB-2/DRP1+MCU+Tubulin/Tubulin 2.jpg]

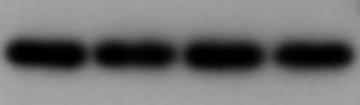

Supplement: Supplementary file 6 [file DataSheet2.zip › WB(1,2)/WB-2/DRP1+MCU+Tubulin/Tubulin 3.jpg]

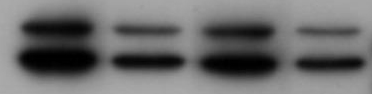

Supplement: Supplementary file 6 [file DataSheet2.zip › WB(1,2)/WB-2/OPA1+Tubulin/OPA1 1.jpg]

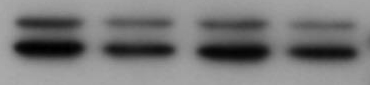

Supplement: Supplementary file 6 [file DataSheet2.zip › WB(1,2)/WB-2/OPA1+Tubulin/OPA1 2.jpg]

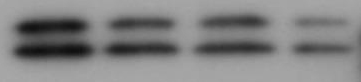

Supplement: Supplementary file 6 [file DataSheet2.zip › WB(1,2)/WB-2/OPA1+Tubulin/OPA1 3.jpg]

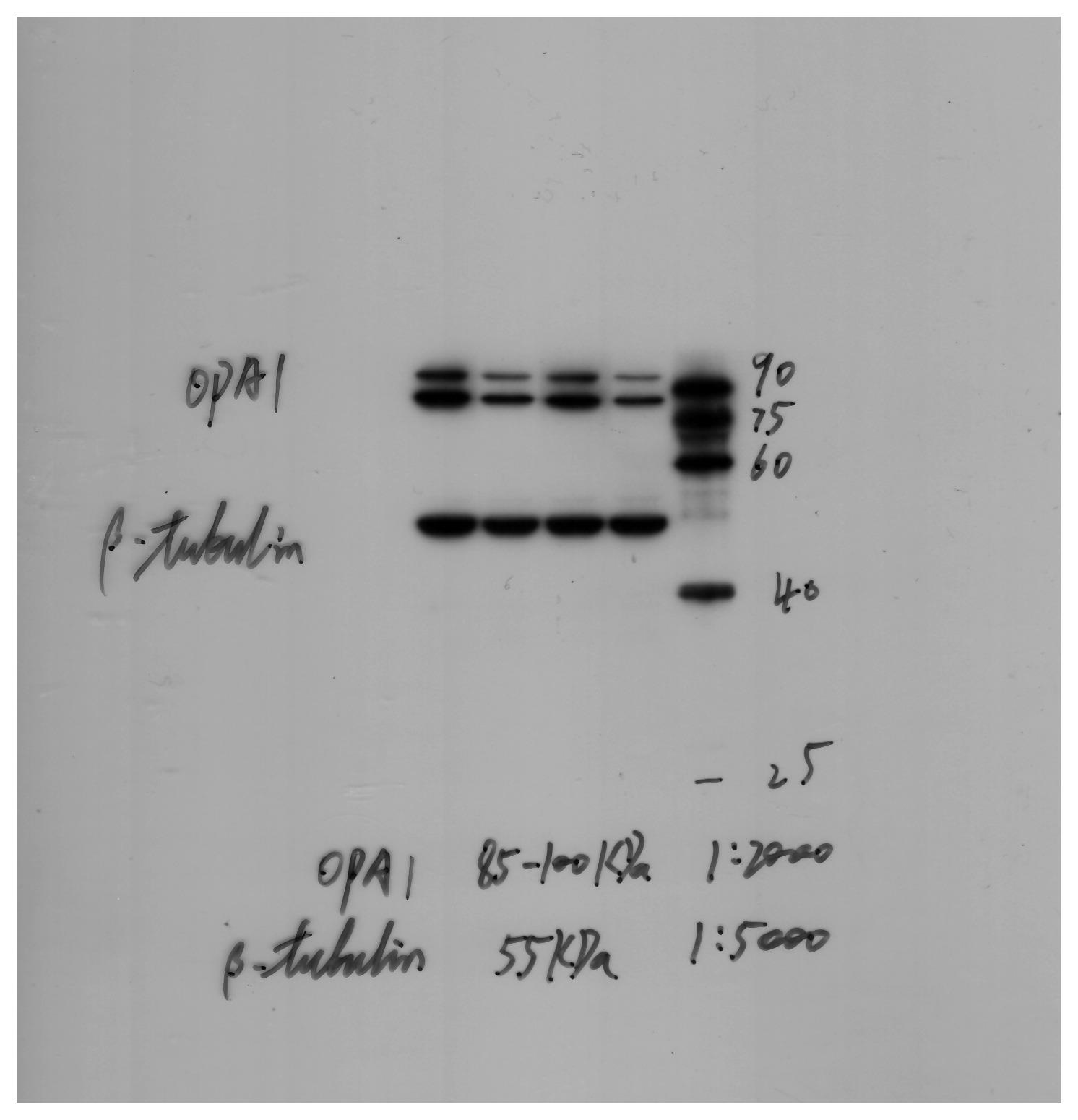

Supplement: Supplementary file 6 [file DataSheet2.zip › WB(1,2)/WB-2/OPA1+Tubulin/OPA1_Tubulin 1.jpg]

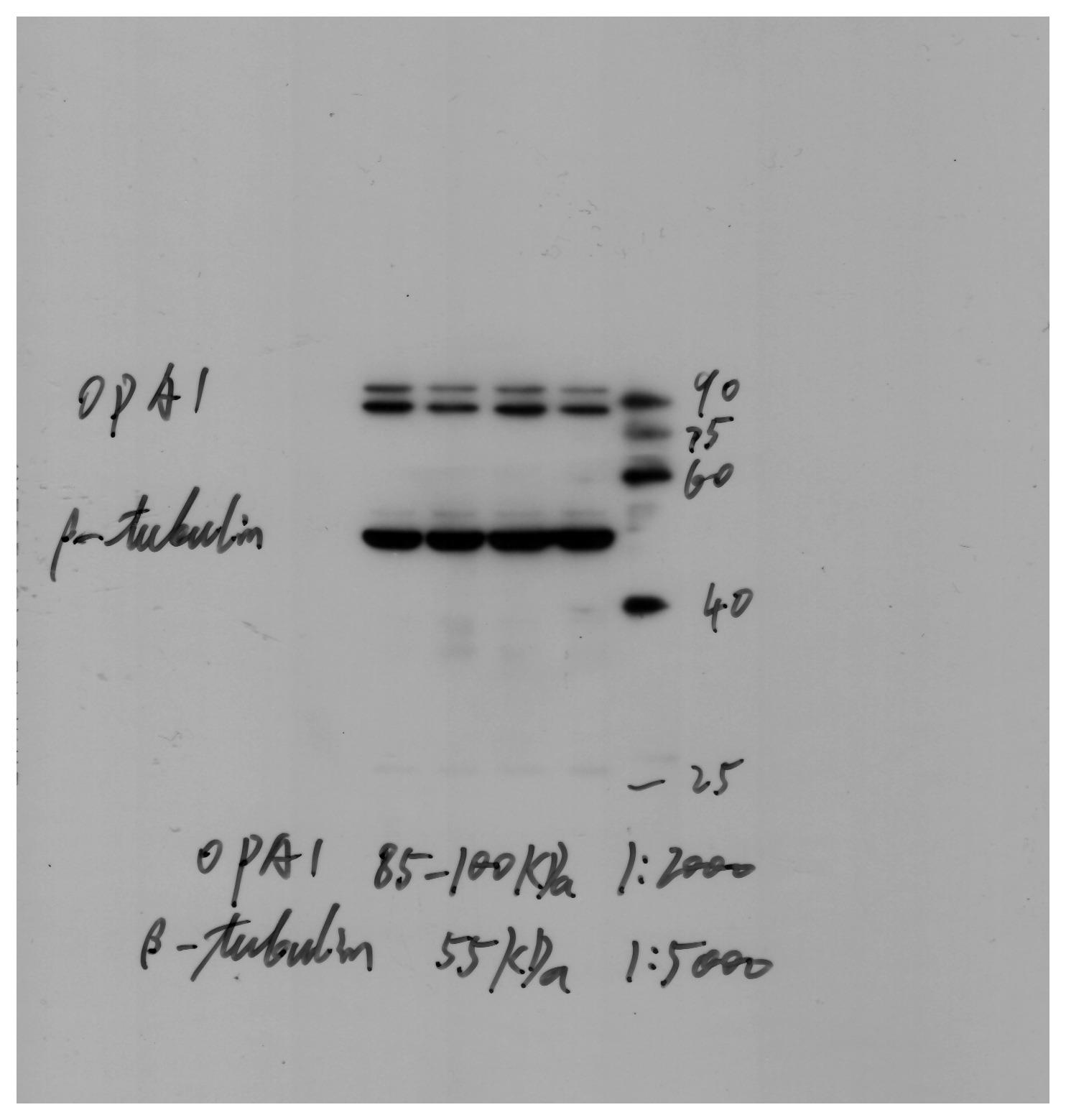

Supplement: Supplementary file 6 [file DataSheet2.zip › WB(1,2)/WB-2/OPA1+Tubulin/OPA1_Tubulin 2.jpg]

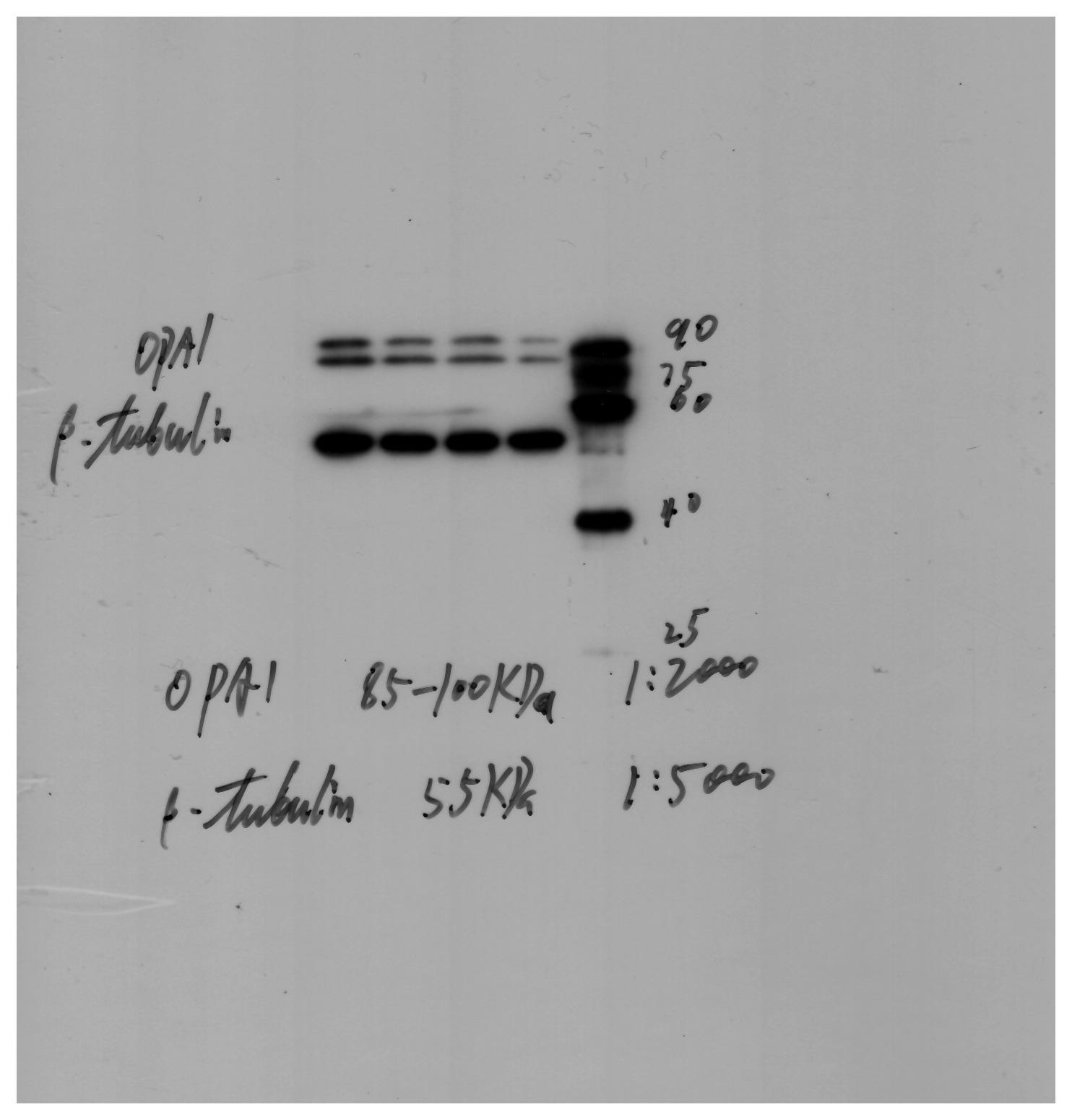

Supplement: Supplementary file 6 [file DataSheet2.zip › WB(1,2)/WB-2/OPA1+Tubulin/OPA1_Tubulin 3.jpg]

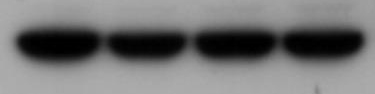

Supplement: Supplementary file 6 [file DataSheet2.zip › WB(1,2)/WB-2/OPA1+Tubulin/Tubulin 1.jpg]

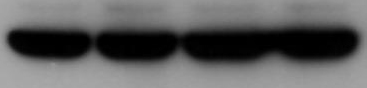

Supplement: Supplementary file 6 [file DataSheet2.zip › WB(1,2)/WB-2/OPA1+Tubulin/Tubulin 2.jpg]

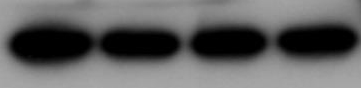

Supplement: Supplementary file 6 [file DataSheet2.zip › WB(1,2)/WB-2/OPA1+Tubulin/Tubulin 3.jpg]

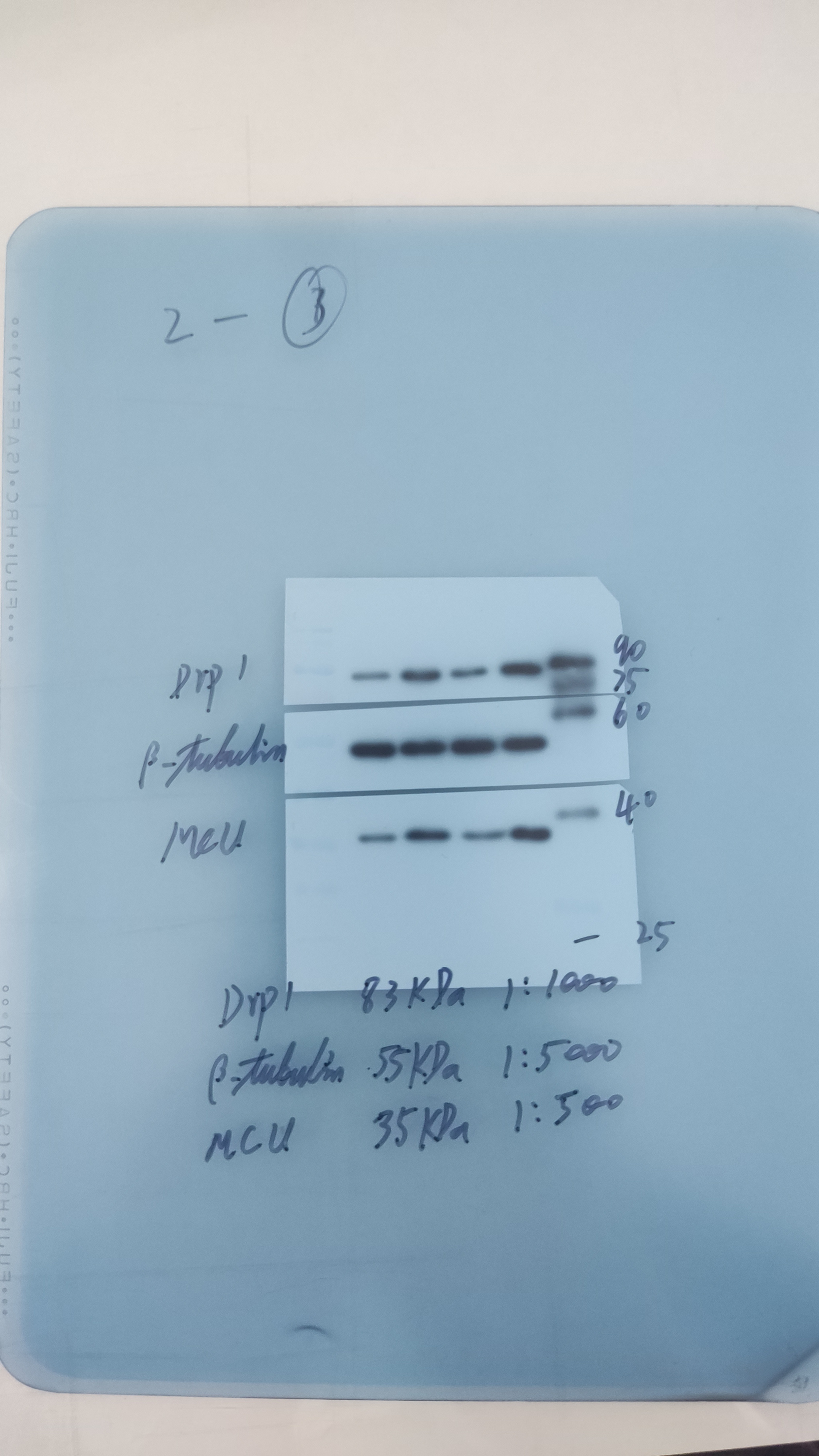

Supplement: Supplementary file 6 [file DataSheet2.zip › WB(1,2)/WB-2/╜║╞1⁄42/1.jpg]

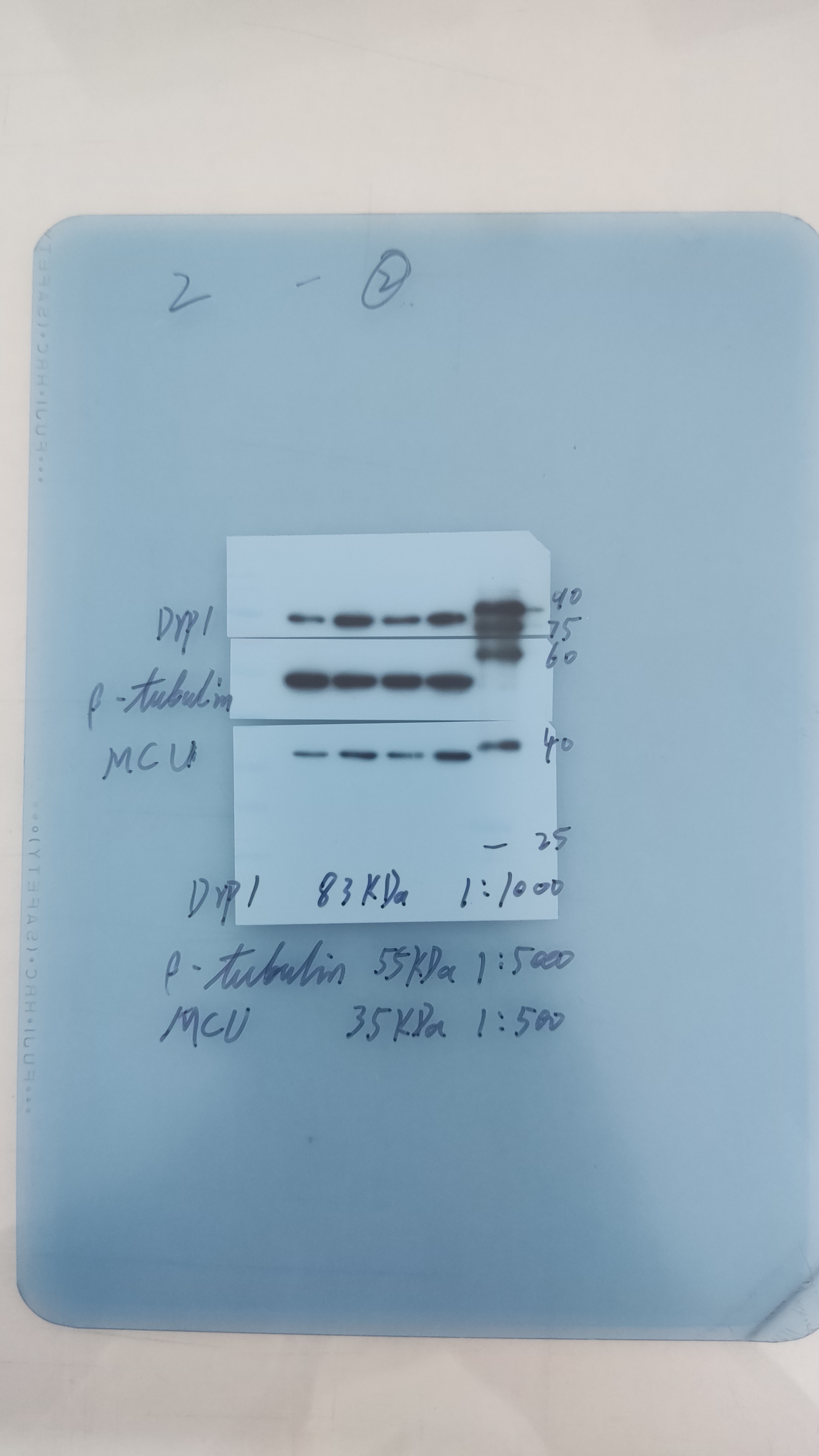

Supplement: Supplementary file 6 [file DataSheet2.zip › WB(1,2)/WB-2/╜║╞1⁄42/2.jpg]

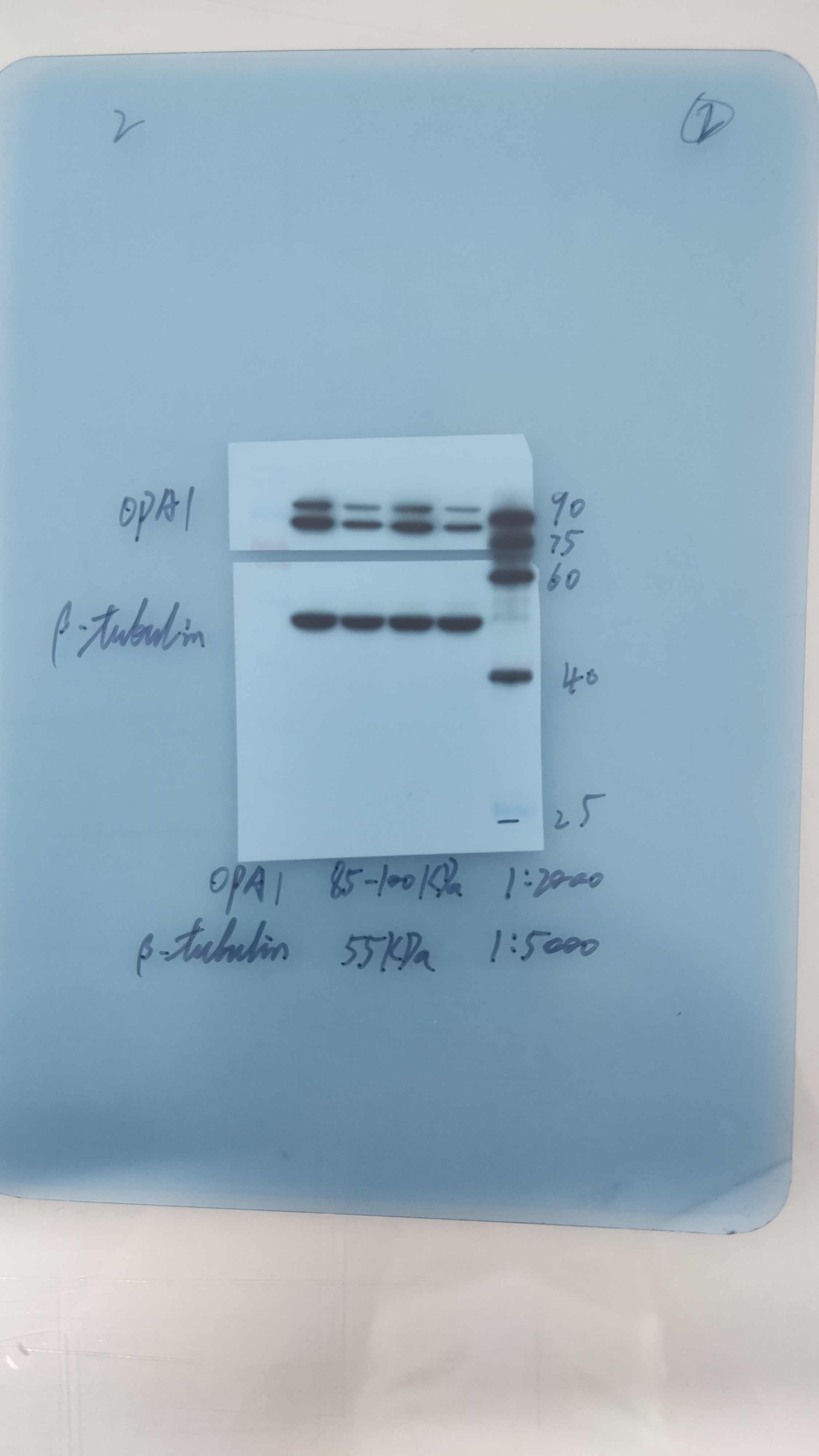

Supplement: Supplementary file 6 [file DataSheet2.zip › WB(1,2)/WB-2/╜║╞1⁄42/3.jpg]

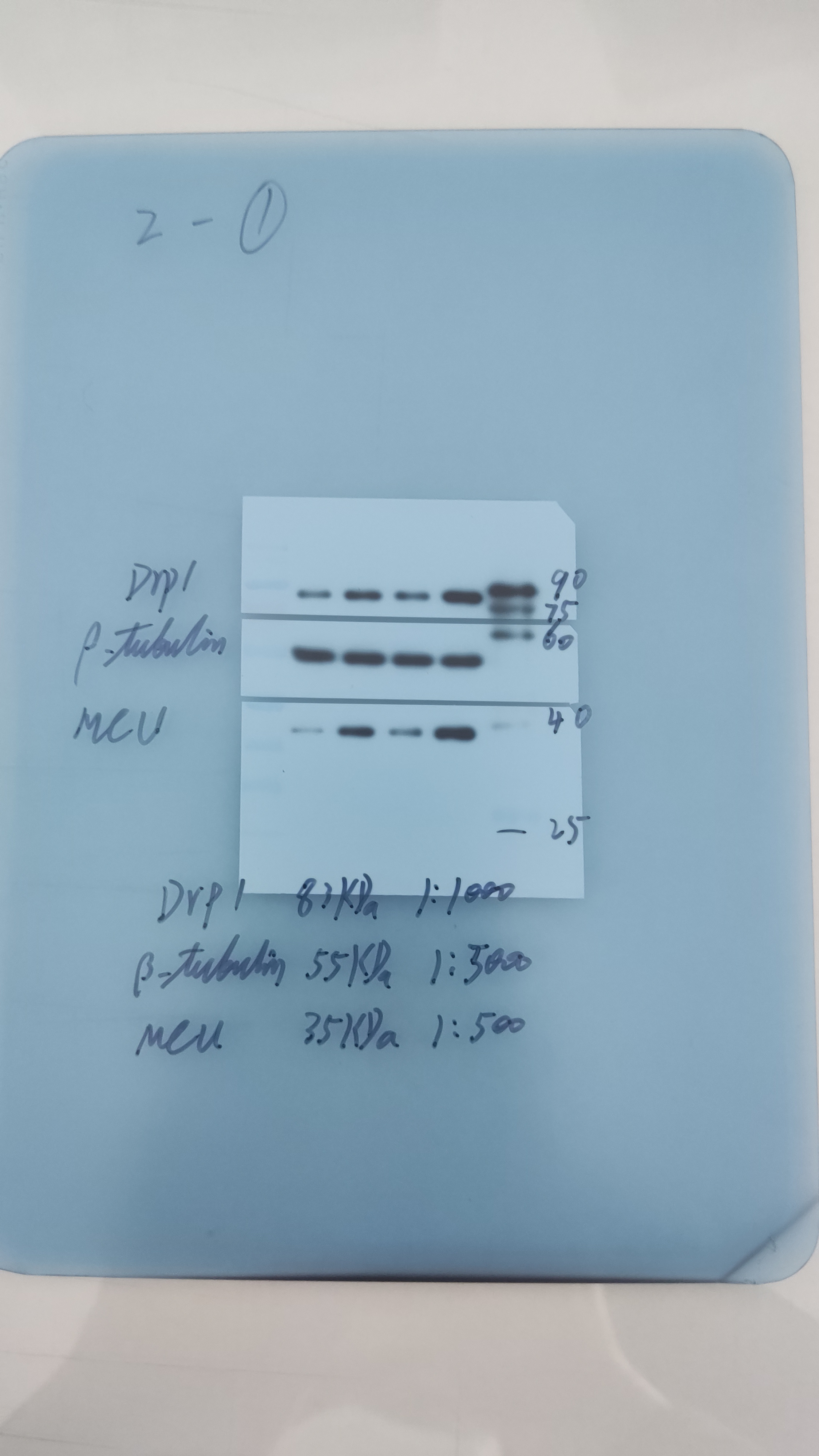

Supplement: Supplementary file 6 [file DataSheet2.zip › WB(1,2)/WB-2/╜║╞1⁄42/4.jpg]

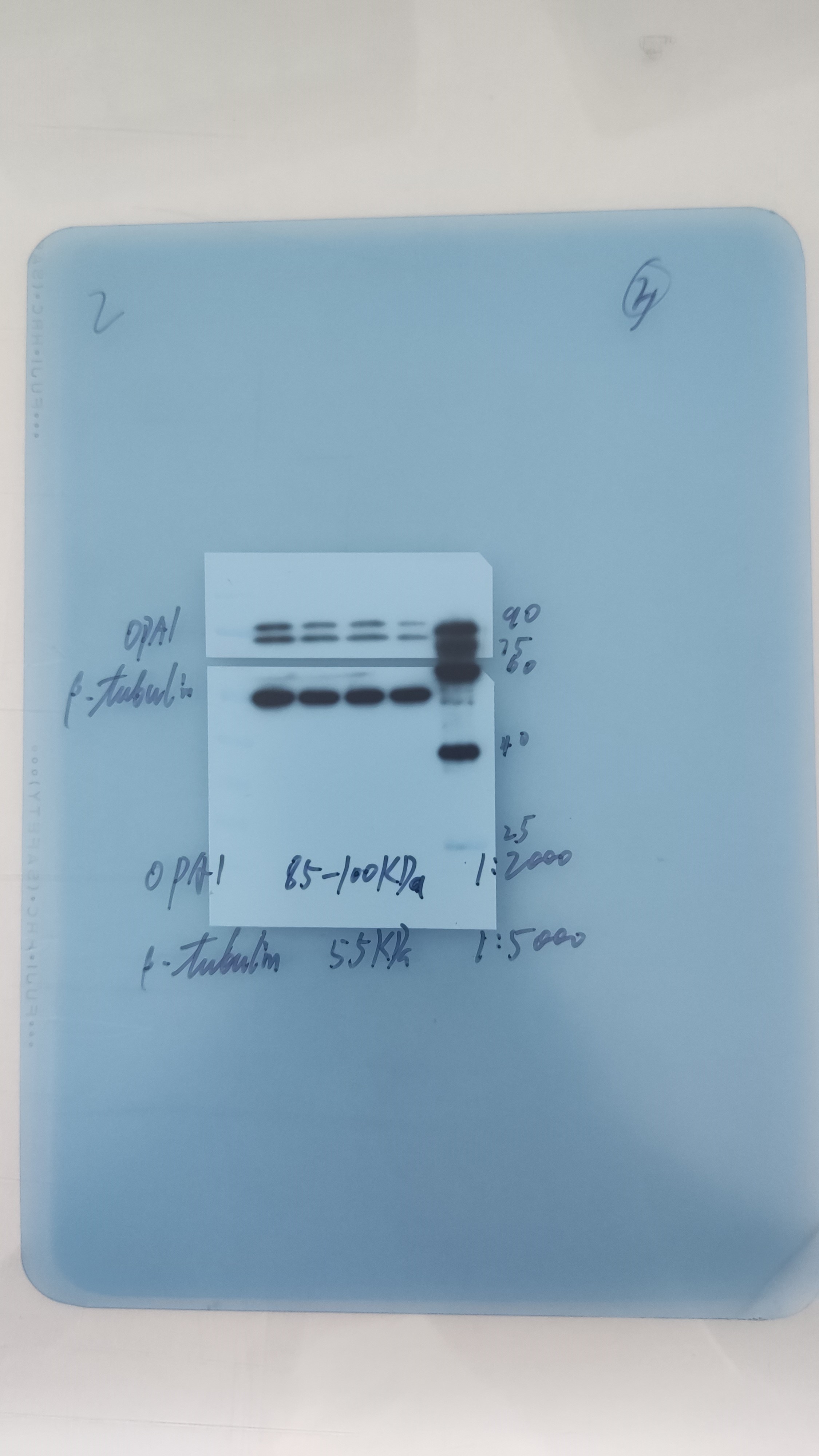

Supplement: Supplementary file 6 [file DataSheet2.zip › WB(1,2)/WB-2/╜║╞1⁄42/5.jpg]

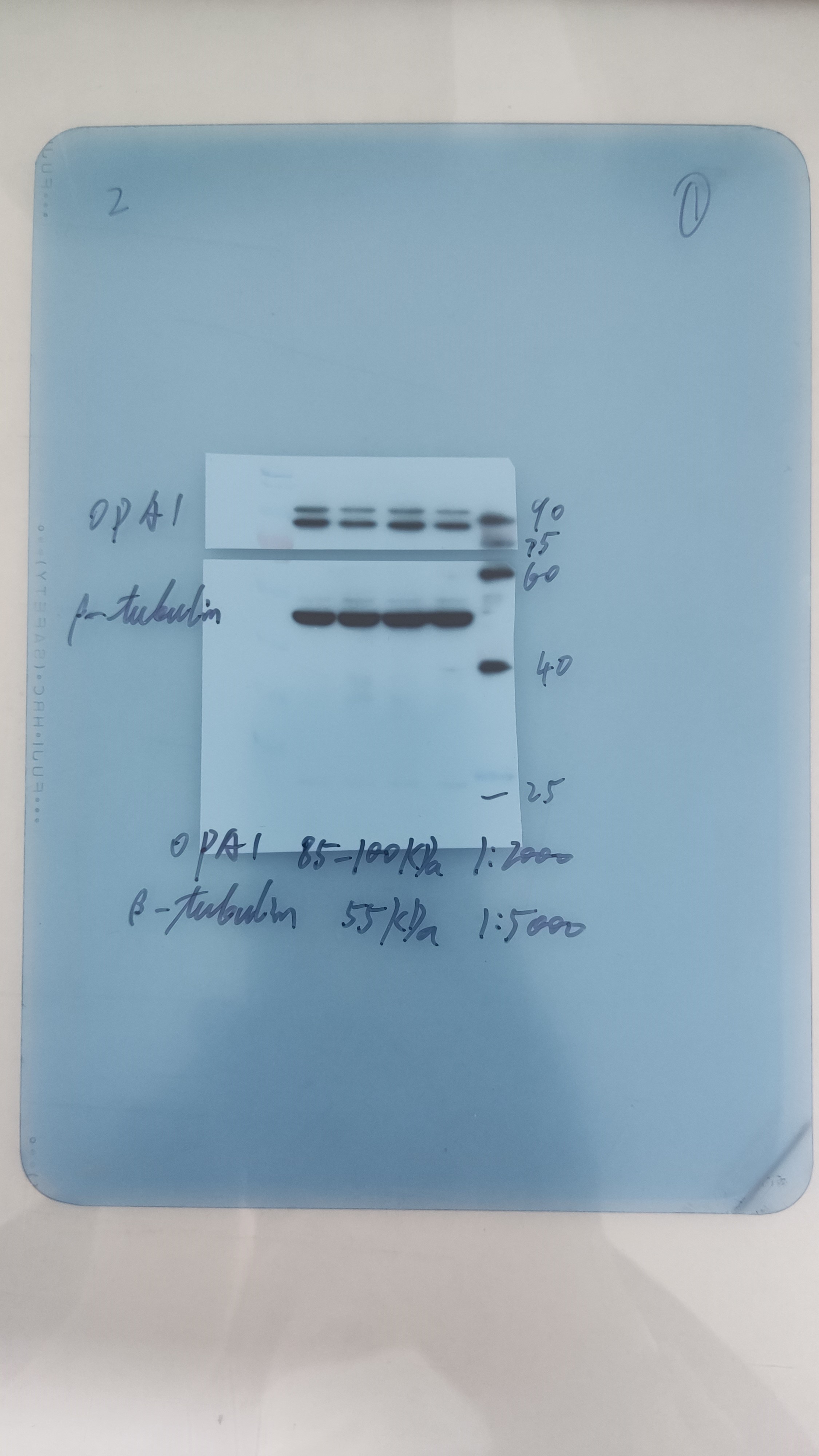

Supplement: Supplementary file 6 [file DataSheet2.zip › WB(1,2)/WB-2/╜║╞1⁄42/6.jpg]

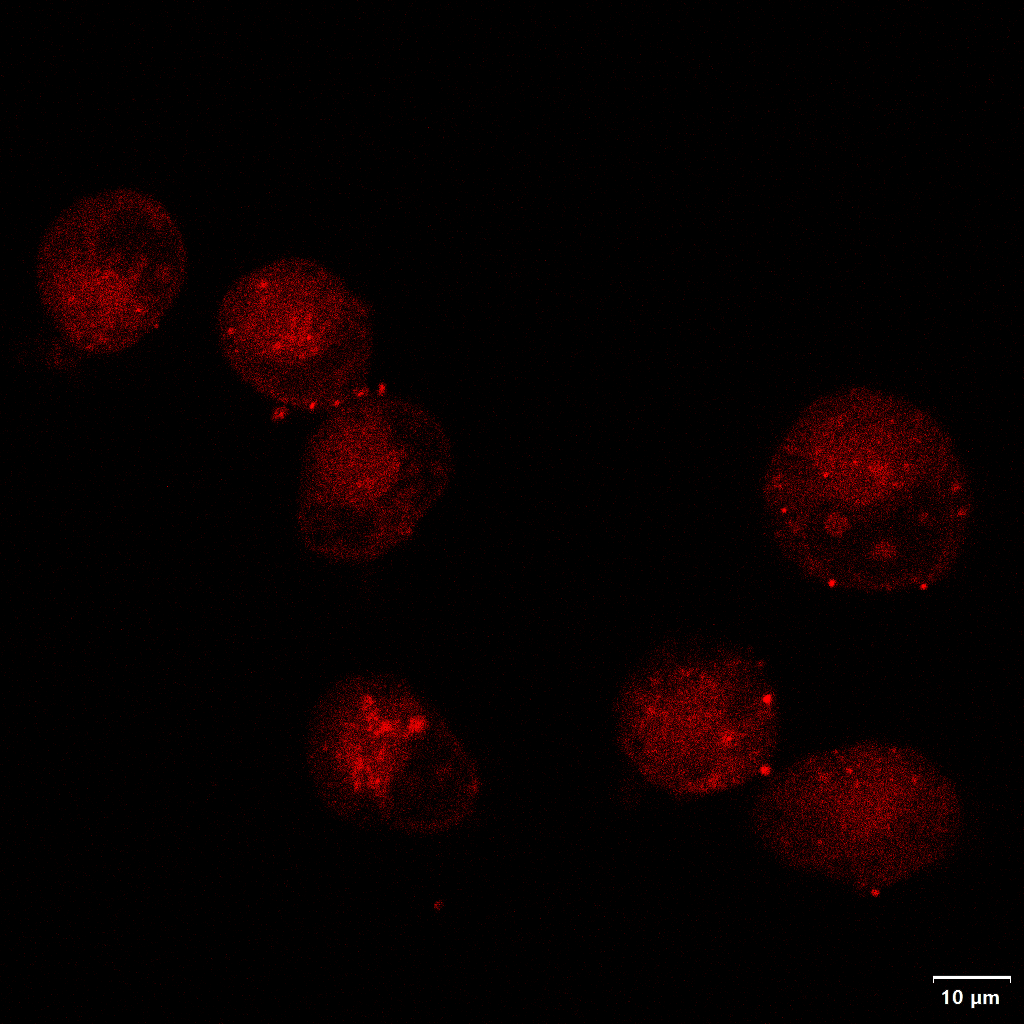

Supplement: Supplementary file 7 [file DataSheet5.zip › Rhod 2AM(1,2)/Rhod 2AM-1/Rhod═╝╞1⁄4/Control Rhod 1.tif]

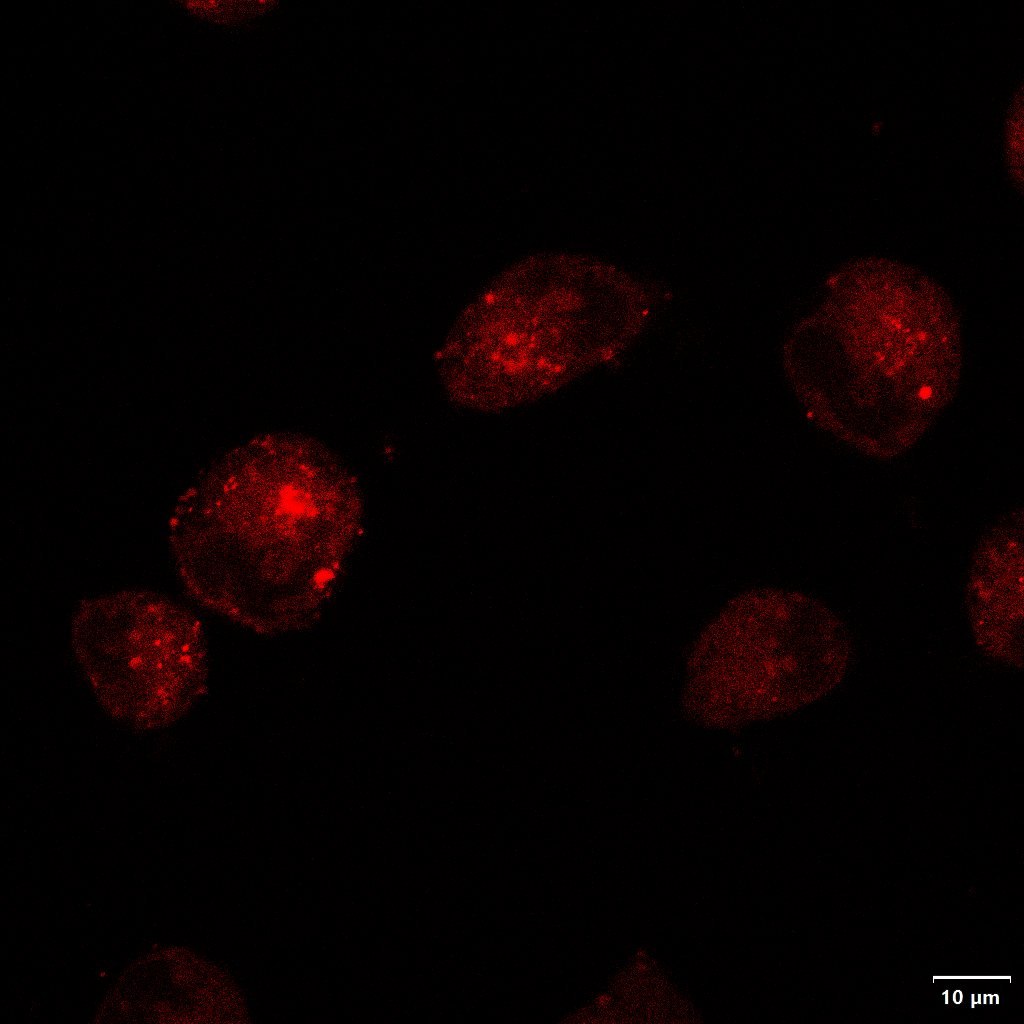

Supplement: Supplementary file 7 [file DataSheet5.zip › Rhod 2AM(1,2)/Rhod 2AM-1/Rhod═╝╞1⁄4/Control Rhod 2.tif]

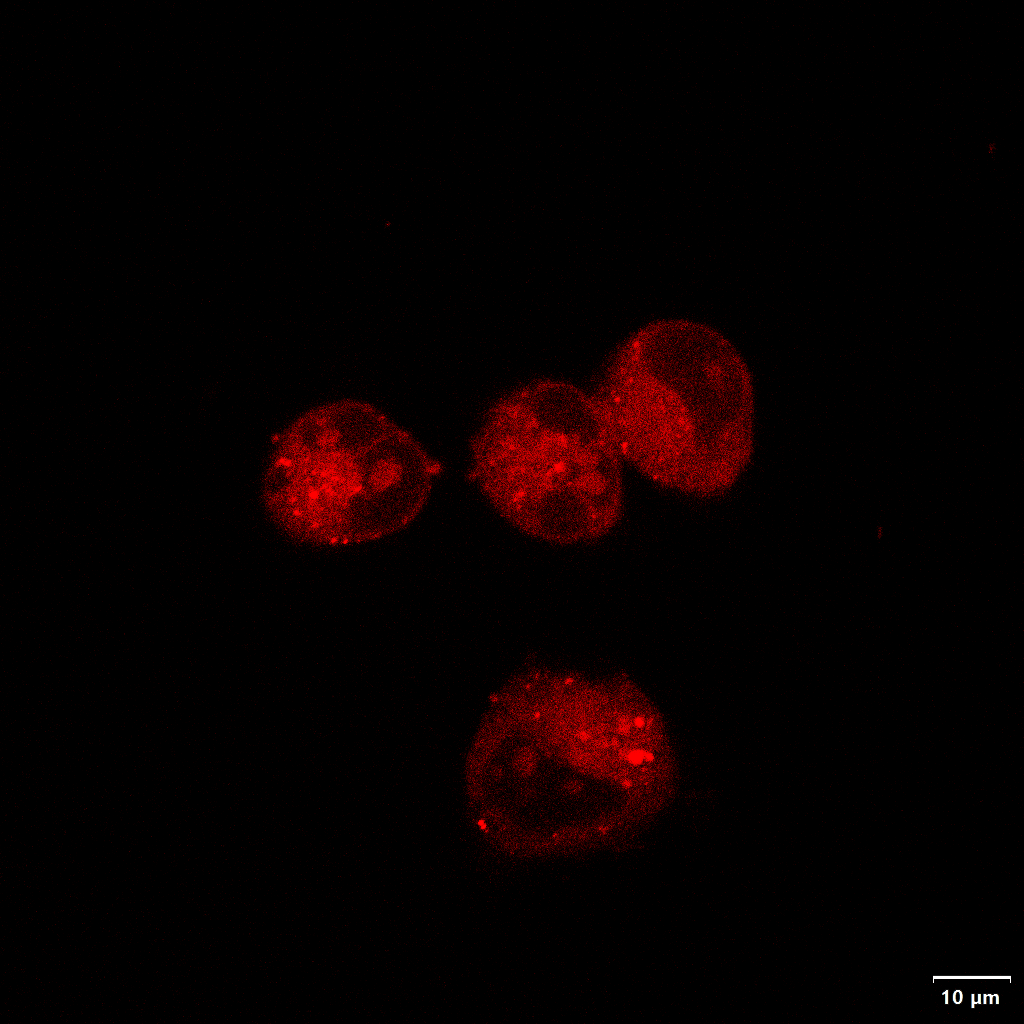

Supplement: Supplementary file 7 [file DataSheet5.zip › Rhod 2AM(1,2)/Rhod 2AM-1/Rhod═╝╞1⁄4/Control Rhod 3.tif]

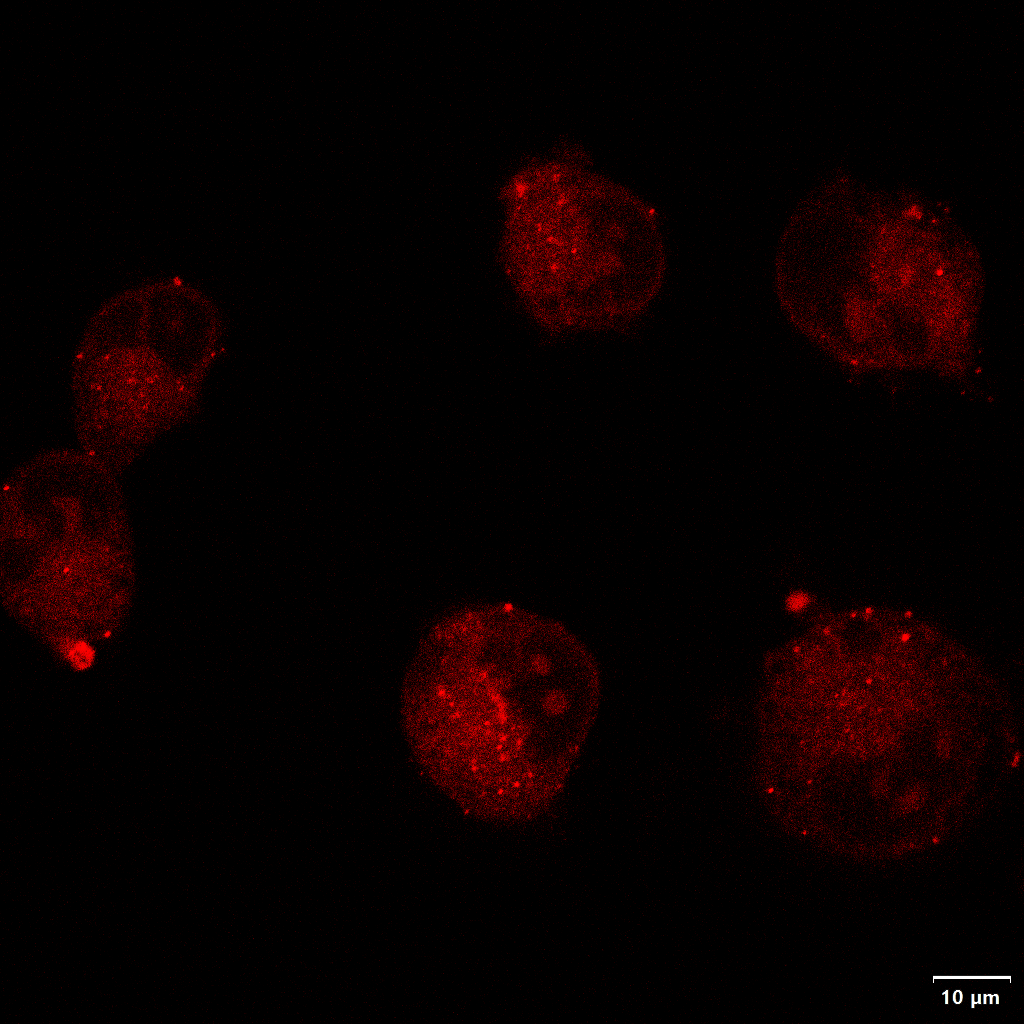

Supplement: Supplementary file 7 [file DataSheet5.zip › Rhod 2AM(1,2)/Rhod 2AM-1/Rhod═╝╞1⁄4/Control Rhod 4.tif]

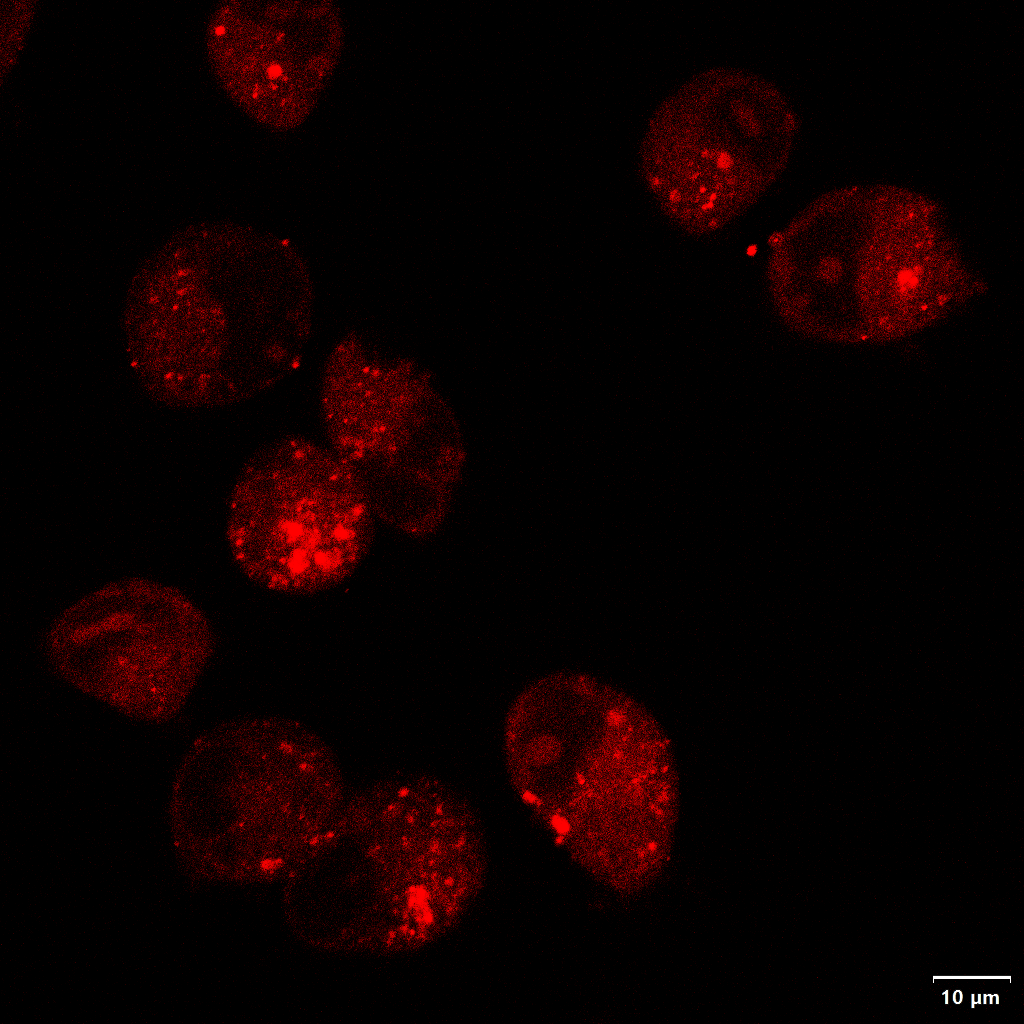

Supplement: Supplementary file 7 [file DataSheet5.zip › Rhod 2AM(1,2)/Rhod 2AM-1/Rhod═╝╞1⁄4/Control Rhod 5.tif]

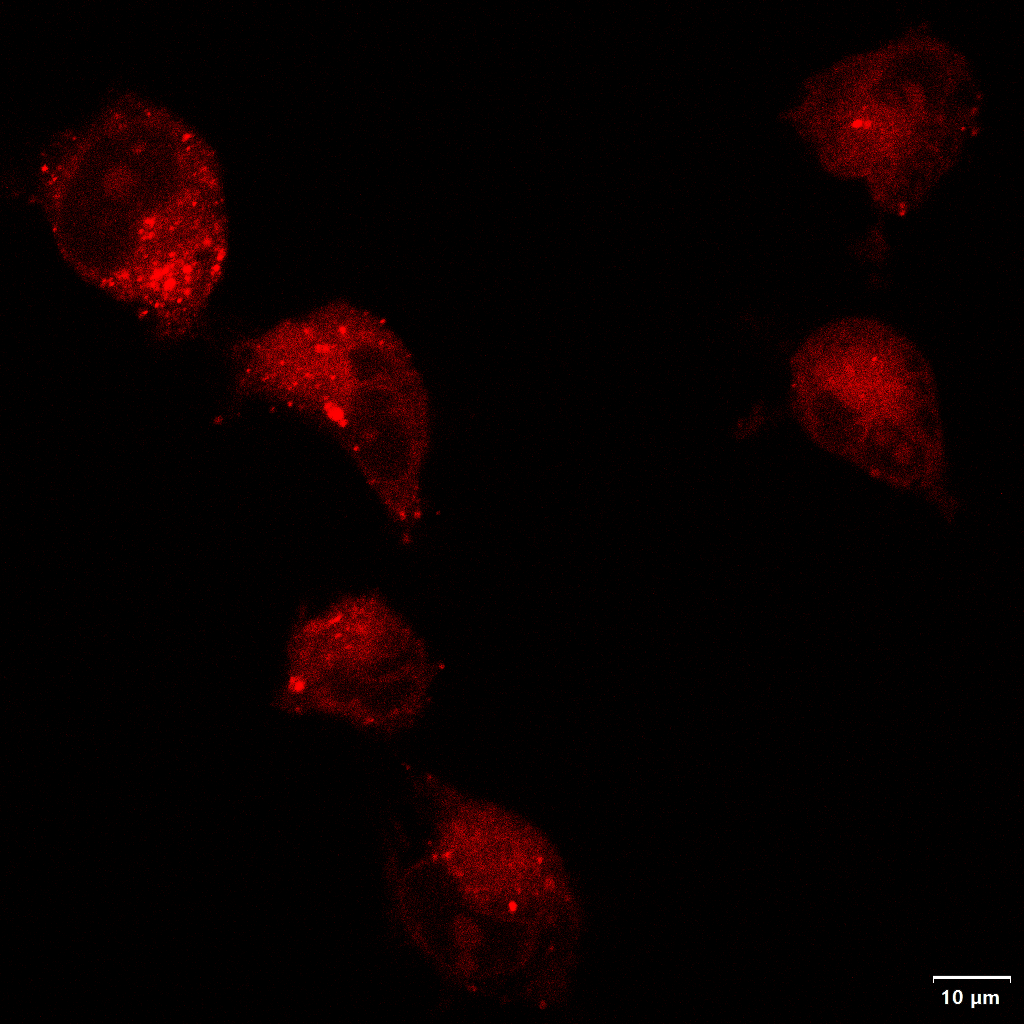

Supplement: Supplementary file 7 [file DataSheet5.zip › Rhod 2AM(1,2)/Rhod 2AM-1/Rhod═╝╞1⁄4/Control Rhod 6.tif]

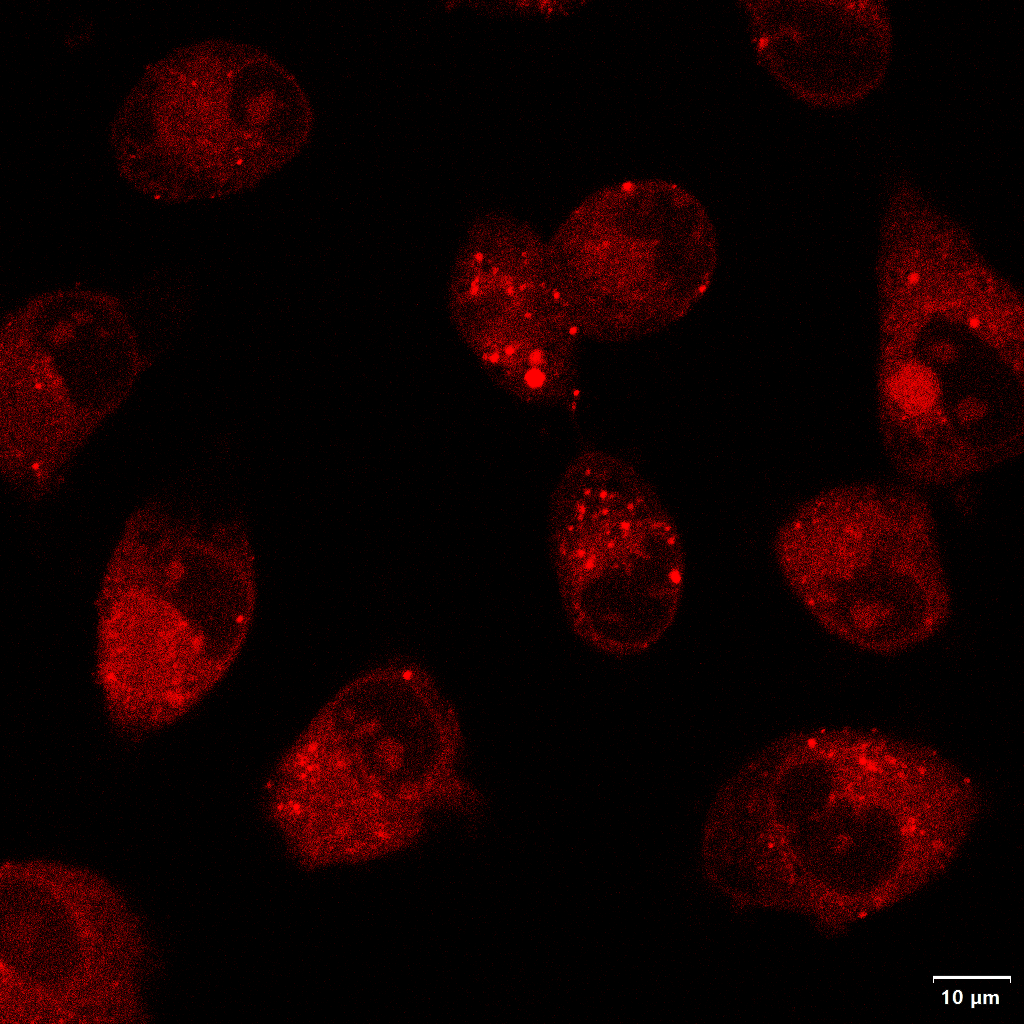

Supplement: Supplementary file 7 [file DataSheet5.zip › Rhod 2AM(1,2)/Rhod 2AM-1/Rhod═╝╞1⁄4/Iohexol 12h Rhod 1.tif]

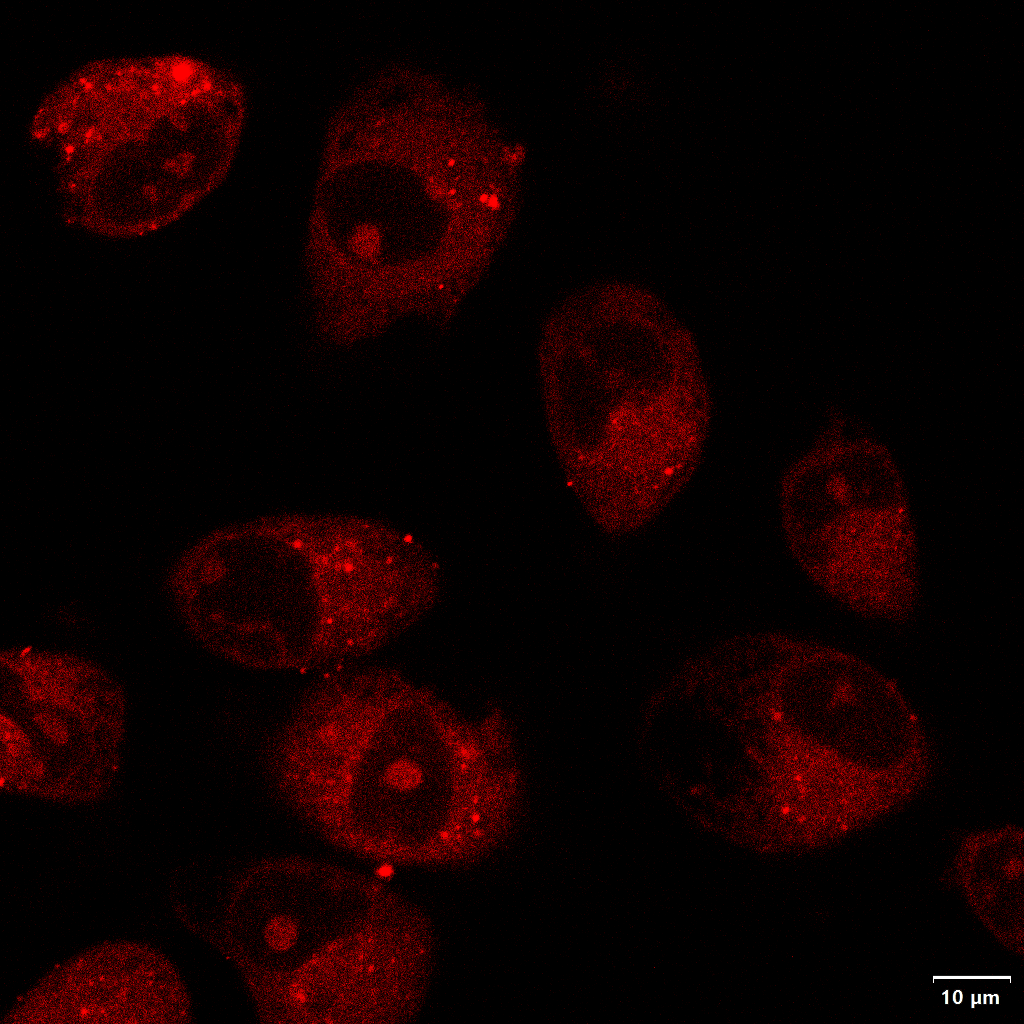

Supplement: Supplementary file 7 [file DataSheet5.zip › Rhod 2AM(1,2)/Rhod 2AM-1/Rhod═╝╞1⁄4/Iohexol 12h Rhod 2.tif]

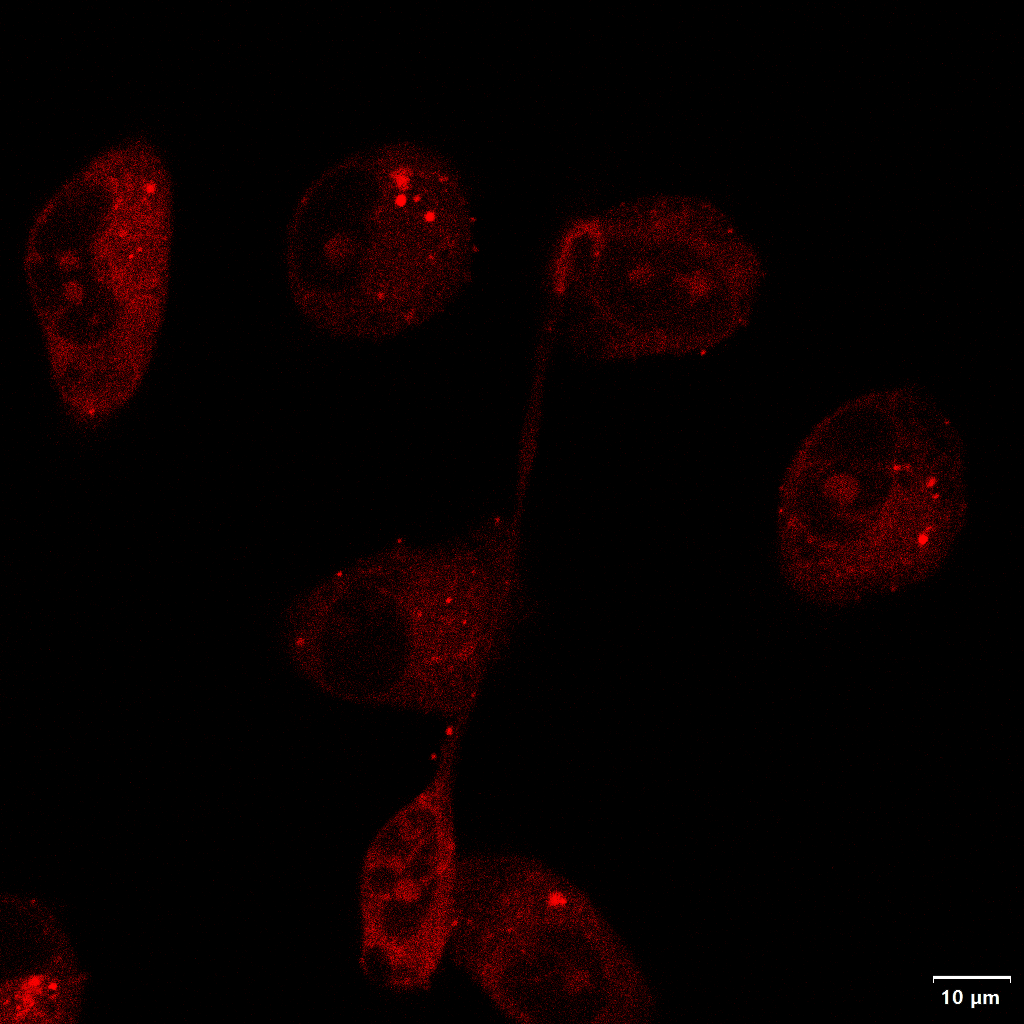

Supplement: Supplementary file 7 [file DataSheet5.zip › Rhod 2AM(1,2)/Rhod 2AM-1/Rhod═╝╞1⁄4/Iohexol 12h Rhod 3.tif]

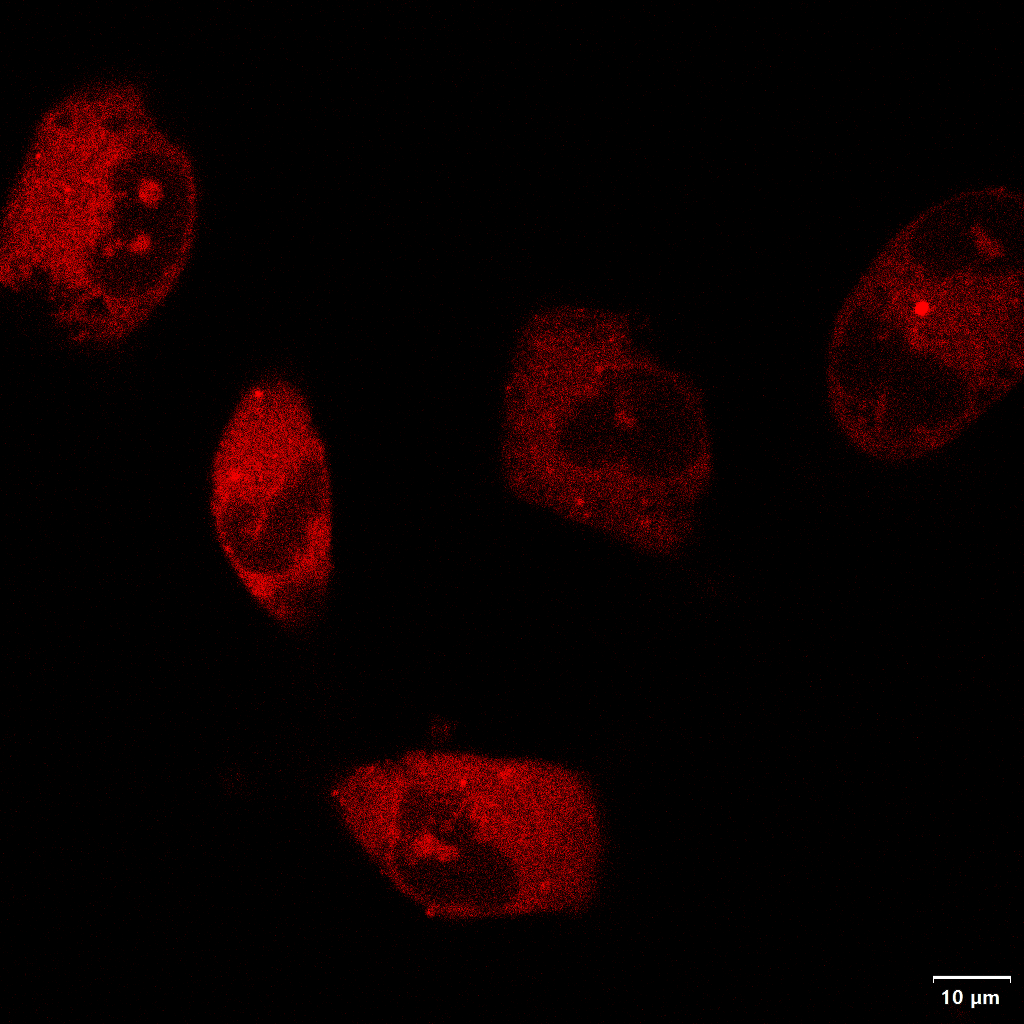

Supplement: Supplementary file 7 [file DataSheet5.zip › Rhod 2AM(1,2)/Rhod 2AM-1/Rhod═╝╞1⁄4/Iohexol 12h Rhod 4.tif]

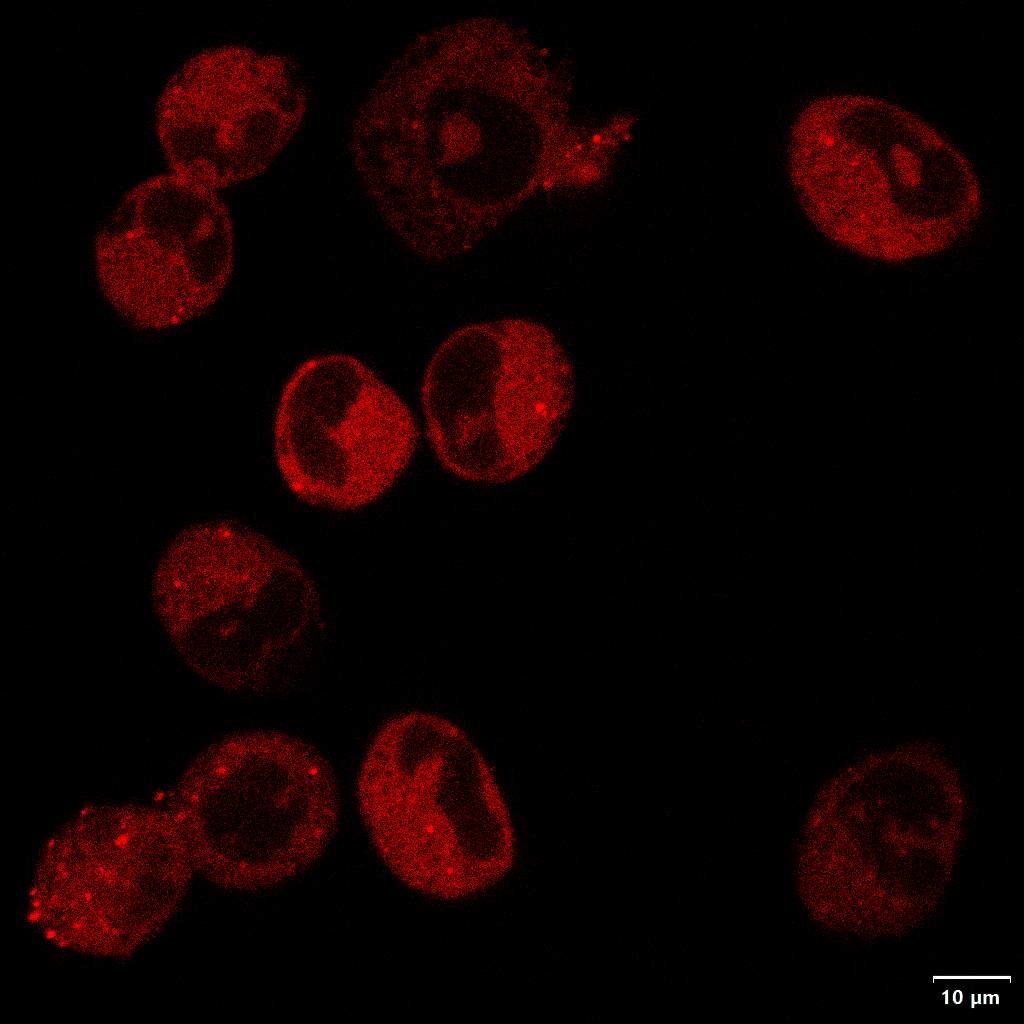

Supplement: Supplementary file 7 [file DataSheet5.zip › Rhod 2AM(1,2)/Rhod 2AM-1/Rhod═╝╞1⁄4/Iohexol 12h Rhod 5.tif]

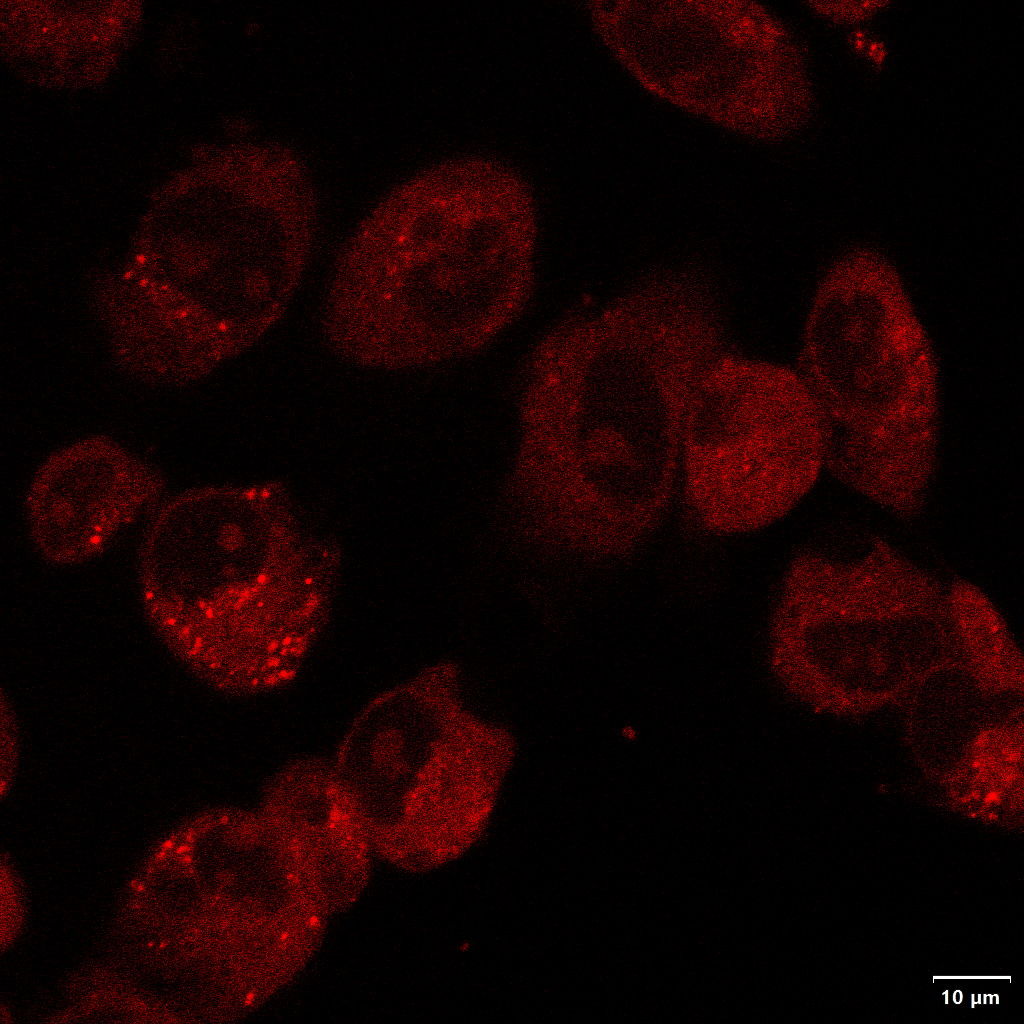

Supplement: Supplementary file 7 [file DataSheet5.zip › Rhod 2AM(1,2)/Rhod 2AM-1/Rhod═╝╞1⁄4/Iohexol 12h Rhod 6.tif]

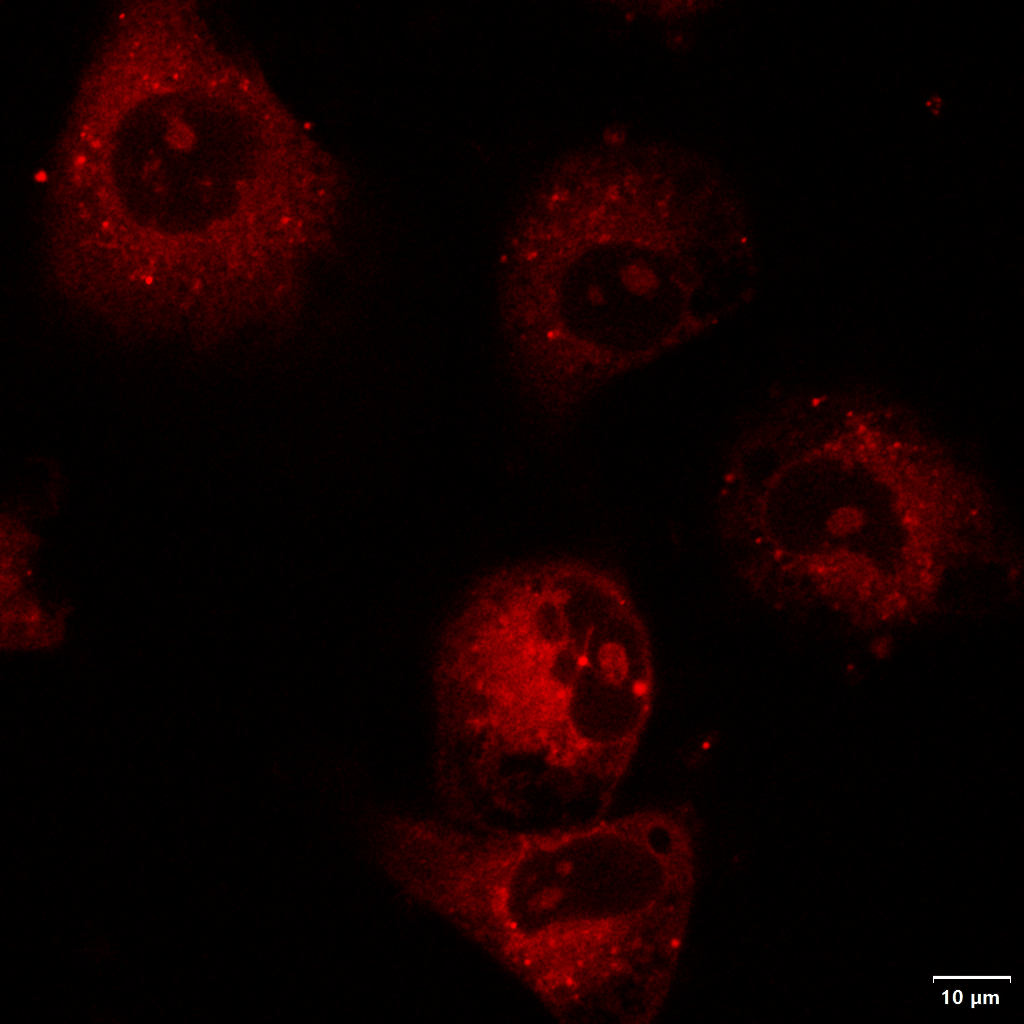

Supplement: Supplementary file 7 [file DataSheet5.zip › Rhod 2AM(1,2)/Rhod 2AM-1/Rhod═╝╞1⁄4/Iohexol 4h Rhod 1.tif]

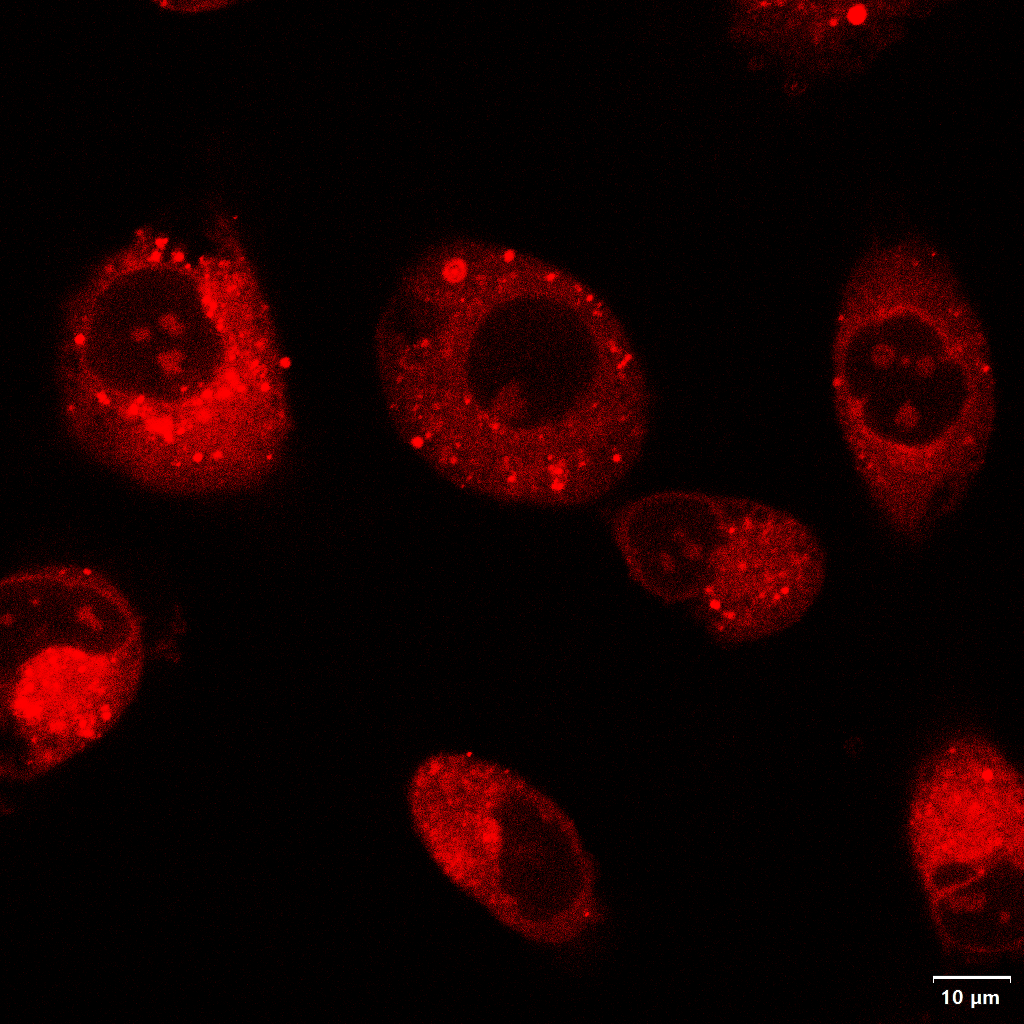

Supplement: Supplementary file 7 [file DataSheet5.zip › Rhod 2AM(1,2)/Rhod 2AM-1/Rhod═╝╞1⁄4/Iohexol 4h Rhod 2.tif]

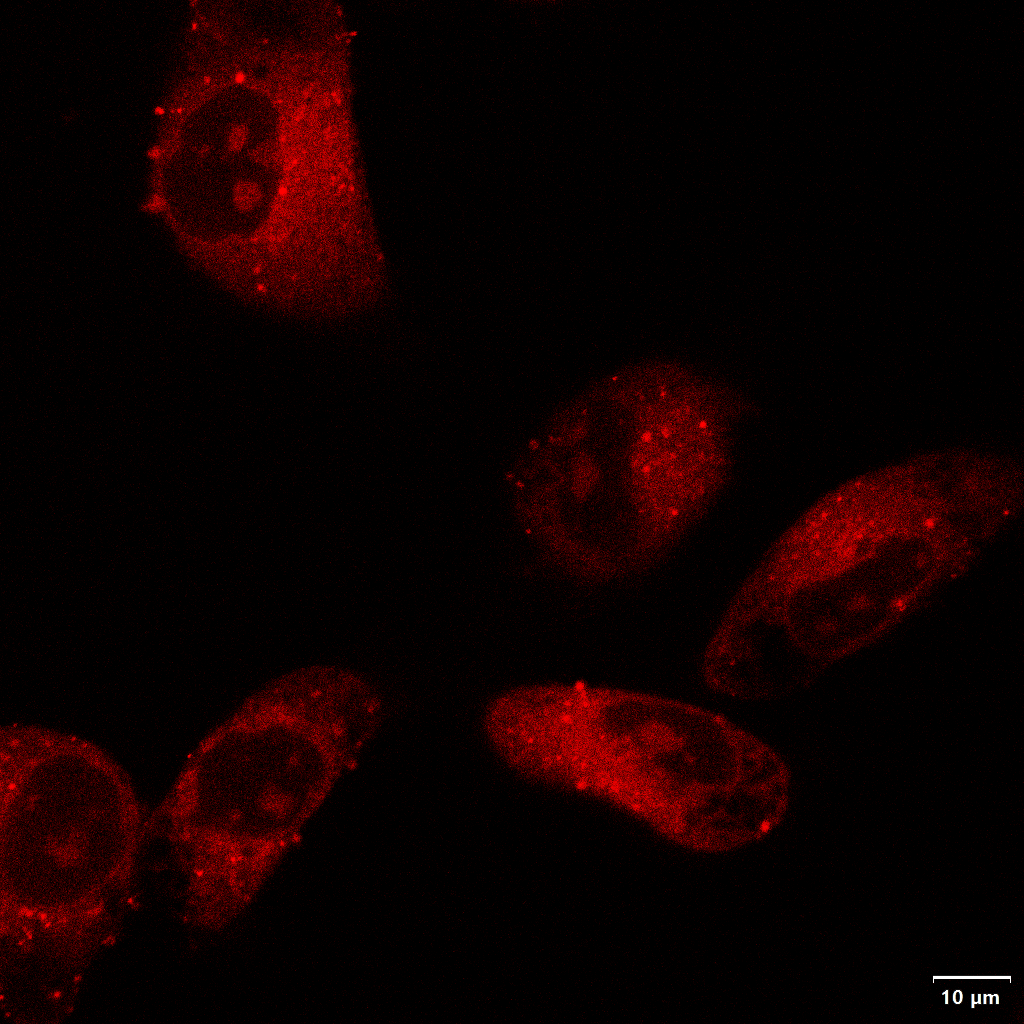

Supplement: Supplementary file 7 [file DataSheet5.zip › Rhod 2AM(1,2)/Rhod 2AM-1/Rhod═╝╞1⁄4/Iohexol 4h Rhod 3.tif]

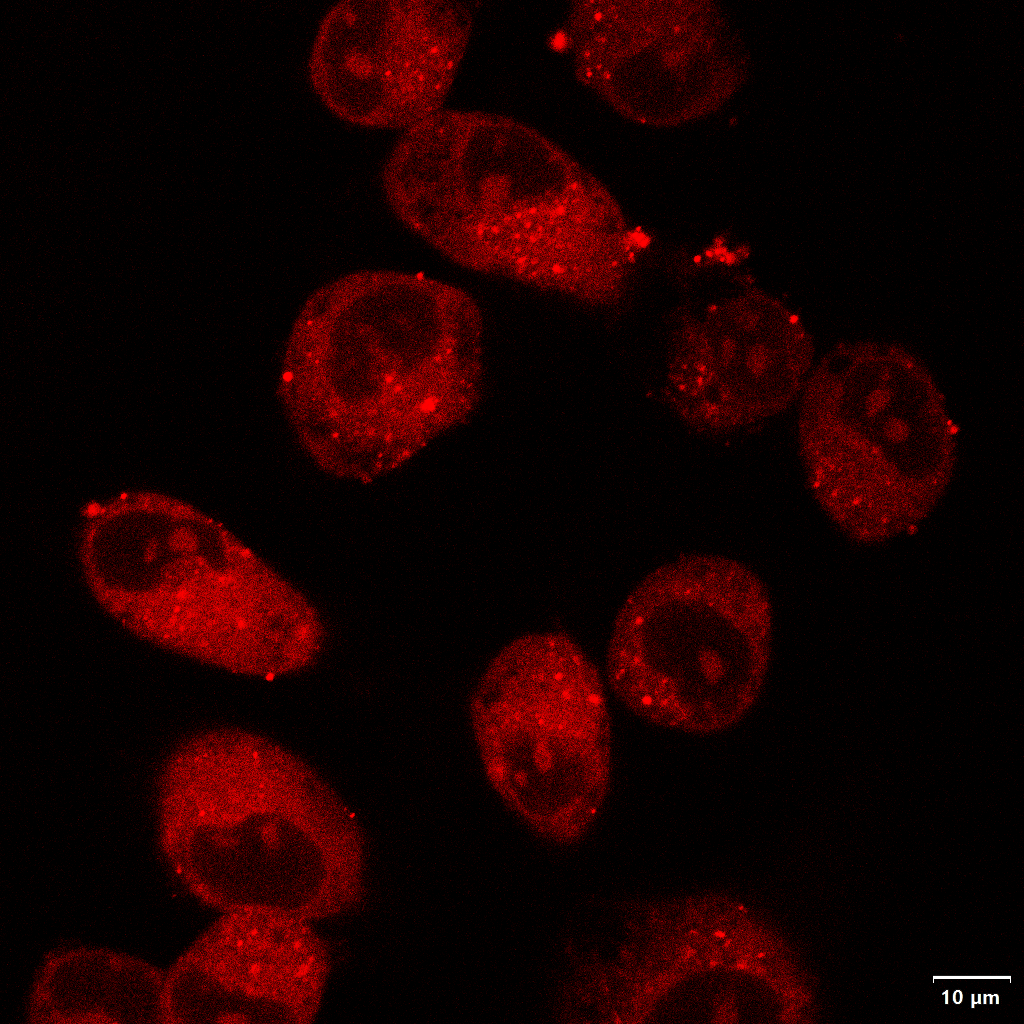

Supplement: Supplementary file 7 [file DataSheet5.zip › Rhod 2AM(1,2)/Rhod 2AM-1/Rhod═╝╞1⁄4/Iohexol 4h Rhod 4.tif]

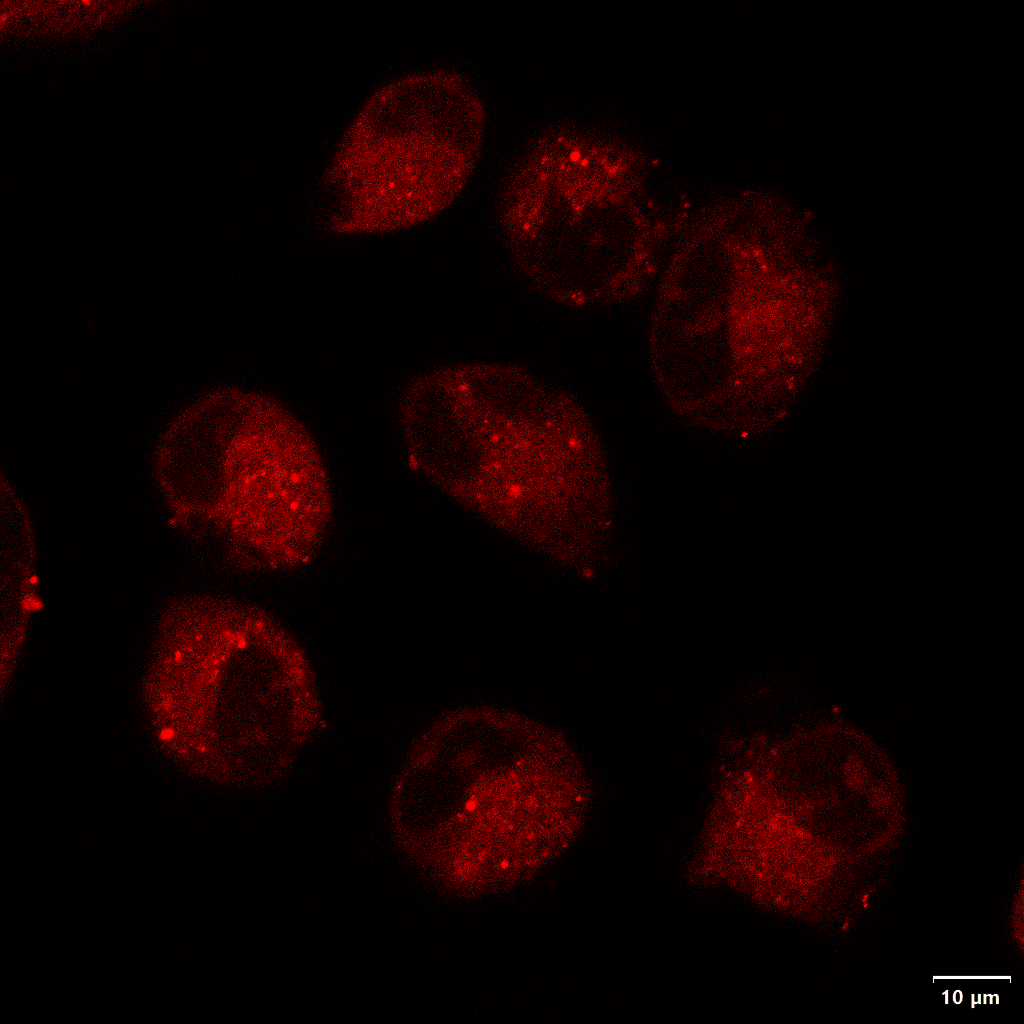

Supplement: Supplementary file 7 [file DataSheet5.zip › Rhod 2AM(1,2)/Rhod 2AM-1/Rhod═╝╞1⁄4/Iohexol 4h Rhod 5.tif]

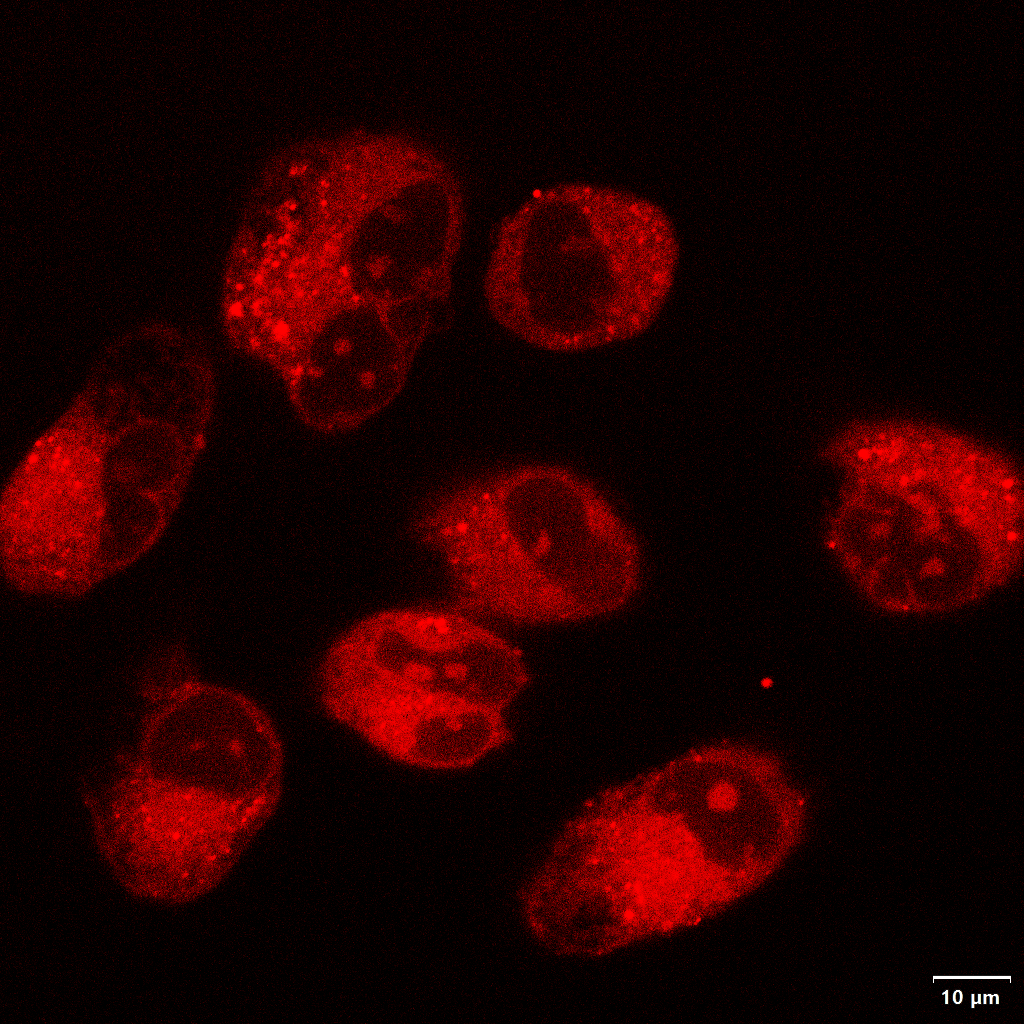

Supplement: Supplementary file 7 [file DataSheet5.zip › Rhod 2AM(1,2)/Rhod 2AM-1/Rhod═╝╞1⁄4/Iohexol 4h Rhod 6.tif]

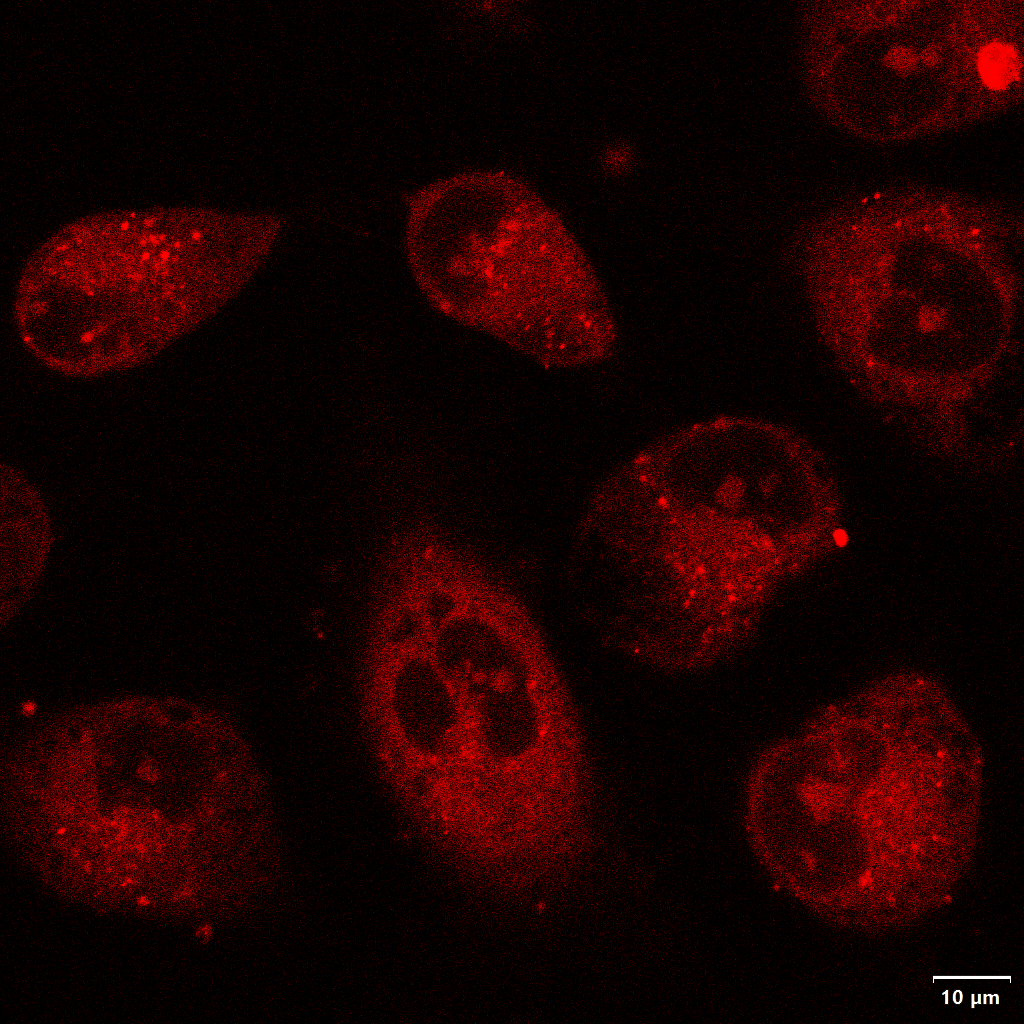

Supplement: Supplementary file 7 [file DataSheet5.zip › Rhod 2AM(1,2)/Rhod 2AM-1/Rhod═╝╞1⁄4/Iohexol 8h Rhod 1.tif]

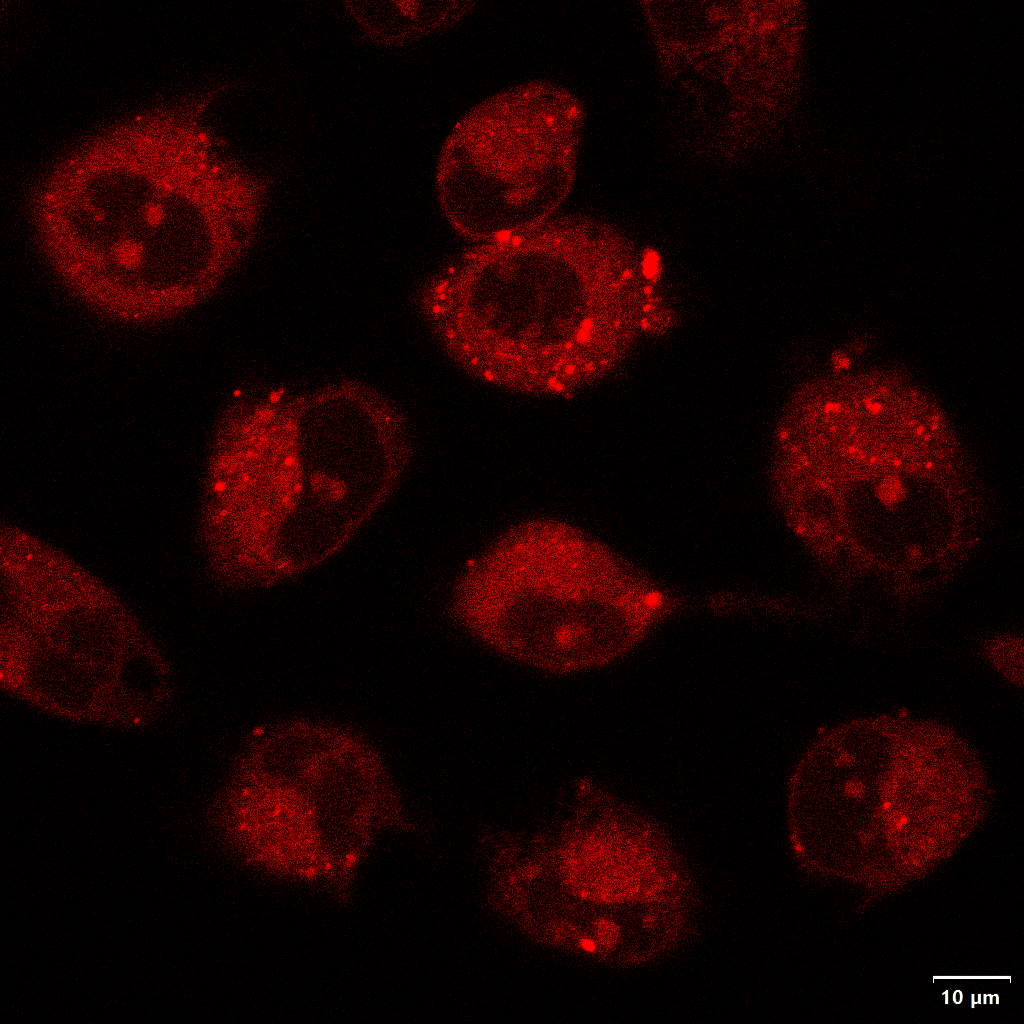

Supplement: Supplementary file 7 [file DataSheet5.zip › Rhod 2AM(1,2)/Rhod 2AM-1/Rhod═╝╞1⁄4/Iohexol 8h Rhod 2.tif]

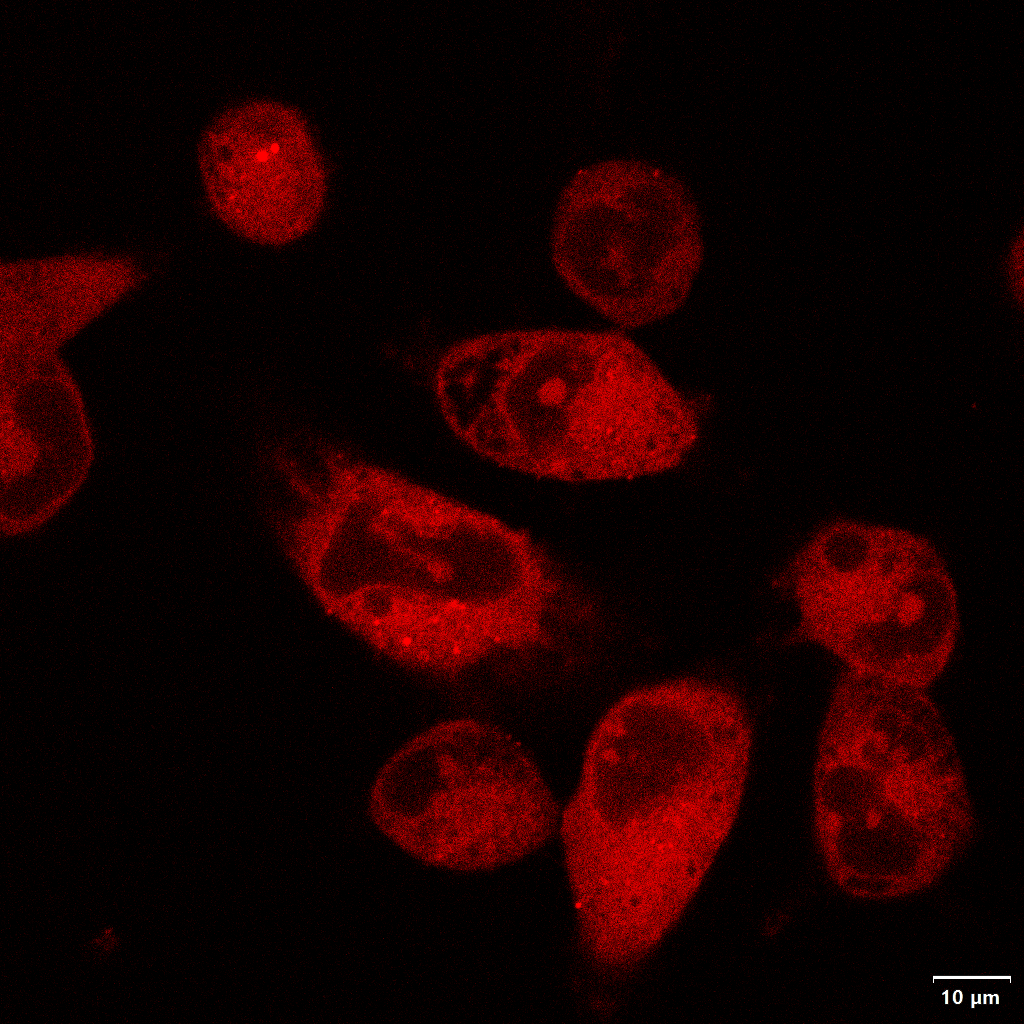

Supplement: Supplementary file 7 [file DataSheet5.zip › Rhod 2AM(1,2)/Rhod 2AM-1/Rhod═╝╞1⁄4/Iohexol 8h Rhod 3.tif]

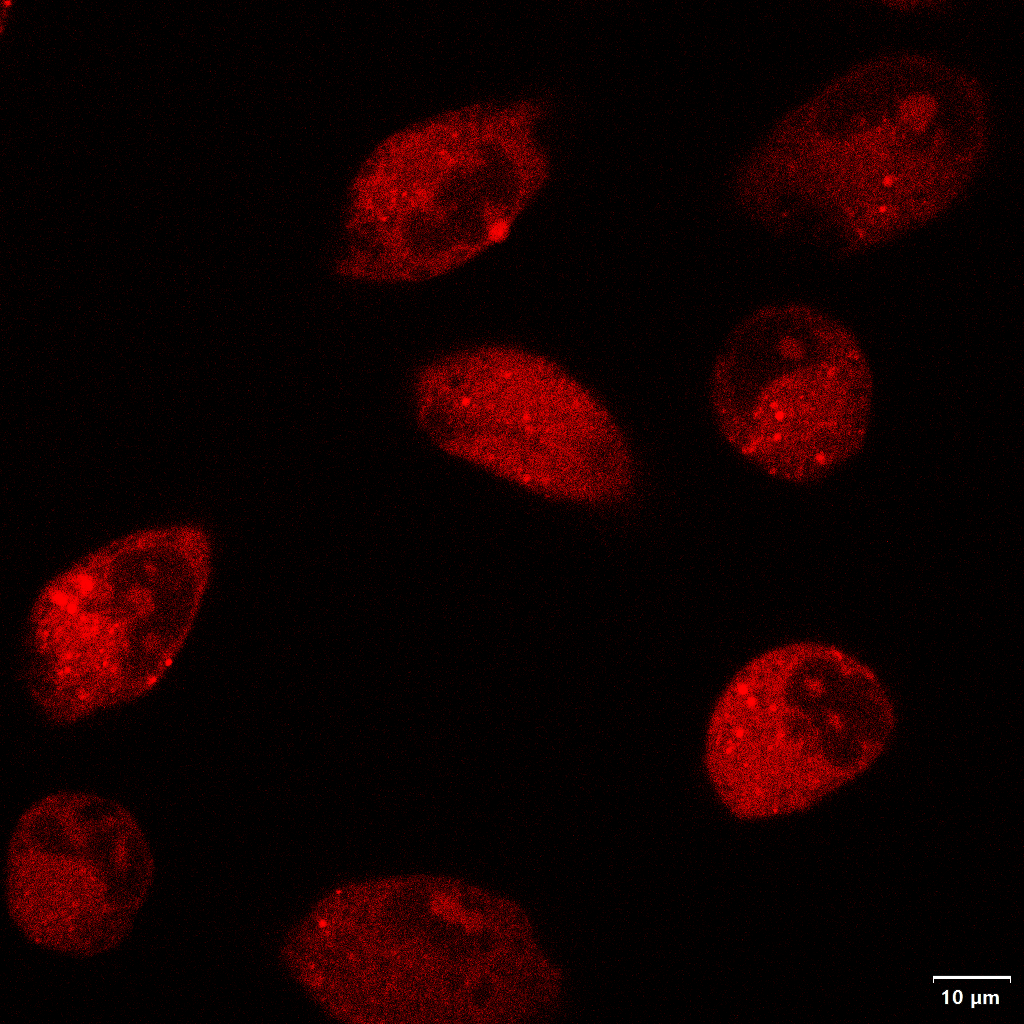

Supplement: Supplementary file 7 [file DataSheet5.zip › Rhod 2AM(1,2)/Rhod 2AM-1/Rhod═╝╞1⁄4/Iohexol 8h Rhod 4.tif]

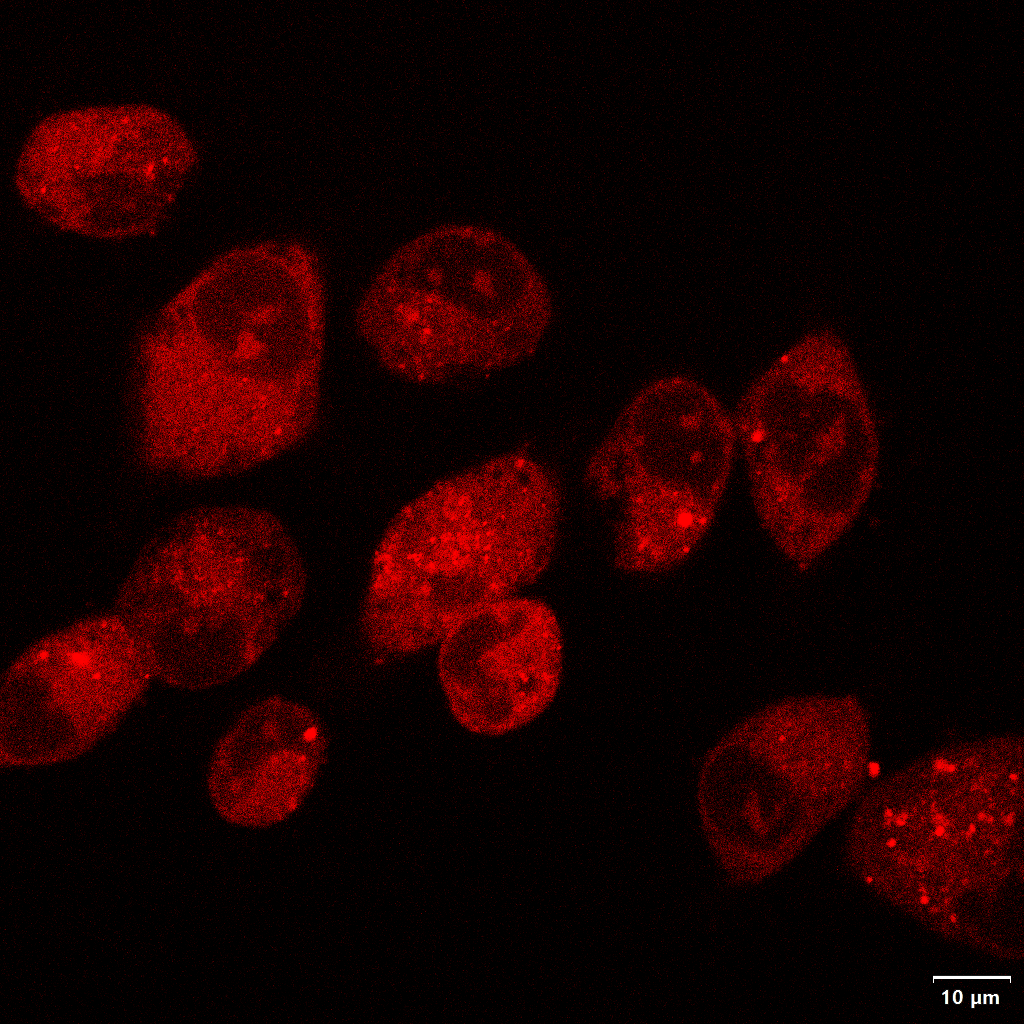

Supplement: Supplementary file 7 [file DataSheet5.zip › Rhod 2AM(1,2)/Rhod 2AM-1/Rhod═╝╞1⁄4/Iohexol 8h Rhod 5.tif]

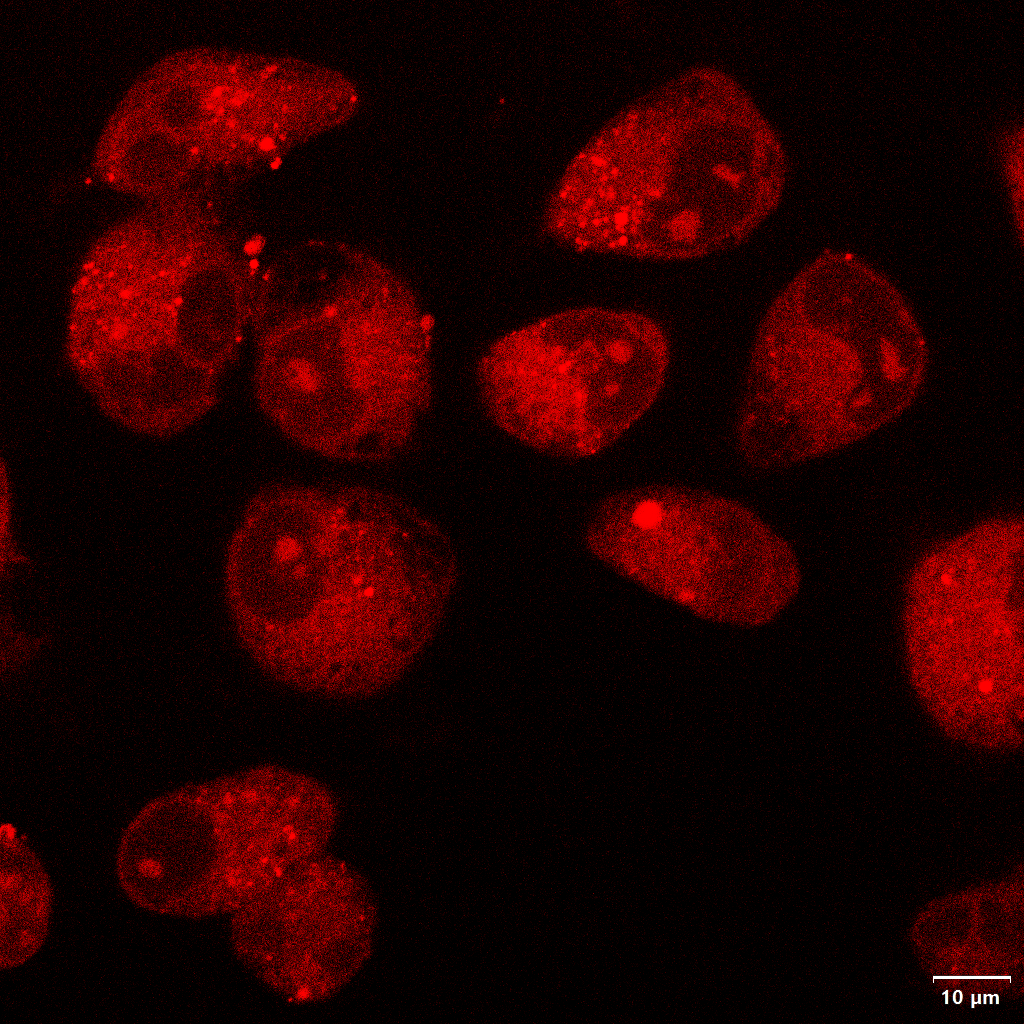

Supplement: Supplementary file 7 [file DataSheet5.zip › Rhod 2AM(1,2)/Rhod 2AM-1/Rhod═╝╞1⁄4/Iohexol 8h Rhod 6.tif]

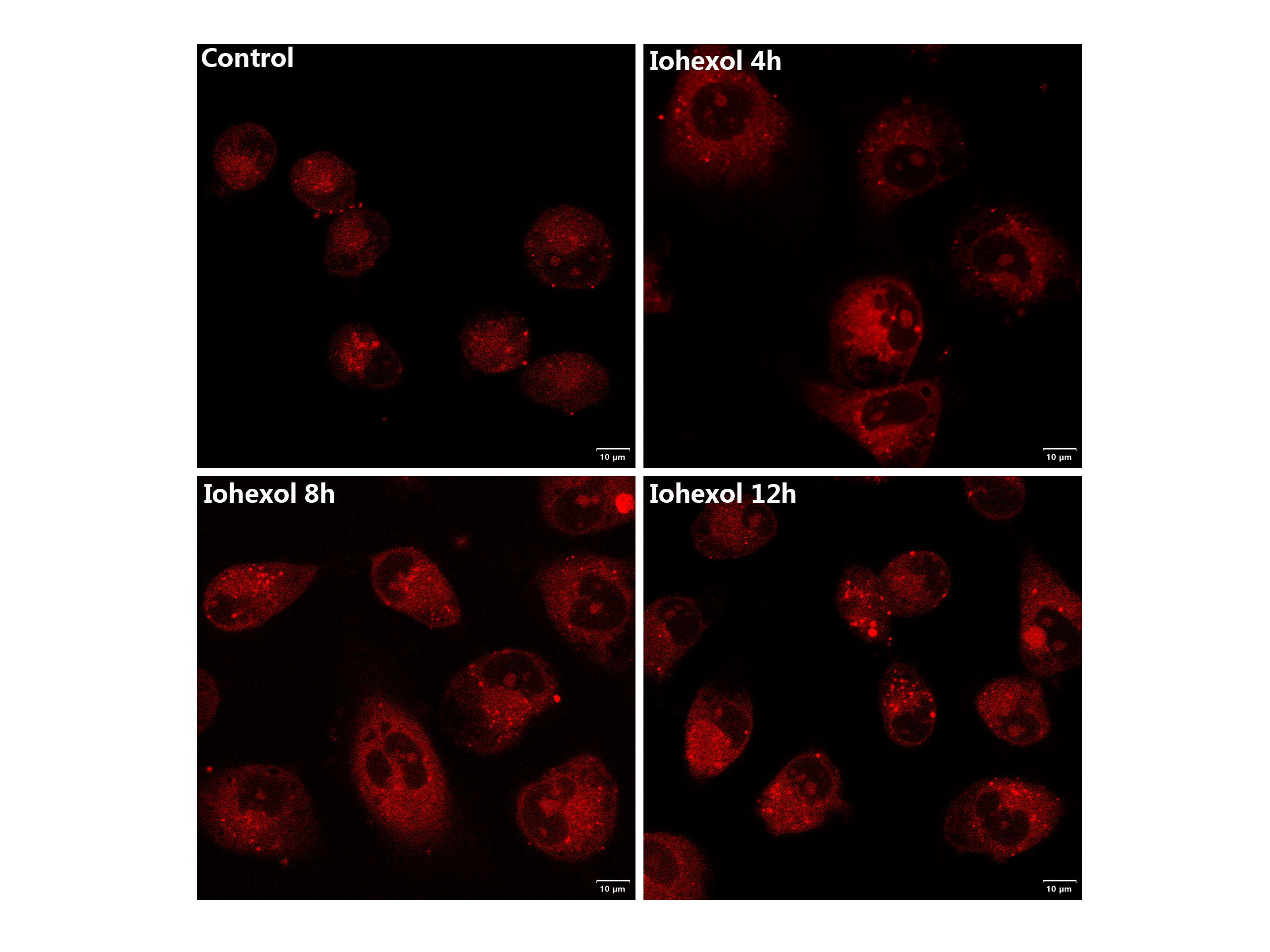

Supplement: Supplementary file 7 [file DataSheet5.zip › Rhod 2AM(1,2)/Rhod 2AM-1/Rhod═╝╞1⁄4/Rhod AM.tif]

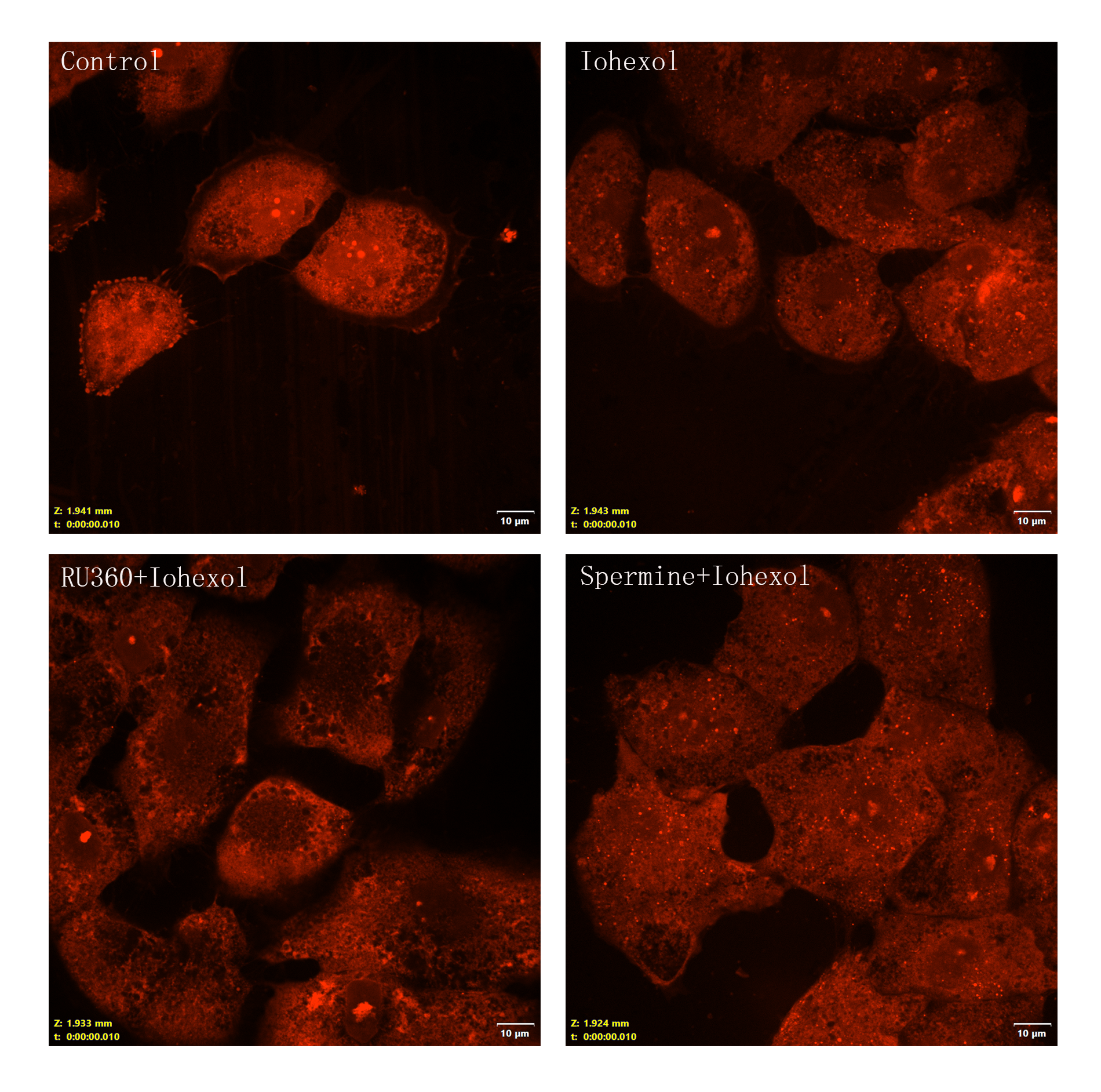

Supplement: Supplementary file 7 [file DataSheet5.zip › Rhod 2AM(1,2)/Rhod-2AM-2/Rhod-2-2═╝╞1⁄4/1.tif]

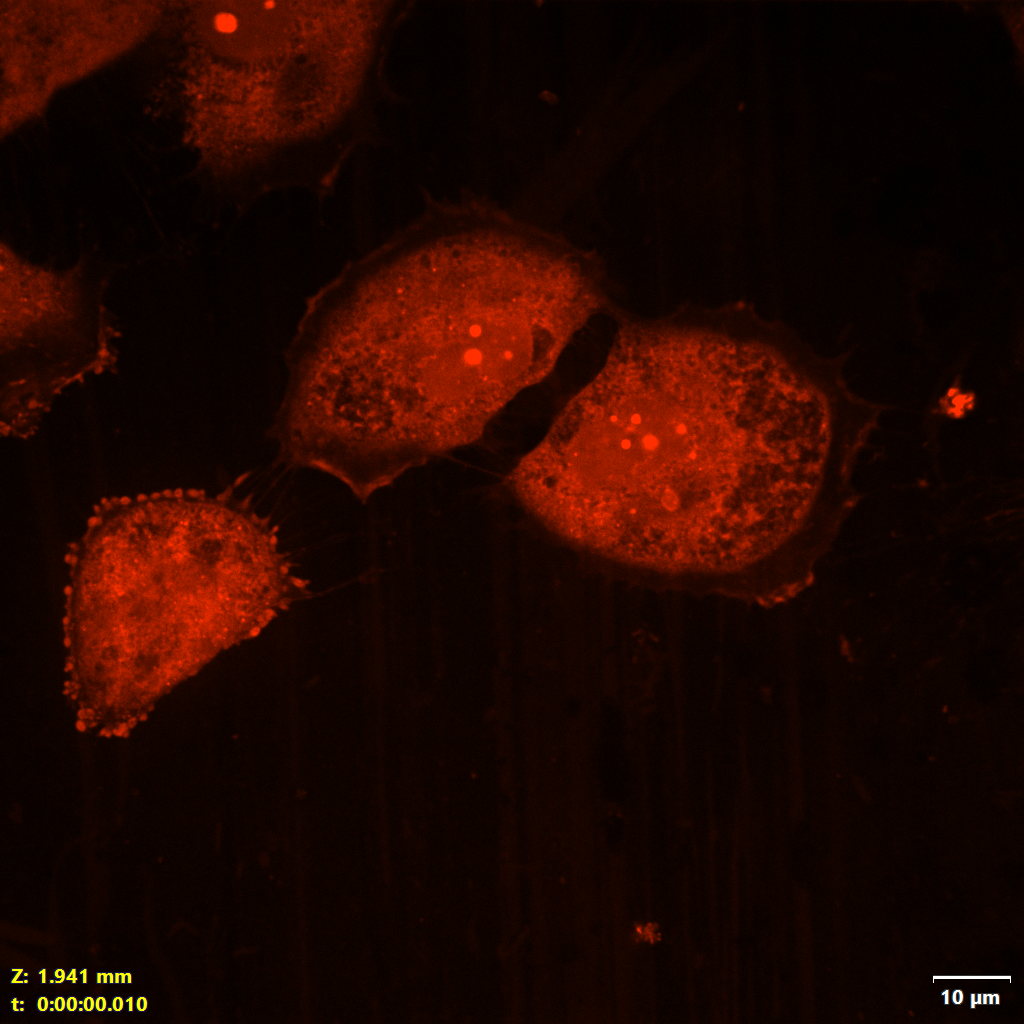

Supplement: Supplementary file 7 [file DataSheet5.zip › Rhod 2AM(1,2)/Rhod-2AM-2/Rhod-2-2═╝╞1⁄4/Control 1.tif]

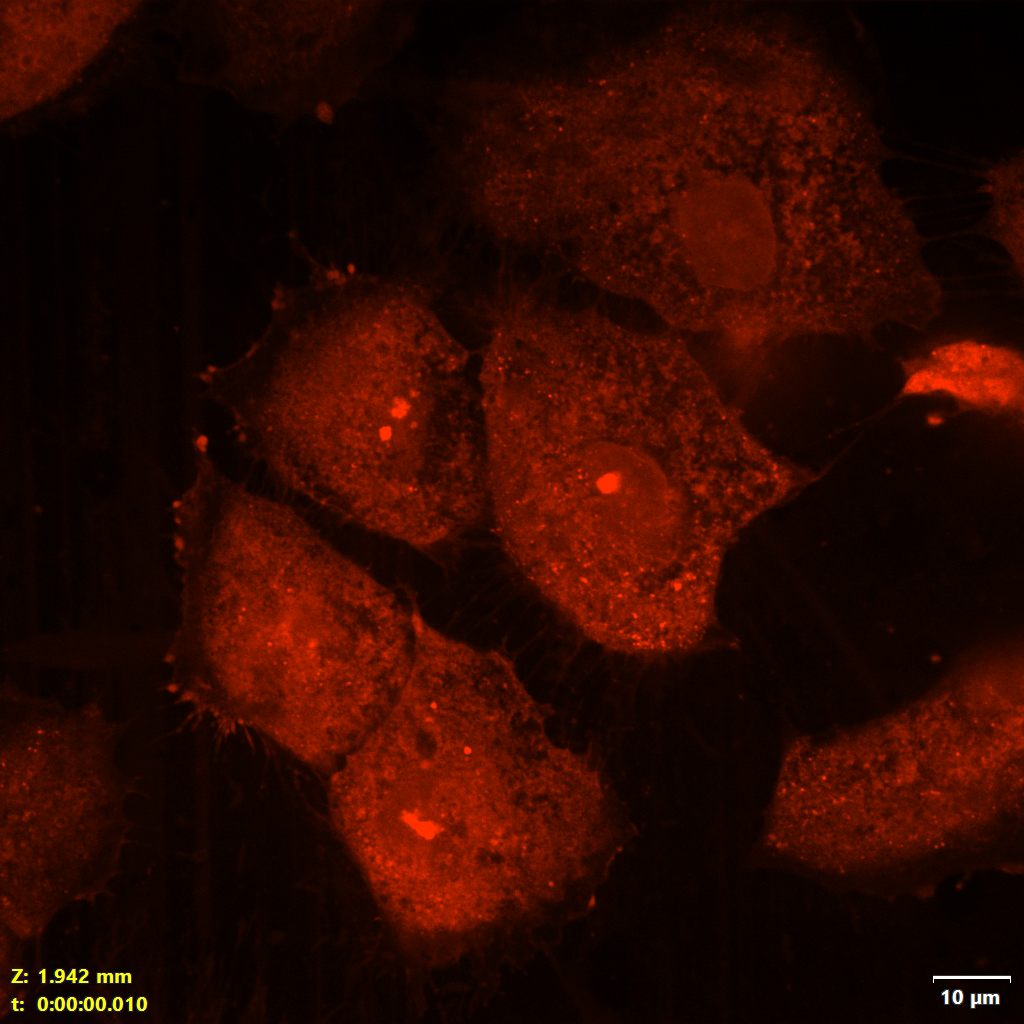

Supplement: Supplementary file 7 [file DataSheet5.zip › Rhod 2AM(1,2)/Rhod-2AM-2/Rhod-2-2═╝╞1⁄4/Control 2.tif]

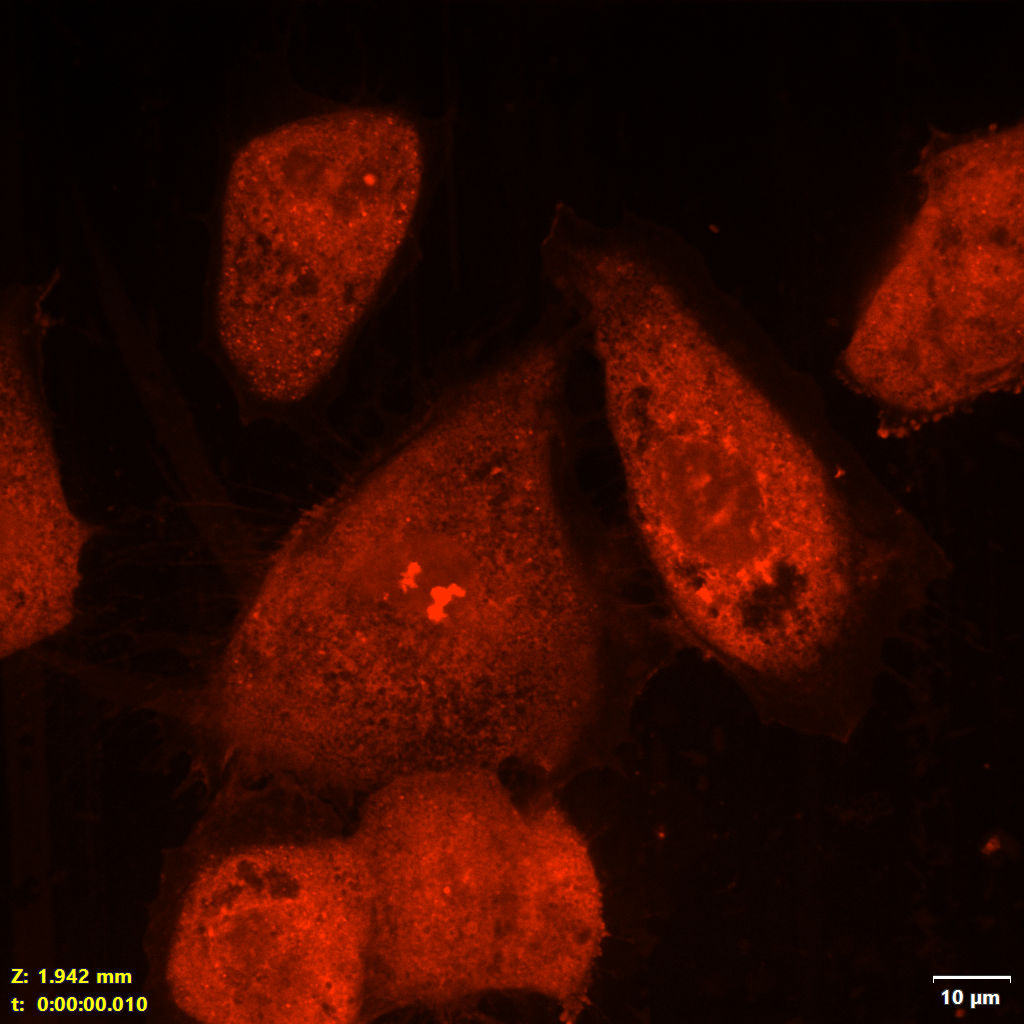

Supplement: Supplementary file 7 [file DataSheet5.zip › Rhod 2AM(1,2)/Rhod-2AM-2/Rhod-2-2═╝╞1⁄4/Control 3.tif]

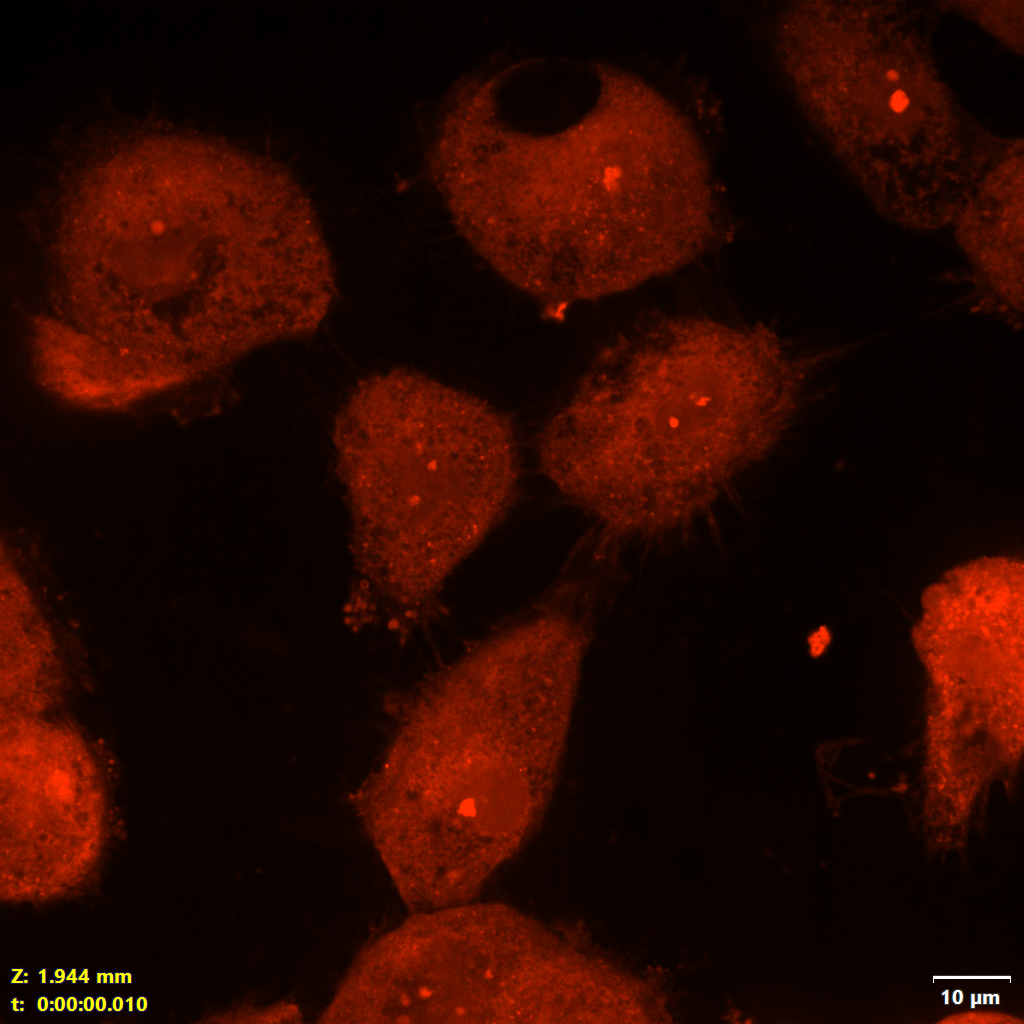

Supplement: Supplementary file 7 [file DataSheet5.zip › Rhod 2AM(1,2)/Rhod-2AM-2/Rhod-2-2═╝╞1⁄4/Control 4.tif]

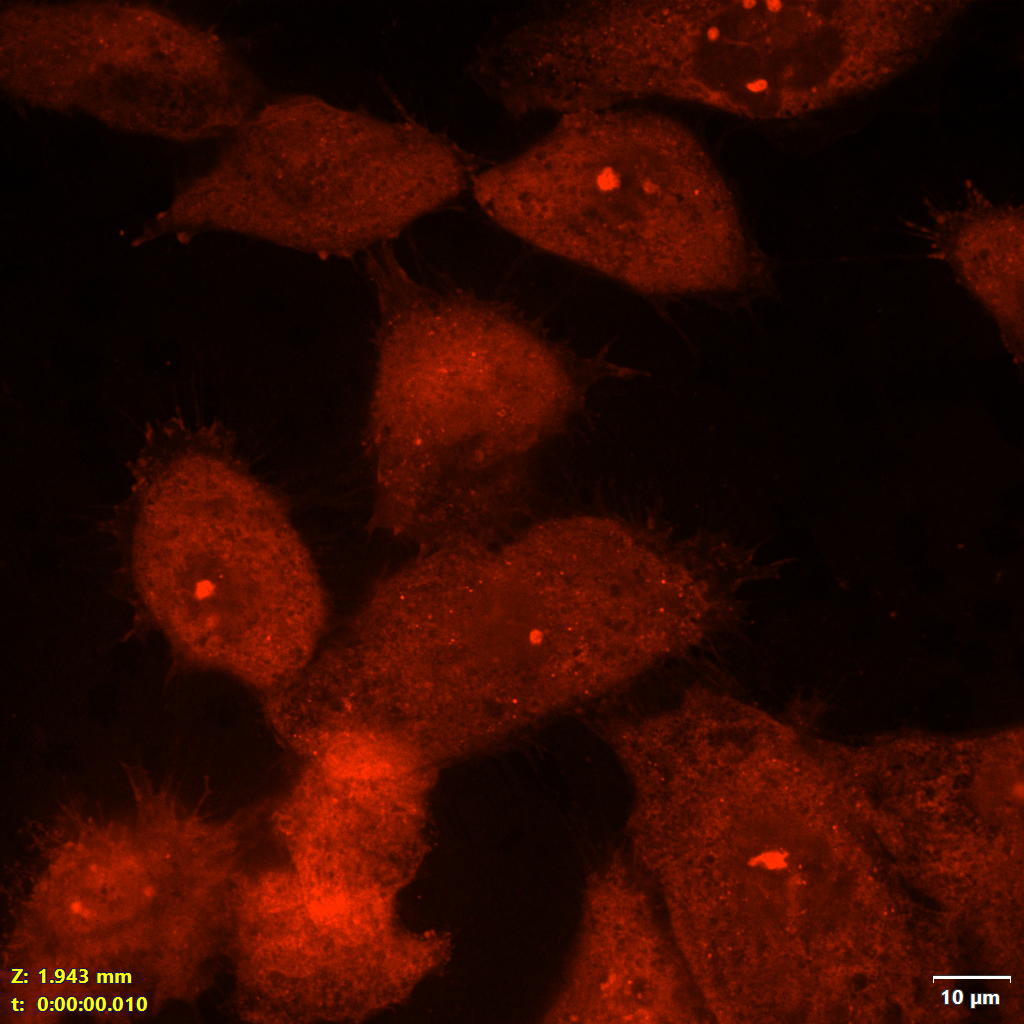

Supplement: Supplementary file 7 [file DataSheet5.zip › Rhod 2AM(1,2)/Rhod-2AM-2/Rhod-2-2═╝╞1⁄4/Control 5.tif]

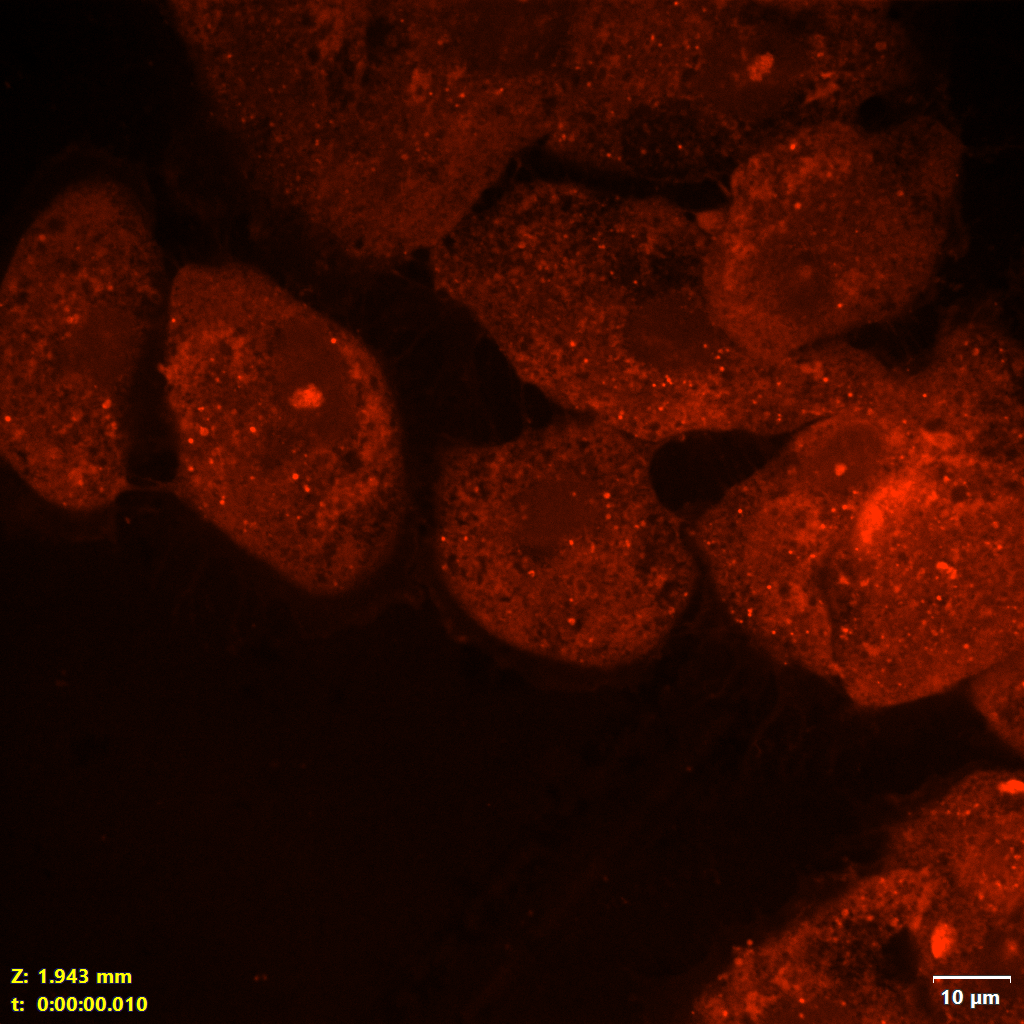

Supplement: Supplementary file 7 [file DataSheet5.zip › Rhod 2AM(1,2)/Rhod-2AM-2/Rhod-2-2═╝╞1⁄4/Iohexol 1.tif]

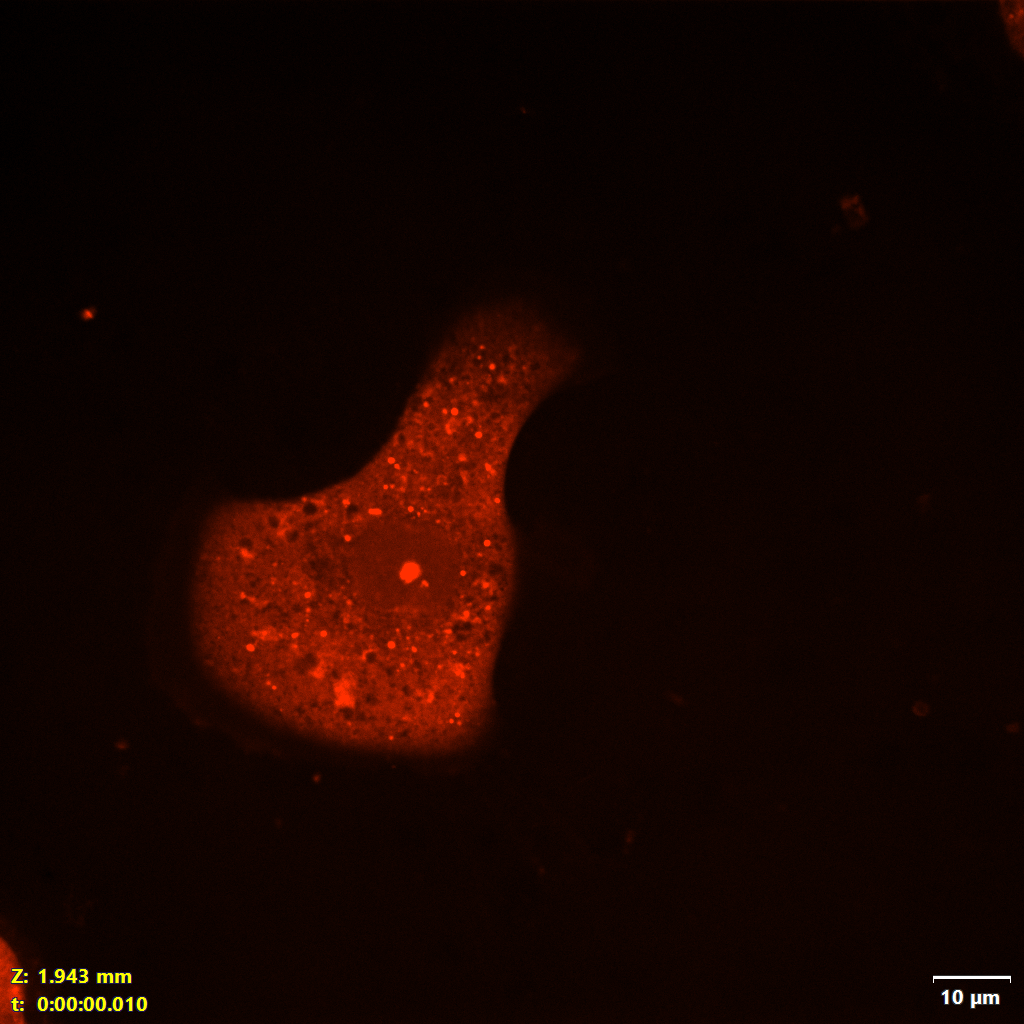

Supplement: Supplementary file 7 [file DataSheet5.zip › Rhod 2AM(1,2)/Rhod-2AM-2/Rhod-2-2═╝╞1⁄4/Iohexol 2.tif]

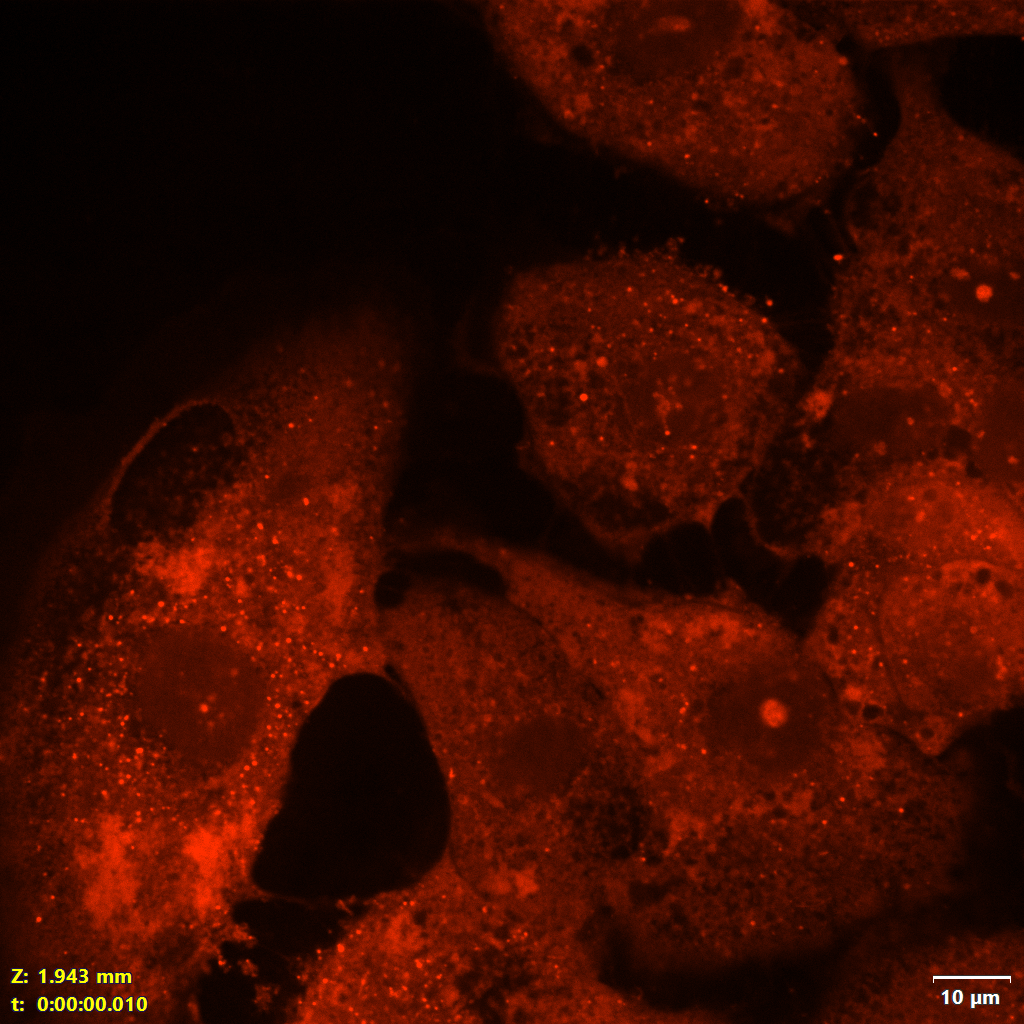

Supplement: Supplementary file 7 [file DataSheet5.zip › Rhod 2AM(1,2)/Rhod-2AM-2/Rhod-2-2═╝╞1⁄4/Iohexol 3.tif]

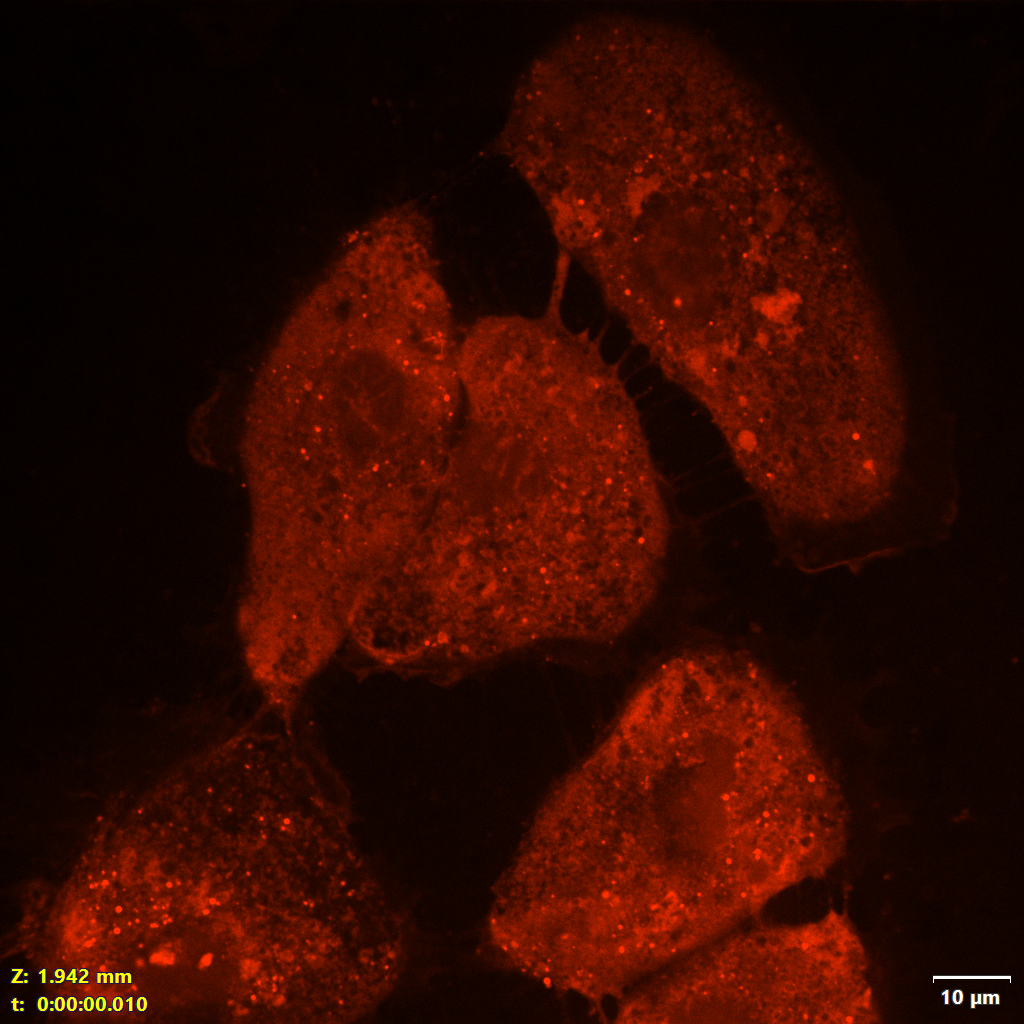

Supplement: Supplementary file 7 [file DataSheet5.zip › Rhod 2AM(1,2)/Rhod-2AM-2/Rhod-2-2═╝╞1⁄4/Iohexol 4.tif]

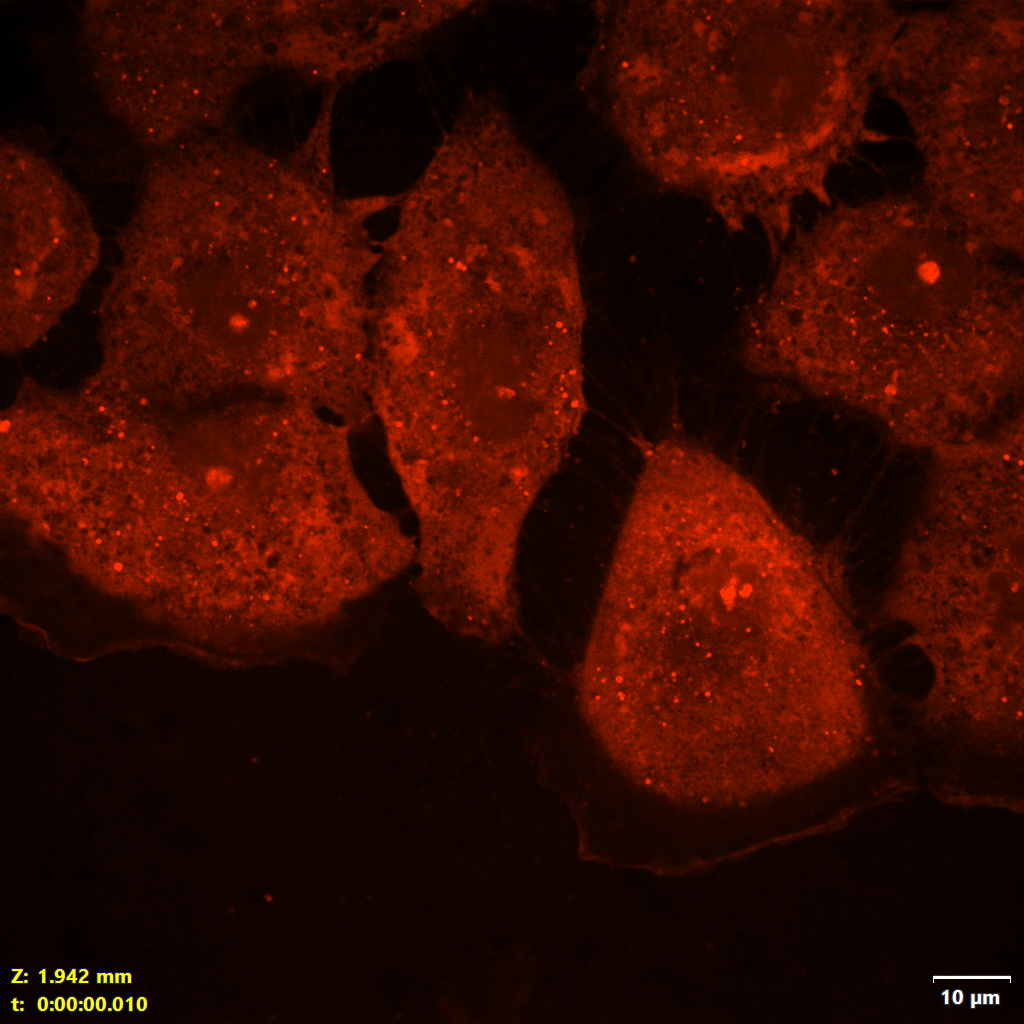

Supplement: Supplementary file 7 [file DataSheet5.zip › Rhod 2AM(1,2)/Rhod-2AM-2/Rhod-2-2═╝╞1⁄4/Iohexol 5.tif]

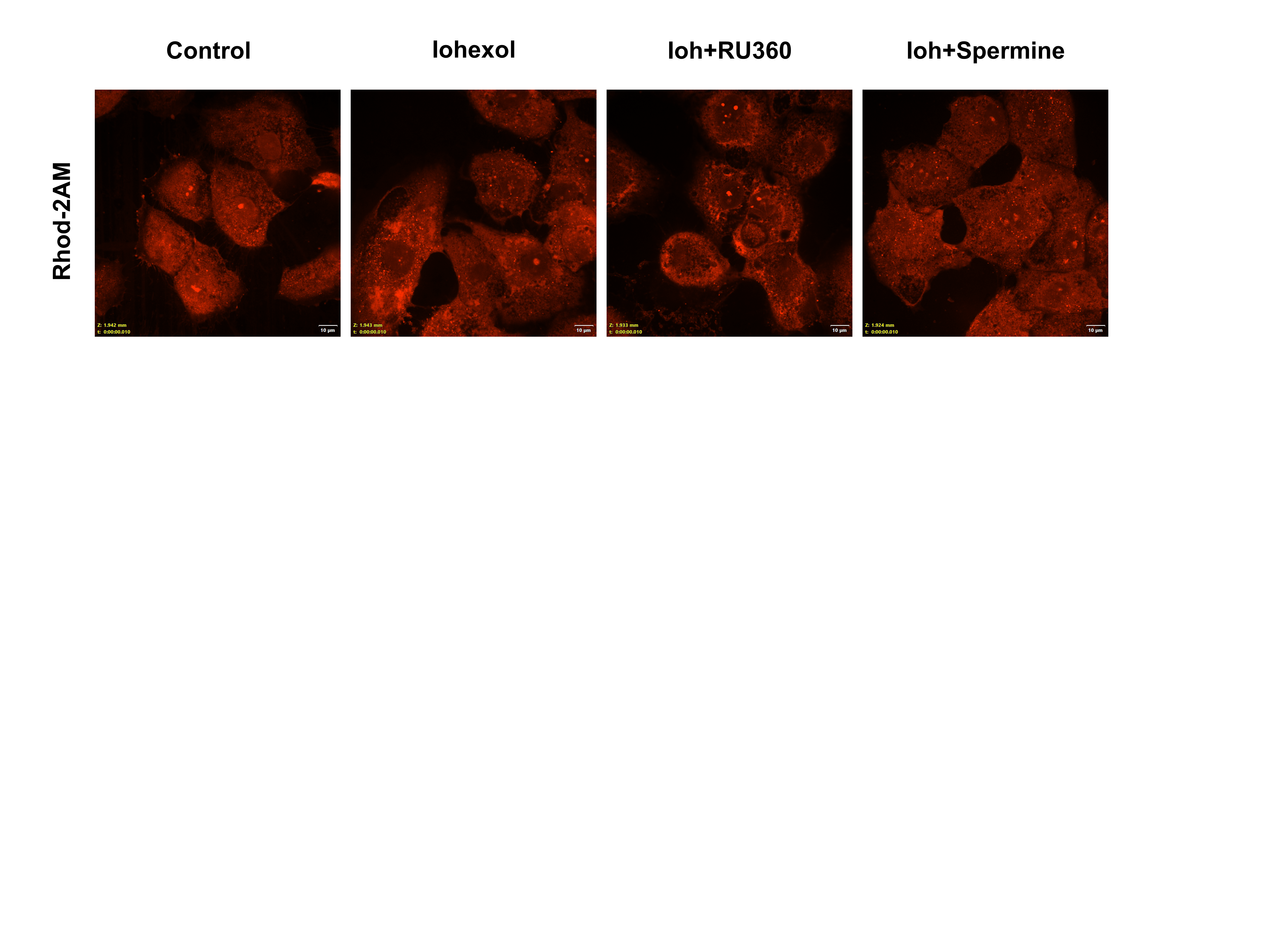

Supplement: Supplementary file 7 [file DataSheet5.zip › Rhod 2AM(1,2)/Rhod-2AM-2/Rhod-2-2═╝╞1⁄4/Rhod-2AM-2║╧═╝.tif]

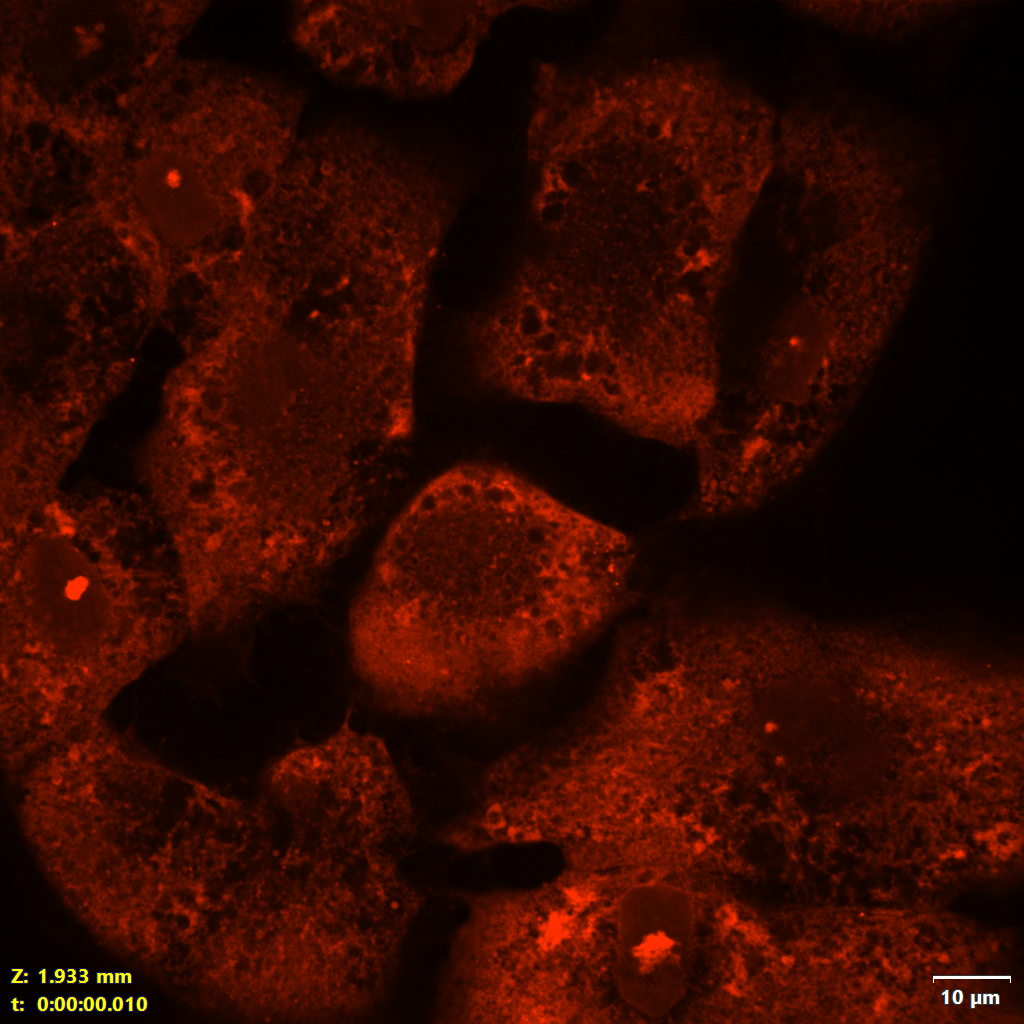

Supplement: Supplementary file 7 [file DataSheet5.zip › Rhod 2AM(1,2)/Rhod-2AM-2/Rhod-2-2═╝╞1⁄4/RU360+Iohexol 1.tif]

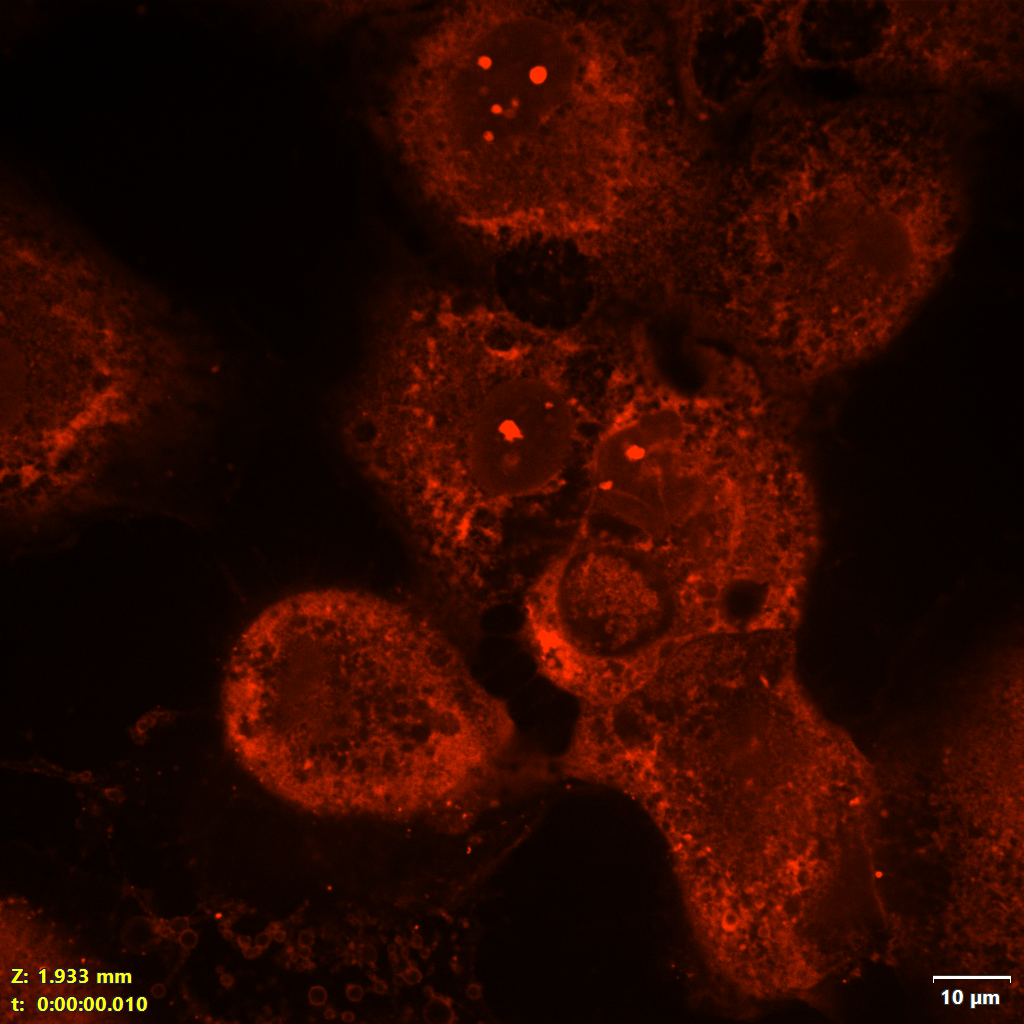

Supplement: Supplementary file 7 [file DataSheet5.zip › Rhod 2AM(1,2)/Rhod-2AM-2/Rhod-2-2═╝╞1⁄4/RU360+Iohexol 2.tif]

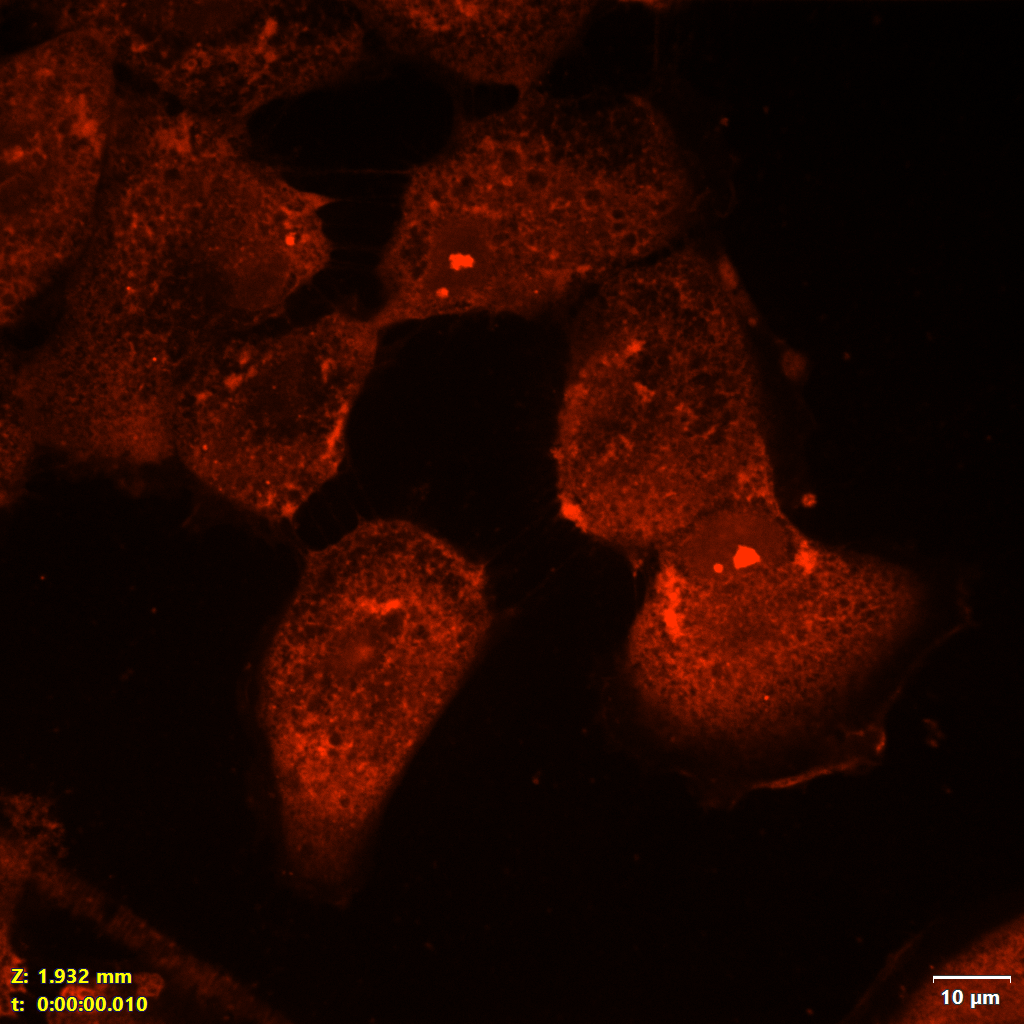

Supplement: Supplementary file 7 [file DataSheet5.zip › Rhod 2AM(1,2)/Rhod-2AM-2/Rhod-2-2═╝╞1⁄4/RU360+Iohexol 3.tif]

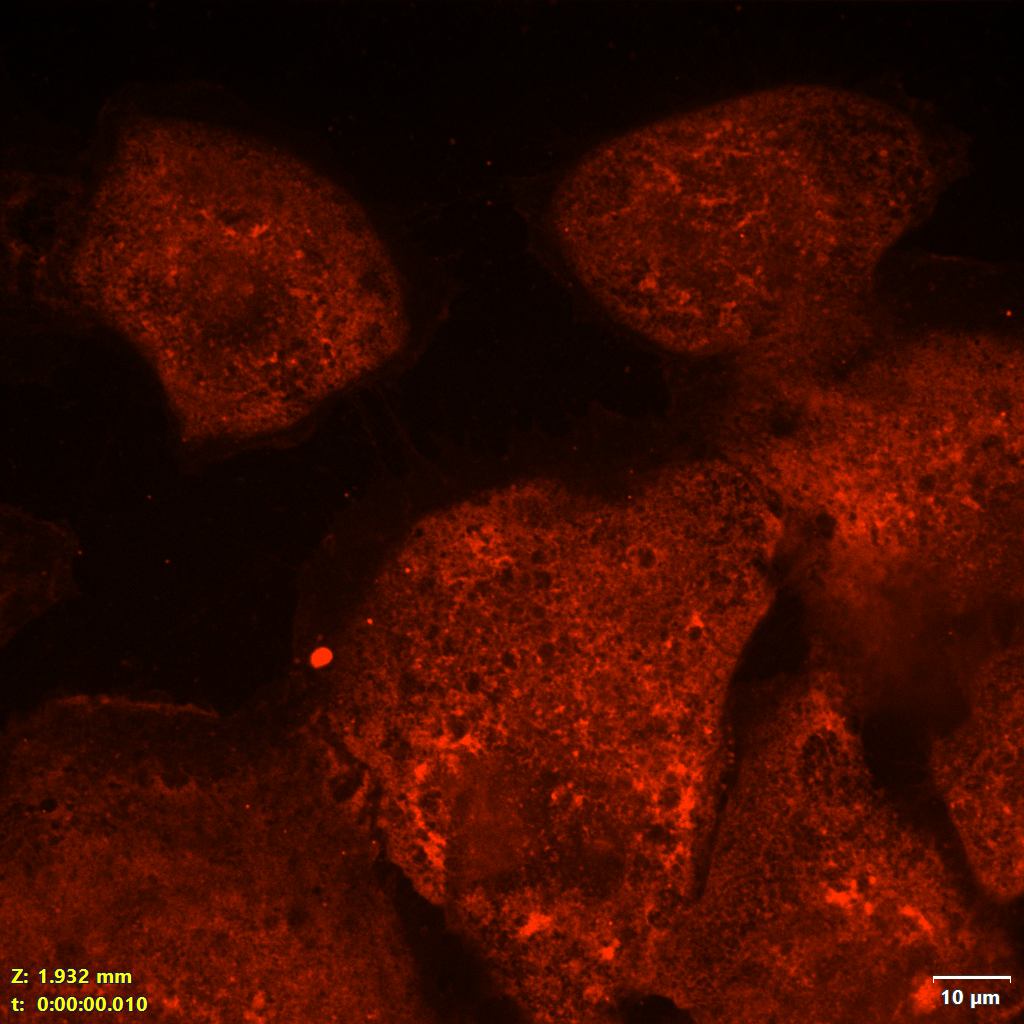

Supplement: Supplementary file 7 [file DataSheet5.zip › Rhod 2AM(1,2)/Rhod-2AM-2/Rhod-2-2═╝╞1⁄4/RU360+Iohexol 4.tif]

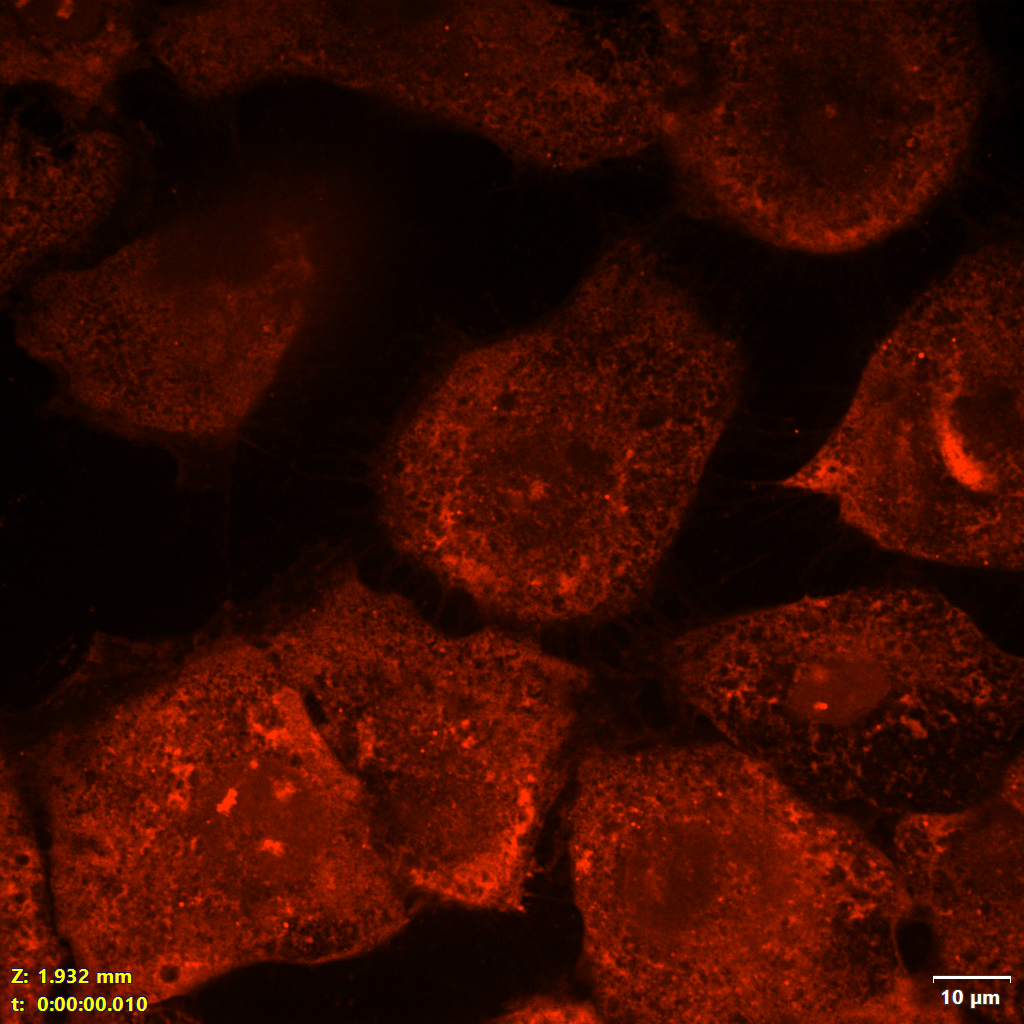

Supplement: Supplementary file 7 [file DataSheet5.zip › Rhod 2AM(1,2)/Rhod-2AM-2/Rhod-2-2═╝╞1⁄4/RU360+Iohexol 5.tif]

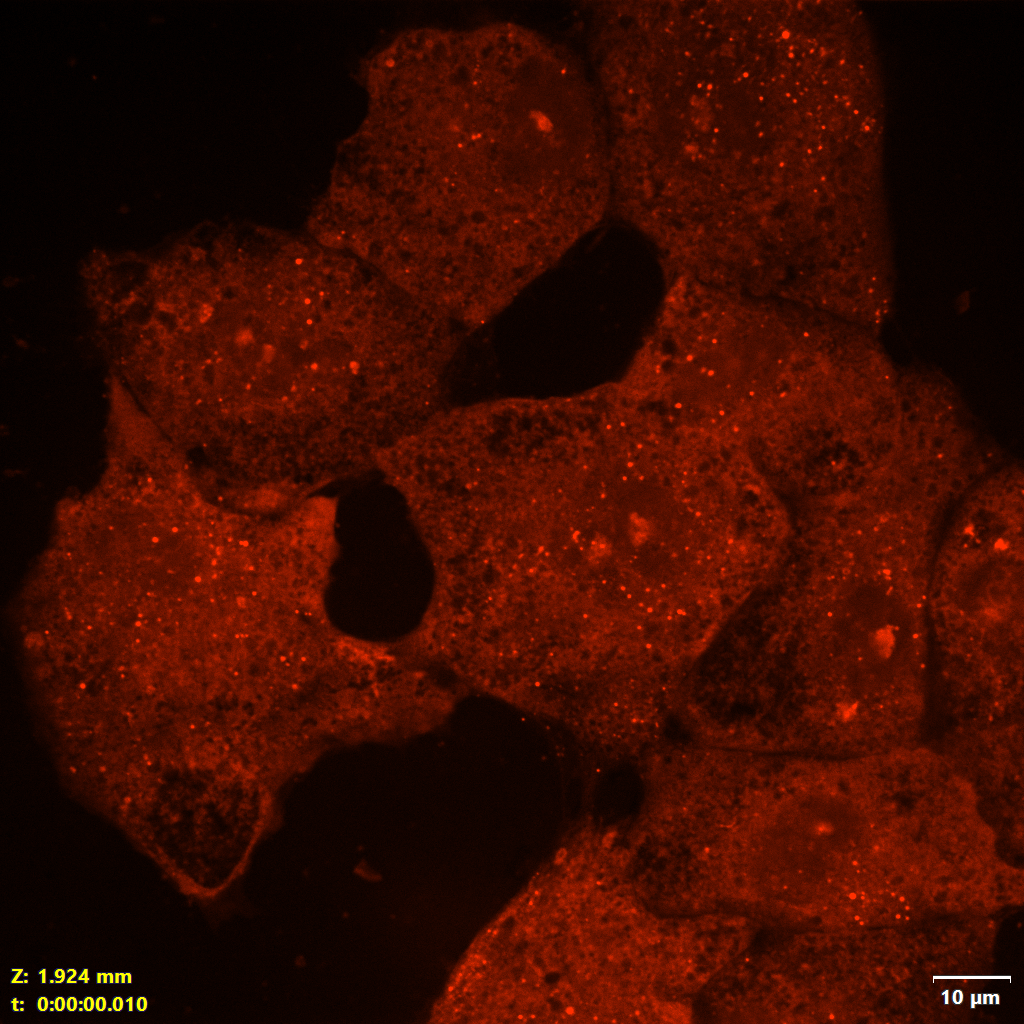

Supplement: Supplementary file 7 [file DataSheet5.zip › Rhod 2AM(1,2)/Rhod-2AM-2/Rhod-2-2═╝╞1⁄4/Spermine+Iohexol 1.tif]

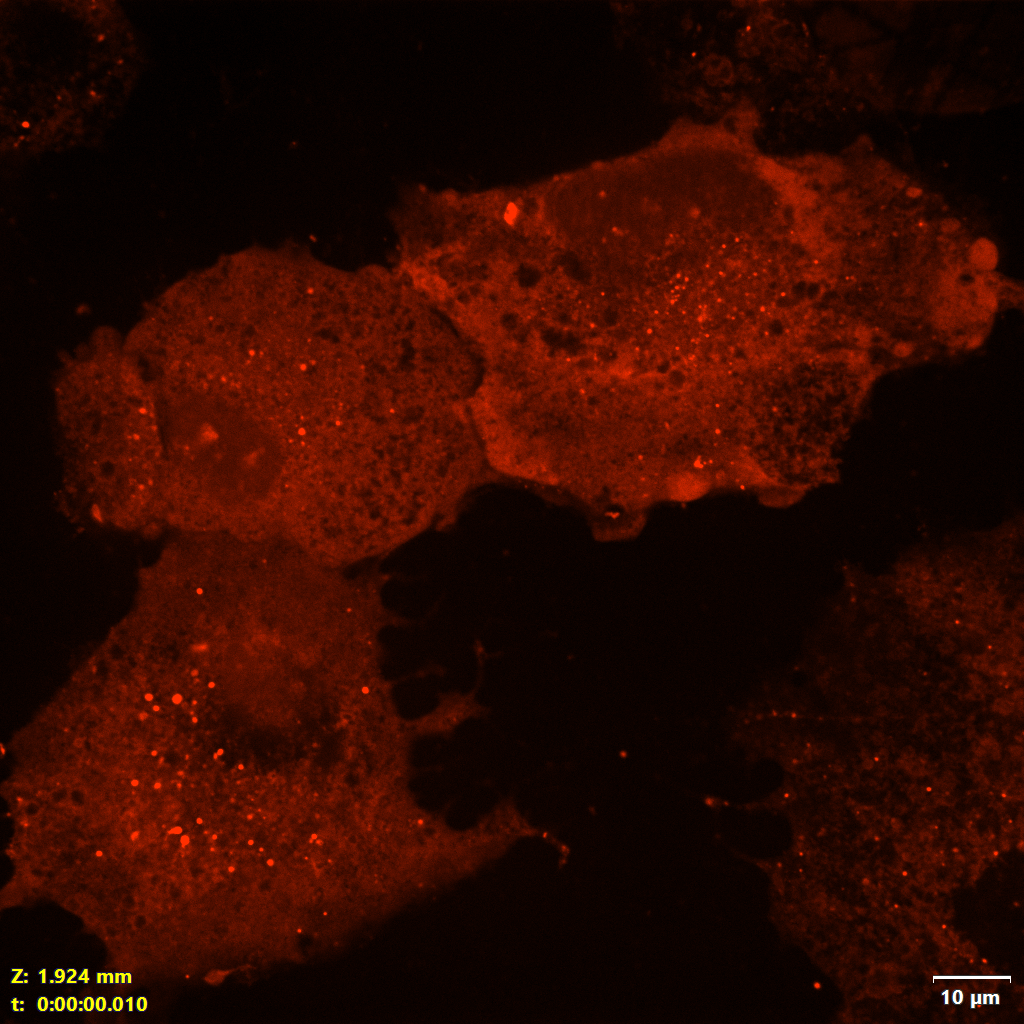

Supplement: Supplementary file 7 [file DataSheet5.zip › Rhod 2AM(1,2)/Rhod-2AM-2/Rhod-2-2═╝╞1⁄4/Spermine+Iohexol 2.tif]

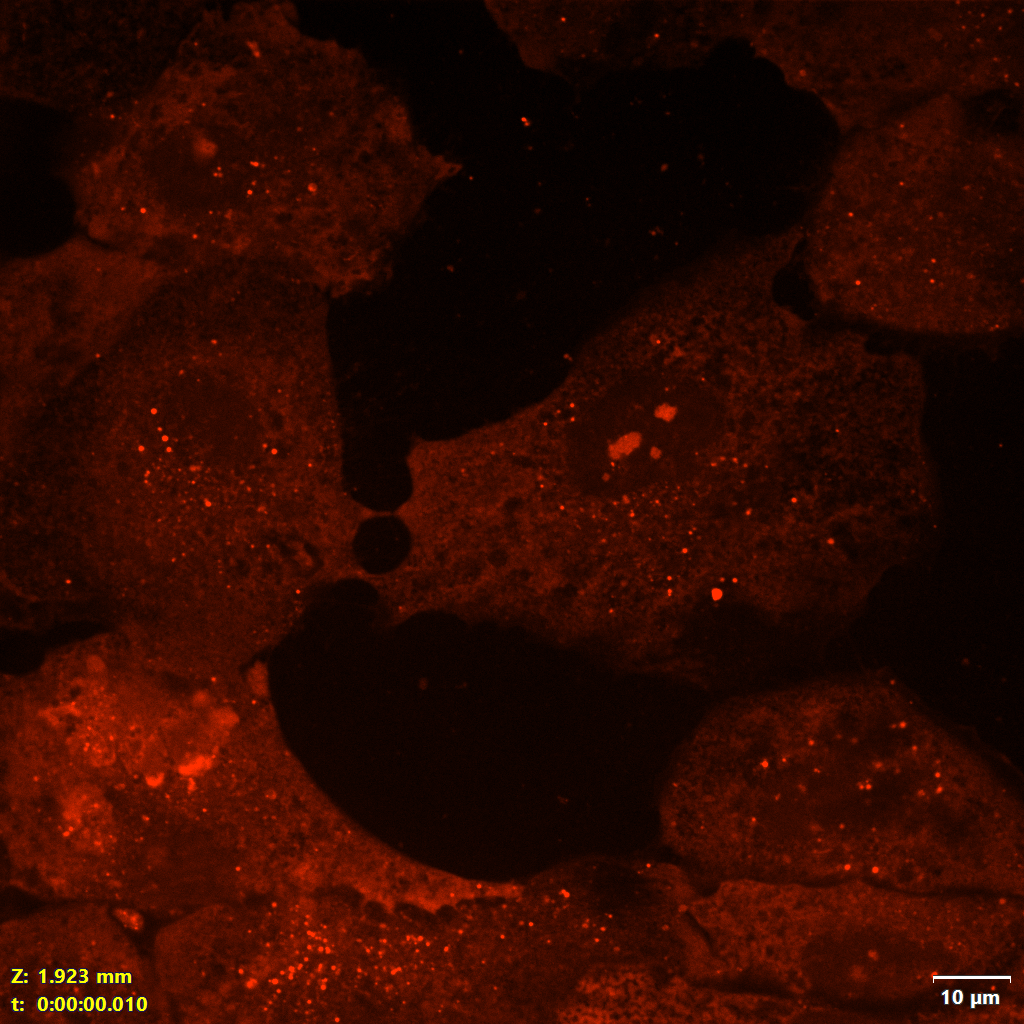

Supplement: Supplementary file 7 [file DataSheet5.zip › Rhod 2AM(1,2)/Rhod-2AM-2/Rhod-2-2═╝╞1⁄4/Spermine+Iohexol 3.tif]

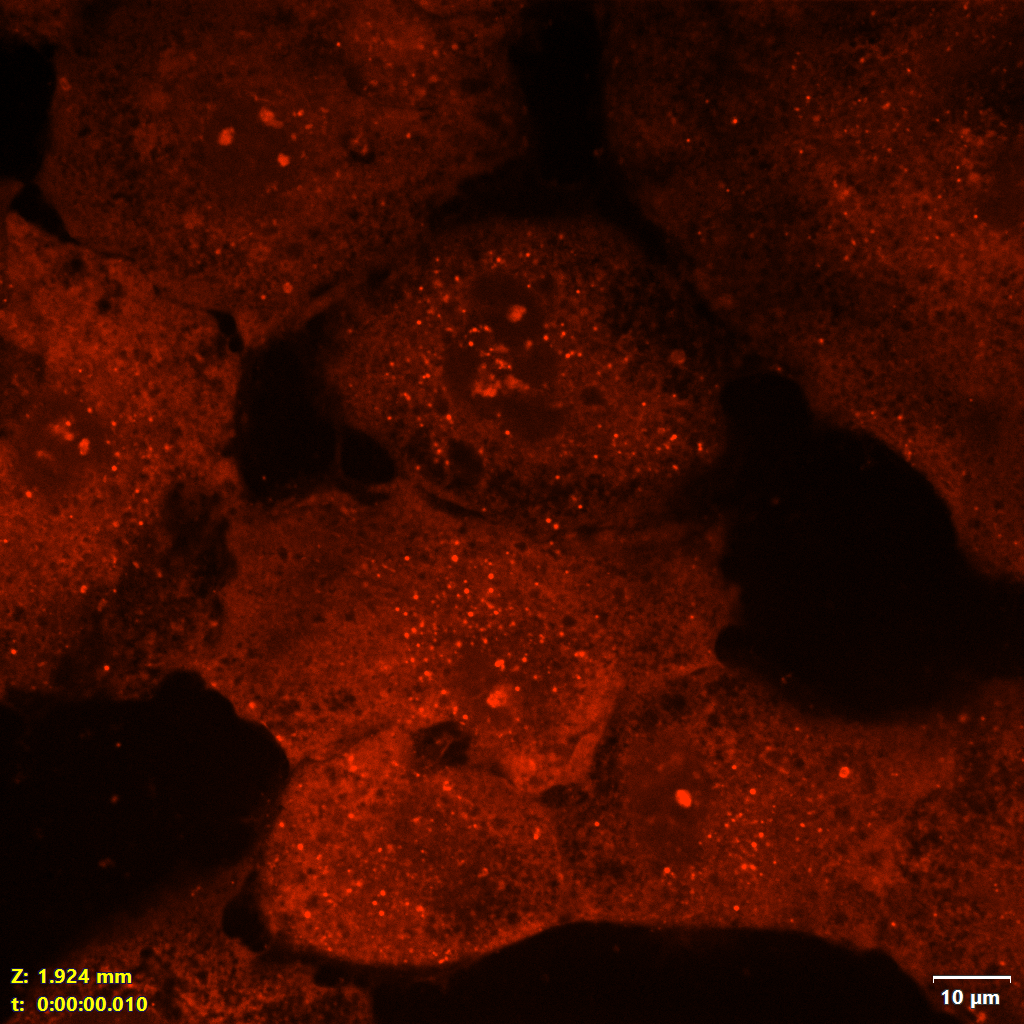

Supplement: Supplementary file 7 [file DataSheet5.zip › Rhod 2AM(1,2)/Rhod-2AM-2/Rhod-2-2═╝╞1⁄4/Spermine+Iohexol 4.tif]

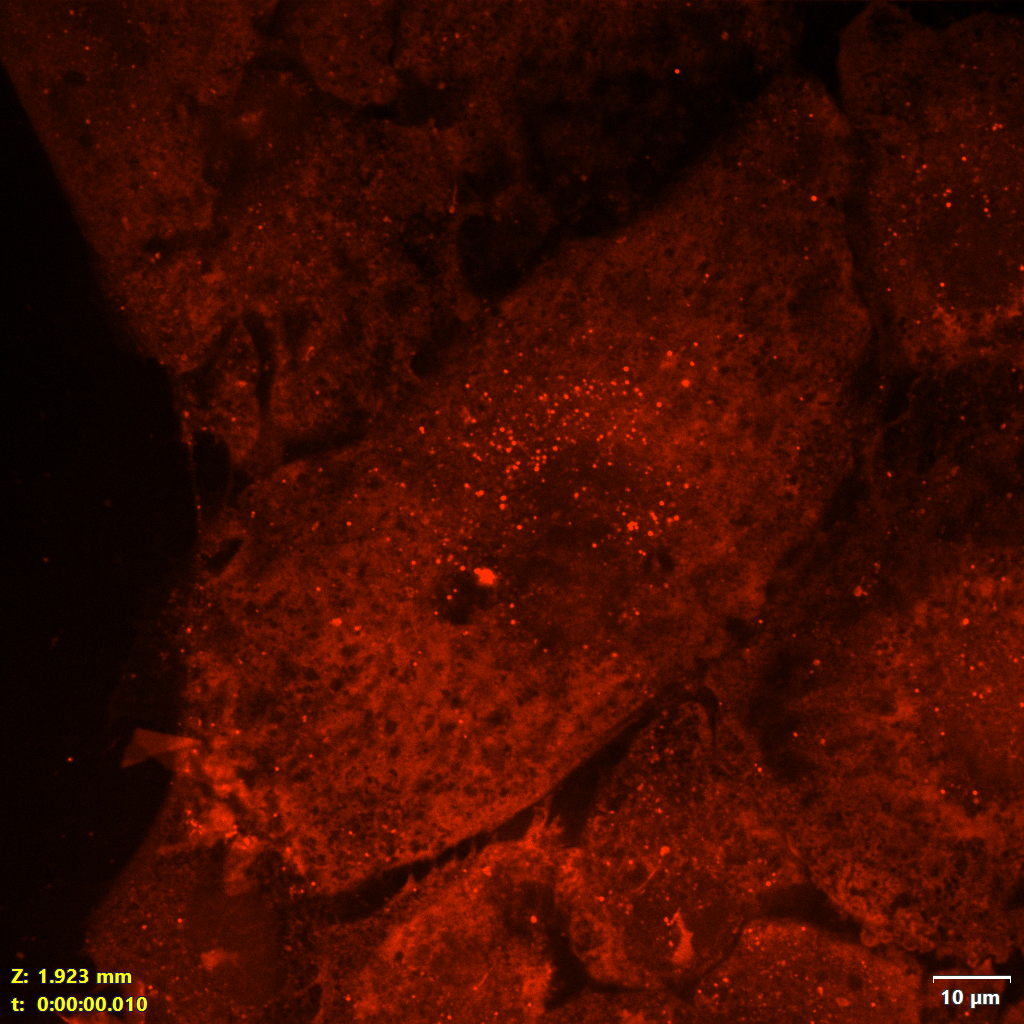

Supplement: Supplementary file 7 [file DataSheet5.zip › Rhod 2AM(1,2)/Rhod-2AM-2/Rhod-2-2═╝╞1⁄4/Spermine+Iohexol 5.tif]

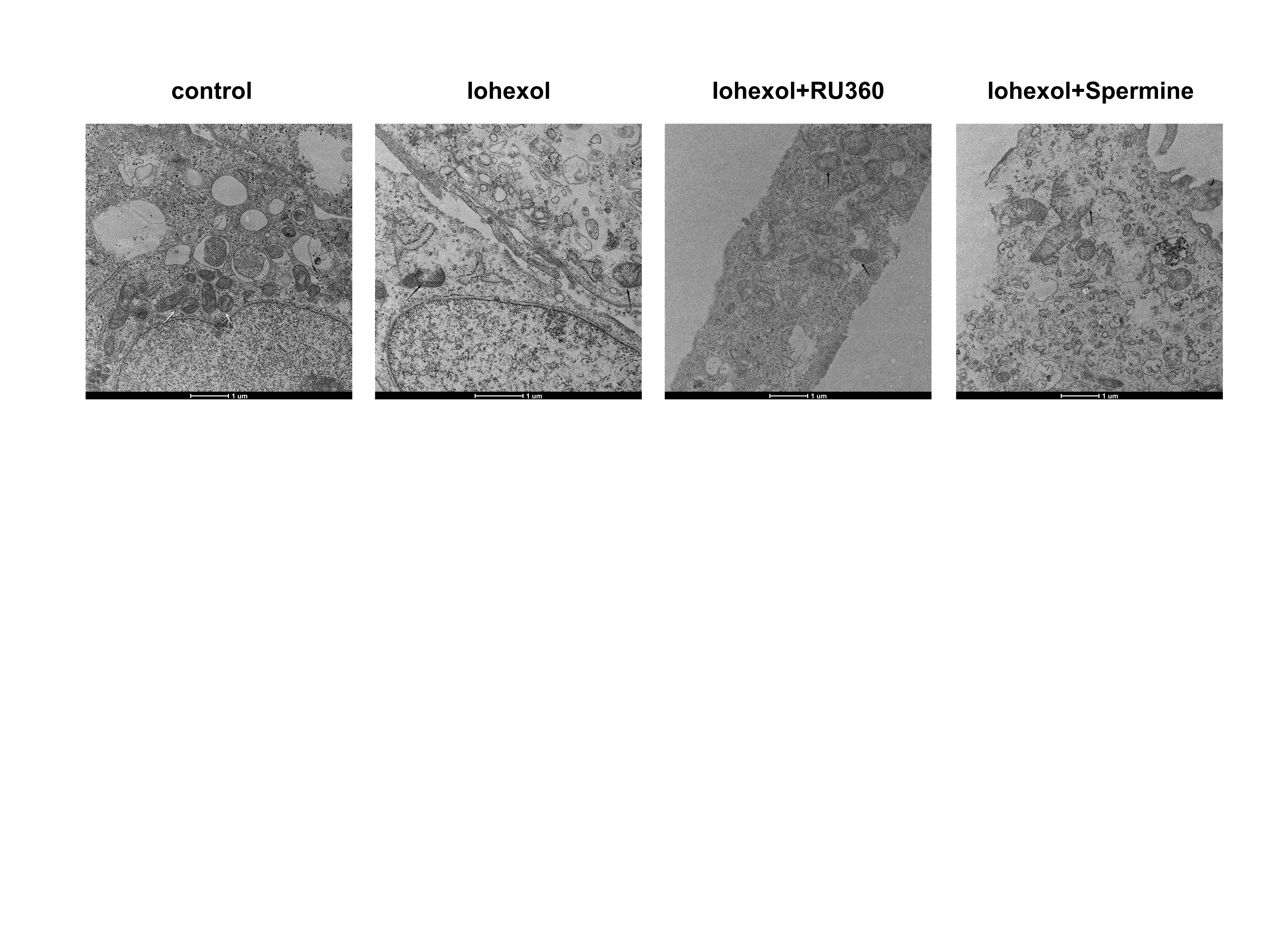

Supplement: Supplementary file 8 [file DataSheet7.zip › ╧▀┴ú╠σ ╡τ╛╡╜ß╣√/╡τ╛╡║╧▓ó.tif]
