# Supplementary material for: Mitochondrial calcium uniporter-mediated mitochondrial dynamics imbalance contributes to contrast medium-induced renal tubular cell injury
Source: Front Mol Biosci. 2026 Jun 29;13:1848361. doi: 10.3389/fmolb.2026.1848361 (PMC13357276; doi:10.3389/fmolb.2026.1848361)
Supplement: Supplementary file 1 [file DataSheet3.zip › Flow Cytometry Assay(1,2)/Flow Cytometry Assay-2/╧╕░√╡≥═÷-2/HK-2 ╡≥═÷ 2/▒¿╕μ - HK-2 ╡≥═÷ 2.pdf]

HK-2 凋亡 2 报告

标本名：HK-2 凋亡 2　　　　检验时间：2024/9/20 11:56

仪器：NovoCyte 451160320945　　　　软件：NovoExpress 1.2.4

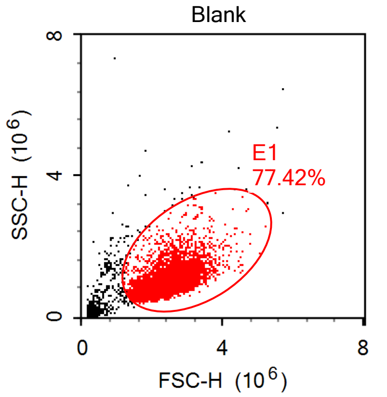

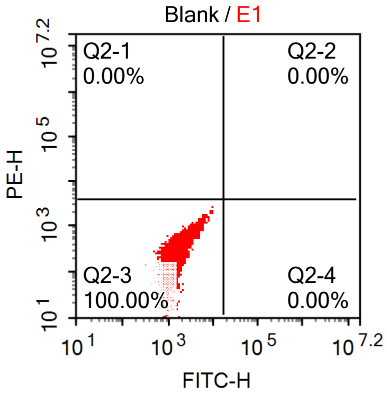

Gate	Count	% All	Mean X	M(
All	10,000	100.00%	1,927,422	84
E1	7,742	77.42%	2,411,340	1,(

Gate	Count	% E1	% All	Mean X	Mean Y	Median X	Median Y
E1	7,742	100.00%	77.42%	1,820	392	1,681	368
Q2-1	0	0.00%	0.00%	0	0	0	0
Q2-2	0	0.00%	0.00%	0	0	0	0
Q2-3	7,742	100.00%	77.42%	1,820	392	1,681	368
Q2-4	0	0.00%	0.00%	0	0	0	0

样本统计表格 - Blank

Gate	Count	% Parent	% All	X	Y	Mean X	Mean Y	Median X	Median Y
All	10,000								
E1	7,742	77.42%	77.42%	FSC-H	SSC-H	2,411,340	1,011,651	2,395,914	931,956
Q2-1	0	0.00%	0.00%	FITC-H	PE-H	0	0	0	0
Q2-2	0	0.00%	0.00%	FITC-H	PE-H	0	0	0	0
Q2-3	7,742	100.00%	77.42%	FITC-H	PE-H	1,820	392	1,681	368
Q2-4	0	0.00%	0.00%	FITC-H	PE-H	0	0	0	0

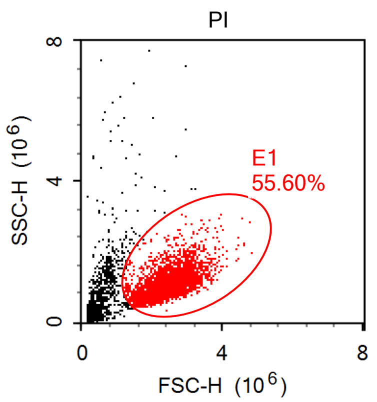

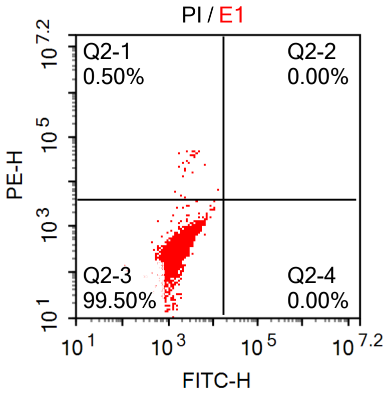

Gate	Count	% All	Mean X	M
All	10,000	100.00%	1,427,053	70
E1	5,560	55.60%	2,375,918	1,(

Gate	Count	% E1	% All	Mean X	Mean Y	Median X	Median Y
E1	5,560	100.00%	55.60%	1,816	546	1,676	381
Q2-1	28	0.50%	0.28%	3,579	27,195	2,928	27,175
Q2-2	0	0.00%	0.00%	0	0	0	0
Q2-3	5,532	99.50%	55.32%	1,807	411	1,673	380
Q2-4	0	0.00%	0.00%	0	0	0	0

样本统计表格 - PI

Gate	Count	% Parent	% All	X	Y	Mean X	Mean Y	Median X	Median Y
All	10,000								
E1	5,560	55.60%	55.60%	FSC-H	SSC-H	2,375,918	1,028,279	2,361,854	952,681
Q2-1	28	0.50%	0.28%	FITC-H	PE-H	3,579	27,195	2,928	27,175
Q2-2	0	0.00%	0.00%	FITC-H	PE-H	0	0	0	0
Q2-3	5,532	99.50%	55.32%	FITC-H	PE-H	1,807	411	1,673	380
Q2-4	0	0.00%	0.00%	FITC-H	PE-H	0	0	0	0

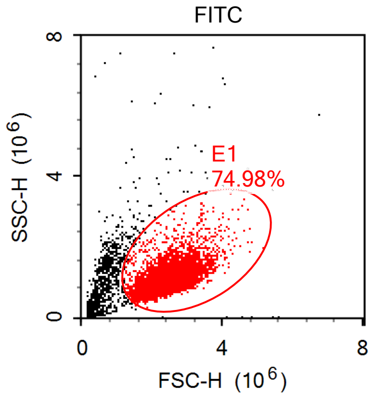

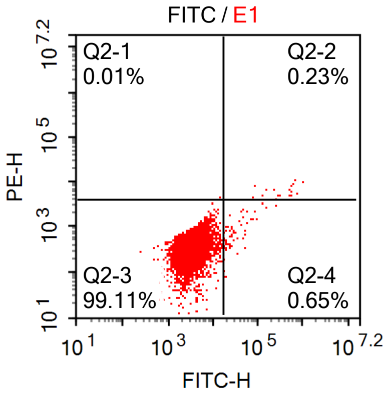

Gate	Count	% All	Mean X	M	Gate	Count	% E1	% All	Mean X	Mean Y	Median X	Median Y
All	10,000	100.00%	1,932,894	94	E1	7,498	100.00%	74.98%	4,633	420	3,052	376
E1	7,498	74.98%	2,440,645	1,(	Q2-1	1	0.01%	0.01%	13,638	4,436	13,638	4,436
					Q2-2	17	0.23%	0.17%	403,082	6,700	481,457	6,431
					Q2-3	7,431	99.11%	74.31%	3,476	401	3,040	375
					Q2-4	49	0.65%	0.49%	41,680	1,050	25,045	771

样本统计表格 - FITC

Gate	Count	% Parent	% All	X	Y	Mean X	Mean Y	Median X	Median Y
All	10,000								
E1	7,498	74.98%	74.98%	FSC-H	SSC-H	2,440,645	1,060,642	2,419,912	979,859
Q2-1	1	0.01%	0.01%	FITC-H	PE-H	13,638	4,436	13,638	4,436
Q2-2	17	0.23%	0.17%	FITC-H	PE-H	403,082	6,700	481,457	6,431
Q2-3	7,431	99.11%	74.31%	FITC-H	PE-H	3,476	401	3,040	375
Q2-4	49	0.65%	0.49%	FITC-H	PE-H	41,680	1,050	25,045	771

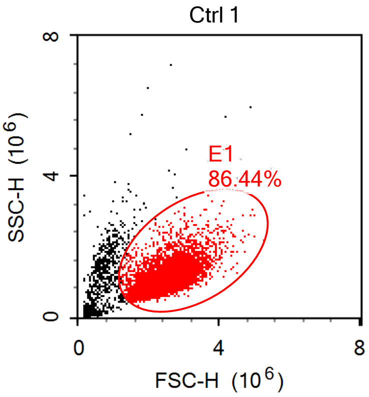

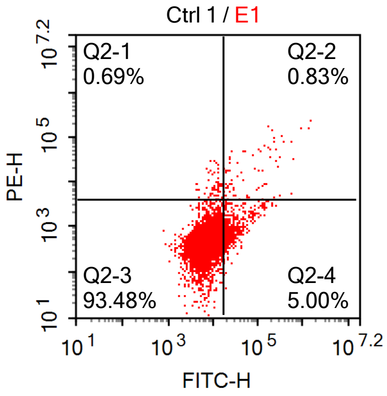

Gate	Count	% All	Mean X	M(
All	10,000	100.00%	2,179,616	1,(
E1	8,644	86.44%	2,446,304	1,(

Gate	Count	% E1	% All	Mean X	Mean Y	Median X	Median Y
E1	8,644	100.00%	86.44%	9,784	903	6,828	480
Q2-1	60	0.69%	0.60%	10,996	9,716	11,244	7,382
Q2-2	72	0.83%	0.72%	160,901	35,569	81,445	17,842
Q2-3	8,080	93.48%	80.80%	7,174	518	6,606	463
Q2-4	432	5.00%	4.32%	33,254	1,112	24,162	861

样本统计表格 - Ctrl 1

Gate	Count	% Parent	% All	X	Y	Mean X	Mean Y	Median X	Median Y
All	10,000								
E1	8,644	86.44%	86.44%	FSC-H	SSC-H	2,446,304	1,087,651	2,419,323	1,013,988
Q2-1	60	0.69%	0.60%	FITC-H	PE-H	10,996	9,716	11,244	7,382
Q2-2	72	0.83%	0.72%	FITC-H	PE-H	160,901	35,569	81,445	17,842
Q2-3	8,080	93.48%	80.80%	FITC-H	PE-H	7,174	518	6,606	463
Q2-4	432	5.00%	4.32%	FITC-H	PE-H	33,254	1,112	24,162	861

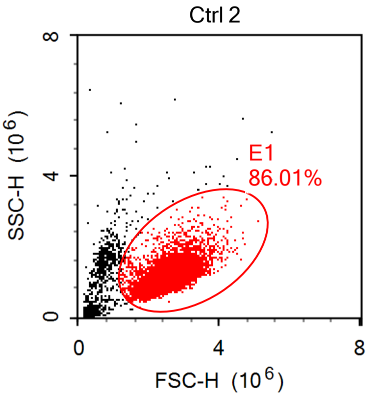

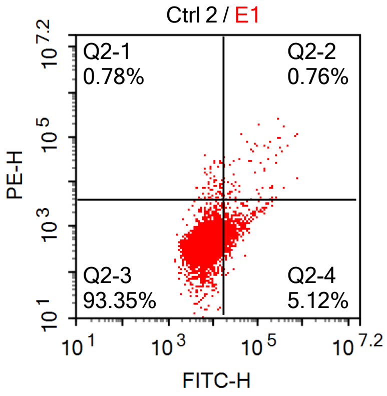

Gate	Count	% All	Mean X	M
All	10,000	100.00%	2,209,893	1,[illegible]
E1	8,601	86.01%	2,490,429	1,[illegible]

Gate	Count	% E1	% All	Mean X	Mean Y	Median X	Median Y
E1	8,601	100.00%	86.01%	9,509	916	6,797	480
Q2-1	67	0.78%	0.67%	10,569	11,329	10,476	7,750
Q2-2	65	0.76%	0.65%	143,269	38,085	84,906	15,572
Q2-3	8,029	93.35%	80.29%	7,174	518	6,572	465
Q2-4	440	5.12%	4.40%	32,199	1,097	23,366	892

样本统计表格 - Ctrl 2

Gate	Count	% Parent	% All	X	Y	Mean X	Mean Y	Median X	Median Y
All	10,000								
E1	8,601	86.01%	86.01%	FSC-H	SSC-H	2,490,429	1,117,334	2,476,168	1,048,401
Q2-1	67	0.78%	0.67%	FITC-H	PE-H	10,569	11,329	10,476	7,750
Q2-2	65	0.76%	0.65%	FITC-H	PE-H	143,269	38,085	84,906	15,572
Q2-3	8,029	93.35%	80.29%	FITC-H	PE-H	7,174	518	6,572	465
Q2-4	440	5.12%	4.40%	FITC-H	PE-H	32,199	1,097	23,366	892

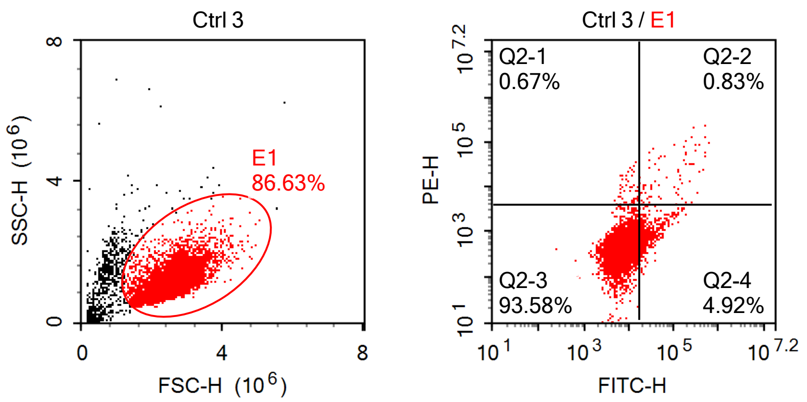

Gate	Count	% All	Mean X	M
All	10,000	100.00%	2,216,837	1,
E1	8,663	86.63%	2,485,499	1,

Gate	Count	% E1	% All	Mean X	Mean Y	Median X	Median Y
E1	8,663	100.00%	86.63%	9,668	920	6,805	480
Q2-1	58	0.67%	0.58%	10,600	10,391	10,603	7,674
Q2-2	72	0.83%	0.72%	144,253	37,405	114,893	19,882
Q2-3	8,107	93.58%	81.07%	7,223	519	6,569	466
Q2-4	426	4.92%	4.26%	33,332	1,096	24,285	918

样本统计表格 - Ctrl 3

Gate	Count	% Parent	% All	X	Y	Mean X	Mean Y	Median X	Median Y
All	10,000								
E1	8,663	86.63%	86.63%	FSC-H	SSC-H	2,485,499	1,123,735	2,474,073	1,048,480
Q2-1	58	0.67%	0.58%	FITC-H	PE-H	10,600	10,391	10,603	7,674
Q2-2	72	0.83%	0.72%	FITC-H	PE-H	144,253	37,405	114,893	19,882
Q2-3	8,107	93.58%	81.07%	FITC-H	PE-H	7,223	519	6,569	466
Q2-4	426	4.92%	4.26%	FITC-H	PE-H	33,332	1,096	24,285	918

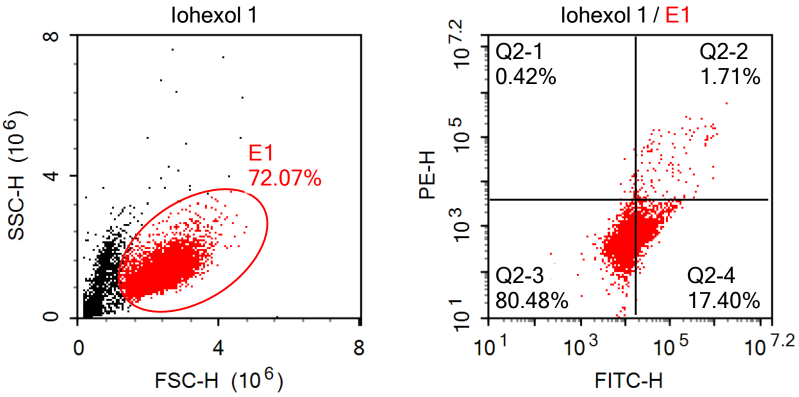

Gate	Count	% All	Mean X	M
All	10,000	100.00%	1,760,165	1,(
E1	7,207	72.07%	2,282,868	1,:

Gate	Count	% E1	% All	Mean X	Mean Y	Median X	Median Y
E1	7,207	100.00%	72.07%	16,976	1,647	11,482	567
Q2-1	30	0.42%	0.30%	14,257	11,241	15,091	6,269
Q2-2	123	1.71%	1.23%	199,850	55,650	76,338	21,717
Q2-3	5,800	80.48%	58.00%	10,569	582	10,373	518
Q2-4	1,254	17.40%	12.54%	28,737	1,045	22,717	878

样本统计表格 - Iohexol 1

Gate	Count	% Parent	% All	X	Y	Mean X	Mean Y	Median X	Median Y
All	10,000								
E1	7,207	72.07%	72.07%	FSC-H	SSC-H	2,282,868	1,259,619	2,259,492	1,198,704
Q2-1	30	0.42%	0.30%	FITC-H	PE-H	14,257	11,241	15,091	6,269
Q2-2	123	1.71%	1.23%	FITC-H	PE-H	199,850	55,650	76,338	21,717
Q2-3	5,800	80.48%	58.00%	FITC-H	PE-H	10,569	582	10,373	518
Q2-4	1,254	17.40%	12.54%	FITC-H	PE-H	28,737	1,045	22,717	878

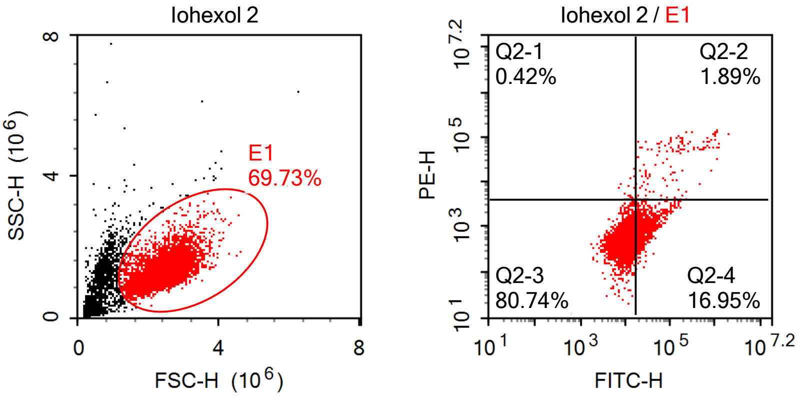

Gate	Count	% All	Mean X	M(
All	10,000	100.00%	1,764,468	1,(
E1	6,973	69.73%	2,348,588	1,:

Gate	Count	% E1	% All	Mean X	Mean Y	Median X	Median Y
E1	6,973	100.00%	69.73%	18,424	1,634	11,618	604
Q2-1	29	0.42%	0.29%	13,463	8,436	13,812	4,706
Q2-2	132	1.89%	1.32%	264,582	48,467	130,999	52,442
Q2-3	5,630	80.74%	56.30%	10,664	616	10,548	552
Q2-4	1,182	16.95%	11.82%	28,016	1,089	22,438	934

样本统计表格 - Iohexol 2

Gate	Count	% Parent	% All	X	Y	Mean X	Mean Y	Median X	Median Y
All	10,000								
E1	6,973	69.73%	69.73%	FSC-H	SSC-H	2,348,588	1,295,507	2,335,949	1,229,420
Q2-1	29	0.42%	0.29%	FITC-H	PE-H	13,463	8,436	13,812	4,706
Q2-2	132	1.89%	1.32%	FITC-H	PE-H	264,582	48,467	130,999	52,442
Q2-3	5,630	80.74%	56.30%	FITC-H	PE-H	10,664	616	10,548	552
Q2-4	1,182	16.95%	11.82%	FITC-H	PE-H	28,016	1,089	22,438	934

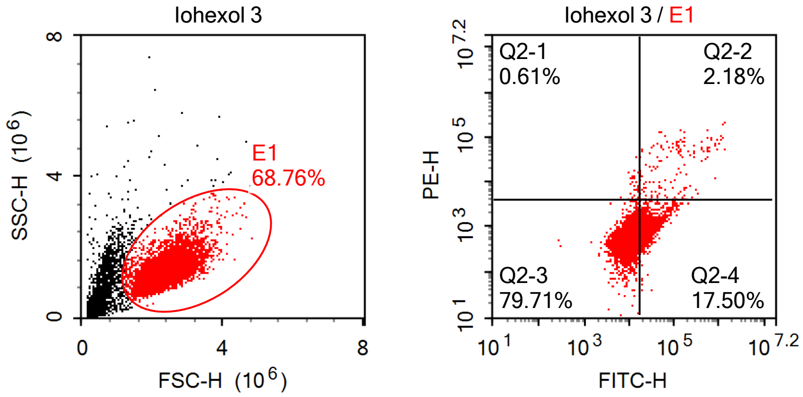

Gate	Count	% All	Mean X	M(	Gate	Count	% E1	% All	Mean X	Mean Y	Median X	Median Y
All	10,000	100.00%	1,750,602	1,(	E1	6,876	100.00%	68.76%	18,036	1,645	11,573	619
E1	6,876	68.76%	2,355,765	1,:	Q2-1	42	0.61%	0.42%	13,567	10,787	13,829	8,841
					Q2-2	150	2.18%	1.50%	211,780	40,514	111,271	31,894
					Q2-3	5,481	79.71%	54.81%	10,537	623	10,374	561
					Q2-4	1,203	17.50%	12.03%	28,205	1,140	22,573	988

样本统计表格 - Iohexol 3

Gate	Count	% Parent	% All	X	Y	Mean X	Mean Y	Median X	Median Y
All	10,000								
E1	6,876	68.76%	68.76%	FSC-H	SSC-H	2,355,765	1,308,482	2,337,684	1,245,253
Q2-1	42	0.61%	0.42%	FITC-H	PE-H	13,567	10,787	13,829	8,841
Q2-2	150	2.18%	1.50%	FITC-H	PE-H	211,780	40,514	111,271	31,894
Q2-3	5,481	79.71%	54.81%	FITC-H	PE-H	10,537	623	10,374	561
Q2-4	1,203	17.50%	12.03%	FITC-H	PE-H	28,205	1,140	22,573	988

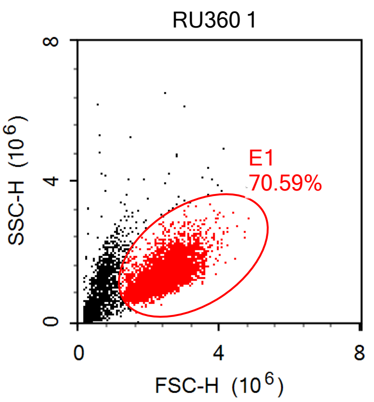

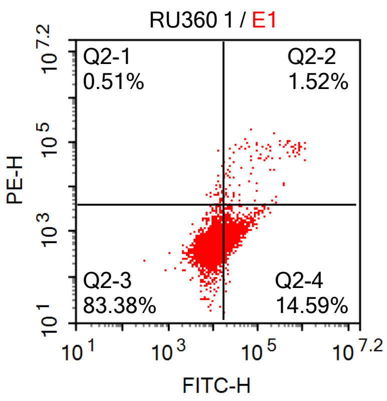

Gate	Count	% All	Mean X	M
All	10,000	100.00%	1,793,846	1,
E1	7,059	70.59%	2,360,863	1,

Gate	Count	% E1	% All	Mean X	Mean Y	Median X	Median Y
E1	7,059	100.00%	70.59%	16,141	1,438	10,658	584
Q2-1	36	0.51%	0.36%	13,078	11,111	13,186	6,969
Q2-2	107	1.52%	1.07%	230,109	47,573	97,638	44,999
Q2-3	5,886	83.38%	58.86%	10,071	602	9,761	543
Q2-4	1,030	14.59%	10.30%	28,708	1,081	22,713	922

样本统计表格 - RU360 1

Gate	Count	% Parent	% All	X	Y	Mean X	Mean Y	Median X	Median Y
All	10,000								
E1	7,059	70.59%	70.59%	FSC-H	SSC-H	2,360,863	1,291,330	2,344,467	1,221,084
Q2-1	36	0.51%	0.36%	FITC-H	PE-H	13,078	11,111	13,186	6,969
Q2-2	107	1.52%	1.07%	FITC-H	PE-H	230,109	47,573	97,638	44,999
Q2-3	5,886	83.38%	58.86%	FITC-H	PE-H	10,071	602	9,761	543
Q2-4	1,030	14.59%	10.30%	FITC-H	PE-H	28,708	1,081	22,713	922

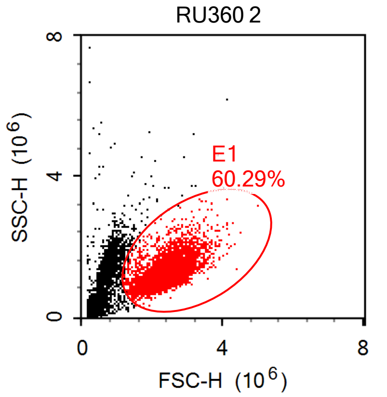

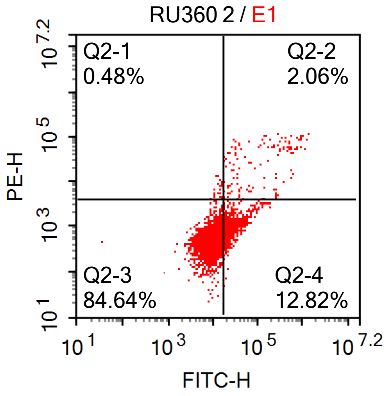

Gate	Count	% All	Mean X	M
All	10,000	100.00%	1,564,672	99
E1	6,029	60.29%	2,334,273	1,

Gate	Count	% E1	% All	Mean X	Mean Y	Median X	Median Y
E1	6,029	100.00%	60.29%	17,397	1,555	10,438	580
Q2-1	29	0.48%	0.29%	13,827	8,733	14,336	6,147
Q2-2	124	2.06%	1.24%	252,833	42,632	129,390	40,152
Q2-3	5,103	84.64%	51.03%	9,952	586	9,635	538
Q2-4	773	12.82%	7.73%	28,911	1,092	22,887	935

样本统计表格 - RU360 2

Gate	Count	% Parent	% All	X	Y	Mean X	Mean Y	Median X	Median Y
All	10,000								
E1	6,029	60.29%	60.29%	FSC-H	SSC-H	2,334,273	1,258,825	2,316,266	1,183,512
Q2-1	29	0.48%	0.29%	FITC-H	PE-H	13,827	8,733	14,336	6,147
Q2-2	124	2.06%	1.24%	FITC-H	PE-H	252,833	42,632	129,390	40,152
Q2-3	5,103	84.64%	51.03%	FITC-H	PE-H	9,952	586	9,635	538
Q2-4	773	12.82%	7.73%	FITC-H	PE-H	28,911	1,092	22,887	935

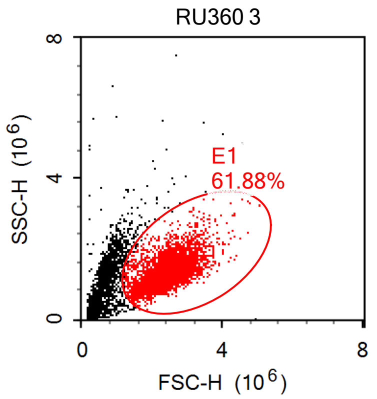

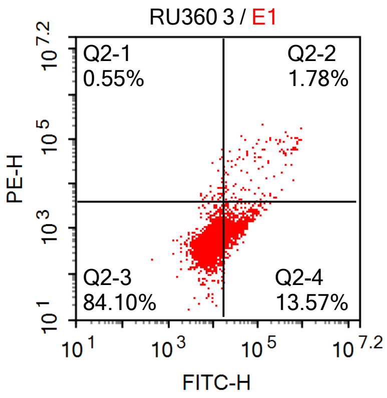

Gate	Count	% All	Mean X	M[illegible]
All	10,000	100.00%	1,612,173	1,[illegible]
E1	6,188	61.88%	2,352,345	1,[illegible]

Gate	Count	% E1	% All	Mean X	Mean Y	Median X	Median Y
E1	6,188	100.00%	61.88%	16,340	1,515	10,560	586
Q2-1	34	0.55%	0.34%	12,477	12,427	13,392	6,464
Q2-2	110	1.78%	1.10%	220,393	44,431	122,671	35,360
Q2-3	5,204	84.10%	52.04%	10,026	597	9,717	540
Q2-4	840	13.57%	8.40%	28,890	1,138	22,585	952

样本统计表格 - RU360 3

Gate	Count	% Parent	% All	X	Y	Mean X	Mean Y	Median X	Median Y
All	10,000								
E1	6,188	61.88%	61.88%	FSC-H	SSC-H	2,352,345	1,269,206	2,330,642	1,195,748
Q2-1	34	0.55%	0.34%	FITC-H	PE-H	12,477	12,427	13,392	6,464
Q2-2	110	1.78%	1.10%	FITC-H	PE-H	220,393	44,431	122,671	35,360
Q2-3	5,204	84.10%	52.04%	FITC-H	PE-H	10,026	597	9,717	540
Q2-4	840	13.57%	8.40%	FITC-H	PE-H	28,890	1,138	22,585	952

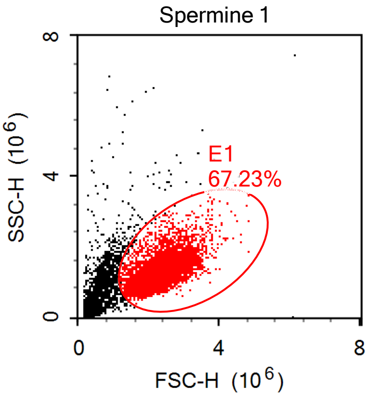

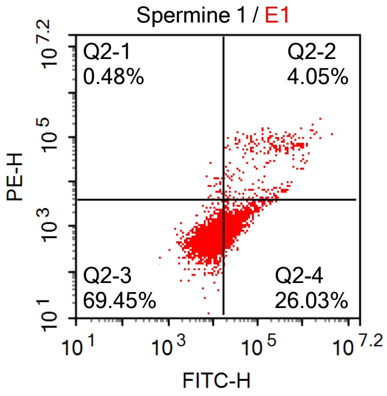

Gate	Count	% All	Mean X	M(
All	10,000	100.00%	1,694,675	1,(
E1	6,723	67.23%	2,313,691	1,:

Gate	Count	% E1	% All	Mean X	Mean Y	Median X	Median Y
E1	6,723	100.00%	67.23%	28,004	2,867	13,076	753
Q2-1	32	0.48%	0.32%	12,221	12,012	12,309	8,164
Q2-2	272	4.05%	2.72%	327,981	49,878	167,344	49,188
Q2-3	4,669	69.45%	46.69%	10,428	689	10,345	640
Q2-4	1,750	26.03%	17.50%	28,562	1,205	23,634	1,081

样本统计表格 - Spermine 1

Gate	Count	% Parent	% All	X	Y	Mean X	Mean Y	Median X	Median Y
All	10,000								
E1	6,723	67.23%	67.23%	FSC-H	SSC-H	2,313,691	1,288,960	2,288,011	1,220,331
Q2-1	32	0.48%	0.32%	FITC-H	PE-H	12,221	12,012	12,309	8,164
Q2-2	272	4.05%	2.72%	FITC-H	PE-H	327,981	49,878	167,344	49,188
Q2-3	4,669	69.45%	46.69%	FITC-H	PE-H	10,428	689	10,345	640
Q2-4	1,750	26.03%	17.50%	FITC-H	PE-H	28,562	1,205	23,634	1,081

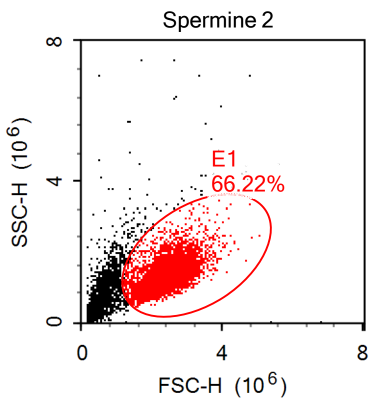

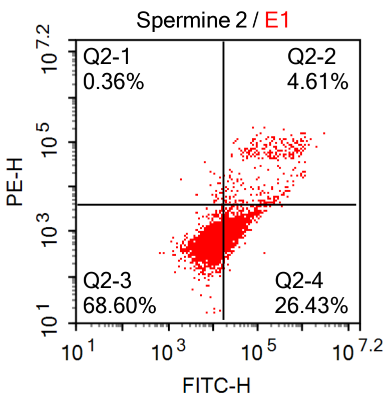

Gate	Count	% All	Mean X	M[illegible]
All	10,000	100.00%	1,695,438	1,[illegible]
E1	6,622	66.22%	2,336,620	1,[illegible]

Gate	Count	% E1	% All	Mean X	Mean Y	Median X	Median Y
E1	6,622	100.00%	66.22%	28,822	3,108	13,163	760
Q2-1	24	0.36%	0.24%	11,361	10,857	11,574	7,752
Q2-2	305	4.61%	3.05%	303,475	49,355	175,774	48,417
Q2-3	4,543	68.60%	45.43%	10,488	693	10,469	644
Q2-4	1,750	26.43%	17.50%	28,787	1,209	23,638	1,091

样本统计表格 - Spermine 2

Gate	Count	% Parent	% All	X	Y	Mean X	Mean Y	Median X	Median Y
All	10,000								
E1	6,622	66.22%	66.22%	FSC-H	SSC-H	2,336,620	1,295,707	2,314,265	1,229,451
Q2-1	24	0.36%	0.24%	FITC-H	PE-H	11,361	10,857	11,574	7,752
Q2-2	305	4.61%	3.05%	FITC-H	PE-H	303,475	49,355	175,774	48,417
Q2-3	4,543	68.60%	45.43%	FITC-H	PE-H	10,488	693	10,469	644
Q2-4	1,750	26.43%	17.50%	FITC-H	PE-H	28,787	1,209	23,638	1,091

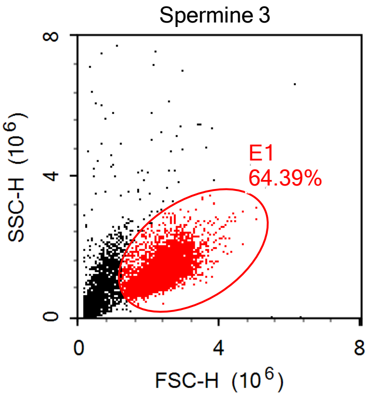

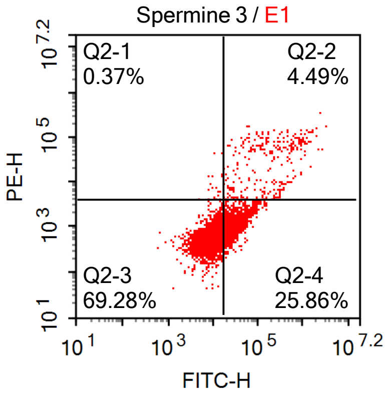

Gate	Count	% All	Mean X	M[illegible]
All	10,000	100.00%	1,631,148	1,[illegible]
E1	6,439	64.39%	2,302,248	1,[illegible]

Gate	Count	% E1	% All	Mean X	Mean Y	Median X	Median Y
E1	6,439	100.00%	64.39%	30,585	3,095	12,997	752
Q2-1	24	0.37%	0.24%	11,651	10,876	11,308	6,348
Q2-2	289	4.49%	2.89%	350,617	50,397	193,247	48,766
Q2-3	4,461	69.28%	44.61%	10,427	688	10,378	640
Q2-4	1,665	25.86%	16.65%	29,318	1,220	24,174	1,084

样本统计表格 - Spermine 3

Gate	Count	% Parent	% All	X	Y	Mean X	Mean Y	Median X	Median Y
All	10,000								
E1	6,439	64.39%	64.39%	FSC-H	SSC-H	2,302,248	1,279,421	2,279,577	1,220,277
Q2-1	24	0.37%	0.24%	FITC-H	PE-H	11,651	10,876	11,308	6,348
Q2-2	289	4.49%	2.89%	FITC-H	PE-H	350,617	50,397	193,247	48,766
Q2-3	4,461	69.28%	44.61%	FITC-H	PE-H	10,427	688	10,378	640
Q2-4	1,665	25.86%	16.65%	FITC-H	PE-H	29,318	1,220	24,174	1,084
