# Supplementary material for: Mitochondrial calcium uniporter-mediated mitochondrial dynamics imbalance contributes to contrast medium-induced renal tubular cell injury
Source: Front Mol Biosci. 2026 Jun 29;13:1848361. doi: 10.3389/fmolb.2026.1848361 (PMC13357276; doi:10.3389/fmolb.2026.1848361)
Supplement: Supplementary file 1 [file DataSheet3.zip › Flow Cytometry Assay(1,2)/Flow Cytometry Assay-2/╧╕░√╡≥═÷-2/HK-2 ╡≥═÷ 3/▒¿╕μ - HK-2 ╡≥═÷ 3.pdf]

HK-2 凋亡 3 报告

标本名：HK-2 凋亡 3　　　　检验时间：2024/9/20 11:43

仪器：NovoCyte 451160320945　　　　软件：NovoExpress 1.2.4

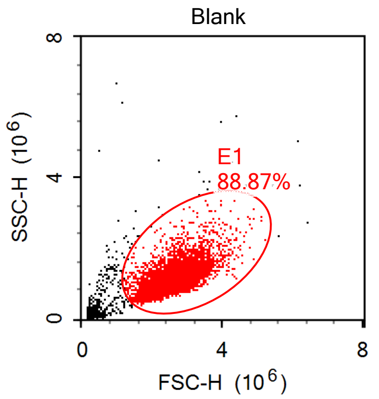

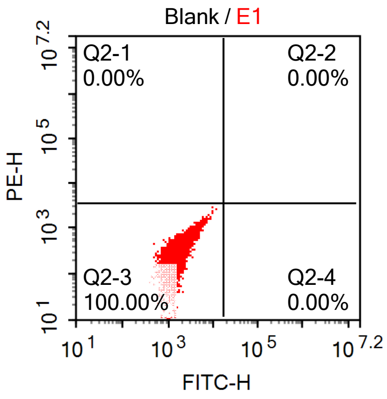

Gate	Count	% All	Mean X	M
All	10,000	100.00%	2,302,196	1,(
E1	8,887	88.87%	2,546,281	1,

Gate	Count	% E1	% All	Mean X	Mean Y	Median X	Median Y
E1	8,887	100.00%	88.87%	1,921	415	1,776	387
Q2-1	0	0.00%	0.00%	0	0	0	0
Q2-2	0	0.00%	0.00%	0	0	0	0
Q2-3	8,887	100.00%	88.87%	1,921	415	1,776	387
Q2-4	0	0.00%	0.00%	0	0	0	0

样本统计表格 - Blank

Gate	Count	% Parent	% All	X	Y	Mean X	Mean Y	Median X	Median Y
All	10,000								
E1	8,887	88.87%	88.87%	FSC-H	SSC-H	2,546,281	1,111,035	2,525,228	1,043,679
Q2-1	0	0.00%	0.00%	FITC-H	PE-H	0	0	0	0
Q2-2	0	0.00%	0.00%	FITC-H	PE-H	0	0	0	0
Q2-3	8,887	100.00%	88.87%	FITC-H	PE-H	1,921	415	1,776	387
Q2-4	0	0.00%	0.00%	FITC-H	PE-H	0	0	0	0

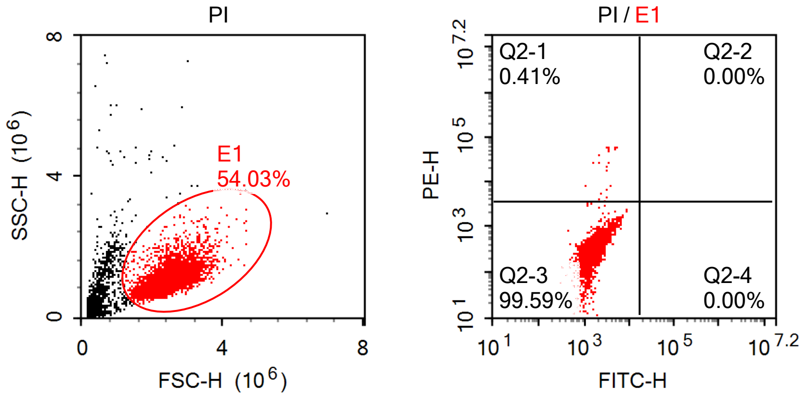

Gate	Count	% All	Mean X	M
All	10,000	100.00%	1,426,346	70
E1	5,403	54.03%	2,439,275	1,

Gate	Count	% E1	% All	Mean X	Mean Y	Median X	Median Y
E1	5,403	100.00%	54.03%	1,858	555	1,733	398
Q2-1	22	0.41%	0.22%	3,101	32,674	3,183	27,315
Q2-2	0	0.00%	0.00%	0	0	0	0
Q2-3	5,381	99.59%	53.81%	1,853	423	1,730	397
Q2-4	0	0.00%	0.00%	0	0	0	0

样本统计表格 - PI

Gate	Count	% Parent	% All	X	Y	Mean X	Mean Y	Median X	Median Y
All	10,000								
E1	5,403	54.03%	54.03%	FSC-H	SSC-H	2,439,275	1,070,653	2,424,405	1,001,494
Q2-1	22	0.41%	0.22%	FITC-H	PE-H	3,101	32,674	3,183	27,315
Q2-2	0	0.00%	0.00%	FITC-H	PE-H	0	0	0	0
Q2-3	5,381	99.59%	53.81%	FITC-H	PE-H	1,853	423	1,730	397
Q2-4	0	0.00%	0.00%	FITC-H	PE-H	0	0	0	0

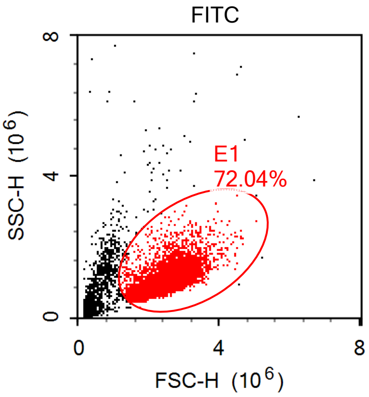

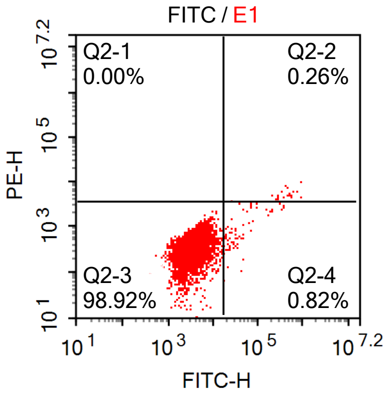

Gate	Count	% All	Mean X	M(
All	10,000	100.00%	1,857,089	90
E1	7,204	72.04%	2,441,531	1,(

Gate	Count	% E1	% All	Mean X	Mean Y	Median X	Median Y
E1	7,204	100.00%	72.04%	5,200	417	3,095	372
Q2-1	0	0.00%	0.00%	0	0	0	0
Q2-2	19	0.26%	0.19%	429,972	5,553	412,044	5,042
Q2-3	7,126	98.92%	71.26%	3,524	396	3,081	371
Q2-4	59	0.82%	0.59%	70,827	1,255	32,704	746

样本统计表格 - FITC

Gate	Count	% Parent	% All	X	Y	Mean X	Mean Y	Median X	Median Y
All	10,000								
└ E1	7,204	72.04%	72.04%	FSC-H	SSC-H	2,441,531	1,065,100	2,424,961	989,428
├ Q2-1	0	0.00%	0.00%	FITC-H	PE-H	0	0	0	0
├ Q2-2	19	0.26%	0.19%	FITC-H	PE-H	429,972	5,553	412,044	5,042
├ Q2-3	7,126	98.92%	71.26%	FITC-H	PE-H	3,524	396	3,081	371
└ Q2-4	59	0.82%	0.59%	FITC-H	PE-H	70,827	1,255	32,704	746

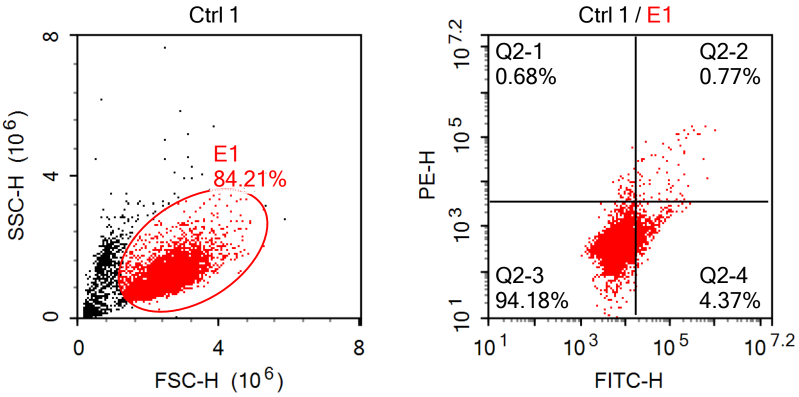

Gate	Count	% All	Mean X	M(
All	10,000	100.00%	2,131,261	1,(
E1	8,421	84.21%	2,434,309	1,(

Gate	Count	% E1	% All	Mean X	Mean Y	Median X	Median Y
E1	8,421	100.00%	84.21%	9,631	909	6,909	482
Q2-1	57	0.68%	0.57%	11,418	8,161	11,353	5,725
Q2-2	65	0.77%	0.65%	148,559	40,165	92,988	14,470
Q2-3	7,931	94.18%	79.31%	7,369	529	6,711	467
Q2-4	368	4.37%	3.68%	33,557	1,058	24,407	870

样本统计表格 - Ctrl 1

Gate	Count	% Parent	% All	X	Y	Mean X	Mean Y	Median X	Median Y
All	10,000								
E1	8,421	84.21%	84.21%	FSC-H	SSC-H	2,434,309	1,084,110	2,409,022	1,008,061
Q2-1	57	0.68%	0.57%	FITC-H	PE-H	11,418	8,161	11,353	5,725
Q2-2	65	0.77%	0.65%	FITC-H	PE-H	148,559	40,165	92,988	14,470
Q2-3	7,931	94.18%	79.31%	FITC-H	PE-H	7,369	529	6,711	467
Q2-4	368	4.37%	3.68%	FITC-H	PE-H	33,557	1,058	24,407	870

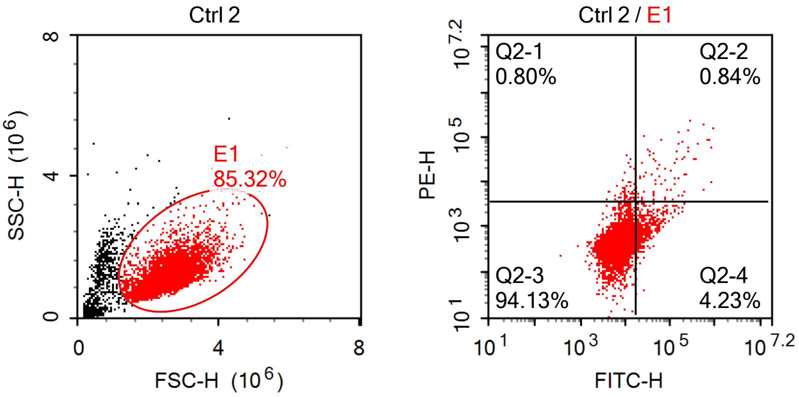

Gate	Count	% All	Mean X	M
All	10,000	100.00%	2,183,788	1,(
E1	8,532	85.32%	2,475,742	1,

Gate	Count	% E1	% All	Mean X	Mean Y	Median X	Median Y
E1	8,532	100.00%	85.32%	9,860	907	6,952	489
Q2-1	68	0.80%	0.68%	11,131	7,421	11,082	5,065
Q2-2	72	0.84%	0.72%	156,091	35,568	71,796	14,597
Q2-3	8,031	94.13%	80.31%	7,380	530	6,723	472
Q2-4	361	4.23%	3.61%	35,631	1,161	26,125	930

样本统计表格 - Ctrl 2

Gate	Count	% Parent	% All	X	Y	Mean X	Mean Y	Median X	Median Y
All	10,000								
E1	8,532	85.32%	85.32%	FSC-H	SSC-H	2,475,742	1,118,240	2,457,931	1,045,620
Q2-1	68	0.80%	0.68%	FITC-H	PE-H	11,131	7,421	11,082	5,065
Q2-2	72	0.84%	0.72%	FITC-H	PE-H	156,091	35,568	71,796	14,597
Q2-3	8,031	94.13%	80.31%	FITC-H	PE-H	7,380	530	6,723	472
Q2-4	361	4.23%	3.61%	FITC-H	PE-H	35,631	1,161	26,125	930

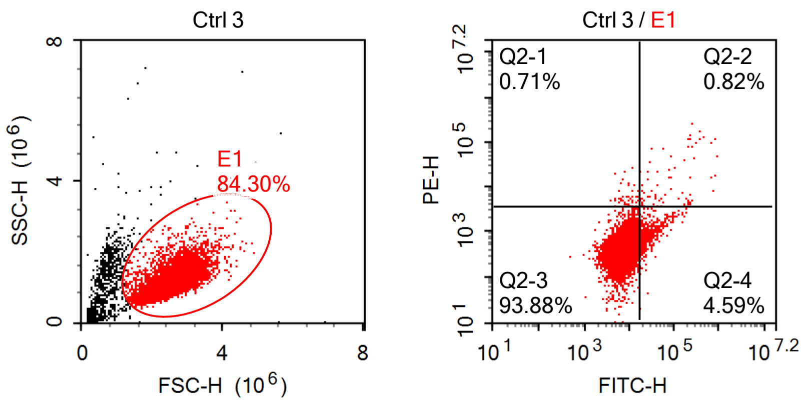

Gate	Count	% All	Mean X	M[illegible]
All	10,000	100.00%	2,164,765	1,[illegible]
E1	8,430	84.30%	2,481,199	1,[illegible]

Gate	Count	% E1	% All	Mean X	Mean Y	Median X	Median Y
E1	8,430	100.00%	84.30%	9,965	923	6,827	488
Q2-1	60	0.71%	0.60%	10,439	6,973	9,700	5,098
Q2-2	69	0.82%	0.69%	180,222	39,587	118,403	8,717
Q2-3	7,914	93.88%	79.14%	7,301	531	6,645	473
Q2-4	387	4.59%	3.87%	34,016	1,103	25,607	951

样本统计表格 - Ctrl 3

Gate	Count	% Parent	% All	X	Y	Mean X	Mean Y	Median X	Median Y
All	10,000								
E1	8,430	84.30%	84.30%	FSC-H	SSC-H	2,481,199	1,115,445	2,460,399	1,044,034
Q2-1	60	0.71%	0.60%	FITC-H	PE-H	10,439	6,973	9,700	5,098
Q2-2	69	0.82%	0.69%	FITC-H	PE-H	180,222	39,587	118,403	8,717
Q2-3	7,914	93.88%	79.14%	FITC-H	PE-H	7,301	531	6,645	473
Q2-4	387	4.59%	3.87%	FITC-H	PE-H	34,016	1,103	25,607	951

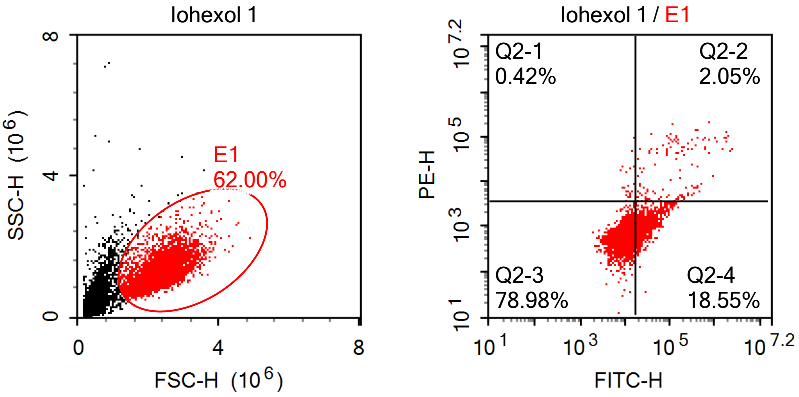

Gate	Count	% All	Mean X	M
All	10,000	100.00%	1,539,104	95
E1	6,200	62.00%	2,265,369	1,:

Gate	Count	% E1	% All	Mean X	Mean Y	Median X	Median Y
E1	6,200	100.00%	62.00%	19,973	1,626	11,997	675
Q2-1	26	0.42%	0.26%	12,712	14,557	12,019	6,084
Q2-2	127	2.05%	1.27%	291,043	40,348	134,600	17,496
Q2-3	4,897	78.98%	48.97%	10,842	665	10,612	617
Q2-4	1,150	18.55%	11.50%	29,084	1,149	24,178	1,014

样本统计表格 - Iohexol 1

Gate	Count	% Parent	% All	X	Y	Mean X	Mean Y	Median X	Median Y
All	10,000								
E1	6,200	62.00%	62.00%	FSC-H	SSC-H	2,265,369	1,276,581	2,241,506	1,215,972
Q2-1	26	0.42%	0.26%	FITC-H	PE-H	12,712	14,557	12,019	6,084
Q2-2	127	2.05%	1.27%	FITC-H	PE-H	291,043	40,348	134,600	17,496
Q2-3	4,897	78.98%	48.97%	FITC-H	PE-H	10,842	665	10,612	617
Q2-4	1,150	18.55%	11.50%	FITC-H	PE-H	29,084	1,149	24,178	1,014

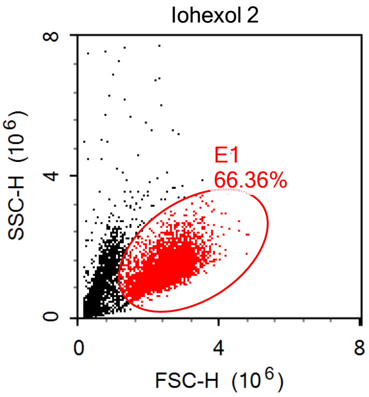

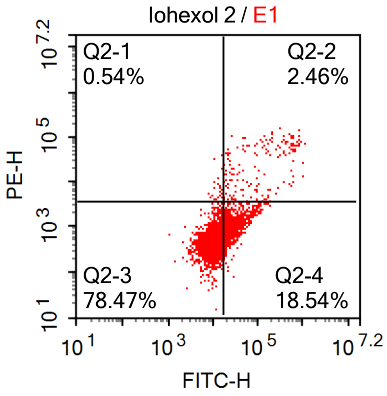

Gate	Count	% All	Mean X	M
All	10,000	100.00%	1,725,306	1,[illegible]
E1	6,636	66.36%	2,376,192	1,[illegible]

Gate	Count	% E1	% All	Mean X	Mean Y	Median X	Median Y
E1	6,636	100.00%	66.36%	20,279	1,773	12,413	639
Q2-1	36	0.54%	0.36%	13,963	9,828	14,859	6,561
Q2-2	163	2.46%	1.63%	242,528	41,694	136,583	37,688
Q2-3	5,207	78.47%	52.07%	11,201	618	10,950	573
Q2-4	1,230	18.54%	12.30%	29,443	1,134	23,463	982

样本统计表格 - Iohexol 2

Gate	Count	% Parent	% All	X	Y	Mean X	Mean Y	Median X	Median Y
All	10,000								
E1	6,636	66.36%	66.36%	FSC-H	SSC-H	2,376,192	1,312,200	2,368,449	1,241,601
Q2-1	36	0.54%	0.36%	FITC-H	PE-H	13,963	9,828	14,859	6,561
Q2-2	163	2.46%	1.63%	FITC-H	PE-H	242,528	41,694	136,583	37,688
Q2-3	5,207	78.47%	52.07%	FITC-H	PE-H	11,201	618	10,950	573
Q2-4	1,230	18.54%	12.30%	FITC-H	PE-H	29,443	1,134	23,463	982

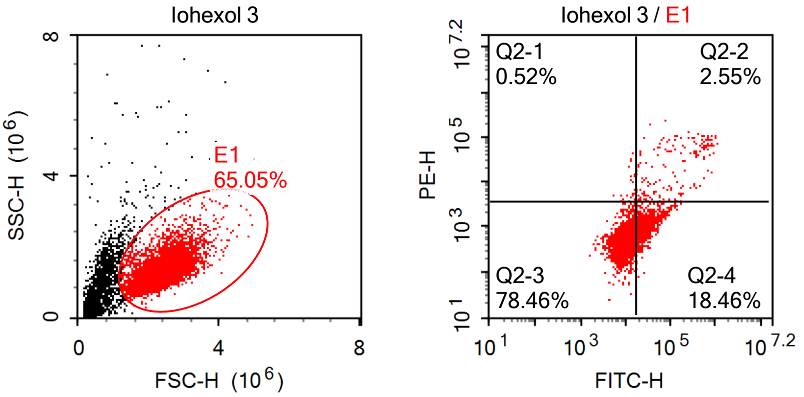

Gate	Count	% All	Mean X	M	Gate	Count	% E1	% All	Mean X	Mean Y	Median X	Median Y
All	10,000	100.00%	1,691,672	1,(	E1	6,505	100.00%	65.05%	20,739	1,848	12,409	646
E1	6,505	65.05%	2,365,287	1,:	Q2-1	34	0.52%	0.34%	13,141	10,566	13,252	5,670
					Q2-2	166	2.55%	1.66%	257,512	42,785	124,317	37,605
					Q2-3	5,104	78.46%	51.04%	11,231	632	11,014	581
					Q2-4	1,201	18.46%	12.01%	28,634	1,109	23,824	992

样本统计表格 - Iohexol 3

Gate	Count	% Parent	% All	X	Y	Mean X	Mean Y	Median X	Median Y
All	10,000								
E1	6,505	65.05%	65.05%	FSC-H	SSC-H	2,365,287	1,317,687	2,352,688	1,247,605
Q2-1	34	0.52%	0.34%	FITC-H	PE-H	13,141	10,566	13,252	5,670
Q2-2	166	2.55%	1.66%	FITC-H	PE-H	257,512	42,785	124,317	37,605
Q2-3	5,104	78.46%	51.04%	FITC-H	PE-H	11,231	632	11,014	581
Q2-4	1,201	18.46%	12.01%	FITC-H	PE-H	28,634	1,109	23,824	992

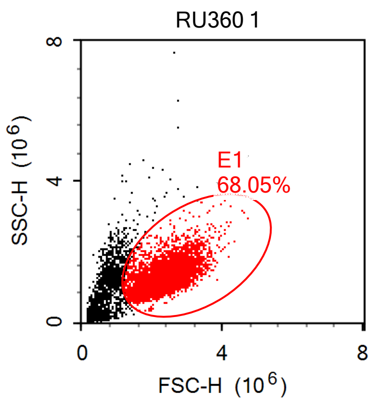

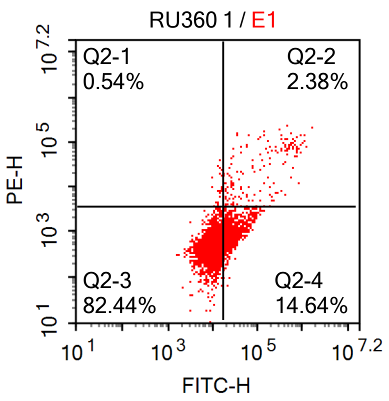

Gate	Count	% All	Mean X	M(	Gate	Count	% E1	% All	Mean X	Mean Y	Median X	Median Y
All	10,000	100.00%	1,708,841	1,(	E1	6,805	100.00%	68.05%	19,564	1,884	11,598	583
E1	6,805	68.05%	2,323,841	1,:	Q2-1	37	0.54%	0.37%	14,281	11,158	13,927	8,016
					Q2-2	162	2.38%	1.62%	264,646	49,775	134,226	29,450
					Q2-3	5,610	82.44%	56.10%	10,891	586	10,658	537
					Q2-4	996	14.64%	9.96%	28,746	1,060	23,347	898

样本统计表格 - RU360 1

Gate	Count	% Parent	% All	X	Y	Mean X	Mean Y	Median X	Median Y
All	10,000								
E1	6,805	68.05%	68.05%	FSC-H	SSC-H	2,323,841	1,278,674	2,297,242	1,215,049
Q2-1	37	0.54%	0.37%	FITC-H	PE-H	14,281	11,158	13,927	8,016
Q2-2	162	2.38%	1.62%	FITC-H	PE-H	264,646	49,775	134,226	29,450
Q2-3	5,610	82.44%	56.10%	FITC-H	PE-H	10,891	586	10,658	537
Q2-4	996	14.64%	9.96%	FITC-H	PE-H	28,746	1,060	23,347	898

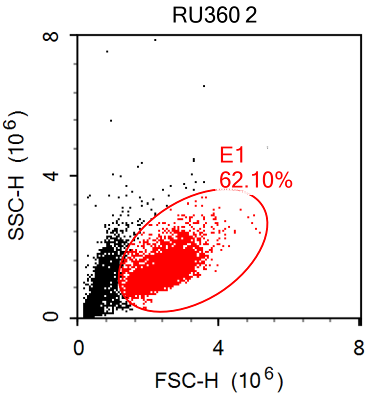

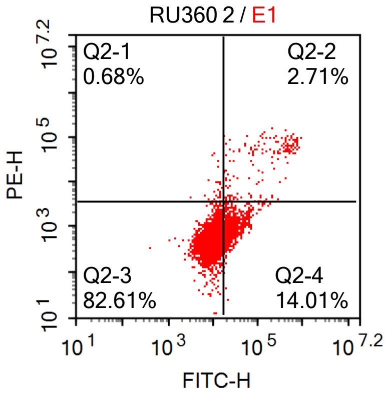

Gate	Count	% All	Mean X	M
All	10,000	100.00%	1,619,176	1,(
E1	6,210	62.10%	2,355,536	1,:

Gate	Count	% E1	% All	Mean X	Mean Y	Median X	Median Y
E1	6,210	100.00%	62.10%	19,485	2,017	11,406	613
Q2-1	42	0.68%	0.42%	12,722	14,041	12,907	5,257
Q2-2	168	2.71%	1.68%	247,283	46,469	159,550	49,061
Q2-3	5,130	82.61%	51.30%	10,684	620	10,430	559
Q2-4	870	14.01%	8.70%	27,717	1,094	23,629	976

样本统计表格 - RU360 2

Gate	Count	% Parent	% All	X	Y	Mean X	Mean Y	Median X	Median Y
All	10,000								
E1	6,210	62.10%	62.10%	FSC-H	SSC-H	2,355,536	1,311,277	2,353,704	1,236,512
Q2-1	42	0.68%	0.42%	FITC-H	PE-H	12,722	14,041	12,907	5,257
Q2-2	168	2.71%	1.68%	FITC-H	PE-H	247,283	46,469	159,550	49,061
Q2-3	5,130	82.61%	51.30%	FITC-H	PE-H	10,684	620	10,430	559
Q2-4	870	14.01%	8.70%	FITC-H	PE-H	27,717	1,094	23,629	976

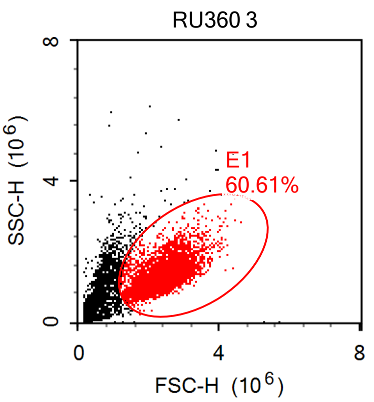

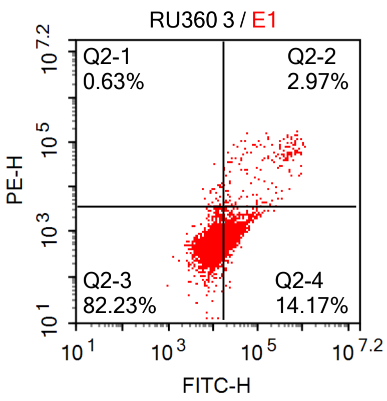

Gate	Count	% All	Mean X	M
All	10,000	100.00%	1,581,160	1,(
E1	6,061	60.61%	2,339,255	1,:

Gate	Count	% E1	% All	Mean X	Mean Y	Median X	Median Y
E1	6,061	100.00%	60.61%	21,716	2,156	11,405	621
Q2-1	38	0.63%	0.38%	12,700	7,804	12,517	4,905
Q2-2	180	2.97%	1.80%	294,502	48,572	219,226	45,397
Q2-3	4,984	82.23%	49.84%	10,615	617	10,277	562
Q2-4	859	14.17%	8.59%	29,360	1,111	24,148	981

样本统计表格 - RU360 3

Gate	Count	% Parent	% All	X	Y	Mean X	Mean Y	Median X	Median Y
All	10,000								
E1	6,061	60.61%	60.61%	FSC-H	SSC-H	2,339,255	1,302,517	2,323,376	1,228,415
Q2-1	38	0.63%	0.38%	FITC-H	PE-H	12,700	7,804	12,517	4,905
Q2-2	180	2.97%	1.80%	FITC-H	PE-H	294,502	48,572	219,226	45,397
Q2-3	4,984	82.23%	49.84%	FITC-H	PE-H	10,615	617	10,277	562
Q2-4	859	14.17%	8.59%	FITC-H	PE-H	29,360	1,111	24,148	981

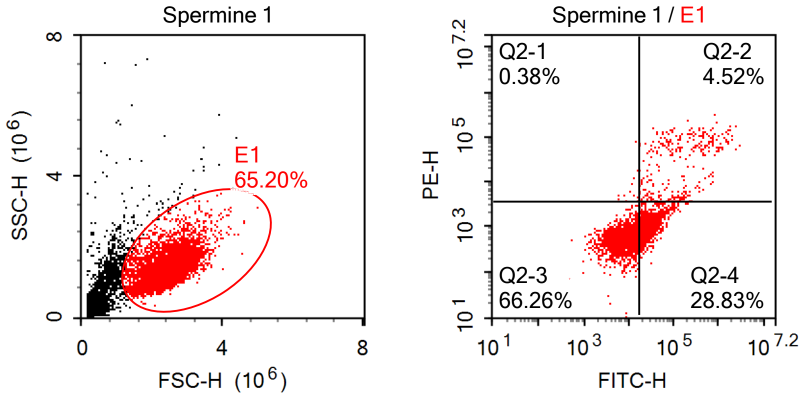

Gate	Count	% All	Mean X	M(
All	10,000	100.00%	1,684,425	1,(
E1	6,520	65.20%	2,369,983	1,:

Gate	Count	% E1	% All	Mean X	Mean Y	Median X	Median Y
E1	6,520	100.00%	65.20%	30,439	3,442	14,298	780
Q2-1	25	0.38%	0.25%	13,979	15,514	15,256	6,402
Q2-2	295	4.52%	2.95%	330,046	57,054	137,275	52,999
Q2-3	4,320	66.26%	43.20%	10,783	688	11,037	653
Q2-4	1,880	28.83%	18.80%	28,813	1,195	24,548	1,083

样本统计表格 - Spermine 1

Gate	Count	% Parent	% All	X	Y	Mean X	Mean Y	Median X	Median Y
All	10,000								
E1	6,520	65.20%	65.20%	FSC-H	SSC-H	2,369,983	1,370,772	2,358,984	1,308,199
Q2-1	25	0.38%	0.25%	FITC-H	PE-H	13,979	15,514	15,256	6,402
Q2-2	295	4.52%	2.95%	FITC-H	PE-H	330,046	57,054	137,275	52,999
Q2-3	4,320	66.26%	43.20%	FITC-H	PE-H	10,783	688	11,037	653
Q2-4	1,880	28.83%	18.80%	FITC-H	PE-H	28,813	1,195	24,548	1,083

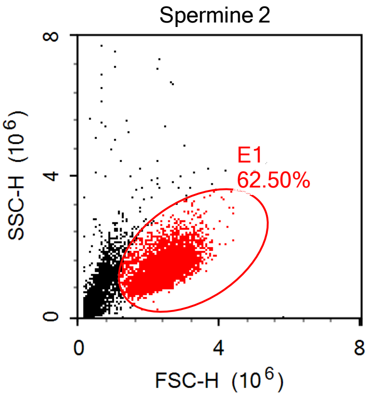

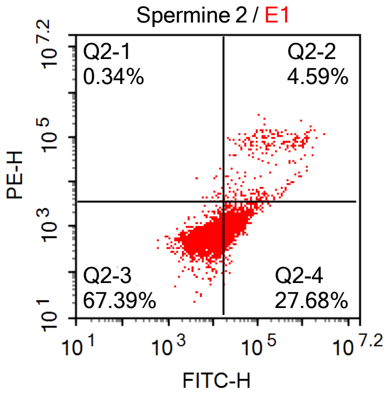

Gate	Count	% All	Mean X	M[illegible]
All	10,000	100.00%	1,619,909	1,[illegible]
E1	6,250	62.50%	2,351,721	1,[illegible]

Gate	Count	% E1	% All	Mean X	Mean Y	Median X	Median Y
E1	6,250	100.00%	62.50%	29,496	3,336	13,995	777
Q2-1	21	0.34%	0.21%	11,533	6,589	11,103	4,749
Q2-2	287	4.59%	2.87%	313,401	54,855	138,114	54,393
Q2-3	4,212	67.39%	42.12%	10,607	691	10,850	655
Q2-4	1,730	27.68%	17.30%	28,605	1,190	25,045	1,081

样本统计表格 - Spermine 2

Gate	Count	% Parent	% All	X	Y	Mean X	Mean Y	Median X	Median Y
All	10,000								
E1	6,250	62.50%	62.50%	FSC-H	SSC-H	2,351,721	1,355,858	2,340,934	1,291,583
Q2-1	21	0.34%	0.21%	FITC-H	PE-H	11,533	6,589	11,103	4,749
Q2-2	287	4.59%	2.87%	FITC-H	PE-H	313,401	54,855	138,114	54,393
Q2-3	4,212	67.39%	42.12%	FITC-H	PE-H	10,607	691	10,850	655
Q2-4	1,730	27.68%	17.30%	FITC-H	PE-H	28,605	1,190	25,045	1,081

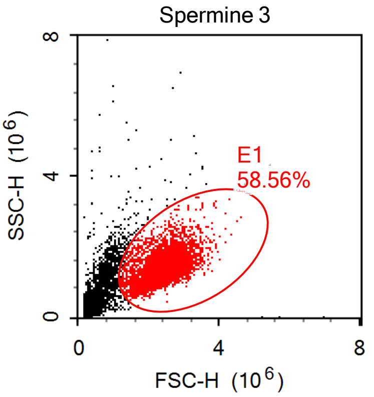

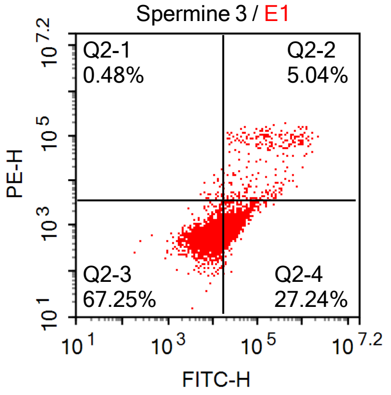

Gate	Count	% All	Mean X	M(
All	10,000	100.00%	1,528,149	1,(
E1	5,856	58.56%	2,333,190	1,:

Gate	Count	% E1	% All	Mean X	Mean Y	Median X	Median Y
E1	5,856	100.00%	58.56%	29,496	3,566	14,282	780
Q2-1	28	0.48%	0.28%	11,932	7,016	12,030	5,302
Q2-2	295	5.04%	2.95%	286,804	54,401	139,621	55,892
Q2-3	3,938	67.25%	39.38%	10,809	701	11,084	658
Q2-4	1,595	27.24%	15.95%	28,353	1,177	24,205	1,073

样本统计表格 - Spermine 3

Gate	Count	% Parent	% All	X	Y	Mean X	Mean Y	Median X	Median Y
All	10,000								
└ E1	5,856	58.56%	58.56%	FSC-H	SSC-H	2,333,190	1,340,030	2,319,724	1,277,418
├ Q2-1	28	0.48%	0.28%	FITC-H	PE-H	11,932	7,016	12,030	5,302
├ Q2-2	295	5.04%	2.95%	FITC-H	PE-H	286,804	54,401	139,621	55,892
├ Q2-3	3,938	67.25%	39.38%	FITC-H	PE-H	10,809	701	11,084	658
└ Q2-4	1,595	27.24%	15.95%	FITC-H	PE-H	28,353	1,177	24,205	1,073
